# Exploring the Genes of Yerba Mate (*Ilex paraguariensis* A. St.-Hil.) by NGS and *De Novo* Transcriptome Assembly

**DOI:** 10.1371/journal.pone.0109835

**Published:** 2014-10-16

**Authors:** Humberto J. Debat, Mauro Grabiele, Patricia M. Aguilera, Rosana E. Bubillo, Mónica B. Otegui, Daniel A. Ducasse, Pedro D. Zapata, Dardo A. Marti

**Affiliations:** 1 Instituto de Patología Vegetal, Centro de Investigaciones Agropecuarias, Instituto Nacional de Tecnología Agropecuaria (IPAVE-CIAP-INTA), Córdoba, Argentina; 2 Instituto de Biología Subtropical, Universidad Nacional de Misiones (IBS-UNaM-CONICET), Posadas, Misiones, Argentina; 3 Estación Experimental Cerro Azul, Instituto Nacional de Tecnología Agropecuaria (EEA Cerro Azul-INTA), Misiones, Argentina; 4 Instituto de Biotecnología de Misiones, Facultad de Ciencias Exactas Químicas y Naturales, Universidad Nacional de Misiones (INBIOMIS-FCEQyN-UNaM), Misiones, Argentina; Institute of Genetics and Developmental Biology, Chinese Academy of Sciences, China

## Abstract

Yerba mate (*Ilex paraguariensis* A. St.-Hil.) is an important subtropical tree crop cultivated on 326,000 ha in Argentina, Brazil and Paraguay, with a total yield production of more than 1,000,000 t. Yerba mate presents a strong limitation regarding sequence information. The NCBI GenBank lacks an EST database of yerba mate and depicts only 80 DNA sequences, mostly uncharacterized. In this scenario, in order to elucidate the yerba mate gene landscape by means of NGS, we explored and discovered a vast collection of *I. paraguariensis* transcripts. Total RNA from *I. paraguariensis* was sequenced by Illumina HiSeq-2000 obtaining 72,031,388 pair-end 100 bp sequences. High quality reads were *de novo* assembled into 44,907 transcripts encompassing 40 million bases with an estimated coverage of 180X. Multiple sequence analysis allowed us to predict that yerba mate contains ∼32,355 genes and 12,551 gene variants or isoforms. We identified and categorized members of more than 100 metabolic pathways. Overall, we have identified ∼1,000 putative transcription factors, genes involved in heat and oxidative stress, pathogen response, as well as disease resistance and hormone response. We have also identified, based in sequence homology searches, novel transcripts related to osmotic, drought, salinity and cold stress, senescence and early flowering. We have also pinpointed several members of the gene silencing pathway, and characterized the silencing effector Argonaute1. We predicted a diverse supply of putative microRNA precursors involved in developmental processes. We present here the first draft of the transcribed genomes of the yerba mate chloroplast and mitochondrion. The putative sequence and predicted structure of the caffeine synthase of yerba mate is presented. Moreover, we provide a collection of over 10,800 SSR accessible to the scientific community interested in yerba mate genetic improvement. This contribution broadly expands the limited knowledge of yerba mate genes, and is presented as the first genomic resource of this important crop.

## Introduction

*Ilex paraguariensis* (Aquifoliaceae) is a dioecious crop tree native to the subtropical rainforest of Northeastern Argentina, Southwestern Brazil and Eastern Paraguay, where it is widely cultivated [1]. This evergreen holly is colloquially known as “yerba mate” or “erva mate” as it is mainly consumed as a nutritional and stimulant beverage named “mate”, a type of hot infusion made from dried milled leaves and twigs of *I. paraguariensis*. Yerba mate is also extensively used to prepare infusions, concoctions and quenchers with similar purposes and, more recently as admixture in ice creams, candies and energy drinks [2], as well as in dyes, cosmetics and spa ingredients [3]. Antioxidant, anti-inflammatory, antimutagenic and lipid-lowering properties have been reported in *I. paraguariensis* [2], leading to an increasing interest in this tree. Yerba mate is an economically important crop cultivated and produced on a total area of more than 326,000 ha [4], [5]. Argentina is the main producer with a total yield of over 880,000 t, representing ∼85% of world-wide yerba mate production [6]. About 15% of total yerba mate production is exported to South American, European and Asian markets [7]. Besides the agricultural and economic importance of yerba mate, it is worth noting its profound and omnipresent influence in Latin American socio-cultural dynamics. Yerba mate widespread consumption embraces and extends ubiquitously, pervasively reaching every economic and cultural niche in South America [8]–[11]. To emphasize the relevance of yerba mate in South American tradition and its introduction and dissemination particularly in Argentinean culture, in 2009 a 5,000 people survey projected that while 81% of the Argentinean population consumes coffee, a striking 98% of the population consumes yerba mate [12].

Genetic improvement of *I. paraguariensis* has been limited by several factors. Agronomic evaluation and selection programs have been performed essentially to improve yield in this crop [13], [1]. However, apart from this trait, very little is known about agronomically important loci on the limited available germplasm of this species. Moreover, yerba mate plants cannot be recognized as male or female prior to their first blooming, which takes 3 to 10 years post seed germination [14], delaying the selection of parentals for breeding purposes. Likewise, knowledge of sequences of interesting genes is needed to achieve genetic improvement based on molecular tools, a valuable information that is lacking in yerba mate. Currently, merely ∼80 sequences originated from *I. paraguariensis* are available in the National Center for Biotechnology Information (NCBI) database, most of them corresponding to microsatellites. In addition, genetic information in the genus is scarce, annotated sequences are virtually inexistent and no expressed sequence tags (ESTs) libraries have been generated so far.

Massively parallel sequencing of RNA (RNA-Seq) is an efficient way to characterize the transcriptional landscape of a species and reveal its complexity [15]. It allows to investigate the transcriptome composition and expression and, in this direction, to explore and reveal the expressed profile of a defined organism [16]. This next-generation sequencing technology (NGS) is a simple and fast tool to analyze the transcriptome since it requires neither cloning library of the cDNAs nor any *a priori* knowledge of the species. Instead of this, RNA-Seq technology generates millions of short direct cDNA reads which are subsequently assembled to construct transcripts [15]. *De novo* transcriptome assembly is suitable in order to reconstruct full length transcripts from these short reads in organisms without a sequenced genome as reference. The most advanced algorithms to achieve this strategy consists in efficiently constructing and analyzing sets of *de Bruijn* graphs to construct and assemble transcripts and requires a great amount of parallel sequence short reads provided by high throughput sequencing technology [17].

This study presents the first analysis of the *I. paraguariensis* transcriptome. We employed the Illumina total RNA-Seq sequencing method to generate 72,031,388 pair-end 100 bp sequence reads. The obtained high quality reads were *de novo* assembled into 44,907 primary transcripts encompassing 40 million bases with an estimated coverage of 180X. Multiple sequence analysis allowed us to predict that yerba mate contains about 32,355 genes and 12,551 gene variants or isoforms. An initial analysis of these genes allowed us to identify and categorize members of more than 100 metabolic pathways. The transcriptome characterization of *I. paraguariensis* generated from our study is a very useful tool derived from a convenient and exhaustive approach of annotation and discovery of genes of several major metabolic pathways. The vast amount of information obtained would encourage and serve as reliable source in the path to the discovery of biologically and agronomically important traits, as well as for molecular markers development, gene mapping, analysis of genetic diversity and selective breeding in yerba mate.

## Results and Discussion

### RNA sequencing analysis and transcriptome *de novo* assembly

In order to shed light on the transcriptional landscape of yerba mate, total RNA was extracted from pooled leaves of *I. paraguariensis* breeding line Pg538 from INTA EEA-Cerro Azul, Misiones, Argentina. After initial quality controls the isolated RNA was sequenced by the Illumina HiSeq-2000 platform. A total of 72,031,388 100 bp pair-end reads were obtained (**Table 1**). An analysis of the sequencing run indicated an absence of cycle-wise multiplied calls of the same nt, an average high quality of Q = 36.3, a lack of positional biases in the call frequency for each base and a typical unimodal distribution of quality average and Kmer enrichment frequency (**Figure S1 a-e**).

**Table 1:** Yerba mate Illumina HiSeq-2000 sequencing run statistics.

Sequencing stats	*Ilex paraguariensis* RNA-seq
*Total Bases*	7,275,170,188
*Read Count*	72,031,388
*GC (%)*	45,38
*N (%)*	0,027
*Q20 (%)*	98,21
*Q30 (%)*	94,99
*Average Q*	36,3
*read length*	100 nt×2

Recently, towards the identification of phosphate starvation-responsive genes in wheat (*Triticum aestivum*), a similar NGS approach was employed based in *de novo* assembly of 73,8 million reads from RNA-seq libraries [18]. The extension of this sequencing process, similar to that of our study, was effective and sufficient to generate a comprehensive transcriptome in wheat in the absence of reference genome information.

After quality filtering, the sequencing reads were assembled with the Trinity software [17] and a transcriptome of 44,907 assembled sequences was obtained. A quality analysis of the assembly suggested a typical distribution and coverage of GC content, a lack of positional kmer enrichment, a high percentage coverage consistent with the sequence length distribution and a regular positional nucleotide contribution in the assembled transcripts (**Figure S2 a-f**). The transcriptome covers 39,969,375 bp, with a mean contig length of 890 bp, N50 of 1,430 bp and 8,353 sequences with a length of over the N50. Our *de novo* assembly utterly indicates that yerba mate presents an estimate of 32,356 genes and 12,551 gene variants or isoforms (**Table 2**).

**Table 2:** Yerba mate Trinity *de novo* assembled transcriptome statistics.

Assembly	*Ilex paraguariensis*
method	Trinity k25
assembled seq.	44,907
unigenes	44,906
gene families	32,355
gene variants	12,551
n: 100	44,907
n: N50	8,353
min	201 bp
median	544 pb
mean	890 bp
N50	1,430 bp
max	15,716 bp
sum	39,969,375 bp

### Evaluating the yerba mate transcriptome by DEG analysis

It has been proposed that a comprehensive catalog of essential genes may constitute a minimal genome, forming a set of functional modules, which play key roles in eukaryotic metabolism [19]. In that direction, a catalog of over 356 genes has been assigned as essential in the cruciferous plant *Arabidopsis thaliana* [20]. To assess and estimate the “completeness” of the assembled yerba mate transcriptome, a DEG (Database of Essential Genes) analysis was performed (**Data S1**). Exploring the genes of yerba mate we observed that the orthologs of 97.2% of the *A. thaliana* essential genes were present in our assembled transcriptome. In a recent study [21] a highly representative *Nicotiana benthamiana* transcriptome was evaluated under a similar platform depicted as “Core eukaryotic genes dataset” (CEGMA) [22], which includes a widely conserved set of 248 eukaryotic proteins. In this CEGMA analysis, 95% of the core proteins were identified in the *N. benthamiana* transcriptome. In this scenario we consider that the yerba mate DEG score is indicative of an overall representative status of the transcript library produced.

### Characterization and functional annotation of yerba mate transcripts

**Figure 1:**
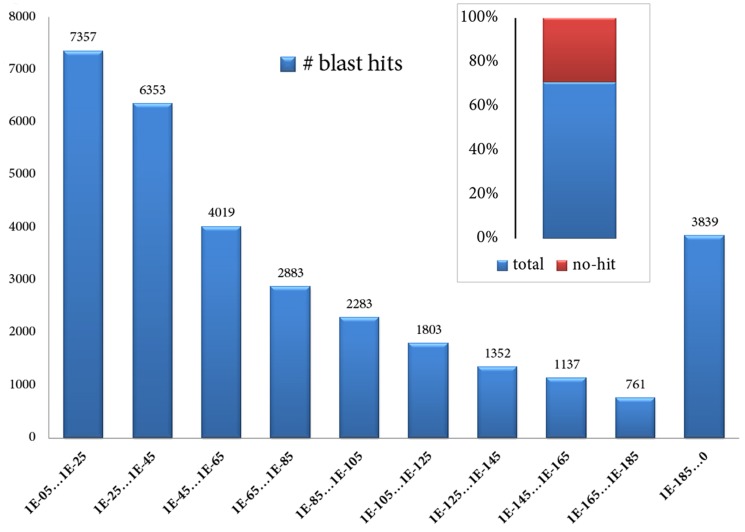
BLASTX hits E-value distribution of assembled transcripts to TAIR *Arabidopsis thaliana* proteins. Using a cut-off value of 10E-05, over 77% of transcripts (31,787 contigs) attained a blast hit based in identity conservation.

The assembled transcriptome of *I. paraguariensis* was sequentially subjected to homology searches using the BLASTX platform against the UniProt *viridiplantae* database (**Table S3**) and TAIR. BLASTX hits E-value distribution of assembled transcripts to TAIR *A. thaliana* proteins is presented in **Figure 1**. Using a cut-off value of 10E-05, over 77% of transcripts (31,787) attained a blast hit based on identity conservation (**Table S4**).

**Figure 2:**
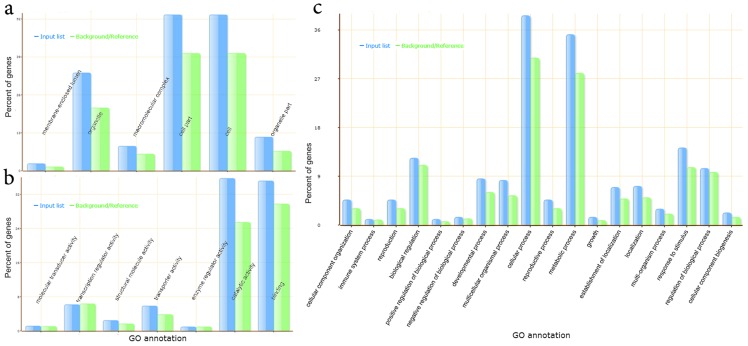
GO annotations obtained for the yerba mate transcriptome. Categorization by cell component (a), molecular function (b), and biological process (c). *Ilex paraguariensis* GO percentages are based on 31,787 BLASTX hits (blue), and the *Arabidopsis thaliana* transcriptome was employed as background (green).

The obtained BLASTX hits were subsequently imported into the Blast2GO software, the KAAS server, and the agriGO platform, where gene ontology annotation, metabolic pathway profiling and GO categorization were performed. Over 217,655 GO term tags were identified in the yerba mate transcriptome, of which 4,341 were associated to KEGG ids corresponding to 315 KEGG maps (**Table S2**). An initial sorting of the yerba mate sequences based on GO terms is presented in **Figure 2** using the *A. thaliana* transcriptome as background. The assembled transcripts are categorized by cell component (**Figure 2a**) where an enrichment in organelle and cell structural components was observed, molecular function (**Figure 2b**) that showed an elevated percentage of catalytic and binding representatives, and biological process (**Figure 2c**) where the distribution of sequences followed the typical frequency observed in *Arabidopsis*. A closer analysis of the GO associated yerba mate sequences (**Table S5**) by semantic similarity-based scatterplots representations and treemaps (**Figure S3** and **Figure S4**) highlighted several terms based on p-values (circle size, rectangle size) associated to the GO enriched categories, such as growth, methylation and reproductive structure development on biological process (**a**), chloroplast, membrane enclosed lumen and ubiquitin ligase complex on cellular component (**b**) and chlorophyll binding, methyltransferase activity and sequence specific DNA binding on molecular function (**c**). An exhaustive analysis of the GO terms is presented as AgriGO generated plots of GO enrichment, significance and relationships in yerba mate based in biological process (**Figure S5**), cellular component (**Figure S6**) and molecular function (**Figure S7**). A 166 catalog of KEGG drawn maps representing the gene members of the yerba mate transcriptome extensively associated with numerous metabolic pathways is presented in **Data S2**. In order to explore the yerba mate genes, we approached a categorization of transcripts based in BLASTX. Overall, we have identified over 1,000 putative transcription factors of yerba mate (**Table S1**), 50 transcripts involved in heat-stress, more than 200 oxidative stress responsive putative genes, 30 transcripts associated with pathogen response, a significant number of transcripts associated with ribosome constituents, ribosome processing, trafficking, rRNA maturation, and ribosome assembly (**Figure S8**), as well as 60 assembled transcripts involved in disease resistance and 150 transcripts probably engaged in hormone response (**Figure S9**). We have also identified nearly 100 transcripts related to osmotic, drought, salinity and cold stresses, senescence, early flowering, and biosynthesis of sugars, flavonoids, carotenoids and chlorophyll (**Table S3**).

When compared with other plant species reported by previous studies that used the Illumina system and the Trinity software, the quality of our transcriptome sequence shows to be significantly high, which is evidenced in several aspects. First, the average length of the unigenes we observed is 890 bp which is comparable to that of chili pepper (*Capsicum frutescens*, 712 bp) [23], ramie (*Boehmeria nivea*, 824 bp) [24], *Salvia splendens* (779 bp and 812 bp for two different strains) [25] and peanut (*Arachis hypogaea*, 751 bp) [26] transcriptome sequences. Second, approximately 77% of the genes discovered in this study were successfully annotated for their putative functions. Previous reports of annotated genes in species such as *C. frutescens* (72.33%) [23], *Boehmeria nivea* (77.70%) [24], barnyardgrass (*Echinochloa crus-galli*, 57.45%) [27] and sugarcane varieties (*Saccharum officinarum* x *S. spontaneum*, 49.06%) [28] further support the notion of our assembly dataset being a fair representation of the yerba mate transcriptome.

### Prediction of yerba mate SSRs

**Figure 3:**
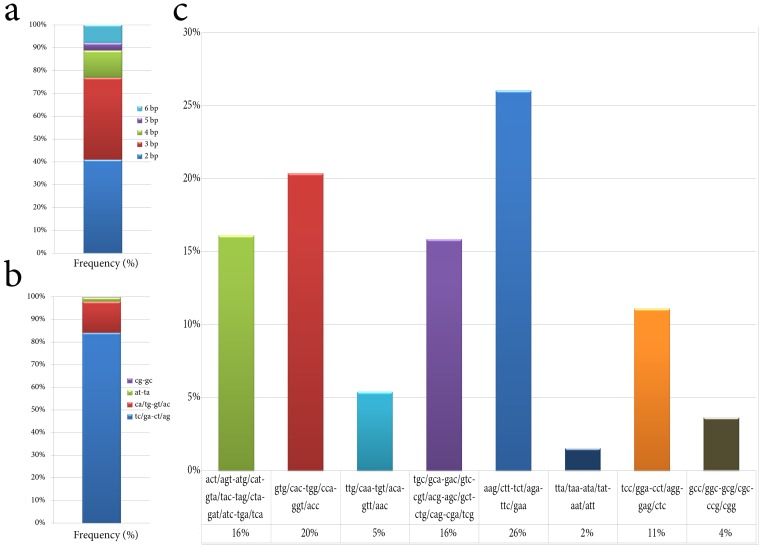
Proportion and frequencies of predicted SSRs in *Ilex paraguariensis* transcriptome. (a) Proportion of SSR predicted in yerba mate transcriptome categorized by k-mer length. (b) ct/ag-tc/ga account for 84% of di-nucleotide SSRs found in yerba mate. (c) Frecuency of tri-nucleotide SSRs predicted in yerba mate. With over 26% of the hits, aag/ctt-tct/aga-ttc/gaa are the most common SSR found in *Ilex paraguariensis*.

Simple sequence repeat (SSR) markers are well-known and widely used as valuable tools for assessing genetic diversity. SSRs are useful in the development of genetic maps, comparative genomics and marker-assisted selection breeding [28]. Thus, in parallel, the yerba mate transcripts library was comprehensively analyzed in search of SSRs. A total of 10,813 SSRs were identified in 8,449 sequences along the transcriptome. We analyzed our data and *in silico* predicted SSRs using 6,4,3,3,3 motifs repeats criteria for di-, tri-, tetra-, penta-, and hexa-nucleotides SSRs. In this context, the 2 nt motif repeats represented 40.9% of total SSRs found, while 3 nt motif repeats constituted roughly a 35.8% of total SSRs (**Figure 3a**). The most represented SSR corresponded to 2 nt motif ct/ag-tc/ga (**Figure 3b**) which encompassed over 84% of the 4,429 SSRs of 2 bp motif (**Table S11**). Among the tri-nucleotide motif repeats, with over 26% of the hits, aag/ctt-tct/aga-ttc/gaa are the most common SSR found in *I. paraguariensis* (**Figure 3c**). In most plant transcriptome studies, tri-nucleotide are the most frequent SSRs. However, the repeat motif abundance in plant transcriptomes is affected by the *in silico* determination of SSRs prediction criteria. For instance, several studies consider di-, tri-, tetra- penta- and hexa-nucleotides when diverse motif repeats are present, i.e. 6,5,4,4,4 in *Salvia splendens* [25], 6,5,5,5,5 in *Saccharum* spp [28], 6,5,5,4,4 in *Capsicum frutescens* [23], 6,4,3,3,3 in *Curcuma longa* [29], 4,4,4,4,4 in *Ipomoea batatas* [30]. In order to be consistent with the literature, we have *in silico* predicted SSRs using 6,4,3,3,3 motifs repeats criteria. In this background, di-nucleotides were the most representative SSR species, followed by tri-nucleotides. This non-standard distribution has also been described for *Salvia splendes* [25] with 39.9%/29.3% di- and tri-nucleotide frequencies, respectively, sweet potato with 43.3%/42.4% [30], rubber tree with 38%/34% [31] and several other plants such as cucumber [32], sesame [33], kiwi [34] and coffee [35] where di-nucleotides are also the most represented SSR species.

### Transposable elements discovery

Several transposable elements (TE) were identified by sequence homology in yerba mate. In the literature, only a few sequences have been recently reported corresponding to yerba mate TE fragments obtained by DNA based methods [14], [36], hence this is the first report describing actively yerba mate TEs. As expected for a transcriptional library, most of the sequences corresponded to Retro-Transposon elements, mainly Group Antigen polyprotein (GAG-Pol), reverse-transcriptase and RNAse H domain hits of Gypsy-like and Copia-like retro-elements. Also, a handful of Non-LTR retro-elements were identified and a few CACTA, En/Spm sub-class Transposons. The predicted repetitive elements were explored in detail, and representative results obtained by the NCBI conserved domain search web-service are presented as graphical summaries depicting typical TE domains such as Reverse Transcriptase domain in Gypsy-like elements and Transposase domain in En/Spm Transposons, of several yerba mate putative TEs (**Table S12**).

### Organelles draft genome *de novo* assembly and analysis

**Figure 4:**
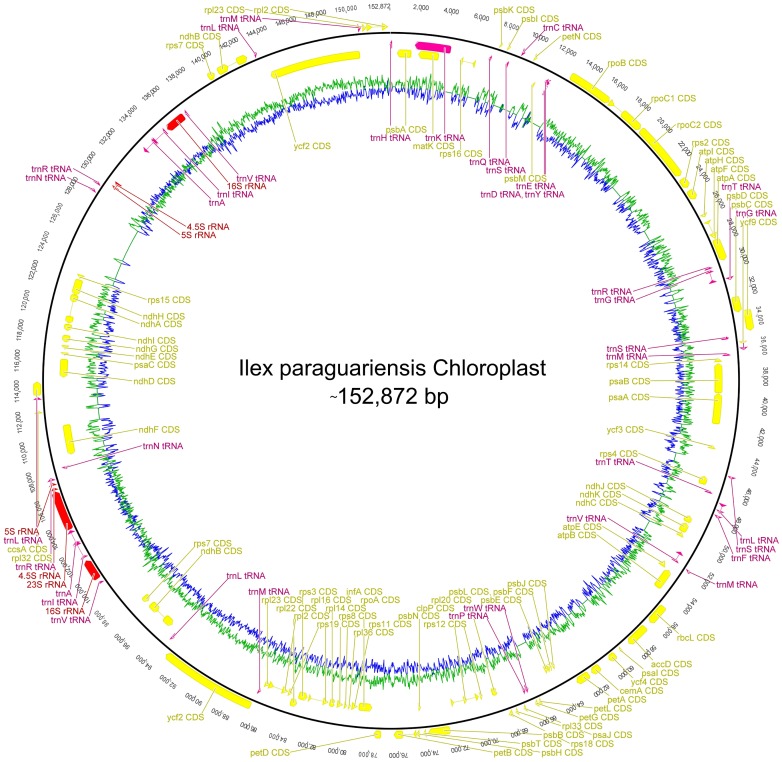
*Ilex paraguariensis* chloroplast is predicted to be ∼152,872 bp long, consisting in 51.6% of coding sequences, representing 83 protein coding genes (yellow), 37 transfer RNA genes (pink) and 7 ribosome RNA genes (red). The 83 protein coding genes include several ribosomal proteins, constituents of photosystem I & II, NADH dehydrogenases and ATP synthases among others.

Recent NGS based studies have emphasized in the abundance and wide extension of chloroplast and mitochondrial transcripts, postulating that most of the organelles genomic DNA is actively transcribed in plants [37]–[40]. In this scenario we surveyed the yerba mate transcriptome in order to generate a draft genome of both organelles based in RNA transcripts and sequence similarity to reference organelle sequences of slightly related plant species. Illumina reads were relaxedly mapped to a *Lactuca sativa* chloroplast sequence and a total of 10,798,227 reads comprised and sustained a high coverage library that was assembled into a consensus sequence draft of the yerba mate chloroplast (**Table 3**). The assembled *I. paraguariensis* chloroplast is predicted to be ∼152,872 bp long, consisting in 51.6% of coding sequences, representing 83 protein coding genes, 37 transfer RNA genes and 7 ribosome RNA genes (**Figure 4**). The 83 protein coding genes included several ribosomal proteins, constituents of photosystem I & II, NADH dehydrogenases and ATP synthases among others (**Table 4** and **Table S6**). A sequence alignment of the *L. sativa* chloroplast complete sequence and yerba mate draft chloroplast shows extensive identity, in some regions exceeding 90%, particularly at gene transcripts such as 16 s rRNA, 23 s rRNA and several transfer RNA genes (**Figure S10**). Mapping of *I. paraguariensis* assembled transcripts to the chloroplast sequence draft shows an extensive and pervasive coverage (**Figure S11**). The assembled draft was subjected to microsatellite discovery (**Table S8**) and a total of 94 SSRs were identified, consisting mainly of 6 bp motif (57.5% of total SSRs).

**Table 3:** General features of Yerba mate draft assembled organelles.

*Feature*	Chloroplast	Mitochondrion
*Genome size (bp) estimated*	∼150,872	∼301,093
*contig coverage (bp)*	118,064	90,151
*total mapped 100 bp reads*	10,798,227	1,265,566
*GC content (%)*	56.06	42.06
*Coding sequences*	127	43
*Gene content (%)*	51.6	9.03
*No. of protein-coding gene*	83	26
*No. of introns*	17	0
*No. of tRNA genes*	37	14
*No. of rRNA operons*	7	3
*Sequence repeat*	94	69

**Table 4:** Yerba mate chloroplast encoded genes by category.

*Chloroplast gene category*	Gene name
*Photosystem I*	psaA, psaB, psaC, psaI, psaJ
*Photosystem II*	q
*Cytochrome b/f complex*	petA, petB, petD, petG, petL, petN
*ATP synthase*	atpA, atpB, atpE, atpF, atpH, atpI
*NADH dehydrogenase*	ndhA, ndhB, ndhC, ndhD, ndhE, ndhF, ndhG, ndhH, ndhI, ndhJ, ndhK
*RubisCO large subunit*	rbcL
*RNA polymerase*	rpoA, rpoB, rpoC1, rpoC2
*Ribosomal proteins (SSU)*	rps2, rps3, rps4, rps7(2), rps8, rps11, rps12, rps14, rps15, rps16, rps18, rps19
*Ribosomal proteins (LSU)*	rpl2(2), rpl14, rpl16, rpl20, rpl22, rpl23(2), rpl32, rpl33, rpl36
*Other genes*	clpP, matK, accD, ccs1, ccsA, infA, cemA
*hypothetical*	ycf2(2), ycf3, ycf4, ycf9
*Transfer RNAs*	trnA-UGC(2), trnC-GCA, trnD-GUC, trnE-UUC, trnF-GAA, trnG-UCC(2), trnH-GUG, trnI-CAU, trnI-GAU, trnK-UUU, trnL-CAA, trnL-GAG, trnL-UAA, trnL-UAG, trnM-CAU(4), trnN-GUU(2), trnP-UGG, trnQ-UUG, trnR-ACG(3), trnS-GCU(2), trnS-UGA, trnT-GGU(2), trnV-GAC(2), trnV-UAC, trnW-CCA, trnY-GUA
*Ribosomal RNAs*	rRNA 4.5 s(2), rRNA 5 s(2), rRNA 16 s(2), rRNA 23 s

A similar approach was employed in order to envisage a mitochondrial genome draft of yerba mate. A total of 1,265,566 Illumina reads were mapped to the *Helianthus annuus* mitochondrial genome sequence. In this case, most mapped reads corresponded mainly to the gene coding regions, and the assembled draft extended at about a third of the total predicted genome (**Table 3**). A sequence alignment of sunflower mitochondrial complete sequence and yerba mate mitochondrial sequence consensus (**Figure S12**) presented high identity at most of the 43 coding sequences that corresponded to protein coding genes such as Complex I NADH dehydrogenases, Complex V ATP synthases and ribosomal proteins (SSU and LSU), transfer RNA genes and ribosome RNA genes (**Table 5** **, Table S7**).

**Table 5:** Yerba mate mitochondrial encoded genes by category.

*Mitochondrial gene category*	Gene name
*Complex I (NADH dehydrogenase)*	nad3, nad4, nad5, nad6, nad8, nad9
*Complex III (cytochrome c reductase)*	cob
*Complex IV (cytochrome c oxidase)*	coxI, coxIII
*Complex V (ATP synthase)*	atp1, atp4, atp6, atp8, atp9
*Cytochrome c biogenesis*	ccmB, ccmC, ccmFc, ccmFn(2)
*Ribosomal proteins (SSU)*	rps4, rps12, rps13
*Ribosomal proteins (LSU)*	rpl5, rpl10, rpl16
*Maturases*	matR
*Other genes*	orf873
Transfer RNAs	trnC-GCA, trnD-GUC, trnE-UUC, trnF-GAA, trnG-GCC, trnH-GUG, trnK-UUU, trnL-UAA, trnM-CAU(2), trnN-GUU, trnP-UGG, trnQ-UUG, trnS-GCU, trnW-CCA, trnY-GUA
*Ribosomal RNAs*	rrn5, rrn18, rrn26

A *Mauve* alignment, which is preferred for rearranged genome sequences [41], was performed with the yerba mate and sunflower mitochondrial sequences. The higher identity, mostly confined to gene encoding regions, is represented hierarchically from white to red. The consensus *I. paraguariensis* sequence conserved most of the *Helianthus* gene annotations. As an example, the consensus sequence of *I. paraguariensis* at 76,000 bp coordinates presented high identity to the 230,000 bp coordinates of sunflower, corresponding to the ccmFn coding sequence (**Figure S13**). The assembled yerba mate consensus sequence was subjected to microsatellite discovery (**Table S9**) and a total of 69 SSRs were identified, consisting mainly of 6 bp motif (69.8% of total predicted SSRs).

### The yerba mate RNA silencing and degradation pathway

**Figure 5:**
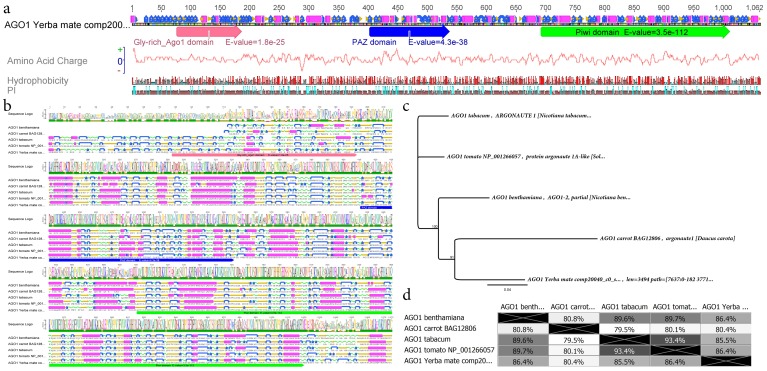
Yerba mate *Argonaute 1* (AGO1): characterization of the catalytic component of the miRNA pathway. (a) The predicted *Ilex paraguariensis* AGO1 protein is 1,062 aa in length and presents the typical AGO1 glycine rich domain, the PAZ domain which is predicted to interact with single stranded small RNAs and the PIWI domain, responsible of the RNA-guided hydrolysis of single stranded-RNA. (b) Multiple protein alignment and secondary structure prediction of yerba mate, *Nicotiana benthamiana*, carrot and tomato AGO1 showing an important conservation in gen structure and domains. (c) A phylogenetic tree based in Jukes-Cantor, neighbor-joining and 1000 bootstraps indicates that AGO1 from yerba mate is more related with carrot than *Solanaceae* AGO1 despite the basic genetic distance among them (d).

A particular limited set of seventy six yerba mate transcripts identified yielded considerable similarity with several members of the RNA silencing and degradation pathway (**Table S3**). RNA interference is a post-transcriptional sequence-specific process of gene silencing that mediates resistance to both endogenous parasitic and exogenous pathogenic nucleic acids, and regulates the expression of protein-coding genes in eukaryotic organisms [42]. Among several enzymatic components of RNA interference such as Dicer-Like proteins, RNA dependent RNA polymerases, exosome members and dsRNA binding domain proteins, the family of Argonaute effectors (AGO) was also pinpointed in the yerba mate transcriptome. AGO and AGO-like proteins are the main RNA silencing effectors across kingdoms, and they mediate the cleavage of target RNAs using small RNAs of 20–24 nt as guides [43]. Argonaute 1 (AGO1) is responsible of two important circuits in plants: gene silencing of endogenous transcripts by microRNAs and virus resistance based in viral derived small RNAS [44], [45]. The yerba mate AGO1 predicted protein is estimated to be 1,062 aa in length and presented the typical AGO1 glycine rich domain (E-value  = 1,8e-25), the PAZ domain (E-value  = 4,3e-38) which is predicted to interact with single stranded small RNAs and the PIWI domain (E-value  = 3,5e-112), responsible of the RNA-guided hydrolysis of single stranded-RNA (**Figure 5a**). Multiple protein alignment and secondary structure prediction of yerba mate, *N. benthamiana*, carrot and tomato AGO1 showed an important conservation in gene structure and domains. A phylogenetic tree based in Jukes-Cantor, neighbor-joining and 1000 bootstraps indicated that AGO1 from yerba mate is more related with carrot than *Solanaceae* AGO1 despite the basic genetic distance among them (**Figure 5 b–d**, **Figure S14**, **Figure S15**).

**Figure 6:**
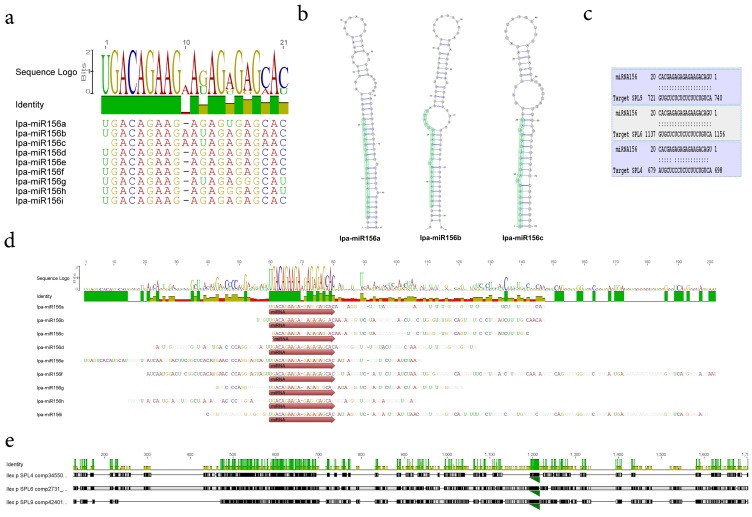
*MiR156* gene family in yerba mate. (a) Several mature miRNAs were predicted in yerba mate based in sequence homology to Mirbase. In the particular case of miR156, nine isoform variants were predicted with high sequence homology and minor mismatches. An insertion of a “A” at position 10 in miR156b and c forms, slightly affected the precursors secondary structure at the miRNA/miRNA* coordinates that can be observed as a bulge in (b). While the homology at the mature miR156 is high, the diversity among precursors of the miRNA gene family is extensive (d). A library generated of predicted SPL mRNAs of yerba mate was evaluated as a target of Ipa-miR156. A strong interaction with a high expectation score was *in silico* predicted for SPL9, SPL6 and SPL4 with Ipa-miR156 (c). These SPL genes significantly differ in their nucleotide sequence, however a strong conservation of the miR156 target can be observed in the 3 genes (green triangle, e).

MicroRNAs (miRNAs) are small non-coding RNAs that modulate plant gene expression by means of gene silencing through sequence-specific inhibition of target mRNAs. MiRNAs derive from precise processing of precursor transcripts with stem-loop secondary-structure features that are recognized by a Dicer-like complex. Mature miRNAs are loaded predominantly onto AGO1 and target endogenous RNAs for their degradation or translational arrest [46]. By combining two *in silico* based approaches we engaged in an attempted characterization of putative miRNA precursors in yerba mate. A yerba mate miRNA sequence prediction report based in UEA small RNA workbench platform and canonical relaxed mapping of conserved precursor miRNAs to the yerba mate transcriptome, indicated the presence of at least 59 pre-miRNAs corresponding to 41 of both young and ancient miRNA families (**Table S10**). The miR156 gene family has been involved in the regulation of developmental timing, vegetative phase change, flowering and sex identity in plants [47]–[49]. In yerba mate several mature miRNAs were predicted based in sequence homology to miRBase [50] (**Figure 6a**). In the particular case of miR156, nine isoform variants were predicted with high sequence homology and minor mismatches. An insertion of a “A” at position 10 in miR156b and c forms, slightly affected the precursors secondary structure at the miRNA/miRNA* coordinates that can be observed as a bulge in **Figure 6b**. While the homology at the mature miR156 was high, the diversity among precursors of the miRNA gene family was extensive **(** **Figure 6d** **)**. A library generated of predicted Squamosa Promoter Binding Protein-Like (SPL) mRNAs of yerba mate was evaluated as a target of Ipa-miR156. A strong interaction with a high expectation score was *in silico* predicted for SPL9, SPL6 and SPL4 with Ipa-miR156 (**Figure 6c**), which are typically conserved and validated targets of miR156 in plants [51]. These SPL genes significantly differed in their nucleotide sequence, however a strong conservation of the specific miR156 target could be observed in the 3 genes (**Figure 6e**). The identification of transcripts related to sex identity, such as miR156 and SPL gene families, is of special interest in yerba mate. In this diclino-dioecious crop, plants cannot be recognized as male or female prior to their first blooming, which occurs between 3 and 10 years post seedling emergence [14], delaying considerably the selection of parentals for breeding purposes. So, a cost-effective early sex determination system would be promising for the yerba mate breeding programs. It is tempting to postulate, that perhaps the determination of expression levels of these particular genes during yerba mate plant development, may be employed as a gender predictor at early stages.

### The yerba mate caffeine synthase

**Figure 7:**
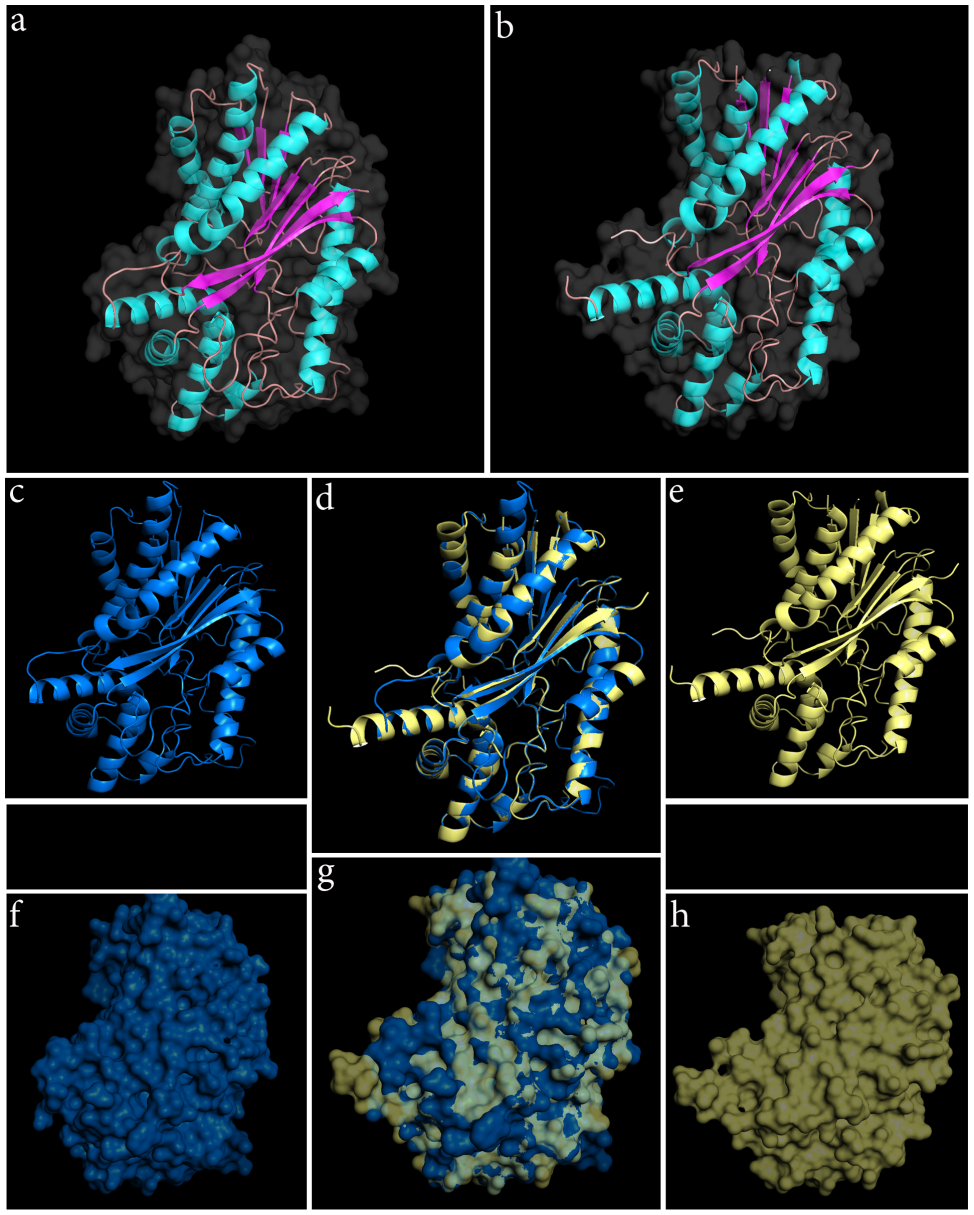
3D structure of *Ilex paraguariensis* caffeine synthase. (CS). Employing the X-ray crystallography solved structure of *Coffea arabica* CS as a template (b), the 3D structure of yerba mate CS was predicted by the swiss-model algorithm (a). A ribbon model of yerba CS (c) and coffee CS (e) suggest high conservation of secondary structure when superimposed (d). A reconstruction of a mesh model of yerba CS is presented (f) and compared to the coffee EM (h), showing extensive quaternary structure similarity (g).

One of the most important constituents of yerba mate extracts is caffeine [52], [53]. Caffeine is responsible for the stimulant effect of mate [54], and perhaps the underlying rationale of its profound influx in Latin American culture based on its effect on the body and mind and its properties that aid in staying awake and improving mental alertness after fatigue among others [55]. Caffeine or 1,3,7-trimethylxanthine is a crystalline xanthine alkaloid. Caffeine biosynthesis involves a series of reactions that direct the conversion of xanthosine to 7-methylxanthosine, to 7-methylxanthine to theobromine which is converted into caffeine [56]. The enzyme, assigned the name caffeine synthase (EC 2.1.1.160), catalyses the last two steps of caffeine biosynthesis, the conversion of 7-methylxanthine to caffeine via theobromine [57]. The gene encoding caffeine synthase (CS) was originally cloned from young tea leaves by Kato et al. [58]. Using the sequence of *Camellia sinensis* CS we probed our library and identified a sequence corresponding to the full length of an assembled transcript of the yerba mate transcriptome. The putative yerba mate assembled CS was identified by similarity to the *C. sinensis* CS complete mRNA (E-value  = 8e-168). The yerba mate 1,491 nt CS transcript was assembled based in 16,851 100 bp reads with an average coverage of 1,113X. Exploring this transcript, a single ORF was predicted encompassing 1,098 nt between coordinates 113 to 1,210, encoding a 366 aa protein with a 61% identity (E-value  = 2e-157) to the corresponding 369 aa *C. sinensis* CS protein, sharing the presence of the Methyltransferase_7 SAM dependent carboxyl methyltransferase domain involved in caffeine synthesis. With the predicted CS ORF, we performed multiple MUSCLE protein alignment and secondary structure prediction. By comparing *Coffea arabica*, *Theobroma cacao* and *C. sinensis* CS to *I. paraguariensis* predicted CS transcript an important conservation in gene structure and domains was observed (**Figure S16**). Since caffeine content is a desirable and important character in breeding programs of this crop, the preliminary and putative nature of the yerba mate predicted CS assembled transcript encourage further experimental validation, heterologous expression experiments and biochemical characterization of the full length CS coding sequence by traditional methods. After sequence annotation, we exploited the SWISS-MODEL algorithm to generate a yerba mate CS 3D prediction using *C. arabica* CS as a template (**Figure S17**). The 3D structure of the predicted *I. paraguariensis* caffeine synthase is presented in **Figure 7a** based in the X-ray crystallography solved structure of *C. arabica* CS (**Figure 7b**). A ribbon model of yerba mate CS (**Figure 7c**) and coffee CS (**Figure 7e**) suggested high conservation of secondary structure when superimposed (**Figure 7d**). A reconstruction of a mesh model of yerba mate CS is presented (**Figure 7f**) and compared with the coffee EM (**Figure 7h**), showing extensive quaternary structure similarity (**Figure 7g**).

### Chlorogenic acid in yerba mate

Chlorogenic acid (CGA, caffeoyl quinate, KEGG compound C00852) is the major phenolic compound found in yerba mate [59]. CGA acts as an antioxidant in plants, is involved in resistance to insects and defense against fungal pathogens. Human CGA consumption and its antioxidant role have been associated to protection against degenerative age-related diseases [59]. The enzyme 4-coumaroyl-CoA: quinnate O-(hydroxycinnamoyl)transferase (HQC, E.C.: 2.3.1.133) and p-coumaroyl quinate 3′-hydroxylase (C3′H, E.C.: 1.14.13.36), corresponding to the flavonoid and stilbenoid metabolic pathways, respectively (**Data S2**, pages 50 and 145), are responsible and limiting for CGA biosynthesis in tobacco, coffee and switchgrass [60]–[62]. We examined our transcriptome and, interestingly we found four putative HQC and two C3′H gene versions sharing over 75% identity with the corresponding coffee enzymes, presenting the typical condensation and Cytochrome P450 domains, respectively. We noticed that these transcripts were amongst the 1–4% most abundantly expressed, ranging from 40 to 160 FPKM. Although further studies are required, we suggest that the presence of redundant and highly expressed Chlorogenic acid related genes might be directly associated with the important production of CGA in yerba mate and its significant effects on oxidative stress reduction when consumed [63].

### *Ilex paraguariensis* PCR detection assay based in 5.8S and ITS2 assembled rRNA regions

In Argentina, *I. paraguariensis* is the only species authorized to be used to manufacture yerba mate products (Argentina law, Act 18.284, articles 1192–1193). It is interesting to note that a molecular based, standardized and cost effective method for *I. paraguariensis* detection is lacking in the literature. Moreover, *I. dumosa* is frequently used in mixtures with *I. paraguariensis*, and amounts above 1% are considered adulterants [64]. Based on our sequencing data we were able to assemble the *I. paraguariensis* rDNA corresponding regions of ITS1, 5.8S and ITS2. Multiple alignment of *I. paraguariensis* assembled 45S consensus sequence transcripts and *I. dumosa* homologous regions was performed (**Data S4**). This analysis showed that while ITS1 is highly conserved in both species, 5.8S and ITS2 are distinctive enough to differentiate them by means of PCR with species-specific primers. PCR amplifications were performed and agarose gel electrophoresis revealed that the designed primers from *I. paraguariensis* are species-specific and able to detect unequivocally genomic *I. paraguariensis* DNA. Moreover, by using the *I. dumosa* species-specific primers we were able to detect *I. dumosa* in DNA preparations obtained of mix leaf tissue of fractions above 1% of *I. dumosa* respective to *I. paraguariensis* as previously reported [64] (**Data S4**). PCR amplifications with both species-specific primers is a versatile, simple and cost effective method to detect *I. paraguariensis* and the typical yerba mate products adulterant.

### Conclusions

This is the first publicly available *I. paraguariensis* NGS study performed to investigate the entire yerba mate transcriptome, and our data provides the unique comprehensive transcriptome resource currently existing for yerba mate. In sum, through a systematic and exhaustive process of gene analysis and annotation, we have identified ∼1,000 putative transcription factors, genes involved in heat and oxidative stress, pathogen response, as well as disease resistance and hormone response. We have also identified transcripts related to osmotic, drought, salinity and cold stress, senescence and early flowering. We have also pinpointed several members of the gene silencing pathway and characterized the silencing effector Argonaute1. We predicted a diverse supply of putative microRNA precursors involved in developmental processes. We developed a draft of the transcribed genomes of the yerba mate chloroplast and mitochondrion. The putative sequence and predicted structure of the caffeine synthase of yerba mate is presented. Finally, we provide here a collection of over 10,800 SSR accessible to the community interested in yerba mate genetic improvement.

The transcriptome characterization of *I. paraguariensis* generated from our study is a very useful tool derived from a convenient and exhaustive approach of annotation and discovery of genes of several major metabolic pathways in this important crop. The vast amount of information obtained would encourage and serve as reliable source in the path to the discovery of biological and agronomic interesting traits, as well as for molecular markers development, gene mapping, analysis of genetic diversity and selection breeding in yerba mate.

## Materials and Methods

### Plant materials and RNA extraction

Leaf samples at emerging, young, fully expanded, and early and late senescent stages from *I. paraguariensis* breeding line Pg538 from INTA EEA-Cerro Azul, Misiones, Argentina, were collected and immediately frozen in liquid Nitrogen. Total RNA was isolated from pooled leaf tissue with the RNeasy Plant Mini Kit (Qiagen Inc.) and supplemented with RNase-free DNase (Qiagen Inc.). To increase the depth of depletion of ribosomal RNA, a process with the RiboMinus Plant Kit (Life Sciences Inc.) was performed with the isolated RNA. The resulting RNA was evaluated in concentration and purity using Nanodrop 1000 (Thermo Inc.), and subsequently subjected to an integrity analysis by agar electrophoresis and by Bioanalizer 2100 (Agilent Inc.) to determine quality parameters by QC and RING.

### RNA-seq library construction for Illumina sequencing

The resulting high quality RNA was employed for the generation of a cDNA library through TruSeq RNA Sample Preparation Kit (Illumina Inc.). The purified cDNA library was used for cluster generation on the Illumina Cluster Station and then sequenced on Illumina HiSeq 2000 following vendor instruction. A paired-end sequencing run with 100 nt read length for each read was performed for RNA-Seq. Raw sequencing intensities were then extracted and the bases were called using Illumina RTA software, followed by sequence quality filtering. The extracted sequencing reads were saved as a pair of fastq files for the first and second read, respectively.

### Sequence data analysis and assembly

Quality reports and filtering of the sequencing run and assembly were generated using CLC Genomics Workbench v7.0.4 (http://www.clcbio.com/) and the RobiNA v1.2.4 software (http://mapman.gabipd.org/web/guest/robin). All raw reads generated from the sequencer were quality filtered and *de novo* assembled into contigs using the Trinity program [17] with optimal parameters of 25 kmer word and group pairs distance of 500. The abundance of assembled contigs/isoforms was estimated using RSEM (http://deweylab.biostat.wisc.edu/rsem/) following the Trinity protocol. The raw sequencing data with quality scores have been deposited in the NCBI Short Read Archive database under accession number SRP043293.

### Sequence annotation

The obtained contigs were bulk analyzed in homology searches by BLASTX (http://blast.ncbi.nlm.nih.gov/Blast.cgi) to protein databases nr, Swiss-Prot (http://www.uniprot.org/), KEGG (http://www.genome.jp/kegg/) and COG (http://www.ncbi.nlm.nih.gov/COG/). The yerba mate transcripts were also investigated and analyzed with the Plant Ontology database (http://www.plantontology.org/), the database of essential genes DEG (http://www.essentialgene.org), Plaza 2.5 (http://bioinformatics.psb.ugent.be/plaza/) and alternatively with TAIR (http://www.arabidopsis.org/) the batch blast tool of the Rosaceae Genome Database (http://www.rosaceae.org/tools/batch_blast) and the PlantGDB BLAST (http://www.plantgdb.org/cgi-bin/blast/PlantGDBblast) using E≤1e-5 as threshold, retrieving best hits and functional annotations. The Blast2GO program www.blast2go.com/ (E≤1e-5) was also used to obtain GO annotation of genes. The GO annotations retrieved were subjected to enrichment analysis with the WEGO software (wego.genomics.org.cn/), Revigo web server (revigo.irb.hr/) and the AgriGO platform (bioinfo.cau.edu.cn/agriGO/).

### Organelle genome assembly and annotation

To generate a draft of yerba mate transcribed chloroplast, Illumina reads were relaxedly mapped to a *Lactuca sativa* chloroplast sequence (Accession no. AP007232.1) employing the map to reference utility of the Geneious 7.0 software (Geneious assembler, medium sensitivity, iterations up to 5 times). A consensus sequence was generated with the mapped reads and aligned to the *Lactuca sativa* chloroplast complete sequence using a Geneious global aligment with free end gaps (93%, gap open penalty 12, gap extension penalty 3). The yerba mate chloroplast draft was annotated integrating *Lactuca sativa* chloroplast gene predictions and *in silico* based estimations obtained with the Dual Organellar GenoMe Annotator (DOGMA, http://dogma.ccbb.utexas.edu/). A similar process was employed to generate a yerba mate mitochondrial draft. In this latter case to compensate for significant sequence gaps a Mauve genome alignment of yerba mate and *Helianthus annuus* mitochondrial complete sequence (Accession no. KF815390.1) was generated. The annotation of the yerba mate transcribed mitochondrion draft was generated based in sequence homology to the respective sunflower predicted genes.

### MicroRNA prediction

We analyzed both the assembled transcripts and the raw reads by two *in silico* based approaches in an attempted characterization of putative miRNA precursors in yerba mate. The UEA small RNA workbench platform (srna-workbench.cmp.uea.ac.uk/) with plant standard parameters and a cut-off P-value of 0.05 was employed using the yerba mate assembled transcriptome as reference. In parallel, a canonical relaxed mapping (similarity: 0.8 to 0.75, length fraction: 0.8, mismatch cost: 2, insertion cost: 2, deletion cost: 3) of conserved precursor miRNAs from miRBASE Release 20 (ftp://mirbase.org/pub/mirbase/CURRENT/miRNA.dat.gz) to the yerba mate transcriptome on the CLC Genomics Workbench v7.0.4 environment was generated and mapped reads were analyzed by eye and evaluated by proper secondary folding. The secondary structures of the predicted stem-loops secondary structures were solved using the mfold web server (http://mfold.rna.albany.edu/) using version 2.3 and adjusting folding temperature to 24°C, and Vienna type RNA structures were predicted with Context Fold (http://www.cs.bgu.ac.il/~negevcb/contextfold/).

### Caffeine synthase structure prediction

*Coffea arabica* (Acc. no: BAC43759.1), *Theobroma cacao* (Acc. no: BAE79730.1), *Camellia sinensis* (Acc. no: ABP98983.1) and *Ilex paraguariensis* predicted caffeine synthase coding sequences were subjected to multiple MUSCLE protein alignments (8 iterations, distance methods kmer6-6 and pctd-kimura, and clustering by UPGMB) and secondary structure prediction with the GOR method (Garnier-Osguthorpe-Robson) with Genious v7.0.

Yerba mate CS cuaternary structure was predicted with the automated protein structure homology-modelling server (swissmodel.expasy.org/; [65]) using as a template the solved structure of *Coffea arabica* CS (2efjA (2.00 A)). The pdb obtained structure was analyzed and rendered using the PyMol software version 1.7 (www.pymol.org/).

### SSR detection

The GDR SSR Server [66], [67] was employed to identify single sequence repeats in the transcriptome of yerba mate. 5 types of SSRs (di-nucleotide, tri-nucleotide, tetra-nucleotide, penta-nucleotide, hexa-nucleotide) were screened with an specific minimum number of 6, 4, 3, 3, 3 repeats respectively.

### Transposable elements analysis

Transposable elements were first identified based in homology searches by NCBI-BLASTN and BLASTX. Specifically, Non-LTR Long Interspersed Elements (LINE) were pinpointed by the retrotransposase domain; LTR elements (Copia-like and Gypsy) were scanned with the LTR-FINDER software [68] and DNA transposons by domain based search. Every candidate was then explored and re-evaluated with NCBI conserved domain search (http://www.ncbi.nlm.nih.gov/Structure/cdd/wrpsb.cgi?).

## Supporting Information

Figure S1 **Illumina RNA sequencing analysis.** (a) A lack of peaks reaching up to 100% at individual cycles in the homopolymer graph indicates an absence of a common technical artifact of cycle-wise multiplied calls of the same nt. (b) The Sequence quality plots allow an overview of the base call qualities assigned to each base by the base caller module of the sequencing pipeline. The plot shows the median (solid blue line), the 25th percentile and the 75th percentile (lower and upper bound of the light blue area) of the qualities at each position (cycle). (c) The base call frequency plot indicates a lack of positional biases in the call frequency for each base. (d) The plot shows the distribution of qualities averaged across the reads. In the yerba mate data, the average quality score is 36.3 (indicated by the red line). (e) The Kmer frequency check identifies short sequences that occur more often than expected. 10 Kmers that occur 3 times more often than expected are indicated.
(TIF)

Figure S2 **Trinity** ***de novo*** **assembly report.** (a) Distribution of GC-contents: The GC-content of a sequence is calculated as the number of GC-bases normalized to the total number of sequences. (b) Combined coverage of G and C bases: number of G and C bases observed at current position normalized to the total number of bases observed at that position. (c) The five most-overrepresented 5mers. The over-representation of a 5mer is calculated as the ratio of the observed and expected 5mer frequency. The expected frequency is calculated as product of the empirical nucleotide probabilities that make up the 5mer. (d) The number of sequences that support (cover) the individual base positions normalized to the total number of sequences. (e) Distribution of sequence lengths: x: sequence length in base-pairs y: number of sequences featuring a particular length normalized to the total number of sequences. (f) Coverages for the four DNA nucleotides.
(TIF)

Figure S3 **Semantic similarity-based scatterplots of gene ontology categories representations of the yerba mate transcriptome based in biological process (a), cellular component (b) or molecular function (c).** Circle size is estimated based in p-values associated to the GO categories. Color legend is represented as an inset in (a). The Revigo web server was used to generate the plots: http://revigo.irb.hr/.
(TIF)

Figure S4 **Tree-map visualization of enriched GO categories of yerba mate transcriptome based in biological process (a), cellular component (b) or molecular function (c).** Rectangle size is estimated based in p-values associated to the GO categories. The Revigo web server was used to generate the maps: http://revigo.irb.hr/.
(TIF)

Figure S5 **AgriGO generated plot of GO enrichment in yerba mate based in biological process.** Significance color levels and arrow types associated with GO relationships are represented as an inset at the superior left corner.
(TIF)

Figure S6 **AgriGO generated plot of GO enrichment in yerba mate based in cellular component.** Significance color levels and arrow types associated with GO relationships are represented as an inset at the superior left corner.
(TIF)

Figure S7 **AgriGO generated plot of GO enrichment in yerba mate based in molecular function.** Significance color levels and arrow types associated with GO relationships are represented as an inset at the superior left corner.
(TIF)

Figure S8 **Profile of ribosomal proteins and ribosome biogenesis related transcripts obtained from the yerba mate transcriptome.** Plant Ribosomes are constituted by 4 rRNAs and ∼80 ribosomal proteins. In this study every major structural ribosome constituents **(a)**, and roughly every enzyme (**b**) responsible for ribosome processing, trafficking, rRNA maturation, and ribosome assembly were identified (green). Images credits: Kanehisa Laboratories, Japan.
(TIF)

Figure S9 **Profile of plant hormone signal transduction related transcripts obtained from the yerba mate transcriptome.** Plant development is regulated by endogenous signaling molecules including plant hormones. Perception of biological cues and signal transduction involves several hormone sensing and effector pathways. The major yerba mate enzymes involved in plant growth, cell division, stem growth, seed dormancy, senescence and cell elongation were identified in the assembled transcriptome (green). Images credits: Kanehisa Laboratories, Japan.
(TIF)

Figure S10 **Genome alignment of** ***Lactuca sativa*** **chloroplast complete sequence (Accession no. AP007232.1) and yerba mate chloroplast.** Identity is obtained based in 1 nt sliding window size and represented by color and bar height from 0% (red) to 100% (green). Annotations are depicted as protein coding genes (yellow), transfer RNA genes (pink) and ribosome RNA genes (red).
(TIF)

Figure S11 **Mapping of** ***Ilex paraguariensis*** **assembled transcripts to the chloroplast sequence draft.**
(TIF)

Figure S12 **Genome alignment of sunflower mitochondrial complete sequence and yerba mate mitochondrial sequence consensus.** Identity is obtained based in 1 nt sliding window size and represented by color and bar height from 0% (red) to 100% (green). Annotations are depicted as protein coding genes (yellow), transfer RNA genes (pink) and ribosome RNA genes (red).
(TIF)

Figure S13 **Mauve genome alignment of yerba mate and** ***Helianthus annuus*** **mitochondrial complete sequence (Accession no. KF815390.1).** Identity is represented hierarchically from white to red. The consensus *Ilex paraguariensis* sequence conserves most of the *Helianthus* gene annotations (rectangles). As an example, the consensus sequence of Ilex p. at 76,000 bp coordinates presents high identity to the 230,000 bp coordinates of sunflower (transparent bars), corresponding to the ccmFn coding sequence.
(TIF)

Figure S14 **Bayesian phylogenic tree of the** ***Argonaute 1*** **(AGO1) genes of 35 plant species and yerba mate determined by the Geneious 7.0 platform.** Values at the nodes indicate bootstrap support percentage obtained for 1,000 replicates.
(TIF)

Figure S15 **Multiple gene alignment of** ***Argonaute 1*** **(AGO1) genes of 35 plant species and yerba mate.** Identity is obtained based in 1 nt sliding window size and represented by color and bar height from 0% (red) to 100% (green).
(TIF)

Figure S16 **Multiple MUSCLE protein alignment and secondary structure prediction of** ***Coffea arabica*** **,** ***Theobroma cacao*** **,** ***Camellia sinensis*** **and** ***Ilex paraguariensis*** **caffeine synthase showing an important conservation in gene structure and domains.**
(TIF)

Figure S17 **SWISS-MODEL report of yerba mate caffeine synthase 3D prediction using** ***Coffea arabica*** **CS as a template.**
(PDF)

Table S1 **Transcription factors predicted in yerba mate based in BLASTX hits descriptions employing the** ***viridiplantae*** **UniProt, or** ***Arabidopsis*** **TAIR protein database as reference.**
(XLSX)

Table S2 **Gene Ontology terms associated to the yerba mate transcriptome.** The GO terms were obtained employing the KAAS (KEGG Automatic Annotation Server, http://www.genome.jp/kegg/kaas/) for ortholog assignment and pathway mapping.
(XLSX)

Table S3 **Categorized yerba mate complete transcriptome best BLASTX hits, against the** ***viridiplantae*** **UniProt database.**
(XLSX)

Table S4 **Yerba mate complete transcriptome best BLASTX hits, against the TAIR protein database.**
(XLSX)

Table S5 **List and description of Gene Ontology, KO terms and mapsassociated to the yerba mate transcriptome obtained by the KAAS and deduced from BLASTX hits against** ***Arabidopsis*** **TAIR protein database and** ***viridiplantae*** **UniProt.**
(XLSX)

Table S6 **Complete gene catalog list of yerba mate draft chloroplast and mitochondrion.**
(XLSX)

Table S7 ***Helianthus annuus*** **mitochondrial gene coordinates and the analogous yerba mate mitochondrial draft ortholog gene sequence coordinate list.**
(XLSX)

Table S8 **Predicted SSR summary, frequencies report and designed alternative primers for the yerba mate chloroplast genome.**
(XLS)

Table S9 **Predicted SSR summary, frequencies report and designed alternative primers for the yerba mate mitochondrial genome draft.**
(XLS)

Table S10 **Yerba mate miRNA sequence prediction report based in UEA small RNA workbench platform and canonical relaxed mapping of conserved precursor miRNAs to the yerba transcriptome on the CLC Genomics environment.**
(XLSX)

Table S11 **Predicted SSR summary and frequencies report for the yerba mate complete transcriptome.**
(XLSX)

Table S12 **Transposable element-like sequences present in the yerba mate transcriptome.**
(XLSX)

Data S1 **DEG analysis of the yerba mate transcriptome.**
(XLSX)

Data S2 **166 KEGG pathways models graphs representing most of yerba mate annotated genes (in green).** Images credits: Kanehisa Laboratories, Japan.
(PDF)

Data S3 **Alternative language abstract in Spanish.**
(DOC)

Data S4 **PCR detection assay of** ***I. paraguariensis*** **vs.** ***I. dumosa*** **based in 5.8S and ITS2 distinctive regions.** Designed primer features, sample preparation and PCR conditions are presented as tables. Multiple alignment of *I. paraguariensis* assembled 45S consensus sequence vs. *I. dumosa* Acc. No. AJ492657 reveals that ITS1 is highly conserved in both species; however 5.8S and ITS2 are distinctive enough to differentiate them by means of PCR with species-specific primers. Primer bind locations are annotated in blue in both species. Agarose gel electrophoresis showing differential amplification according to primers specificity and reliability of the method is presented. M1: Promega 200 bp ladder molecular DNA ruler; M2: in-house control Marker; NTC: Non Template Control; 100%, 50%, 10%, 1%, 0.1%: percentage of *I. dumosa* (I.du) fraction respective to *I. paraguariensis* (I.pa) of leaf tissue employed for DNA isolation and subjected to specific PCR.
(XLSX)
