# Supplementary figures and images for: Exploring the Genes of Yerba Mate (Ilex paraguariensis A. St.-Hil.) by NGS and De Novo Transcriptome Assembly
Source: PLoS One. 2014 Oct 16;9(10):e109835. doi: 10.1371/journal.pone.0109835 (PMC4199719; doi:10.1371/journal.pone.0109835)

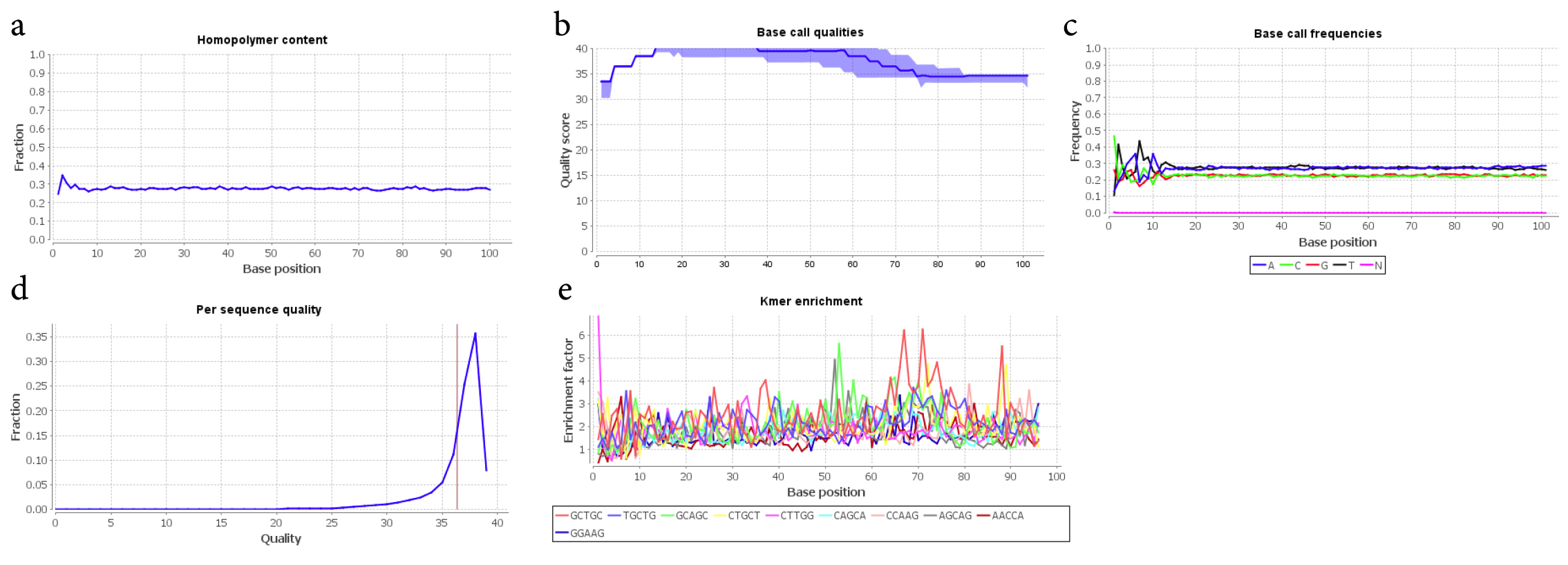

Supplement: Figure S1 — Illumina RNA sequencing analysis. (a) A lack of peaks reaching up to 100% at individual cycles in the homopolymer graph indicates an absence of a common technical artifact of cycle-wise multiplied calls of the same nt. (b) The Sequence quality plots allow an overview of the base call qualities assigned to each base by the base caller module of the sequencing pipeline. The plot shows the median (solid blue line), the 25th percentile and the 75th percentile (lower and upper bound of the light blue area) of the qualities at each position (cycle). (c) The base call frequency plot indicates a lack of positional biases in the call frequency for each base. (d) The plot shows the distribution of qualities averaged across the reads. In the yerba mate data, the average quality score is 36.3 (indicated by the red line). (e) The Kmer frequency check identifies short sequences that occur more often than expected. 10 Kmers that occur 3 times more often than expected are indicated. (TIF) [file pone.0109835.s001.tif]

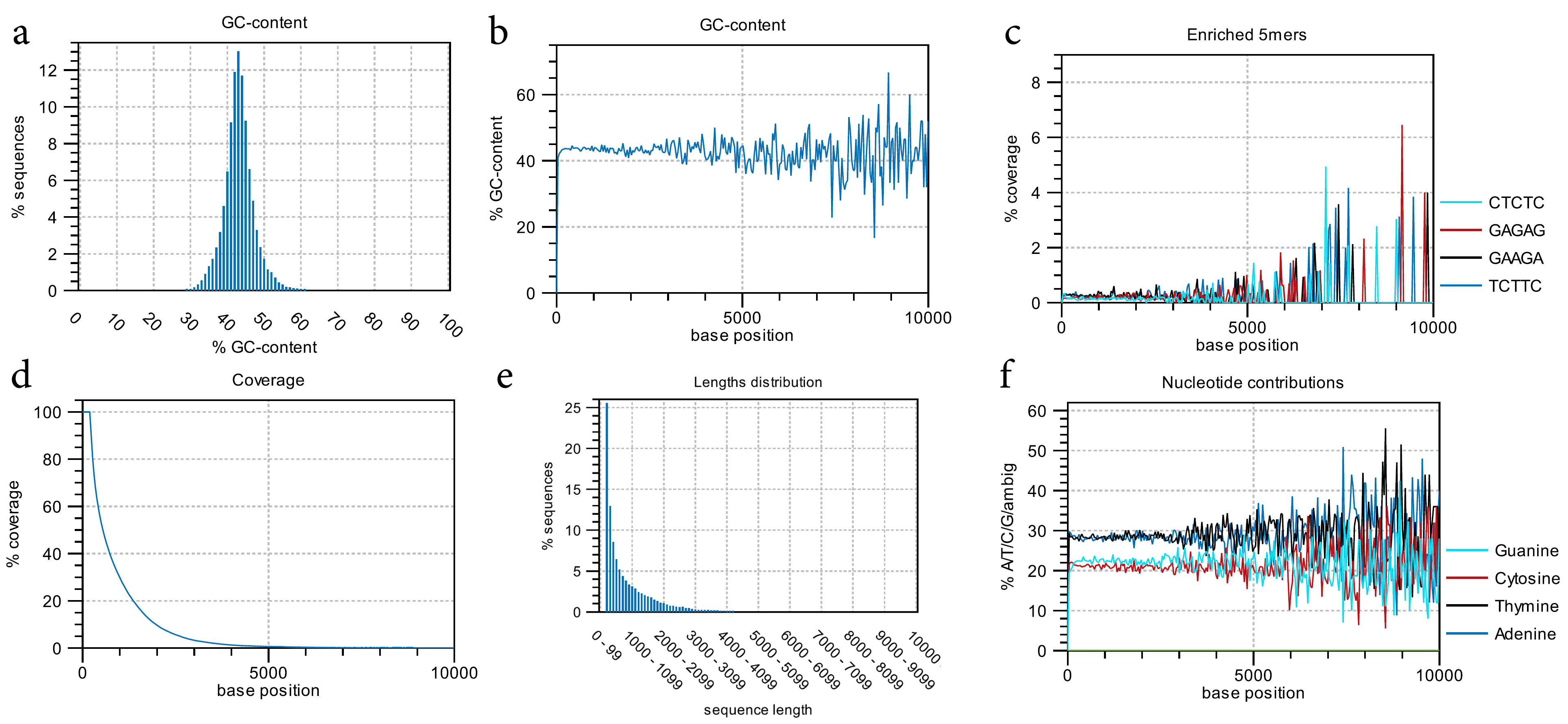

Supplement: Figure S2 — Trinity de novo assembly report. (a) Distribution of GC-contents: The GC-content of a sequence is calculated as the number of GC-bases normalized to the total number of sequences. (b) Combined coverage of G and C bases: number of G and C bases observed at current position normalized to the total number of bases observed at that position. (c) The five most-overrepresented 5mers. The over-representation of a 5mer is calculated as the ratio of the observed and expected 5mer frequency. The expected frequency is calculated as product of the empirical nucleotide probabilities that make up the 5mer. (d) The number of sequences that support (cover) the individual base positions normalized to the total number of sequences. (e) Distribution of sequence lengths: x: sequence length in base-pairs y: number of sequences featuring a particular length normalized to the total number of sequences. (f) Coverages for the four DNA nucleotides. (TIF) [file pone.0109835.s002.tif]

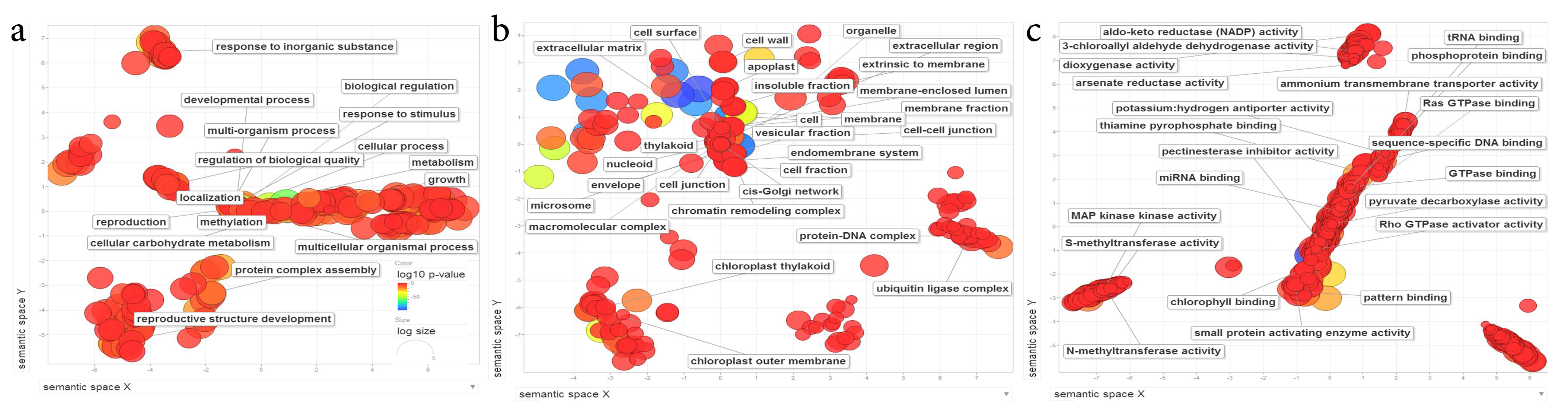

Supplement: Figure S3 — Semantic similarity-based scatterplots of gene ontology categories representations of the yerba mate transcriptome based in biological process (a), cellular component (b) or molecular function (c). Circle size is estimated based in p-values associated to the GO categories. Color legend is represented as an inset in (a). The Revigo web server was used to generate the plots: http://revigo.irb.hr/. (TIF) [file pone.0109835.s003.tif]

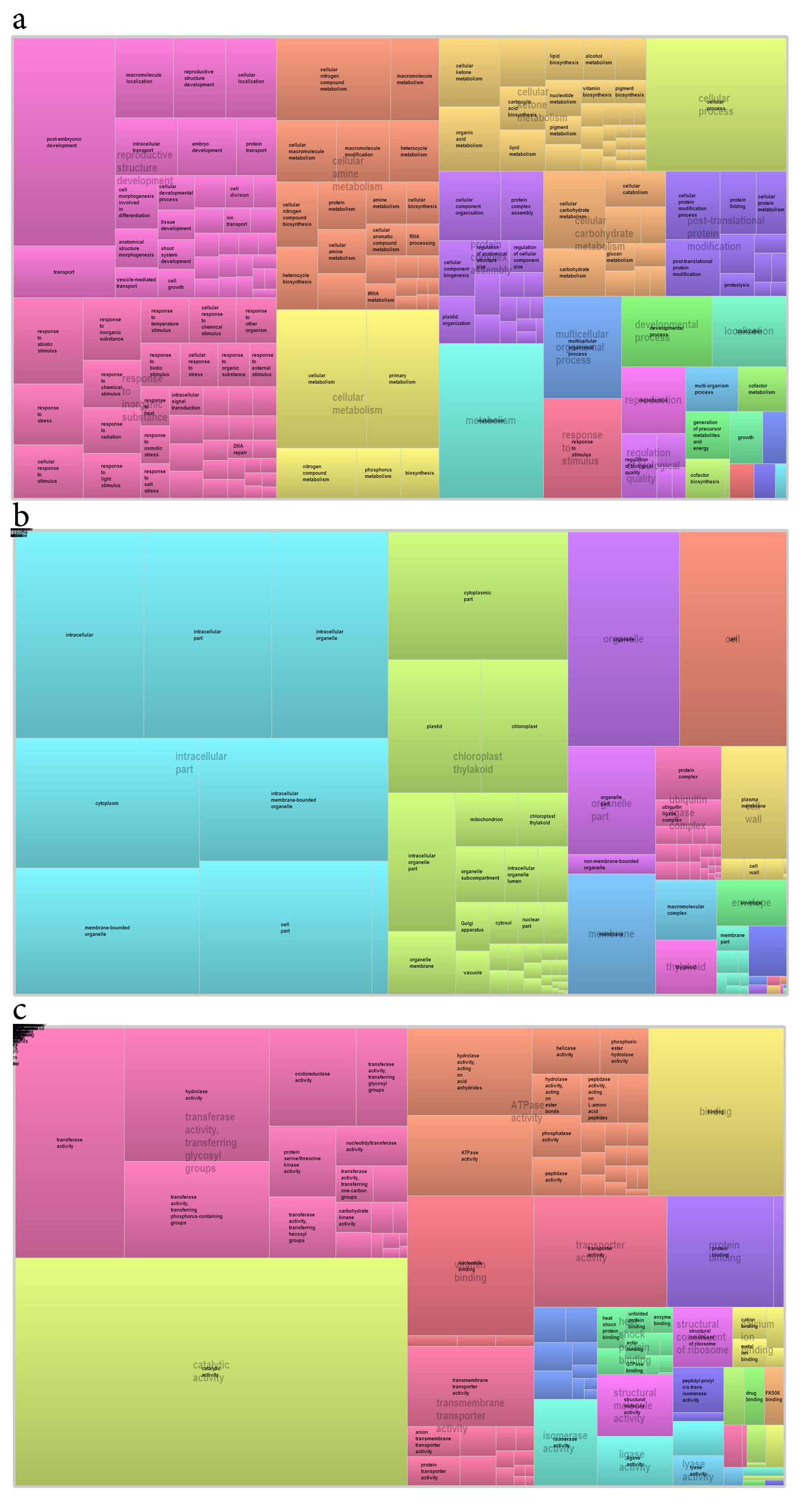

Supplement: Figure S4 — Tree-map visualization of enriched GO categories of yerba mate transcriptome based in biological process (a), cellular component (b) or molecular function (c). Rectangle size is estimated based in p-values associated to the GO categories. The Revigo web server was used to generate the maps: http://revigo.irb.hr/. (TIF) [file pone.0109835.s004.tif]

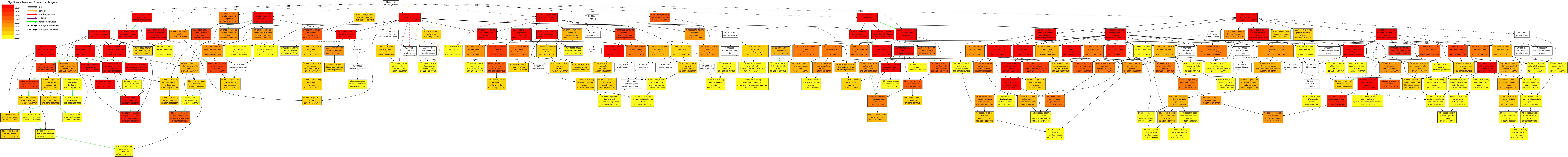

Supplement: Figure S5 — AgriGO generated plot of GO enrichment in yerba mate based in biological process. Significance color levels and arrow types associated with GO relationships are represented as an inset at the superior left corner. (TIF) [file pone.0109835.s005.tif]

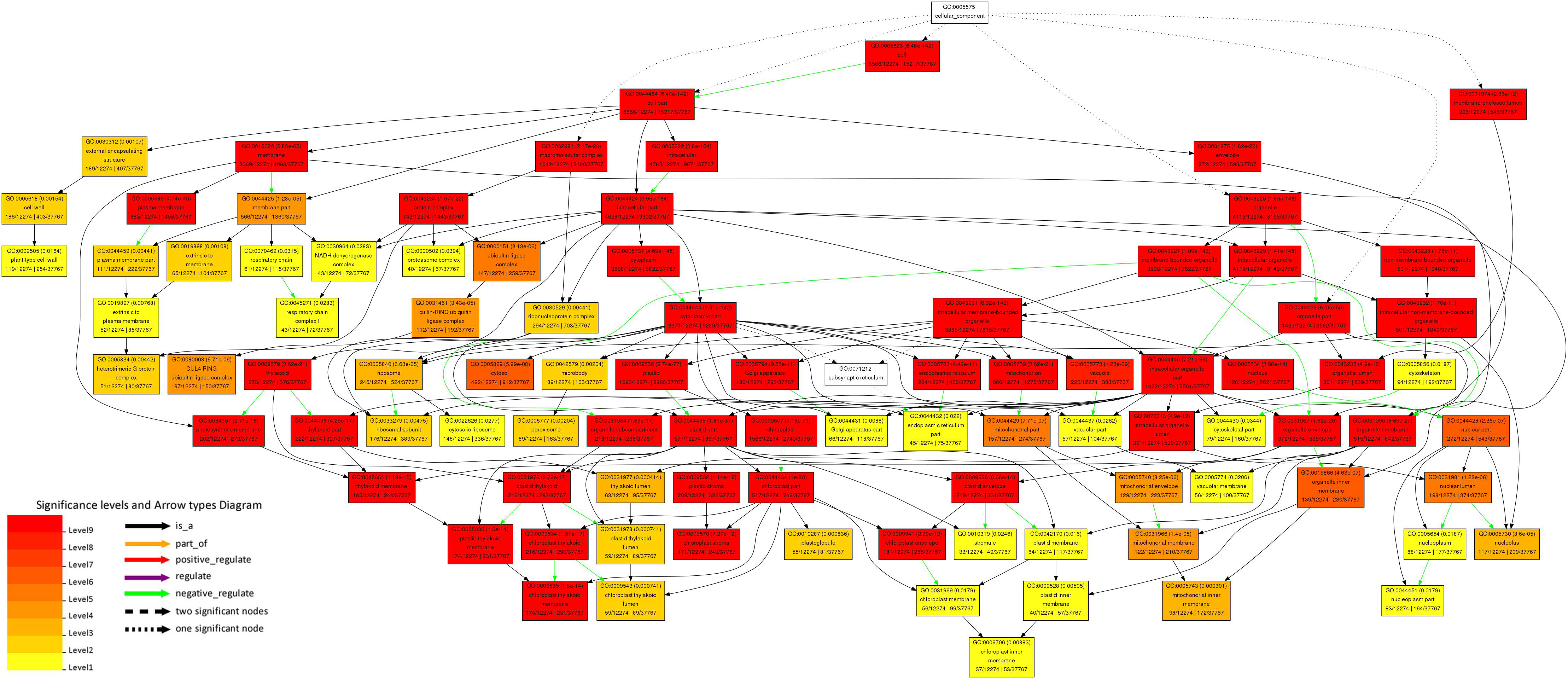

Supplement: Figure S6 — AgriGO generated plot of GO enrichment in yerba mate based in cellular component. Significance color levels and arrow types associated with GO relationships are represented as an inset at the superior left corner. (TIF) [file pone.0109835.s006.tif]

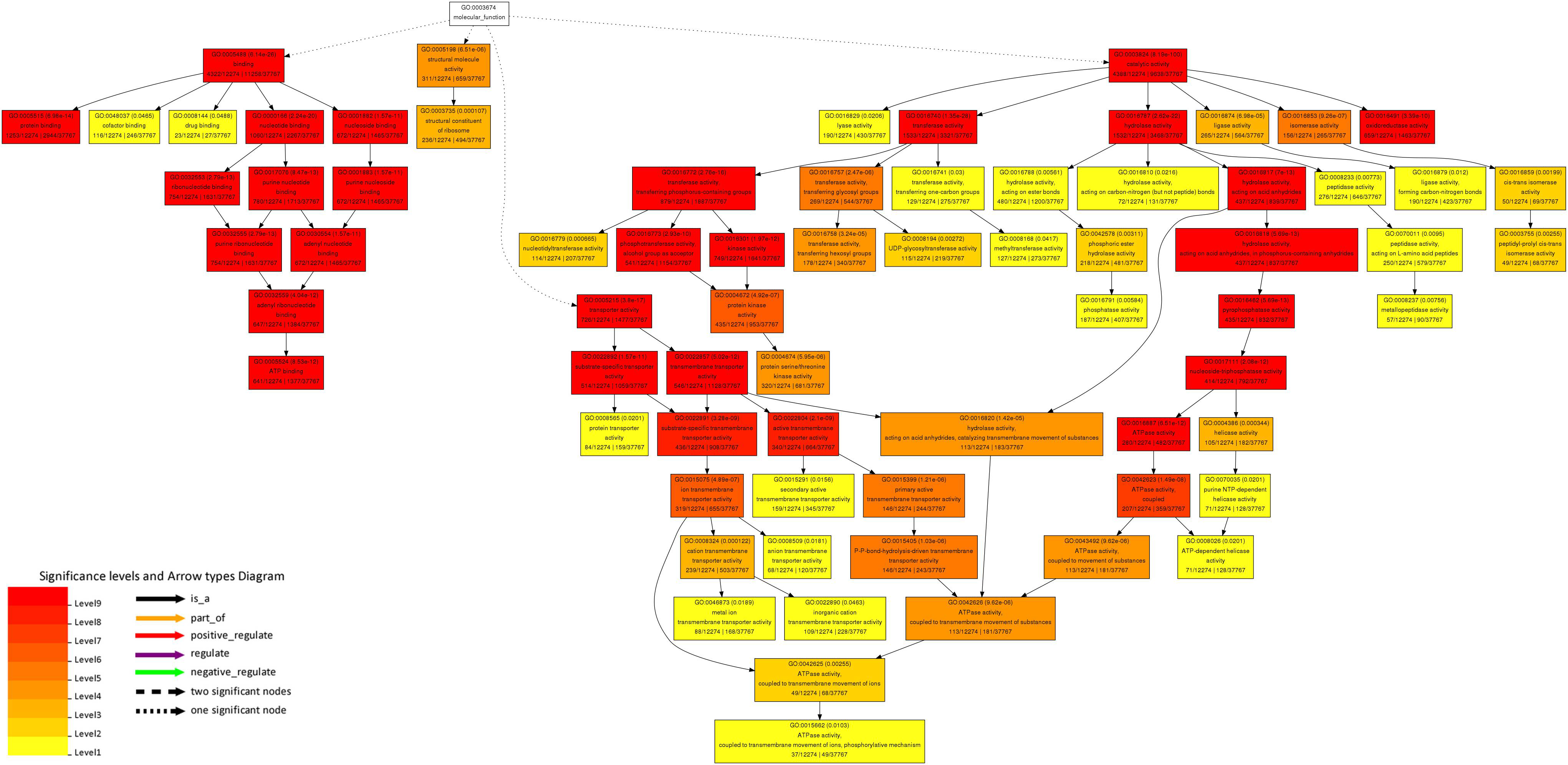

Supplement: Figure S7 — AgriGO generated plot of GO enrichment in yerba mate based in molecular function. Significance color levels and arrow types associated with GO relationships are represented as an inset at the superior left corner. (TIF) [file pone.0109835.s007.tif]

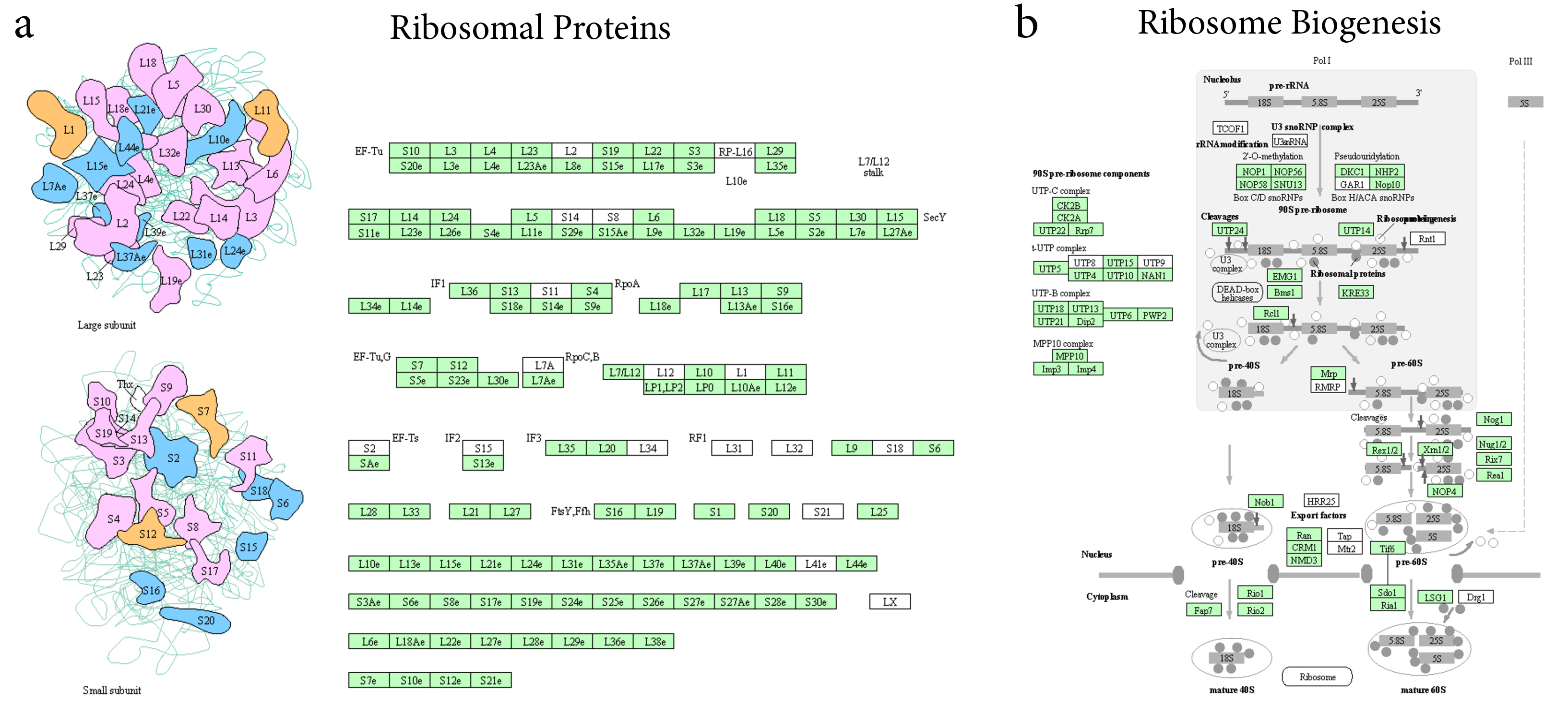

Supplement: Figure S8 — Profile of ribosomal proteins and ribosome biogenesis related transcripts obtained from the yerba mate transcriptome. Plant Ribosomes are constituted by 4 rRNAs and ∼80 ribosomal proteins. In this study every major structural ribosome constituents (a), and roughly every enzyme (b) responsible for ribosome processing, trafficking, rRNA maturation, and ribosome assembly were identified (green). Images credits: Kanehisa Laboratories, Japan. (TIF) [file pone.0109835.s008.tif]

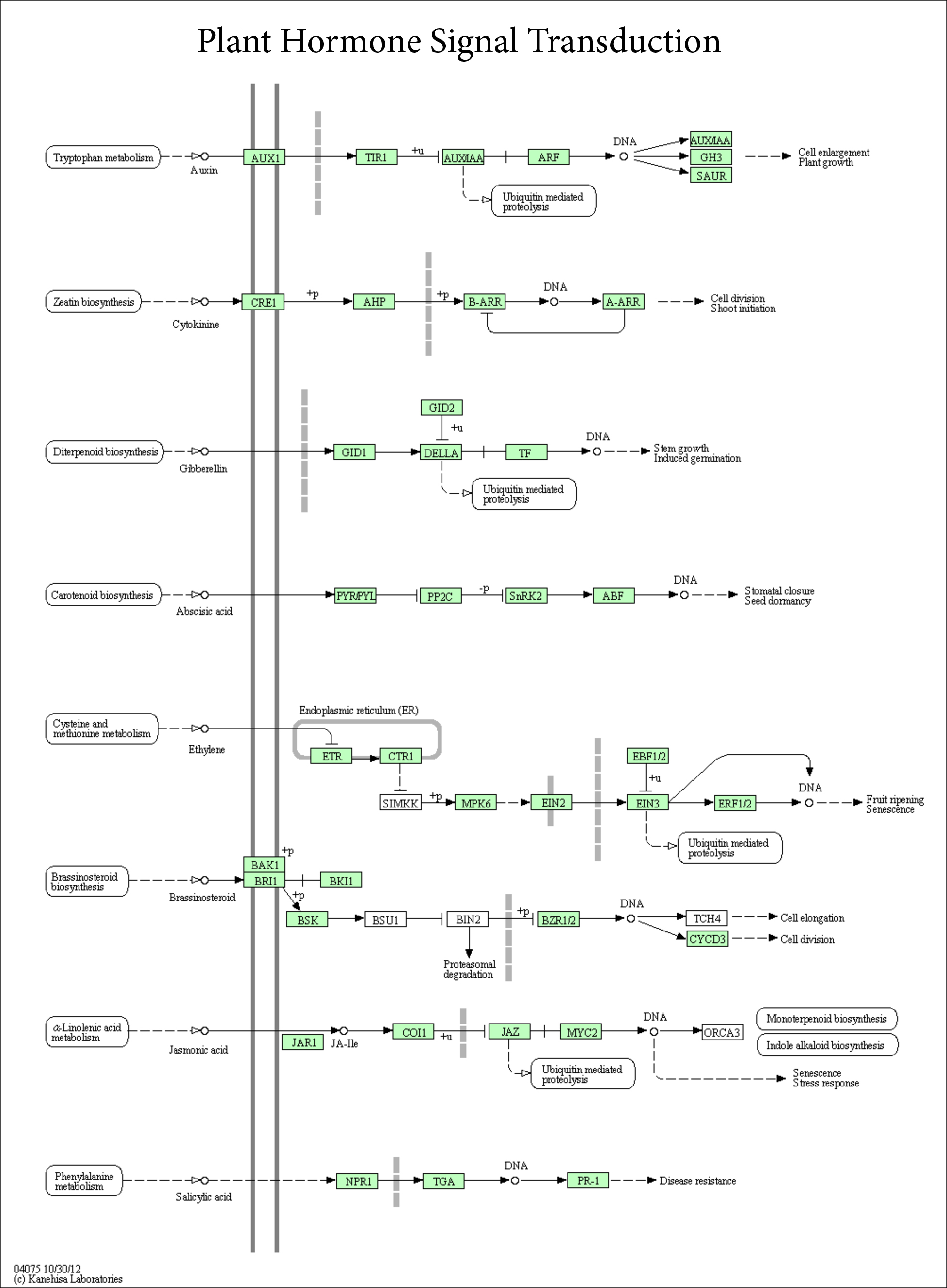

Supplement: Figure S9 — Profile of plant hormone signal transduction related transcripts obtained from the yerba mate transcriptome. Plant development is regulated by endogenous signaling molecules including plant hormones. Perception of biological cues and signal transduction involves several hormone sensing and effector pathways. The major yerba mate enzymes involved in plant growth, cell division, stem growth, seed dormancy, senescence and cell elongation were identified in the assembled transcriptome (green). Images credits: Kanehisa Laboratories, Japan. (TIF) [file pone.0109835.s009.tif]

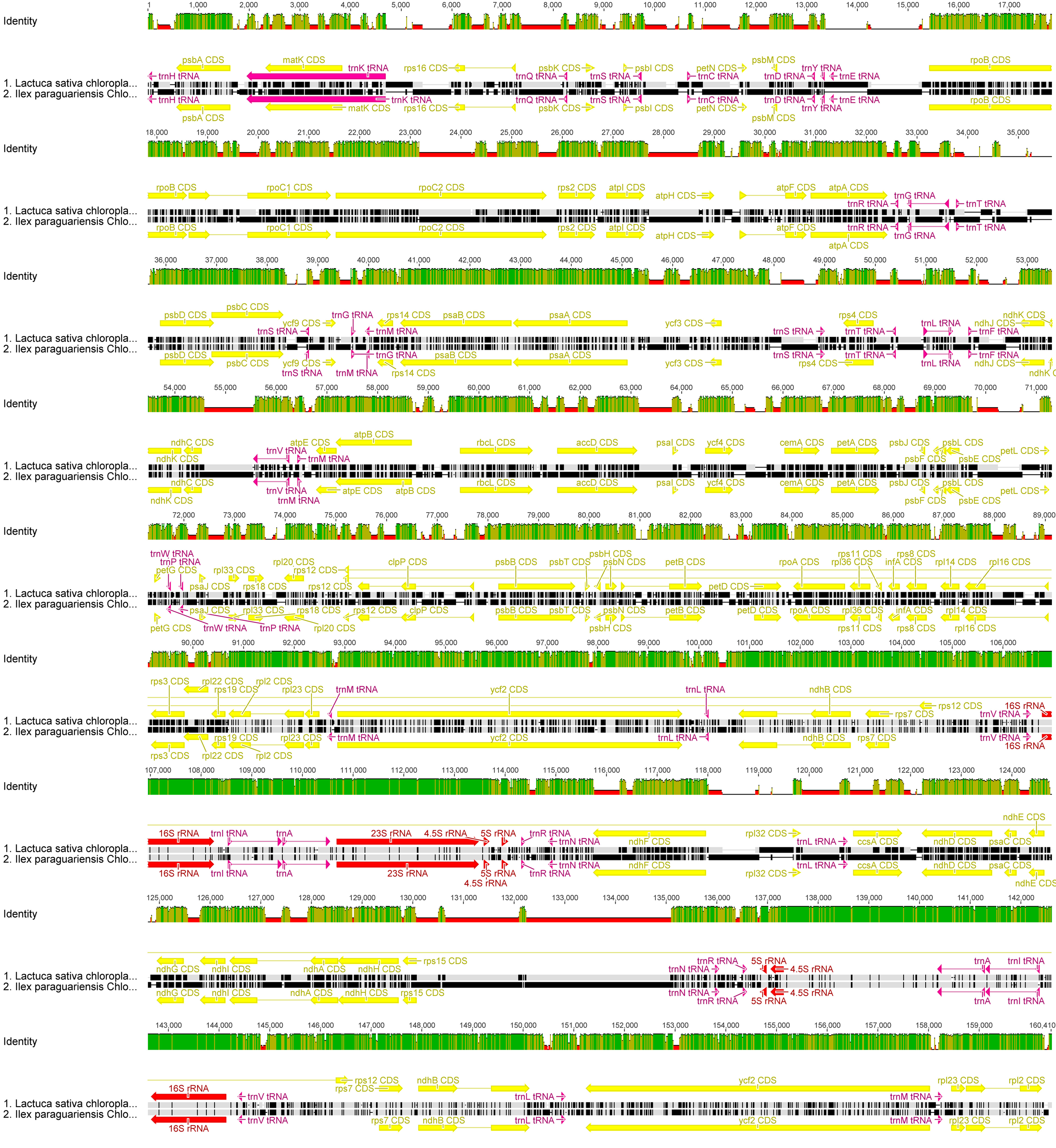

Supplement: Figure S10 — Genome alignment of Lactuca sativa chloroplast complete sequence (Accession no. AP007232.1) and yerba mate chloroplast. Identity is obtained based in 1 nt sliding window size and represented by color and bar height from 0% (red) to 100% (green). Annotations are depicted as protein coding genes (yellow), transfer RNA genes (pink) and ribosome RNA genes (red). (TIF) [file pone.0109835.s010.tif]

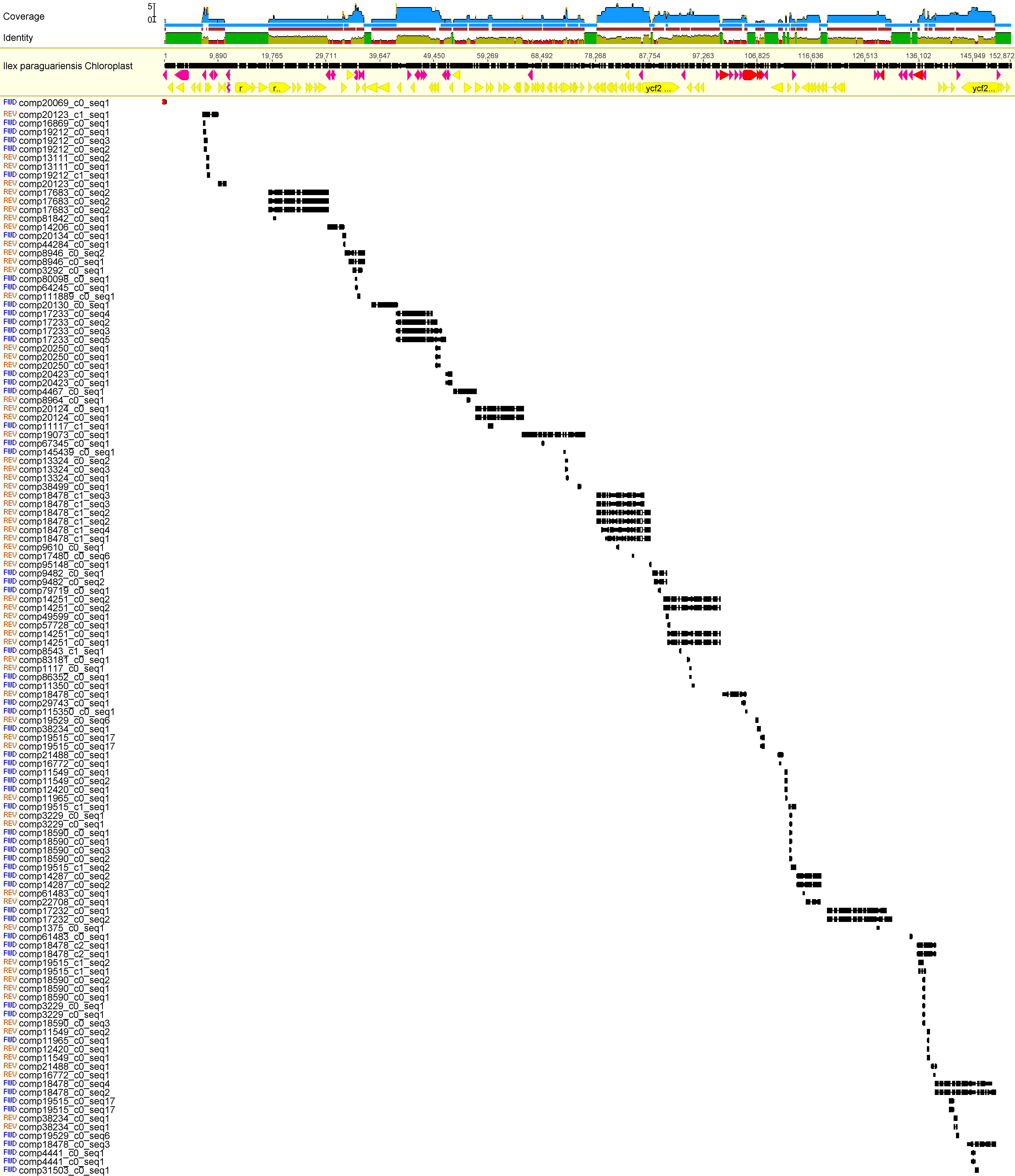

Supplement: Figure S11 — Mapping of Ilex paraguariensis assembled transcripts to the chloroplast sequence draft. (TIF) [file pone.0109835.s011.tif]

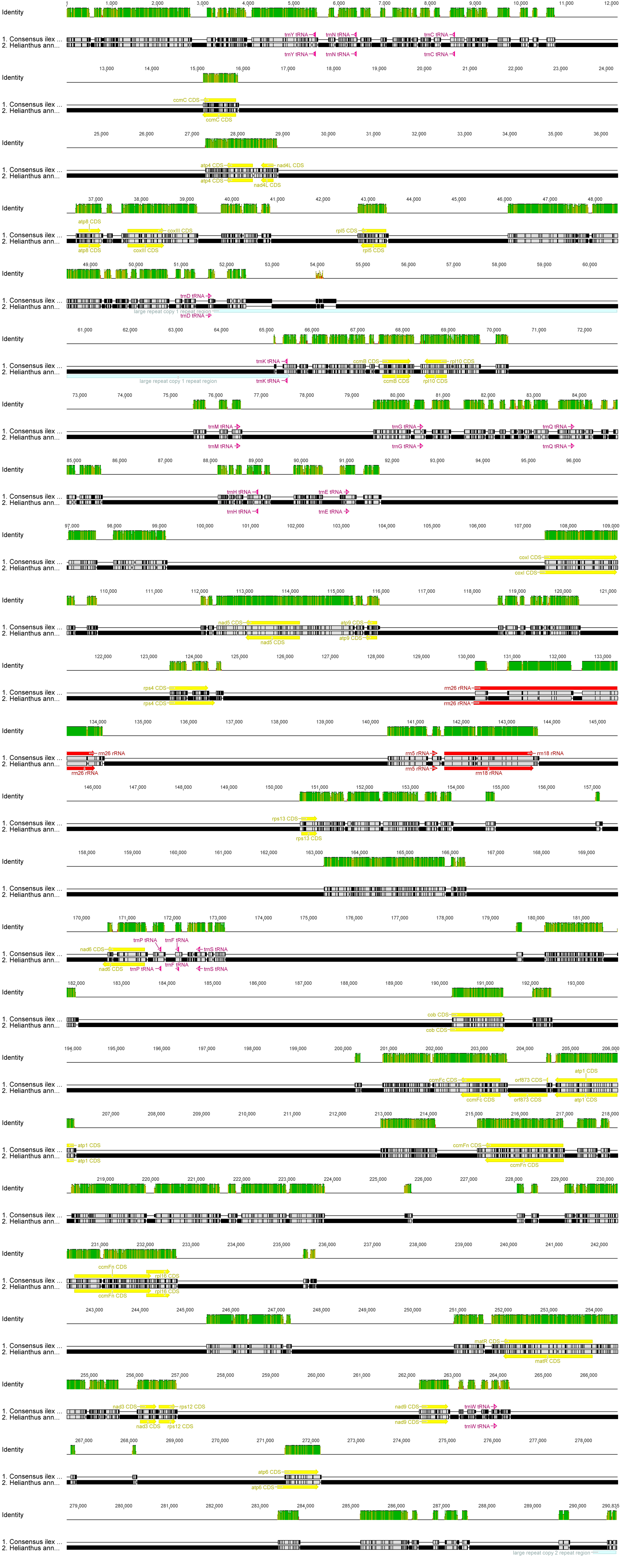

Supplement: Figure S12 — Genome alignment of sunflower mitochondrial complete sequence and yerba mate mitochondrial sequence consensus. Identity is obtained based in 1 nt sliding window size and represented by color and bar height from 0% (red) to 100% (green). Annotations are depicted as protein coding genes (yellow), transfer RNA genes (pink) and ribosome RNA genes (red). (TIF) [file pone.0109835.s012.tif]

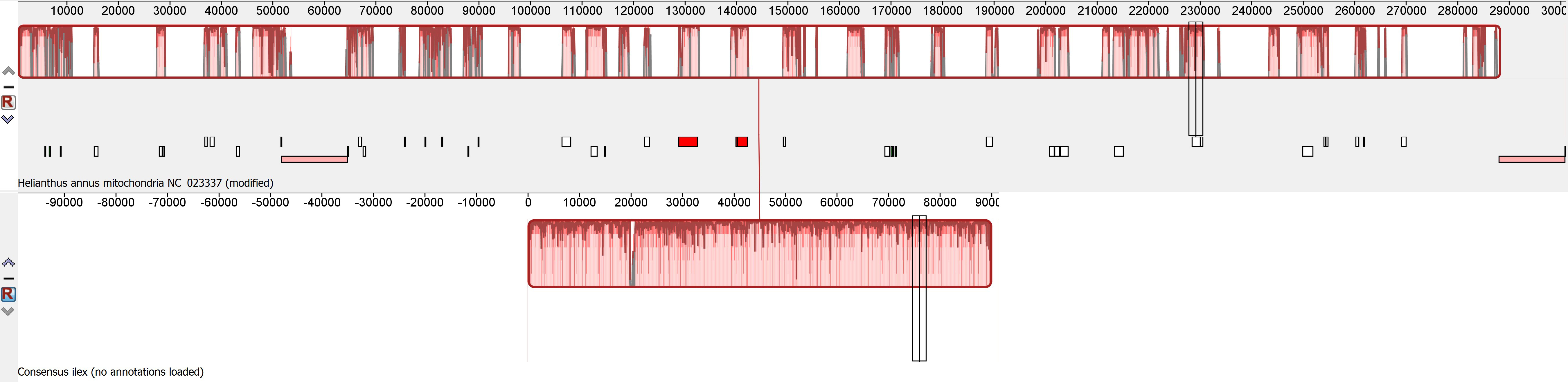

Supplement: Figure S13 — Mauve genome alignment of yerba mate and Helianthus annuus mitochondrial complete sequence (Accession no. KF815390.1). Identity is represented hierarchically from white to red. The consensus Ilex paraguariensis sequence conserves most of the Helianthus gene annotations (rectangles). As an example, the consensus sequence of Ilex p. at 76,000 bp coordinates presents high identity to the 230,000 bp coordinates of sunflower (transparent bars), corresponding to the ccmFn coding sequence. (TIF) [file pone.0109835.s013.tif]

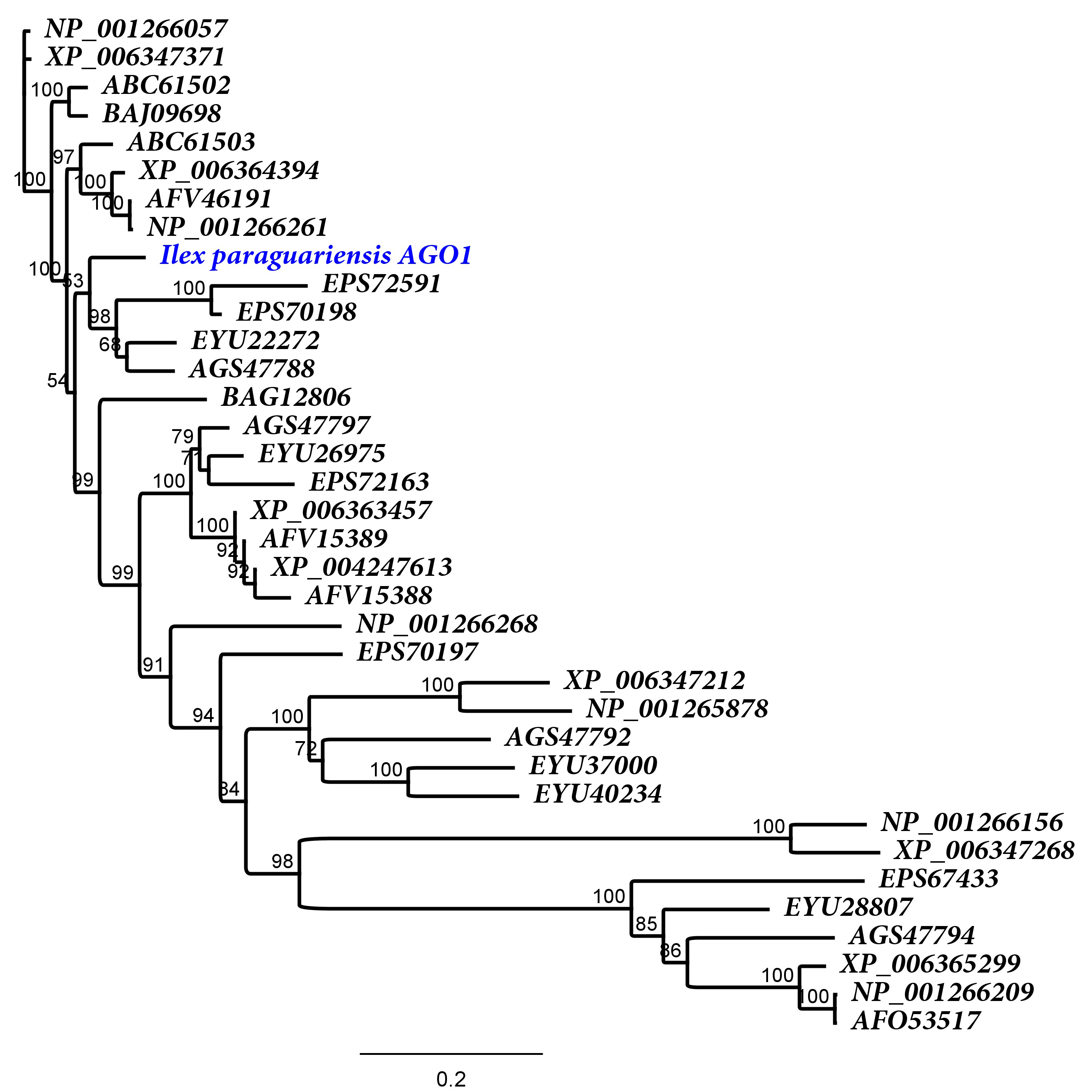

Supplement: Figure S14 — Bayesian phylogenic tree of the Argonaute 1 (AGO1) genes of 35 plant species and yerba mate determined by the Geneious 7.0 platform. Values at the nodes indicate bootstrap support percentage obtained for 1,000 replicates. (TIF) [file pone.0109835.s014.tif]

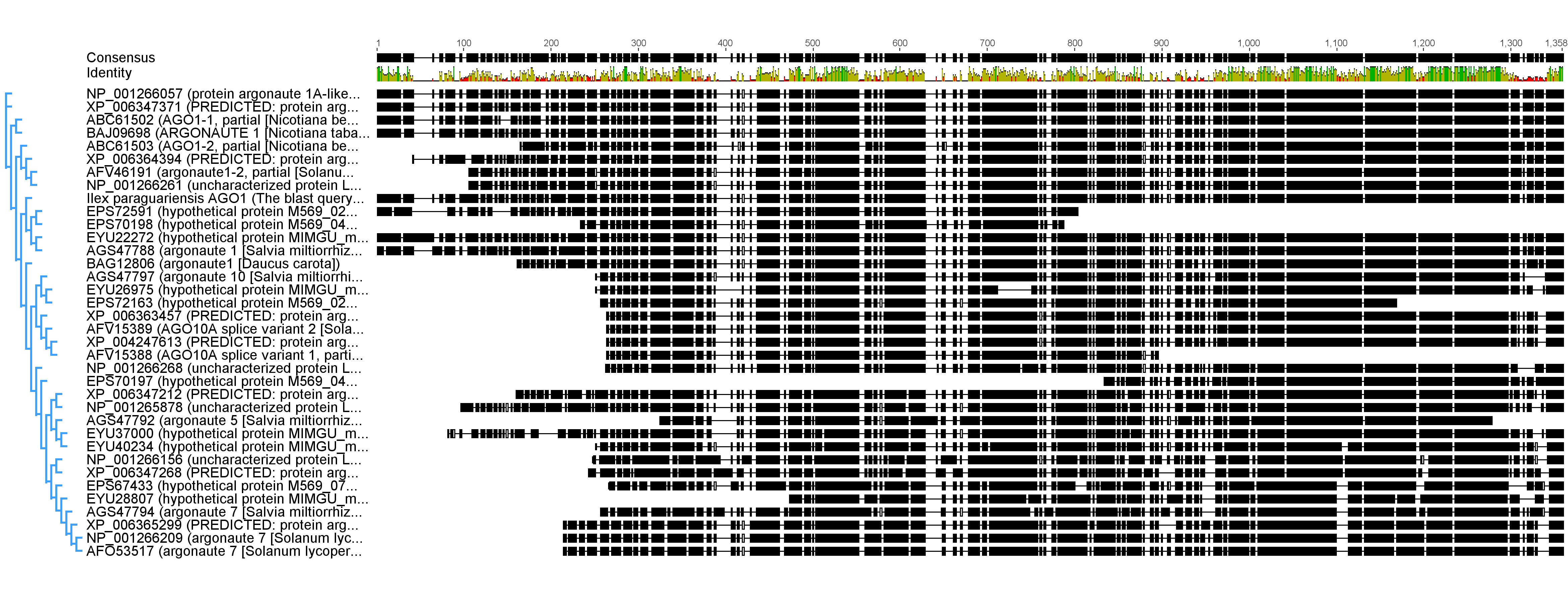

Supplement: Figure S15 — Multiple gene alignment of Argonaute 1 (AGO1) genes of 35 plant species and yerba mate. Identity is obtained based in 1 nt sliding window size and represented by color and bar height from 0% (red) to 100% (green). (TIF) [file pone.0109835.s015.tif]

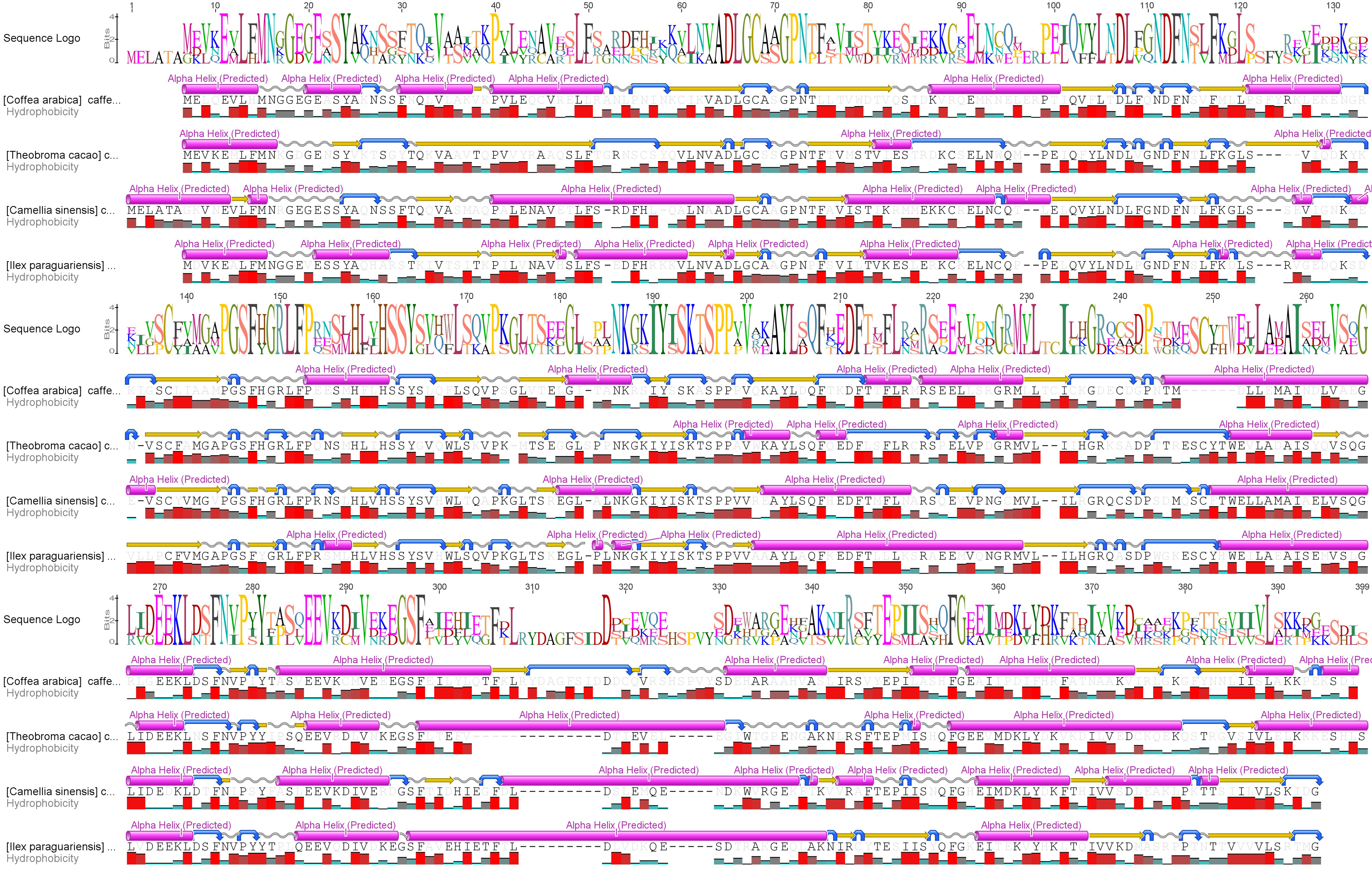

Supplement: Figure S16 — Multiple MUSCLE protein alignment and secondary structure prediction of Coffea arabica , Theobroma cacao , Camellia sinensis and Ilex paraguariensis caffeine synthase showing an important conservation in gene structure and domains. (TIF) [file pone.0109835.s016.tif]
