# Supplementary material for: Exploring the Genes of Yerba Mate (Ilex paraguariensis A. St.-Hil.) by NGS and De Novo Transcriptome Assembly
Source: PLoS One. 2014 Oct 16;9(10):e109835. doi: 10.1371/journal.pone.0109835 (PMC4199719; doi:10.1371/journal.pone.0109835)
Supplement: Figure S17 — SWISS-MODEL report of yerba mate caffeine synthase 3D prediction using Coffea arabica CS as a template. (PDF) [file pone.0109835.s017.pdf]

SWISS-MODEL Workspace

Workunit: P000004   Title:ilex cs con template

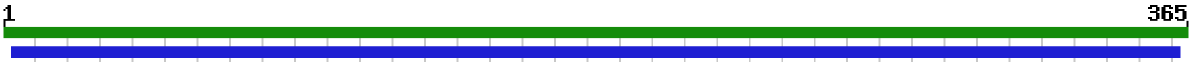

Model Summary:

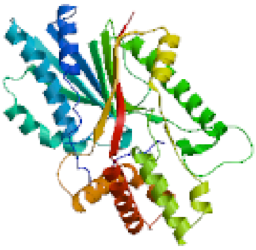

Model information:

Modelled residue range:	3 to 363
Based on template:	2efjA (2.00 A)
	**Remark:** No search for template was performed. Only user specified template was used for modelling.
Sequence Identity [%]:	34.324
Evalue:	0

Quaternary structure information:

Template (2efj): DIMER
Model built: SINGLE CHAIN

Ligand information:

Ligands in the template: 37T: 1, ALA: 1.
Ligands in the model: none.

Quality information:

QMEAN Z-Score: -3.396

Global Model Quality Estimation:

QMEAN4 global scores:

QMEANscore4: 0.565

Estimated absolute model quality:

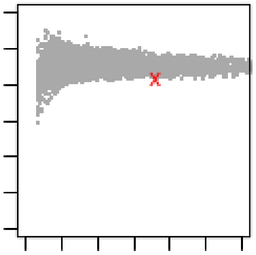

Z-Score: -3.396

Score components:

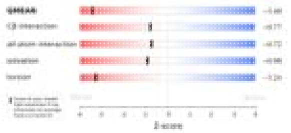

Local scores:

Coloring by residue error:

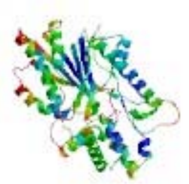

Residue error plot:

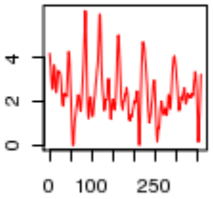

QMEAN4 global scores:

The QMEAN4 score is a composite score consisting of a linear combination of 4 statistical potential terms (estimated model reliability between 0-1). The pseudo-energies of the contributing terms are given below together with their Z-scores with respect to scores obtained for high-resolution experimental structures of similar size solved by X-ray crystallography:

Scoring function term	Raw score	Z-score
C_beta interaction energy	-113.31	-0.77
All-atom pairwise energy	-8826.12	-0.72
Solvation energy	-26.53	-0.90
Torsion angle energy	-31.23	-3.24
QMEAN4 score	0.565	-3.40

If you publish results from QMEAN, please cite the following paper:
Benkert P, Biasini M, Schwede T. (2011). "Toward the estimation of the absolute quality of individual protein structure models." Bioinformatics, 27(3):343-50.

Local Model Quality Estimation:

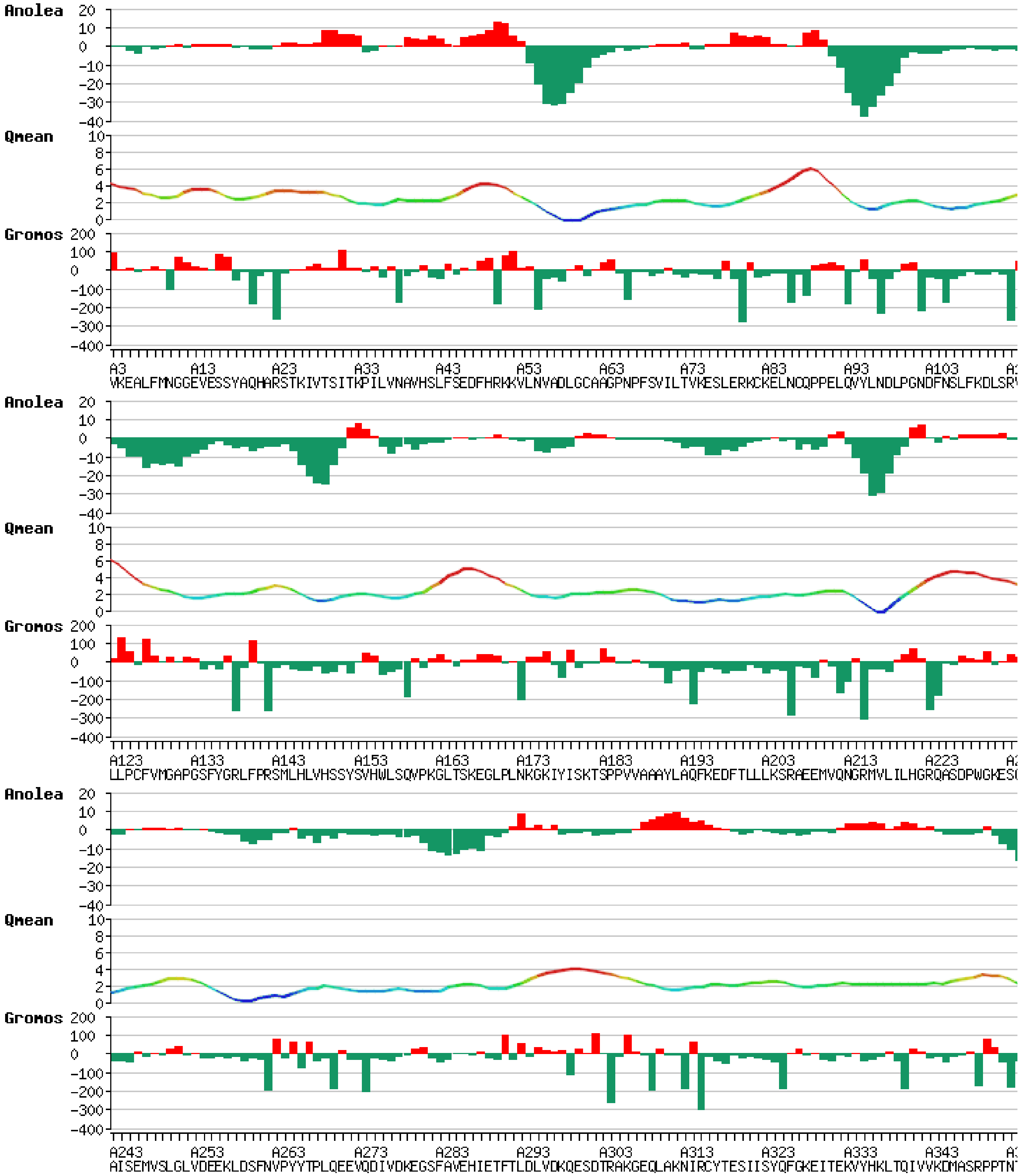

Alignment:

```
TARGET    1       VKEALFMN GGEVESSYAQ HARSTKIVTS ITKPILVNAV HSLFSEDFH-
2efjA     3       lqevlhmn gt----syak nssynlfli- rvkpvleqci qellranlpn

TARGET                            hhh h            hhhhhhhh hhhhh
2efjA                             hhh h      hh  hhhhhhhhhh hhhhh


TARGET    48    RKKVLNVADL GCAAGPNPFS VILTVKESLE RKCKELNCQP PELQVYLNDL
2efjA     50    inkcfkvgdl gcasgpntfs tvrdivqsid --------kv ptiqiflndl

TARGET              ssssss s     hhhh hhhhhhhhhh hhhhhh      ssssssss
2efjA             ssssssss s     hhhh hhhhhhhhhh             ssssssss


TARGET    98    PGNDFNSLFK DLSRVGED-- -QKSDVLLPC FVMGAPGSFY GRLFPRSMLH
2efjA     102   fqndfnsvfk llpsfyrnle kengrkigsc ligampgsfy srlfpeesmh

TARGET               hhhhh hhhhhhhh           ss sssssss             s
2efjA                hhhh    hhhhhhhh hhh     ss sssssss             s


TARGET    145   LVHSSYSVHW LSQVPKGLTS KEGLPLNKGK IYISKTSPPV VAAAYLAQFK
2efjA     152   flhscyclhw lsqvpsgi-- ----svnkgc iysskasrpp iqkayldqft

TARGET          ssssss  ss s                             hh hhhhhhhhhh
2efjA           ssssss  ss s                             hh hhhhhhhhhh


TARGET    195   EDFTLLLKSR AEEMVQNGRM VLILHGRQAS DPWGKESCYH WEILAEAISE
2efjA     202   kdfttflrih seelisrgrm lltfickede fd----hpns mdllemsind

TARGET          hhhhhhhhhh hhh    sss ssssss              h hhhhhhhhhh
2efjA           hhhhhhhhhh hhh    sss ssssss              h hhhhhhhhhh


TARGET    245   MVSLGLVDEE KLDSFNVPYY TPLQEEVQDI VDKEGSFAVE HIETFTLDLV
2efjA     248   lvieghleee kldsfnvpiy apsteevkri veeegsfeil yletfnapyd

TARGET          hhhh     h hhhh   sss    hhhhhhh hhhh sssss sssssssss
2efjA           hhhh     h hhhh   sss    hhhhhhh hhhh sssss sssssssss


TARGET    295   DKQESD---- -TRAKGEQLA KNIRCYTESI ISYQFGKEIT EKVYHKLTQI
2efjA     298   agfsispvsc deharaahva svvrsiyepi lashfgeail pdlshriakn

TARGET                       hhhhhhhh hhhhh   hh hhhh   hhh hhhhhhhhhh
2efjA                   hh hhhhhhhhhh hhhhh   hh hhhh   hhh hhhhhhhhhh


TARGET    340   VVKDMASRPP TNTTVVVVLS RT
2efjA     357   aakvlrsgkg fydsviisla kkp

TARGET          hhhhhh   s ssssssssss ss
2efjA           hhhhhhh  s ssssssssss ss
```

Modeling Log:

```
3.70 (SP3)
Loading Template: 2efjA.pdb
Loading Raw Sequence
Loading Alignment: ./NXXX.align.submit.fasta
Removing HET groups from template structure
Refining Raw Sequence Alignment
ProModII: doing simple assignment of backbone
ProModII: adding blocking groups
Adding Missing Sidechains
AddPolar H
BuildDeletetedLoopsModel
connectivity problem (C-N > 3.0A) at residue: 11
Trying Ligating with anchor residues ASN 8 and GLU 11
Trying Ligating with anchor residues ASN 8 and VAL 12
Trying Ligating with anchor residues ASN 8 and GLU 13
Trying Ligating with anchor residues ASN 8 and SER 14
Trying Ligating with anchor residues ASN 8 and SER 15
Number of Ligations found: 500
all loops are bad; continuing CSP with larger segment
Trying Ligating with anchor residues MET 7 and SER 15
Number of Ligations found: 500
ACCEPTING loop  210: clash=   0 FF=        297.4 PP=  2.00
Trying Ligating with anchor residues PHE 46 and LYS 49
Number of Ligations found: 7
ACCEPTING loop    2: clash=   0 FF=        600.9 PP= -1.00
connectivity problem (C-N > 3.0A) at residue: 89
Trying Ligating with anchor residues GLN 86 and GLU 89
Trying Ligating with anchor residues CYS 85 and GLU 89
Trying Ligating with anchor residues ASN 84 and GLU 89
Trying Ligating with anchor residues LEU 83 and GLU 89
Trying Ligating with anchor residues GLU 82 and GLU 89
Trying Ligating with anchor residues LYS 81 and GLU 89
Trying Ligating with anchor residues CYS 80 and GLU 89
Trying Ligating with anchor residues LYS 79 and GLU 89
+++ Warning: Ligation Failed, SparePart will be inserted later
+++          It is usually the sign that the region is misaligned.
Trying Ligating with anchor residues ASP 115 and SER 118
Number of Ligations found: 5
ACCEPTING loop    3: clash=   0 FF=       -278.4 PP= -2.00
connectivity problem (C-N > 3.0A) at residue: 163
Trying Ligating with anchor residues LYS 160 and THR 163
Trying Ligating with anchor residues LYS 160 and SER 164
Trying Ligating with anchor residues LYS 160 and LYS 165
Trying Ligating with anchor residues LYS 160 and GLU 166
Trying Ligating with anchor residues LYS 160 and GLY 167
Trying Ligating with anchor residues LYS 160 and LEU 168
Trying Ligating with anchor residues LYS 160 and PRO 169
Number of Ligations found: 500
ACCEPTING loop  174: clash=   0 FF=         32.0 PP= -4.00
connectivity problem (C-N > 3.0A) at residue: 301
Trying Ligating with anchor residues GLU 298 and THR 301
Trying Ligating with anchor residues GLN 297 and THR 301
Number of Ligations found: 15
all loops are bad; continuing CSP with larger segment
Trying Ligating with anchor residues LYS 296 and THR 301
Number of Ligations found: 147
all loops are bad; continuing CSP with larger segment
Trying Ligating with anchor residues ASP 295 and THR 301
Number of Ligations found: 63
ACCEPTING loop   31: clash=   0 FF=       1162.3 PP= -3.00
Small Ligation (C-N <3.0A) ignored;
GROMOS will repair it at residue THR 302
Building CSP loop with anchor residues THR 27 and THR 30
Number of Ligations found: 8
ACCEPTING loop    0: clash=   0 FF=        263.7 PP= -1.00
Building CSP loop with anchor residues PRO 226 and SER 231
```

```
Number of Ligations found: 30
all loops are bad; continuing CSP with larger segment
Building CSP loop with anchor residues PRO 226 and CYS 232
Number of Ligations found: 500
ACCEPTING loop  308: clash=   0 FF=        195.7 PP= -1.00
Finding Spare-Part loop with anchor residues GLU 77 and PRO 88
connectivity problem --> including residue PRO 89
Finding Spare-Part loop with anchor residues GLU 77 and GLU 89
ACCEPTING loop  72 from 8ABP_  Clash=   2 FF=        -53.0 PP=120.73
BadPhi=   1 BadGX=   0 BadXP=   1 weakXP=   1 Score= 4.75  rms= 0.00
Optimizing Sidechains
Adding Hydrogens
Optimizing loops and OXT (nb = 47)
Final Total Energy:      -10339.067 KJ/mol
Dumping Sequence Alignment
```

Template Selection Log:

```
 - Start SMR-Pipeline in automated mode on BC2-cluster at Wed Feb 12 01:14:21 2014


 - User specified template structure by a PDB identifier(2efjA), entering user template mode

 - Aligning sequence of the user template structure with the target sequence using BLAST
 - Alignment quality between target and specified template is too low

 - Aligning sequence of the user template structure with the target sequence using HHSearch
 - Send 1 target-template alignments for modeling

 - @@@@@@@@@@@@@@@@@@@@@@@@@@@@@@@@@@@@@@@@@@@@@@@@@@@@@@@@@@@@@@@@@@@@@@@@@@@@@@@@
 -  *******************************************************************************
 - building model based on 2efjA (3-363) was successfull
 - Workspace Pipeline parameter

   Cut-off parameters to model the target based on a BLAST target-template alignment
    Evalue :                                    0.0001
    Minimum Template size (aa) for ranking :    25
    Minimum Sequence identity :                 60

   Cut-off parameters to model the target based on a HHSearch target-template alignment
    Evalue :                                    0.0001
    Probability :                               50
    MAC :                                       0.3

   Parameters for model selection
    Minimal number of uncovered target
     residues after BLAST to run HHSEARCH :     50
    Minimal number of uncovered target
     residues to model an additional template : 25

 - Finish SMR-Pipeline in automated mode on BC2-cluster at Wed Feb 12 01:42:07 2014
```

Quaternary Structure Annotation of the Template
2efj is annotated as DIMER
The oligomeric state of the structure was assigned by the authors of the corresponding PDB entry
The following biological unit was used to build the template structure: 2efj.pdb1.gz

Quaternary Structure Modelling of the Target Protein
The target and template sequences are too diverse (seqid: 34.324) to infer a conservation of the oligomeric state
Please use the advancend features of the SwissModel Project Mode

The target structure was calculated as SINGLE CHAIN

Ligand Modeling Log: Template's ligands section
Ligands in the template: 37T: 1, ALA: 1.
The template contains ligands that are not yet part of the pipeline. Ligands which are currently assessed are listed in the help page.
No ligands were included in the model.

References: If you publish results using SWISS-MODEL, please cite the following papers:

- Arnold K., Bordoli L., Kopp J., and Schwede T. (2006). The SWISS-MODEL Workspace: A web-based environment for protein structure homology modeling. Bioinformatics, 22,195-201.
- Schwede T, Kopp J, Guex N, and Peitsch MC (2003) SWISS-MODEL: an automated protein homology-modeling server. Nucleic Acids Research 31: 3381-3385.
- Guex, N. and Peitsch, M. C. (1997) SWISS-MODEL and the Swiss-PdbViewer: An environment for comparative protein modeling. Electrophoresis 18: 2714-2723.

Swiss Institute of Bioinformatics | About SWISS-MODEL | Privacy | Terms of use | News   Back to the Top

SWISS-MODEL is developed by the Protein Structure Bioinformatics group at the SIB - Swiss Institute of Bioinformatics & the Biozentrum University of Basel. © 2010.
