# Supplementary material for: Exploring the Genes of Yerba Mate (Ilex paraguariensis A. St.-Hil.) by NGS and De Novo Transcriptome Assembly
Source: PLoS One. 2014 Oct 16;9(10):e109835. doi: 10.1371/journal.pone.0109835 (PMC4199719; doi:10.1371/journal.pone.0109835)
Supplement: Data S2 — 166 KEGG pathways models graphs representing most of yerba mate annotated genes (in green). Images credits: Kanehisa Laboratories, Japan. (PDF) [file pone.0109835.s031.pdf]

2-OXOCARBOXYLIC ACID METABOLISM

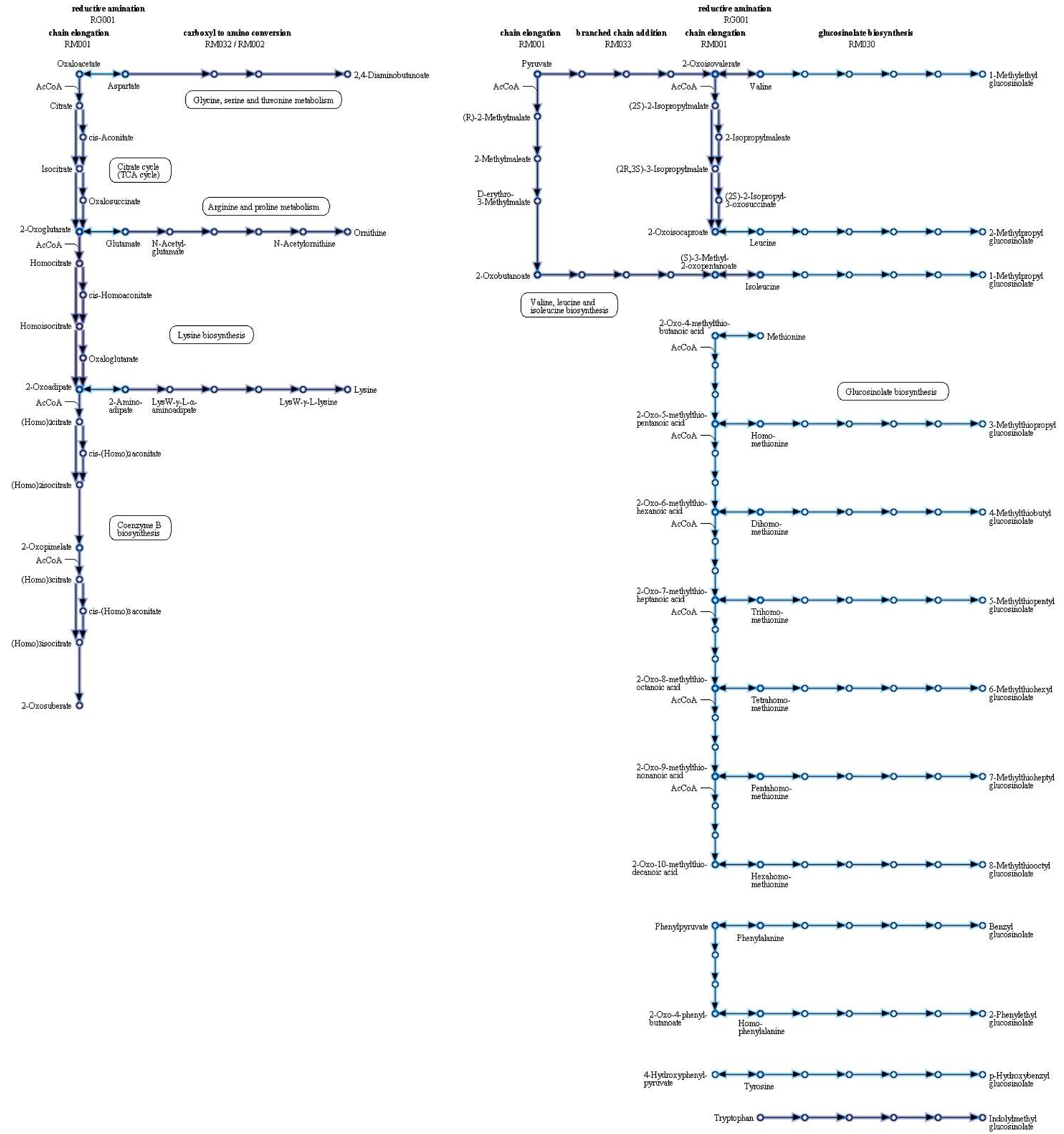

01210 11/25/13
(c) Kanehisa Laboratories

ABC TRANSPORTERS

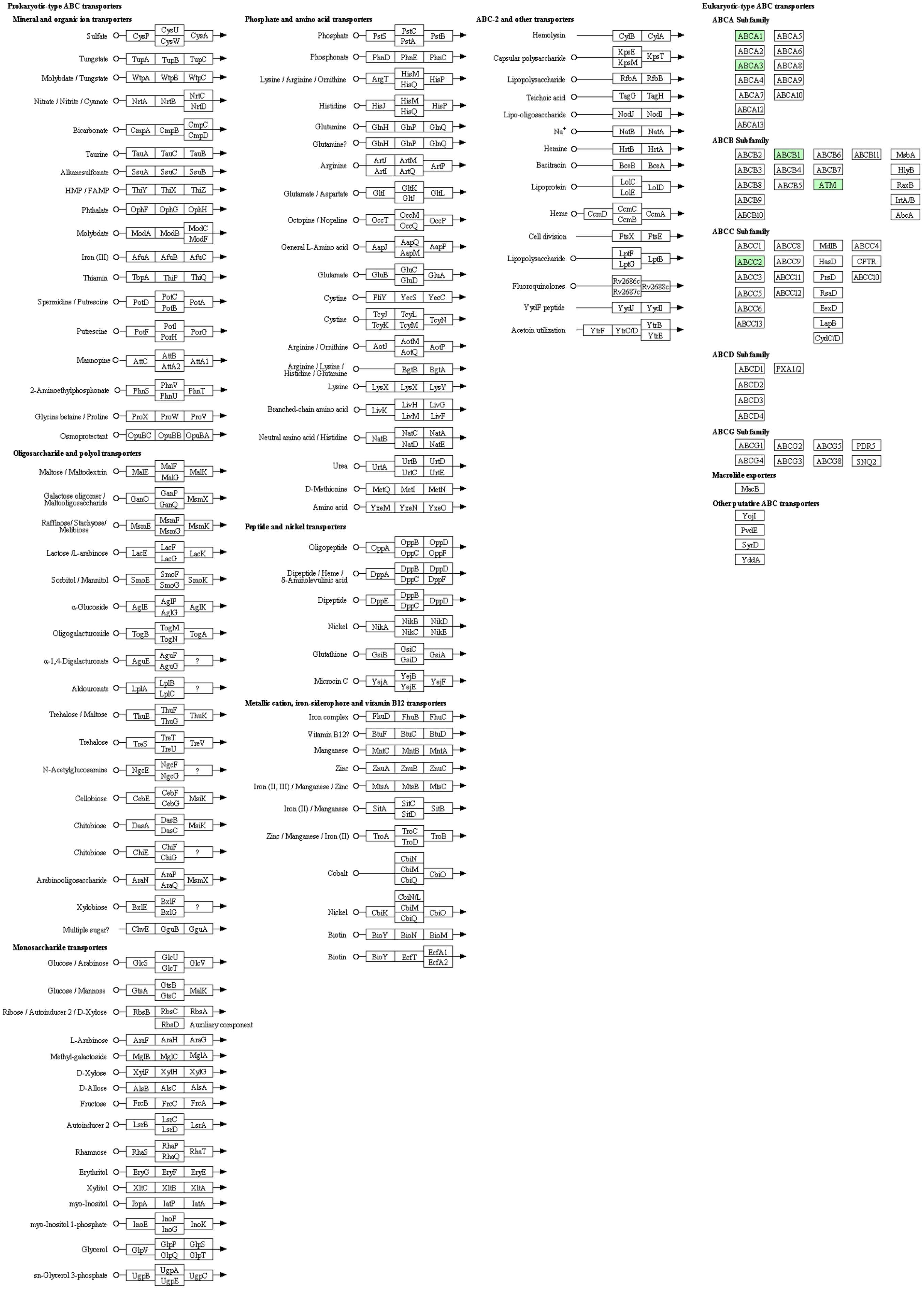

02010 3/12/14
(c) Kanehisa Laboratories

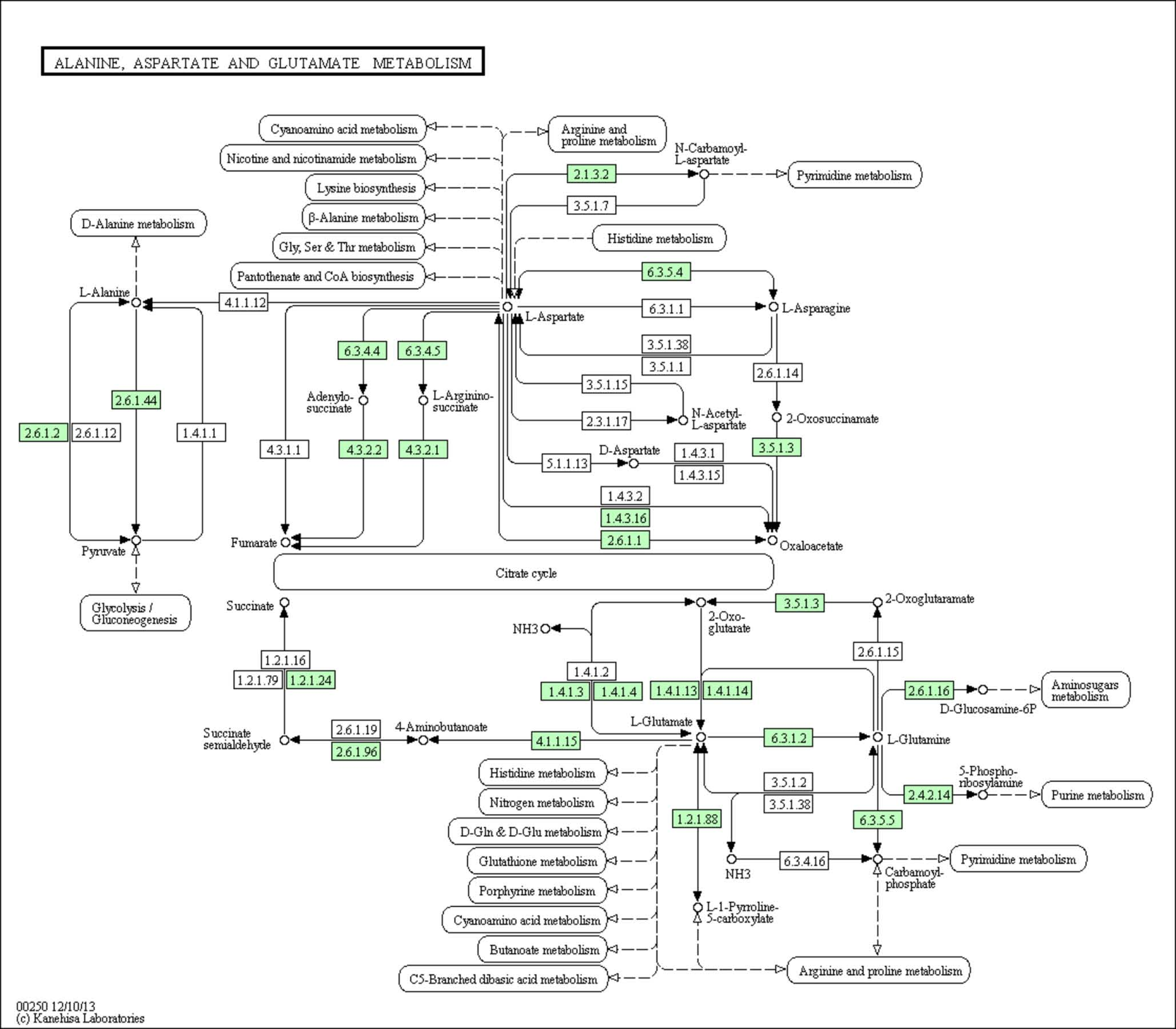
ALANINE, ASPARTATE AND GLUTAMATE METABOLISM
Cyanoamino acid metabolism
Nicotine and nicotinamide metabolism
Lysine biosynthesis
β-Alanine metabolism
Gly, Ser & Thr metabolism
Pantothenate and CoA biosynthesis
Arginine and proline metabolism
N-Carbamoyl-L-aspartate
Pyrimidine metabolism
2.1.3.2
3.5.1.7
Histidine metabolism
6.3.5.4
D-Alanine metabolism
L-Alanine
4.1.1.12
L-Aspartate
6.3.1.1
L-Asparagine
3.5.1.38
3.5.1.1
6.3.4.4
6.3.4.5
2.6.1.14
3.5.1.15
2.6.1.44
Adenylo-succinate
L-Argino-succinate
N-Acetyl-L-aspartate
2.3.1.17
2-Oxosuccinamate
2.6.1.2
2.6.1.12
1.4.1.1
4.3.1.1
4.3.2.2
4.3.2.1
3.5.1.3
D-Aspartate
1.4.3.1
5.1.1.13
1.4.3.15
1.4.3.2
1.4.3.16
2.6.1.1
Fumarate
Pyruvate
Oxaloacetate
Citrate cycle
Glycolysis / Gluconeogenesis
Succinate
3.5.1.3
2-Oxoglutaramate
2-Oxo-glutarate
NH3
2.6.1.15
1.2.1.16
1.4.1.2
1.2.1.79
1.2.1.24
1.4.1.3
1.4.1.4
1.4.1.13
1.4.1.14
2.6.1.16
Aminosugars metabolism
D-Glucosamine-6P
Succinate semialdehyde
2.6.1.19
4-Aminobutanoate
4.1.1.15
L-Glutamate
6.3.1.2
L-Glutamine
2.6.1.96
Histidine metabolism
5-Phospho-ribosylamine
3.5.1.2
Nitrogen metabolism
3.5.1.38
2.4.2.14
Purine metabolism
D-Gln & D-Glu metabolism
1.2.1.88
6.3.5.5
Glutathione metabolism
NH3
6.3.4.16
Pyrimidine metabolism
Carbamoyl-phosphate
Porphyrine metabolism
L-1-Pyrroline-5-carboxylate
Cyanoamino acid metabolism
Butanoate metabolism
C5-Branched dibasic acid metabolism
Arginine and proline metabolism
00250 12/10/13
(c) Kanehisa Laboratories

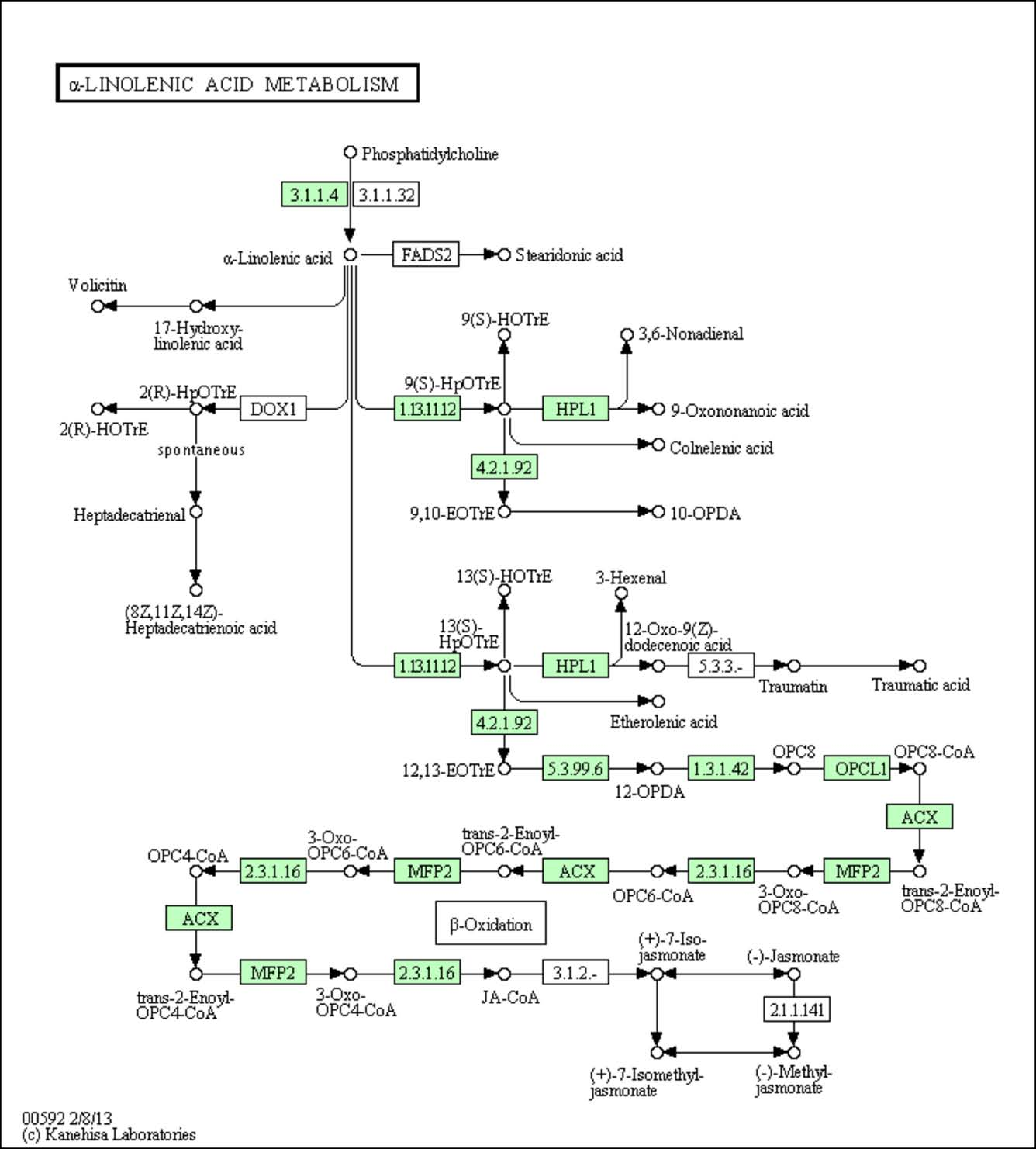
α-LINOLENIC ACID METABOLISM
Phosphatidylcholine
3.1.1.4
3.1.1.32
α-Linolenic acid
FADS2
Stearidonic acid
Volicitin
17-Hydroxy-linolenic acid
9(S)-HOTrE
3,6-Nonadienal
9(S)-HpOTrE
2(R)-HpOTrE
DOX1
1.13.11.12
HPL1
9-Oxononanoic acid
2(R)-HOTrE
spontaneous
Colnelenic acid
4.2.1.92
Heptadecatrienal
9,10-EOTrE
10-OPDA
13(S)-HOTrE
3-Hexenal
(8Z,11Z,14Z)-Heptadecatrienoic acid
13(S)-HpOTrE
12-Oxo-9(Z)-dodecenoic acid
1.13.11.12
HPL1
5.3.3.-
Traumatin
Traumatic acid
Etherolenic acid
4.2.1.92
OPC8
OPC8-CoA
12,13-EOTrE
5.3.99.6
1.3.1.42
OPCL1
12-OPDA
ACX
3-Oxo-OPC6-CoA
trans-2-Enoyl-OPC6-CoA
OPC4-CoA
2.3.1.16
MFP2
ACX
2.3.1.16
MFP2
OPC6-CoA
3-Oxo-OPC8-CoA
trans-2-Enoyl-OPC8-CoA
ACX
β-Oxidation
(+)-7-Iso-jasmonate
(-)-Jasmonate
MFP2
2.3.1.16
3.1.2.-
trans-2-Enoyl-OPC4-CoA
3-Oxo-OPC4-CoA
JA-CoA
2.1.1.141
(+)-7-Isomethyl-jasmonate
(-)-Methyl-jasmonate
00592 2/8/13
(c) Kanehisa Laboratories

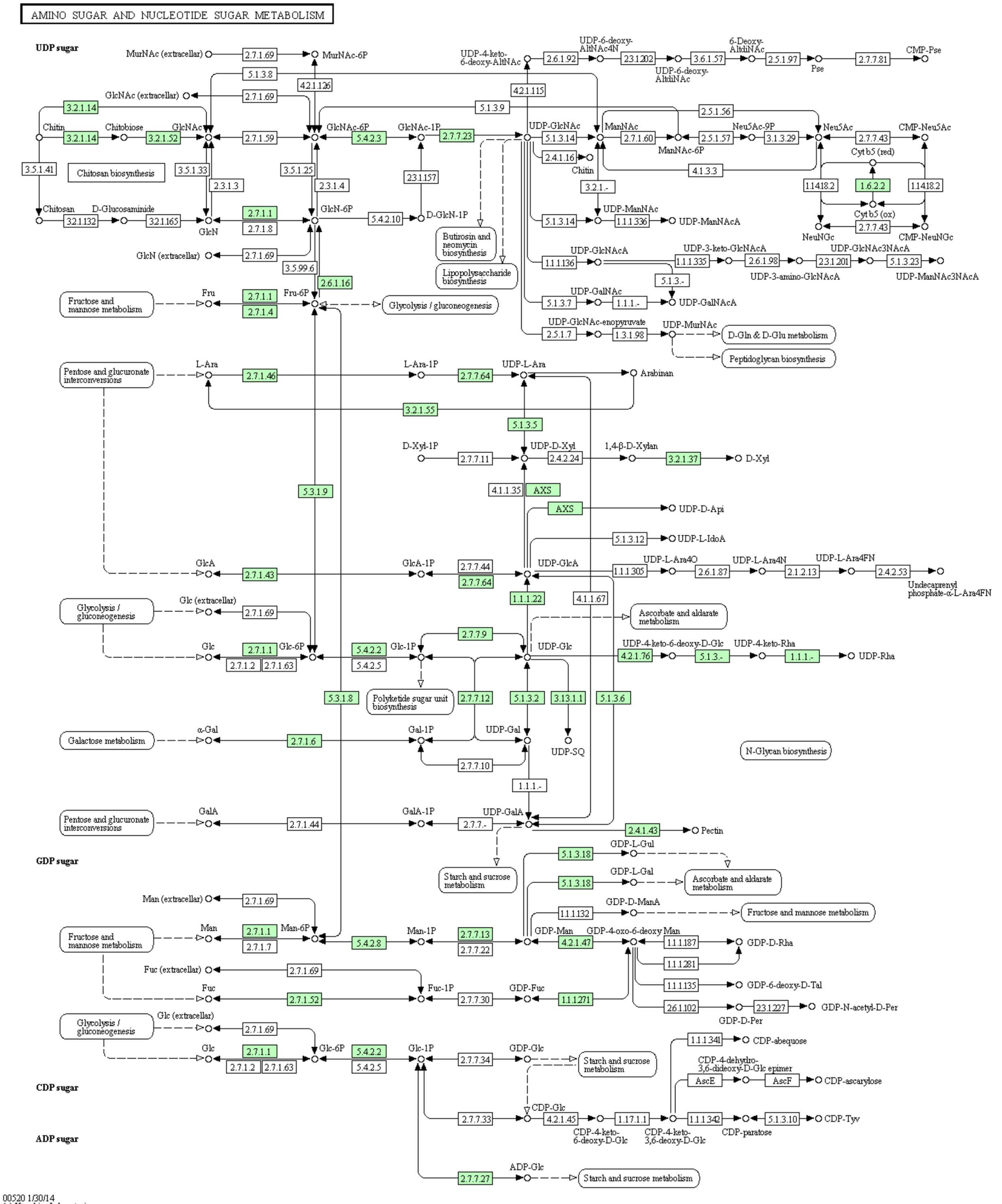

00520 1/30/14
(c) Kanehisa Laboratories

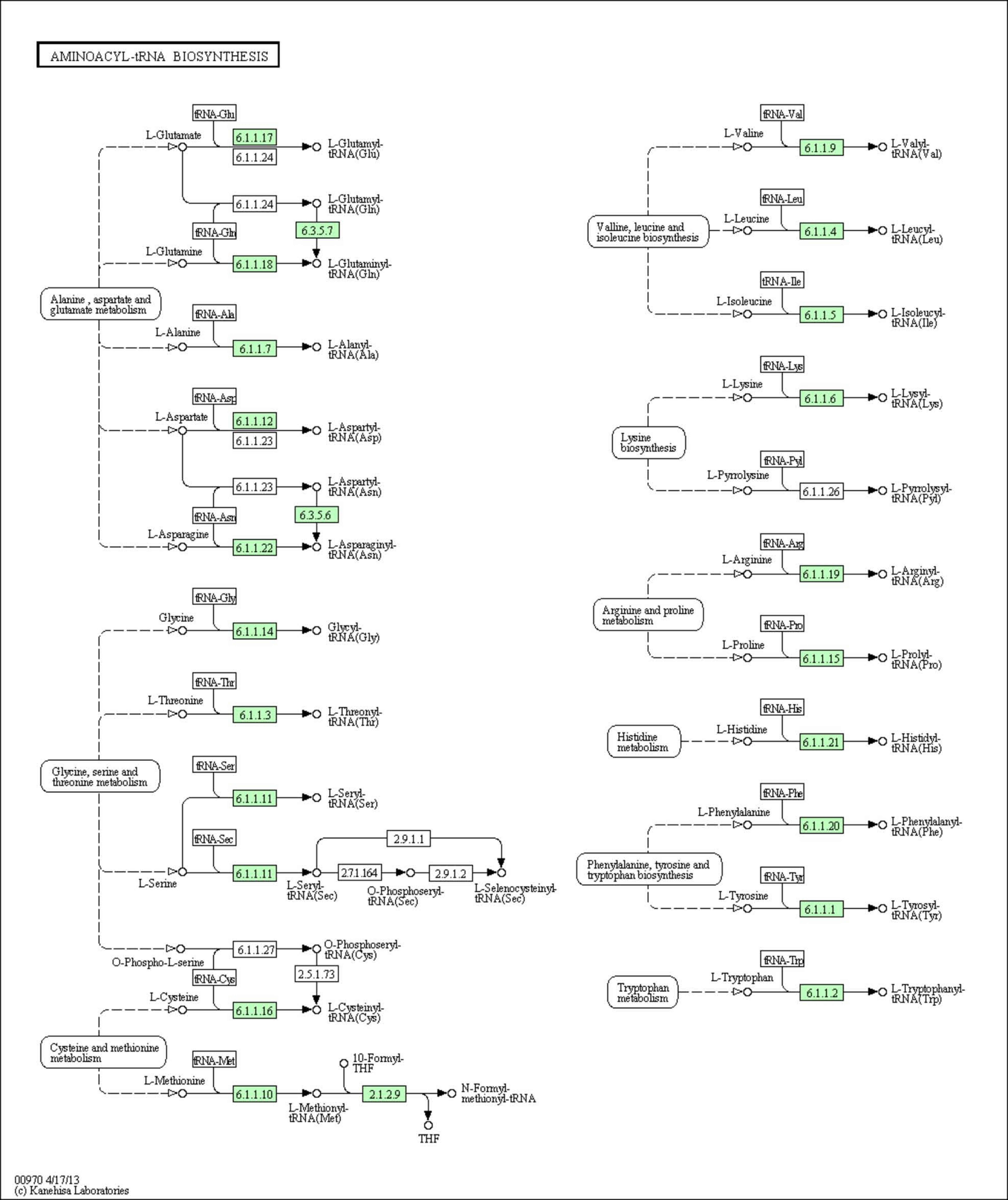
AMINOACYL-tRNA BIOSYNTHESIS
tRNA-Glu
L-Glutamate
6.1.1.17
6.1.1.24
L-Glutamyl-tRNA(Glu)
6.1.1.24
L-Glutamyl-tRNA(Gln)
6.3.5.7
tRNA-Gln
L-Glutamine
6.1.1.18
L-Glutaminyl-tRNA(Gln)
Alanine , aspartate and glutamate metabolism
tRNA-Ala
L-Alanine
6.1.1.7
L-Alanyl-tRNA(Ala)
tRNA-Asp
L-Aspartate
6.1.1.12
6.1.1.23
L-Aspartyl-tRNA(Asp)
6.1.1.23
L-Aspartyl-tRNA(Asn)
6.3.5.6
tRNA-Asn
L-Asparagine
6.1.1.22
L-Asparaginyl-tRNA(Asn)
tRNA-Gly
Glycine
6.1.1.14
Glycyl-tRNA(Gly)
tRNA-Thr
L-Threonine
6.1.1.3
L-Threonyl-tRNA(Thr)
Glycine, serine and threonine metabolism
tRNA-Ser
6.1.1.11
L-Seryl-tRNA(Ser)
tRNA-Sec
2.9.1.1
L-Serine
6.1.1.11
L-Seryl-tRNA(Sec)
2.7.1.164
O-Phosphoseryl-tRNA(Sec)
2.9.1.2
L-Selenocysteinyl-tRNA(Sec)
O-Phospho-L-serine
6.1.1.27
O-Phosphoseryl-tRNA(Cys)
2.5.1.73
tRNA-Cys
L-Cysteine
6.1.1.16
L-Cysteinyl-tRNA(Cys)
Cysteine and methionine metabolism
tRNA-Met
L-Methionine
6.1.1.10
L-Methionyl-tRNA(Met)
10-Formyl-THF
2.1.2.9
N-Formyl-methionyl-tRNA
THF
tRNA-Val
L-Valine
6.1.1.9
L-Valyl-tRNA(Val)
Valline, leucine and isoleucine biosynthesis
tRNA-Leu
L-Leucine
6.1.1.4
L-Leucyl-tRNA(Leu)
tRNA-Ile
L-Isoleucine
6.1.1.5
L-Isoleucyl-tRNA(Ile)
tRNA-Lys
L-Lysine
6.1.1.6
L-Lysyl-tRNA(Lys)
Lysine biosynthesis
tRNA-Pyl
L-Pyrrolysine
6.1.1.26
L-Pyrrolysyl-tRNA(Pyl)
tRNA-Arg
L-Arginine
6.1.1.19
L-Arginyl-tRNA(Arg)
Arginine and proline metabolism
tRNA-Pro
L-Proline
6.1.1.15
L-Prolyl-tRNA(Pro)
tRNA-His
Histidine metabolism
L-Histidine
6.1.1.21
L-Histidyl-tRNA(His)
tRNA-Phe
L-Phenylalanine
6.1.1.20
L-Phenylalanyl-tRNA(Phe)
Phenylalanine, tyrosine and tryptophan biosynthesis
tRNA-Tyr
L-Tyrosine
6.1.1.1
L-Tyrosyl-tRNA(Tyr)
tRNA-Trp
Tryptophan metabolism
L-Tryptophan
6.1.1.2
L-Tryptophanyl-tRNA(Trp)
00970 4/17/13
(c) Kanehisa Laboratories

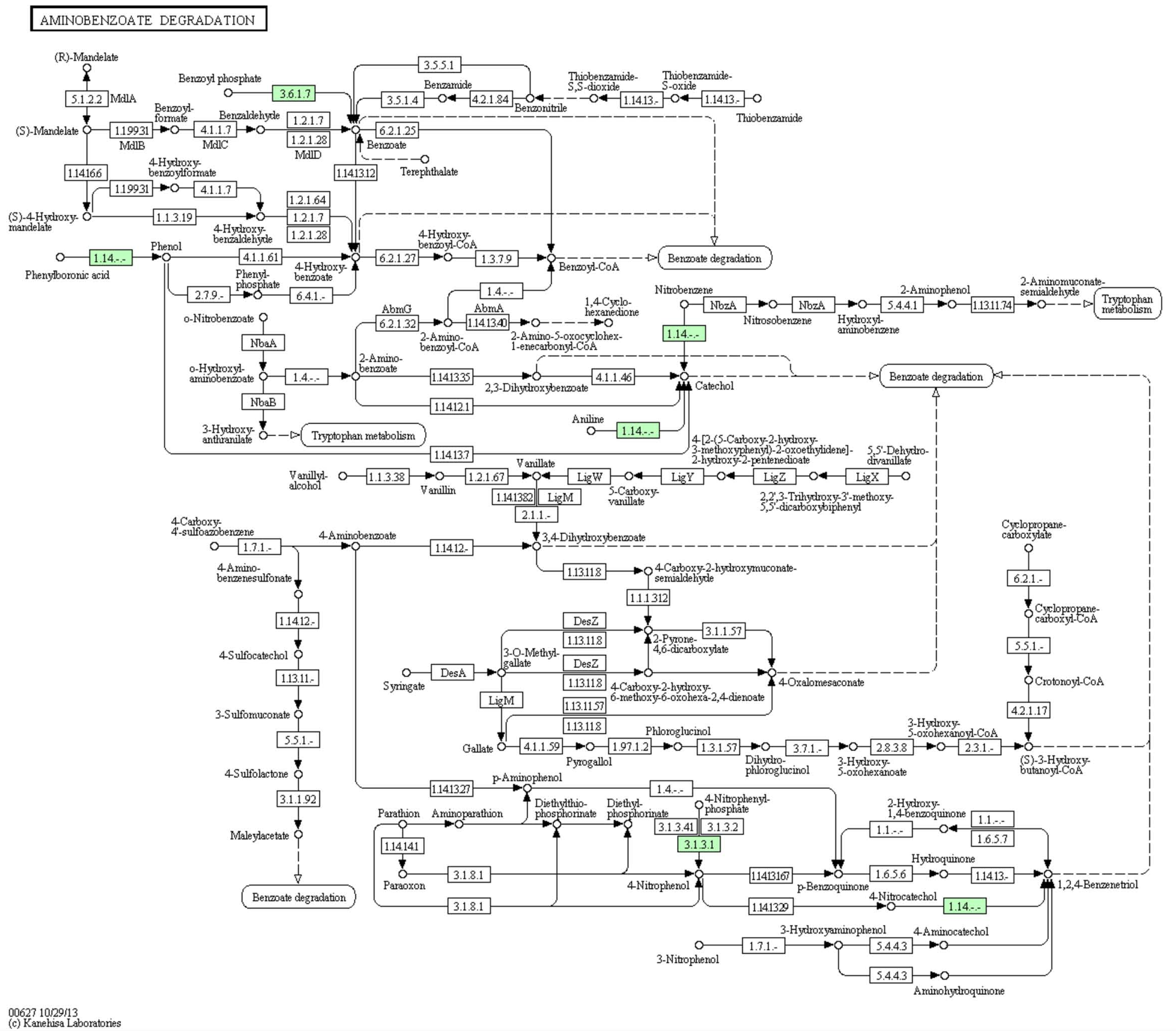
AMINOBENZOATE DEGRADATION
(R)-Mandelate
5.1.2.2
MdlA
(S)-Mandelate
1.1.99.31
MdlB
Benzoyl-formate
4.1.1.7
MdlC
Benzaldehyde
1.2.1.7
1.2.1.28
MdlD
Benzoyl phosphate
3.6.1.7
3.5.5.1
Benzamide
3.5.1.4
4.2.1.84
Benzonitrile
Thiobenzamide-S,S-dioxide
1.14.13.-
Thiobenzamide-S-oxide
1.14.13.-
Thiobenzamide
6.2.1.25
Benzoate
Terephthalate
1.14.13.12
1.14.16.6
4-Hydroxy-benzoylformate
1.1.99.31
4.1.1.7
1.2.1.64
1.2.1.7
1.2.1.28
(S)-4-Hydroxy-mandelate
1.1.3.19
4-Hydroxy-benzaldehyde
Phenylboronic acid
1.14.-.-
Phenol
4.1.1.61
4-Hydroxy-benzoate
6.2.1.27
4-Hydroxy-benzoyl-CoA
1.3.7.9
Benzoyl-CoA
Benzoate degradation
2.7.9.-
Phenyl-phosphate
6.4.1.-
1.4.-.-
AbmG
6.2.1.32
AbmA
1.14.13.40
1,4-Cyclo-hexanedione
2-Amino-benzoyl-CoA
2-Amino-5-oxocyclohex-1-enecarbonyl-CoA
o-Nitrobenzoate
NbaA
o-Hydroxyl-aminobenzoate
1.4.-.-
2-Amino-benzoate
1.14.13.35
2,3-Dihydroxybenzoate
4.1.1.46
Catechol
1.14.12.1
NbaB
3-Hydroxy-anthranilate
Tryptophan metabolism
1.14.13.7
Nitrobenzene
NbzA
Nitrosobenzene
NbzA
Hydroxyl-aminobenzene
5.4.4.1
2-Aminophenol
1.13.11.74
2-Aminomuconate-semialdehyde
Tryptophan metabolism
1.14.-.-
Benzoate degradation
Aniline
1.14.-.-
4-[2-(5-Carboxy-2-hydroxy-3-methoxyphenyl)-2-oxoethylidene]-2-hydroxy-2-pentenedioate
5,5'-Dehydro-divanillate
Vanillyl-alcohol
1.1.3.38
Vanillin
1.2.1.67
Vanillate
LigW
5-Carboxy-vanillate
LigY
LigZ
LigX
2,2',3-Trihydroxy-3'-methoxy-5,5'-dicarboxybiphenyl
1.14.13.82
LigM
2.1.1.-
3,4-Dihydroxybenzoate
4-Carboxy-4'-sulfoazobenzene
1.7.1.-
4-Aminobenzoate
1.14.12.-
4-Amino-benzenesulfonate
1.14.12.-
4-Sulfocatechol
1.13.11.-
3-Sulfomuconate
5.5.1.-
4-Sulfolactone
3.1.1.92
Maleylacetate
Benzoate degradation
1.13.11.8
4-Carboxy-2-hydroxymuconate-semialdehyde
1.1.1.312
DesZ
1.13.11.8
2-Pyrone-4,6-dicarboxylate
3.1.1.57
3-O-Methyl-gallate
DesZ
1.13.11.8
Syringate
DesA
4-Carboxy-2-hydroxy-6-methoxy-6-oxohexa-2,4-dienoate
4-Oxalomesaconate
LigM
1.13.11.57
1.13.11.8
Gallate
4.1.1.59
Pyrogallol
1.97.1.2
Phloroglucinol
1.3.1.57
Dihydro-phloroglucinol
3.7.1.-
3-Hydroxy-5-oxohexanoate
2.8.3.8
3-Hydroxy-5-oxohexanoyl-CoA
2.3.1.-
(S)-3-Hydroxy-butanoyl-CoA
Cyclopropane-carboxylate
6.2.1.-
Cyclopropane-carboxyl-CoA
5.5.1.-
Crotonoyl-CoA
4.2.1.17
1.14.13.27
p-Aminophenol
1.4.-.-
Parathion
Aminoparathion
Diethylthio-phosphorinate
Diethyl-phosphorinate
4-Nitrophenyl-phosphate
3.1.3.41
3.1.3.2
3.1.3.1
1.14.14.1
Paraoxon
3.1.8.1
4-Nitrophenol
3.1.8.1
1.14.13.167
p-Benzoquinone
2-Hydroxy-1,4-benzoquinone
1.1.-.-
1.1.-.-
1.6.5.7
1.6.5.6
Hydroquinone
1.14.13.-
1,2,4-Benzenetriol
1.14.13.29
4-Nitrocatechol
1.14.-.-
3-Nitrophenol
1.7.1.-
3-Hydroxyaminophenol
5.4.4.3
4-Aminocatechol
5.4.4.3
Aminohydroquinone
00627 10/29/13
(c) Kanehisa Laboratories

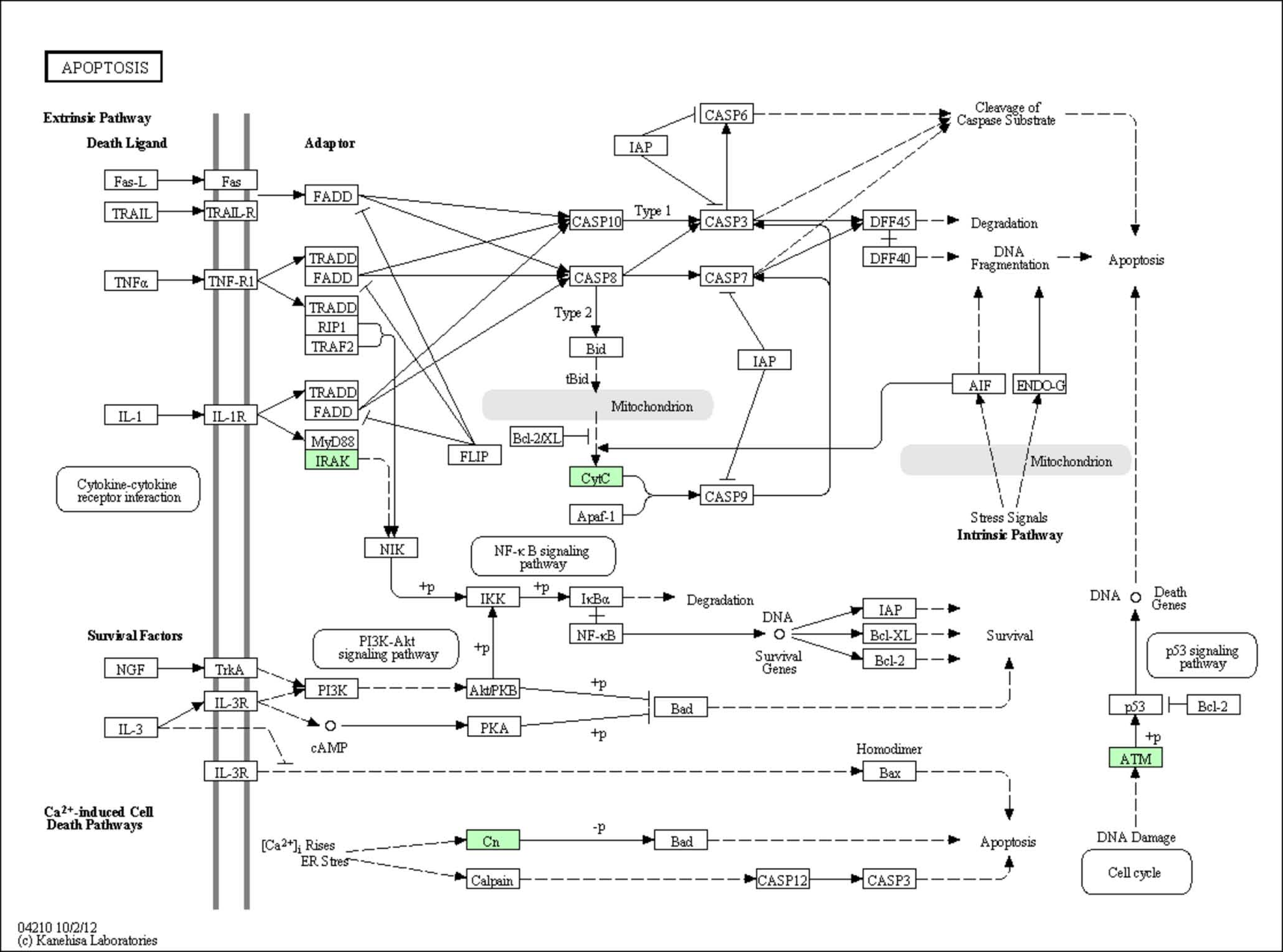
APOPTOSIS
Extrinsic Pathway
Death Ligand
Adaptor
Fas-L
Fas
TRAIL
TRAIL-R
FADD
TNFα
TNF-R1
TRADD
FADD
TRADD
RIP1
TRAF2
IL-1
IL-1R
TRADD
FADD
MyD88
IRAK
FLIP
Cytokine-cytokine receptor interaction
CASP6
IAP
Cleavage of Caspase Substrate
CASP10
Type 1
CASP3
DFF45
Degradation
DFF40
DNA Fragmentation
Apoptosis
CASP8
CASP7
Type 2
Bid
tBid
IAP
Mitochondrion
Bcl-2/XL
CytC
Apaf-1
CASP9
AIF
ENDO-G
Mitochondrion
Stress Signals
Intrinsic Pathway
NIK
NF-κB signaling pathway
+p
IKK
+p
IκBα
Degradation
NF-κB
DNA
Survival Genes
IAP
Bcl-XL
Bcl-2
Survival
DNA
Death Genes
p53 signaling pathway
Survival Factors
NGF
TrkA
PI3K-Akt signaling pathway
+p
IL-3R
PI3K
Akt/PKB
+p
Bad
IL-3
cAMP
PKA
+p
p53
Bcl-2
+p
ATM
IL-3R
Homodimer
Bax
Ca2+-induced Cell Death Pathways
[Ca2+]i Rises
ER Stres
Cn
-p
Bad
Apoptosis
DNA Damage
Calpain
CASP12
CASP3
Cell cycle
04210 10/2/12
(c) Kanehisa Laboratories

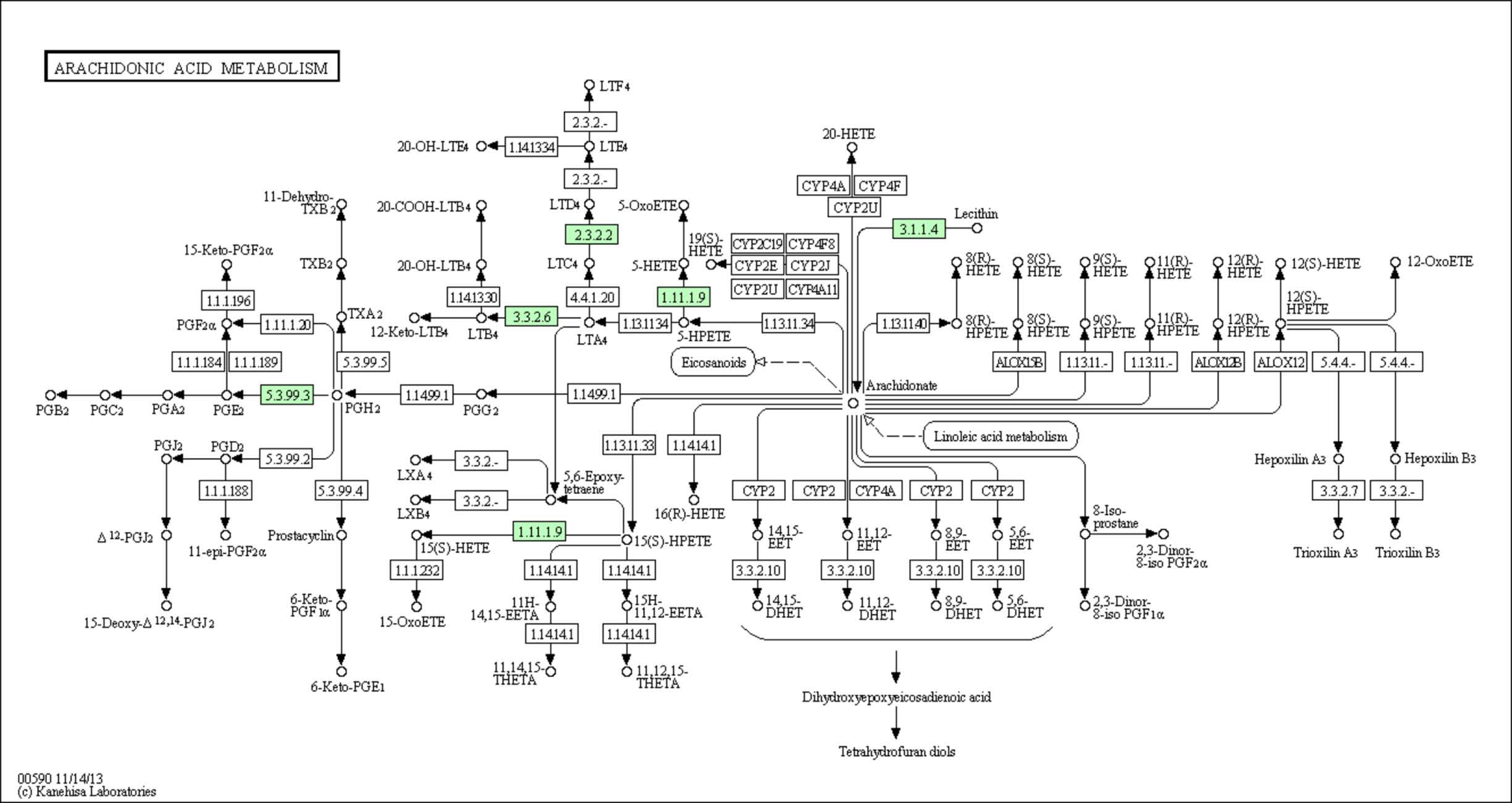
ARACHIDONIC ACID METABOLISM
LTF4
2.3.2.-
20-OH-LTE4
1.14.13.34
LTE4
2.3.2.-
11-Dehydro-TXB2
20-COOH-LTB4
LTD4
5-OxoETE
2.3.2.2
15-Keto-PGF2α
TXB2
20-OH-LTB4
LTC4
5-HETE
19(S)-HETE
20-HETE
CYP4A
CYP4F
CYP2U
Lecithin
3.1.1.4
CYP2C19
CYP4F8
CYP2E
CYP2J
CYP2U
CYP4A11
1.1.1.196
1.14.13.30
4.4.1.20
1.11.1.9
TXA2
PGF2α
1.1.1.20
3.3.2.6
12-Keto-LTB4
LTB4
LTA4
1.13.11.34
5-HPETE
1.13.11.34
1.13.11.40
8(R)-HETE
8(S)-HETE
9(S)-HETE
11(R)-HETE
12(R)-HETE
12(S)-HETE
12-OxoETE
12(S)-HPETE
8(R)-HPETE
8(S)-HPETE
9(S)-HPETE
11(R)-HPETE
12(R)-HPETE
1.1.1.184
1.1.1.189
5.3.99.5
Eicosanoids
ALOX15B
1.13.11.-
1.13.11.-
ALOX12B
ALOX12
5.4.4.-
5.4.4.-
PGB2
PGC2
PGA2
PGE2
5.3.99.3
PGH2
1.14.99.1
PGG2
1.14.99.1
Arachidonate
Linoleic acid metabolism
PGJ2
PGD2
5.3.99.2
1.13.11.33
1.14.14.1
3.3.2.-
LXA4
5,6-Epoxy-tetraene
Hepoxilin A3
Hepoxilin B3
1.1.1.188
5.3.99.4
3.3.2.-
LXB4
16(R)-HETE
CYP2
CYP2
CYP4A
CYP2
CYP2
3.3.2.7
3.3.2.-
Δ12-PGJ2
11-epi-PGF2α
Prostacyclin
1.11.1.9
15(S)-HETE
15(S)-HPETE
8-Iso-prostane
14,15-EET
11,12-EET
8,9-EET
5,6-EET
2,3-Dinor-8-iso PGF2α
Trioxilin A3
Trioxilin B3
1.1.1.232
1.14.14.1
1.14.14.1
3.3.2.10
3.3.2.10
3.3.2.10
3.3.2.10
6-Keto-PGF1α
11H-14,15-EETA
15H-11,12-EETA
14,15-DHET
11,12-DHET
8,9-DHET
5,6-DHET
2,3-Dinor-8-iso PGF1α
15-Deoxy-Δ12,14-PGJ2
15-OxoETE
1.14.14.1
1.14.14.1
11,14,15-THETA
11,12,15-THETA
6-Keto-PGE1
Dihydroxyepoxyeicosadienoic acid
Tetrahydrofuran diols
00590 11/14/13
(c) Kanehisa Laboratories

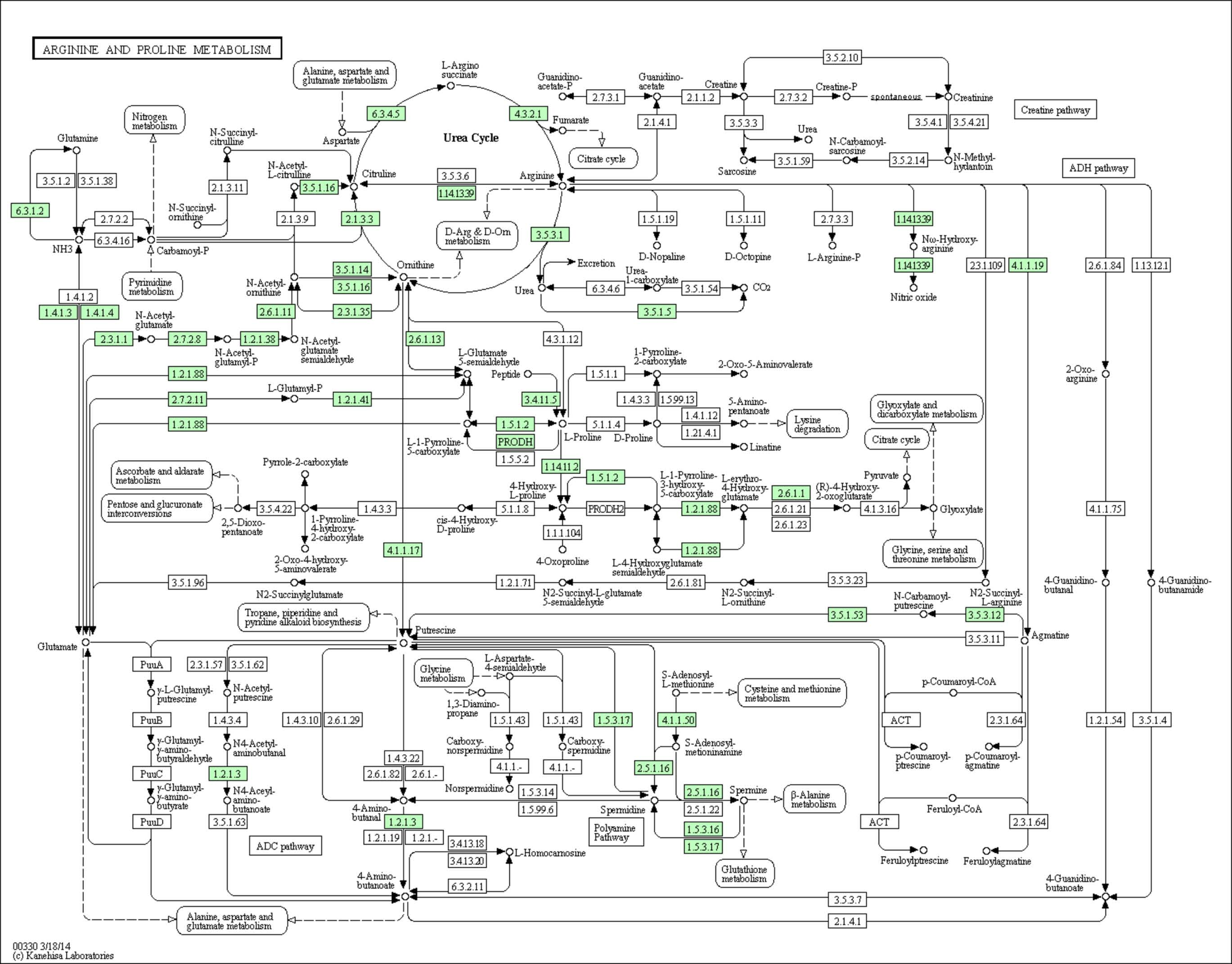
ARGININE AND PROLINE METABOLISM
Alanine, aspartate and glutamate metabolism
L-Argino succinate
Guanidino-acetate-P
2.7.3.1
Guanidino-acetate
2.1.1.2
Creatine
3.5.2.10
2.7.3.2
Creatine-P
spontaneous
Creatinine
Creatine pathway
6.3.4.5
4.3.2.1
Fumarate
2.1.4.1
3.5.3.3
Urea
3.5.4.1
3.5.4.21
Nitrogen metabolism
Urea Cycle
Citrate cycle
N-Carbamoyl-sarcosine
3.5.2.14
N-Methyl-hydantoin
Glutamine
N-Succinyl-citrulline
Aspartate
Sarcosine
3.5.1.59
ADH pathway
N-Acetyl-L-citrulline
Citrulline
3.5.3.6
Arginine
3.5.1.2
3.5.1.38
2.1.3.11
3.5.1.16
1.14.13.39
6.3.1.2
N-Succinyl-ornithine
2.7.2.2
2.1.3.9
2.1.3.3
D-Arg & D-Orn metabolism
1.5.1.19
1.5.1.11
2.7.3.3
Nω-Hydroxy-arginine
3.5.3.1
6.3.4.16
NH3
Carbamoyl-P
D-Nopaline
D-Octopine
L-Arginine-P
Ornithine
Excretion
3.5.1.14
Urea-1-carboxylate
2.3.1.109
4.1.1.19
2.6.1.84
1.13.12.1
Pyrimidine metabolism
N-Acetyl-ornithine
Urea
6.3.4.6
3.5.1.54
CO2
Nitric oxide
1.4.1.2
1.4.1.3
1.4.1.4
2.6.1.11
2.3.1.35
3.5.1.5
N-Acetyl-glutamate
2.3.1.1
2.7.2.8
1.2.1.38
N-Acetyl-glutamate semialdehyde
N-Acetyl-glutamyl-P
2.6.1.13
4.3.1.12
L-Glutamate 5-semialdehyde
1-Pyrroline-2-carboxylate
2-Oxo-5-Aminovalerate
2-Oxo-arginine
1.2.1.88
Peptide
1.5.1.1
L-Glutamyl-P
2.7.2.11
1.2.1.41
3.4.11.5
1.4.3.3
1.5.99.13
5-Amino-pentanoate
Glyoxylate and dicarboxylate metabolism
1.4.1.12
Lysine degradation
1.5.1.2
5.1.1.4
1.21.4.1
PRODH
L-Proline
D-Proline
Citrate cycle
L-1-Pyrroline-5-carboxylate
1.5.5.2
Linatine
Pyrrole-2-carboxylate
1.14.11.2
Ascorbate and aldarate metabolism
L-1-Pyrroline-3-hydroxy-5-carboxylate
L-erythro-4-Hydroxy-glutamate
Pyruvate
4-Hydroxy-L-proline
2.6.1.1
(R)-4-Hydroxy-2-oxoglutarate
Pentose and glucuronate interconversions
3.5.4.22
1.4.3.3
5.1.1.8
PRODH2
2.6.1.21
4.1.3.16
Glyoxylate
4.1.1.75
2,5-Dioxo-pentanoate
1-Pyrroline-4-hydroxy-2-carboxylate
cis-4-Hydroxy-D-proline
2.6.1.23
1.1.1.104
4.1.1.17
Glycine, serine and threonine metabolism
2-Oxo-4-hydroxy-5-aminovalerate
4-Oxoproline
L-4-Hydroxyglutamate semialdehyde
3.5.3.23
3.5.1.96
1.2.1.71
2.6.1.81
4-Guanidino-butanal
4-Guanidino-butanamide
N2-Succinylglutamate
N2-Succinyl-L-glutamate 5-semialdehyde
N2-Succinyl-L-ornithine
N-Carbamoyl-putrescine
N2-Succinyl-L-arginine
Tropane, piperidine and pyridine alkaloid biosynthesis
3.5.1.53
3.5.3.12
Putrescine
3.5.3.11
Agmatine
Glutamate
PuuA
2.3.1.57
3.5.1.62
Glycine metabolism
L-Aspartate-4-semialdehyde
S-Adenosyl-L-methionine
p-Coumaroyl-CoA
γ-L-Glutamyl-putrescine
N-Acetyl-putrescine
1,3-Diamino-propane
Cysteine and methionine metabolism
PuuB
1.4.3.4
1.4.3.10
2.6.1.29
1.5.1.43
1.5.1.43
1.5.3.17
4.1.1.50
ACT
2.3.1.64
1.2.1.54
3.5.1.4
γ-Glutamyl-γ-amino-butyraldehyde
N4-Acetyl-aminobutanal
Carboxy-norspermidine
Carboxy-spermidine
S-Adenosyl-metioninamine
p-Coumaroyl-ptrescine
p-Coumaroyl-agmatine
1.4.3.22
PuuC
1.2.1.3
2.6.1.82
2.6.1.-
4.1.1.-
4.1.1.-
2.5.1.16
γ-Glutamyl-γ-amino-butyrate
N4-Aceyl-amino-butanoate
Norspermidine
1.5.3.14
Spermine
β-Alanine metabolism
Feruloyl-CoA
4-Amino-butanal
1.2.1.3
1.5.99.6
Spermidine
2.5.1.22
PuuD
3.5.1.63
ACT
2.3.1.64
1.2.1.19
1.2.1.-
Polyamine Pathway
1.5.3.16
ADC pathway
3.4.13.18
L-Homocarnosine
1.5.3.17
Feruloylptrescine
Feruloylagmatine
3.4.13.20
Glutathione metabolism
4-Amino-butanoate
6.3.2.11
4-Guanidino-butanoate
3.5.3.7
Alanine, aspartate and glutamate metabolism
2.1.4.1
00330 3/18/14
(c) Kanehisa Laboratories

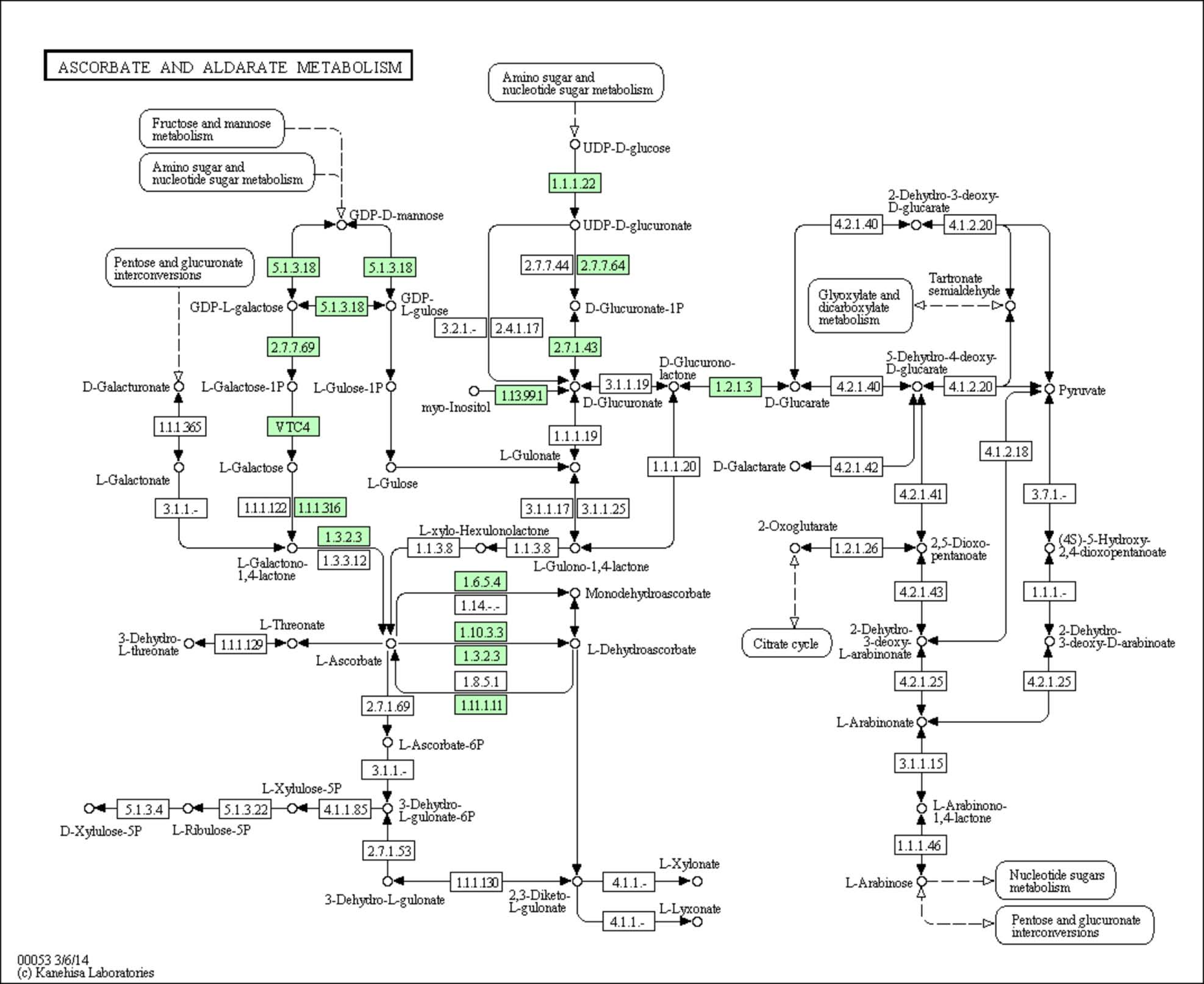

ASCORBATE AND ALDARATE METABOLISM
Fructose and mannose metabolism
Amino sugar and nucleotide sugar metabolism
Pentose and glucuronate interconversions
GDP-D-mannose
5.1.3.18
GDP-L-galactose
GDP-L-gulose
2.7.7.69
D-Galacturonate
L-Galactose-1P
L-Gulose-1P
1.1.1.365
VTC4
L-Galactonate
L-Galactose
L-Gulose
3.1.1.-
1.1.1.122
1.1.1.316
1.3.2.3
1.3.3.12
L-Galactono-1,4-lactone
UDP-D-glucose
1.1.1.22
UDP-D-glucuronate
2.7.7.44
2.7.7.64
D-Glucuronate-1P
3.2.1.-
2.4.1.17
2.7.1.43
myo-Inositol
1.13.99.1
D-Glucuronate
3.1.1.19
D-Glucurono-lactone
1.2.1.3
1.1.1.19
L-Gulonate
1.1.1.20
3.1.1.17
3.1.1.25
L-xylo-Hexulonolactone
1.1.3.8
L-Gulono-1,4-lactone
1.6.5.4
1.14.-.-
Monodehydroascorbate
1.10.3.3
1.8.5.1
1.11.1.11
L-Dehydroascorbate
L-Threonate
3-Dehydro-L-threonate
1.1.1.129
L-Ascorbate
2.7.1.69
L-Ascorbate-6P
3.1.1.-
L-Xylulose-5P
5.1.3.4
5.1.3.22
4.1.1.85
3-Dehydro-L-gulonate-6P
D-Xylulose-5P
L-Ribulose-5P
2.7.1.53
3-Dehydro-L-gulonate
1.1.1.130
2,3-Diketo-L-gulonate
4.1.1.-
L-Xylonate
L-Lyxonate
2-Dehydro-3-deoxy-D-glucarate
4.2.1.40
4.1.2.20
Glyoxylate and dicarboxylate metabolism
Tartronate semialdehyde
5-Dehydro-4-deoxy-D-glucarate
D-Glucarate
Pyruvate
D-Galactarate
4.2.1.42
4.1.2.18
4.2.1.41
3.7.1.-
2-Oxoglutarate
1.2.1.26
2,5-Dioxo-pentanoate
(4S)-5-Hydroxy-2,4-dioxopentanoate
4.2.1.43
1.1.1.-
Citrate cycle
2-Dehydro-3-deoxy-L-arabinonate
2-Dehydro-3-deoxy-D-arabinoate
4.2.1.25
L-Arabinonate
3.1.1.15
L-Arabinono-1,4-lactone
1.1.1.46
L-Arabinose
Nucleotide sugars metabolism
Pentose and glucuronate interconversions
00053 3/6/14
(c) Kanehisa Laboratories

BACTERIAL SECRETION SYSTEM

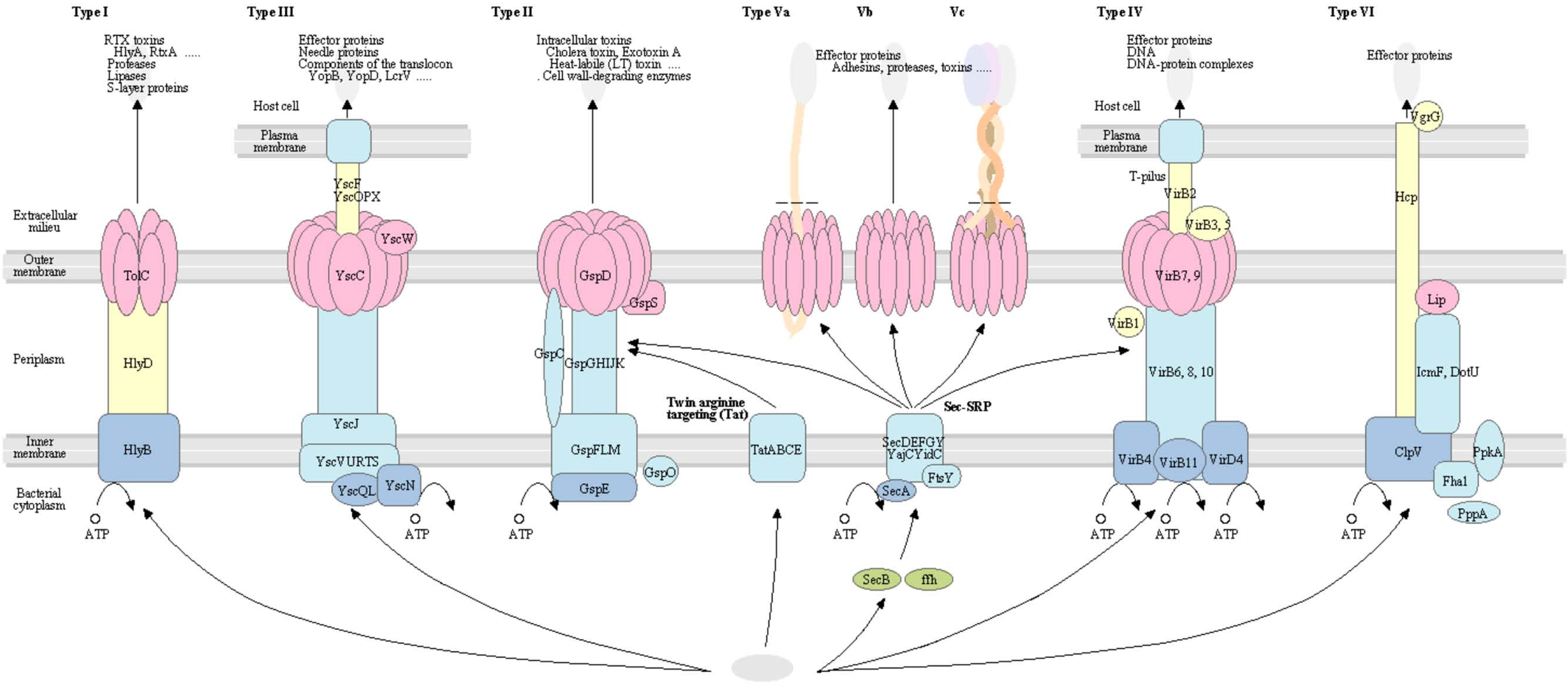

Type I

Outer membrane protein (OMP)	TolC
Membrane fusion protein	HlyD
ABC transporter	HlyB

Type III

Needle	YscF		
	YscO	YscP	YscX
Secretin	YscC		
OMP	YscW		
Inner membrane protein (IMP)	YscJ	YscR	YscS
	YscT	YscU	YscV
ATPase	YscN		
ATPase-associated protein	YscQ	YscL	

Type II

Secretin	GspD		
OMP	GspS		
IMP	GspC	GspF	GspG
	GspH	GspI	GspJ
	GspK	GspL	GspM
ATPase	GspE		
Leaderpeptidase	GspO		

Type Va **Vb** **Vc**

OMP	VacA	ShlB	YadA
		ShlA	YadB/C

Type IV

Periplasmic protein	VirB1		
Surface/pilus protein	VirB2	VirB3	VirB5
OMP	VirB7	VirB9	
IMP	VirB6	VirB8	VirB10
ATPase	VirB4	VirB11	VirD4

Type VI

Secreted substrate	VgrG		
	Hcp		
OMP	Lip		
IMP	IcmF	DotU	
ATPase	ClpV		
Regulatory protein	PpkA	Fha1	PppA

Sec-SRP

IMP	SecD/F	SecE	SecG
	SecY	YajC	YidC
ATPase	SecA		
Secretion monitor	SecM		
SRP receptor	FtsY		
Targeting protein	SecB	ffh	

Twin arginine targeting (Tat)

IMP	TatA	TatB	TatC	TatE

03070 4/12/10
(c) Kanehisa Laboratories

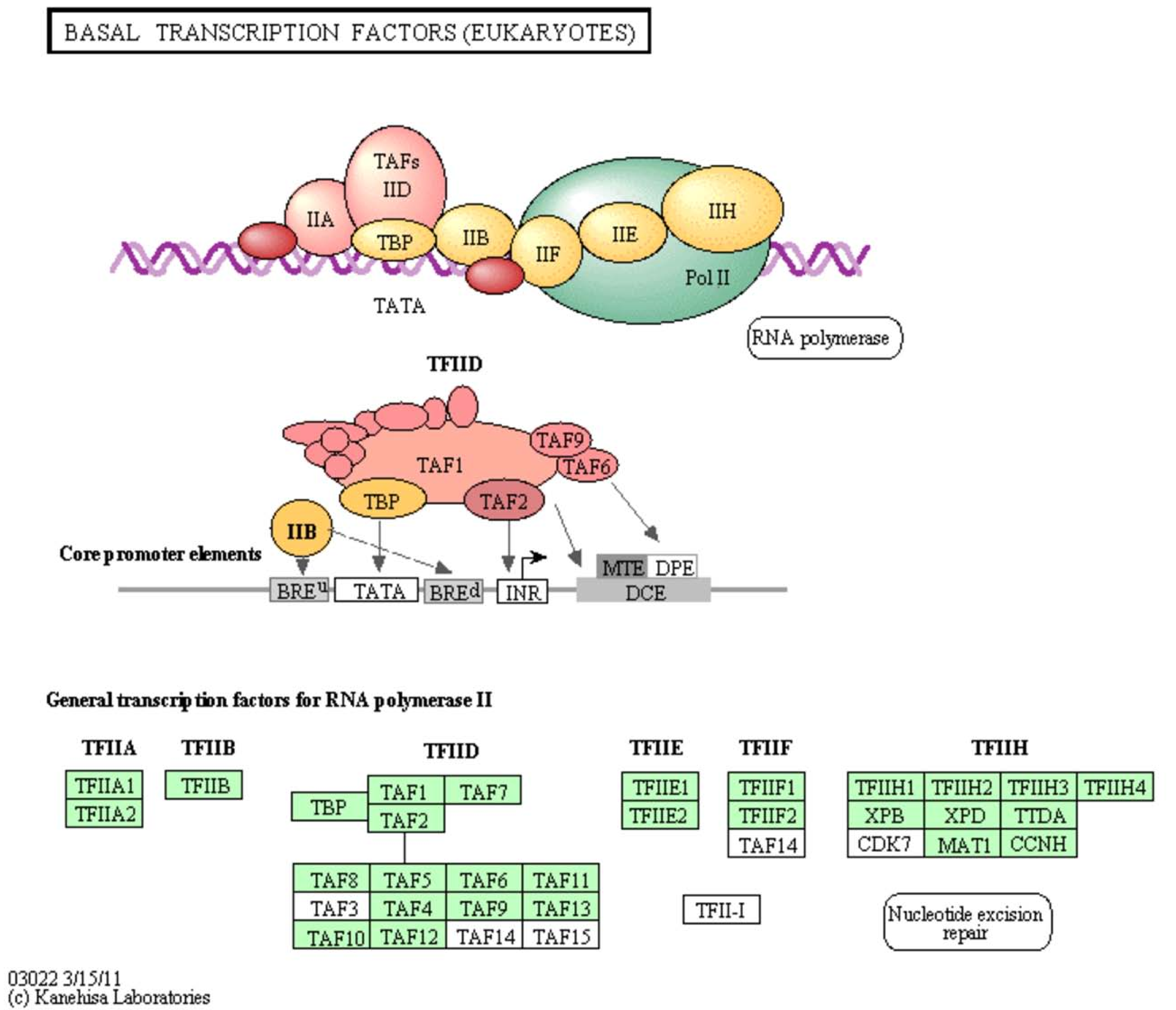

BASAL TRANSCRIPTION FACTORS (EUKARYOTES)
TAFs
IID
IIA
TBP
IIB
IIF
IIE
IIH
Pol II
TATA
RNA polymerase
TFIID
TAF9
TAF6
TAF1
TBP
TAF2
IIB
Core promoter elements
BRE u
TATA
BRE d
INR
MTE
DPE
DCE
General transcription factors for RNA polymerase II
TFIIA
TFIIA1
TFIIA2
TFIIB
TFIIB
TFIID
TBP
TAF1
TAF7
TAF2
TAF8
TAF5
TAF6
TAF11
TAF3
TAF4
TAF9
TAF13
TAF10
TAF12
TAF14
TAF15
TFIIE
TFIIE1
TFIIE2
TFIIF
TFIIF1
TFIIF2
TAF14
TFII-I
TFIIH
TFIIH1
TFIIH2
TFIIH3
TFIIH4
XPB
XPD
TTDA
CDK7
MAT1
CCNH
Nucleotide excision repair
03022 3/15/11
(c) Kanehisa Laboratories

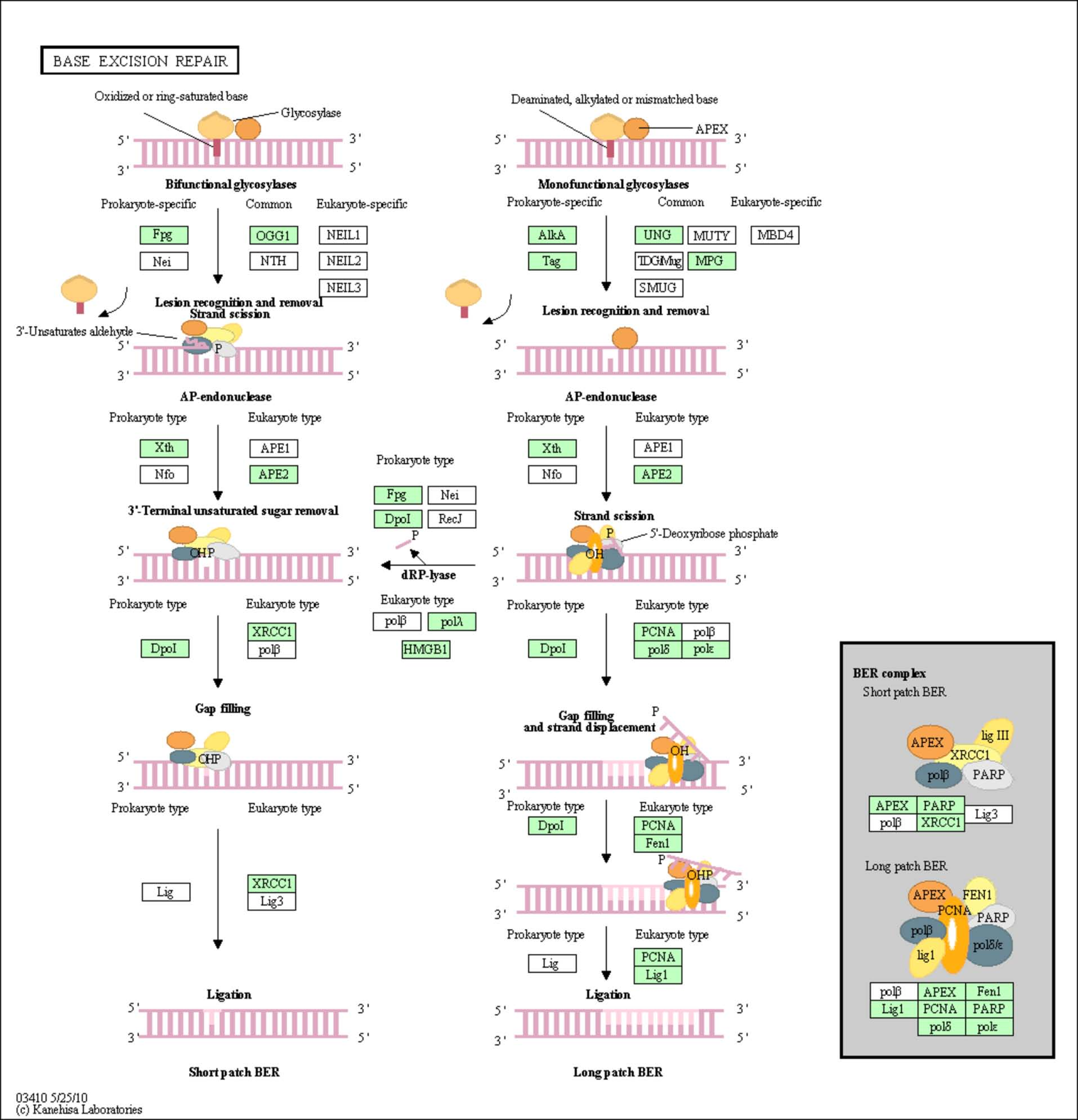
BASE EXCISION REPAIR
Oxidized or ring-saturated base
Glycosylase
Deaminated, alkylated or mismatched base
APEX
Bifunctional glycosylases
Prokaryote-specific
Common
Eukaryote-specific
Fpg
Nei
OGG1
NTH
NEIL1
NEIL2
NEIL3
Monofunctional glycosylases
AlkA
Tag
UNG
MUTY
MBD4
TDG/Mug
MPG
SMUG
Lesion recognition and removal
Strand scission
3'-Unsaturates aldehyde
AP-endonuclease
Prokaryote type
Eukaryote type
Xth
Nfo
APE1
APE2
3'-Terminal unsaturated sugar removal
Dpol
RecJ
dRP-lyase
polβ
polλ
HMGB1
Strand scission
5'-Deoxyribose phosphate
XRCC1
PCNA
polδ
polε
Gap filling
Gap filling and strand displacement
Fen1
Lig
Lig3
Lig1
Ligation
Short patch BER
Long patch BER
BER complex
Short patch BER
lig III
PARP
Long patch BER
FEN1
polδ/ε
lig1
03410 5/25/10
(c) Kanehisa Laboratories

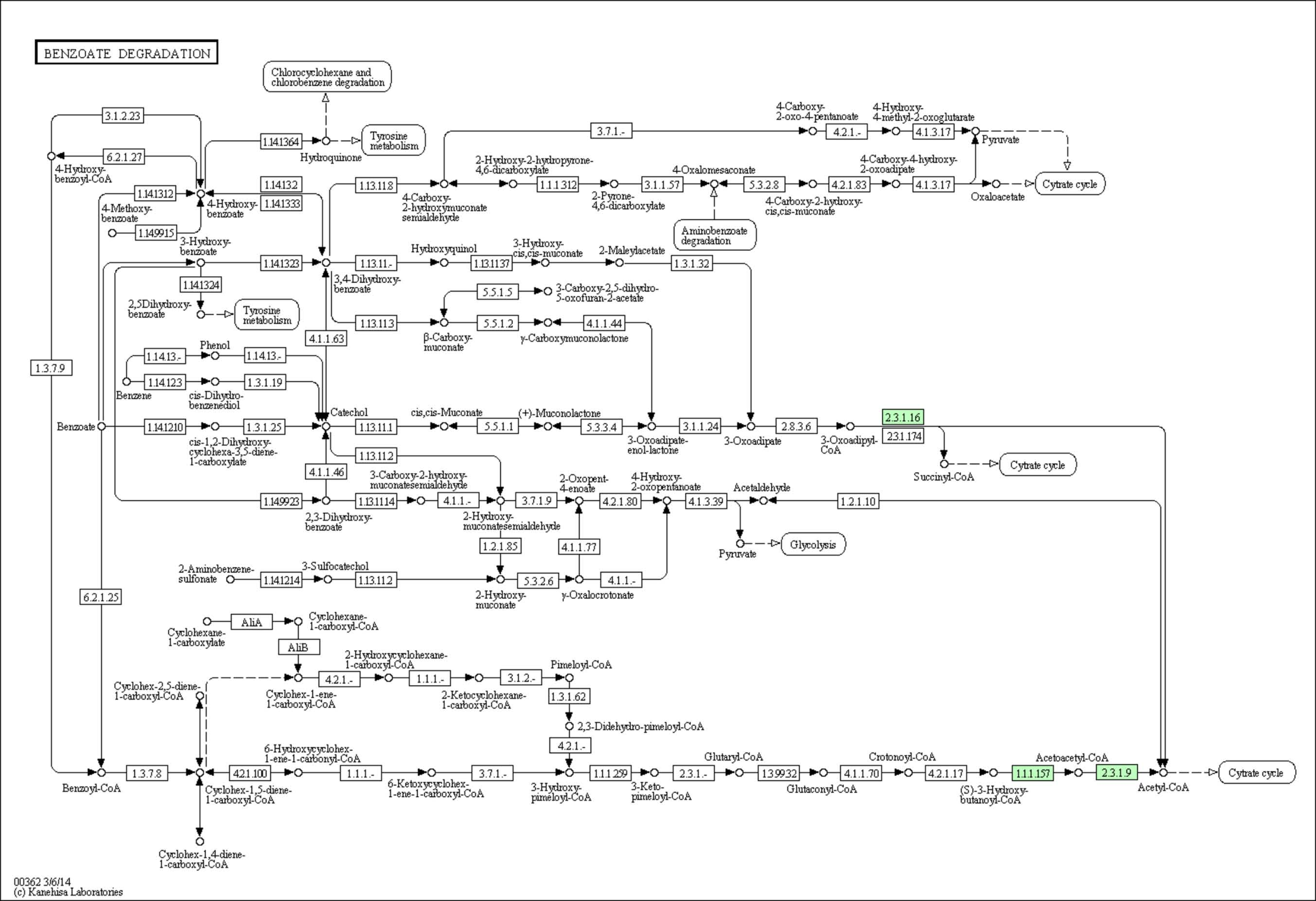
BENZOATE DEGRADATION
Chlorocyclohexane and chlorobenzene degradation
Tyrosine metabolism
Hydroquinone
3.1.2.23
6.2.1.27
4-Hydroxy-benzoyl-CoA
1.14.13.64
1.14.13.12
1.14.13.2
1.14.13.33
4-Hydroxy-benzoate
4-Methoxy-benzoate
1.14.99.15
3-Hydroxy-benzoate
1.14.13.23
1.14.13.24
2,5Dihydroxy-benzoate
Tyrosine metabolism
1.3.7.9
1.13.11.8
4-Carboxy-2-hydroxymuconate semialdehyde
2-Hydroxy-2-hydropyrone-4,6-dicarboxylate
1.1.1.312
2-Pyrone-4,6-dicarboxylate
3.1.1.57
4-Oxalomesaconate
Aminobenzoate degradation
5.3.2.8
4-Carboxy-2-hydroxy-cis,cis-muconate
4.2.1.83
4-Carboxy-4-hydroxy-2-oxoadipate
4.1.3.17
Oxaloacetate
Cytrate cycle
3.7.1.-
4-Carboxy-2-oxo-4-pentanoate
4.2.1.-
4-Hydroxy-4-methyl-2-oxoglutarate
4.1.3.17
Pyruvate
1.13.11.-
Hydroxyquinol
1.13.11.37
3-Hydroxy-cis,cis-muconate
2-Maleylacetate
1.3.1.32
3,4-Dihydroxy-benzoate
5.5.1.5
3-Carboxy-2,5-dihydro-5-oxofuran-2-acetate
1.13.11.3
β-Carboxy-muconate
5.5.1.2
γ-Carboxymuconolactone
4.1.1.44
4.1.1.63
Phenol
1.14.13.-
1.14.13.-
Benzene
1.14.12.3
cis-Dihydro-benzenediol
1.3.1.19
Catechol
Benzoate
1.14.12.10
cis-1,2-Dihydroxy-cyclohexa-3,5-diene-1-carboxylate
1.3.1.25
1.13.11.1
cis,cis-Muconate
5.5.1.1
(+)-Muconolactone
5.3.3.4
3-Oxoadipate-enol-lactone
3.1.1.24
3-Oxoadipate
2.8.3.6
3-Oxoadipyl-CoA
2.3.1.16
2.3.1.174
Succinyl-CoA
Cytrate cycle
1.13.11.2
4.1.1.46
3-Carboxy-2-hydroxy-muconatesemialdehyde
1.14.99.23
2,3-Dihydroxy-benzoate
1.13.11.14
4.1.1.-
2-Hydroxy-muconatesemialdehyde
3.7.1.9
2-Oxopent-4-enoate
4.2.1.80
4-Hydroxy-2-oxopentanoate
4.1.3.39
Acetaldehyde
1.2.1.10
Pyruvate
Glycolysis
1.2.1.85
4.1.1.77
2-Aminobenzene-sulfonate
1.14.12.14
3-Sulfocatechol
1.13.11.2
2-Hydroxy-muconate
5.3.2.6
γ-Oxalocrotonate
4.1.1.-
6.2.1.25
Cyclohexane-1-carboxylate
AliA
Cyclohexane-1-carboxyl-CoA
AliB
2-Hydroxycyclohexane-1-carboxyl-CoA
Cyclohex-2,5-diene-1-carboxyl-CoA
Cyclohex-1-ene-1-carboxyl-CoA
4.2.1.-
1.1.1.-
2-Ketocyclohexane-1-carboxyl-CoA
3.1.2.-
Pimeloyl-CoA
1.3.1.62
2,3-Didehydro-pimeloyl-CoA
4.2.1.-
Benzoyl-CoA
1.3.7.8
Cyclohex-1,5-diene-1-carboxyl-CoA
4.2.1.100
6-Hydroxycyclohex-1-ene-1-carbonyl-CoA
1.1.1.-
6-Ketoxycyclohex-1-ene-1-carboxyl-CoA
3.7.1.-
3-Hydroxy-pimeloyl-CoA
1.1.1.259
3-Keto-pimeloyl-CoA
2.3.1.-
Glutaryl-CoA
1.3.99.32
Glutaconyl-CoA
4.1.1.70
Crotonoyl-CoA
4.2.1.17
(S)-3-Hydroxy-butanoyl-CoA
1.1.1.157
Acetoacetyl-CoA
2.3.1.9
Acetyl-CoA
Cytrate cycle
Cyclohex-1,4-diene-1-carboxyl-CoA
00362 3/6/14
(c) Kanehisa Laboratories

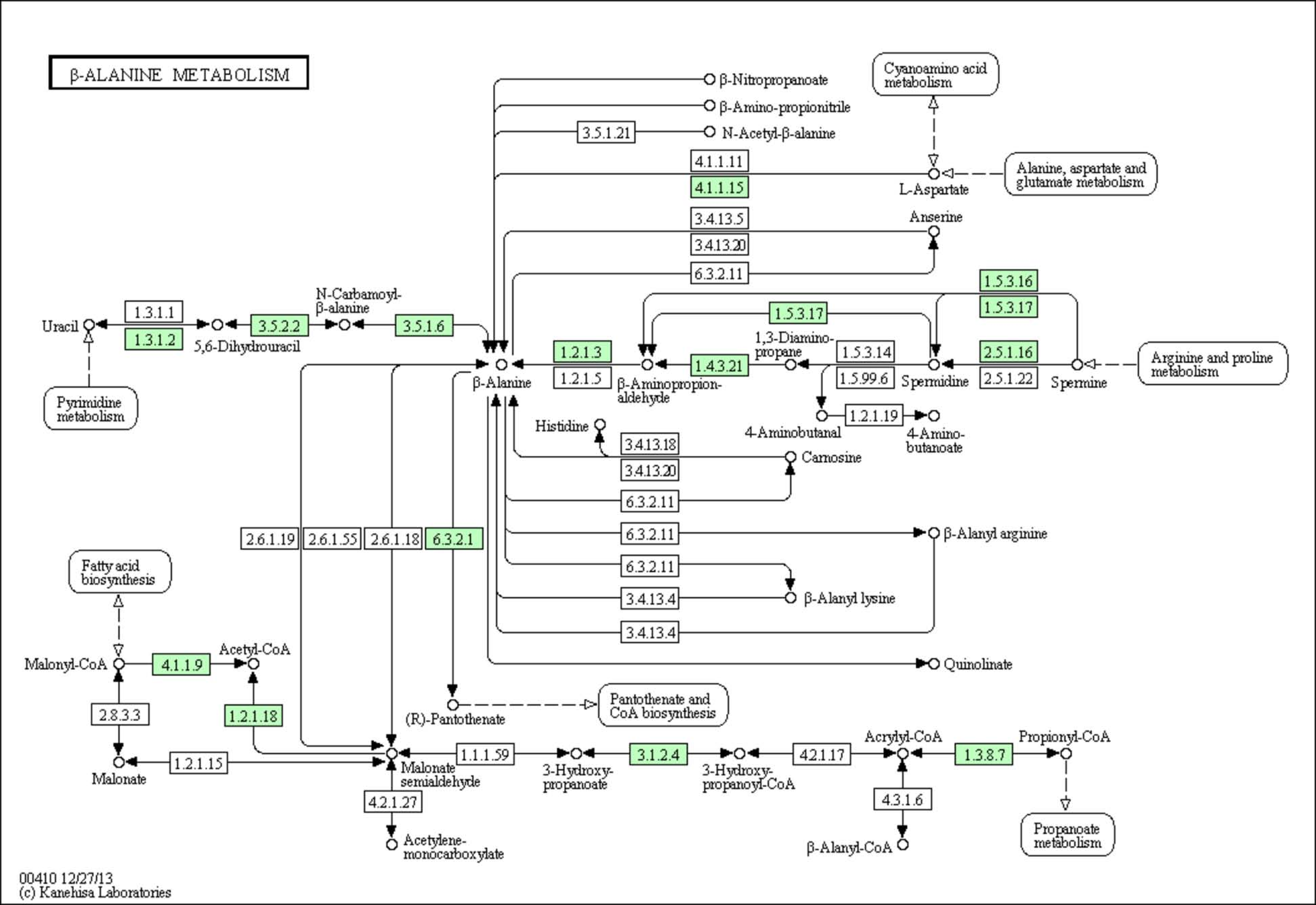
β-ALANINE METABOLISM
β-Nitropropanoate
β-Amino-propionitrile
3.5.1.21
N-Acetyl-β-alanine
Cyanoamino acid metabolism
4.1.1.11
4.1.1.15
L-Aspartate
Alanine, aspartate and glutamate metabolism
3.4.13.5
3.4.13.20
6.3.2.11
Anserine
1.5.3.16
1.5.3.17
1.5.3.17
Uracil
1.3.1.1
1.3.1.2
5,6-Dihydrouracil
3.5.2.2
N-Carbamoyl-β-alanine
3.5.1.6
1.2.1.3
1.2.1.5
β-Alanine
β-Aminopropion-aldehyde
1.4.3.21
1,3-Diamino-propane
1.5.3.14
1.5.99.6
Spermidine
2.5.1.16
2.5.1.22
Spermine
Arginine and proline metabolism
Pyrimidine metabolism
4-Aminobutanal
1.2.1.19
4-Amino-butanoate
Histidine
3.4.13.18
3.4.13.20
Carnosine
6.3.2.11
6.3.2.11
β-Alanyl arginine
6.3.2.11
β-Alanyl lysine
3.4.13.4
3.4.13.4
Quinolinate
2.6.1.19
2.6.1.55
2.6.1.18
6.3.2.1
Fatty acid biosynthesis
Malonyl-CoA
4.1.1.9
Acetyl-CoA
2.8.3.3
1.2.1.18
(R)-Pantothenate
Pantothenate and CoA biosynthesis
Malonate
1.2.1.15
Malonate semialdehyde
1.1.1.59
3-Hydroxy-propanoate
3.1.2.4
3-Hydroxy-propanoyl-CoA
4.2.1.17
Acrylyl-CoA
1.3.8.7
Propionyl-CoA
4.2.1.27
Acetylene-monocarboxylate
4.3.1.6
β-Alanyl-CoA
Propanoate metabolism
00410 12/27/13
(c) Kanehisa Laboratories

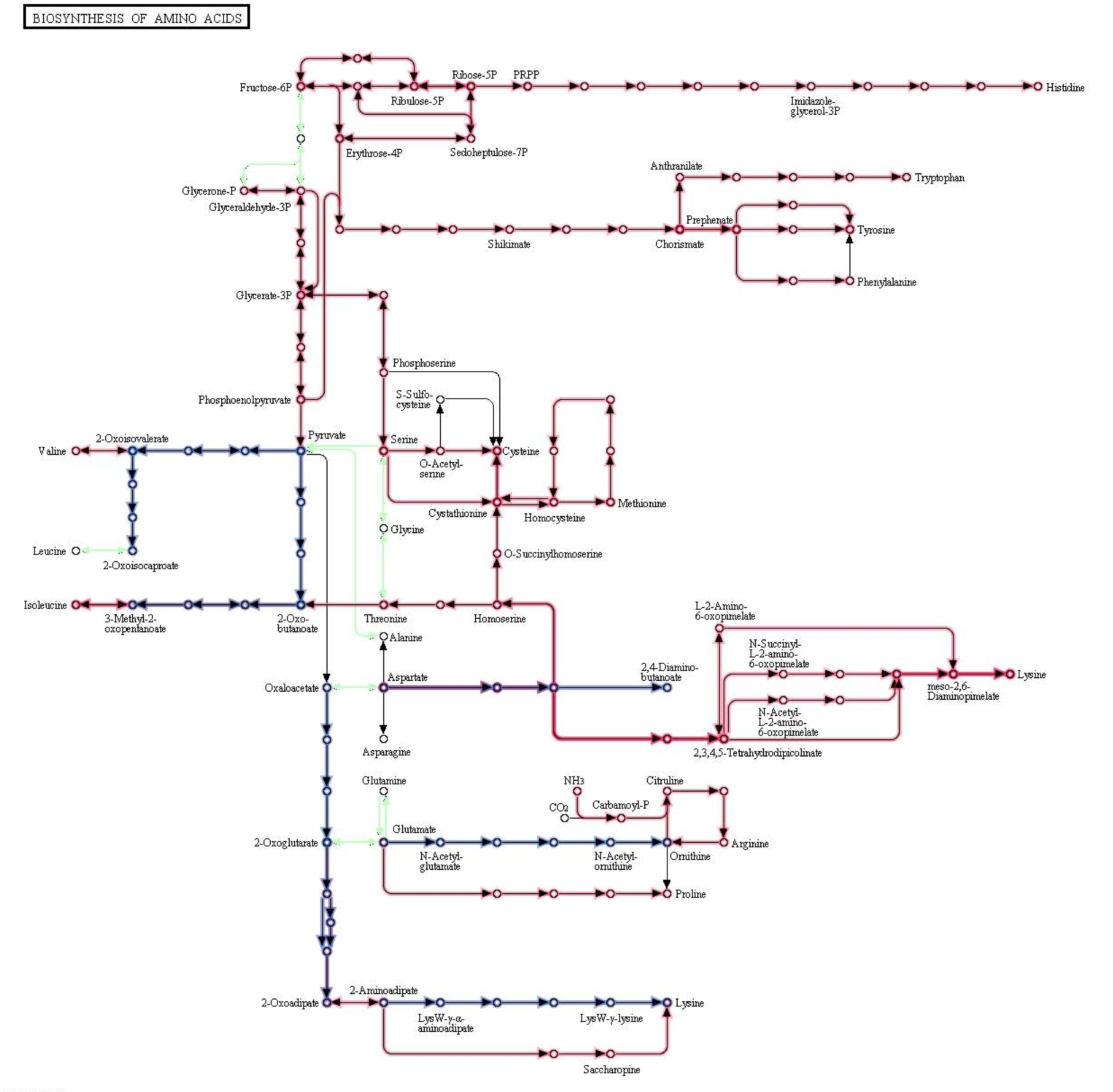

01230 3/13/14
(c) Kanehisa Laboratories

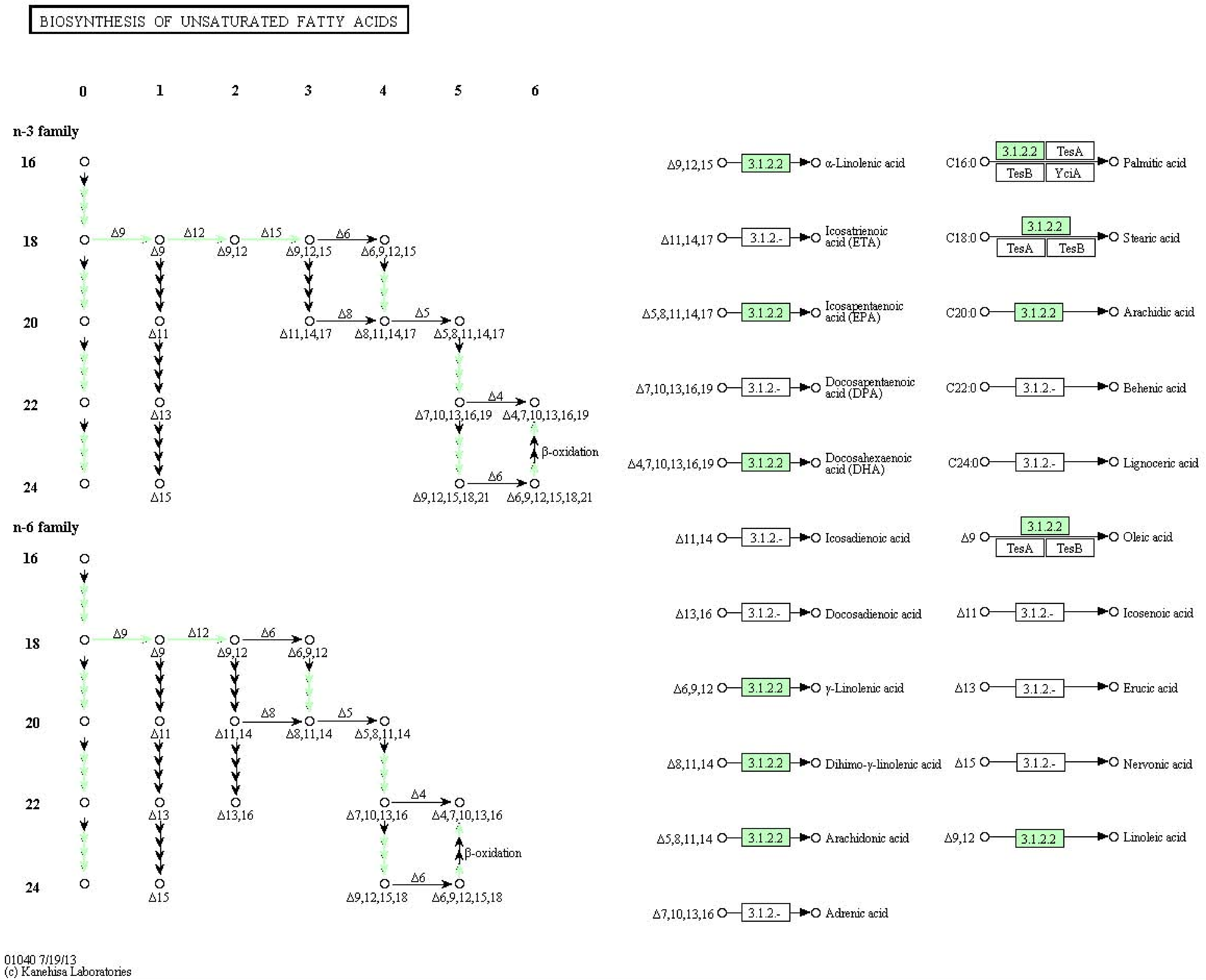
BIOSYNTHESIS OF UNSATURATED FATTY ACIDS
0
1
2
3
4
5
6
n-3 family
16
18
20
22
24
Δ9
Δ12
Δ15
Δ6
Δ9
Δ9,12
Δ9,12,15
Δ6,9,12,15
Δ8
Δ5
Δ11
Δ11,14,17
Δ8,11,14,17
Δ5,8,11,14,17
Δ4
Δ13
Δ7,10,13,16,19
Δ4,7,10,13,16,19
β-oxidation
Δ6
Δ15
Δ9,12,15,18,21
Δ6,9,12,15,18,21
n-6 family
16
18
20
22
24
Δ9
Δ12
Δ6
Δ9
Δ9,12
Δ6,9,12
Δ8
Δ5
Δ11
Δ11,14
Δ8,11,14
Δ5,8,11,14
Δ4
Δ13
Δ13,16
Δ7,10,13,16
Δ4,7,10,13,16
β-oxidation
Δ6
Δ15
Δ9,12,15,18
Δ6,9,12,15,18
Δ9,12,15 3.1.2.2 α-Linolenic acid
Δ11,14,17 3.1.2.- Icosatrienoic acid (ETA)
Δ5,8,11,14,17 3.1.2.2 Icosapentaenoic acid (EPA)
Δ7,10,13,16,19 3.1.2.- Docosapentaenoic acid (DPA)
Δ4,7,10,13,16,19 3.1.2.2 Docosahexaenoic acid (DHA)
Δ11,14 3.1.2.- Icosadienoic acid
Δ13,16 3.1.2.- Docosadienoic acid
Δ6,9,12 3.1.2.2 γ-Linolenic acid
Δ8,11,14 3.1.2.2 Dihimo-γ-linolenic acid
Δ5,8,11,14 3.1.2.2 Arachidonic acid
Δ7,10,13,16 3.1.2.- Adrenic acid
C16:0 3.1.2.2 TesA TesB YciA Palmitic acid
C18:0 3.1.2.2 TesA TesB Stearic acid
C20:0 3.1.2.2 Arachidic acid
C22:0 3.1.2.- Behenic acid
C24:0 3.1.2.- Lignoceric acid
Δ9 3.1.2.2 TesA TesB Oleic acid
Δ11 3.1.2.- Icosenoic acid
Δ13 3.1.2.- Erucic acid
Δ15 3.1.2.- Nervonic acid
Δ9,12 3.1.2.2 Linoleic acid
01040 7/19/13
(c) Kanehisa Laboratories

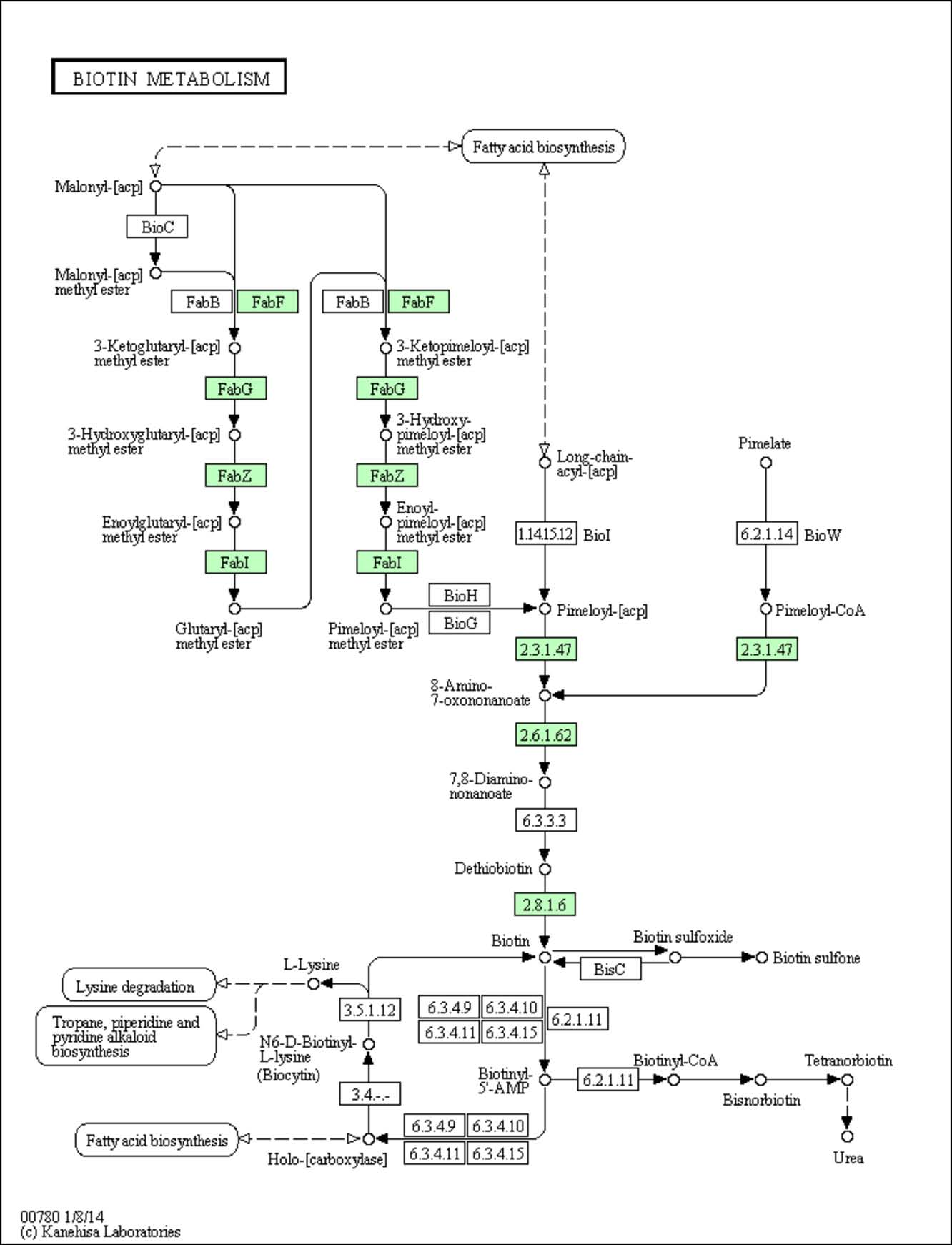
BIOTIN METABOLISM
Fatty acid biosynthesis
Malonyl-[acp]
BioC
Malonyl-[acp] methyl ester
FabB
FabF
FabB
FabF
3-Ketoglutaryl-[acp] methyl ester
3-Ketopimeloyl-[acp] methyl ester
FabG
FabG
3-Hydroxyglutaryl-[acp] methyl ester
3-Hydroxy-pimeloyl-[acp] methyl ester
FabZ
FabZ
Enoylglutaryl-[acp] methyl ester
Enoyl-pimeloyl-[acp] methyl ester
FabI
FabI
Glutaryl-[acp] methyl ester
Pimeloyl-[acp] methyl ester
BioH
BioG
Long-chain-acyl-[acp]
1.14.15.12
BioI
Pimelate
6.2.1.14
BioW
Pimeloyl-[acp]
Pimeloyl-CoA
2.3.1.47
2.3.1.47
8-Amino-7-oxononanoate
2.6.1.62
7,8-Diamino-nonanoate
6.3.3.3
Dethiobiotin
2.8.1.6
Biotin
Biotin sulfoxide
Biotin sulfone
BisC
L-Lysine
Lysine degradation
Tropane, piperidine and pyridine alkaloid biosynthesis
3.5.1.12
6.3.4.9
6.3.4.10
6.3.4.11
6.3.4.15
6.2.1.11
N6-D-Biotinyl-L-lysine (Biocytin)
3.4.-.-
Biotinyl-5'-AMP
6.2.1.11
Biotinyl-CoA
Bisnorbiotin
Tetranorbiotin
Urea
Fatty acid biosynthesis
6.3.4.9
6.3.4.10
6.3.4.11
6.3.4.15
Holo-[carboxylase]
00780 1/8/14
(c) Kanehisa Laboratories

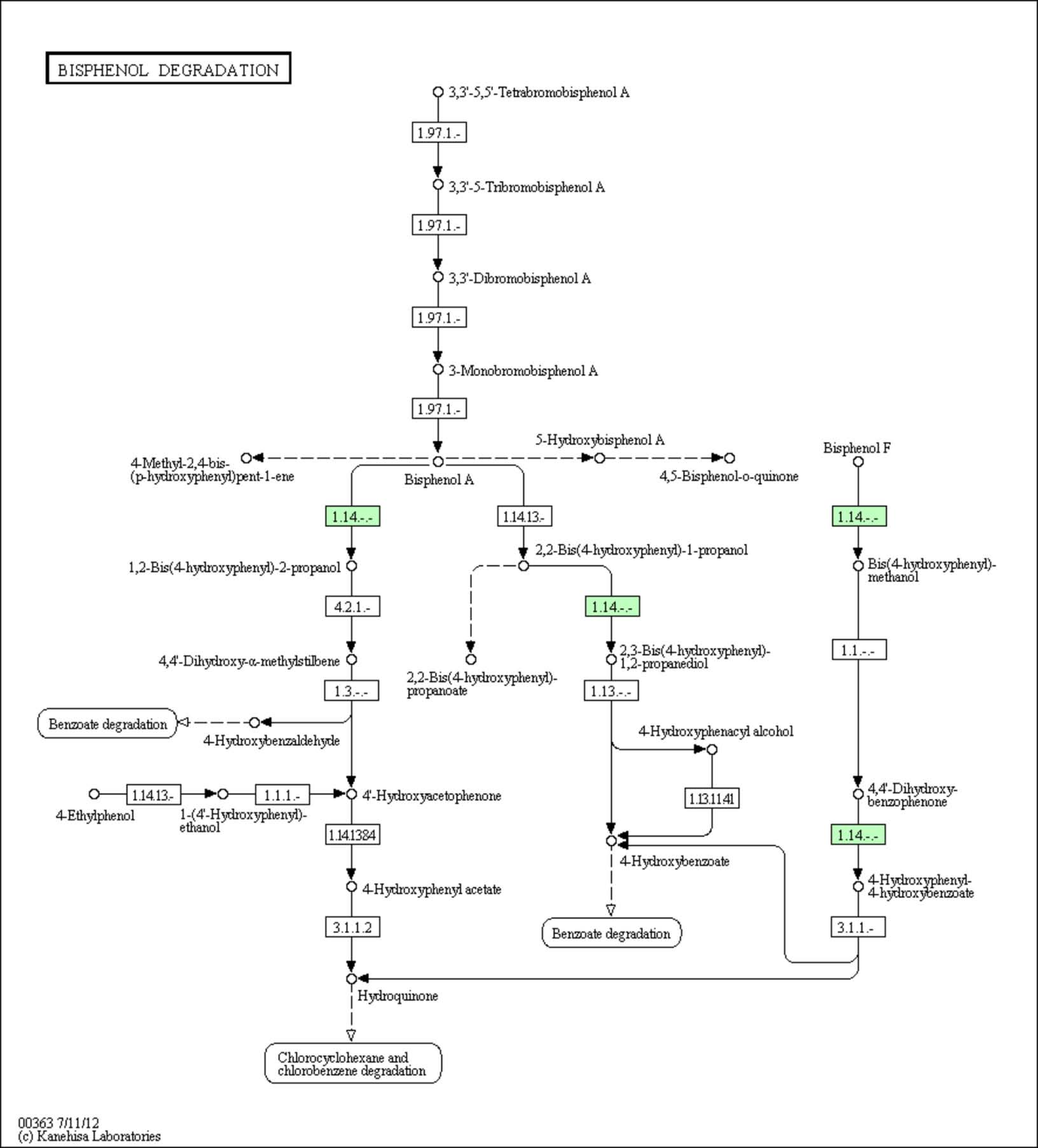
BISPHENOL DEGRADATION
3,3'-5,5'-Tetrabromobisphenol A
1.97.1.-
3,3'-5-Tribromobisphenol A
1.97.1.-
3,3'-Dibromobisphenol A
1.97.1.-
3-Monobromobisphenol A
1.97.1.-
4-Methyl-2,4-bis-(p-hydroxyphenyl)pent-1-ene
Bisphenol A
5-Hydroxybisphenol A
4,5-Bisphenol-o-quinone
Bisphenol F
1.14.-.-
1.14.13.-
1.14.-.-
1,2-Bis(4-hydroxyphenyl)-2-propanol
2,2-Bis(4-hydroxyphenyl)-1-propanol
Bis(4-hydroxyphenyl)-methanol
4.2.1.-
1.14.-.-
1.1.-.-
4,4'-Dihydroxy-α-methylstilbene
2,2-Bis(4-hydroxyphenyl)-propanoate
2,3-Bis(4-hydroxyphenyl)-1,2-propanediol
1.3.-.-
1.13.-.-
Benzoate degradation
4-Hydroxybenzaldehyde
4-Hydroxyphenacyl alcohol
4-Ethylphenol
1.14.13.-
1-(4'-Hydroxyphenyl)-ethanol
1.1.1.-
4'-Hydroxyacetophenone
1.13.11.41
4,4'-Dihydroxy-benzophenone
1.14.13.84
1.14.-.-
4-Hydroxybenzoate
4-Hydroxyphenyl acetate
4-Hydroxyphenyl-4-hydroxybenzoate
3.1.1.2
Benzoate degradation
3.1.1.-
Hydroquinone
Chlorocyclohexane and chlorobenzene degradation
00363 7/11/12
(c) Kanehisa Laboratories

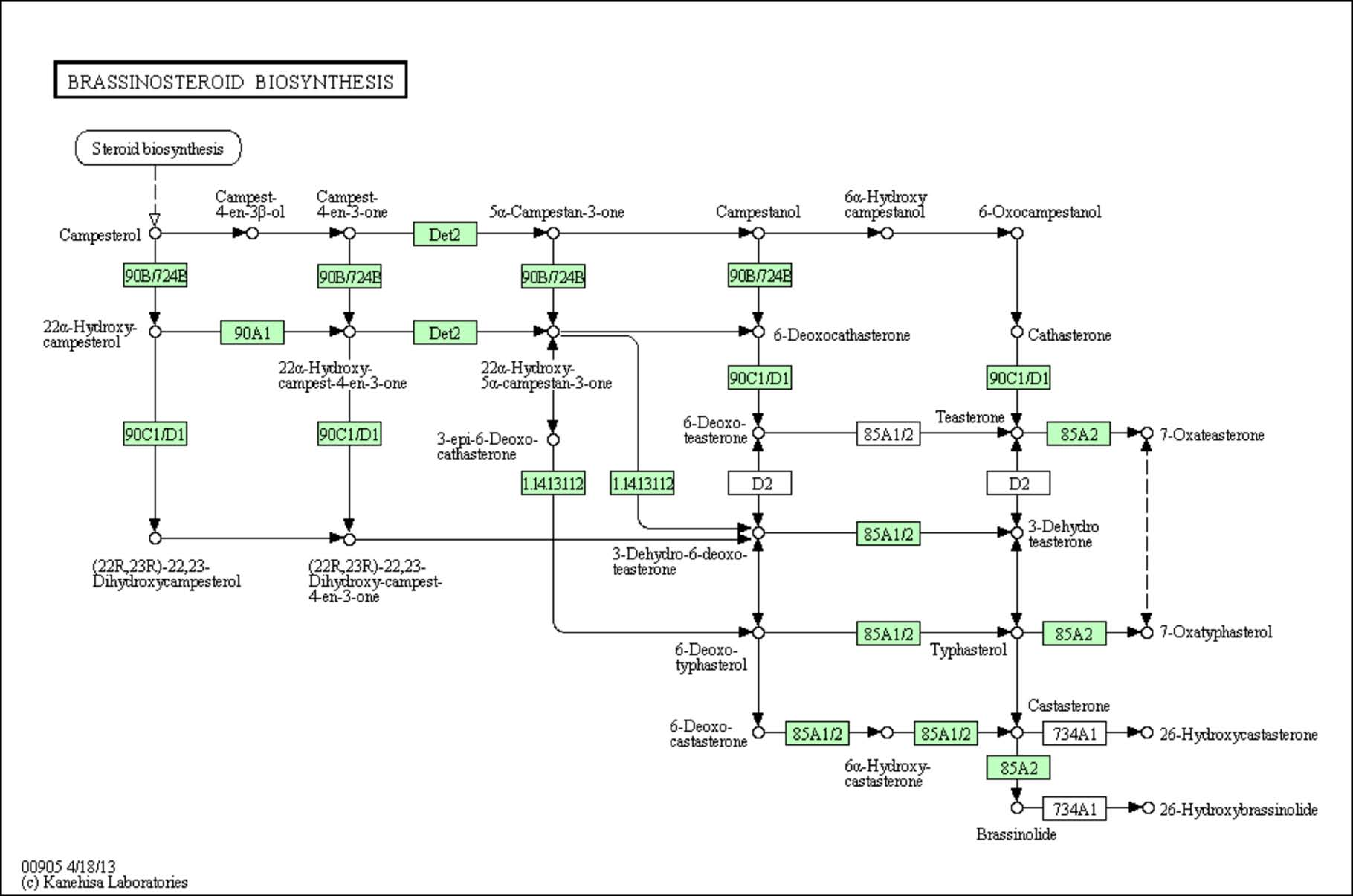
BRASSINOSTEROID BIOSYNTHESIS
Steroid biosynthesis
Campesterol
Campest-4-en-3β-ol
Campest-4-en-3-one
Det2
5α-Campestan-3-one
Campestanol
6α-Hydroxy campestanol
6-Oxocampestanol
90B/724B
90B/724B
90B/724B
90B/724B
22α-Hydroxy-campesterol
90A1
Det2
6-Deoxocathasterone
Cathasterone
22α-Hydroxy-campest-4-en-3-one
22α-Hydroxy-5α-campestan-3-one
90C1/D1
90C1/D1
90C1/D1
90C1/D1
3-epi-6-Deoxo-cathasterone
6-Deoxo-teasterone
85A1/2
Teasterone
85A2
7-Oxateasterone
1.14.13112
1.14.13112
D2
D2
3-Dehydro teasterone
85A1/2
3-Dehydro-6-deoxo-teasterone
(22R,23R)-22,23-Dihydroxycampesterol
(22R,23R)-22,23-Dihydroxy-campest-4-en-3-one
6-Deoxo-typhasterol
85A1/2
Typhasterol
85A2
7-Oxatyphasterol
Castasterone
6-Deoxo-castasterone
85A1/2
85A1/2
734A1
26-Hydroxycastasterone
6α-Hydroxy-castasterone
85A2
734A1
26-Hydroxybrassinolide
Brassinolide
00905 4/18/13
(c) Kanehisa Laboratories

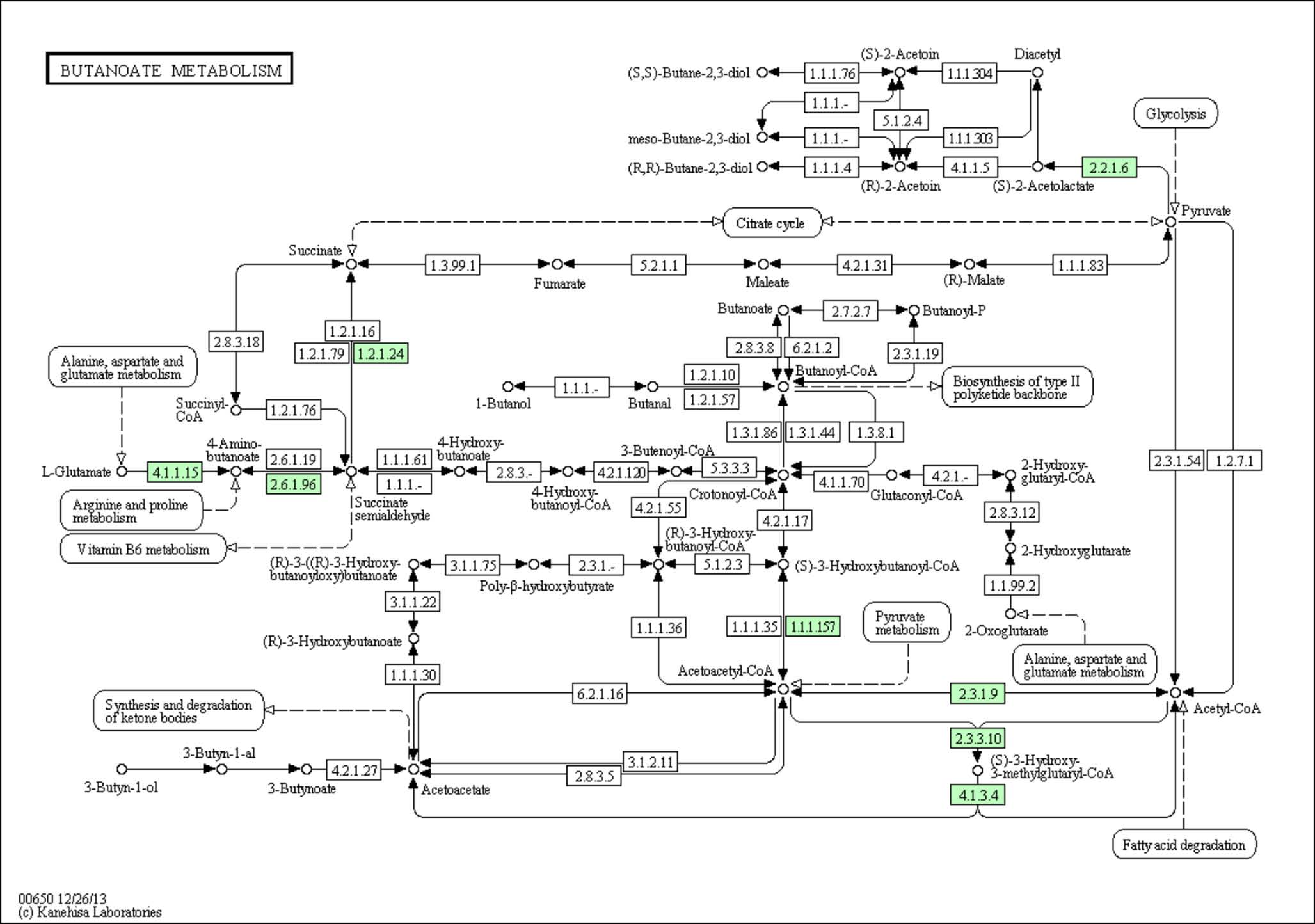

BUTANOATE METABOLISM
(S)-2-Acetoin
Diacetyl
(S,S)-Butane-2,3-diol
1.1.1.76
1.1.1.304
1.1.1.-
5.1.2.4
meso-Butane-2,3-diol
1.1.1.-
1.1.1.303
Glycolysis
(R,R)-Butane-2,3-diol
1.1.1.4
4.1.1.5
2.2.1.6
(R)-2-Acetoin
(S)-2-Acetolactate
Pyruvate
Citrate cycle
Succinate
1.3.99.1
Fumarate
5.2.1.1
Maleate
4.2.1.31
(R)-Malate
1.1.1.83
Butanoate
2.7.2.7
Butanoyl-P
1.2.1.16
2.8.3.18
1.2.1.79
1.2.1.24
2.8.3.8
6.2.1.2
2.3.1.19
Alanine, aspartate and glutamate metabolism
Butanoyl-CoA
1.2.1.10
1.1.1.-
Biosynthesis of type II polyketide backbone
1-Butanol
Butanal
1.2.1.57
Succinyl-CoA
1.2.1.76
1.3.1.86
1.3.1.44
1.3.8.1
4-Amino-butanoate
4-Hydroxy-butanoate
3-Butenoyl-CoA
L-Glutamate
4.1.1.15
2.6.1.19
1.1.1.61
2.8.3.-
4.2.1.120
5.3.3.3
2-Hydroxy-glutaryl-CoA
4.2.1.-
2.3.1.54
1.2.7.1
2.6.1.96
1.1.1.-
4-Hydroxy-butanoyl-CoA
4.1.1.70
Glutaconyl-CoA
Arginine and proline metabolism
Succinate semialdehyde
Crotonoyl-CoA
4.2.1.55
4.2.1.17
2.8.3.12
Vitamin B6 metabolism
(R)-3-Hydroxy-butanoyl-CoA
2-Hydroxyglutarate
(R)-3-((R)-3-Hydroxy-butanoyloxy)butanoate
3.1.1.75
2.3.1.-
5.1.2.3
(S)-3-Hydroxybutanoyl-CoA
Poly-β-hydroxybutyrate
1.1.99.2
3.1.1.22
1.1.1.36
1.1.1.35
1.1.1.157
Pyruvate metabolism
2-Oxoglutarate
(R)-3-Hydroxybutanoate
Alanine, aspartate and glutamate metabolism
1.1.1.30
Acetoacetyl-CoA
Synthesis and degradation of ketone bodies
6.2.1.16
2.3.1.9
Acetyl-CoA
2.3.3.10
3-Butyn-1-al
3.1.2.11
(S)-3-Hydroxy-3-methylglutaryl-CoA
3-Butyn-1-ol
3-Butynoate
4.2.1.27
2.8.3.5
Acetoacetate
4.1.3.4
Fatty acid degradation
00650 12/26/13
(c) Kanehisa Laboratories

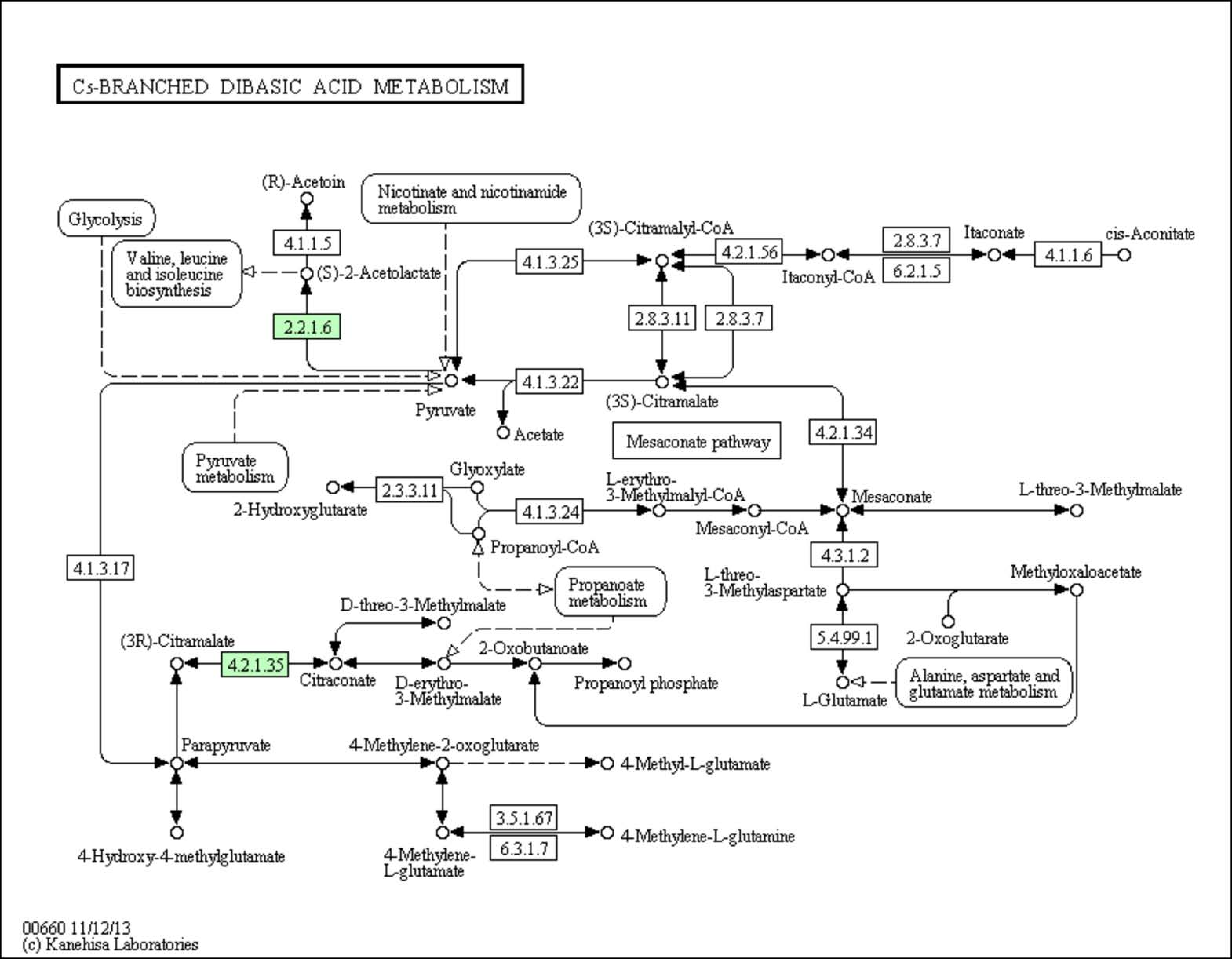
C5-BRANCHED DIBASIC ACID METABOLISM
(R)-Acetoin
Nicotinate and nicotinamide metabolism
Glycolysis
4.1.1.5
Valine, leucine and isoleucine biosynthesis
(S)-2-Acetolactate
2.2.1.6
(3S)-Citramalyl-CoA
4.1.3.25
4.2.1.56
2.8.3.7
6.2.1.5
Itaconyl-CoA
Itaconate
4.1.1.6
cis-Aconitate
2.8.3.11
2.8.3.7
Pyruvate
4.1.3.22
(3S)-Citramalate
Acetate
Mesaconate pathway
4.2.1.34
Pyruvate metabolism
Glyoxylate
2.3.3.11
2-Hydroxyglutarate
4.1.3.24
L-erythro-3-Methylmalyl-CoA
Mesaconyl-CoA
Mesaconate
L-threo-3-Methylmalate
Propanoyl-CoA
4.1.3.17
4.3.1.2
L-threo-3-Methylaspartate
Methyloxaloacetate
Propanoate metabolism
D-threo-3-Methylmalate
(3R)-Citramalate
4.2.1.35
Citraconate
D-erythro-3-Methylmalate
2-Oxobutanoate
Propanoyl phosphate
5.4.99.1
2-Oxoglutarate
L-Glutamate
Alanine, aspartate and glutamate metabolism
Parapyruvate
4-Methylene-2-oxoglutarate
4-Methyl-L-glutamate
3.5.1.67
6.3.1.7
4-Methylene-L-glutamine
4-Hydroxy-4-methylglutamate
4-Methylene-L-glutamate
00660 11/12/13
(c) Kanehisa Laboratories

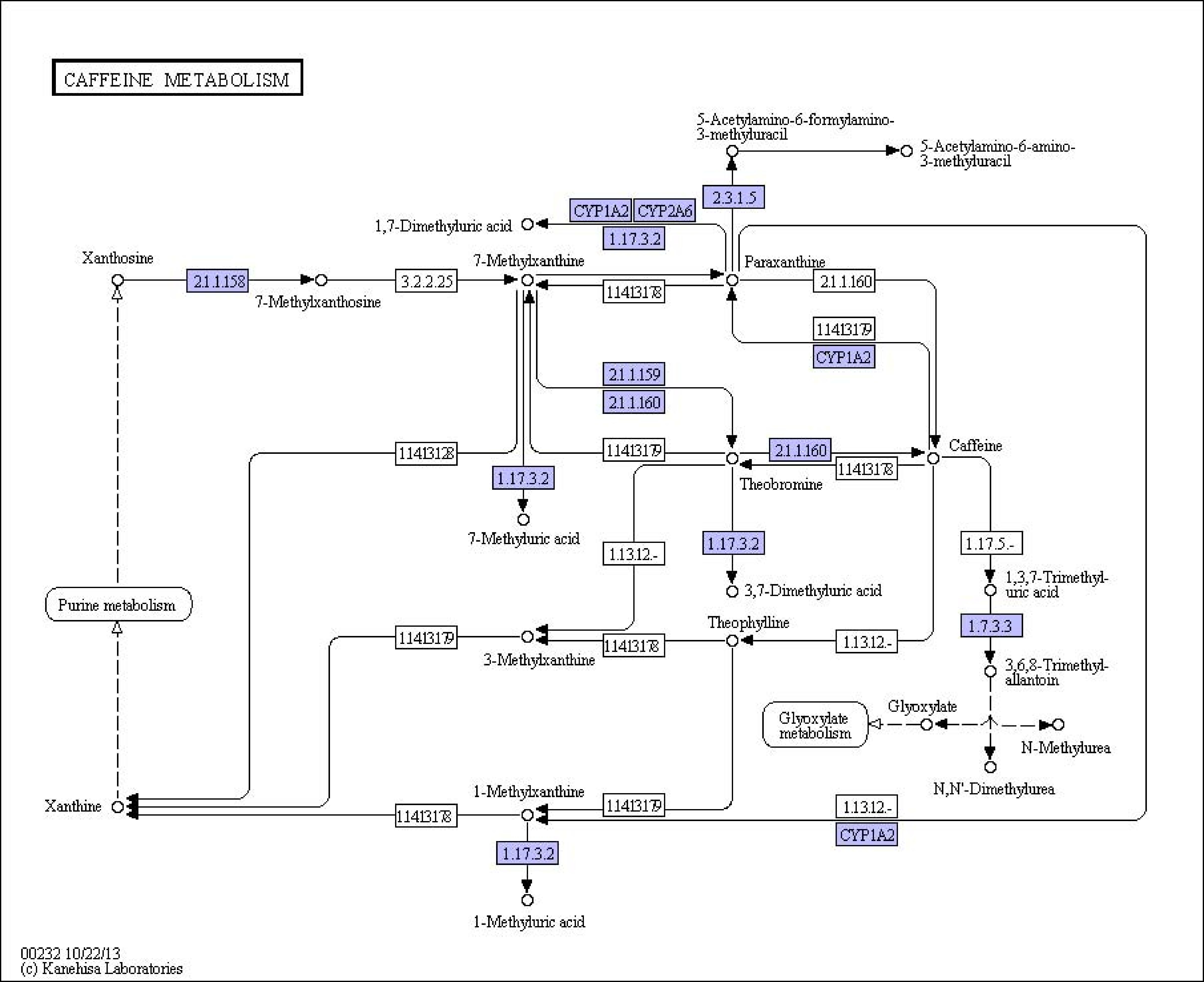
CAFFEINE METABOLISM
5-Acetylamino-6-formylamino-3-methyluracil
5-Acetylamino-6-amino-3-methyluracil
2.3.1.5
CYP1A2
CYP2A6
1.17.3.2
1,7-Dimethyluric acid
Xanthosine
2.1.1.158
7-Methylxanthosine
3.2.2.25
7-Methylxanthine
1.14.13.178
Paraxanthine
2.1.1.160
1.14.13.179
CYP1A2
2.1.1.159
2.1.1.160
1.14.13.128
1.14.13.179
2.1.1.160
Caffeine
1.17.3.2
1.14.13.178
Theobromine
7-Methyluric acid
1.13.12.-
1.17.3.2
1.17.5.-
3,7-Dimethyluric acid
1,3,7-Trimethyluric acid
Purine metabolism
Theophylline
1.7.3.3
1.14.13.179
3-Methylxanthine
1.14.13.178
1.13.12.-
3,6,8-Trimethylallantoin
Glyoxylate
Glyoxylate metabolism
N-Methylurea
N,N'-Dimethylurea
Xanthine
1-Methylxanthine
1.14.13.179
1.14.13.178
1.13.12.-
CYP1A2
1.17.3.2
1-Methyluric acid
00232 10/22/13
(c) Kanehisa Laboratories

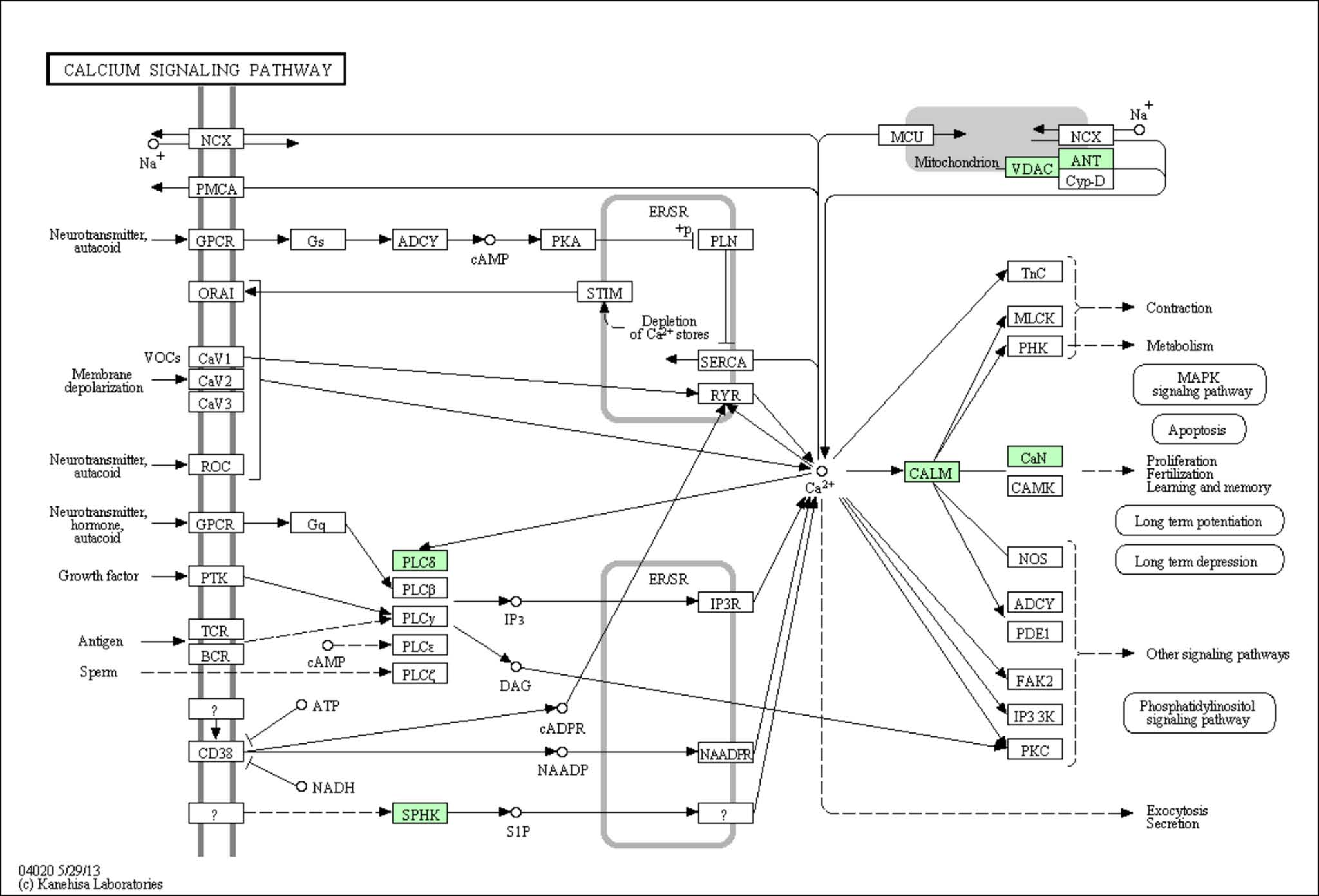

CALCIUM SIGNALING PATHWAY
NCX
Na+
PMCA
Neurotransmitter, autacoid
GPCR
Gs
ADCY
cAMP
PKA
ER/SR
+p
PLN
ORAI
STIM
Depletion of Ca2+ stores
VOCs
CaV1
Membrane depolarization
CaV2
CaV3
SERCA
RYR
Neurotransmitter, autacoid
ROC
Neurotransmitter, hormone, autacoid
GPCR
Gq
PLCδ
PLCβ
Growth factor
PTK
PLCγ
IP3
IP3R
Antigen
TCR
BCR
cAMP
PLCε
Sperm
PLCζ
DAG
?
ATP
cADPR
CD38
NAADP
NAADPR
NADH
?
SPHK
S1P
?
MCU
NCX
Na+
Mitochondrion
VDAC
ANT
Cyp-D
TnC
MLCK
PHK
Contraction
Metabolism
MAPK signalng pathway
Apoptosis
Ca2+
CALM
CaN
CAMK
Proliferation
Fertilization
Learning and memory
Long term potentiation
Long term depression
NOS
ADCY
PDE1
Other signaling pathways
FAK2
IP3 3K
PKC
Phosphatidylinositol signaling pathway
Exocytosis
Secretion
04020 5/29/13
(c) Kanehisa Laboratories

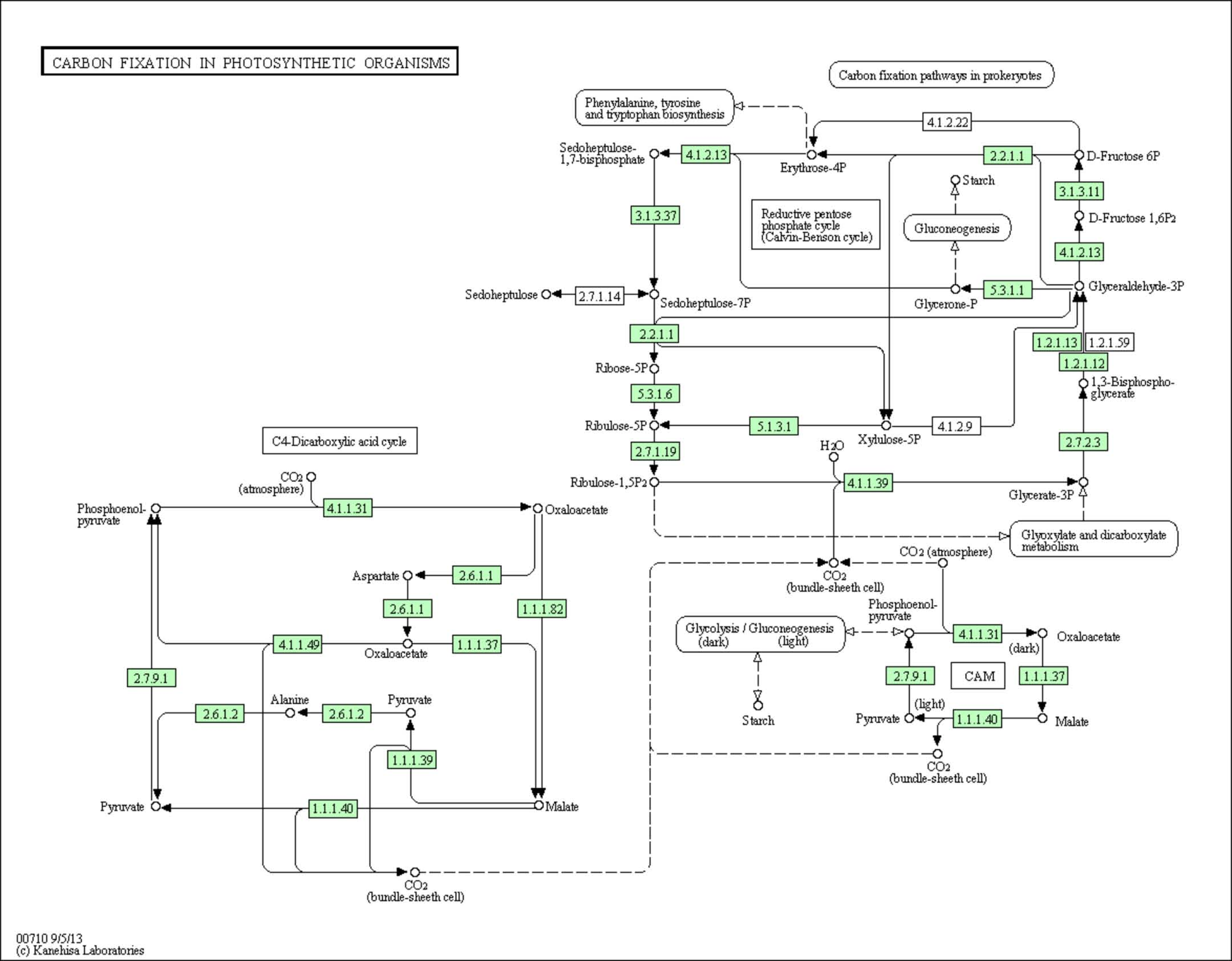
CARBON FIXATION IN PHOTOSYNTHETIC ORGANISMS
Carbon fixation pathways in prokaryotes
Phenylalanine, tyrosine and tryptophan biosynthesis
4.1.2.22
Sedoheptulose-1,7-bisphosphate
4.1.2.13
Erythrose-4P
2.2.1.1
D-Fructose 6P
3.1.3.11
Starch
3.1.3.37
Reductive pentose phosphate cycle (Calvin-Benson cycle)
Gluconeogenesis
D-Fructose 1,6P2
4.1.2.13
Sedoheptulose
2.7.1.14
Sedoheptulose-7P
5.3.1.1
Glycerone-P
Glyceraldehyde-3P
2.2.1.1
1.2.1.13
1.2.1.59
1.2.1.12
Ribose-5P
1,3-Bisphospho-glycerate
5.3.1.6
Ribulose-5P
5.1.3.1
Xylulose-5P
4.1.2.9
2.7.2.3
2.7.1.19
H_2O
Ribulose-1,5P2
4.1.1.39
Glycerate-3P
C4-Dicarboxylic acid cycle
CO_2 (atmosphere)
Phosphoenol-pyruvate
4.1.1.31
Oxaloacetate
Glyoxylate and dicarboxylate metabolism
CO_2 (atmosphere)
CO_2 (bundle-sheeth cell)
Aspartate
2.6.1.1
2.6.1.1
1.1.1.82
Phosphoenol-pyruvate
Glycolysis / Gluconeogenesis (dark) (light)
4.1.1.31
Oxaloacetate
(dark)
4.1.1.49
Oxaloacetate
1.1.1.37
2.7.9.1
CAM
2.7.9.1
1.1.1.37
Starch
(light)
Pyruvate
1.1.1.40
Malate
Alanine
Pyruvate
2.6.1.2
2.6.1.2
1.1.1.39
CO_2 (bundle-sheeth cell)
Pyruvate
1.1.1.40
Malate
CO_2 (bundle-sheeth cell)
00710 9/5/13
(c) Kanehisa Laboratories

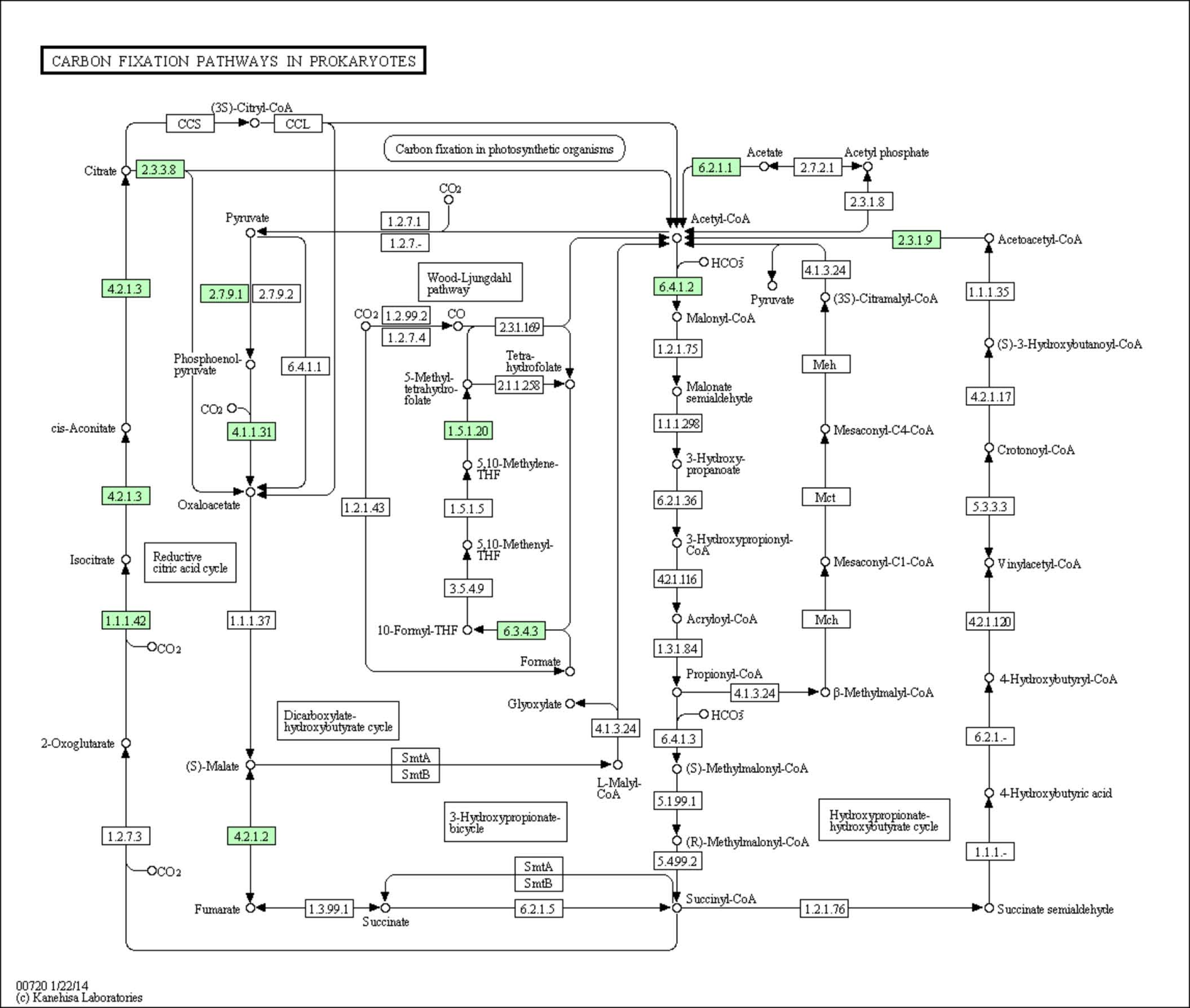
CARBON FIXATION PATHWAYS IN PROKARYOTES
(3S)-Citryl-CoA
CCS
CCL
Carbon fixation in photosynthetic organisms
Citrate
2.3.3.8
Acetate
Acetyl phosphate
6.2.1.1
2.7.2.1
2.3.1.8
CO2
Pyruvate
1.2.7.1
1.2.7.-
Acetyl-CoA
2.3.1.9
Acetoacetyl-CoA
HCO3-
6.4.1.2
4.1.3.24
Pyruvate
(3S)-Citramalyl-CoA
1.1.1.35
(S)-3-Hydroxybutanoyl-CoA
4.2.1.3
2.7.9.1
2.7.9.2
Wood-Ljungdahl pathway
CO2
1.2.99.2
1.2.7.4
CO
2.3.1.169
Malonyl-CoA
1.2.1.75
Meh
Phosphoenol-pyruvate
6.4.1.1
5-Methyl-tetrahydro-folate
Tetra-hydrofolate
2.1.1.258
Malonate semialdehyde
4.2.1.17
CO2
4.1.1.31
1.5.1.20
1.1.1.298
cis-Aconitate
Mesaconyl-C4-CoA
Crotonoyl-CoA
5,10-Methylene-THF
3-Hydroxy-propanoate
Oxaloacetate
4.2.1.3
1.2.1.43
1.5.1.5
Mct
6.2.1.36
5.3.3.3
Isocitrate
Reductive citric acid cycle
5,10-Methenyl-THF
3-Hydroxypropionyl-CoA
Mesaconyl-C1-CoA
Vinylacetyl-CoA
3.5.4.9
4.2.1.116
1.1.1.42
1.1.1.37
10-Formyl-THF
6.3.4.3
Acryloyl-CoA
Mch
4.2.1.120
CO2
1.3.1.84
Formate
Propionyl-CoA
4.1.3.24
β-Methylmalyl-CoA
4-Hydroxybutyryl-CoA
Glyoxylate
4.1.3.24
HCO3-
Dicarboxylate-hydroxybutyrate cycle
6.4.1.3
6.2.1.-
2-Oxoglutarate
(S)-Malate
SmtA
SmtB
L-Malyl-CoA
(S)-Methylmalonyl-CoA
4-Hydroxybutyric acid
5.1.99.1
3-Hydroxypropionate-bicycle
Hydroxypropionate-hydroxybutyrate cycle
1.2.7.3
4.2.1.2
(R)-Methylmalonyl-CoA
1.1.1.-
CO2
5.4.99.2
SmtA
SmtB
Fumarate
1.3.99.1
Succinate
6.2.1.5
Succinyl-CoA
1.2.1.76
Succinate semialdehyde
00720 1/22/14
(c) Kanehisa Laboratories

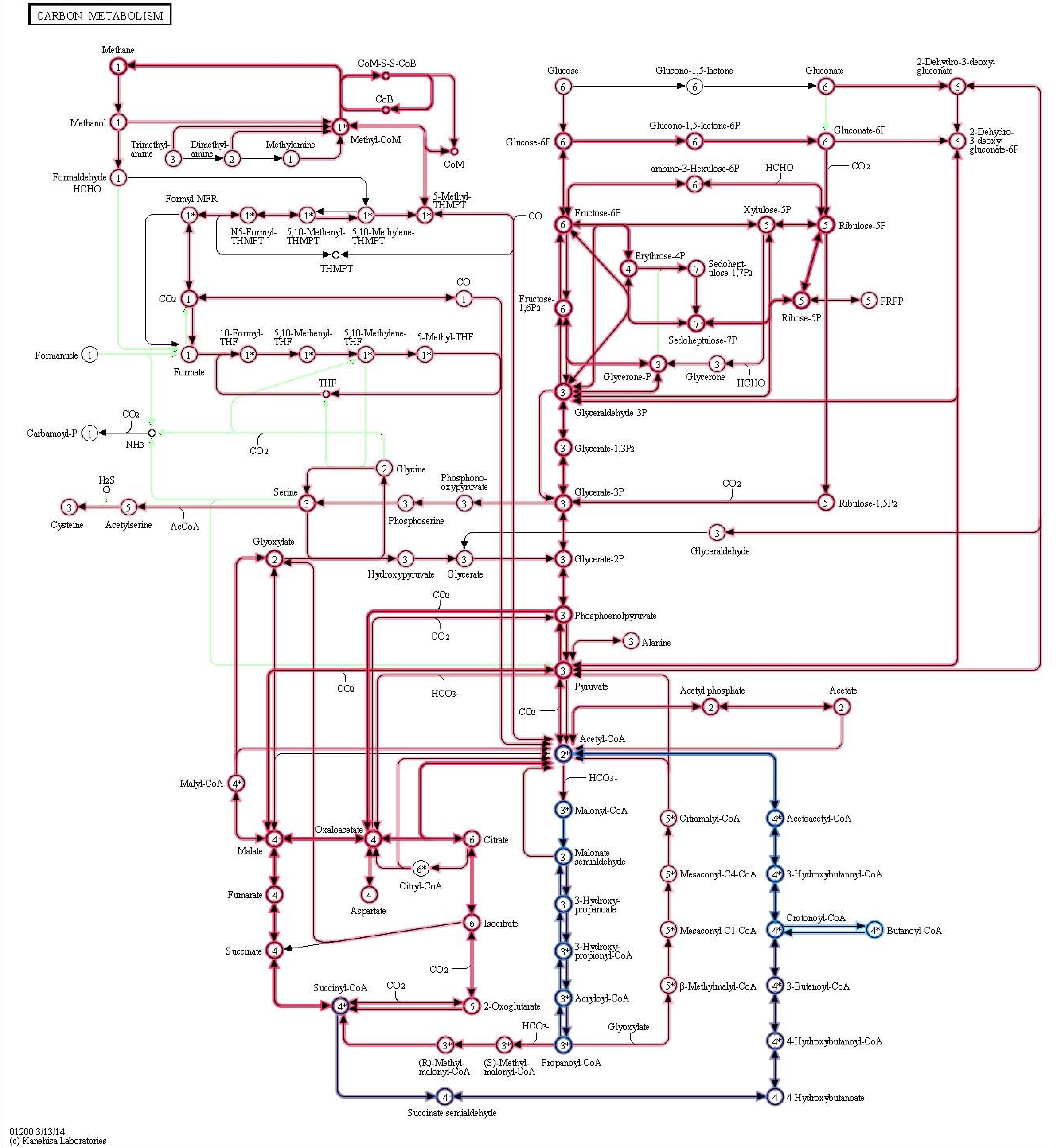
CARBON METABOLISM
Methane
CoM-S-S-CoB
CoB
Methanol
Methyl-CoM
Trimethyl-amine
Dimethyl-amine
Methylamine
CoM
Formaldehyde
HCHO
5-Methyl-THMPT
Formyl-MFR
N5-Formyl-THMPT
5,10-Methenyl-THMPT
5,10-Methylene-THMPT
CO
THMPT
CO_2
Formamide
Formate
10-Formyl-THF
5,10-Methenyl-THF
5,10-Methylene-THF
5-Methyl-THF
THF
Carbamoyl-P
NH_3
H_2S
Cysteine
Acetylserine
AcCoA
Serine
Glycine
Phosphono-oxypyruvate
Phosphoserine
Glyoxylate
Hydroxypyruvate
Glycerate
Glucose
Glucono-1,5-lactone
Gluconate
2-Dehydro-3-deoxy-gluconate
Glucose-6P
Glucono-1,5-lactone-6P
Gluconate-6P
2-Dehydro-3-deoxy-gluconate-6P
arabino-3-Hexulose-6P
HCHO
Fructose-6P
Xylulose-5P
Ribulose-5P
Erythrose-4P
Sedohept-ulose-1,7P_2
Fructose-1,6P_2
Ribose-5P
PRPP
Sedoheptulose-7P
Glycerone-P
Glycerone
Glyceraldehyde-3P
Glycerate-1,3P_2
Glycerate-3P
Ribulose-1,5P_2
Glyceraldehyde
Glycerate-2P
Phosphoenolpyruvate
Alanine
Pyruvate
HCO_3-
Acetyl phosphate
Acetate
Acetyl-CoA
Malyl-CoA
Malonyl-CoA
Citramalyl-CoA
Acetoacetyl-CoA
Malate
Oxaloacetate
Citrate
Malonate semialdehyde
Citryl-CoA
Aspartate
Mesaconyl-C4-CoA
3-Hydroxybutanoyl-CoA
Fumarate
3-Hydroxy-propanoate
Isocitrate
Mesaconyl-C1-CoA
Crotonoyl-CoA
Butanoyl-CoA
Succinate
3-Hydroxy-propionyl-CoA
Succinyl-CoA
2-Oxoglutarate
Acryloyl-CoA
β-Methylmalyl-CoA
3-Butenoyl-CoA
4-Hydroxybutanoyl-CoA
(R)-Methyl-malonyl-CoA
(S)-Methyl-malonyl-CoA
Propanoyl-CoA
Succinate semialdehyde
4-Hydroxybutanoate
01200 3/13/14
(c) Kanehisa Laboratories

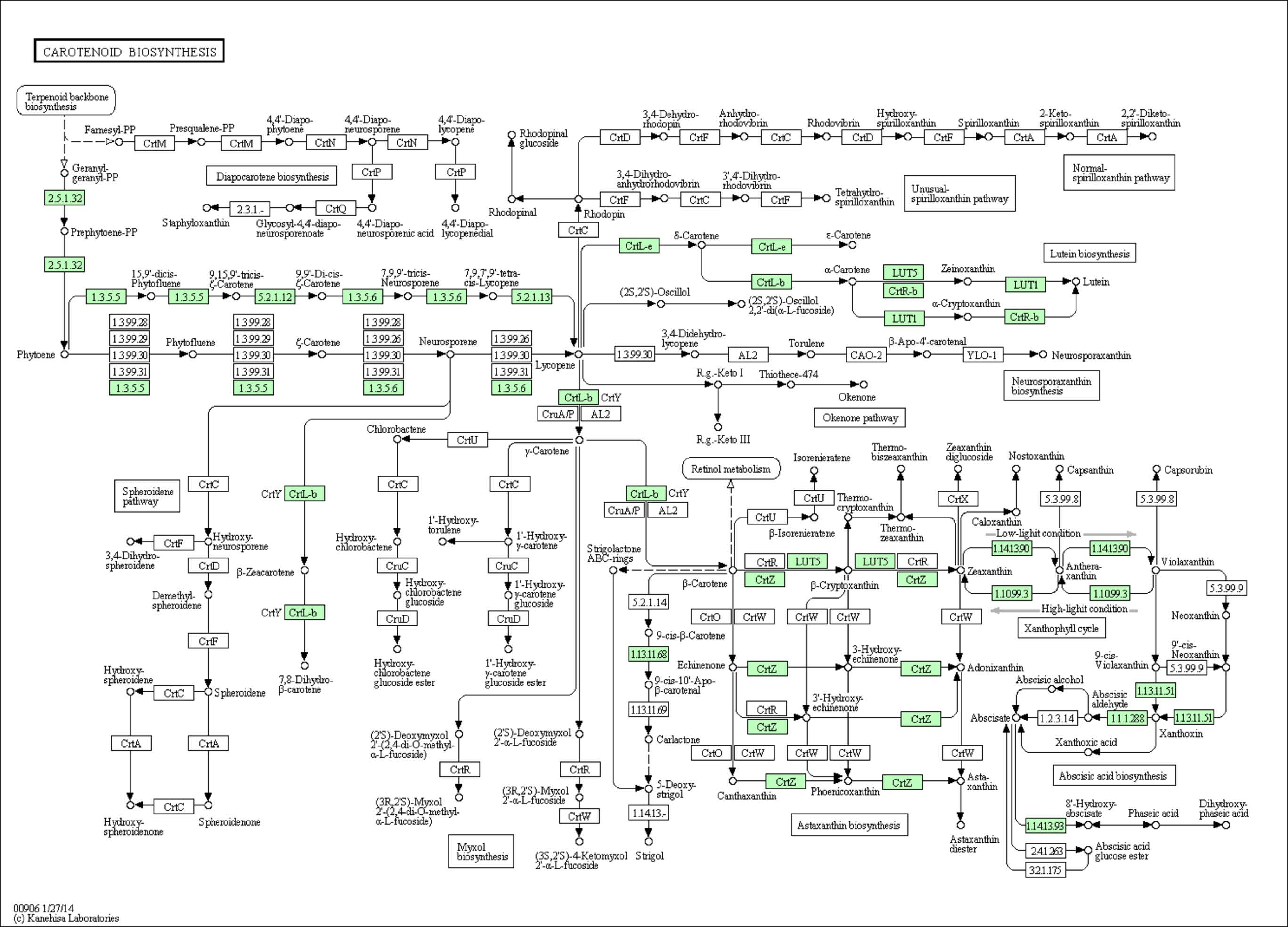

CAROTENOID BIOSYNTHESIS
Terpenoid backbone biosynthesis
Farnesyl-PP
Presqualene-PP
CrtM
CrtM
4,4'-Diapophytoene
CrtN
4,4'-Diaponeurosporene
CrtN
4,4'-Diapolycopene
CrtP
CrtP
Geranylgeranyl-PP
2.5.1.32
Diapocarotene biosynthesis
2.3.1.-
CrtQ
Staphyloxanthin
Glycosyl-4,4'-diaponeurosporenoate
4,4'-Diaponeurosporenic acid
4,4'-Diapolycopenedial
Prephytoene-PP
2.5.1.32
15,9'-dicis-Phytofluene
9,15,9'-tricis-ζ-Carotene
9,9'-Di-cis-ζ-Carotene
7,9,9'-tricis-Neurosporene
7,9,7',9'-tetra-cis-Lycopene
1.3.5.5
1.3.5.5
5.2.1.12
1.3.5.6
1.3.5.6
5.2.1.13
Phytoene
1.3.99.28
1.3.99.29
1.3.99.30
1.3.99.31
1.3.5.5
Phytofluene
ζ-Carotene
1.3.99.26
1.3.5.6
Neurosporene
Lycopene
Rhodopinal glucoside
Rhodopinal
Rhodopin
CrtC
CrtD
3,4-Dehydrorhodopin
CrtF
Anhydrorhodovibrin
CrtC
Rhodovibrin
CrtD
Hydroxyspirilloxanthin
CrtF
Spirilloxanthin
CrtA
2-Keto-spirilloxanthin
CrtA
2,2'-Diketo-spirilloxanthin
Normal-spirilloxanthin pathway
3,4-Dihydroanhydrorhodovibrin
3',4'-Dihydrorhodovibrin
Tetrahydrospirilloxanthin
Unusual-spirilloxanthin pathway
CrtL-e
δ-Carotene
CrtL-e
ε-Carotene
Lutein biosynthesis
α-Carotene
CrtL-b
LUT5
CrtR-b
Zeinoxanthin
LUT1
Lutein
LUT1
α-Cryptoxanthin
CrtR-b
(2S,2'S)-Oscillol
(2S,2'S)-Oscillol 2,2'-di(α-L-fucoside)
3,4-Didehydrolycopene
1.3.99.30
AL2
Torulene
CAO-2
β-Apo-4'-carotenal
YLO-1
Neurosporaxanthin
Neurosporaxanthin biosynthesis
R.g.-Keto I
Thiothece-474
Okenone
Okenone pathway
R.g.-Keto III
CrtL-b
CrtY
CruA/P
AL2
γ-Carotene
Chlorobactene
CrtU
Spheroidene pathway
CrtC
CrtY
CrtL-b
CrtC
CrtC
1'-Hydroxytorulene
1'-Hydroxy-γ-carotene
Hydroxychlorobactene
CruC
CruC
Hydroxychlorobactene glucoside
1'-Hydroxy-γ-carotene glucoside
CruD
CruD
Hydroxychlorobactene glucoside ester
1'-Hydroxy-γ-carotene glucoside ester
Hydroxyneurosporene
CrtF
3,4-Dihydrospheroidene
CrtD
β-Zeacarotene
Demethylspheroidene
CrtY
CrtL-b
7,8-Dihydro-β-carotene
CrtF
Hydroxyspheroidene
CrtC
Spheroidene
CrtA
CrtA
CrtC
Hydroxyspheroidenone
Spheroidenone
(2'S)-Deoxymyxol 2'-(2,4-di-O-methyl-α-L-fucoside)
(2'S)-Deoxymyxol 2'-α-L-fucoside
CrtR
CrtR
(3R,2'S)-Myxol 2'-(2,4-di-O-methyl-α-L-fucoside)
(3R,2'S)-Myxol 2'-α-L-fucoside
CrtW
Myxol biosynthesis
(3S,2'S)-4-Ketomyxol 2'-α-L-fucoside
Retinol metabolism
CrtL-b
CrtY
CruA/P
AL2
Strigolactone ABC-rings
β-Carotene
5.2.1.14
9-cis-β-Carotene
1.13.11.68
9-cis-10'-Apo-β-carotenal
1.13.11.69
Carlactone
5-Deoxystrigol
1.14.13.-
Strigol
Isorenieratene
CrtU
CrtU
β-Isorenieratene
Thermobiszeaxanthin
Thermocryptoxanthin
Thermozeaxanthin
Zeaxanthin diglucoside
CrtX
Nostoxanthin
Caloxanthin
Capsanthin
Capsorubin
5.3.99.8
5.3.99.8
CrtR
LUT5
CrtZ
LUT5
CrtR
CrtZ
β-Cryptoxanthin
Zeaxanthin
Low-light condition
1.14.13.90
1.14.13.90
Antheraxanthin
Violaxanthin
1.10.99.3
1.10.99.3
High-light condition
Xanthophyll cycle
5.3.99.9
Neoxanthin
CrtO
CrtW
CrtW
CrtW
CrtW
Echinenone
CrtZ
3-Hydroxyechinenone
CrtZ
Adonixanthin
3'-Hydroxyechinenone
CrtR
CrtZ
CrtZ
CrtO
CrtW
CrtW
CrtW
CrtW
Canthaxanthin
CrtZ
Phoenicoxanthin
CrtZ
Astaxanthin
Astaxanthin biosynthesis
Astaxanthin diester
9-cis-Violaxanthin
9'-cis-Neoxanthin
5.3.99.9
1.13.11.51
1.13.11.51
Abscisic alcohol
Abscisic aldehyde
Abscisate
1.2.3.14
1.1.1.288
Xanthoxin
Xanthoxic acid
Abscisic acid biosynthesis
1.14.13.93
8'-Hydroxyabscisate
Phaseic acid
Dihydrophaseic acid
2.4.1.263
Abscisic acid glucose ester
3.2.1.175
00906 1/27/14
(c) Kanehisa Laboratories

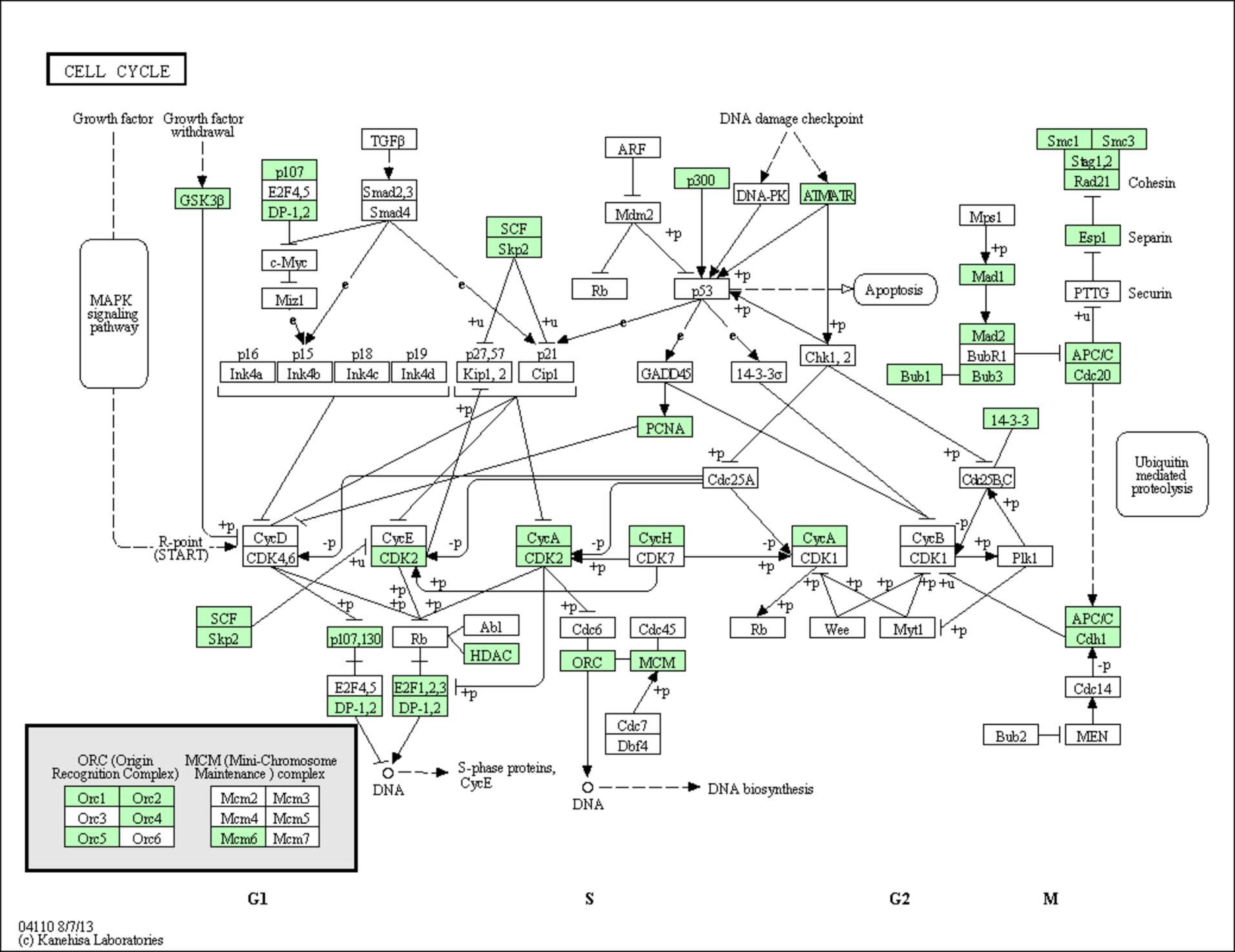
CELL CYCLE
Growth factor
Growth factor withdrawal
MAPK signaling pathway
GSK3β
R-point (START)
p107
E2F4,5
DP-1,2
TGFβ
Smad2,3
Smad4
c-Myc
Miz1
SCF
Skp2
ARF
Mdm2
Rb
DNA damage checkpoint
p300
DNA-PK
ATM/ATR
p53
Apoptosis
Chk1, 2
GADD45
14-3-3σ
PCNA
p16
Ink4a
p15
Ink4b
p18
Ink4c
p19
Ink4d
p27,57
Kip1, 2
p21
Cip1
Mps1
Mad1
Mad2
BubR1
Bub3
Bub1
Smc1
Smc3
Stag1,2
Rad21
Cohesin
Esp1
Separin
PTTG
Securin
APC/C
Cdc20
14-3-3
Cdc25A
Cdc25B,C
Ubiquitin mediated proteolysis
CycD
CDK4,6
CycE
CDK2
CycA
CDK2
CycH
CDK7
CycA
CDK1
CycB
CDK1
Plk1
SCF
Skp2
p107,130
Rb
Abl
HDAC
E2F4,5
DP-1,2
E2F1,2,3
DP-1,2
Cdc6
Cdc45
ORC
MCM
Cdc7
Dbf4
Rb
Wee
Myt1
APC/C
Cdh1
Cdc14
Bub2
MEN
DNA
S-phase proteins, CycE
DNA
DNA biosynthesis
ORC (Origin Recognition Complex)
Orc1
Orc2
Orc3
Orc4
Orc5
Orc6
MCM (Mini-Chromosome Maintenance) complex
Mcm2
Mcm3
Mcm4
Mcm5
Mcm6
Mcm7
G1
S
G2
M
04110 8/7/13
(c) Kanehisa Laboratories

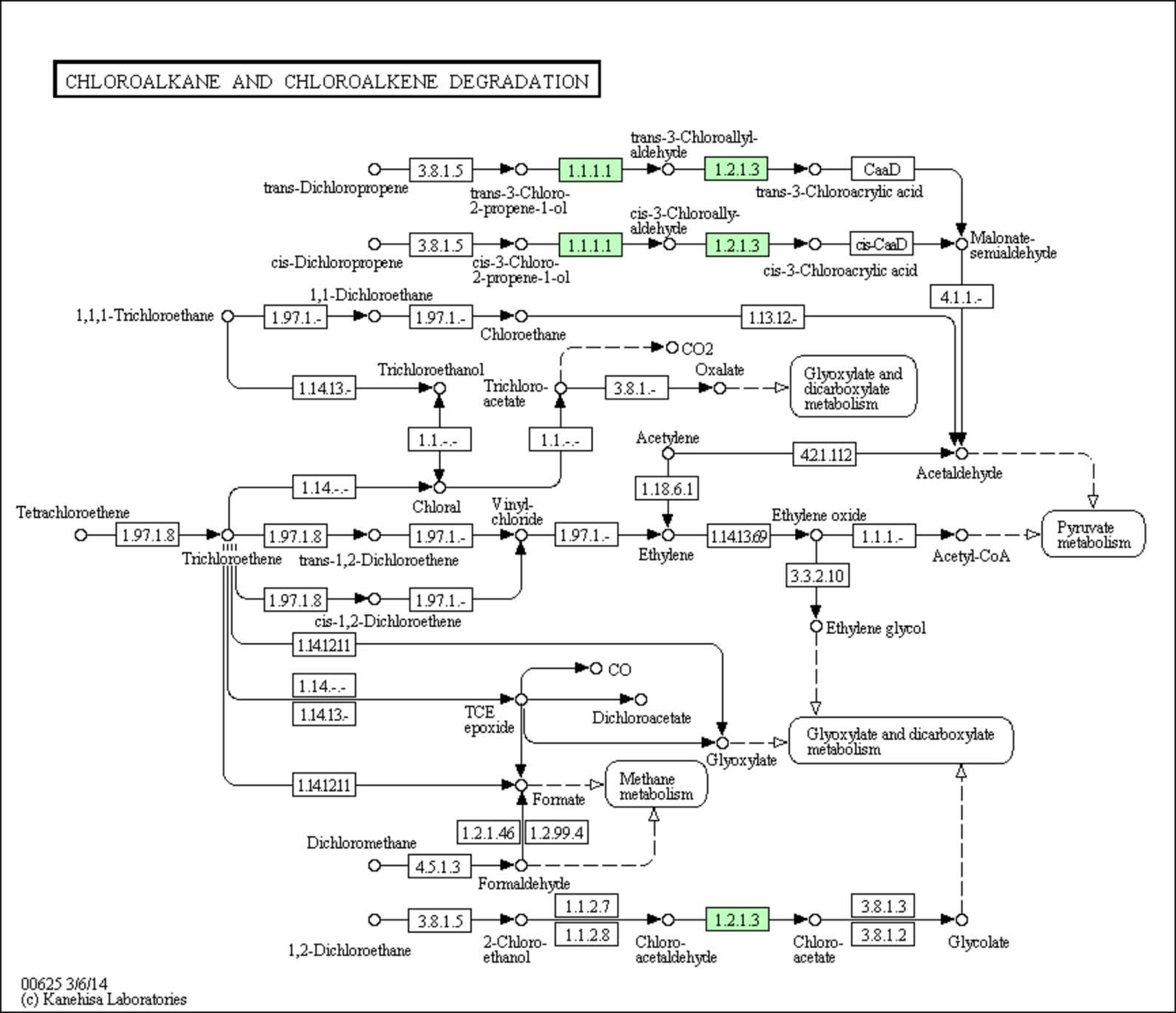

CHLOROALKANE AND CHLOROALKENE DEGRADATION
trans-3-Chloroallyl-aldehyde
trans-Dichloropropene
3.8.1.5
1.1.1.1
1.2.1.3
CaaD
trans-3-Chloro-2-propene-1-ol
trans-3-Chloroacrylic acid
cis-3-Chloroally-aldehyde
cis-Dichloropropene
3.8.1.5
1.1.1.1
1.2.1.3
cis-CaaD
Malonate-semialdehyde
cis-3-Chloro-2-propene-1-ol
cis-3-Chloroacrylic acid
4.1.1.-
1,1-Dichloroethane
1,1,1-Trichloroethane
1.97.1.-
1.97.1.-
1.13.12.-
Chloroethane
CO2
Trichloroethanol
Trichloro-acetate
Oxalate
1.14.13.-
3.8.1.-
Glyoxylate and dicarboxylate metabolism
1.1.-.-
1.1.-.-
Acetylene
4.2.1.112
Acetaldehyde
1.14.-.-
1.18.6.1
Chloral
Tetrachloroethene
Vinyl-chloride
Ethylene oxide
1.97.1.8
1.97.1.8
1.97.1.-
1.97.1.-
1.14.13.69
1.1.1.-
Pyruvate metabolism
Trichloroethene
trans-1,2-Dichloroethene
Ethylene
Acetyl-CoA
3.3.2.10
1.97.1.8
1.97.1.-
cis-1,2-Dichloroethene
Ethylene glycol
1.14.12.11
CO
1.14.-.-
TCE epoxide
Dichloroacetate
1.14.13.-
Glyoxylate and dicarboxylate metabolism
Glyoxylate
Methane metabolism
1.14.12.11
Formate
1.2.1.46
1.2.99.4
Dichloromethane
4.5.1.3
Formaldehyde
1.1.2.7
3.8.1.3
3.8.1.5
1.2.1.3
1.1.2.8
3.8.1.2
2-Chloro-ethanol
Chloro-acetaldehyde
Chloro-acetate
Glycolate
1,2-Dichloroethane
00625 3/6/14
(c) Kanehisa Laboratories

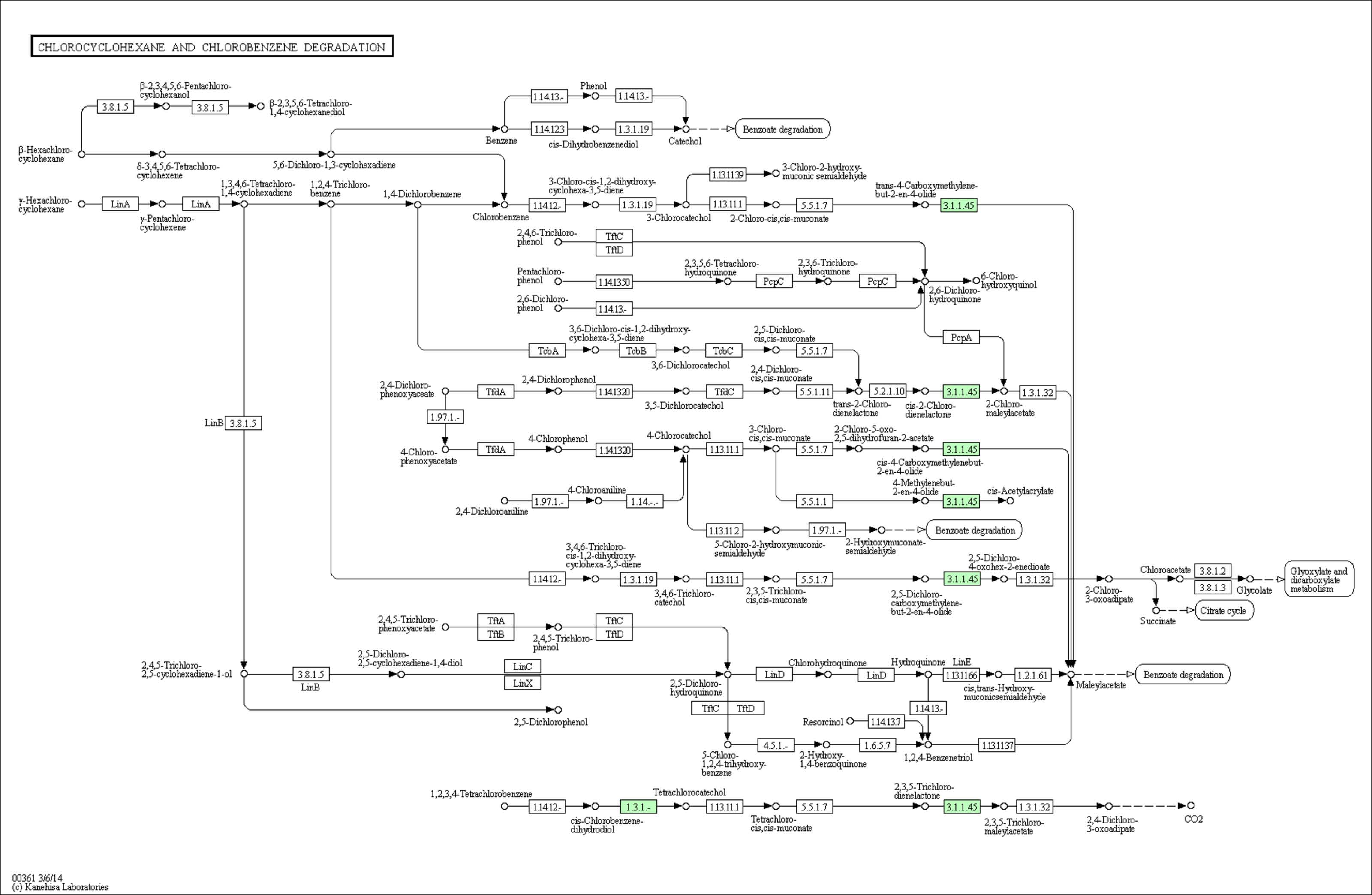

CHLOROCYCLOHEXANE AND CHLOROBENZENE DEGRADATION
β-2,3,4,5,6-Pentachloro-cyclohexanol
β-2,3,5,6-Tetrachloro-1,4-cyclohexanediol
3.8.1.5
β-Hexachloro-cyclohexane
δ-3,4,5,6-Tetrachloro-cyclohexene
5,6-Dichloro-1,3-cyclohexadiene
Phenol
1.14.13.-
1.14.12.3
1.3.1.19
Benzene
cis-Dihydrobenzenediol
Catechol
Benzoate degradation
γ-Hexachloro-cyclohexane
LinA
γ-Pentachloro-cyclohexene
1,3,4,6-Tetrachloro-1,4-cyclohexadiene
1,2,4-Trichloro-benzene
1,4-Dichlorobenzene
Chlorobenzene
1.14.12.-
3-Chloro-cis-1,2-dihydroxy-cyclohexa-3,5-diene
1.13.11.39
3-Chloro-2-hydroxy-muconic semialdehyde
3-Chlorocatechol
1.13.11.1
2-Chloro-cis,cis-muconate
5.5.1.7
trans-4-Carboxymethylene-but-2-en-4-olide
3.1.1.45
2,4,6-Trichloro-phenol
TftC
TftD
Pentachloro-phenol
1.14.13.50
2,3,5,6-Tetrachloro-hydroquinone
PcpC
2,3,6-Trichloro-hydroquinone
6-Chloro-hydroxyquinol
2,6-Dichloro-hydroquinone
2,6-Dichloro-phenol
PcpA
3,6-Dichloro-cis-1,2-dihydroxy-cyclohexa-3,5-diene
TcbA
TcbB
TcbC
3,6-Dichlorocatechol
2,5-Dichloro-cis,cis-muconate
2,4-Dichloro-phenoxyacetate
TfdA
2,4-Dichlorophenol
1.14.13.20
3,5-Dichlorocatechol
TfdC
2,4-Dichloro-cis,cis-muconate
5.5.1.11
trans-2-Chloro-dienelactone
5.2.1.10
cis-2-Chloro-dienelactone
2-Chloro-maleylacetate
1.3.1.32
LinB
1.97.1.-
4-Chloro-phenoxyacetate
4-Chlorophenol
4-Chlorocatechol
3-Chloro-cis,cis-muconate
2-Chloro-5-oxo-2,5-dihydrofuran-2-acetate
cis-4-Carboxymethylenebut-2-en-4-olide
4-Methylenebut-2-en-4-olide
5.5.1.1
cis-Acetylacrylate
4-Chloroaniline
2,4-Dichloroaniline
1.14.-.-
1.13.11.2
5-Chloro-2-hydroxymuconic-semialdehyde
2-Hydroxymuconate-semialdehyde
3,4,6-Trichloro-cis-1,2-dihydroxy-cyclohexa-3,5-diene
3,4,6-Trichloro-catechol
2,3,5-Trichloro-cis,cis-muconate
2,5-Dichloro-carboxymethylene-but-2-en-4-olide
2,5-Dichloro-4-oxohex-2-enedioate
2-Chloro-3-oxoadipate
Chloroacetate
3.8.1.2
3.8.1.3
Glycolate
Glyoxylate and dicarboxylate metabolism
Succinate
Citrate cycle
2,4,5-Trichloro-phenoxyacetate
TftA
TftB
2,4,5-Trichloro-phenol
2,4,5-Trichloro-2,5-cyclohexadiene-1-ol
2,5-Dichloro-2,5-cyclohexadiene-1,4-diol
LinC
LinX
2,5-Dichloro-hydroquinone
LinD
Chlorohydroquinone
Hydroquinone
LinE
1.13.11.66
cis,trans-Hydroxy-muconicsemialdehyde
1.2.1.61
Maleylacetate
2,5-Dichlorophenol
Resorcinol
1.14.13.7
4.5.1.-
1.6.5.7
5-Chloro-1,2,4-trihydroxy-benzene
2-Hydroxy-1,4-benzoquinone
1,2,4-Benzenetriol
1.13.11.37
1,2,3,4-Tetrachlorobenzene
1.3.1.-
cis-Chlorobenzene-dihydrodiol
Tetrachlorocatechol
Tetrachloro-cis,cis-muconate
2,3,5-Trichloro-dienelactone
2,3,5-Trichloro-maleylacetate
2,4-Dichloro-3-oxoadipate
CO2
00361 3/6/14
(c) Kanehisa Laboratories

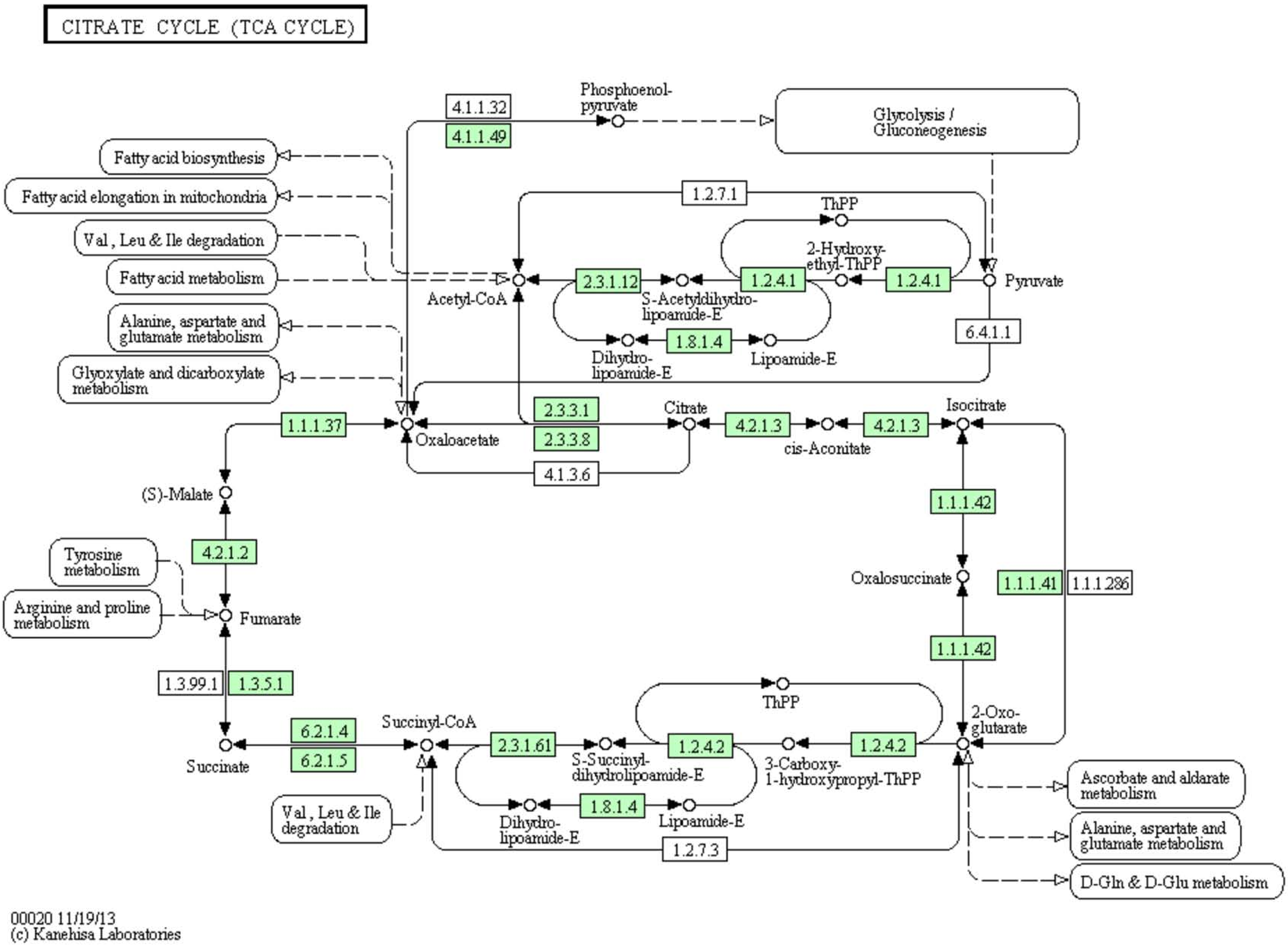
CITRATE CYCLE (TCA CYCLE)
4.1.1.32
4.1.1.49
Phosphoenol-pyruvate
Glycolysis / Gluconeogenesis
Fatty acid biosynthesis
Fatty acid elongation in mitochondria
Val, Leu & Ile degradation
Fatty acid metabolism
Alanine, aspartate and glutamate metabolism
Glyoxylate and dicarboxylate metabolism
1.2.7.1
ThPP
2-Hydroxy-ethyl-ThPP
2.3.1.12
1.2.4.1
1.2.4.1
Pyruvate
Acetyl-CoA
S-Acetyldihydro-lipoamide-E
1.8.1.4
Dihydro-lipoamide-E
Lipoamide-E
6.4.1.1
2.3.3.1
2.3.3.8
Citrate
4.2.1.3
4.2.1.3
Isocitrate
cis-Aconitate
1.1.1.37
Oxaloacetate
4.1.3.6
(S)-Malate
4.2.1.2
1.1.1.42
Oxalosuccinate
1.1.1.41
1.1.1.286
1.1.1.42
Tyrosine metabolism
Arginine and proline metabolism
Fumarate
1.3.99.1
1.3.5.1
ThPP
6.2.1.4
6.2.1.5
Succinyl-CoA
2.3.1.61
1.2.4.2
1.2.4.2
2-Oxo-glutarate
Succinate
S-Succinyl-dihydrolipoamide-E
3-Carboxy-1-hydroxypropyl-ThPP
Val, Leu & Ile degradation
1.8.1.4
Dihydro-lipoamide-E
Lipoamide-E
1.2.7.3
Ascorbate and aldarate metabolism
Alanine, aspartate and glutamate metabolism
D-Gln & D-Glu metabolism
00020 11/19/13
(c) Kanehisa Laboratories

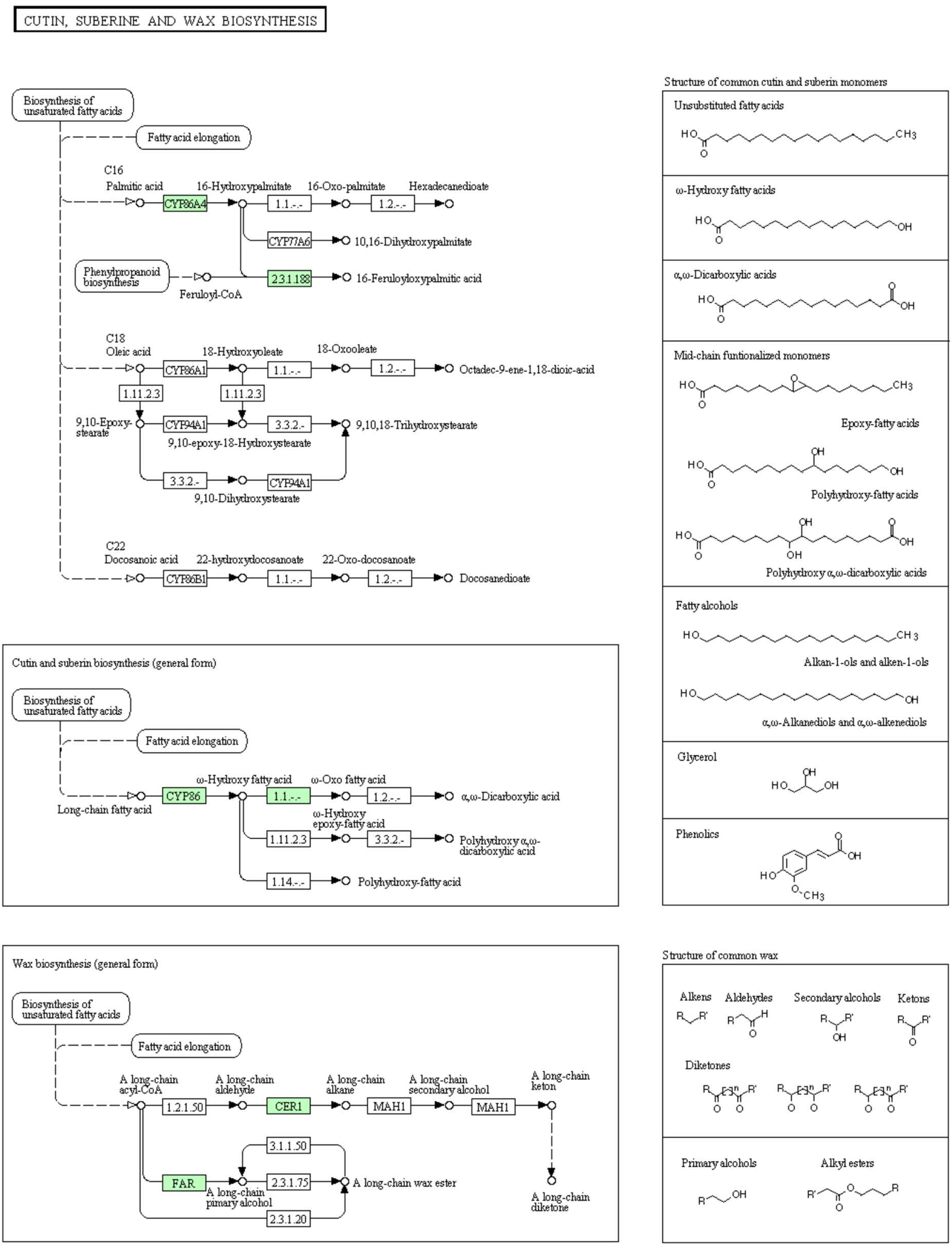

00073 8/23/11
(c) Kanehisa Laboratories

CYANOAMINO ACID METABOLISM

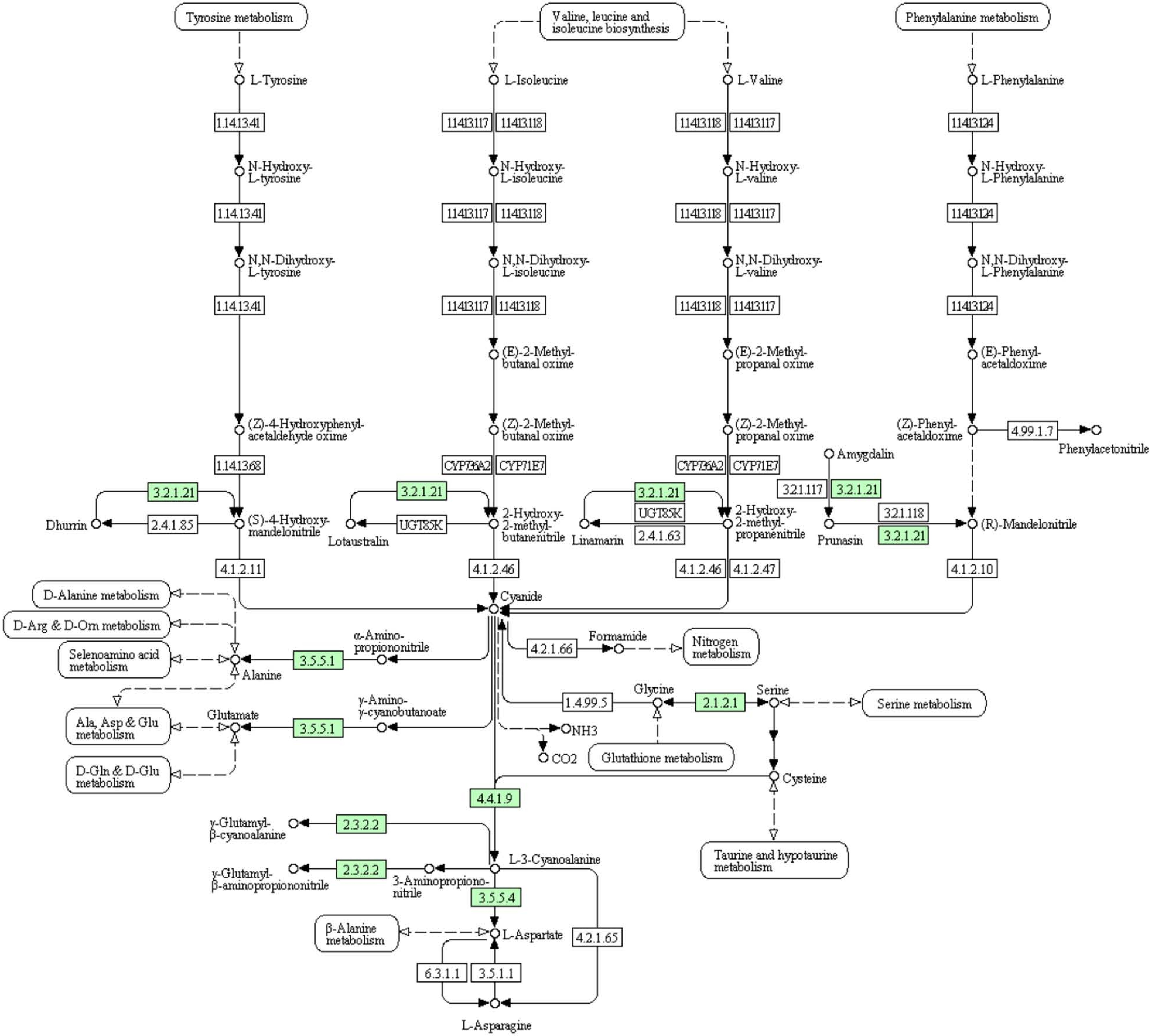

00460 8/29/12

(c) Kanehisa Laboratories

CYSTEINE AND METHIONINE METABOLISM

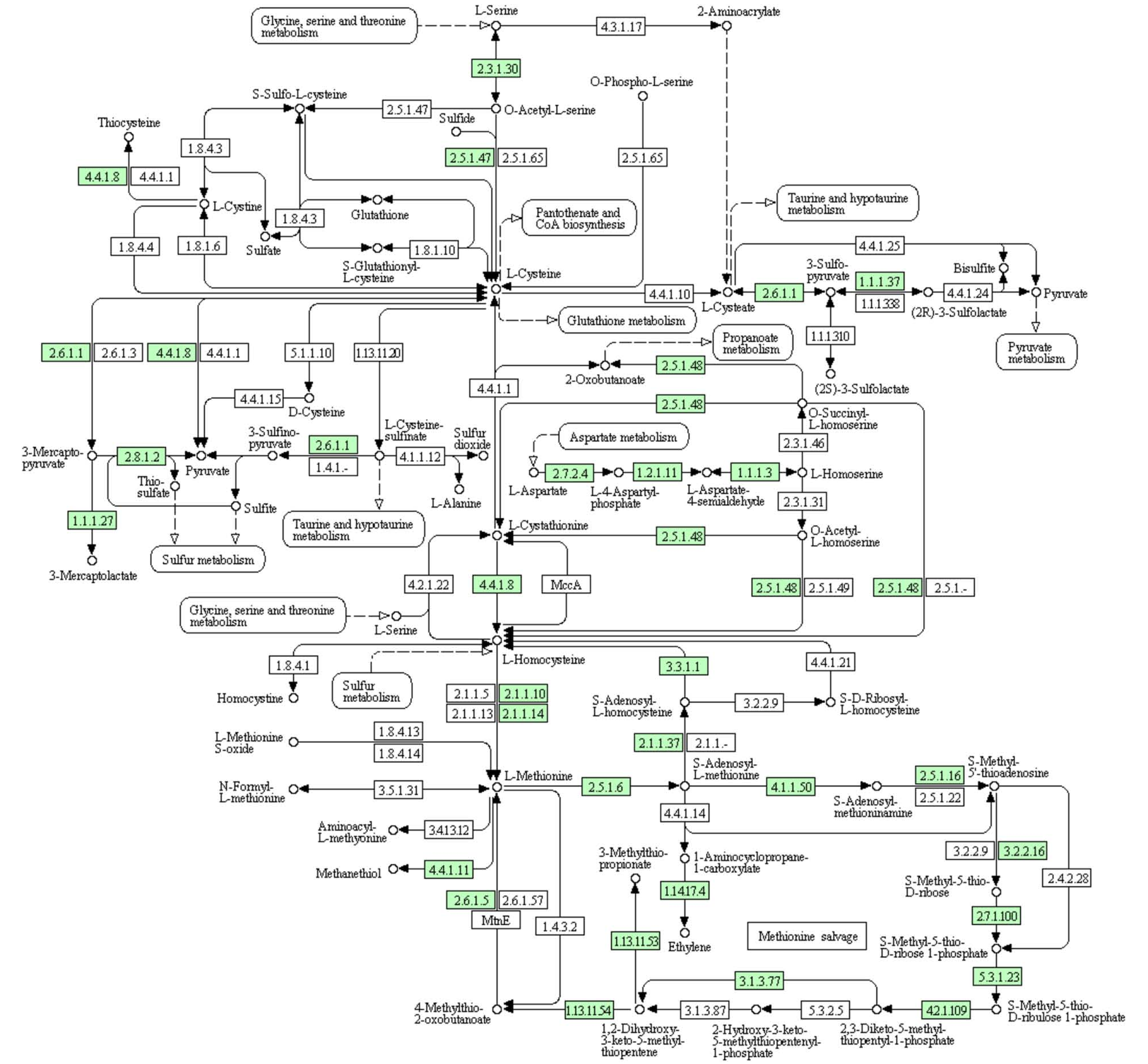

00270 2/4/14

(c) Kanehisa Laboratories

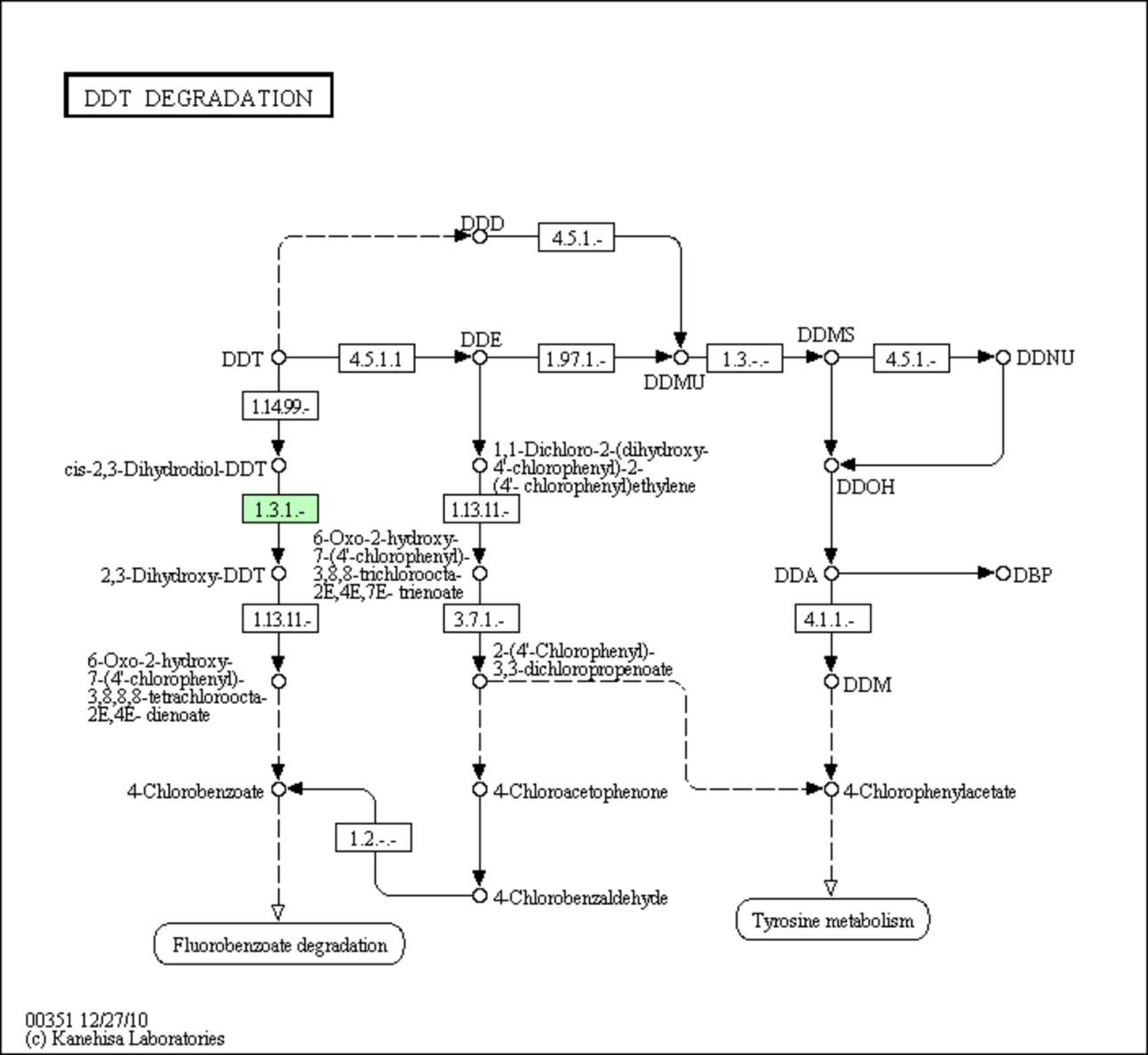

DDT DEGRADATION
DDD
4.5.1.-
DDT
4.5.1.1
DDE
1.97.1.-
DDMU
1.3.-.-
DDMS
4.5.1.-
DDNU
1.14.99.-
cis-2,3-Dihydrodiol-DDT
1.3.1.-
2,3-Dihydroxy-DDT
1.13.11.-
6-Oxo-2-hydroxy-7-(4'-chlorophenyl)-3,8,8,8-tetrachloroocta-2E,4E- dienoate
1,1-Dichloro-2-(dihydroxy-4'-chlorophenyl)-2-(4'- chlorophenyl)ethylene
1.13.11.-
6-Oxo-2-hydroxy-7-(4'-chlorophenyl)-3,8,8-trichloroocta-2E,4E,7E- trienoate
3.7.1.-
2-(4'-Chlorophenyl)-3,3-dichloropropenoate
DDOH
DDA
DBP
4.1.1.-
DDM
4-Chlorobenzoate
1.2.-.-
4-Chloroacetophenone
4-Chlorobenzaldehyde
4-Chlorophenylacetate
Fluorobenzoate degradation
Tyrosine metabolism
00351 12/27/10
(c) Kanehisa Laboratories

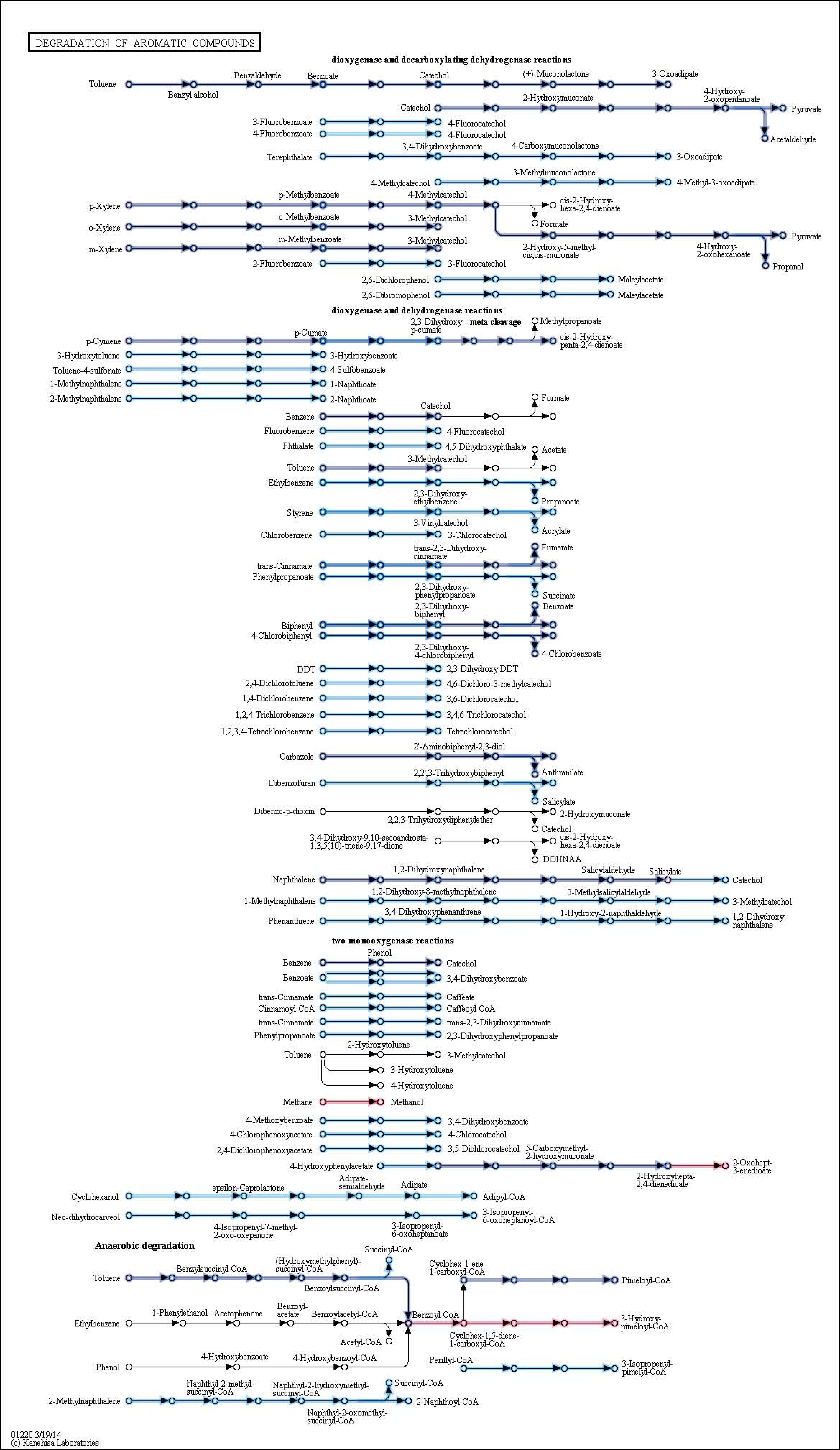
DEGRADATION OF AROMATIC COMPOUNDS
dioxygenase and decarboxylating dehydrogenase reactions
Toluene
Benzyl alcohol
Benzaldehyde
Benzoate
Catechol
(+)-Muconolactone
3-Oxoadipate
Catechol
2-Hydroxymuconate
4-Hydroxy-
2-oxopentanoate
Pyruvate
Acetaldehyde
3-Fluorobenzoate
4-Fluorocatechol
4-Fluorobenzoate
4-Fluorocatechol
Terephthalate
3,4-Dihydroxybenzoate
4-Carboxymuconolactone
3-Oxoadipate
4-Methylcatechol
3-Methylmuconolactone
4-Methyl-3-oxoadipate
p-Xylene
p-Methylbenzoate
4-Methylcatechol
cis-2-Hydroxy-
hexa-2,4-dienoate
Formate
o-Xylene
o-Methylbenzoate
3-Methylcatechol
m-Xylene
m-Methylbenzoate
3-Methylcatechol
2-Hydroxy-5-methyl-
cis,cis-muconate
4-Hydroxy-
2-oxohexanoate
Pyruvate
Propanal
2-Fluorobenzoate
3-Fluorocatechol
2,6-Dichlorophenol
Maleylacetate
2,6-Dibromophenol
Maleylacetate
dioxygenase and dehydrogenase reactions
p-Cymene
p-Cumate
2,3-Dihydroxy-
p-cumate
meta-cleavage
Methylpropanoate
cis-2-Hydroxy-
penta-2,4-dienoate
3-Hydroxytoluene
3-Hydroxybenzoate
Toluene-4-sulfonate
4-Sulfobenzoate
1-Methylnaphthalene
1-Naphthoate
2-Methylnaphthalene
2-Naphthoate
Formate
Catechol
Benzene
Fluorobenzene
4-Fluorocatechol
Phthalate
4,5-Dihydroxyphthalate
Acetate
Toluene
3-Methylcatechol
Ethylbenzene
2,3-Dihydroxy-
ethylbenzene
Propanoate
Styrene
3-Vinylcatechol
Acrylate
Chlorobenzene
3-Chlorocatechol
trans-2,3-Dihydroxy-
cinnamate
Fumarate
trans-Cinnamate
Phenylpropanoate
2,3-Dihydroxy-
phenylpropanoate
Succinate
2,3-Dihydroxy-
biphenyl
Benzoate
Biphenyl
4-Chlorobiphenyl
2,3-Dihydroxy-
4-chlorobiphenyl
4-Chlorobenzoate
DDT
2,3-Dihydroxy DDT
2,4-Dichlorotoluene
4,6-Dichloro-3-methylcatechol
1,4-Dichlorobenzene
3,6-Dichlorocatechol
1,2,4-Trichlorobenzene
3,4,6-Trichlorocatechol
1,2,3,4-Tetrachlorobenzene
Tetrachlorocatechol
2'-Aminobiphenyl-2,3-diol
Carbazole
Anthranilate
2,2',3-Trihydroxybiphenyl
Dibenzofuran
Salicylate
Dibenzo-p-dioxin
2,2,3-Trihydroxydiphenylether
2-Hydroxymuconate
Catechol
3,4-Dihydroxy-9,10-secoandrosta-
1,3,5(10)-triene-9,17-dione
cis-2-Hydroxy-
hexa-2,4-dienoate
DOHNAA
Naphthalene
1,2-Dihydroxynaphthalene
Salicylaldehyde
Salicylate
Catechol
1-Methylnaphthalene
1,2-Dihydroxy-8-methylnaphthalene
3-Methylsalicylaldehyde
3-Methylcatechol
Phenanthrene
3,4-Dihydroxyphenanthrene
1-Hydroxy-2-naphthaldehyde
1,2-Dihydroxy-
naphthalene
two monooxygenase reactions
Benzene
Phenol
Catechol
Benzoate
3,4-Dihydroxybenzoate
trans-Cinnamate
Caffeate
Cinnamoyl-CoA
Caffeoyl-CoA
trans-Cinnamate
trans-2,3-Dihydroxycinnamate
Phenylpropanoate
2,3-Dihydroxyphenylpropanoate
2-Hydroxytoluene
Toluene
3-Methylcatechol
3-Hydroxytoluene
4-Hydroxytoluene
Methane
Methanol
4-Methoxybenzoate
3,4-Dihydroxybenzoate
4-Chlorophenoxyacetate
4-Chlorocatechol
2,4-Dichlorophenoxyacetate
3,5-Dichlorocatechol
5-Carboxymethyl-
2-hydroxymuconate
4-Hydroxyphenylacetate
2-Hydroxyhepta-
2,4-dienedioate
2-Oxohept-
3-enedioate
Cyclohexanol
epsilon-Caprolactone
Adipate-
semialdehyde
Adipate
Adipyl-CoA
Neo-dihydrocarveol
4-Isopropenyl-7-methyl-
2-oxo-oxepanone
3-Isopropenyl-
6-oxoheptanoate
3-Isopropenyl-
6-oxoheptanoyl-CoA
Anaerobic degradation
Succinyl-CoA
Toluene
Benzylsuccinyl-CoA
(Hydroxymethylphenyl)-
succinyl-CoA
Benzoylsuccinyl-CoA
Cyclohex-1-ene-
1-carboxyl-CoA
Pimeloyl-CoA
Ethylbenzene
1-Phenylethanol
Acetophenone
Benzoyl-
acetate
Benzoylacetyl-CoA
Benzoyl-CoA
3-Hydroxy-
pimeloyl-CoA
Acetyl-CoA
Cyclohex-1,5-diene-
1-carboxyl-CoA
Phenol
4-Hydroxybenzoate
4-Hydroxybenzoyl-CoA
Perillyl-CoA
3-Isopropenyl-
pimelyl-CoA
2-Methylnaphthalene
Naphthyl-2-methyl-
succinyl-CoA
Naphthyl-2-hydroxymethyl-
succinyl-CoA
Succinyl-CoA
2-Naphthoyl-CoA
Naphthyl-2-oxomethyl-
succinyl-CoA
01220 3/19/14
(c) Kanehisa Laboratories

DITERPENOID BIOSYNTHESIS

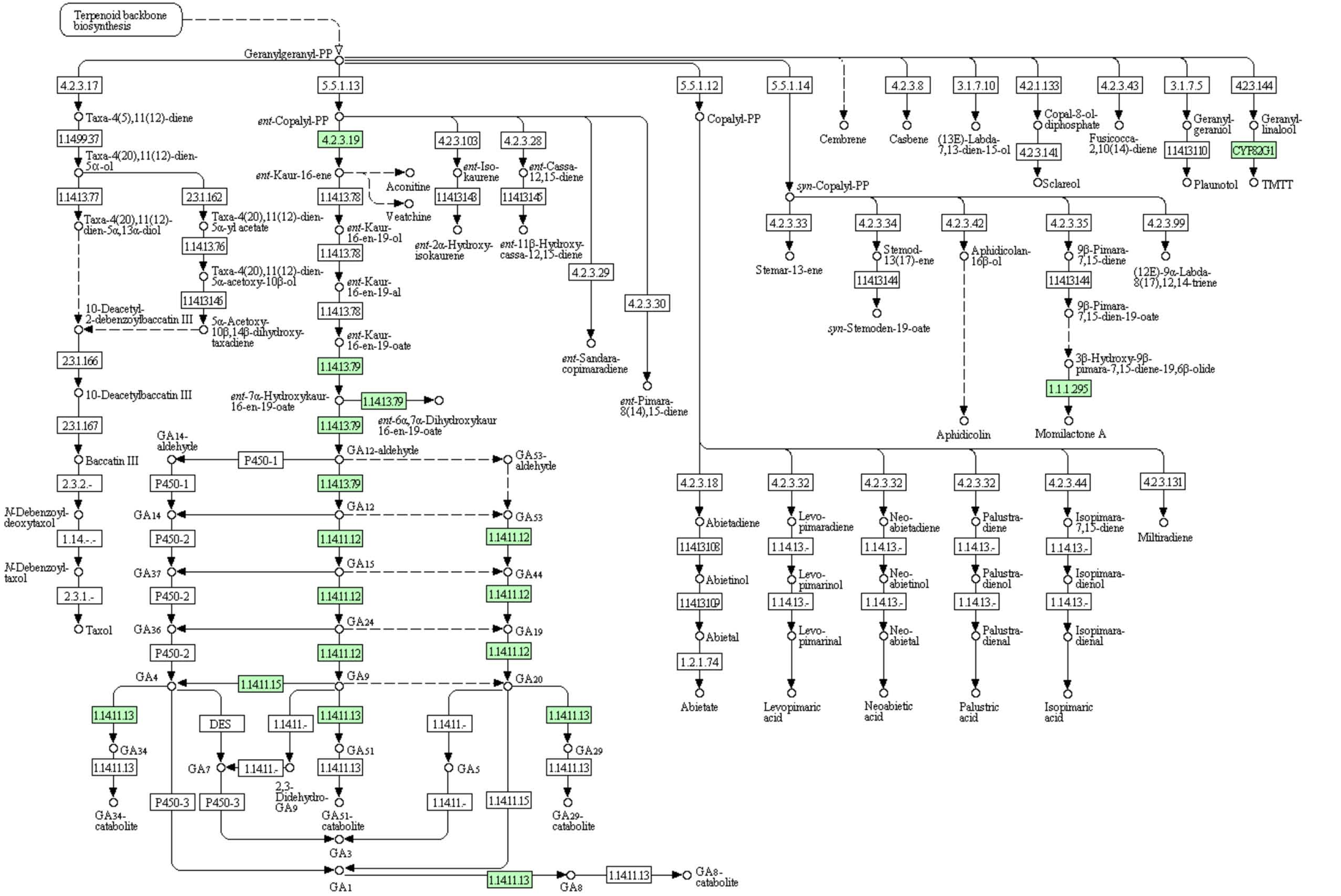

00904 3/13/14

(c) Kanehisa Laboratories

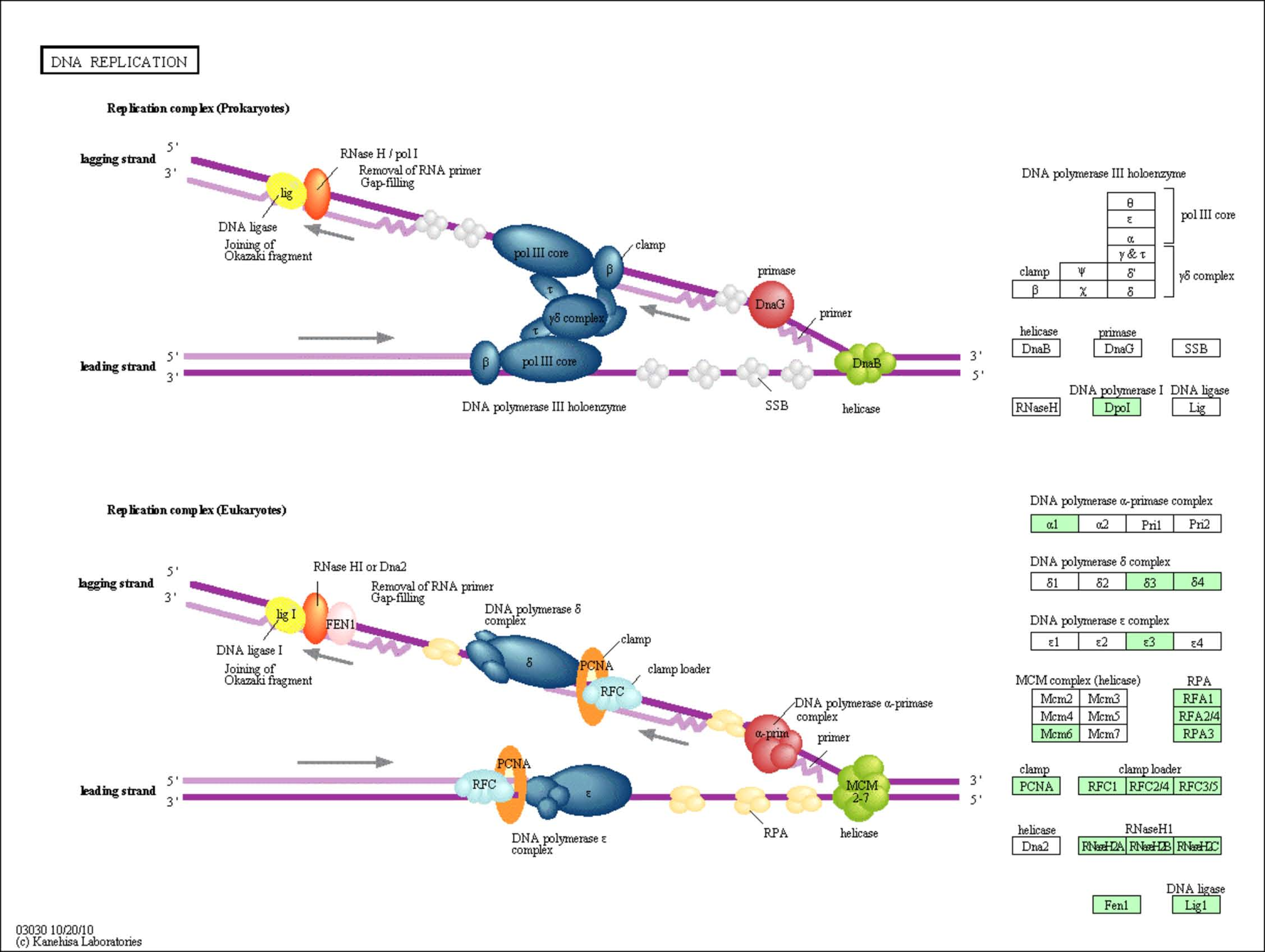
DNA REPLICATION
Replication complex (Prokaryotes)
lagging strand
leading strand
5'
3'
RNase H / pol I
Removal of RNA primer
Gap-filling
lig
DNA ligase
Joining of
Okazaki fragment
pol III core
clamp
β
τ
γδ complex
primase
DnaG
primer
DnaB
SSB
helicase
DNA polymerase III holoenzyme
θ
ε
α
pol III core
γ & τ
ψ
δ'
χ
δ
γδ complex
DnaB
DnaG
SSB
DNA polymerase I
DNA ligase
RNaseH
DpoI
Lig
Replication complex (Eukaryotes)
RNase HI or Dna2
lig I
FEN1
DNA ligase I
DNA polymerase δ
complex
PCNA
RFC
clamp loader
DNA polymerase α-primase
complex
α-prim
MCM
2-7
RPA
DNA polymerase ε
complex
DNA polymerase α-primase complex
α1
α2
Pri1
Pri2
DNA polymerase δ complex
δ1
δ2
δ3
δ4
DNA polymerase ε complex
ε1
ε2
ε3
ε4
MCM complex (helicase)
Mcm2
Mcm3
Mcm4
Mcm5
Mcm6
Mcm7
RFA1
RFA2/4
RPA3
RFC1
RFC2/4
RFC3/5
Dna2
RNaseH1
RNaseH2A
RNaseH2B
RNaseH2C
Fen1
Lig1
03030 10/20/10
(c) Kanehisa Laboratories

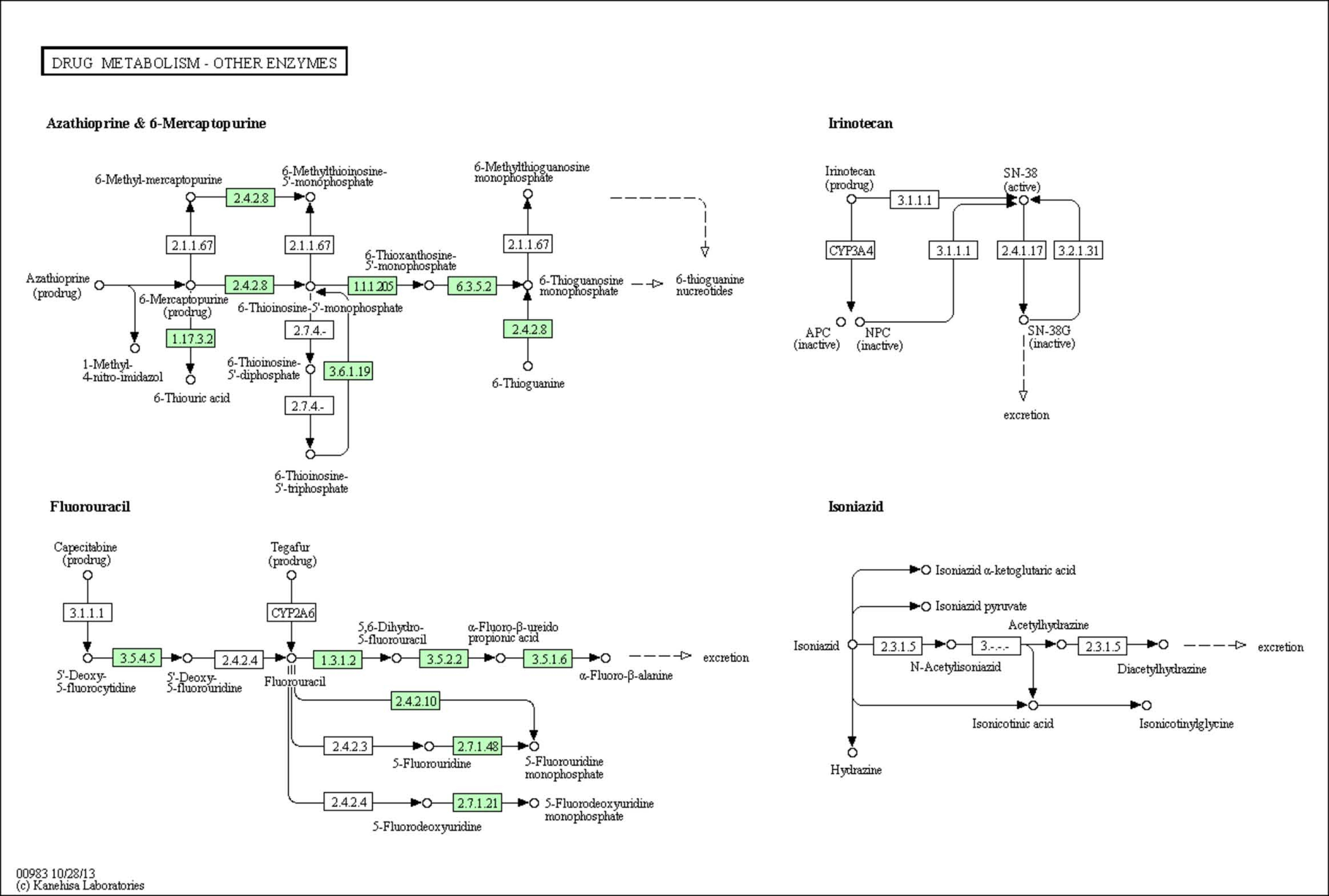

DRUG METABOLISM - OTHER ENZYMES
Azathioprine & 6-Mercaptopurine
6-Methyl-mercaptopurine
2.4.2.8
6-Methylthioinosine-
5'-monophosphate
6-Methylthioguanosine
monophosphate
2.1.67
2.1.67
2.1.67
6-Thioxanthosine-
5'-monophosphate
Azathioprine
(prodrug)
2.4.2.8
1.1.1.205
6.3.5.2
6-Thioguanosine
monophosphate
6-thioguanine
nucreotides
6-Mercaptopurine
(prodrug)
6-Thioinosine-5'-monophosphate
1.17.3.2
2.7.4.-
2.4.2.8
1-Methyl-
4-nitro-imidazol
6-Thioinosine-
5'-diphosphate
3.6.1.19
6-Thioguanine
6-Thiouric acid
2.7.4.-
6-Thioinosine-
5'-triphosphate
Irinotecan
Irinotecan
(prodrug)
SN-38
(active)
3.1.1.1
CYP3A4
3.1.1.1
2.4.1.17
3.2.1.31
APC
(inactive)
NPC
(inactive)
SN-38G
(inactive)
excretion
Fluorouracil
Capecitabine
(prodrug)
Tegafur
(prodrug)
3.1.1.1
CYP2A6
5,6-Dihydro-
5-fluorouracil
α-Fluoro-β-ureido
propionic acid
3.5.4.5
2.4.2.4
1.3.1.2
3.5.2.2
3.5.1.6
excretion
5'-Deoxy-
5-fluorocytidine
5'-Deoxy-
5-fluorouridine
Fluorouracil
α-Fluoro-β-alanine
2.4.2.10
2.4.2.3
2.7.1.48
5-Fluorouridine
5-Fluorouridine
monophosphate
2.4.2.4
2.7.1.21
5-Fluorodeoxyuridine
monophosphate
5-Fluorodeoxyuridine
Isoniazid
Isoniazid α-ketoglutaric acid
Isoniazid pyruvate
Acetylhydrazine
Isoniazid
2.3.1.5
3.-.-.-
2.3.1.5
excretion
N-Acetylisoniazid
Diacetylhydrazine
Isonicotinic acid
Isonicotinylglycine
Hydrazine
00983 10/28/13
(c) Kanehisa Laboratories

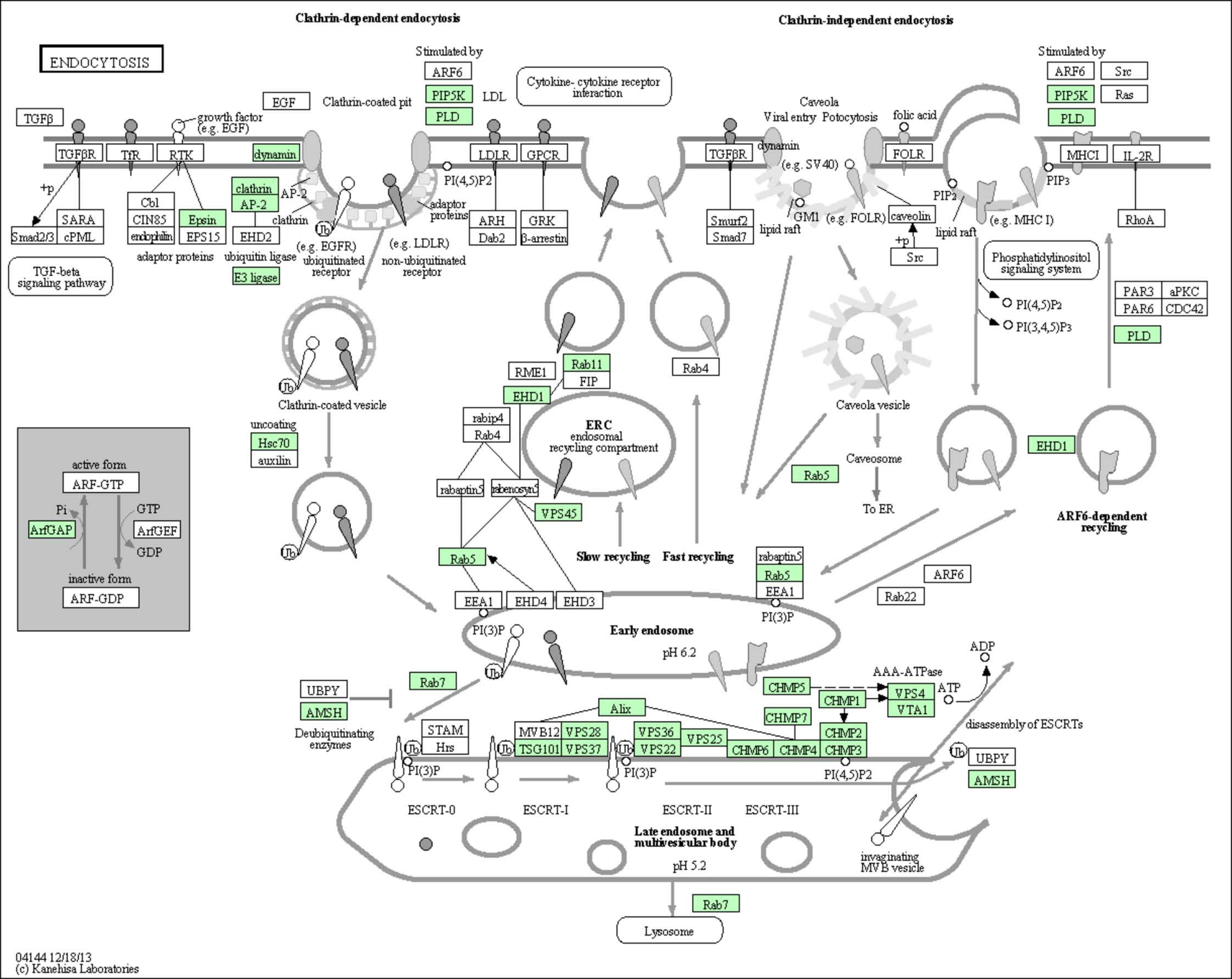
ENDOCYTOSIS
Clathrin-dependent endocytosis
Clathrin-independent endocytosis
Stimulated by
ARF6
PIP5K
PLD
LDL
Cytokine- cytokine receptor interaction
EGF
Clathrin-coated pit
TGFβ
growth factor (e.g. EGF)
TGFβR
TfR
RTK
dynamin
LDLR
GPCR
PI(4,5)P2
+p
Smad2/3
SARA
cPML
Cbl
CIN85
endophilin
Epsin
EPS15
clathrin
AP-2
EHD2
ARH
Dab2
GRK
β-arrestin
adaptor proteins
adaptor proteins
ubiquitin ligase
E3 ligase
(e.g. EGFR) ubiquitinated receptor
(e.g. LDLR) non-ubiquitinated receptor
TGF-beta signaling pathway
Ub
Caveola
Viral entry
Potocytosis
folic acid
dynamin
(e.g. SV40)
FOLR
MHCI
IL-2R
Src
Ras
Smurf2
Smad7
lipid raft
GM1
(e.g. FOLR)
caveolin
+p
Src
PIP2
PIP3
lipid raft
(e.g. MHC I)
RhoA
Phosphatidylinositol signaling system
PI(4,5)P2
PI(3,4,5)P3
PAR3
aPKC
PAR6
CDC42
PLD
Clathrin-coated vesicle
uncoating
Hsc70
auxilin
RME1
Rab11
FIP
EHD1
Rab4
rabip4
Rab4
ERC endosomal recycling compartment
rabaptin5
rabenosyn5
VPS45
Rab5
Slow recycling
Fast recycling
Caveola vesicle
Caveosome
Rab5
To ER
EHD1
ARF6-dependent recycling
active form
ARF-GTP
Pi
GTP
ArfGAP
ArfGEF
GDP
inactive form
ARF-GDP
rabaptin5
Rab5
EEA1
ARF6
Rab22
EEA1
EHD4
EHD3
PI(3)P
PI(3)P
Early endosome
pH 6.2
ADP
AAA-ATPase
ATP
UBPY
AMSH
Rab7
Deubiquitinating enzymes
CHMP5
CHMP1
VPS4
VTA1
Alix
CHMP7
CHMP2
CHMP3
disassembly of ESCRTs
STAM
Hrs
MVB12
TSG101
VPS28
VPS37
VPS36
VPS22
VPS25
CHMP6
CHMP4
PI(3)P
PI(3)P
PI(4,5)P2
UBPY
AMSH
ESCRT-0
ESCRT-I
ESCRT-II
ESCRT-III
Late endosome and multivesicular body
pH 5.2
invaginating MVB vesicle
Rab7
Lysosome
04144 12/18/13
(c) Kanehisa Laboratories

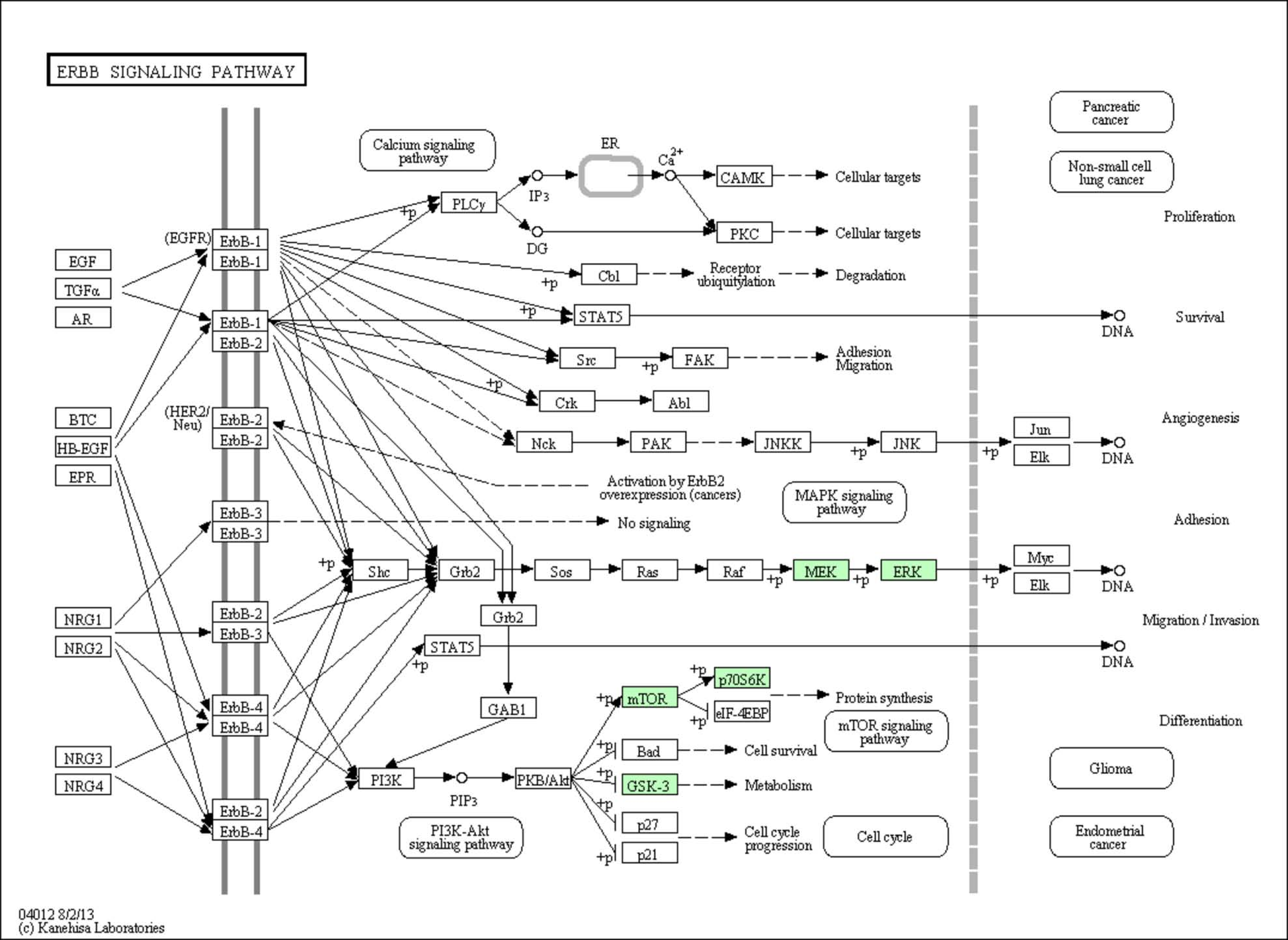
ERBB SIGNALING PATHWAY
EGF
TGFα
AR
BTC
HB-EGF
EPR
NRG1
NRG2
NRG3
NRG4
(EGFR)
ErbB-1
ErbB-1
ErbB-1
ErbB-2
(HER2/Neu)
ErbB-2
ErbB-2
ErbB-3
ErbB-3
ErbB-2
ErbB-3
ErbB-4
ErbB-4
ErbB-2
ErbB-4
Calcium signaling pathway
PLCγ
IP3
DG
ER
Ca2+
CAMK
PKC
Cellular targets
Cellular targets
Cbl
Receptor ubiquitylation
Degradation
STAT5
DNA
Src
FAK
Adhesion Migration
Crk
Abl
Nck
PAK
JNKK
JNK
Jun
Elk
DNA
Activation by ErbB2 overexpression (cancers)
MAPK signaling pathway
No signaling
Shc
Grb2
Sos
Ras
Raf
MEK
ERK
Myc
Elk
DNA
Grb2
STAT5
DNA
GAB1
PI3K
PIP3
PKB/Akt
PI3K-Akt signaling pathway
mTOR
p70S6K
eIF-4EBP
Protein synthesis
mTOR signaling pathway
Bad
Cell survival
GSK-3
Metabolism
p27
p21
Cell cycle progression
Cell cycle
+p
Pancreatic cancer
Non-small cell lung cancer
Proliferation
Survival
Angiogenesis
Adhesion
Migration / Invasion
Differentiation
Glioma
Endometrial cancer
04012 8/2/13
(c) Kanehisa Laboratories

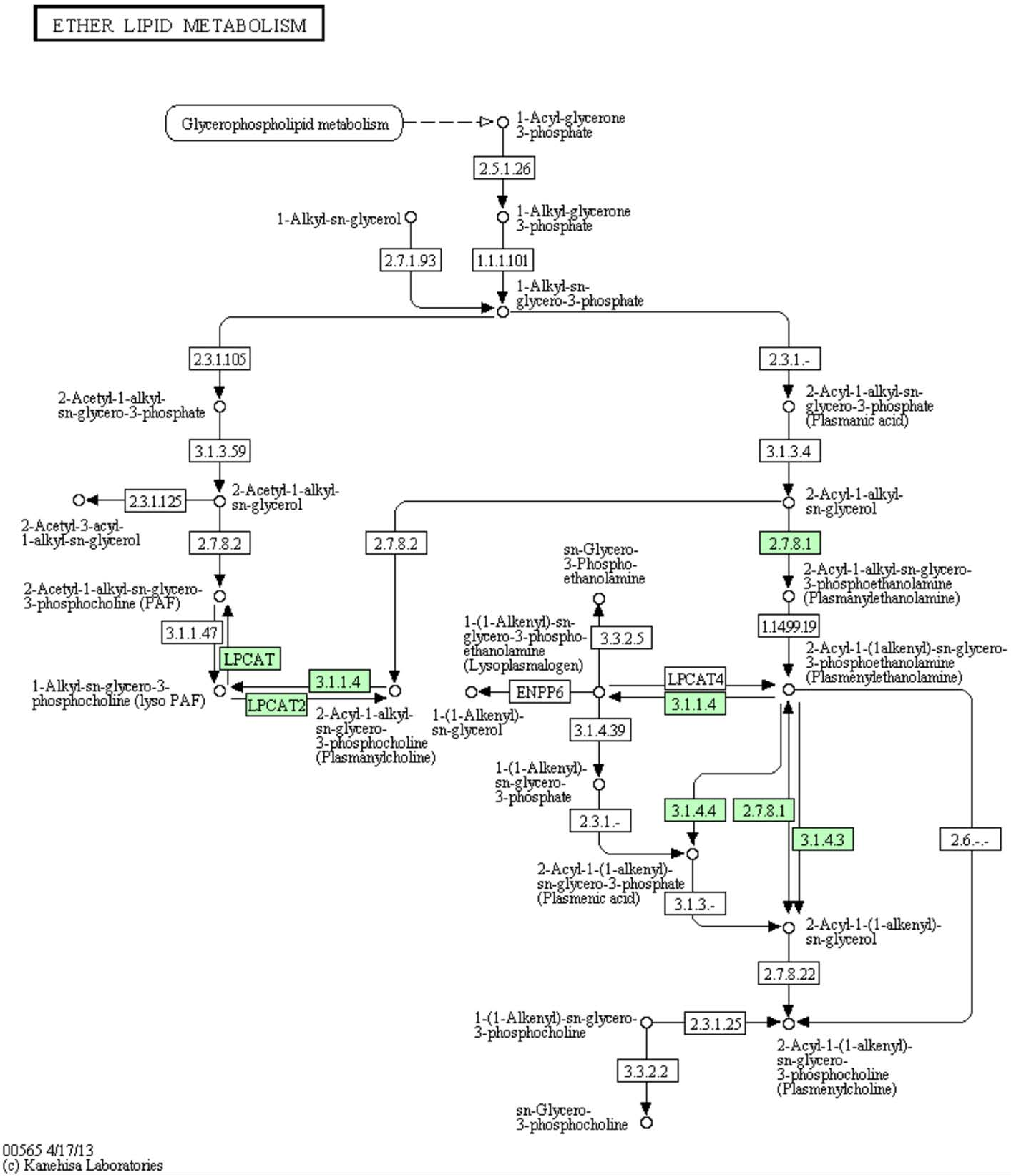
ETHER LIPID METABOLISM
Glycerophospholipid metabolism
1-Acyl-glycerone 3-phosphate
2.5.1.26
1-Alkyl-glycerone 3-phosphate
1-Alkyl-sn-glycerol
2.7.1.93
1.1.1.101
1-Alkyl-sn-glycero-3-phosphate
2.3.1.105
2.3.1.-
2-Acetyl-1-alkyl-sn-glycero-3-phosphate
2-Acyl-1-alkyl-sn-glycero-3-phosphate (Plasmanic acid)
3.1.3.59
3.1.3.4
2.3.1.125
2-Acetyl-1-alkyl-sn-glycerol
2-Acyl-1-alkyl-sn-glycerol
2-Acetyl-3-acyl-1-alkyl-sn-glycerol
2.7.8.2
2.7.8.2
2.7.8.1
sn-Glycero-3-Phospho-ethanolamine
2-Acyl-1-alkyl-sn-glycero-3-phosphoethanolamine (Plasmanylethanolamine)
2-Acetyl-1-alkyl-sn-glycero-3-phosphocholine (PAF)
1.14.99.19
1-(1-Alkenyl)-sn-glycero-3-phospho-ethanolamine (Lysoplasmalogen)
3.3.2.5
2-Acyl-1-(1alkenyl)-sn-glycero-3-phosphoethanolamine (Plasmenylethanolamine)
3.1.1.47
LPCAT
LPCAT4
3.1.1.4
1-Alkyl-sn-glycero-3-phosphocholine (lyso PAF)
ENPP6
LPCAT2
3.1.1.4
2-Acyl-1-alkyl-sn-glycero-3-phosphocholine (Plasmanylcholine)
1-(1-Alkenyl)-sn-glycerol
3.1.4.39
1-(1-Alkenyl)-sn-glycero-3-phosphate
3.1.4.4
2.7.8.1
3.1.4.3
2.3.1.-
2.6.-.-
2-Acyl-1-(1-alkenyl)-sn-glycero-3-phosphate (Plasmenic acid)
3.1.3.-
2-Acyl-1-(1-alkenyl)-sn-glycerol
2.7.8.22
1-(1-Alkenyl)-sn-glycero-3-phosphocholine
2.3.1.25
2-Acyl-1-(1-alkenyl)-sn-glycero-3-phosphocholine (Plasmenylcholine)
3.3.2.2
sn-Glycero-3-phosphocholine
00565 4/17/13
(c) Kanehisa Laboratories

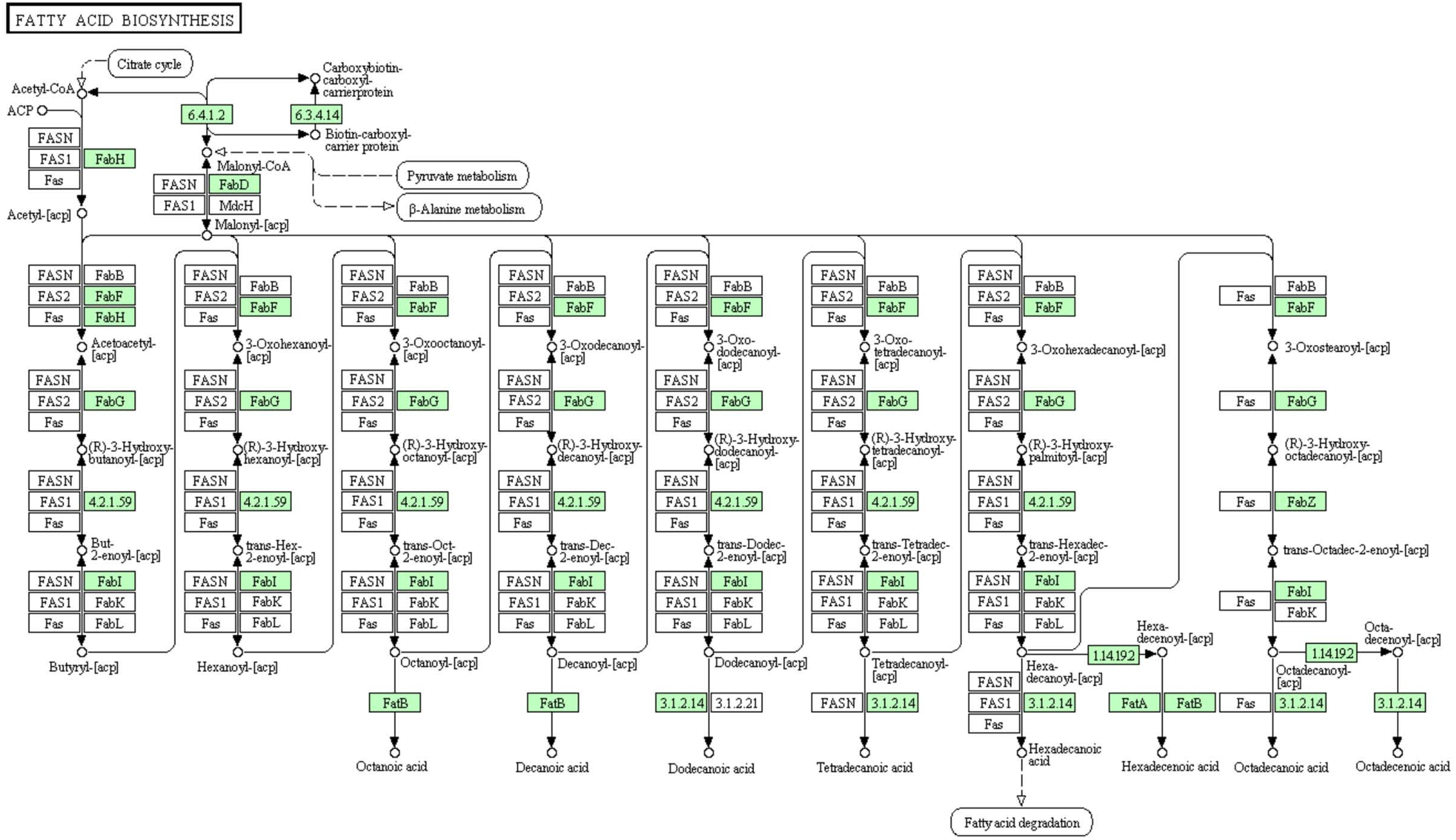

00061 8/30/13
(c) Kanehisa Laboratories

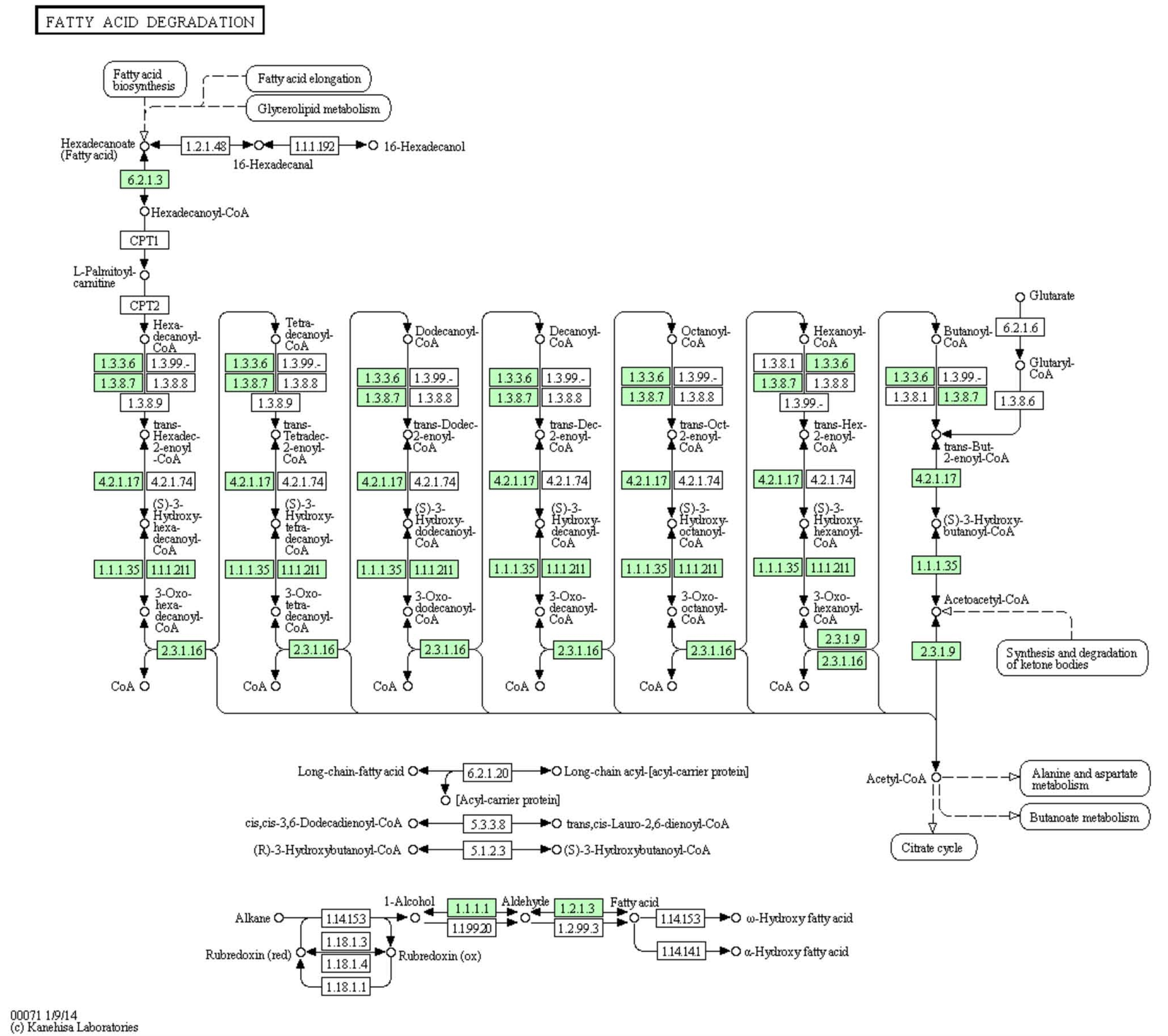
FATTY ACID DEGRADATION
Fatty acid biosynthesis
Fatty acid elongation
Glycerolipid metabolism
Hexadecanoate (Fatty acid)
1.2.1.48
16-Hexadecanal
1.1.1.192
16-Hexadecanol
6.2.1.3
Hexadecanoyl-CoA
CPT1
L-Palmitoyl-carnitine
CPT2
Hexa-decanoyl-CoA
Tetra-decanoyl-CoA
Dodecanoyl-CoA
Decanoyl-CoA
Octanoyl-CoA
Hexanoyl-CoA
Butanoyl-CoA
Glutarate
6.2.1.6
Glutaryl-CoA
1.3.8.6
1.3.3.6
1.3.99.-
1.3.8.7
1.3.8.8
1.3.8.9
1.3.8.1
trans-Hexadec-2-enoyl-CoA
trans-Tetradec-2-enoyl-CoA
trans-Dodec-2-enoyl-CoA
trans-Dec-2-enoyl-CoA
trans-Oct-2-enoyl-CoA
trans-Hex-2-enoyl-CoA
trans-But-2-enoyl-CoA
4.2.1.17
4.2.1.74
(S)-3-Hydroxy-hexadecanoyl-CoA
(S)-3-Hydroxy-tetradecanoyl-CoA
(S)-3-Hydroxy-dodecanoyl-CoA
(S)-3-Hydroxy-decanoyl-CoA
(S)-3-Hydroxy-octanoyl-CoA
(S)-3-Hydroxy-hexanoyl-CoA
(S)-3-Hydroxy-butanoyl-CoA
1.1.1.35
1.1.1.211
3-Oxo-hexadecanoyl-CoA
3-Oxo-tetradecanoyl-CoA
3-Oxo-dodecanoyl-CoA
3-Oxo-decanoyl-CoA
3-Oxo-octanoyl-CoA
3-Oxo-hexanoyl-CoA
Acetoacetyl-CoA
2.3.1.16
2.3.1.9
CoA
Synthesis and degradation of ketone bodies
Acetyl-CoA
Alanine and aspartate metabolism
Butanoate metabolism
Citrate cycle
Long-chain-fatty acid
6.2.1.20
Long-chain acyl-[acyl-carrier protein]
[Acyl-carrier protein]
cis,cis-3,6-Dodecadienoyl-CoA
5.3.3.8
trans,cis-Lauro-2,6-dienoyl-CoA
(R)-3-Hydroxybutanoyl-CoA
5.1.2.3
(S)-3-Hydroxybutanoyl-CoA
Alkane
1.14.15.3
1-Alcohol
1.1.1.1
1.1.99.20
Aldehyde
1.2.1.3
1.2.99.3
Fatty acid
1.14.15.3
ω-Hydroxy fatty acid
1.14.14.1
α-Hydroxy fatty acid
1.18.1.3
1.18.1.4
1.18.1.1
Rubredoxin (red)
Rubredoxin (ox)
00071 1/9/14
(c) Kanehisa Laboratories

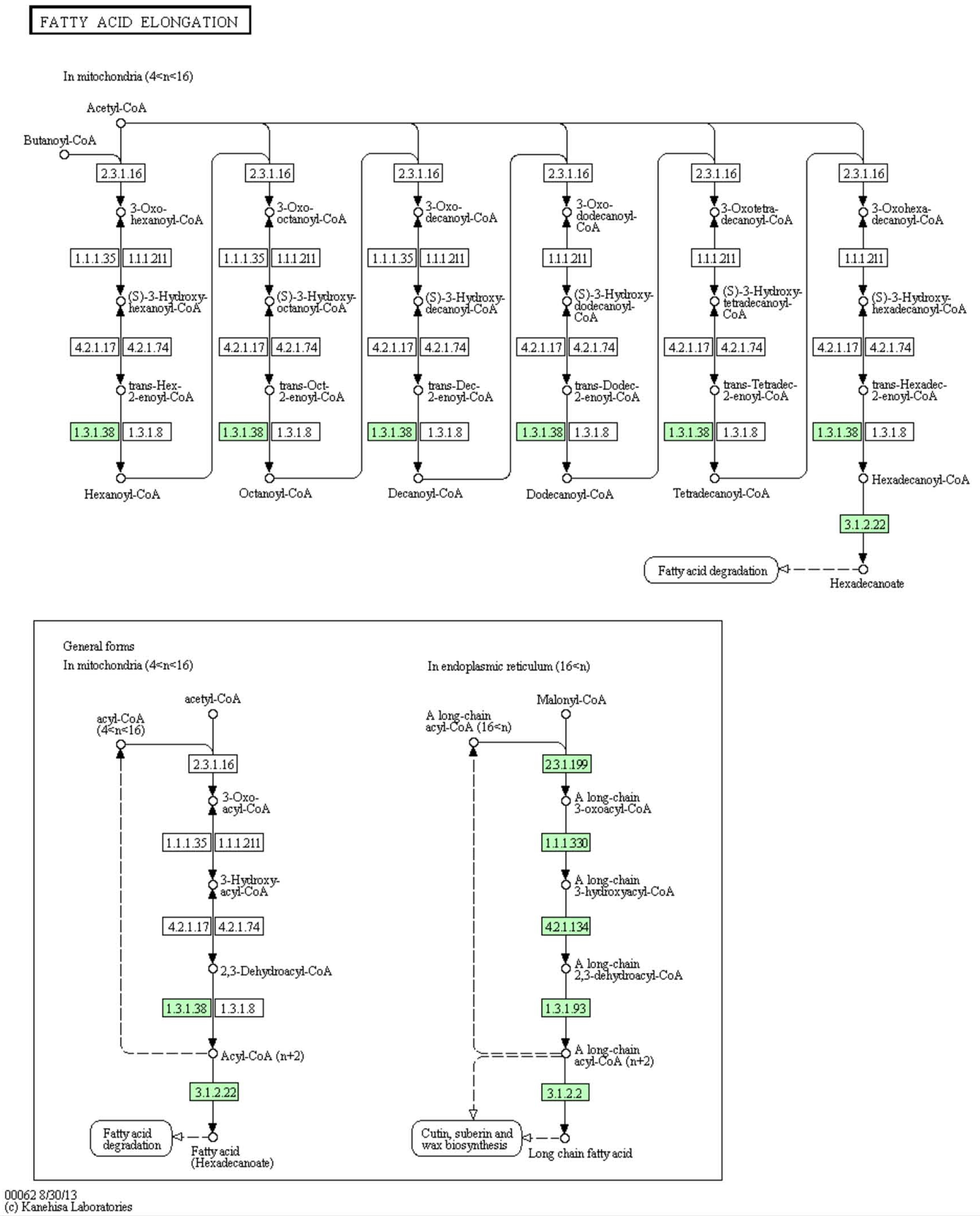

FATTY ACID ELONGATION
In mitochondria (4<n<16)
Acetyl-CoA
Butanoyl-CoA
2.3.1.16
3-Oxo-hexanoyl-CoA
3-Oxo-octanoyl-CoA
3-Oxo-decanoyl-CoA
3-Oxo-dodecanoyl-CoA
3-Oxotetra-decanoyl-CoA
3-Oxohexa-decanoyl-CoA
1.1.1.35
1.1.1.211
(S)-3-Hydroxy-hexanoyl-CoA
(S)-3-Hydroxy-octanoyl-CoA
(S)-3-Hydroxy-decanoyl-CoA
(S)-3-Hydroxy-dodecanoyl-CoA
(S)-3-Hydroxy-tetradecanoyl-CoA
(S)-3-Hydroxy-hexadecanoyl-CoA
4.2.1.17
4.2.1.74
trans-Hex-2-enoyl-CoA
trans-Oct-2-enoyl-CoA
trans-Dec-2-enoyl-CoA
trans-Dodec-2-enoyl-CoA
trans-Tetradec-2-enoyl-CoA
trans-Hexadec-2-enoyl-CoA
1.3.1.38
1.3.1.8
Hexanoyl-CoA
Octanoyl-CoA
Decanoyl-CoA
Dodecanoyl-CoA
Tetradecanoyl-CoA
Hexadecanoyl-CoA
3.1.2.22
Fatty acid degradation
Hexadecanoate
General forms
In mitochondria (4<n<16)
In endoplasmic reticulum (16<n)
acetyl-CoA
acyl-CoA (4<n<16)
2.3.1.16
3-Oxo-acyl-CoA
1.1.1.35
1.1.1.211
3-Hydroxy-acyl-CoA
4.2.1.17
4.2.1.74
2,3-Dehydroacyl-CoA
1.3.1.38
1.3.1.8
Acyl-CoA (n+2)
3.1.2.22
Fatty acid degradation
Fatty acid (Hexadecanoate)
Malonyl-CoA
A long-chain acyl-CoA (16<n)
2.3.1.199
A long-chain 3-oxoacyl-CoA
1.1.1.330
A long-chain 3-hydroxyacyl-CoA
4.2.1.134
A long-chain 2,3-dehydroacyl-CoA
1.3.1.93
A long-chain acyl-CoA (n+2)
3.1.2.2
Cutin, suberin and wax biosynthesis
Long chain fatty acid
00062 8/30/13
(c) Kanehisa Laboratories

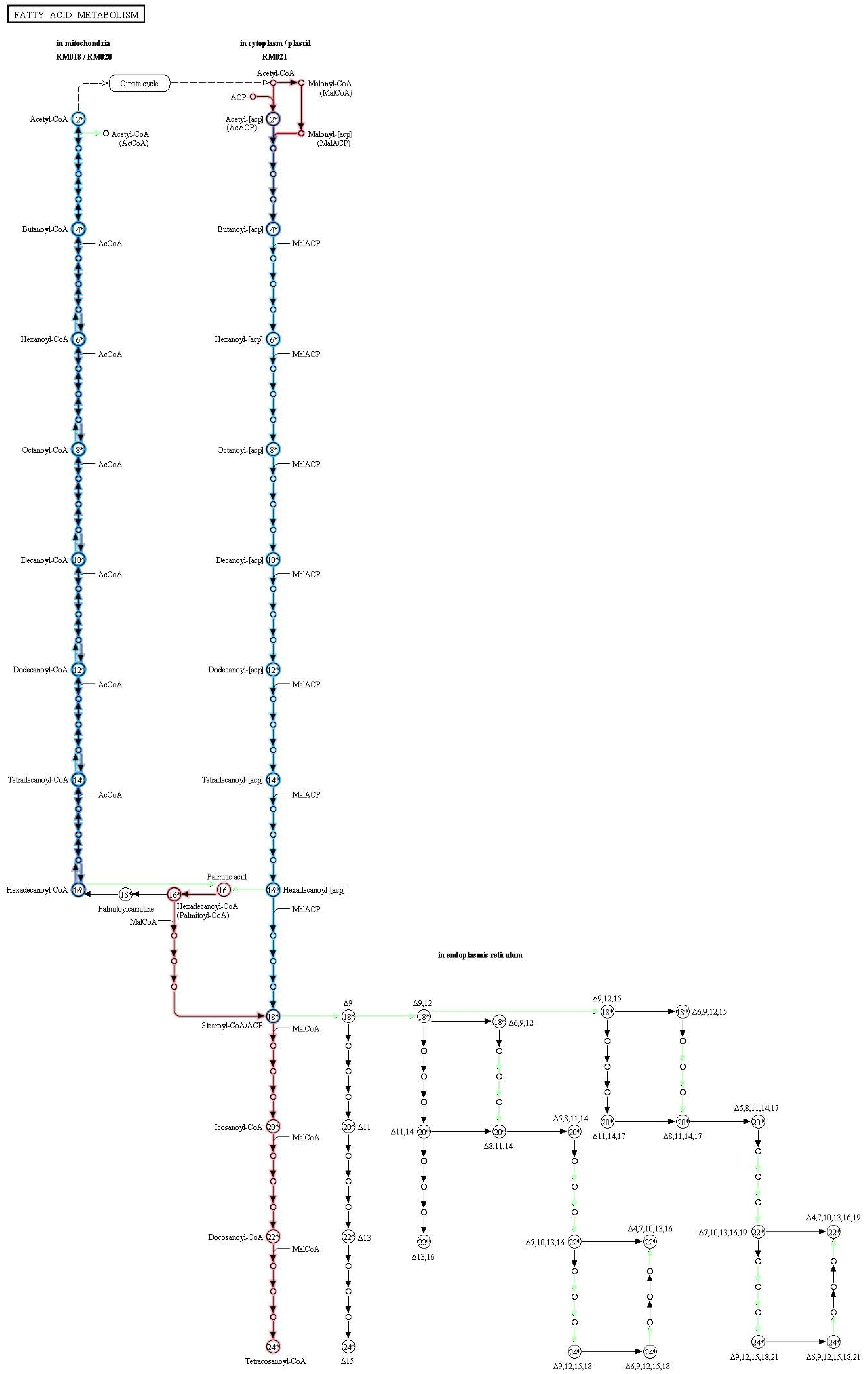

01212 10/8/13
(c) Kanehisa Laboratories

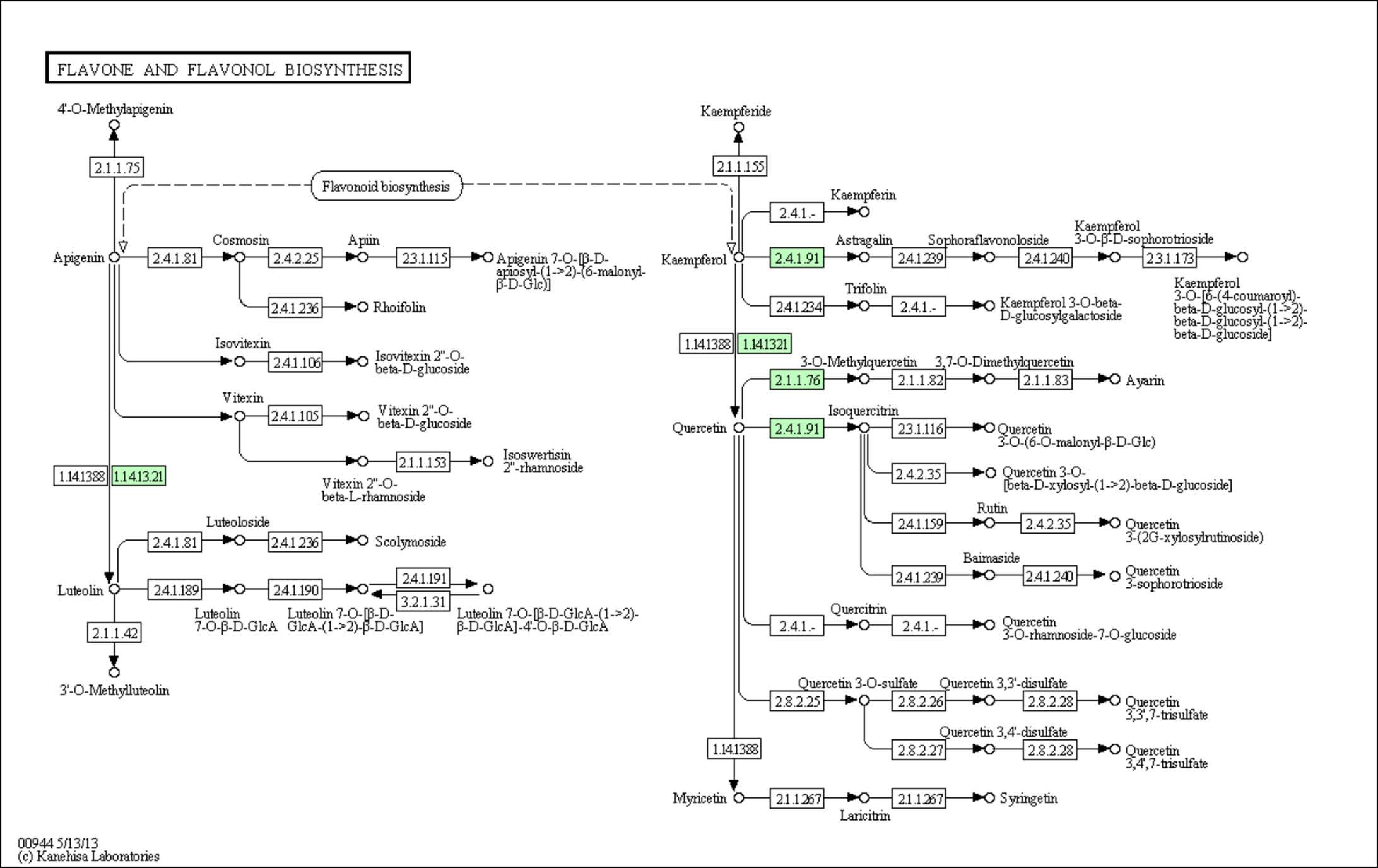
FLAVONE AND FLAVONOL BIOSYNTHESIS
4'-O-Methylapigenin
2.1.1.75
Flavonoid biosynthesis
Apigenin
2.4.1.81
Cosmosin
2.4.2.25
Apiin
2.3.1.115
Apigenin 7-O-[β-D-apiosyl-(1->2)-(6-malonyl-β-D-Glc)]
2.4.1.236
Rhoifolin
Isovitexin
2.4.1.106
Isovitexin 2''-O-beta-D-glucoside
Vitexin
2.4.1.105
Vitexin 2''-O-beta-D-glucoside
2.1.1.153
Isoswertisin 2''-rhamnoside
Vitexin 2''-O-beta-L-rhamnoside
1.14.13.88
1.14.13.21
Luteoloside
2.4.1.81
2.4.1.236
Scolymoside
Luteolin
2.4.1.189
2.4.1.190
2.4.1.191
3.2.1.31
Luteolin 7-O-β-D-GlcA
Luteolin 7-O-[β-D-GlcA-(1->2)-β-D-GlcA]
Luteolin 7-O-[β-D-GlcA-(1->2)-β-D-GlcA]-4'-O-β-D-GlcA
2.1.1.42
3'-O-Methylluteolin
Kaempferide
2.1.1.155
Kaempferin
2.4.1.-
Kaempferol
2.4.1.91
Astragalin
2.4.1.239
Sophoraflavonoloside
2.4.1.240
Kaempferol 3-O-β-D-sophorotrioside
2.3.1.173
Kaempferol 3-O-[6-(4-coumaroyl)-beta-D-glucosyl-(1->2)-beta-D-glucosyl-(1->2)-beta-D-glucoside]
Trifolin
2.4.1.234
2.4.1.-
Kaempferol 3-O-beta-D-glucosylgalactoside
1.14.13.88
1.14.13.21
3-O-Methylquercetin
3,7-O-Dimethylquercetin
2.1.1.76
2.1.1.82
2.1.1.83
Ayarin
Quercetin
2.4.1.91
Isoquercitrin
2.3.1.116
Quercetin 3-O-(6-O-malonyl-β-D-Glc)
2.4.2.35
Quercetin 3-O-[beta-D-xylosyl-(1->2)-beta-D-glucoside]
2.4.1.159
Rutin
2.4.2.35
Quercetin 3-(2G-xylosylrutinoside)
2.4.1.239
Baimaside
2.4.1.240
Quercetin 3-sophorotrioside
2.4.1.-
Quercitrin
2.4.1.-
Quercetin 3-O-rhamnoside-7-O-glucoside
2.8.2.25
Quercetin 3-O-sulfate
2.8.2.26
Quercetin 3,3'-disulfate
2.8.2.28
Quercetin 3,3',7-trisulfate
2.8.2.27
Quercetin 3,4'-disulfate
2.8.2.28
Quercetin 3,4',7-trisulfate
1.14.13.88
Myricetin
2.1.1.267
Laricitrin
2.1.1.267
Syringetin
00944 5/13/13
(c) Kanehisa Laboratories

FLAVONOID BIOSYNTHESIS

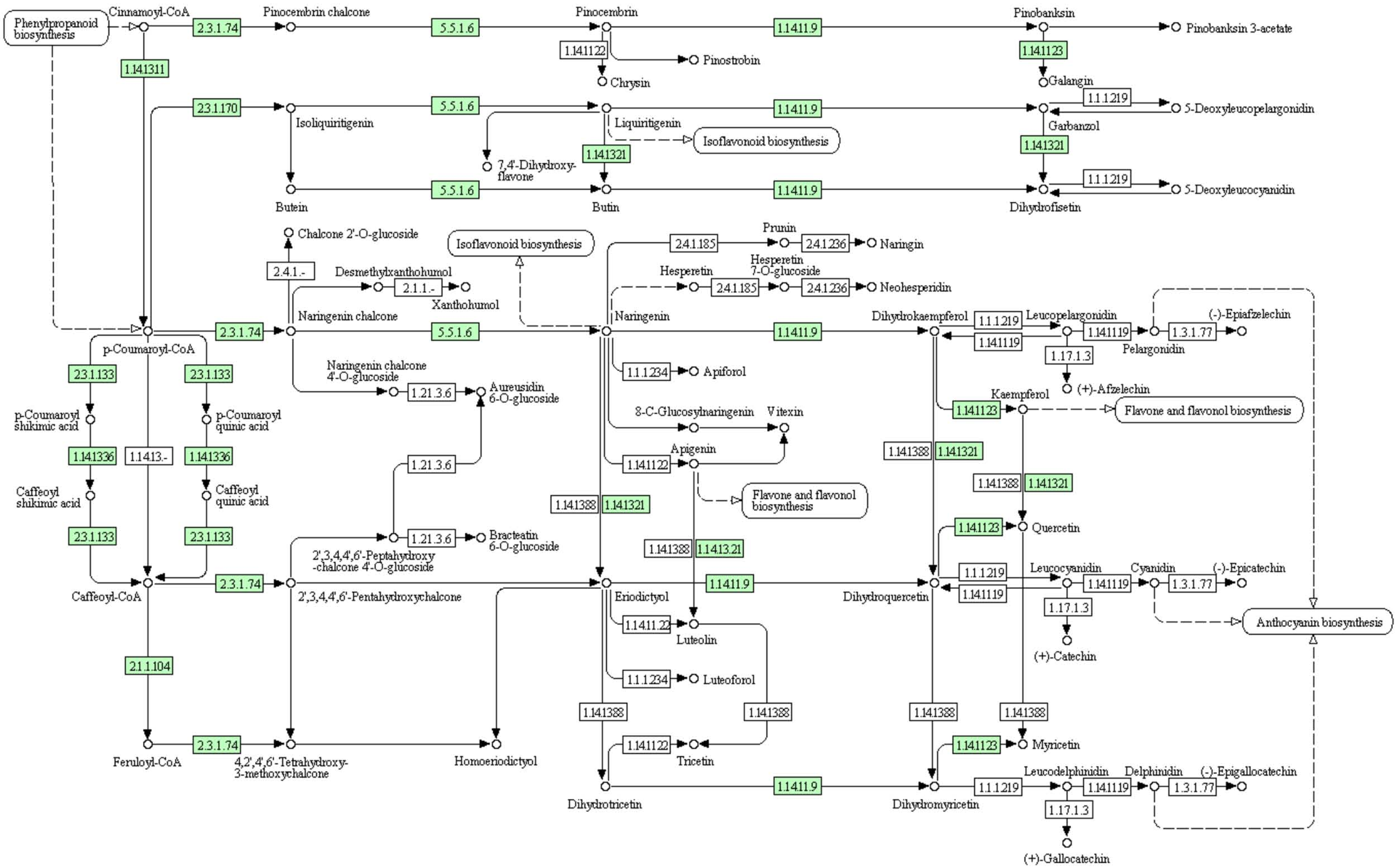

00941 5/13/13

(c) Kanehisa Laboratories

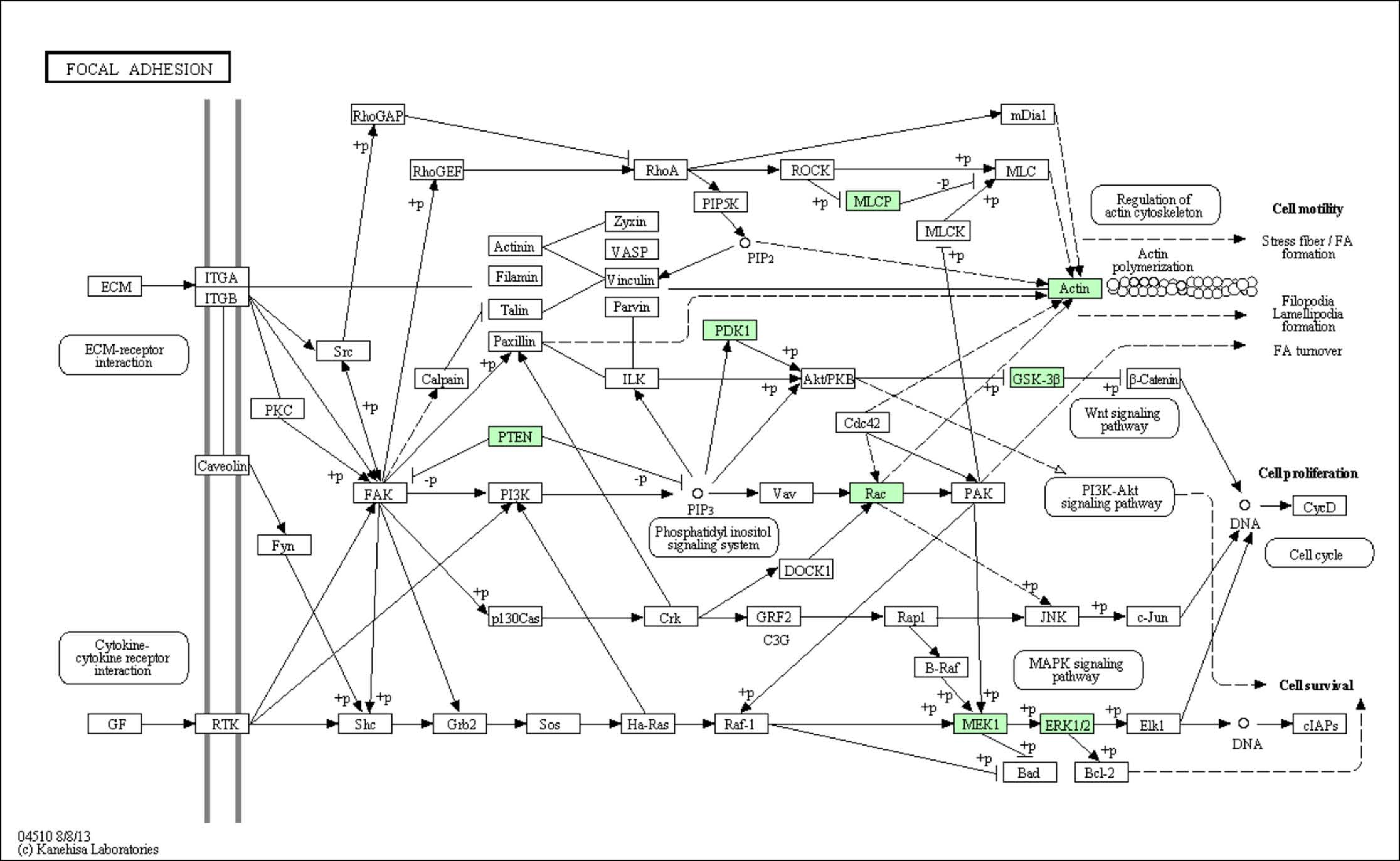
FOCAL ADHESION
ECM
ITGA
ITGB
ECM-receptor interaction
Src
RhoGAP
RhoGEF
RhoA
mDia1
ROCK
MLC
MLCP
MLCK
PIP5K
PIP2
Zyxin
VASP
Actinin
Filamin
Vinculin
Talin
Parvin
Paxillin
Calpain
ILK
PDK1
Akt/PKB
GSK-3β
β-Catenin
Wnt signaling pathway
PKC
Caveolin
Fyn
PTEN
FAK
PI3K
PIP3
Phosphatidyl inositol signaling system
Vav
Rac
PAK
Cdc42
PI3K-Akt signaling pathway
DOCK1
p130Cas
Crk
GRF2
C3G
Rap1
JNK
c-Jun
B-Raf
MAPK signaling pathway
Cytokine-cytokine receptor interaction
GF
RTK
Shc
Grb2
Sos
Ha-Ras
Raf-1
MEK1
ERK1/2
Elk1
Bad
Bcl-2
DNA
cIAPs
CycD
Cell cycle
Regulation of actin cytoskeleton
Actin
Actin polymerization
Cell motility
Stress fiber / FA formation
Filopodia Lamellipodia formation
FA turnover
Cell proliferation
Cell survival
04510 8/8/13
(c) Kanehisa Laboratories

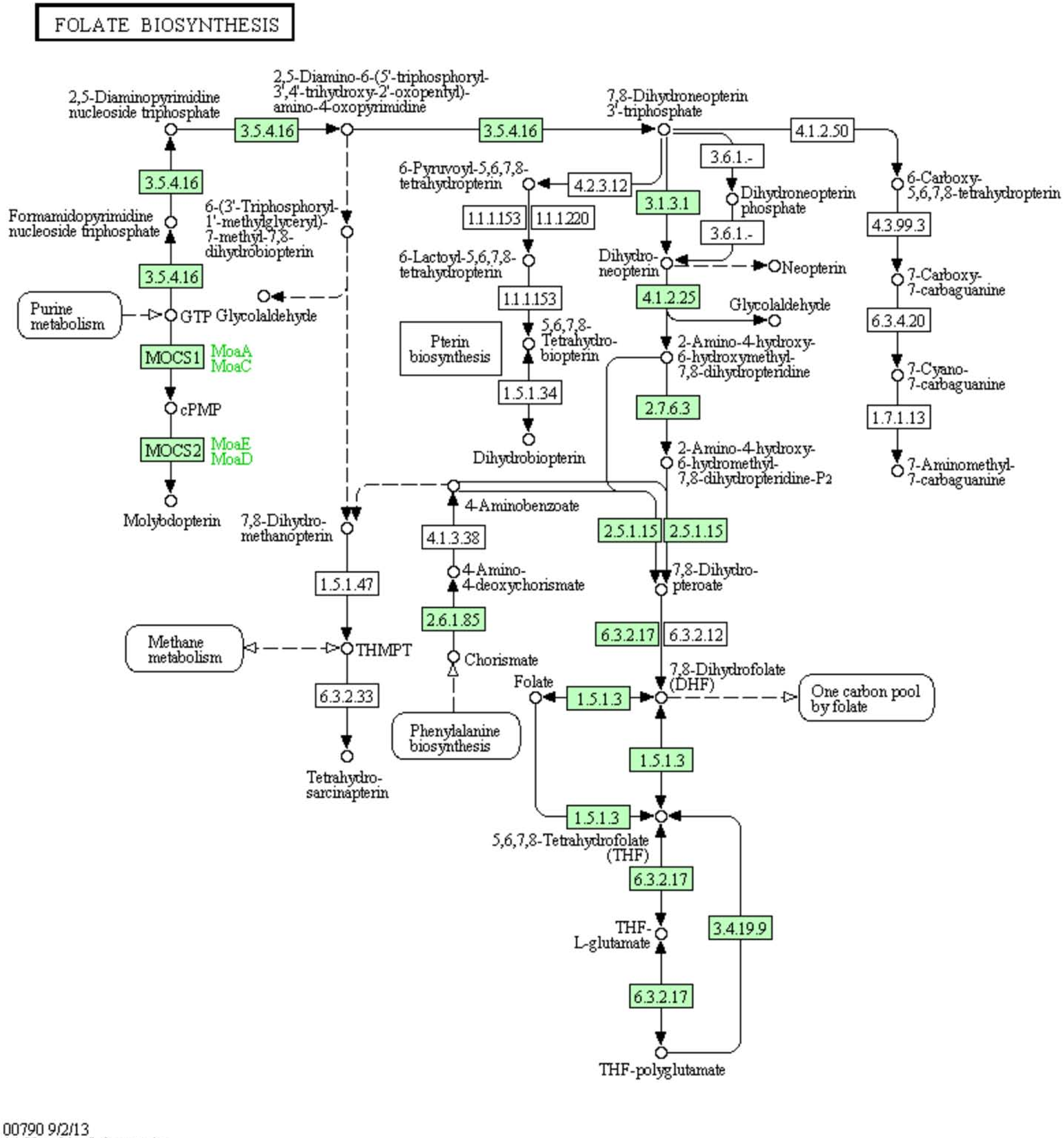

00790 9/2/13

(c) Kanehisa Laboratories

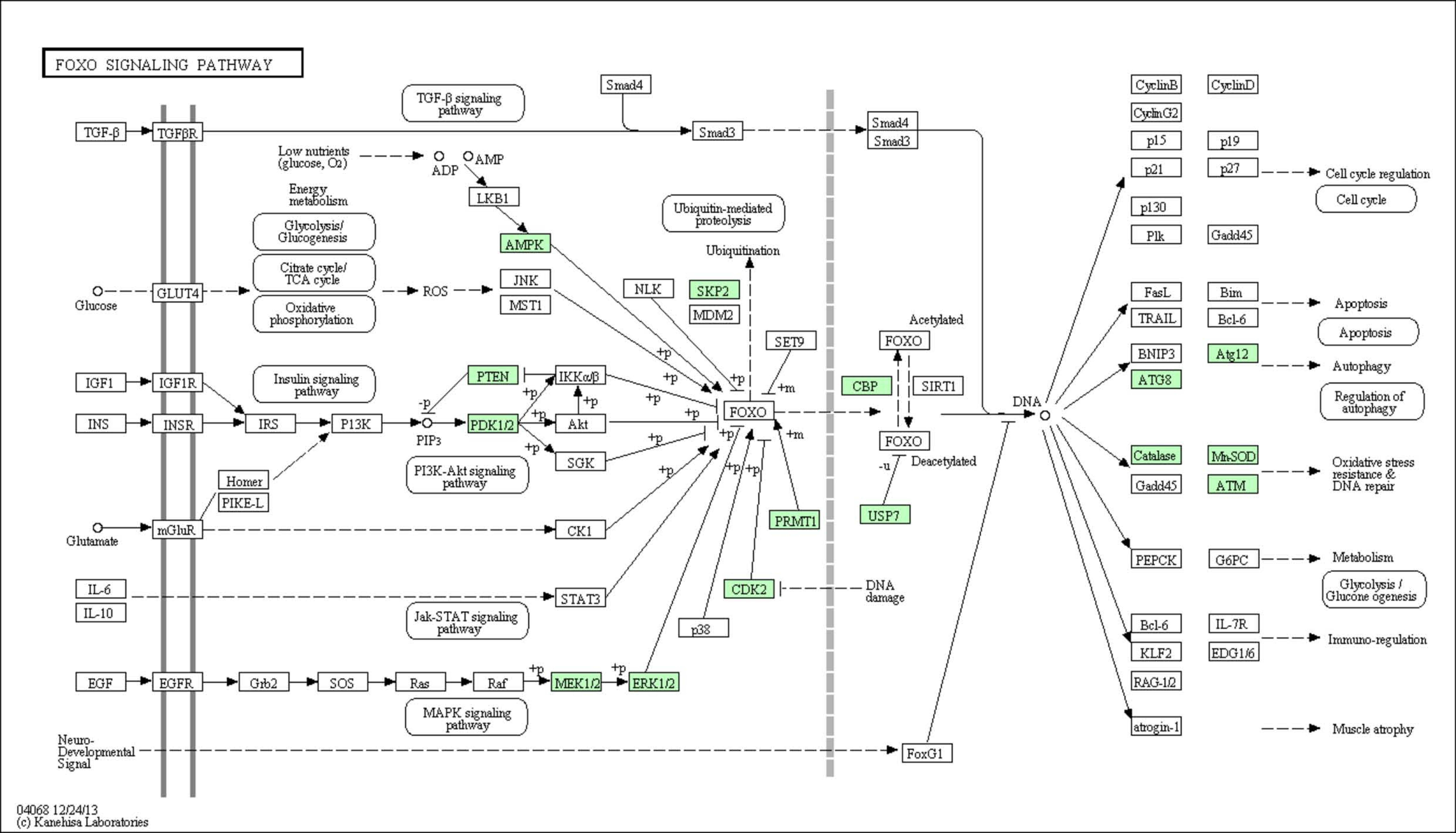
FOXO SIGNALING PATHWAY
TGF-β
TGFβR
TGF-β signaling pathway
Smad4
Smad3
Smad4
Smad3
Low nutrients (glucose, O2)
ADP
AMP
LKB1
Energy metabolism
Glycolysis/ Glucogenesis
Citrate cycle/ TCA cycle
Oxidative phosphorylation
Glucose
GLUT4
ROS
AMPK
JNK
MST1
Ubiquitin-mediated proteolysis
Ubiquitination
NLK
SKP2
MDM2
SET9
IGF1
IGF1R
INS
INSR
IRS
PI3K
Insulin signaling pathway
PTEN
PDK1/2
PIP3
IKKα/β
Akt
SGK
PI3K-Akt signaling pathway
FOXO
+p
+m
-p
-u
Homer
PIKE-L
Glutamate
mGluR
CK1
IL-6
IL-10
STAT3
Jak-STAT signaling pathway
p38
CDK2
PRMT1
DNA damage
EGF
EGFR
Grb2
SOS
Ras
Raf
MEK1/2
ERK1/2
MAPK signaling pathway
Neuro-Developmental Signal
FoxG1
Acetylated
CBP
SIRT1
Deacetylated
USP7
DNA
CyclinB
CyclinD
CyclinG2
p15
p19
p21
p27
p130
Plk
Gadd45
Cell cycle regulation
Cell cycle
FasL
Bim
TRAIL
Bcl-6
Apoptosis
Apoptosis
BNIP3
Atg12
ATG8
Autophagy
Regulation of autophagy
Catalase
MnSOD
Gadd45
ATM
Oxidative stress resistance & DNA repair
PEPCK
G6PC
Metabolism
Glycolysis / Glucone ogenesis
Bcl-6
IL-7R
KLF2
EDG1/6
Immuno-regulation
RAG-1/2
atrogin-1
Muscle atrophy
04068 12/24/13
(c) Kanehisa Laboratories

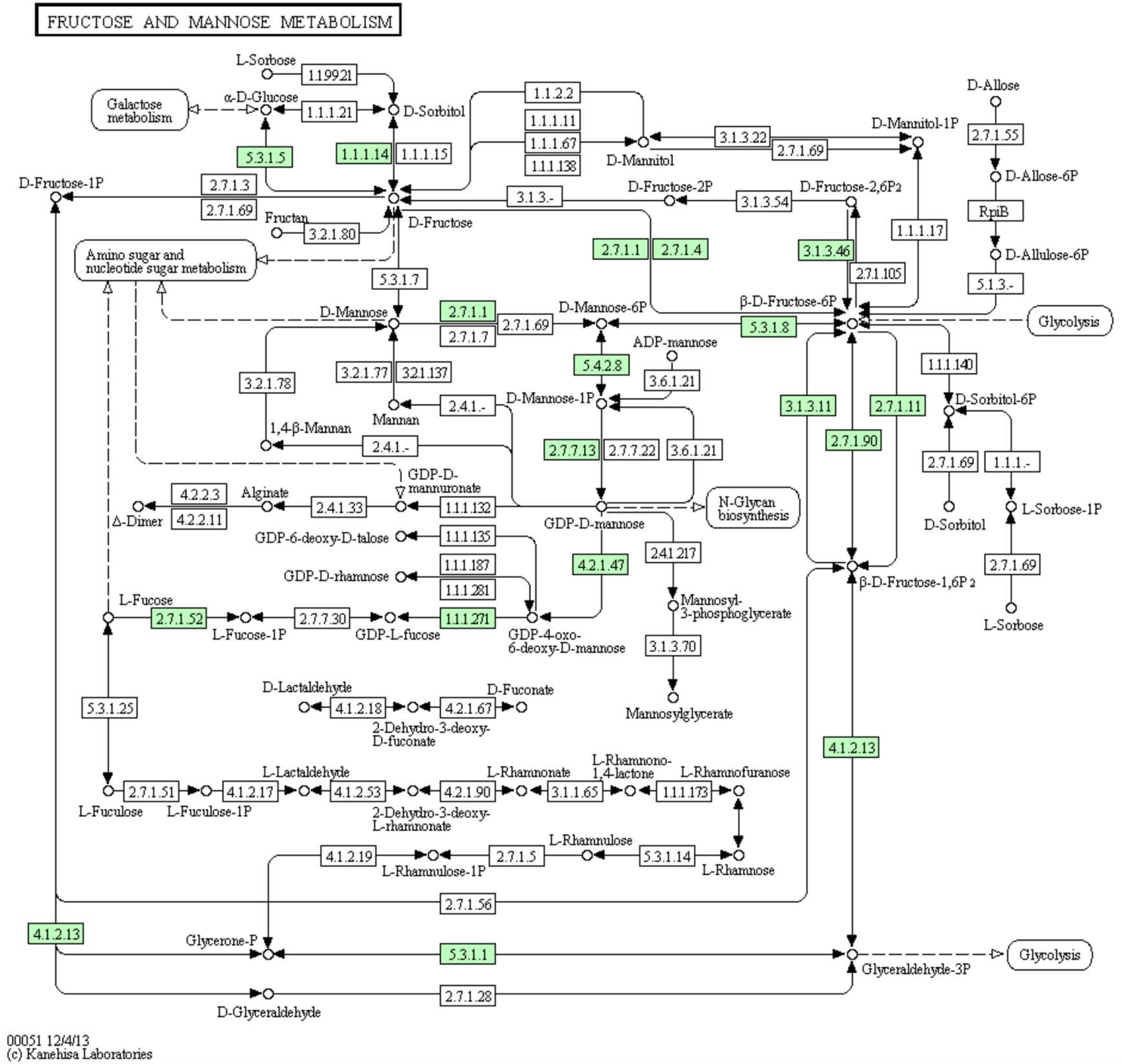
FRUCTOSE AND MANNOSE METABOLISM
L-Sorbose
1.1.99.21
Galactose metabolism
α-D-Glucose
1.1.1.21
D-Sorbitol
1.1.2.2
1.1.1.11
1.1.1.67
1.1.1.138
D-Mannitol
3.1.3.22
2.7.1.69
D-Mannitol-1P
D-Allose
2.7.1.55
D-Allose-6P
RpiB
D-Allulose-6P
5.1.3.-
5.3.1.5
1.1.1.14
1.1.1.15
D-Fructose-1P
2.7.1.3
2.7.1.69
Fructan
3.2.1.80
D-Fructose
3.1.3.-
D-Fructose-2P
3.1.3.54
D-Fructose-2,6P2
1.1.1.17
Amino sugar and nucleotide sugar metabolism
2.7.1.1
2.7.1.4
3.1.3.46
2.7.1.105
5.3.1.7
D-Mannose
2.7.1.1
2.7.1.7
2.7.1.69
D-Mannose-6P
5.3.1.8
β-D-Fructose-6P
Glycolysis
ADP-mannose
5.4.2.8
3.6.1.21
3.2.1.78
3.2.1.77
3.2.1.137
Mannan
2.4.1.-
D-Mannose-1P
1.1.1.140
D-Sorbitol-6P
3.1.3.11
2.7.1.11
2.7.1.90
1,4-β-Mannan
2.4.1.-
2.7.7.13
2.7.7.22
3.6.1.21
2.7.1.69
1.1.1.-
GDP-D-mannuronate
4.2.2.3
4.2.2.11
Alginate
2.4.1.33
Δ-Dimer
1.1.1.132
GDP-D-mannose
N-Glycan biosynthesis
D-Sorbitol
L-Sorbose-1P
GDP-6-deoxy-D-talose
1.1.1.135
1.1.1.187
1.1.1.281
GDP-D-rhamnose
4.2.1.47
2.4.1.217
β-D-Fructose-1,6P2
2.7.1.69
L-Sorbose
L-Fucose
2.7.1.52
L-Fucose-1P
2.7.7.30
GDP-L-fucose
1.1.1.271
GDP-4-oxo-6-deoxy-D-mannose
Mannosyl-3-phosphoglycerate
3.1.3.70
Mannosylglycerate
5.3.1.25
D-Lactaldehyde
4.1.2.18
4.2.1.67
D-Fuconate
2-Dehydro-3-deoxy-D-fuconate
4.1.2.13
L-Lactaldehyde
L-Rhamnonate
L-Rhamnono-1,4-lactone
L-Rhamnofuranose
2.7.1.51
4.1.2.17
4.1.2.53
4.2.1.90
3.1.1.65
1.1.1.173
L-Fuculose
L-Fuculose-1P
2-Dehydro-3-deoxy-L-rhamnonate
L-Rhamnulose
4.1.2.19
2.7.1.5
5.3.1.14
L-Rhamnulose-1P
L-Rhamnose
2.7.1.56
4.1.2.13
Glycerone-P
5.3.1.1
Glyceraldehyde-3P
Glycolysis
2.7.1.28
D-Glyceraldehyde
00051 12/4/13
(c) Kanehisa Laboratories

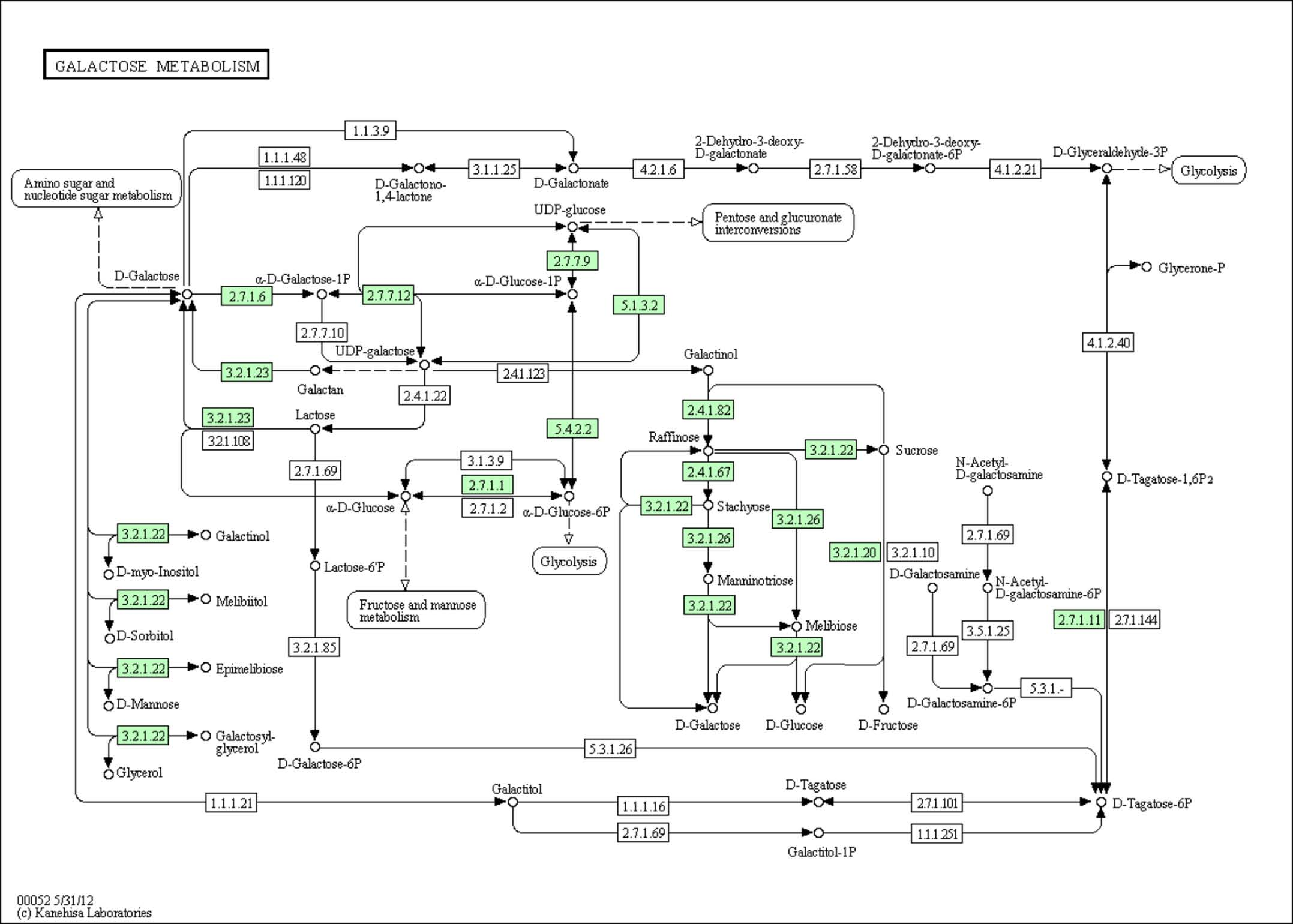
GALACTOSE METABOLISM
1.1.3.9
1.1.1.48
1.1.1.120
3.1.1.25
4.2.1.6
2.7.1.58
4.1.2.21
2-Dehydro-3-deoxy-D-galactonate
2-Dehydro-3-deoxy-D-galactonate-6P
D-Glyceraldehyde-3P
Glycolysis
Amino sugar and nucleotide sugar metabolism
D-Galactono-1,4-lactone
D-Galactonate
UDP-glucose
Pentose and glucuronate interconversions
2.7.7.9
D-Galactose
α-D-Galactose-1P
2.7.1.6
2.7.7.12
α-D-Glucose-1P
5.1.3.2
Glycerone-P
2.7.7.10
UDP-galactose
3.2.1.23
Galactan
2.4.1.123
Galactinol
4.1.2.40
2.4.1.22
3.2.1.23
3.2.1.108
Lactose
5.4.2.2
2.4.1.82
Raffinose
3.2.1.22
Sucrose
3.1.3.9
2.7.1.69
2.7.1.1
2.7.1.2
α-D-Glucose
α-D-Glucose-6P
2.4.1.67
N-Acetyl-D-galactosamine
D-Tagatose-1,6P2
3.2.1.22
Stachyose
3.2.1.26
3.2.1.22
Galactinol
D-myo-Inositol
Glycolysis
3.2.1.26
3.2.1.20
3.2.1.10
2.7.1.69
Lactose-6'P
Mannintriose
D-Galactosamine
N-Acetyl-D-galactosamine-6P
3.2.1.22
Melibiitol
D-Sorbitol
Fructose and mannose metabolism
3.2.1.22
Melibiose
2.7.1.11
2.7.1.144
3.2.1.85
3.2.1.22
3.5.1.25
2.7.1.69
3.2.1.22
Epimelibiose
D-Mannose
5.3.1.-
D-Galactosamine-6P
D-Galactose
D-Glucose
D-Fructose
3.2.1.22
Galactosyl-glycerol
Glycerol
D-Galactose-6P
5.3.1.26
Galactitol
D-Tagatose
1.1.1.21
1.1.1.16
2.7.1.101
D-Tagatose-6P
2.7.1.69
1.1.1.251
Galactitol-1P
00052 5/31/12
(c) Kanehisa Laboratories

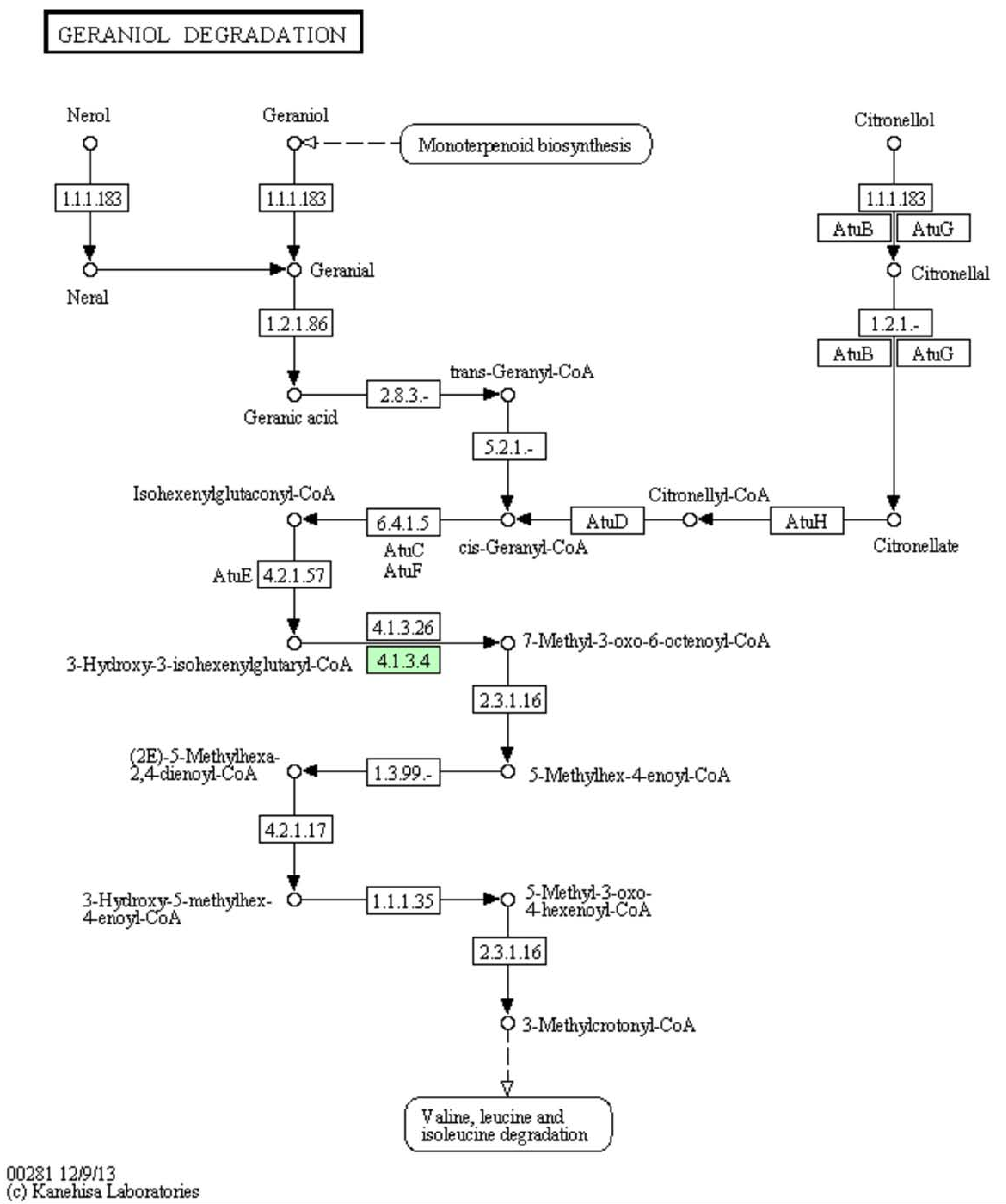
GERANIOL DEGRADATION
Nerol
Geraniol
Monoterpenoid biosynthesis
Citronellol
1.1.1.183
1.1.1.183
1.1.1.183
AtuB
AtuG
Geranial
Neral
Citronellal
1.2.1.86
1.2.1.-
AtuB
AtuG
trans-Geranyl-CoA
2.8.3.-
Geranic acid
5.2.1.-
Isohexenylglutaconyl-CoA
Citronellyl-CoA
6.4.1.5
AtuD
AtuH
AtuC
AtuF
cis-Geranyl-CoA
Citronellate
AtuE
4.2.1.57
4.1.3.26
4.1.3.4
7-Methyl-3-oxo-6-octenoyl-CoA
3-Hydroxy-3-isohexenylglutaryl-CoA
2.3.1.16
(2E)-5-Methylhexa-2,4-dienoyl-CoA
1.3.99.-
5-Methylhex-4-enoyl-CoA
4.2.1.17
3-Hydroxy-5-methylhex-4-enoyl-CoA
1.1.1.35
5-Methyl-3-oxo-4-hexenoyl-CoA
2.3.1.16
3-Methylcrotonyl-CoA
Valine, leucine and isoleucine degradation
00281 12/9/13
(c) Kanehisa Laboratories

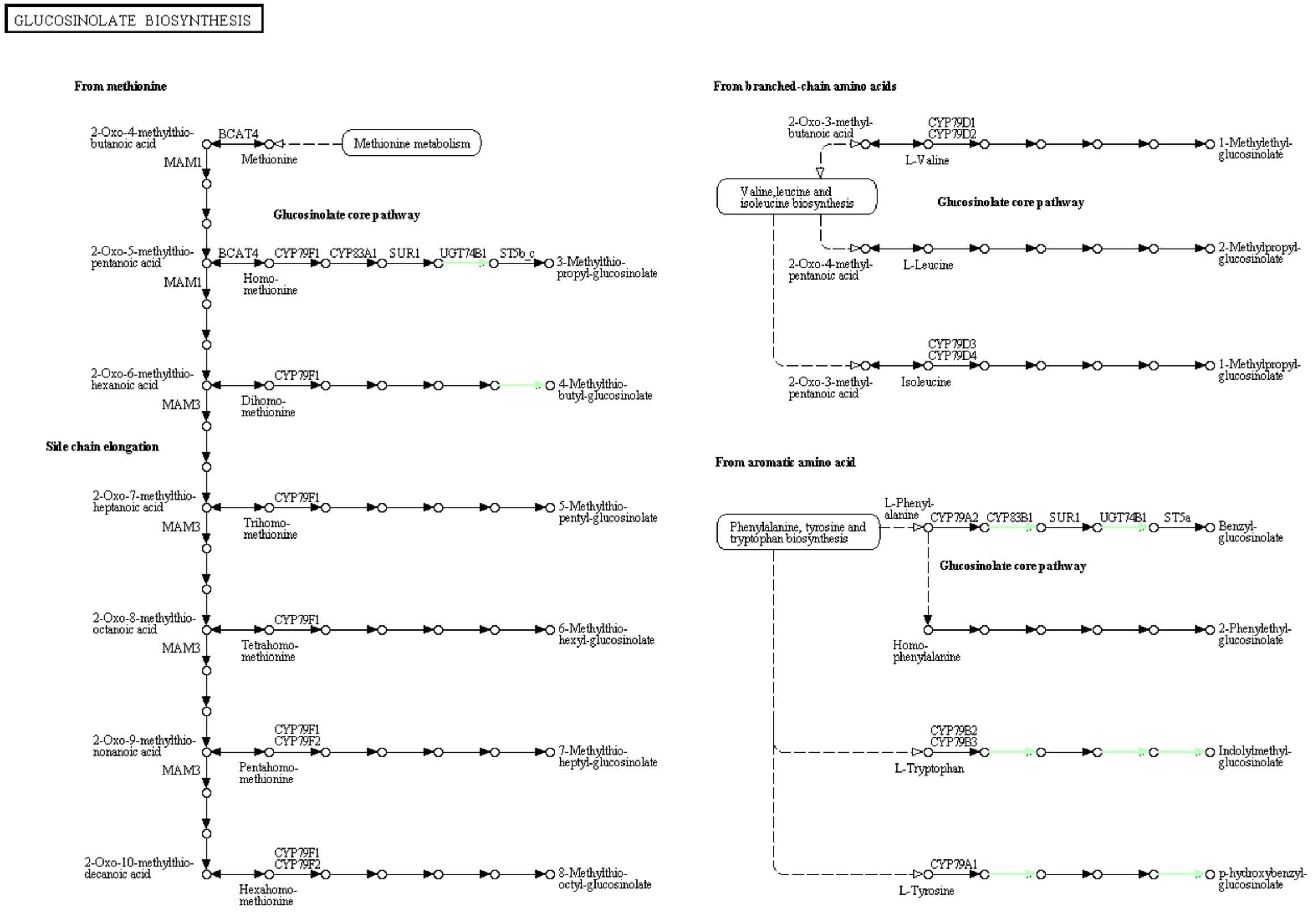

00966 9/3/12
(c) Kanehisa Laboratories

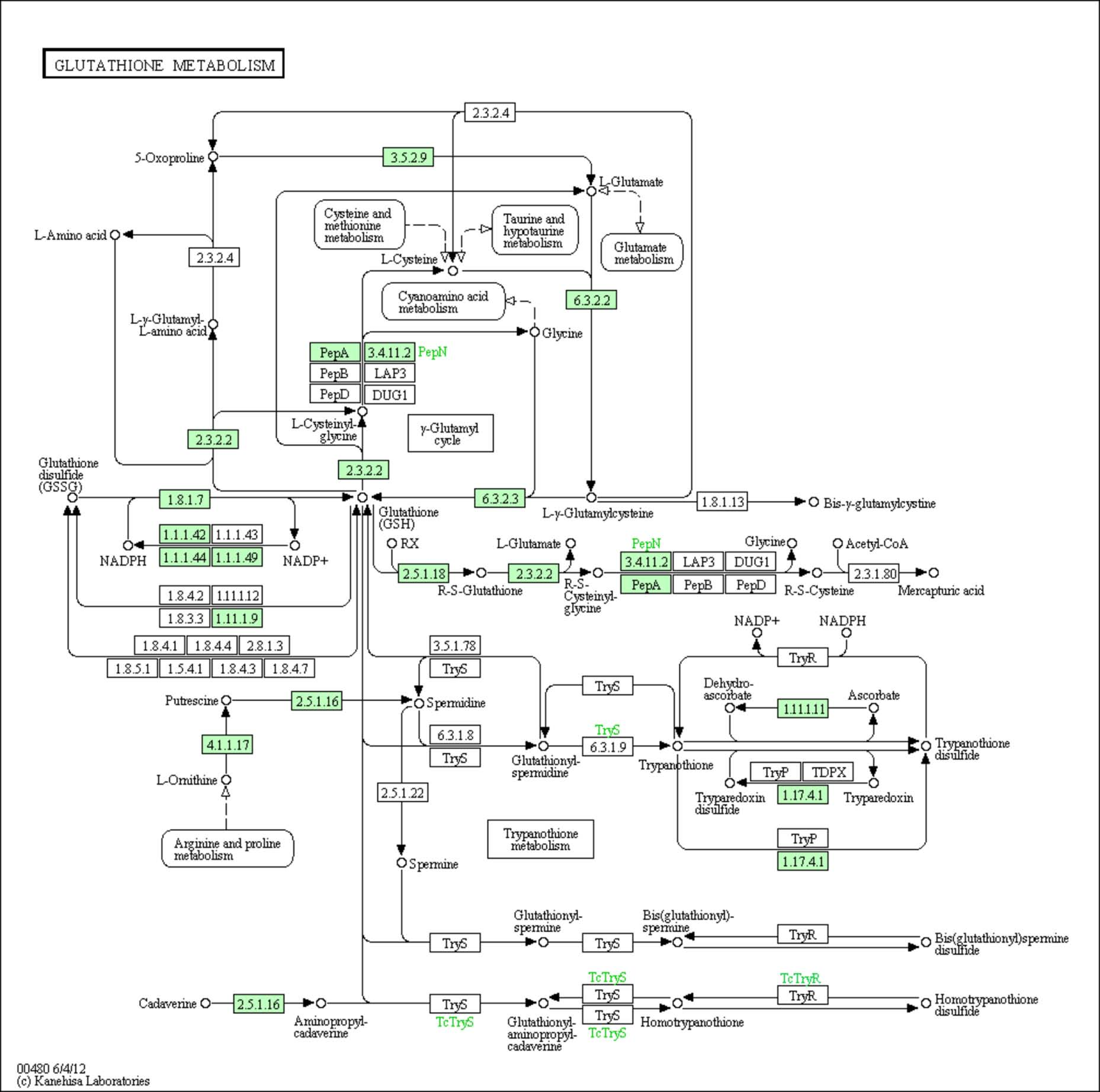

GLUTATHIONE METABOLISM
2.3.2.4
5-Oxoproline
3.5.2.9
L-Glutamate
Cysteine and methionine metabolism
Taurine and hypotaurine metabolism
Glutamate metabolism
L-Amino acid
2.3.2.4
L-Cysteine
Cyanoamino acid metabolism
6.3.2.2
L-γ-Glutamyl-L-amino acid
Glycine
PepA
3.4.11.2
PepN
PepB
LAP3
PepD
DUG1
L-Cysteinyl-glycine
γ-Glutamyl cycle
2.3.2.2
2.3.2.2
Glutathione disulfide (GSSG)
1.8.1.7
Glutathione (GSH)
6.3.2.3
L-γ-Glutamylcysteine
1.8.1.13
Bis-γ-glutamylcystine
1.1.1.42
1.1.1.43
1.1.1.44
1.1.1.49
NADPH
NADP+
RX
L-Glutamate
PepN
Glycine
Acetyl-CoA
2.5.1.18
2.3.2.2
3.4.11.2
LAP3
DUG1
2.3.1.80
PepA
PepB
PepD
R-S-Glutathione
R-S-Cysteinyl-glycine
R-S-Cysteine
Mercapturic acid
1.8.4.2
1.11.1.12
1.8.3.3
1.11.1.9
1.8.4.1
1.8.4.4
2.8.1.3
1.8.5.1
1.5.4.1
1.8.4.3
1.8.4.7
3.5.1.78
TryS
NADP+
NADPH
TryR
Putrescine
2.5.1.16
Spermidine
TryS
Dehydro-ascorbate
Ascorbate
1.11.1.11
4.1.1.17
TryS
6.3.1.8
6.3.1.9
TryS
Glutathionyl-spermidine
Trypanothione
Trypanothione disulfide
L-Ornithine
TryP
TDPX
2.5.1.22
Tryparedoxin disulfide
1.17.4.1
Tryparedoxin
Arginine and proline metabolism
Trypanothione metabolism
TryP
1.17.4.1
Spermine
Glutathionyl-spermine
Bis(glutathionyl)-spermine
TryS
TryS
TryR
Bis(glutathionyl)spermine disulfide
TcTryS
TcTryR
Cadaverine
2.5.1.16
TryS
TryS
TryR
Homotrypanothione disulfide
Aminopropyl-cadaverine
TcTryS
TryS
Glutathionyl-aminopropyl-cadaverine
TcTryS
Homotrypanothione
00480 6/4/12
(c) Kanehisa Laboratories

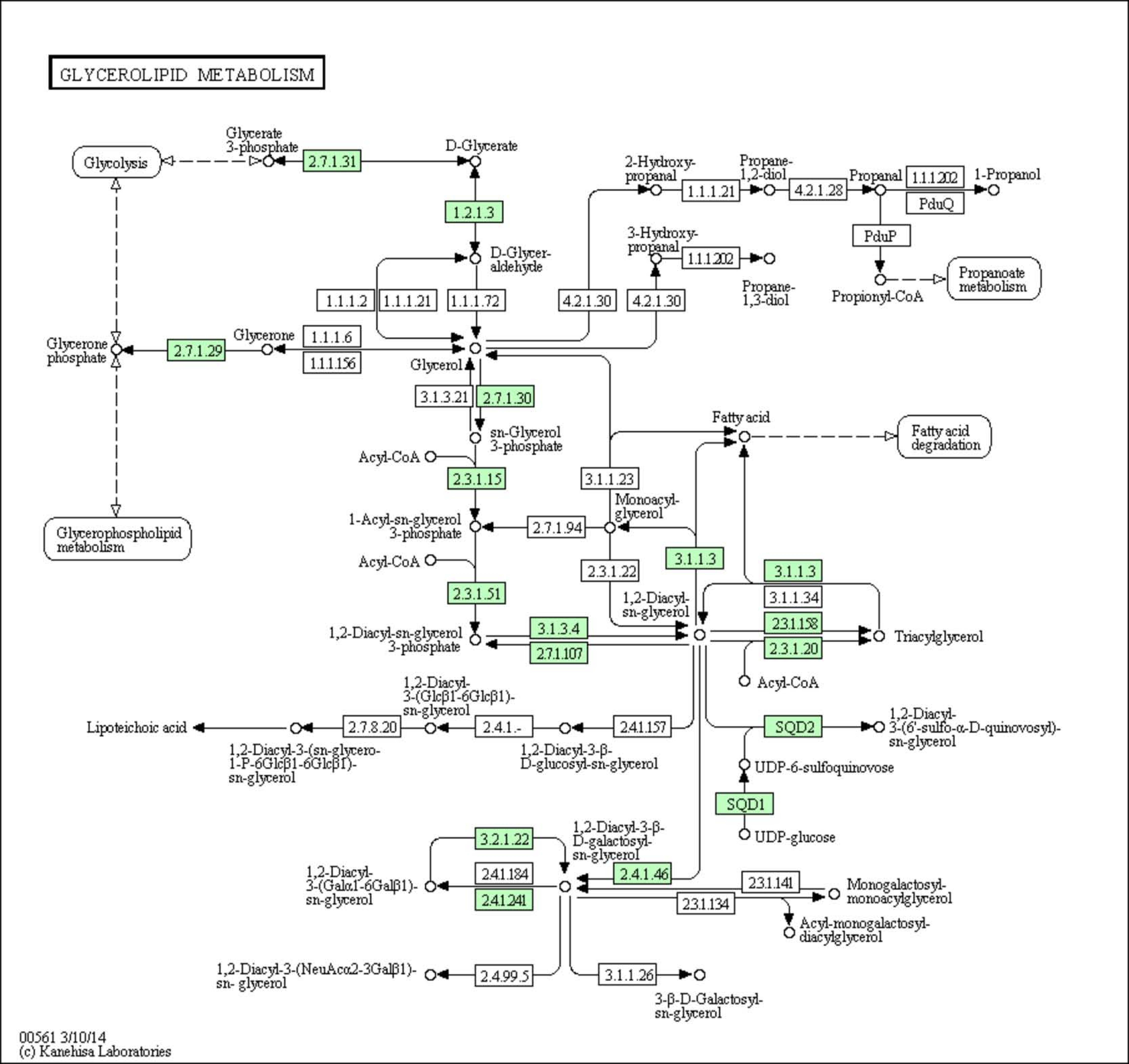
GLYCEROLIPID METABOLISM
Glycolysis
Glycerate 3-phosphate
2.7.1.31
D-Glycerate
1.2.1.3
D-Glyceraldehyde
1.1.1.2
1.1.1.21
1.1.1.72
Glycerone phosphate
2.7.1.29
Glycerone
1.1.1.6
1.1.1.156
Glycerol
3.1.3.21
2.7.1.30
sn-Glycerol 3-phosphate
Acyl-CoA
2.3.1.15
1-Acyl-sn-glycerol 3-phosphate
2.7.1.94
Monoacylglycerol
Acyl-CoA
2.3.1.51
1,2-Diacyl-sn-glycerol 3-phosphate
3.1.3.4
2.7.1.107
Glycerophospholipid metabolism
2-Hydroxypropanal
1.1.1.21
Propane-1,2-diol
4.2.1.28
Propanal
1.1.1.202
PduQ
1-Propanol
PduP
Propionyl-CoA
Propanoate metabolism
3-Hydroxypropanal
1.1.1.202
Propane-1,3-diol
4.2.1.30
4.2.1.30
Fatty acid
Fatty acid degradation
3.1.1.23
2.3.1.22
1,2-Diacyl-sn-glycerol
3.1.1.3
3.1.1.3
3.1.1.34
2.3.1.158
2.3.1.20
Triacylglycerol
Acyl-CoA
Lipoteichoic acid
1,2-Diacyl-3-(sn-glycero-1-P-6Glcβ1-6Glcβ1)-sn-glycerol
2.7.8.20
1,2-Diacyl-3-(Glcβ1-6Glcβ1)-sn-glycerol
2.4.1.-
1,2-Diacyl-3-β-D-glucosyl-sn-glycerol
2.4.1.157
SQD2
1,2-Diacyl-3-(6'-sulfo-α-D-quinovosyl)-sn-glycerol
UDP-6-sulfoquinovose
SQD1
UDP-glucose
3.2.1.22
1,2-Diacyl-3-(Galα1-6Galβ1)-sn-glycerol
2.4.1.184
2.4.1.241
1,2-Diacyl-3-β-D-galactosyl-sn-glycerol
2.4.1.46
2.3.1.141
Monogalactosylmonoacylglycerol
2.3.1.134
Acyl-monogalactosyl-diacylglycerol
1,2-Diacyl-3-(NeuAcα2-3Galβ1)-sn-glycerol
2.4.99.5
3.1.1.26
3-β-D-Galactosyl-sn-glycerol
00561 3/10/14
(c) Kanehisa Laboratories

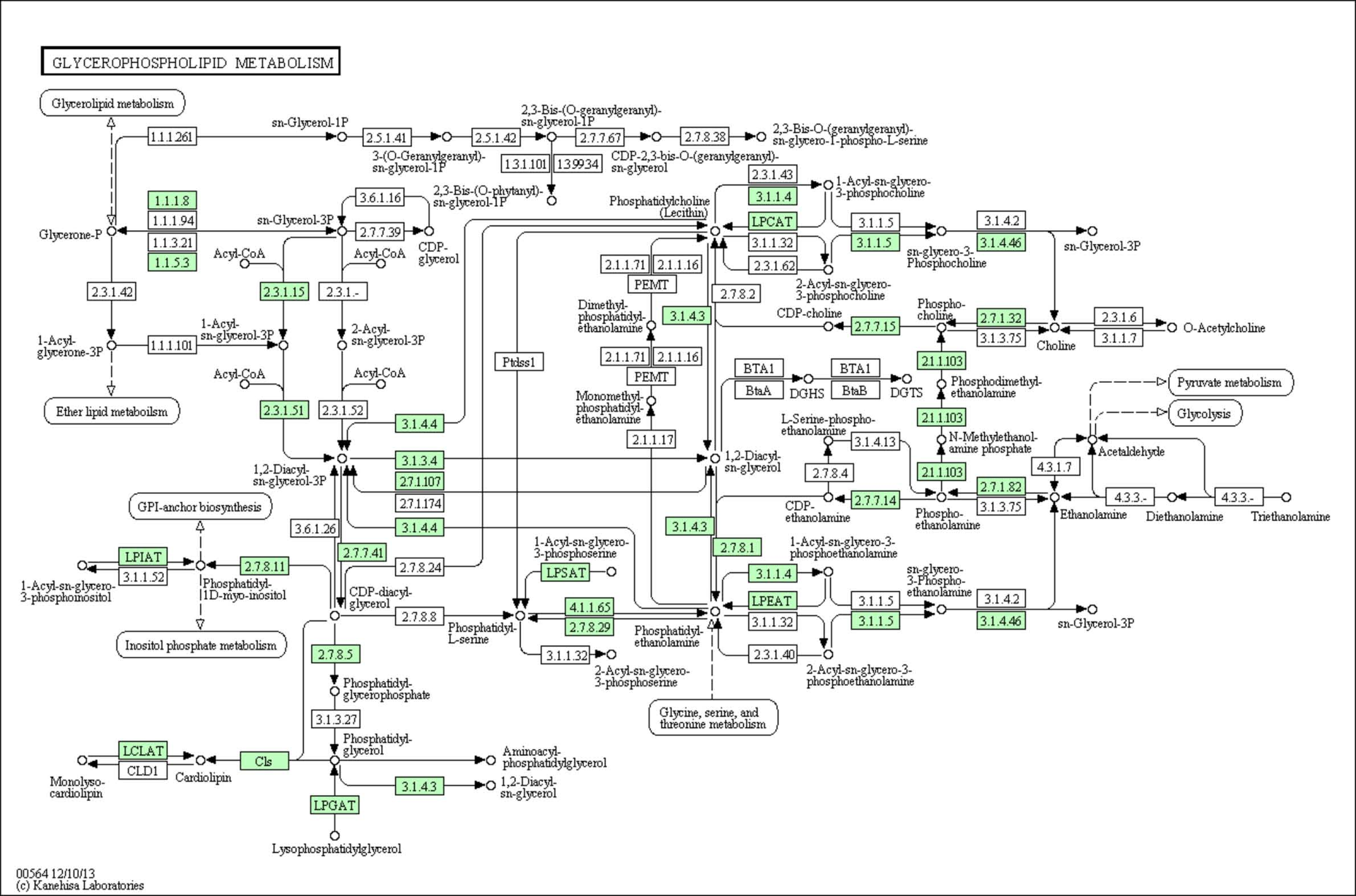
GLYCEROPHOSPHOLIPID METABOLISM
Glycerolipid metabolism
Ether lipid metaboilsm
GPI-anchor biosynthesis
Inositol phosphate metabolism
Glycine, serine, and threonine metabolism
Pyruvate metabolism
Glycolysis
Glycerone-P
1-Acyl-glycerone-3P
sn-Glycerol-1P
3-(O-Geranylgeranyl)-sn-glycerol-1P
2,3-Bis-(O-geranylgeranyl)-sn-glycerol-1P
CDP-2,3-bis-O-(geranylgeranyl)-sn-glycerol
2,3-Bis-O-(geranylgeranyl)-sn-glycero-1-phospho-L-serine
2,3-Bis-(O-phytanyl)-sn-glycerol-1P
sn-Glycerol-3P
Acyl-CoA
1-Acyl-sn-glycerol-3P
2-Acyl-sn-glycerol-3P
CDP-glycerol
1,2-Diacyl-sn-glycerol-3P
Phosphatidylcholine (Lecithin)
1-Acyl-sn-glycero-3-phosphocholine
2-Acyl-sn-glycero-3-phosphocholine
sn-glycero-3-Phosphocholine
Dimethyl-phosphatidyl-ethanolamine
Monomethyl-phosphatidyl-ethanolamine
CDP-choline
Phospho-choline
Choline
O-Acetylcholine
Phosphodimethyl-ethanolamine
N-Methylethanol-amine phosphate
DGHS
DGTS
L-Serine-phospho-ethanolamine
1,2-Diacyl-sn-glycerol
CDP-ethanolamine
Phospho-ethanolamine
Ethanolamine
Diethanolamine
Triethanolamine
Acetaldehyde
1-Acyl-sn-glycero-3-phosphoserine
2-Acyl-sn-glycero-3-phosphoserine
Phosphatidyl-L-serine
Phosphatidyl-ethanolamine
1-Acyl-sn-glycero-3-phosphoethanolamine
2-Acyl-sn-glycero-3-phosphoethanolamine
sn-glycero-3-Phospho-ethanolamine
1-Acyl-sn-glycero-3-phosphoinositol
Phosphatidyl-1D-myo-inositol
CDP-diacyl-glycerol
Phosphatidyl-glycerophosphate
Phosphatidyl-glycerol
Aminoacyl-phosphatidylglycerol
1,2-Diacyl-sn-glycerol
Lysophosphatidylglycerol
Cardiolipin
Monolyso-cardiolipin
1.1.1.261
2.5.1.41
2.5.1.42
2.7.7.67
2.7.8.38
1.3.1.101
1.3.99.34
1.1.1.8
1.1.1.94
1.1.3.21
1.1.5.3
3.6.1.16
2.7.7.39
2.3.1.42
2.3.1.15
2.3.1.-
1.1.1.101
2.3.1.51
2.3.1.52
3.1.4.4
3.1.3.4
2.7.1.107
2.7.1.174
3.6.1.26
2.7.7.41
2.7.8.24
Ptdss1
2.3.1.43
3.1.1.4
LPCAT
3.1.1.32
2.3.1.62
3.1.1.5
3.1.4.2
3.1.4.46
2.1.1.71
2.1.1.16
PEMT
2.7.8.2
3.1.4.3
2.7.7.15
2.7.1.32
3.1.3.75
2.3.1.6
3.1.1.7
2.1.1.17
BTA1
BtaA
BtaB
2.1.1.103
3.1.4.13
2.7.8.4
4.3.1.7
4.3.3.-
2.7.1.82
2.7.7.14
2.7.8.1
LPSAT
LPEAT
2.3.1.40
4.1.1.65
2.7.8.29
LPIAT
3.1.1.52
2.7.8.11
2.7.8.8
2.7.8.5
3.1.3.27
LCLAT
CLD1
Cls
LPGAT
00564 12/10/13
(c) Kanehisa Laboratories

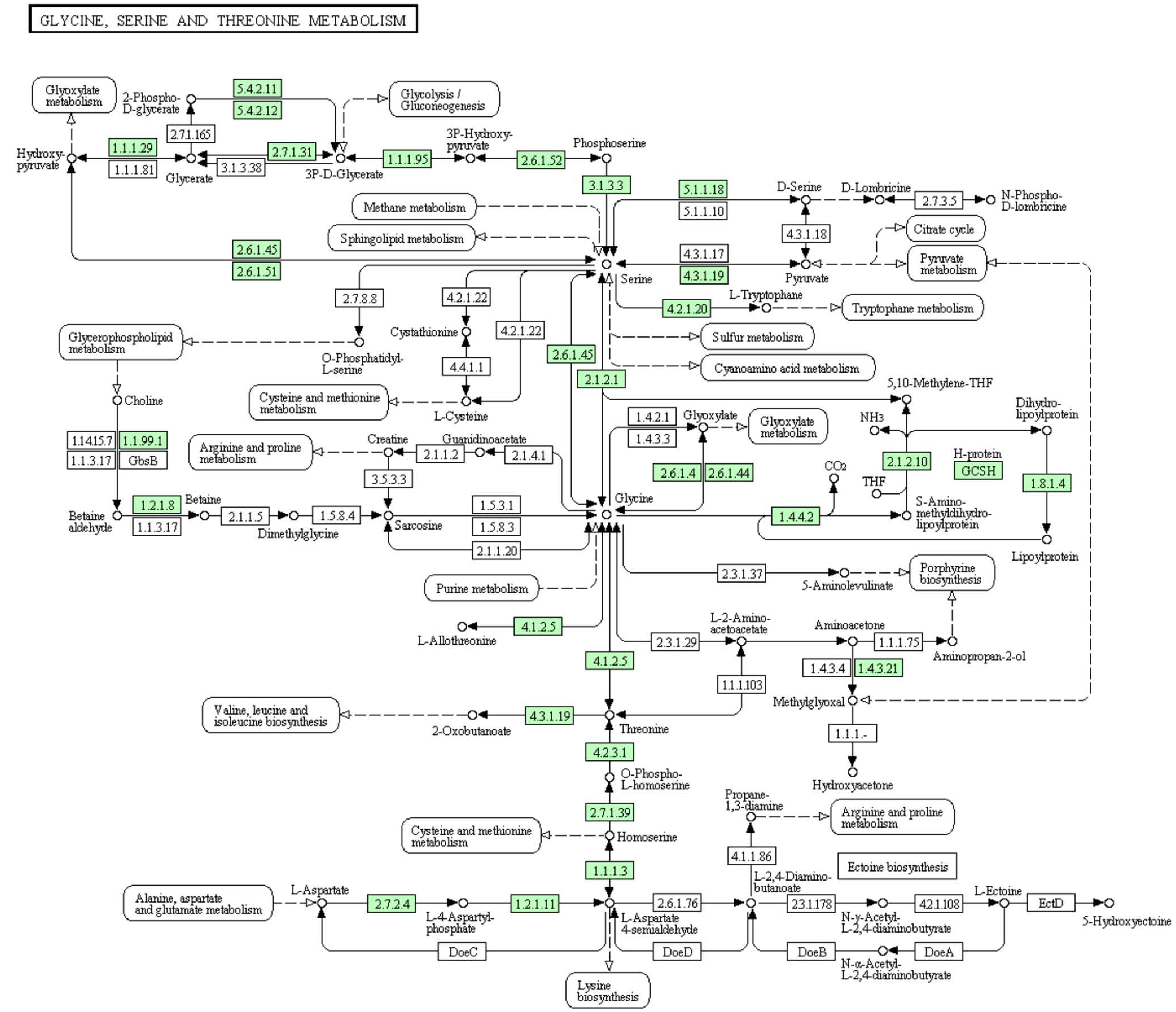

00260 3/6/14

(c) Kanehisa Laboratories

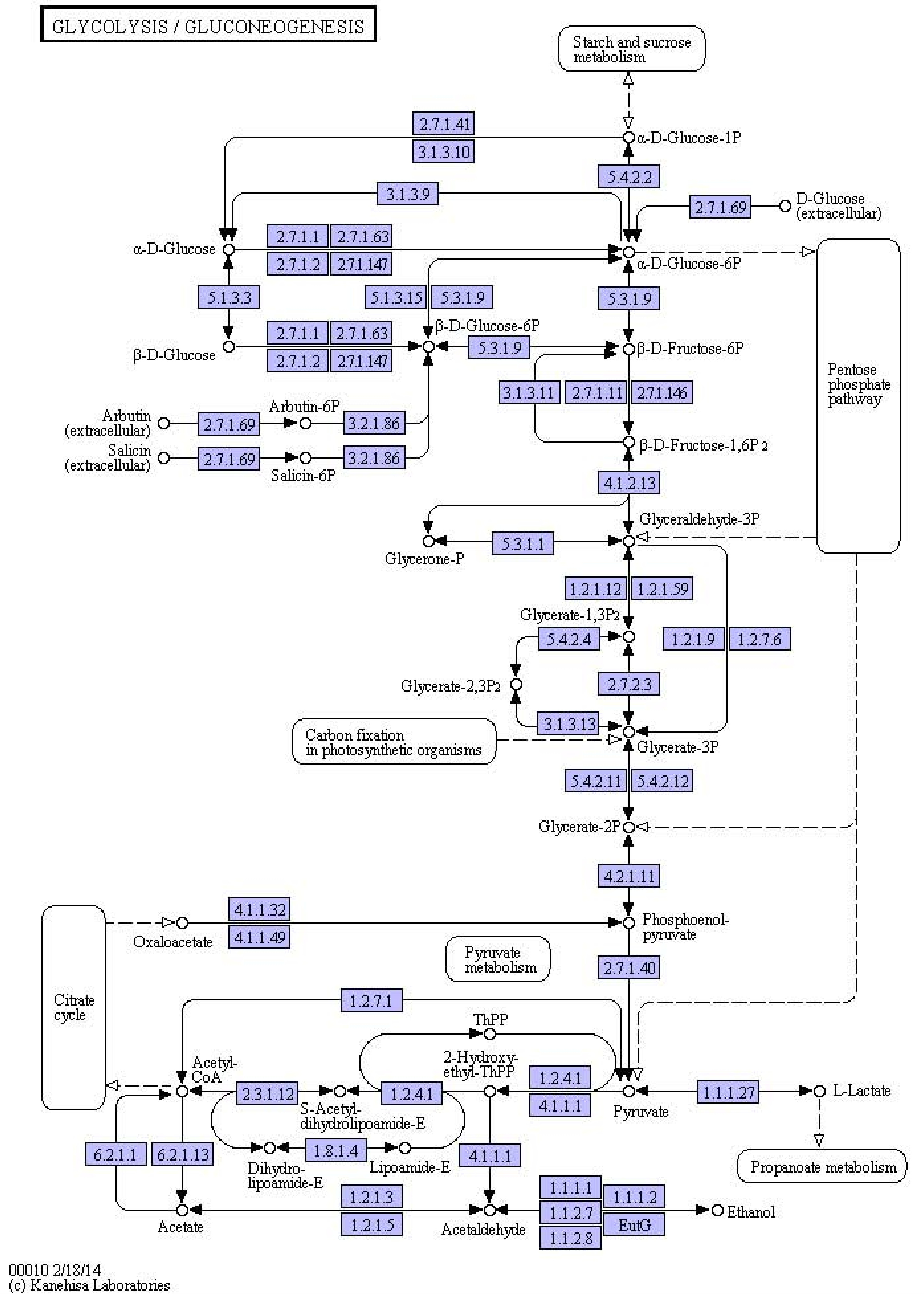
GLYCOLYSIS / GLUCONEOGENESIS
Starch and sucrose metabolism
2.7.1.41
3.1.3.10
α-D-Glucose-1P
5.4.2.2
3.1.3.9
2.7.1.69
D-Glucose (extracellular)
α-D-Glucose
2.7.1.1
2.7.1.63
2.7.1.2
2.7.1.147
α-D-Glucose-6P
5.1.3.3
5.1.3.15
5.3.1.9
5.3.1.9
β-D-Glucose-6P
β-D-Glucose
2.7.1.1
2.7.1.63
2.7.1.2
2.7.1.147
5.3.1.9
β-D-Fructose-6P
Pentose phosphate pathway
3.1.3.11
2.7.1.11
2.7.1.146
Arbutin (extracellular)
2.7.1.69
Arbutin-6P
3.2.1.86
Salicin (extracellular)
2.7.1.69
Salicin-6P
3.2.1.86
β-D-Fructose-1,6P2
4.1.2.13
Glyceraldehyde-3P
Glycerone-P
5.3.1.1
1.2.1.12
1.2.1.59
Glycerate-1,3P2
5.4.2.4
1.2.1.9
1.2.7.6
Glycerate-2,3P2
2.7.2.3
3.1.3.13
Carbon fixation in photosynthetic organisms
Glycerate-3P
5.4.2.11
5.4.2.12
Glycerate-2P
4.2.1.11
Oxaloacetate
4.1.1.32
4.1.1.49
Phosphoenol-pyruvate
Citrate cycle
Pyruvate metabolism
2.7.1.40
1.2.7.1
ThPP
2-Hydroxy-ethyl-ThPP
Acetyl-CoA
2.3.1.12
S-Acetyl-dihydrolipoamide-E
1.2.4.1
1.2.4.1
4.1.1.1
Pyruvate
1.1.1.27
L-Lactate
Propanoate metabolism
6.2.1.1
6.2.1.13
Dihydro-lipoamide-E
1.8.1.4
Lipoamide-E
4.1.1.1
Acetate
1.2.1.3
1.2.1.5
Acetaldehyde
1.1.1.1
1.1.1.2
1.1.2.7
EutG
1.1.2.8
Ethanol
00010 2/18/14
(c) Kanehisa Laboratories

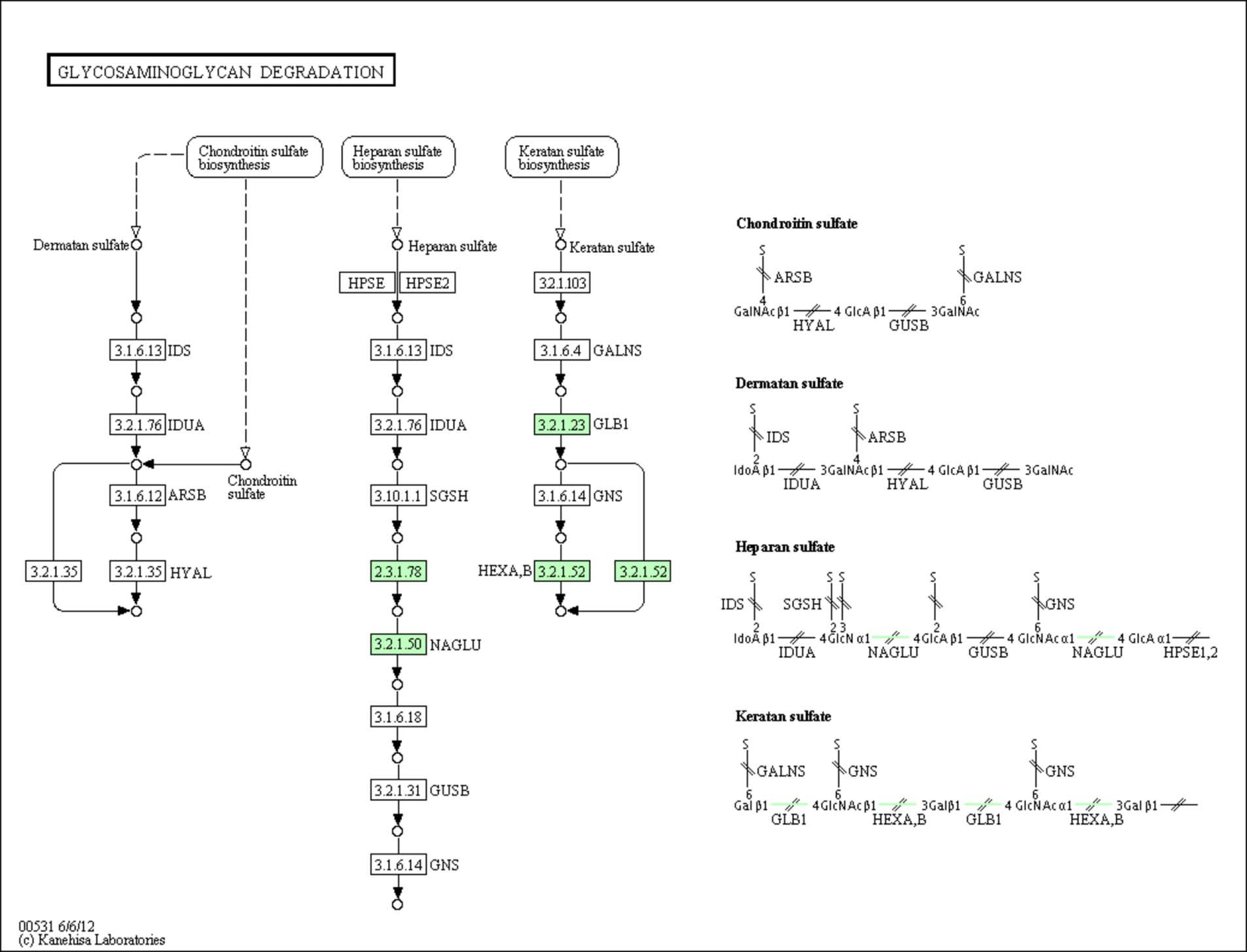

GLYCOSAMINOGLYCAN DEGRADATION
Chondroitin sulfate biosynthesis
Heparan sulfate biosynthesis
Keratan sulfate biosynthesis
Dermatan sulfate
Heparan sulfate
Keratan sulfate
Chondroitin sulfate
HPSE
HPSE2
3.2.1.103
3.1.6.13 IDS
3.1.6.4 GALNS
3.2.1.76 IDUA
3.2.1.23 GLB1
3.1.6.12 ARSB
3.10.1.1 SGSH
3.1.6.14 GNS
3.2.1.35
3.2.1.35 HYAL
2.3.1.78
HEXA,B 3.2.1.52
3.2.1.52
3.2.1.50 NAGLU
3.1.6.18
3.2.1.31 GUSB
Chondroitin sulfate
ARSB
GALNS
GalNAc β1 4 GlcA β1 3GalNAc
HYAL
GUSB
Dermatan sulfate
IDS
ARSB
IdoA β1 3GalNAc β1 4 GlcA β1 3GalNAc
IDUA
HYAL
GUSB
Heparan sulfate
IDS
SGSH
GNS
IdoA β1 4GlcN α1 4GlcA β1 4 GlcNAc α1 4 GlcA α1
IDUA
NAGLU
GUSB
NAGLU
HPSE1,2
Keratan sulfate
GALNS
GNS
GNS
Gal β1 4GlcNAc β1 3Gal β1 4 GlcNAc α1 3Gal β1
GLB1
HEXA,B
GLB1
HEXA,B
00531 6/6/12
(c) Kanehisa Laboratories

GLYCOSPHINGOLIPID BIOSYNTHESIS - GANGLIO SERIES

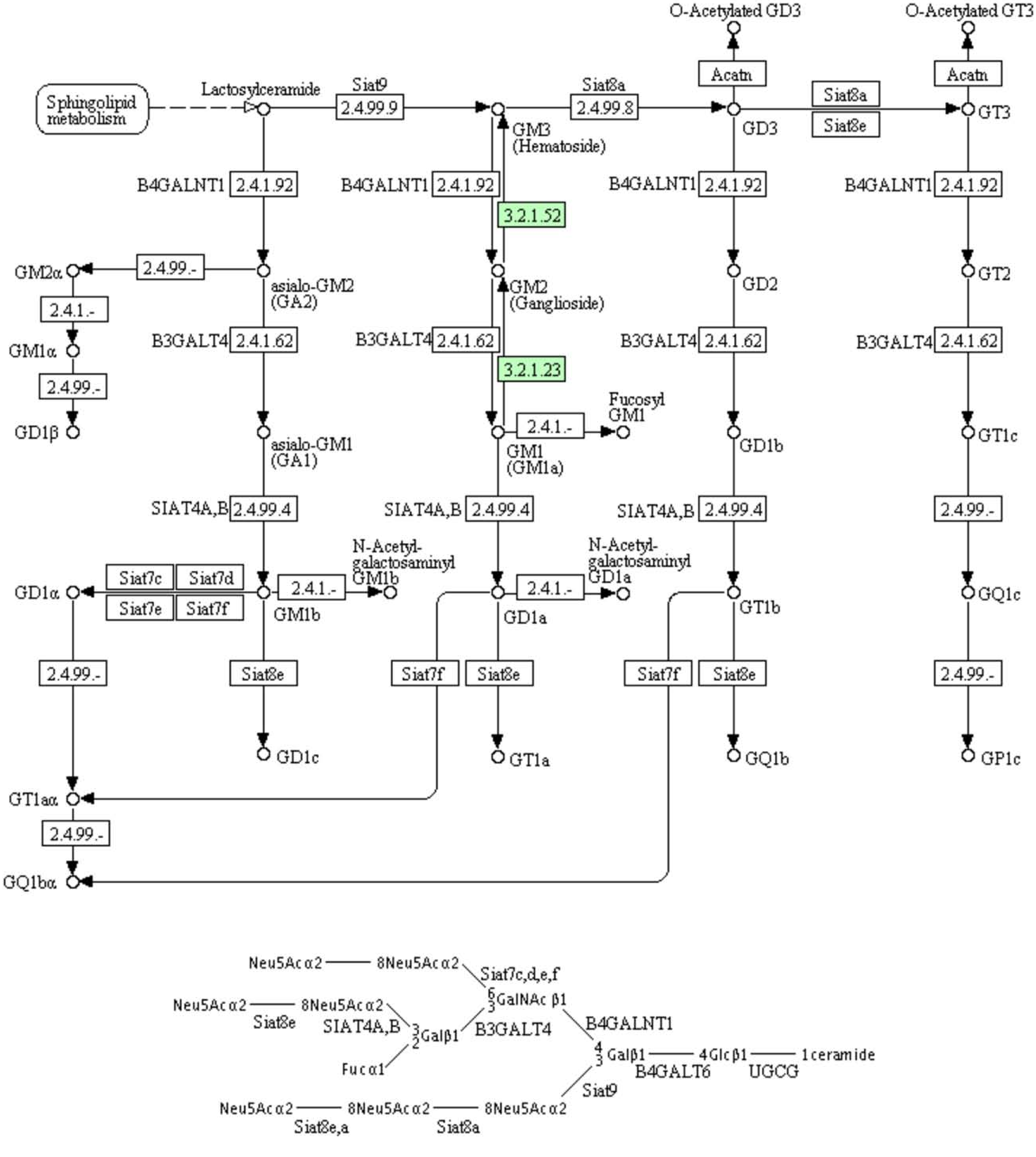

00604 7/2/12

(c) Kanehisa Laboratories

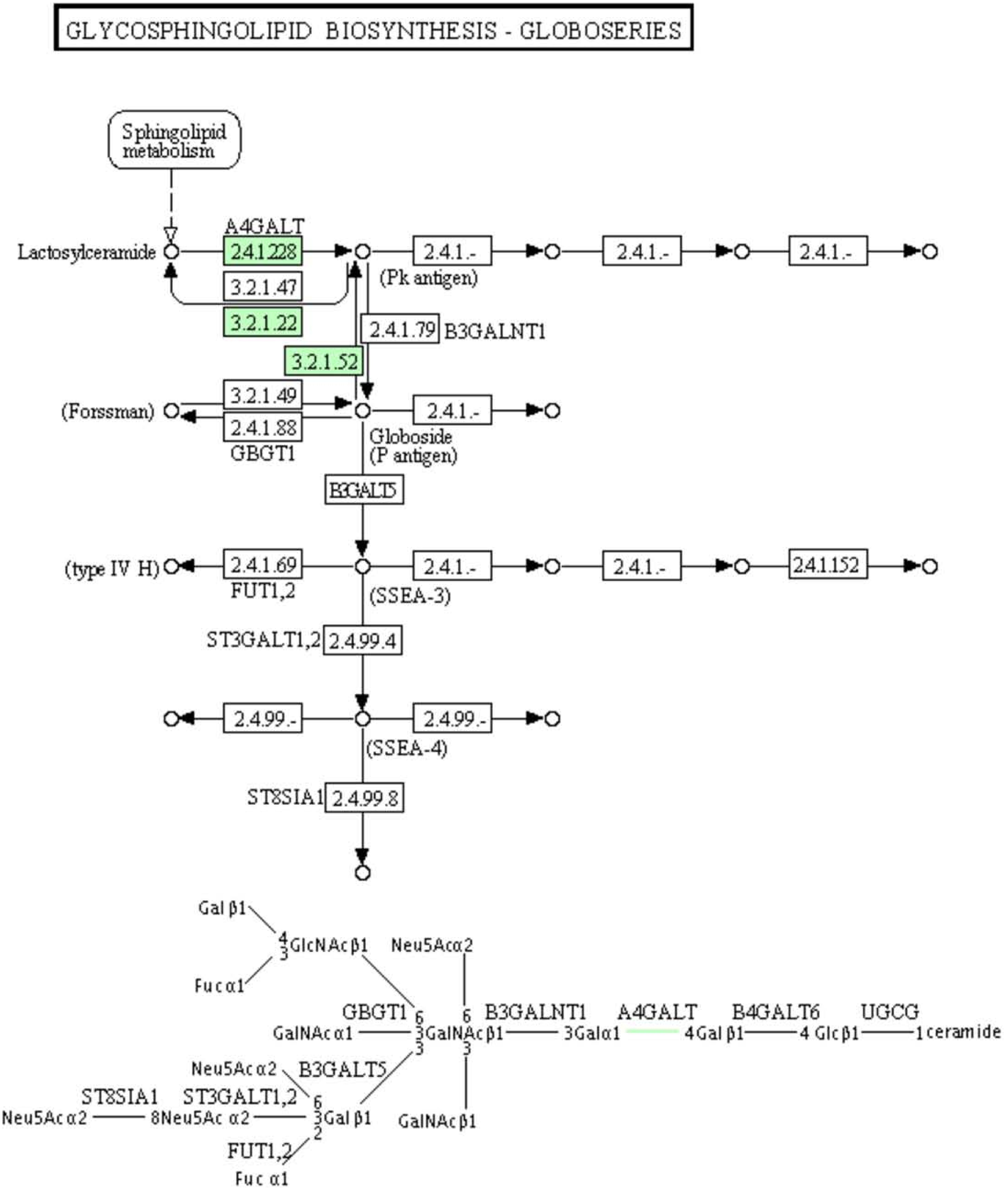

00603 6/6/12
(c) Kanehisa Laboratories

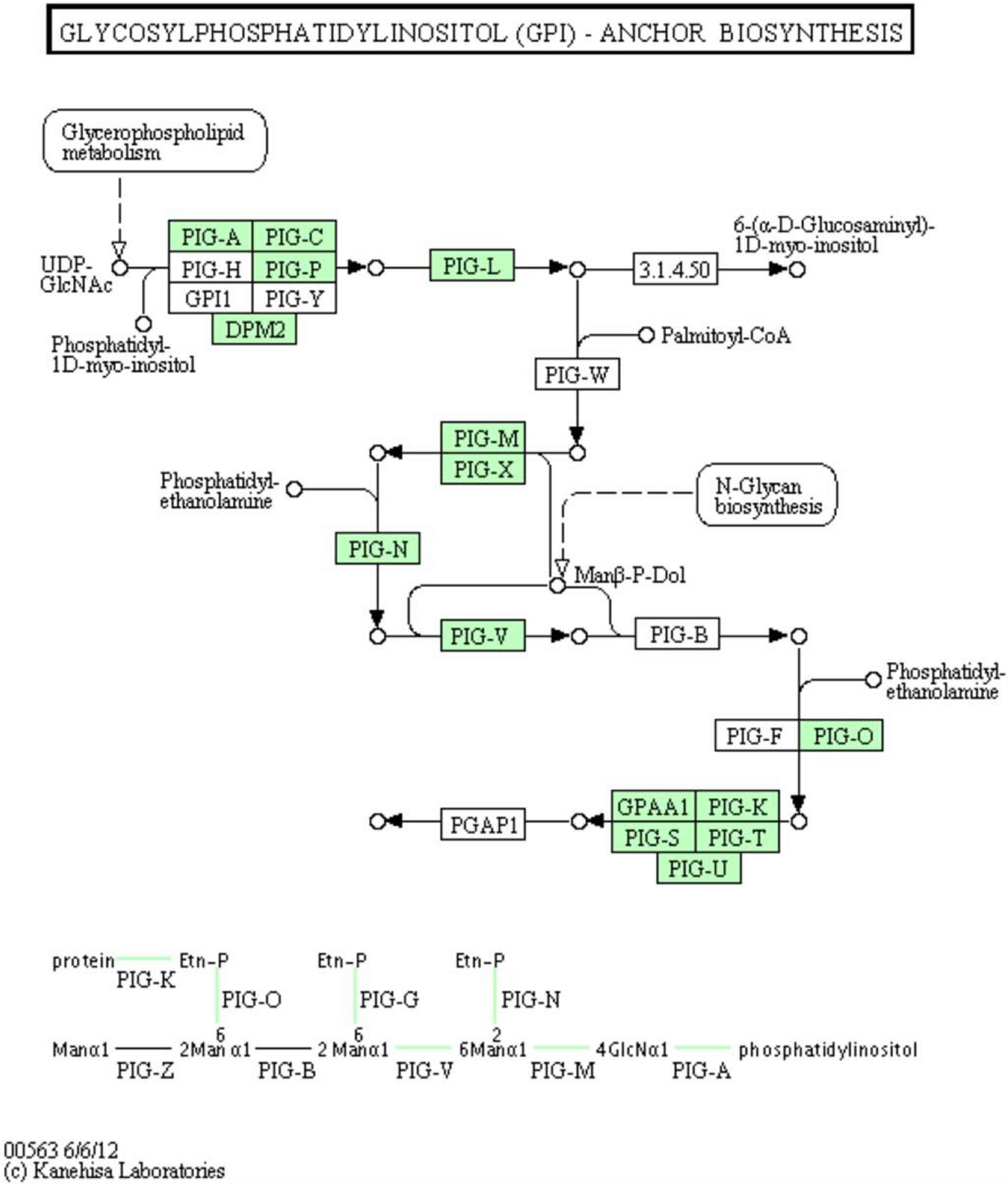
GLYCOSYLPHOSPHATIDYLINOSITOL (GPI) - ANCHOR BIOSYNTHESIS
Glycerophospholipid metabolism
UDP-GlcNAc
Phosphatidyl-1D-myo-inositol
PIG-A
PIG-C
PIG-H
PIG-P
GPI1
PIG-Y
DPM2
PIG-L
3.1.4.50
6-(α-D-Glucosaminyl)-1D-myo-inositol
Palmitoyl-CoA
PIG-W
PIG-M
PIG-X
Phosphatidyl-ethanolamine
N-Glycan biosynthesis
PIG-N
Manβ-P-Dol
PIG-V
PIG-B
Phosphatidyl-ethanolamine
PIG-F
PIG-O
GPAA1
PIG-K
PIG-S
PIG-T
PIG-U
PGAP1
protein
PIG-K
Etn-P
PIG-O
Etn-P
PIG-G
Etn-P
PIG-N
6
6
2
Manα1
2Man α1
2 Manα1
6Manα1
4GlcNα1
phosphatidylinositol
PIG-Z
PIG-B
PIG-V
PIG-M
PIG-A
00563 6/6/12
(c) Kanehisa Laboratories

GLYOXYLATE AND DICARBOXYLATE METABOLISM

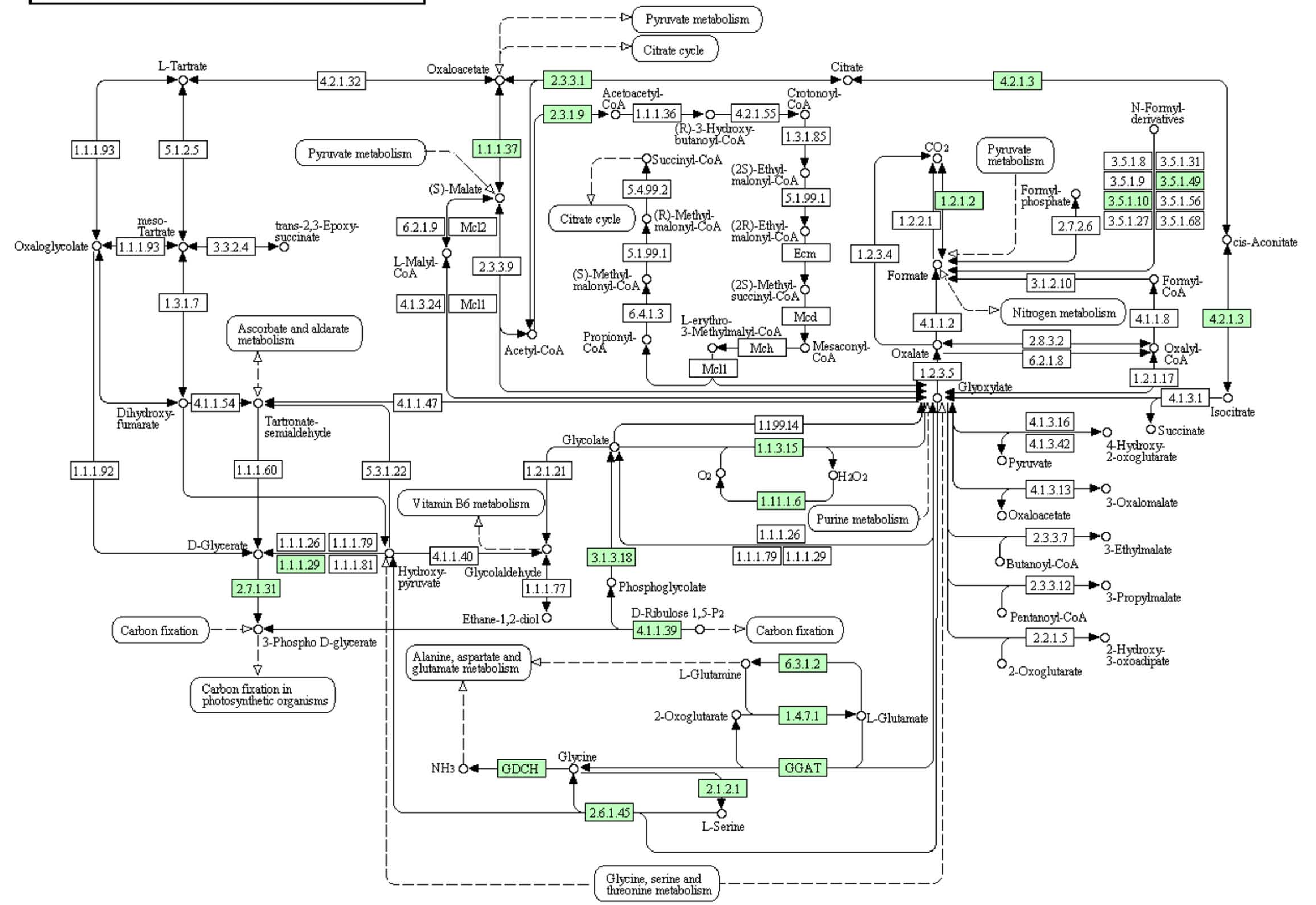

00630 3/14/14

(c) Kanehisa Laboratories

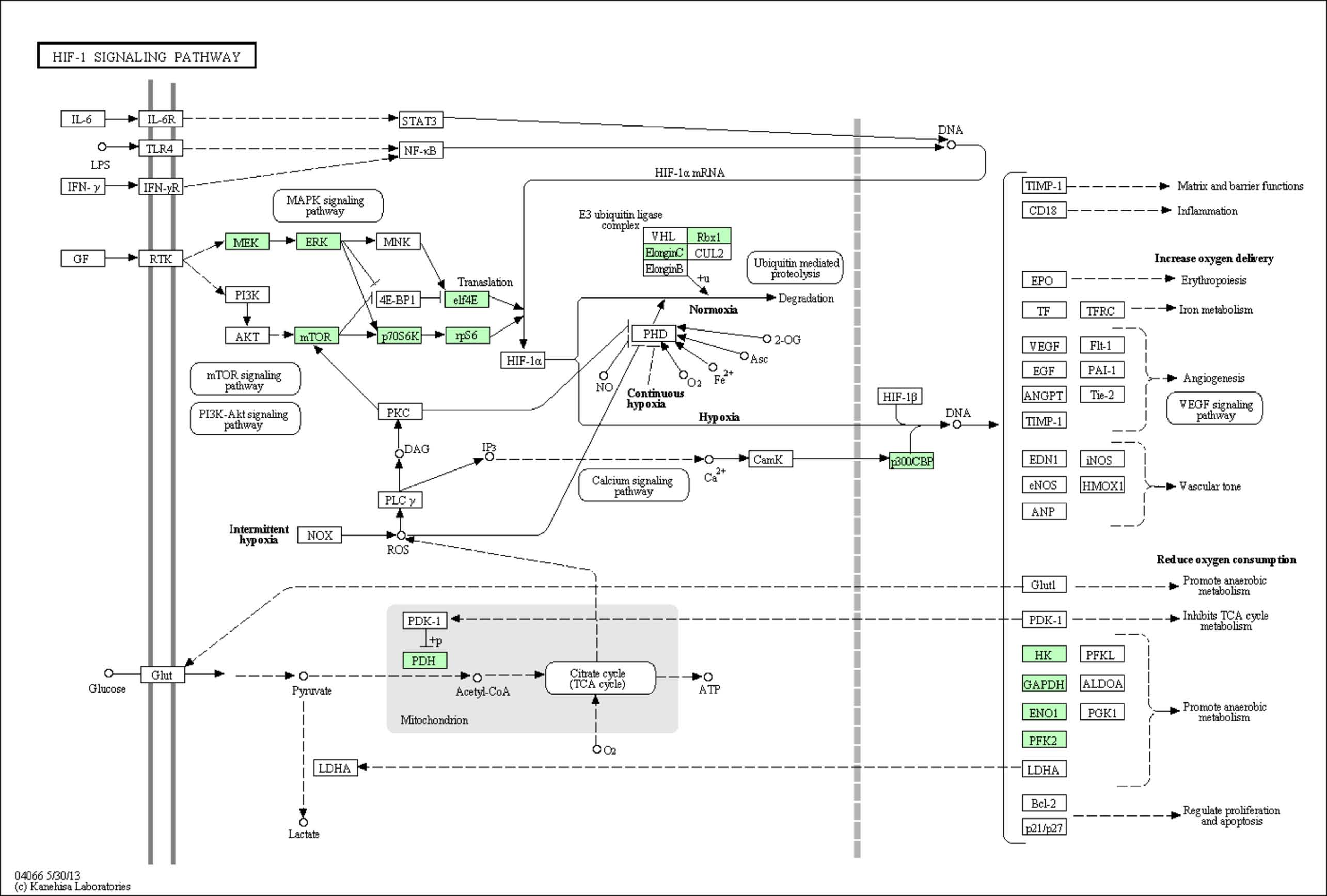
HIF-1 SIGNALING PATHWAY
IL-6
IL-6R
STAT3
LPS
TLR4
NF-κB
IFN-γ
IFN-γR
DNA
HIF-1α mRNA
MAPK signaling pathway
E3 ubiquitin ligase complex
VHL
Rbx1
ElonginC
CUL2
ElonginB
+u
Ubiquitin mediated proteolysis
GF
RTK
MEK
ERK
MNK
PI3K
4E-BP1
eIF4E
Translation
AKT
mTOR
p70S6K
rpS6
HIF-1α
Normoxia
Degradation
PHD
2-OG
Asc
NO
O2
Fe2+
Continuous hypoxia
Hypoxia
mTOR signaling pathway
PI3K-Akt signaling pathway
PKC
DAG
IP3
HIF-1β
DNA
CamK
p300/CBP
Ca2+
Calcium signaling pathway
PLC γ
Intermittent hypoxia
NOX
ROS
Glut1
Promote anaerobic metabolism
PDK-1
+p
PDH
Inhibits TCA cycle metabolism
Glucose
Glut
Pyruvate
Acetyl-CoA
Citrate cycle (TCA cycle)
ATP
Mitochondrion
O2
LDHA
Lactate
TIMP-1
Matrix and barrier functions
CD18
Inflammation
Increase oxygen delivery
EPO
Erythropoiesis
TF
TFRC
Iron metabolism
VEGF
Flt-1
EGF
PAI-1
ANGPT
Tie-2
TIMP-1
Angiogenesis
VEGF signaling pathway
EDN1
iNOS
eNOS
HMOX1
ANP
Vascular tone
Reduce oxygen consumption
HK
PFKL
GAPDH
ALDOA
ENO1
PGK1
PFK2
LDHA
Promote anaerobic metabolism
Bcl-2
p21/p27
Regulate proliferation and apoptosis
04066 5/30/13
(c) Kanehisa Laboratories

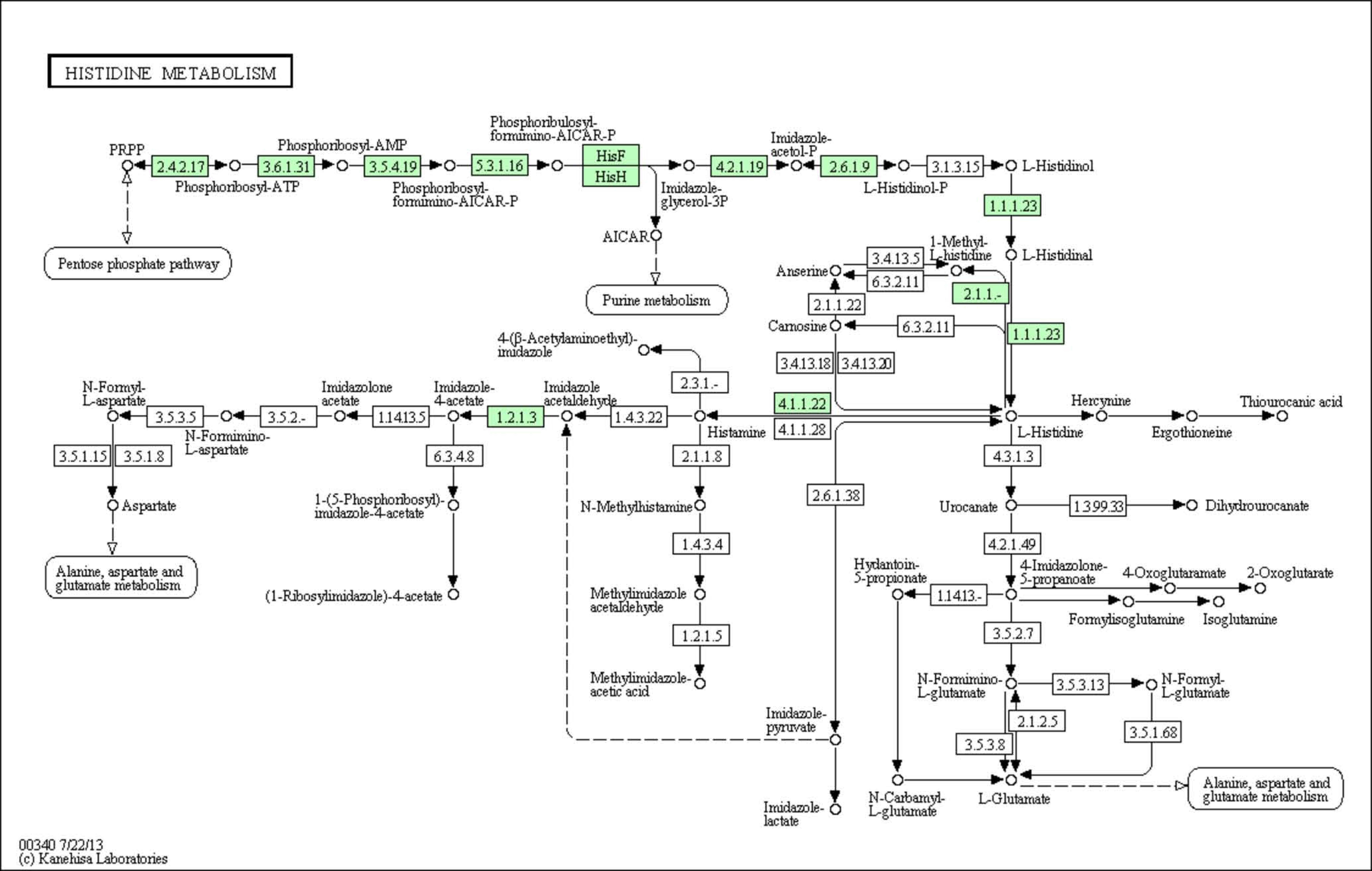

HISTIDINE METABOLISM
PRPP
2.4.2.17
Phosphoribosyl-ATP
3.6.1.31
Phosphoribosyl-AMP
3.5.4.19
Phosphoribosyl-formimino-AICAR-P
5.3.1.16
Phosphoribulosyl-formimino-AICAR-P
HisF
HisH
Imidazole-glycerol-3P
AICAR
Purine metabolism
4.2.1.19
Imidazole-acetol-P
2.6.1.9
L-Histidinol-P
3.1.3.15
L-Histidinol
1.1.1.23
L-Histidinal
1.1.1.23
Pentose phosphate pathway
1-Methyl-L-histidine
3.4.13.5
6.3.2.11
Anserine
2.1.1.-
2.1.1.22
Carnosine
6.3.2.11
3.4.13.18
3.4.13.20
4.1.1.22
4.1.1.28
4-(β-Acetylaminoethyl)-imidazole
2.3.1.-
N-Formyl-L-aspartate
3.5.3.5
N-Formimino-L-aspartate
3.5.2.-
Imidazolone acetate
1.14.13.5
Imidazole-4-acetate
1.2.1.3
Imidazole acetaldehyde
1.4.3.22
Histamine
L-Histidine
Hercynine
Ergothioneine
Thiourocanic acid
3.5.1.15
3.5.1.8
Aspartate
Alanine, aspartate and glutamate metabolism
6.3.4.8
1-(5-Phosphoribosyl)-imidazole-4-acetate
(1-Ribosylimidazole)-4-acetate
2.1.1.8
N-Methylhistamine
1.4.3.4
Methylimidazole acetaldehyde
1.2.1.5
Methylimidazole-acetic acid
2.6.1.38
Imidazole-pyruvate
Imidazole-lactate
4.3.1.3
Urocanate
1.3.99.33
Dihydrourocanate
4.2.1.49
Hydantoin-5-propionate
1.14.13.-
4-Imidazolone-5-propanoate
4-Oxoglutaramate
2-Oxoglutarate
Formylisoglutamine
Isoglutamine
3.5.2.7
N-Formimino-L-glutamate
3.5.3.13
N-Formyl-L-glutamate
2.1.2.5
3.5.3.8
3.5.1.68
N-Carbamyl-L-glutamate
L-Glutamate
Alanine, aspartate and glutamate metabolism
00340 7/22/13
(c) Kanehisa Laboratories

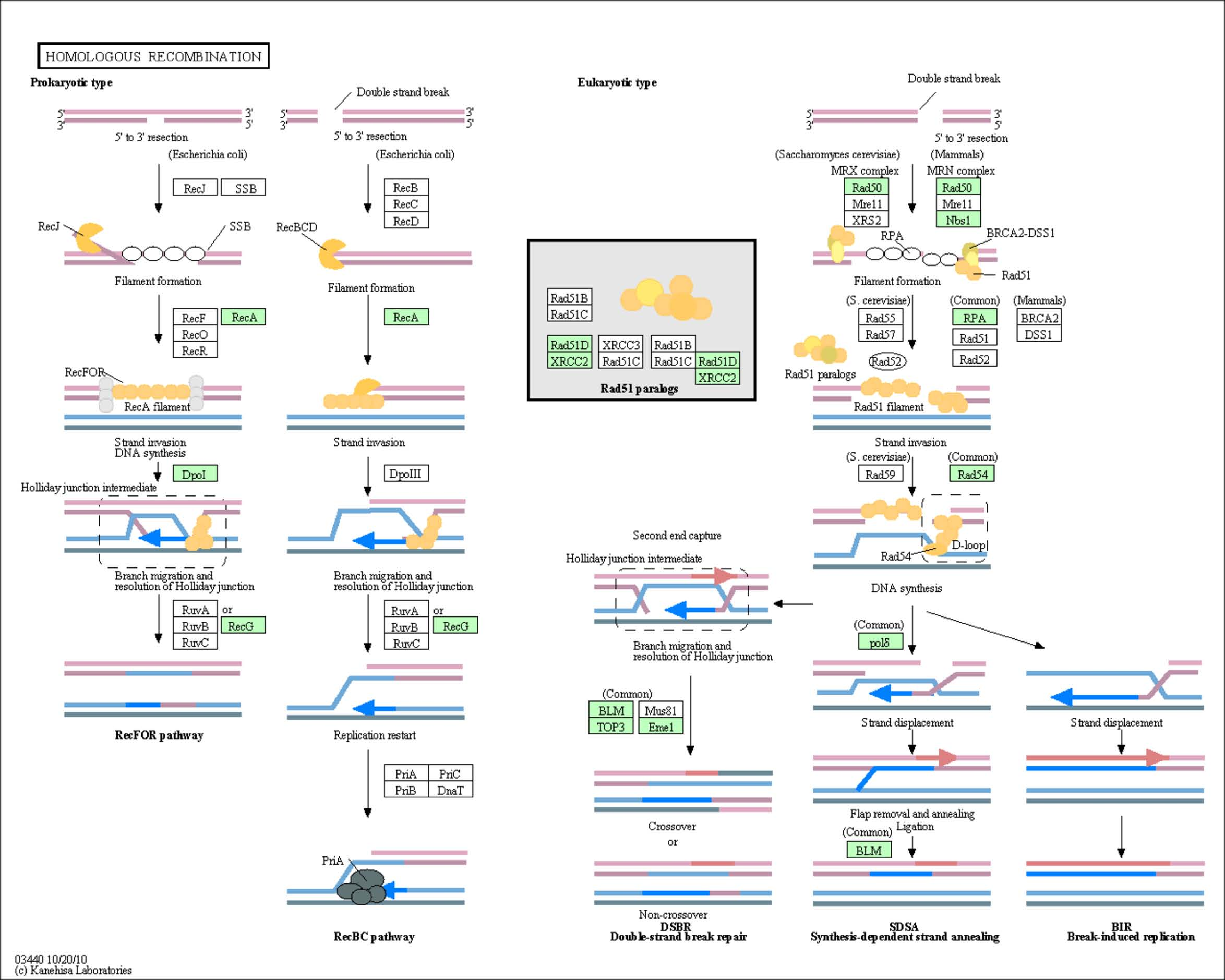
HOMOLOGOUS RECOMBINATION
Prokaryotic type
5'
3'
3'
5'
5' to 3' resection
(Escherichia coli)
RecJ
SSB
RecJ
SSB
Filament formation
RecF
RecO
RecR
RecA
RecFOR
RecA filament
Strand invasion
DNA synthesis
DpoI
Holliday junction intermediate
Branch migration and
resolution of Holliday junction
RuvA
RuvB
RuvC
or
RecG
RecFOR pathway
Double strand break
5' to 3' resection
(Escherichia coli)
RecB
RecC
RecD
RecBCD
Filament formation
RecA
Strand invasion
DpoIII
Branch migration and
resolution of Holliday junction
RuvA
RuvB
RuvC
or
RecG
Replication restart
PriA
PriC
PriB
DnaT
PriA
RecBC pathway
Eukaryotic type
Rad51B
Rad51C
Rad51D
XRCC2
XRCC3
Rad51C
Rad51B
Rad51C
Rad51D
XRCC2
Rad51 paralogs
Double strand break
5' to 3' resection
(Saccharomyces cerevisiae)
MRX complex
Rad50
Mre11
XRS2
(Mammals)
MRN complex
Rad50
Mre11
Nbs1
RPA
BRCA2-DSS1
Rad51
Filament formation
(S. cerevisiae)
Rad55
Rad57
Rad52
(Common)
RPA
Rad51
Rad52
(Mammals)
BRCA2
DSS1
Rad51 paralogs
Rad51 filament
Strand invasion
(S. cerevisiae)
Rad59
(Common)
Rad54
D-loop
Rad54
DNA synthesis
Second end capture
Holliday junction intermediate
Branch migration and
resolution of Holliday junction
(Common)
BLM
TOP3
Mus81
Eme1
Crossover
or
Non-crossover
DSBR
Double-strand break repair
(Common)
polδ
Strand displacement
Flap removal and annealing
Ligation
(Common)
BLM
SDSA
Synthesis-dependent strand annealing
Strand displacement
BIR
Break-induced replication
03440 10/20/10
(c) Kanehisa Laboratories

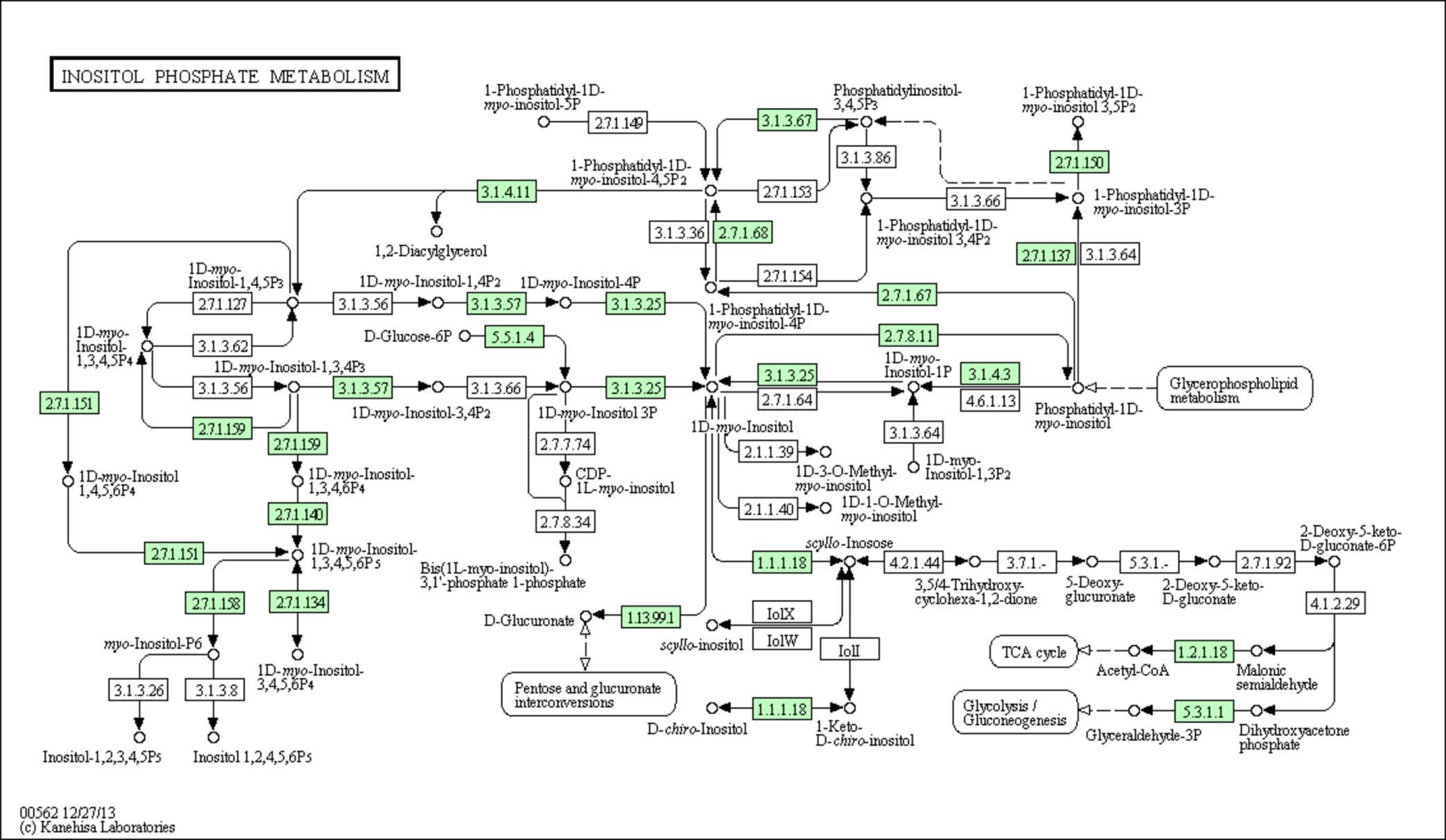
INOSITOL PHOSPHATE METABOLISM
1-Phosphatidyl-1D-myo-inositol-5P
2.7.1.149
Phosphatidylinositol-3,4,5P3
3.1.3.67
1-Phosphatidyl-1D-myo-inositol 3,5P2
2.7.1.150
3.1.3.86
1-Phosphatidyl-1D-myo-inositol-4,5P2
3.1.4.11
2.7.1.153
3.1.3.66
1-Phosphatidyl-1D-myo-inositol-3P
1,2-Diacylglycerol
3.1.3.36
2.7.1.68
1-Phosphatidyl-1D-myo-inositol 3,4P2
2.7.1.137
3.1.3.64
2.7.1.154
1D-myo-Inositol-1,4,5P3
1D-myo-Inositol-1,4P2
1D-myo-Inositol-4P
2.7.1.67
2.7.1.127
3.1.3.56
3.1.3.57
3.1.3.25
1-Phosphatidyl-1D-myo-inositol-4P
1D-myo-Inositol-1,3,4,5P4
3.1.3.62
D-Glucose-6P
5.5.1.4
2.7.8.11
1D-myo-Inositol-1,3,4P3
3.1.3.25
1D-myo-Inositol-1P
3.1.4.3
3.1.3.56
3.1.3.57
3.1.3.66
3.1.3.25
2.7.1.64
4.6.1.13
Glycerophospholipid metabolism
2.7.1.151
1D-myo-Inositol-3,4P2
1D-myo-Inositol 3P
Phosphatidyl-1D-myo-inositol
2.7.1.159
1D-myo-Inositol
2.7.1.159
2.7.7.74
3.1.3.64
2.1.1.39
1D-myo-Inositol 1,4,5,6P4
1D-myo-Inositol-1,3,4,6P4
CDP-1L-myo-inositol
1D-3-O-Methyl-myo-inositol
1D-myo-Inositol-1,3P2
2.7.1.140
2.7.8.34
2.1.1.40
1D-1-O-Methyl-myo-inositol
2.7.1.151
1D-myo-Inositol-1,3,4,5,6P5
scyllo-Inosose
2-Deoxy-5-keto-D-gluconate-6P
1.1.1.18
4.2.1.44
3.7.1.-
5.3.1.-
2.7.1.92
Bis(1L-myo-inositol)-3,1'-phosphate 1-phosphate
3,5/4-Trihydroxy-cyclohexa-1,2-dione
5-Deoxy-glucuronate
2-Deoxy-5-keto-D-gluconate
2.7.1.158
2.7.1.134
D-Glucuronate
1.13.99.1
IolX
4.1.2.29
scyllo-inositol
IolW
IolI
myo-Inositol-P6
TCA cycle
Acetyl-CoA
1.2.1.18
Malonic semialdehyde
1D-myo-Inositol-3,4,5,6P4
3.1.3.26
3.1.3.8
Pentose and glucuronate interconversions
Glycolysis / Gluconeogenesis
1.1.1.18
5.3.1.1
D-chiro-Inositol
1-Keto-D-chiro-inositol
Glyceraldehyde-3P
Dihydroxyacetone phosphate
Inositol-1,2,3,4,5P5
Inositol 1,2,4,5,6P5
00562 12/27/13
(c) Kanehisa Laboratories

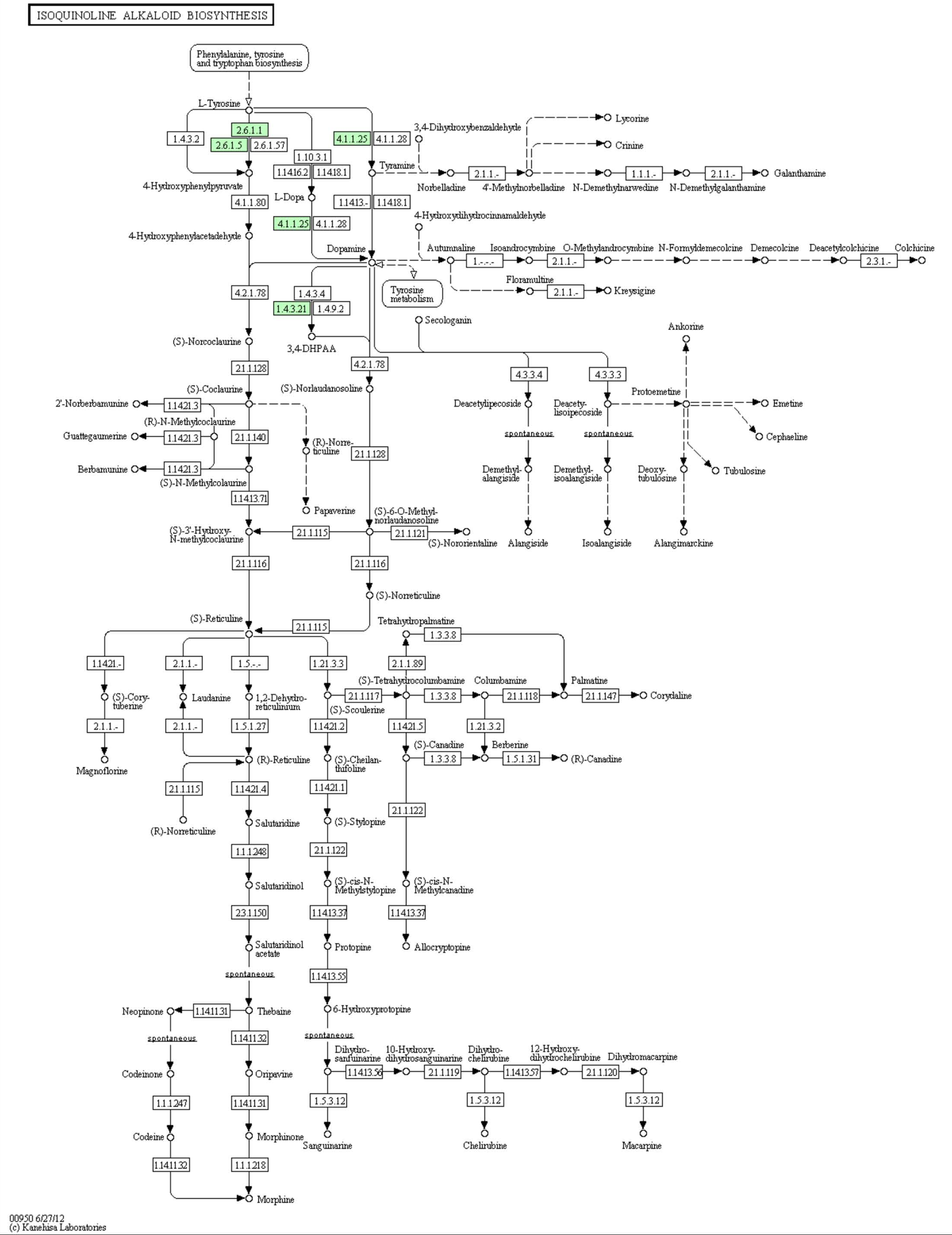
ISOQUINOLINE ALKALOID BIOSYNTHESIS
Phenylalanine, tyrosine and tryptophan biosynthesis
L-Tyrosine
2.6.1.1
2.6.1.5
2.6.1.57
1.4.3.2
1.10.3.1
1.14.16.2
1.14.18.1
4.1.1.25
4.1.1.28
Tyramine
3,4-Dihydroxybenzaldehyde
Lycorine
Crinine
2.1.1.-
1.1.1.-
Galanthamine
Norbelladine
4'-Methylnorbelladine
N-Demethylnarwedine
N-Demethylgalanthamine
4-Hydroxyphenylpyruvate
L-Dopa
4.1.1.80
1.14.13.-
4-Hydroxyphenylacetadehyde
4-Hydroxydihydrocinnamaldehyde
Dopamine
Autumnaline
Isoandrocymbine
O-Methylandrocymbine
N-Formyldemecolcine
Demecolcine
Deacetylcolchicine
Colchicine
1.-.-.-
2.3.1.-
Floramultine
Kreysigine
Tyrosine metabolism
4.2.1.78
1.4.3.4
1.4.3.21
1.4.9.2
Secologanin
Ankorine
(S)-Norcoclaurine
3,4-DHPAA
2.1.1.128
4.3.3.4
4.3.3.3
(S)-Coclaurine
(S)-Norlaudanosoline
Deacetylipecoside
Deacety-lisoipecoside
Protoemetine
Emetine
2'-Norberbamunine
1.14.21.3
(R)-N-Methylcoclaurine
Guattegaumerine
2.1.1.140
(R)-Norre-ticuline
spontaneous
Cephaeline
Berbamunine
(S)-N-Methylcolaurine
Demethyl-alangiside
Demethyl-isoalangiside
Deoxy-tubulosine
Tubulosine
1.14.13.71
Papaverine
(S)-6-O-Methyl-norlaudanosoline
(S)-3'-Hydroxy-N-methylcoclaurine
2.1.1.115
2.1.1.121
(S)-Nororientaline
Alangiside
Isoalangiside
Alangimarckine
2.1.1.116
(S)-Norreticuline
(S)-Reticuline
Tetrahydropalmatine
1.3.3.8
1.14.21.-
2.1.1.-
1.5.-.-
1.21.3.3
2.1.1.89
(S)-Tetrahydrocolumbamine
Columbamine
Palmatine
(S)-Cory-tuberine
Laudanine
1,2-Dehydro-reticulinium
2.1.1.117
2.1.1.118
2.1.1.147
Corydaline
(S)-Scoulerine
1.5.1.27
1.14.21.2
1.14.21.5
1.21.3.2
(S)-Canadine
Berberine
1.5.1.31
(R)-Canadine
Magnoflorine
(R)-Reticuline
(S)-Cheilan-thifoline
1.14.21.4
1.14.21.1
2.1.1.122
(R)-Norreticuline
Salutaridine
(S)-Stylopine
1.1.1.248
Salutaridinol
(S)-cis-N-Methylstylopine
(S)-cis-N-Methylcanadine
2.3.1.150
1.14.13.37
Salutaridinol acetate
Protopine
Allocryptopine
1.14.13.55
Neopinone
1.14.11.31
Thebaine
6-Hydroxyprotopine
1.14.11.32
Dihydro-sanfuinarine
10-Hydroxy-dihydrosanguinarine
Dihydro-chelirubine
12-Hydroxy-dihydrochelirubine
Dihydromacarpine
1.14.13.56
2.1.1.119
1.14.13.57
2.1.1.120
Codeinone
Oripavine
1.5.3.12
1.1.1.247
Codeine
Morphinone
Sanguinarine
Chelirubine
Macarpine
1.1.1.218
Morphine
00950 6/27/12
(c) Kanehisa Laboratories

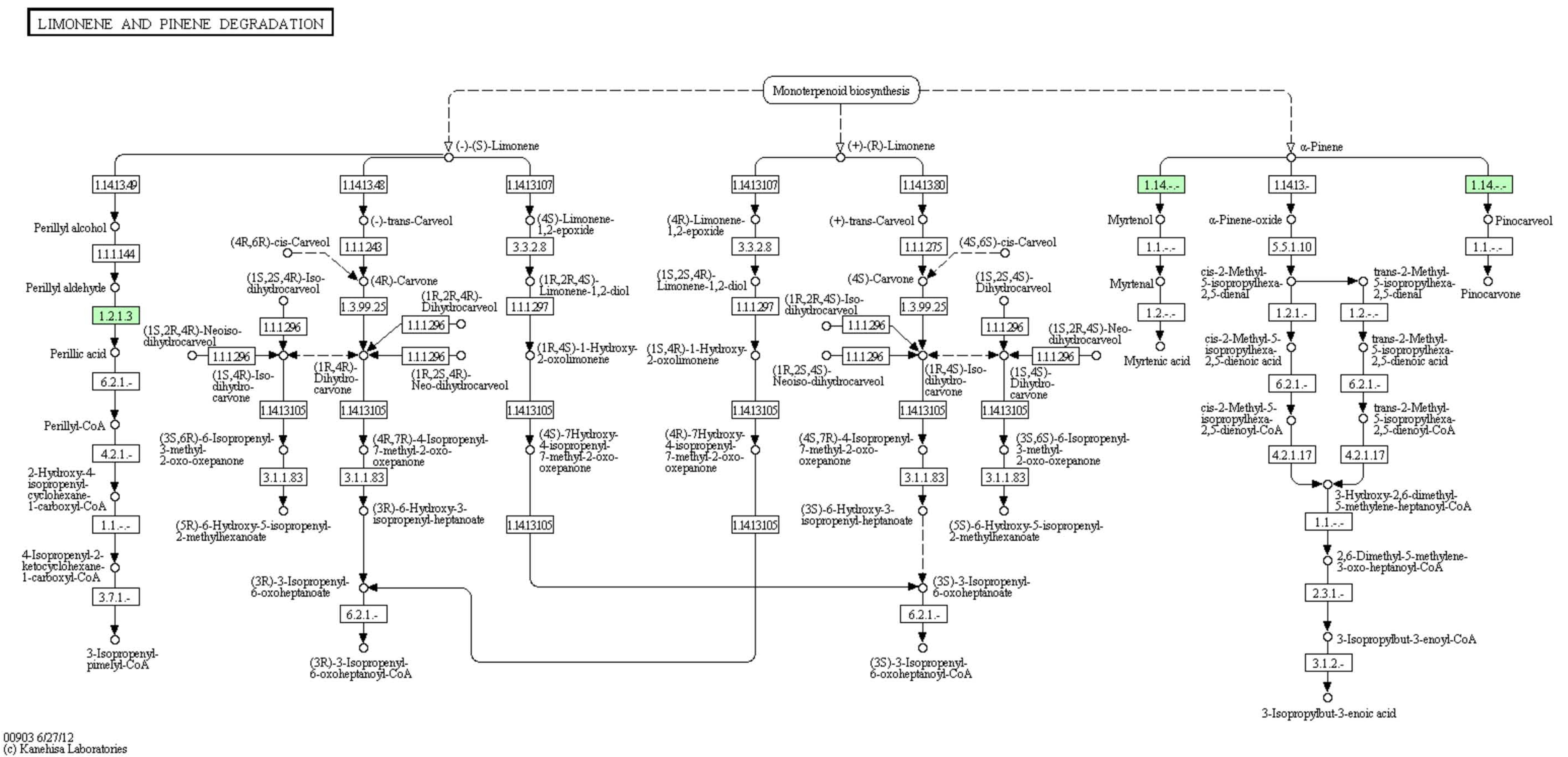

LIMONENE AND PINENE DEGRADATION
Monoterpenoid biosynthesis
(-)-(S)-Limonene
(+)-(R)-Limonene
α-Pinene
1.14.13.49
Perillyl alcohol
1.1.1.144
Perillyl aldehyde
1.2.1.3
Perillic acid
6.2.1.-
Perillyl-CoA
4.2.1.-
2-Hydroxy-4-isopropenyl-cyclohexane-1-carboxyl-CoA
1.1.-.-
4-Isopropenyl-2-ketocyclohexane-1-carboxyl-CoA
3.7.1.-
3-Isopropenyl-pimelyl-CoA
1.14.13.48
(-)-trans-Carveol
1.1.1.243
(4R,6R)-cis-Carveol
(4R)-Carvone
1.3.99.25
(1S,2S,4R)-Iso-dihydrocarveol
(1R,2R,4R)-Dihydrocarveol
1.1.1.296
(1S,2R,4R)-Neoiso-dihydrocarveol
(1S,4R)-Iso-dihydro-carvone
(1R,4R)-Dihydro-carvone
(1R,2S,4R)-Neo-dihydrocarveol
1.14.13.105
(3S,6R)-6-Isopropenyl-3-methyl-2-oxo-oxepanone
3.1.1.83
(5R)-6-Hydroxy-5-isopropenyl-2-methylhexanoate
(4R,7R)-4-Isopropenyl-7-methyl-2-oxo-oxepanone
(3R)-6-Hydroxy-3-isopropenyl-heptanoate
(3R)-3-Isopropenyl-6-oxoheptanoate
6.2.1.-
(3R)-3-Isopropenyl-6-oxoheptanoyl-CoA
1.14.13.107
(4S)-Limonene-1,2-epoxide
3.3.2.8
(1R,2R,4S)-Limonene-1,2-diol
1.1.1.297
(1R,4S)-1-Hydroxy-2-oxolimonene
(4S)-7Hydroxy-4-isopropenyl-7-methyl-2-oxo-oxepanone
(4R)-Limonene-1,2-epoxide
(1S,2S,4R)-Limonene-1,2-diol
(1S,4R)-1-Hydroxy-2-oxolimonene
(4R)-7Hydroxy-4-isopropenyl-7-methyl-2-oxo-oxepanone
1.14.13.80
(+)-trans-Carveol
1.1.1.275
(4S,6S)-cis-Carveol
(4S)-Carvone
(1R,2R,4S)-Iso-dihydrocarveol
(1S,2S,4S)-Dihydrocarveol
(1S,2R,4S)-Neo-dihydrocarveol
(1R,2S,4S)-Neoiso-dihydrocarveol
(1R,4S)-Iso-dihydro-carvone
(1S,4S)-Dihydro-carvone
(4S,7R)-4-Isopropenyl-7-methyl-2-oxo-oxepanone
(3S,6S)-6-Isopropenyl-3-methyl-2-oxo-oxepanone
(3S)-6-Hydroxy-3-isopropenyl-heptanoate
(5S)-6-Hydroxy-5-isopropenyl-2-methylhexanoate
(3S)-3-Isopropenyl-6-oxoheptanoate
(3S)-3-Isopropenyl-6-oxoheptanoyl-CoA
1.14.-.-
Myrtenol
1.1.-.-
Myrtenal
1.2.-.-
Myrtenic acid
1.14.13.-
α-Pinene-oxide
5.5.1.10
cis-2-Methyl-5-isopropylhexa-2,5-dienal
trans-2-Methyl-5-isopropylhexa-2,5-dienal
1.2.1.-
cis-2-Methyl-5-isopropylhexa-2,5-dienoic acid
trans-2-Methyl-5-isopropylhexa-2,5-dienoic acid
cis-2-Methyl-5-isopropylhexa-2,5-dienoyl-CoA
trans-2-Methyl-5-isopropylhexa-2,5-dienoyl-CoA
4.2.1.17
3-Hydroxy-2,6-dimethyl-5-methylene-heptanoyl-CoA
2,6-Dimethyl-5-methylene-3-oxo-heptanoyl-CoA
2.3.1.-
3-Isopropylbut-3-enoyl-CoA
3.1.2.-
3-Isopropylbut-3-enoic acid
Pinocarveol
Pinocarvone
00903 6/27/12
(c) Kanehisa Laboratories

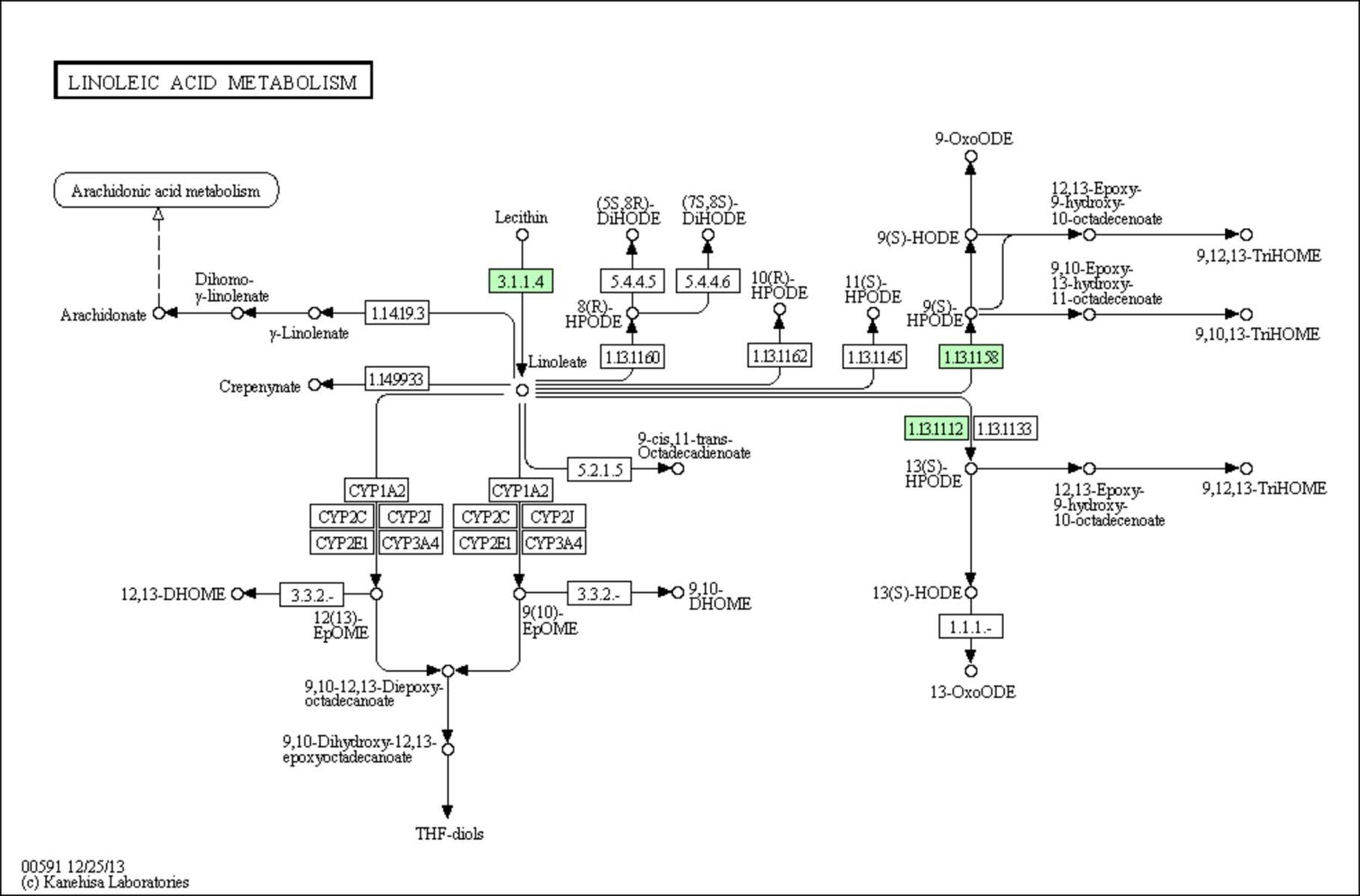
LINOLEIC ACID METABOLISM
Arachidonic acid metabolism
Arachidonate
Dihomo-γ-linolenate
γ-Linolenate
1.14.19.3
Crepenynate
1.14.99.33
Lecithin
3.1.1.4
Linoleate
(5S,8R)-DiHODE
(7S,8S)-DiHODE
5.4.4.5
5.4.4.6
8(R)-HPODE
1.13.11.60
10(R)-HPODE
1.13.11.62
11(S)-HPODE
1.13.11.45
9-OxoODE
9(S)-HODE
9(S)-HPODE
1.13.11.58
12,13-Epoxy-9-hydroxy-10-octadecenoate
9,12,13-TriHOME
9,10-Epoxy-13-hydroxy-11-octadecenoate
9,10,13-TriHOME
1.13.11.12
1.13.11.33
13(S)-HPODE
12,13-Epoxy-9-hydroxy-10-octadecenoate
9,12,13-TriHOME
13(S)-HODE
1.1.1.-
13-OxoODE
9-cis,11-trans-Octadecadienoate
5.2.1.5
CYP1A2
CYP2C
CYP2J
CYP2E1
CYP3A4
CYP1A2
CYP2C
CYP2J
CYP2E1
CYP3A4
12,13-DHOME
3.3.2.-
12(13)-EpOME
9(10)-EpOME
3.3.2.-
9,10-DHOME
9,10-12,13-Diepoxy-octadecanoate
9,10-Dihydroxy-12,13-epoxyoctadecanoate
THF-diols
00591 12/25/13
(c) Kanehisa Laboratories

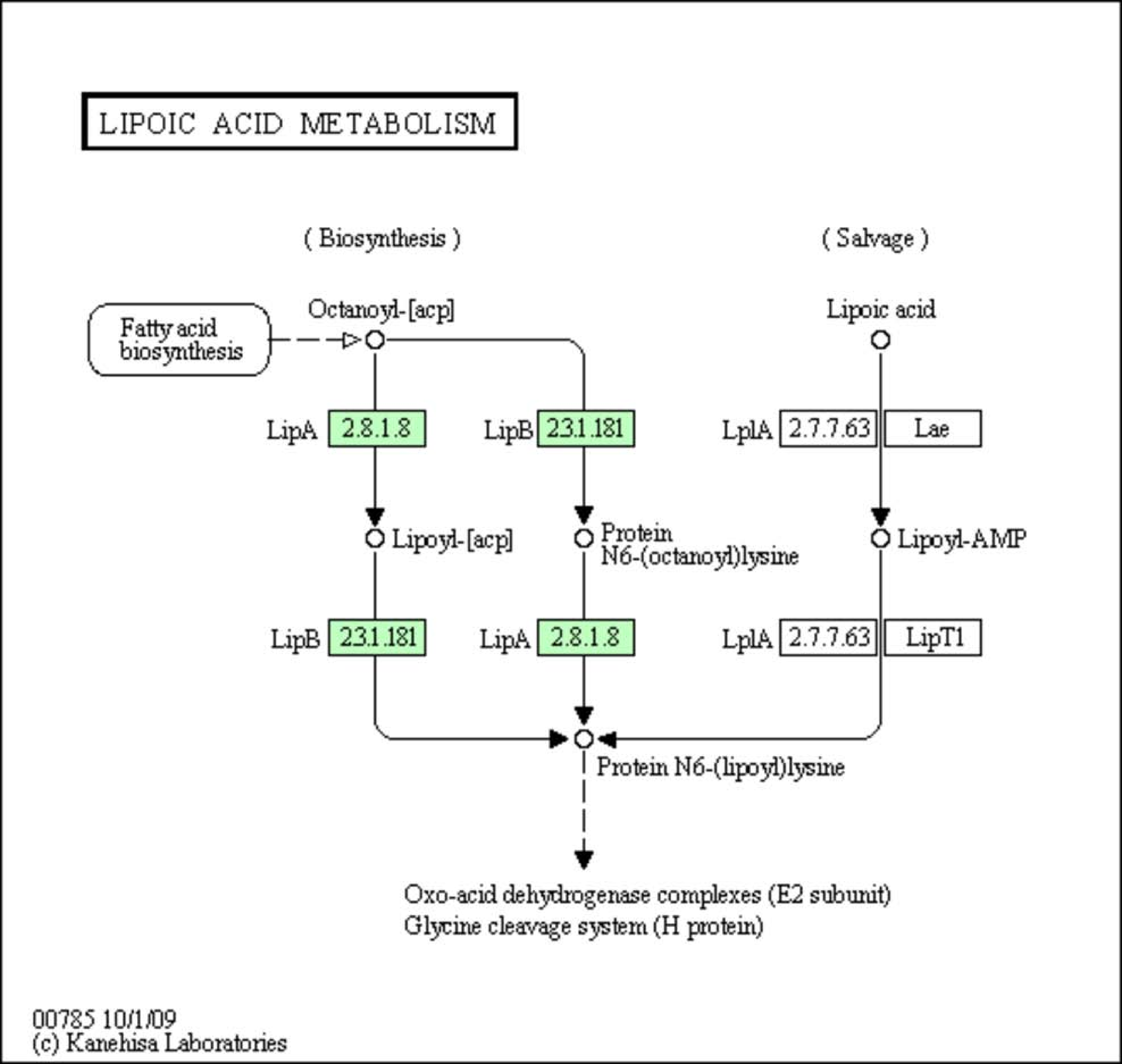
LIPOIC ACID METABOLISM
(Biosynthesis)
(Salvage)
Fatty acid biosynthesis
Octanoyl-[acp]
Lipoic acid
LipA
2.8.1.8
LipB
23.1.181
LplA
2.7.7.63
Lae
Lipoyl-[acp]
Protein N6-(octanoyl)lysine
Lipoyl-AMP
LipB
23.1.181
LipA
2.8.1.8
LplA
2.7.7.63
LipT1
Protein N6-(lipoyl)lysine
Oxo-acid dehydrogenase complexes (E2 subunit)
Glycine cleavage system (H protein)
00785 10/1/09
(c) Kanehisa Laboratories

LIPOPOLYSACCHARIDE BIOSYNTHESIS

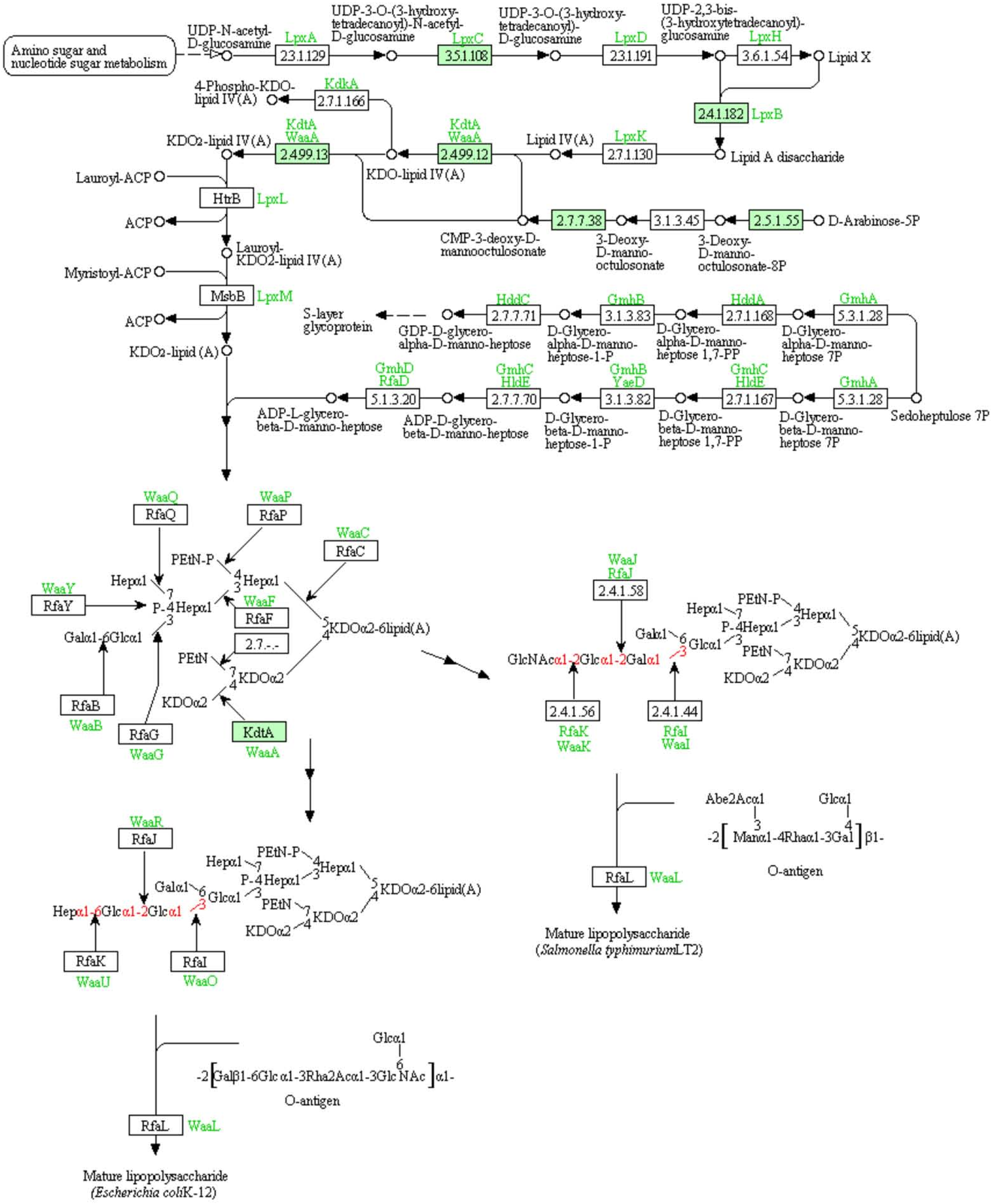

00540 10/10/12

(c) Kanehisa Laboratories

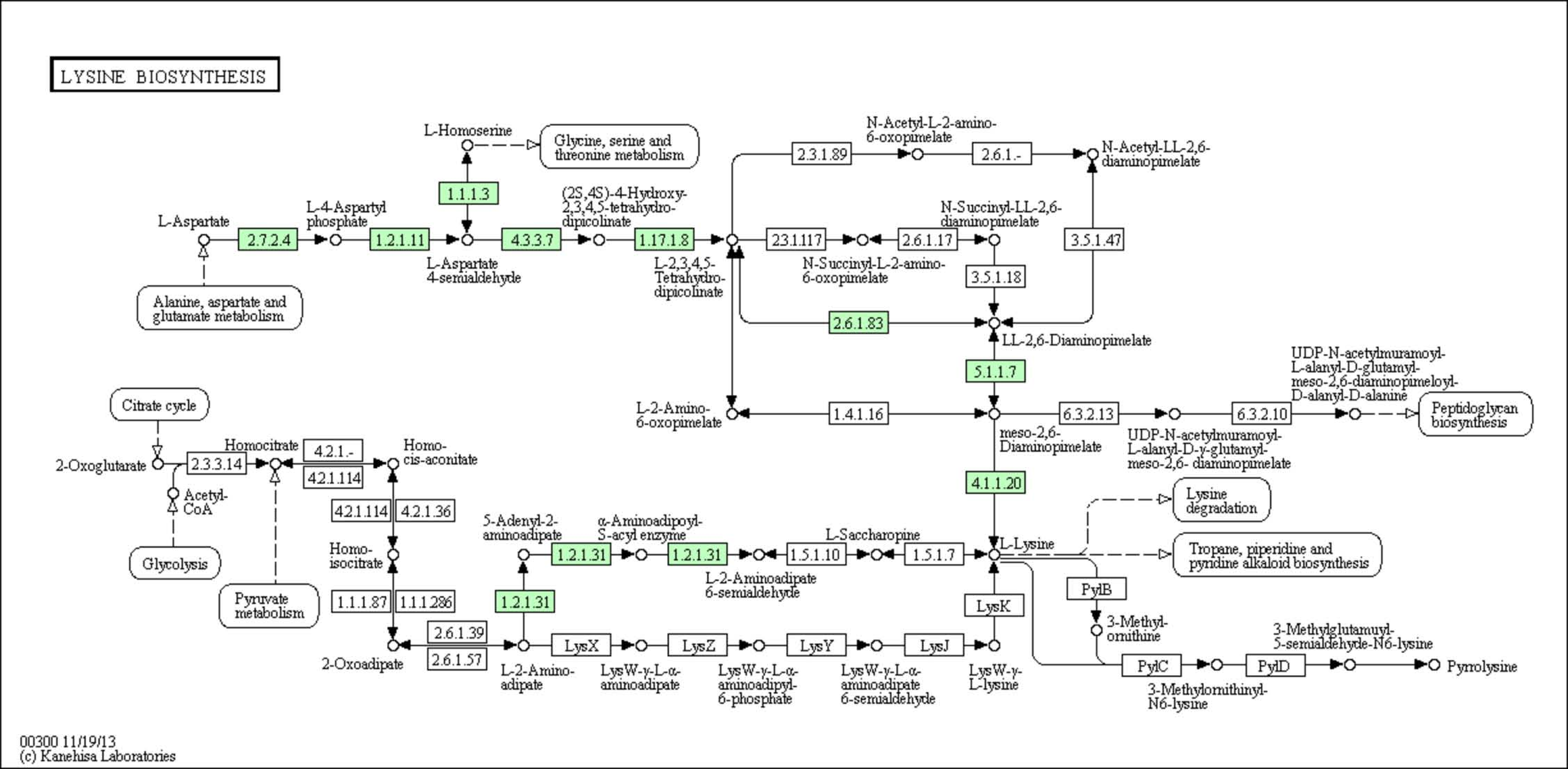

LYSINE BIOSYNTHESIS
L-Homoserine
Glycine, serine and threonine metabolism
1.1.1.3
L-Aspartate
2.7.2.4
L-4-Aspartyl phosphate
1.2.1.11
L-Aspartate 4-semialdehyde
4.3.3.7
(2S,4S)-4-Hydroxy-2,3,4,5-tetrahydro-dipicolinate
1.17.1.8
L-2,3,4,5-Tetrahydro-dipicolinate
Alanine, aspartate and glutamate metabolism
2.3.1.89
N-Acetyl-L-2-amino-6-oxopimelate
2.6.1.-
N-Acetyl-LL-2,6-diaminopimelate
2.3.1.117
N-Succinyl-L-2-amino-6-oxopimelate
2.6.1.17
N-Succinyl-LL-2,6-diaminopimelate
3.5.1.18
3.5.1.47
2.6.1.83
LL-2,6-Diaminopimelate
5.1.1.7
L-2-Amino-6-oxopimelate
1.4.1.16
meso-2,6-Diaminopimelate
6.3.2.13
UDP-N-acetylmuramoyl-L-alanyl-D-γ-glutamyl-meso-2,6- diaminopimelate
6.3.2.10
UDP-N-acetylmuramoyl-L-alanyl-D-glutamyl-meso-2,6-diaminopimeloyl-D-alanyl-D-alanine
Peptidoglycan biosynthesis
Citrate cycle
2-Oxoglutarate
2.3.3.14
Homocitrate
4.2.1.-
4.2.1.114
Homo-cis-aconitate
Acetyl-CoA
Glycolysis
Pyruvate metabolism
4.2.1.114
4.2.1.36
Homo-isocitrate
1.1.1.87
1.1.1.286
2-Oxoadipate
2.6.1.39
2.6.1.57
4.1.1.20
Lysine degradation
5-Adenyl-2-aminoadipate
1.2.1.31
α-Aminoadipoyl-S-acyl enzyme
1.2.1.31
L-2-Aminoadipate 6-semialdehyde
1.5.1.10
L-Saccharopine
1.5.1.7
L-Lysine
Tropane, piperidine and pyridine alkaloid biosynthesis
1.2.1.31
L-2-Amino-adipate
LysX
LysW-γ-L-α-aminoadipate
LysZ
LysW-γ-L-α-aminoadipyl-6-phosphate
LysY
LysW-γ-L-α-aminoadipate 6-semialdehyde
LysJ
LysW-γ-L-lysine
LysK
PylB
3-Methyl-ornithine
PylC
3-Methylornithinyl-N6-lysine
PylD
3-Methylglutamuyl-5-semialdehyde-N6-lysine
Pyrrolysine
00300 11/19/13
(c) Kanehisa Laboratories

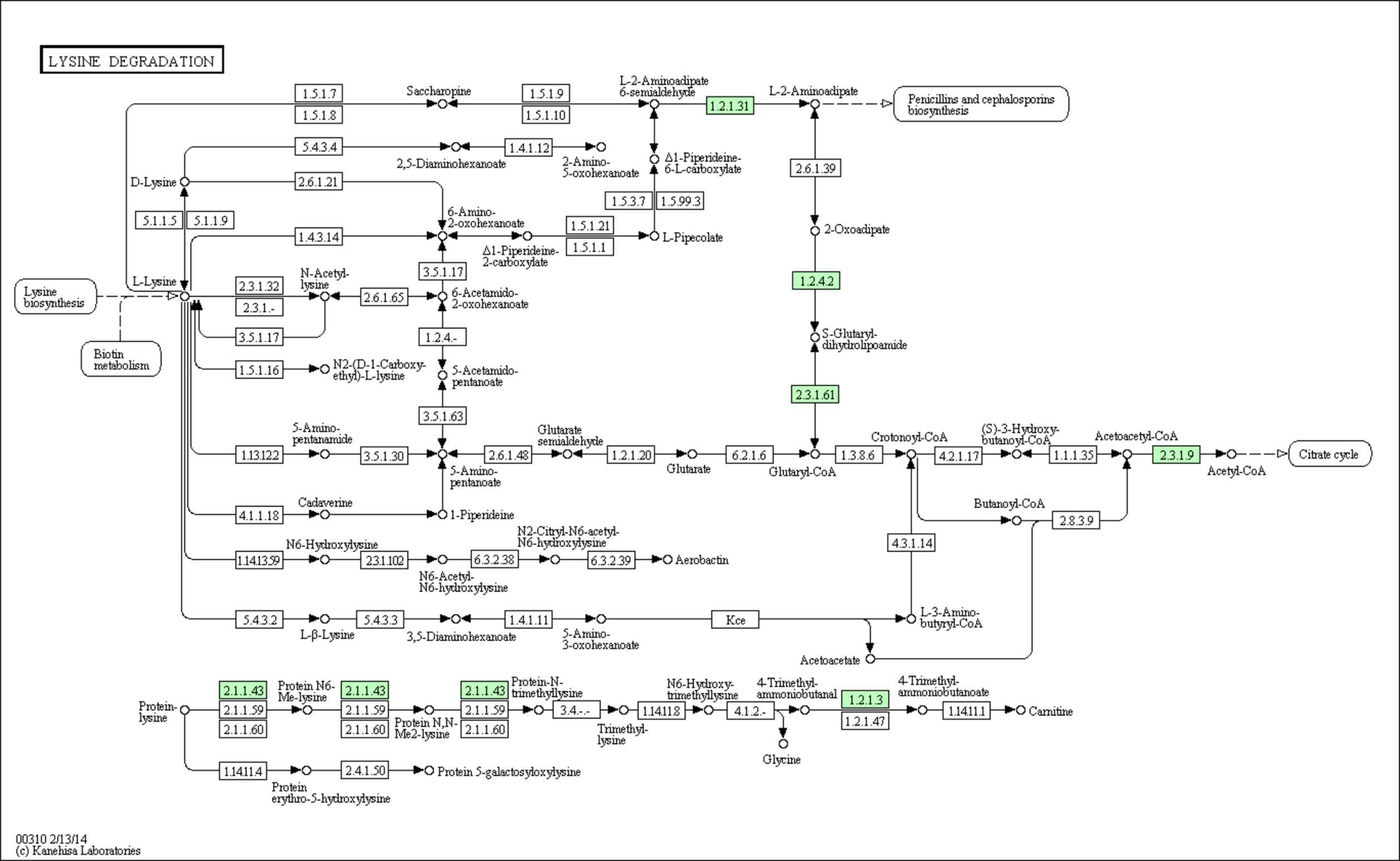
LYSINE DEGRADATION
1.5.1.7
1.5.1.8
Saccharopine
1.5.1.9
1.5.1.10
L-2-Aminoadipate 6-semialdehyde
1.2.1.31
L-2-Aminoadipate
Penicillins and cephalosporins biosynthesis
5.4.3.4
2,5-Diaminohexanoate
1.4.1.12
2-Amino-5-oxohexanoate
Δ1-Piperideine-6-L-carboxylate
2.6.1.39
D-Lysine
2.6.1.21
1.5.3.7
1.5.99.3
5.1.1.5
5.1.1.9
6-Amino-2-oxohexanoate
1.4.3.14
1.5.1.21
1.5.1.1
L-Pipecolate
2-Oxoadipate
Δ1-Piperideine-2-carboxylate
3.5.1.17
1.2.4.2
Lysine biosynthesis
L-Lysine
2.3.1.32
2.3.1.-
N-Acetyl-lysine
2.6.1.65
6-Acetamido-2-oxohexanoate
Biotin metabolism
3.5.1.17
1.2.4.-
S-Glutaryl-dihydrolipoamide
1.5.1.16
N2-(D-1-Carboxy-ethyl)-L-lysine
5-Acetamido-pentanoate
2.3.1.61
3.5.1.63
5-Amino-pentanamide
Glutarate semialdehyde
Crotonoyl-CoA
(S)-3-Hydroxy-butanoyl-CoA
Acetoacetyl-CoA
1.13.12.2
3.5.1.30
2.6.1.48
1.2.1.20
Glutarate
6.2.1.6
Glutaryl-CoA
1.3.8.6
4.2.1.17
1.1.1.35
2.3.1.9
Acetyl-CoA
Citrate cycle
5-Amino-pentanoate
Cadaverine
4.1.1.18
1-Piperideine
Butanoyl-CoA
2.8.3.9
N2-Citryl-N6-acetyl-N6-hydroxylysine
N6-Hydroxylysine
4.3.1.14
1.14.13.59
2.3.1.102
6.3.2.38
6.3.2.39
Aerobactin
N6-Acetyl-N6-hydroxylysine
5.4.3.2
5.4.3.3
1.4.1.11
Kce
L-3-Amino-butyryl-CoA
L-β-Lysine
3,5-Diaminohexanoate
5-Amino-3-oxohexanoate
Acetoacetate
2.1.1.43
Protein N6-Me-lysine
2.1.1.43
2.1.1.43
Protein-N-trimethyllysine
N6-Hydroxy-trimethyllysine
4-Trimethyl-ammoniobutanal
1.2.1.3
4-Trimethyl-ammoniobutanoate
Protein-lysine
2.1.1.59
2.1.1.59
2.1.1.59
3.4.-.-
1.14.11.8
4.1.2.-
1.14.11.1
Carnitine
2.1.1.60
2.1.1.60
2.1.1.60
Protein N,N-Me2-lysine
Trimethyl-lysine
1.2.1.47
Glycine
1.14.11.4
2.4.1.50
Protein 5-galactosyloxylysine
Protein erythro-5-hydroxylysine
00310 2/13/14
(c) Kanehisa Laboratories

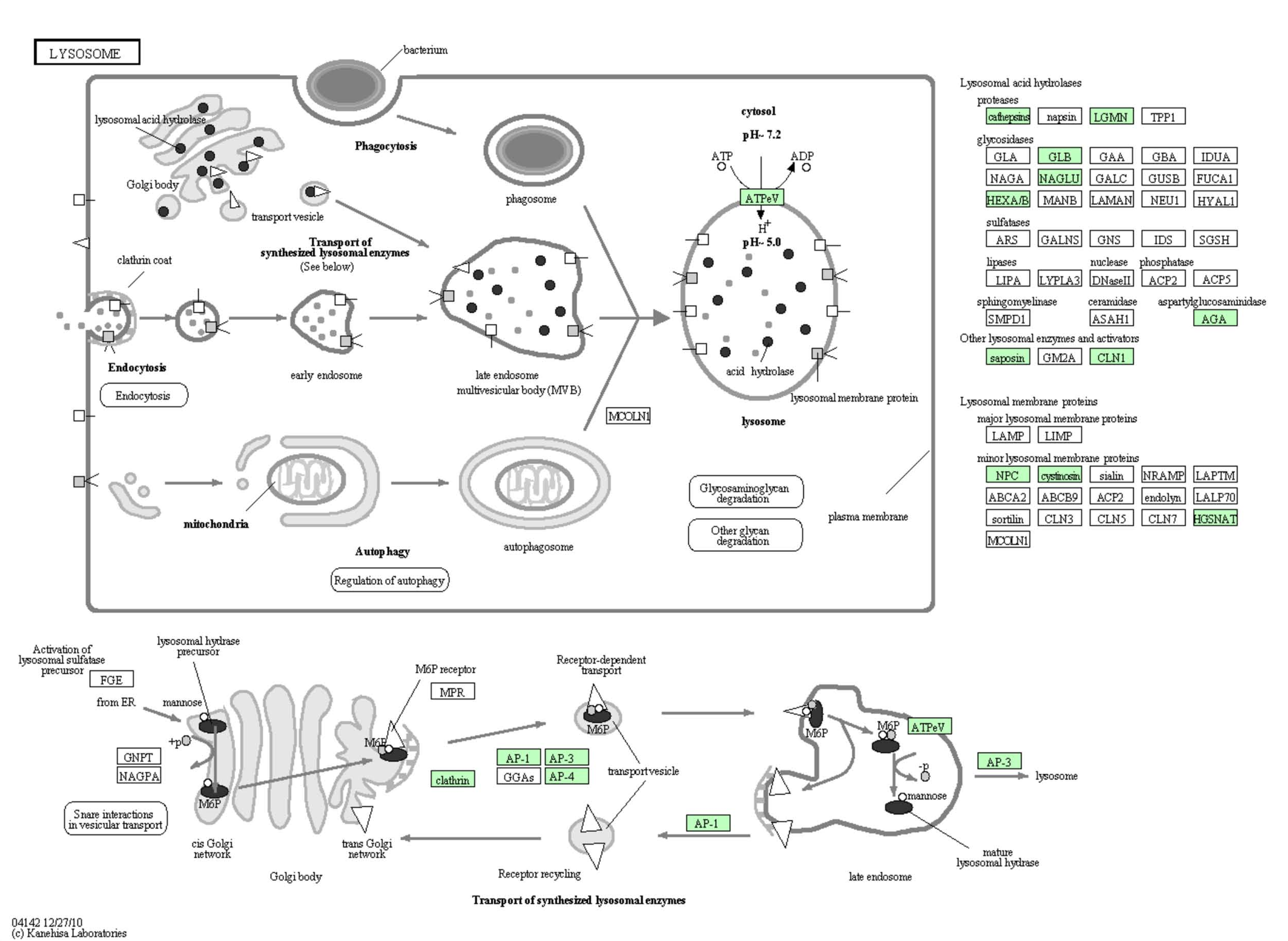
LYSOSOME
bacterium
lysosomal acid hydrolase
Golgi body
transport vesicle
Phagocytosis
phagosome
Transport of synthesized lysosomal enzymes
(See below)
clathrin coat
Endocytosis
Endocytosis
early endosome
late endosome
multivesicular body (MVB)
cytosol
pH~ 7.2
ATP
ADP
ATPeV
H+
pH~ 5.0
acid hydrolase
lysosomal membrane protein
lysosome
MCOLN1
mitochondria
Autophagy
Regulation of autophagy
autophagosome
Glycosaminoglycan degradation
Other glycan degradation
plasma membrane
Lysosomal acid hydrolases
proteases
cathepsins
napsin
LGMN
TPP1
glycosidases
GLA
GLB
GAA
GBA
IDUA
NAGA
NAGLU
GALC
GUSB
FUCA1
HEXA/B
MANB
LAMAN
NEU1
HYAL1
sulfatases
ARS
GALNS
GNS
IDS
SGSH
lipases
LIPA
LYPLA3
nuclease
DNaseII
phosphatase
ACP2
ACP5
sphingomyelinase
SMPD1
ceramidase
ASAH1
aspartylglucosaminidase
AGA
Other lysosomal enzymes and activators
saposin
GM2A
CLN1
Lysosomal membrane proteins
major lysosomal membrane proteins
LAMP
LIMP
minor lysosomal membrane proteins
NPC
cystinosin
sialin
NRAMP
LAPTM
ABCA2
ABCB9
ACP2
endolyn
LALP70
sortilin
CLN3
CLN5
CLN7
HGSNAT
MCOLN1
Activation of lysosomal sulfatase precursor
FGE
lysosomal hydrase precursor
from ER
mannose
+p
GNPT
NAGPA
M6P
Snare interactions in vesicular transport
cis Golgi network
M6P receptor
MPR
M6P
clathrin
trans Golgi network
Golgi body
Receptor-dependent transport
M6P
AP-1
AP-3
GGAs
AP-4
transport vesicle
Receptor recycling
M6P
M6P
ATPeV
-p
mannose
AP-3
lysosome
AP-1
mature lysosomal hydrase
late endosome
Transport of synthesized lysosomal enzymes
04142 12/27/10
(c) Kanehisa Laboratories

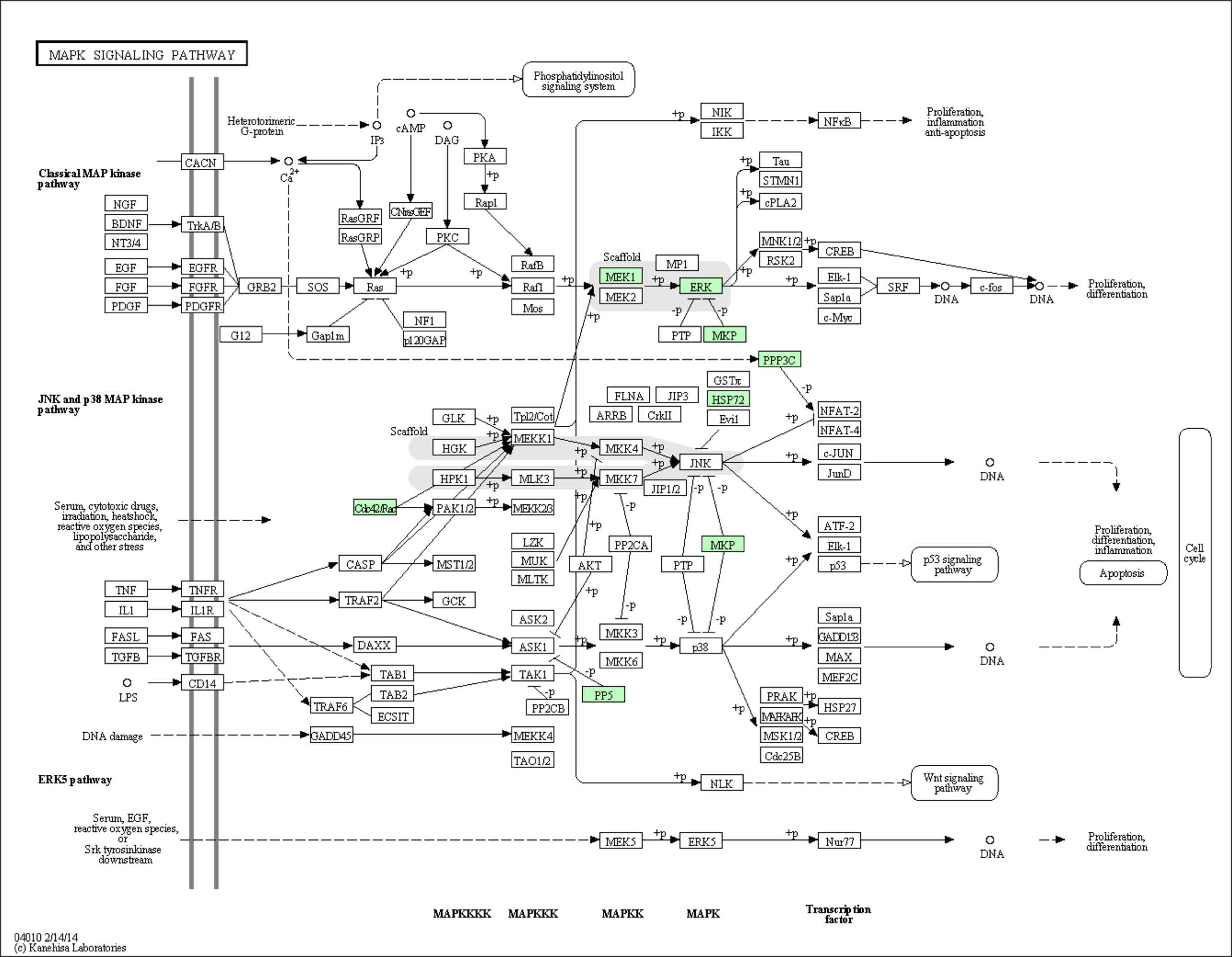
MAPK SIGNALING PATHWAY
Classical MAP kinase pathway
JNK and p38 MAP kinase pathway
ERK5 pathway
Phosphatidylinositol signaling system
Heterotorimeric G-protein
IP3
cAMP
DAG
CACN
Ca2+
PKA
Rap1
RasGRF
RasGRP
CNrasGEF
PKC
RafB
Raf1
Mos
NGF
BDNF
NT3/4
TrkA/B
EGF
FGF
PDGF
EGFR
FGFR
PDGFR
GRB2
SOS
Ras
NF1
p120GAP
G12
Gap1m
Scaffold
MP1
MEK1
MEK2
ERK
PTP
MKP
NIK
IKK
NFκB
Proliferation, inflammation anti-apoptosis
Tau
STMN1
cPLA2
MNK1/2
RSK2
CREB
Elk-1
Sap1a
c-Myc
SRF
DNA
c-fos
Proliferation, differentiation
PPP3C
NFAT-2
NFAT-4
GSTπ
HSP72
Evi1
FLNA
JIP3
ARRB
CrkII
GLK
HGK
HPK1
Tpl2/Cot
MEKK1
MLK3
MKK4
MKK7
JNK
JIP1/2
c-JUN
JunD
Cdc42/Rac
PAK1/2
MEKK2/3
Serum, cytotoxic drugs, irradiation, heatshock, reactive oxygen species, lipopolysaccharide, and other stress
LZK
MUK
MLTK
AKT
PP2CA
ATF-2
p53
p53 signaling pathway
Proliferation, differentiation, inflammation
Apoptosis
Cell cycle
TNF
IL1
FASL
TGFB
LPS
TNFR
IL1R
FAS
TGFBR
CD14
CASP
TRAF2
DAXX
MST1/2
GCK
ASK2
ASK1
MKK3
MKK6
p38
Sap1a
GADD153
MAX
MEF2C
TAB1
TAB2
TRAF6
ECSIT
TAK1
PP2CB
PP5
PRAK
MAPKAPK
MSK1/2
Cdc25B
HSP27
CREB
DNA damage
GADD45
MEKK4
TAO1/2
NLK
Wnt signaling pathway
Serum, EGF, reactive oxygen species, or Srk tyrosinkinase downstream
MEK5
ERK5
Nur77
MAPKKKK
MAPKKK
MAPKK
MAPK
Transcription factor
04010 2/14/14
(c) Kanehisa Laboratories

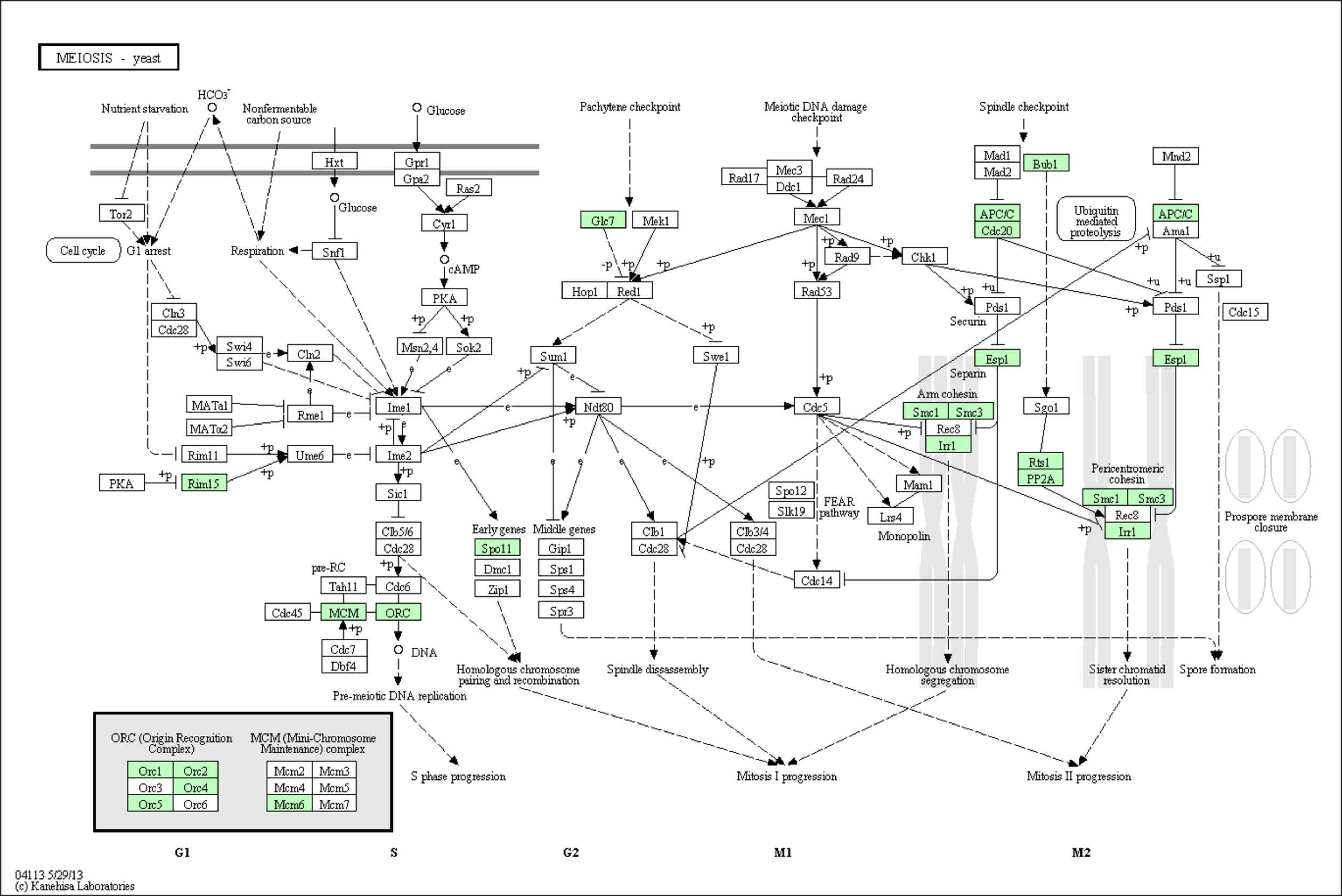
MEIOSIS - yeast
Nutrient starvation
HCO3-
Nonfermentable carbon source
Glucose
Pachytene checkpoint
Meiotic DNA damage checkpoint
Spindle checkpoint
Hxt
Gpr1
Gpa2
Ras2
Cyr1
cAMP
PKA
Tor2
Cell cycle
G1 arrest
Respiration
Snf1
Msn2,4
Sok2
Cln3
Cdc28
Swi4
Swi6
Cln2
MATa1
MATα2
Rme1
Ime1
Ime2
Rim11
Ume6
PKA
Rim15
Sic1
Clb5/6
Cdc28
pre-RC
Tah11
Cdc6
Cdc45
MCM
ORC
Cdc7
Dbf4
DNA
Pre-meiotic DNA replication
S phase progression
Glc7
Mek1
Hop1
Red1
Sum1
Swe1
Ndt80
Early genes
Spo11
Dmc1
Zip1
Middle genes
Gip1
Sps1
Sps4
Spr3
Clb1
Cdc28
Clb3/4
Cdc28
Homologous chromosome pairing and recombination
Spindle dissassembly
Mitosis I progression
Rad17
Mec3
Ddc1
Rad24
Mec1
Rad9
Chk1
Rad53
Cdc5
Spo12
Slk19
FEAR pathway
Cdc14
Mam1
Lrs4
Monopolin
Mad1
Mad2
Bub1
APC/C
Cdc20
Ubiquitin mediated proteolysis
Mnd2
APC/C
Ama1
Ssp1
Pds1
Securin
Pds1
Cdc15
Esp1
Separin
Esp1
Arm cohesin
Smc1
Smc3
Rec8
Irr1
Sgo1
Rts1
PP2A
Pericentromeric cohesin
Smc1
Smc3
Rec8
Irr1
Prospore membrane closure
Homologous chromosome segregation
Sister chromatid resolution
Spore formation
Mitosis II progression
ORC (Origin Recognition Complex)
Orc1
Orc2
Orc3
Orc4
Orc5
Orc6
MCM (Mini-Chromosome Maintenance) complex
Mcm2
Mcm3
Mcm4
Mcm5
Mcm6
Mcm7
G1
S
G2
M1
M2
04113 5/29/13
(c) Kanehisa Laboratories

METABOLISM OF XENOBIOTICS BY CYTOCHROME P450

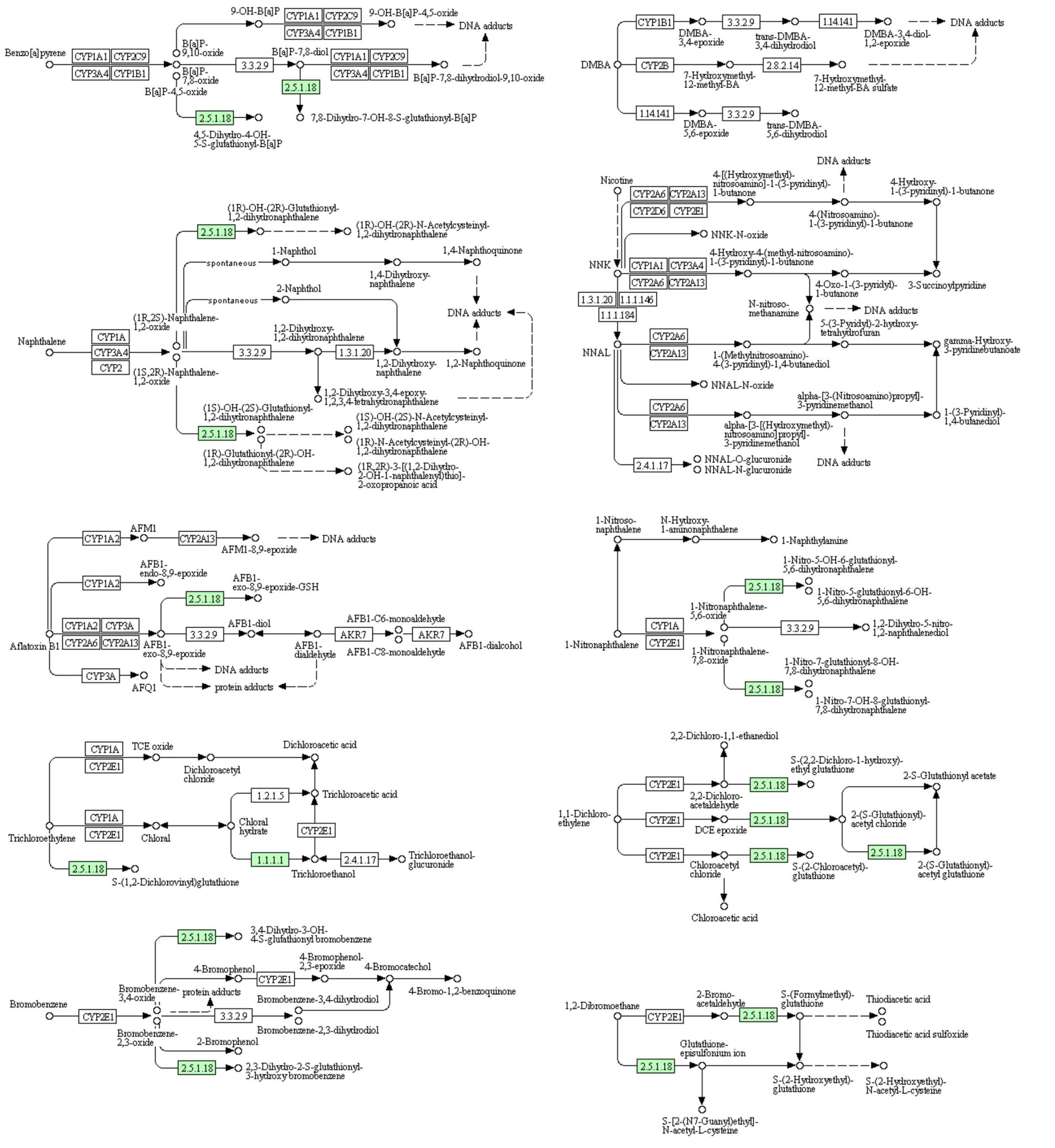

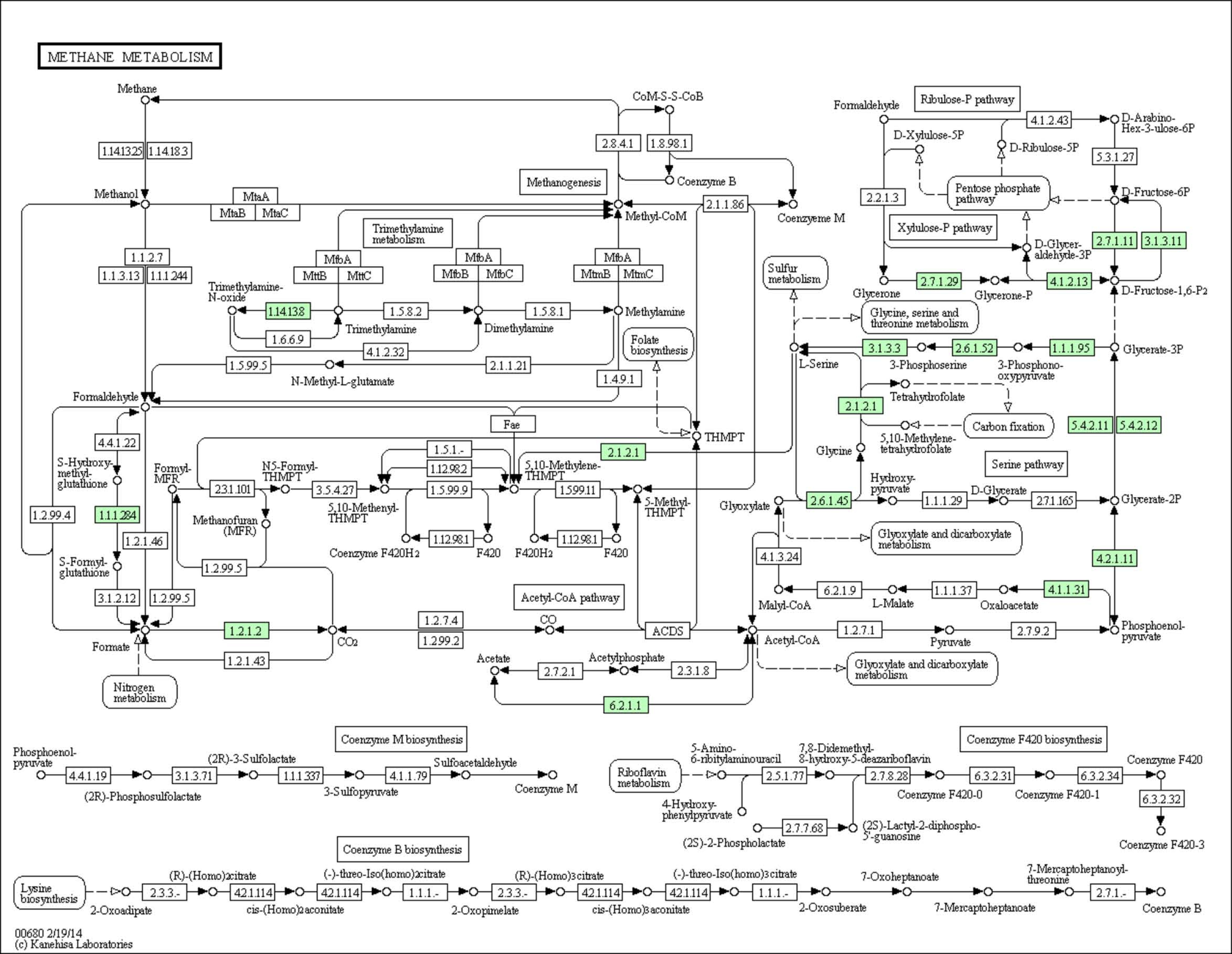

METHANE METABOLISM
Methane
CoM-S-S-CoB
1.14.13.25
1.14.18.3
2.8.4.1
1.8.98.1
Coenzyme B
Methanogenesis
Methanol
MtaA
MtaB
MtaC
Methyl-CoM
2.1.1.86
Coenzyme M
Trimethylamine metabolism
1.1.2.7
1.1.3.13
1.1.1.244
MtbA
MttB
MttC
MtbB
MtbC
MtmB
MtmC
Trimethylamine-N-oxide
1.14.13.8
1.5.8.2
1.5.8.1
Methylamine
Trimethylamine
Dimethylamine
1.6.6.9
4.1.2.32
Folate biosynthesis
1.5.99.5
N-Methyl-L-glutamate
2.1.1.21
1.4.9.1
Formaldehyde
Fae
THMPT
4.4.1.22
S-Hydroxy-methyl-glutathione
Formyl-MFR
N5-Formyl-THMPT
1.5.1.-
1.12.98.2
2.1.2.1
5,10-Methylene-THMPT
2.3.1.101
3.5.4.27
1.5.99.9
1.5.99.11
5-Methyl-THMPT
1.2.99.4
1.1.1.284
Methanofuran (MFR)
5,10-Methenyl-THMPT
1.2.1.46
1.12.98.1
Coenzyme F420H2
F420
F420H2
S-Formyl-glutathione
1.2.99.5
3.1.2.12
Acetyl-CoA pathway
1.2.7.4
1.2.99.2
CO
ACDS
Formate
1.2.1.2
CO2
1.2.1.43
Nitrogen metabolism
Acetate
2.7.2.1
Acetylphosphate
2.3.1.8
6.2.1.1
Formaldehyde
Ribulose-P pathway
4.1.2.43
D-Arabino-Hex-3-ulose-6P
D-Xylulose-5P
D-Ribulose-5P
5.3.1.27
2.2.1.3
Pentose phosphate pathway
D-Fructose-6P
Xylulose-P pathway
D-Glyceraldehyde-3P
2.7.1.11
3.1.3.11
Sulfur metabolism
Glycerone
2.7.1.29
Glycerone-P
4.1.2.13
D-Fructose-1,6-P2
Glycine, serine and threonine metabolism
L-Serine
3.1.3.3
3-Phosphoserine
2.6.1.52
3-Phosphono-oxypyruvate
1.1.1.95
Glycerate-3P
2.1.2.1
Tetrahydrofolate
Carbon fixation
5,10-Methylene-tetrahydrofolate
5.4.2.11
5.4.2.12
Glycine
Serine pathway
Hydroxy-pyruvate
Glyoxylate
2.6.1.45
1.1.1.29
D-Glycerate
2.7.1.165
Glycerate-2P
Glyoxylate and dicarboxylate metabolism
4.1.3.24
4.2.1.11
Malyl-CoA
6.2.1.9
L-Malate
1.1.1.37
Oxaloacetate
4.1.1.31
Acetyl-CoA
1.2.7.1
Pyruvate
2.7.9.2
Phosphoenol-pyruvate
Glyoxylate and dicarboxylate metabolism
Coenzyme M biosynthesis
Phosphoenol-pyruvate
4.4.1.19
(2R)-3-Sulfolactate
3.1.3.71
1.1.1.337
Sulfoacetaldehyde
4.1.1.79
Coenzyme M
(2R)-Phosphosulfolactate
3-Sulfopyruvate
Coenzyme F420 biosynthesis
Riboflavin metabolism
5-Amino-6-ribitylaminouracil
7,8-Didemethyl-8-hydroxy-5-deazariboflavin
2.5.1.77
2.7.8.28
6.3.2.31
6.3.2.34
Coenzyme F420
Coenzyme F420-0
Coenzyme F420-1
6.3.2.32
4-Hydroxy-phenylpyruvate
2.7.7.68
(2S)-Lactyl-2-diphospho-5'-guanosine
(2S)-2-Phospholactate
Coenzyme F420-3
Coenzyme B biosynthesis
Lysine biosynthesis
2.3.3.-
(R)-(Homo)2citrate
4.2.1.114
(-)-threo-Iso(homo)2citrate
1.1.1.-
(R)-(Homo)3citrate
(-)-threo-Iso(homo)3citrate
7-Oxoheptanoate
7-Mercaptoheptanoyl-threonine
2.7.1.-
2-Oxoadipate
cis-(Homo)2aconitate
2-Oxopimelate
cis-(Homo)3aconitate
2-Oxosuberate
7-Mercaptoheptanoate
Coenzyme B
00680 2/19/14
(c) Kanehisa Laboratories

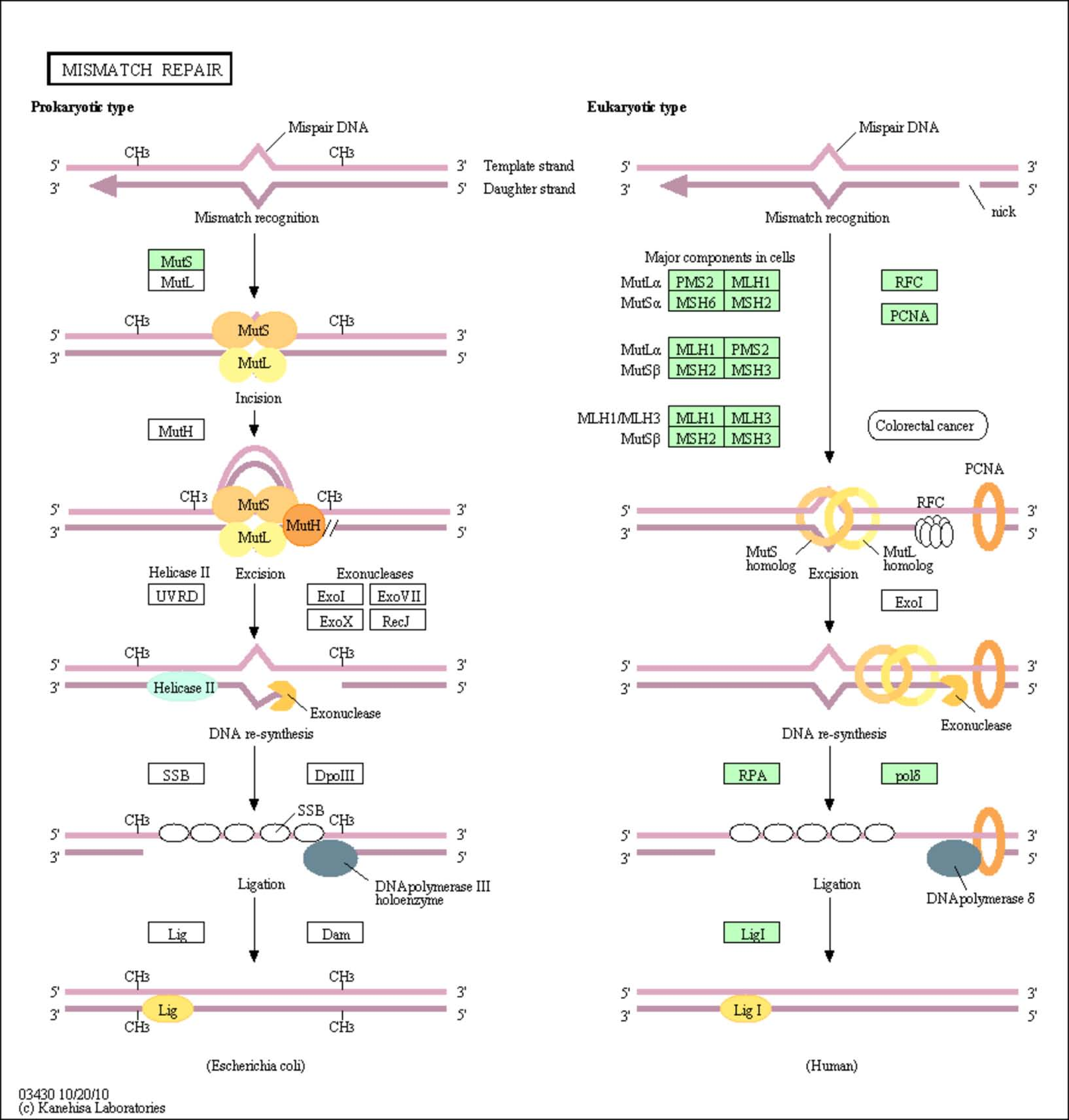

MISMATCH REPAIR
Prokaryotic type
Mispair DNA
CH3
5'
3'
Template strand
Daughter strand
Mismatch recognition
MutS
MutL
Incision
MutH
Helicase II
UVRD
Excision
Exonucleases
ExoI
ExoVII
ExoX
RecJ
Exonuclease
DNA re-synthesis
SSB
DpoIII
Ligation
DNApolymerase III holoenzyme
Lig
Dam
(Escherichia coli)
Eukaryotic type
nick
Major components in cells
MutLα
MutSα
PMS2
MLH1
MSH6
MSH2
MutSβ
MSH3
MLH1/MLH3
MLH3
RFC
PCNA
Colorectal cancer
MutS homolog
MutL homolog
RPA
polδ
DNApolymerase δ
LigI
Lig I
(Human)
03430 10/20/10
(c) Kanehisa Laboratories

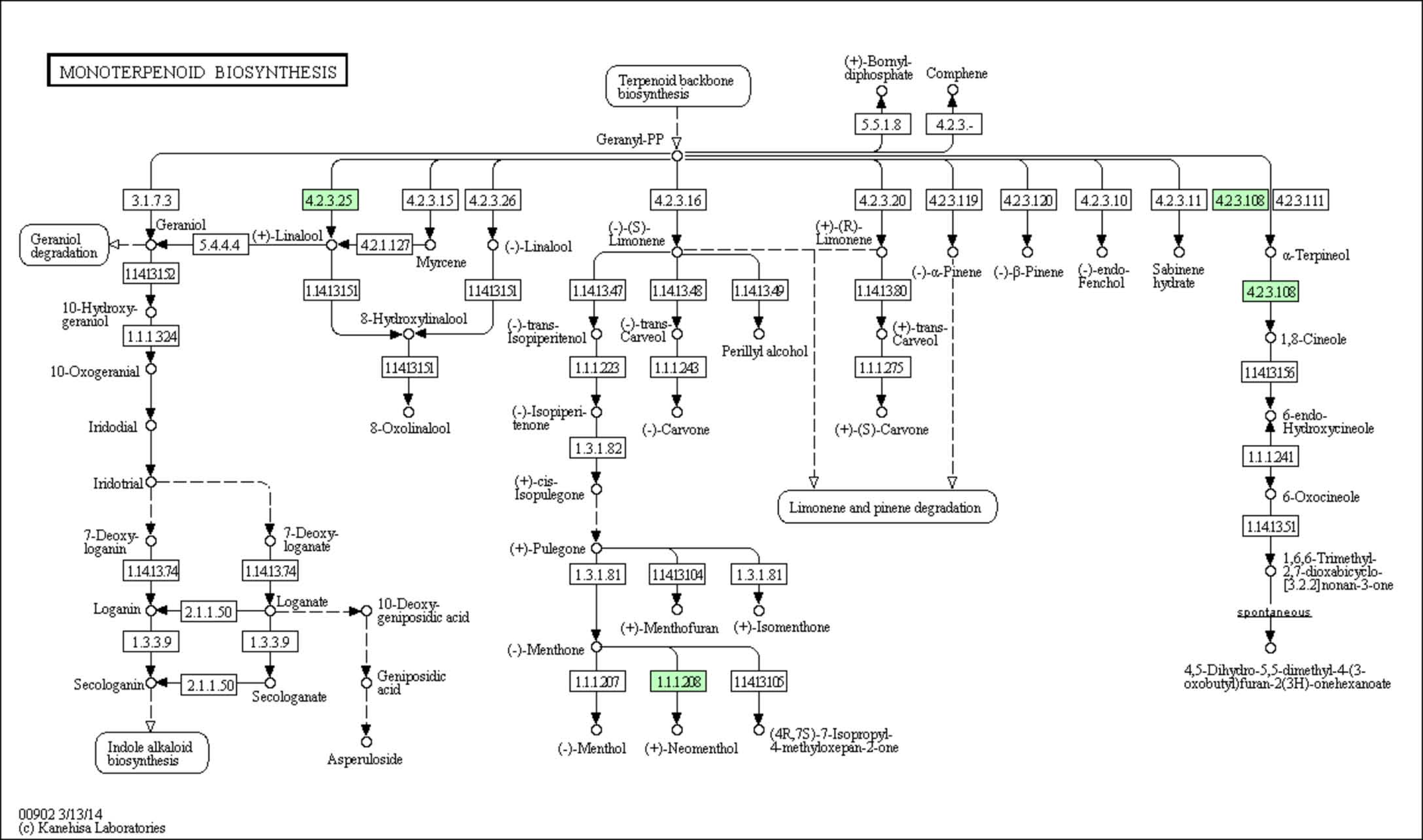
MONOTERPENOID BIOSYNTHESIS
Terpenoid backbone biosynthesis
Geranyl-PP
(+)-Bornyl-diphosphate
Camphene
5.5.1.8
4.2.3.-
3.1.7.3
4.2.3.25
4.2.3.15
4.2.3.26
4.2.3.16
4.2.3.20
4.2.3.119
4.2.3.120
4.2.3.10
4.2.3.11
4.2.3.108
4.2.3.111
Geraniol degradation
Geraniol
5.4.4.4
(+)-Linalool
4.2.1.127
Myrcene
(-)-Linalool
(-)-(S)-Limonene
(+)-(R)-Limonene
(-)-α-Pinene
(-)-β-Pinene
(-)-endo-Fenchol
Sabinene hydrate
α-Terpineol
1.14.13.152
10-Hydroxy-geraniol
1.1.1.324
10-Oxogeranial
Iridodial
Iridotrial
7-Deoxy-loganin
7-Deoxy-loganate
1.14.13.74
Loganin
Loganate
2.1.1.50
10-Deoxy-geniposidic acid
1.3.3.9
Secologanin
Secologanate
Geniposidic acid
Asperuloside
Indole alkaloid biosynthesis
1.14.13.151
8-Hydroxylinalool
8-Oxolinalool
1.14.13.47
1.14.13.48
1.14.13.49
1.14.13.80
(-)-trans-Isopiperitenol
(-)-trans-Carveol
Perillyl alcohol
(+)-trans-Carveol
1.1.1.223
1.1.1.243
1.1.1.275
(-)-Isopiperi-tenone
(-)-Carvone
(+)-(S)-Carvone
1.3.1.82
(+)-cis-Isopulegone
Limonene and pinene degradation
(+)-Pulegone
1.3.1.81
1.14.13.104
(+)-Menthofuran
(+)-Isomenthone
(-)-Menthone
1.1.1.207
1.1.1.208
1.14.13.105
(-)-Menthol
(+)-Neomenthol
(4R,7S)-7-Isopropyl-4-methyloxepan-2-one
1,8-Cineole
1.14.13.156
6-endo-Hydroxycineole
1.1.1.241
6-Oxocineole
1.14.13.51
1,6,6-Trimethyl-2,7-dioxabicyclo-[3.2.2]nonan-3-one
spontaneous
4,5-Dihydro-5,5-dimethyl-4-(3-oxobutyl)furan-2(3H)-onehexanoate
00902 3/13/14
(c) Kanehisa Laboratories

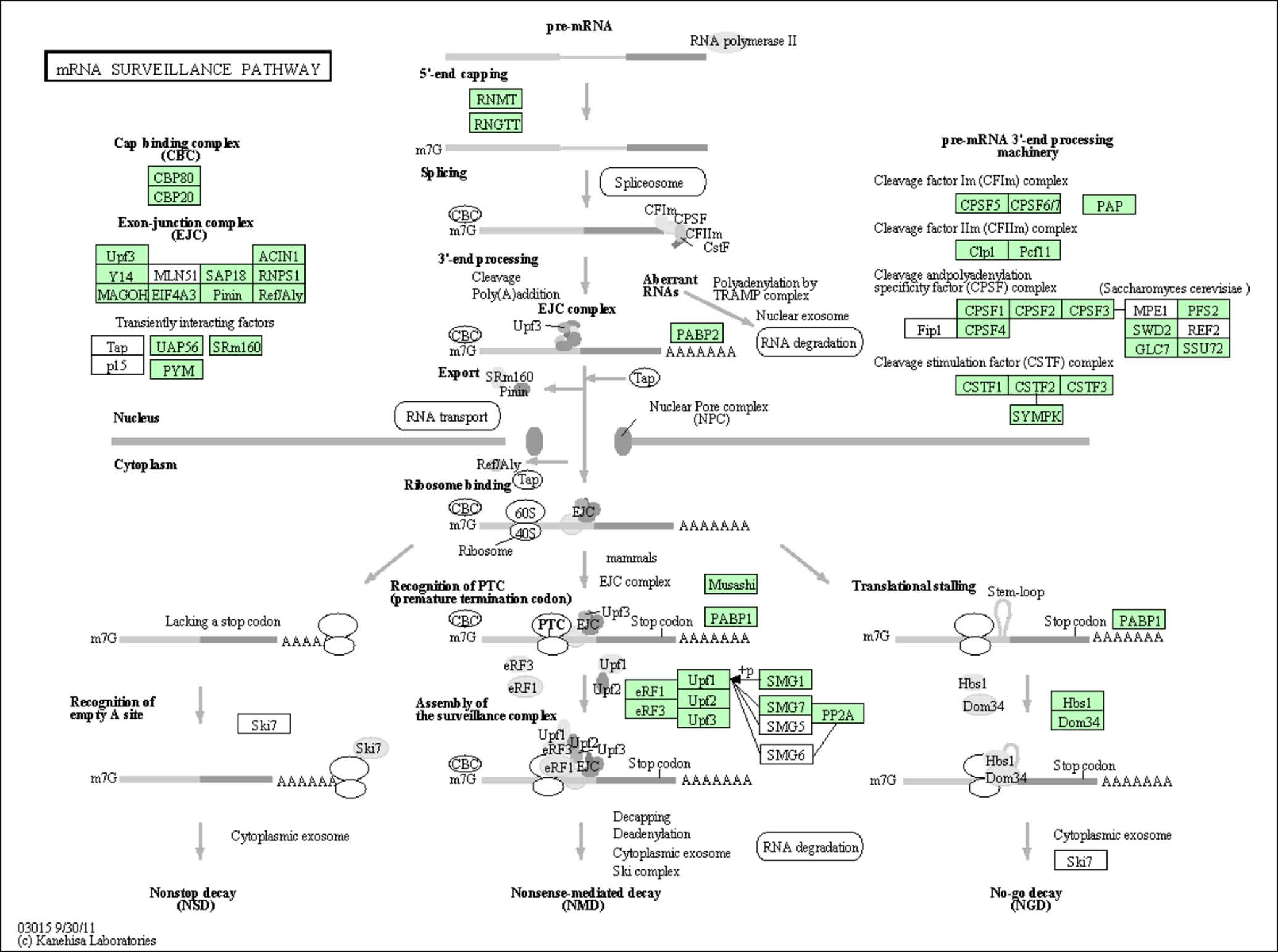

mRNA SURVEILLANCE PATHWAY
pre-mRNA
RNA polymerase II
5'-end capping
RNMT
RNGTT
m7G
Splicing
Spliceosome
CBC
CFIm
CPSF
CFIIm
CstF
3'-end processing
Cleavage
Poly(A)addition
EJC complex
Upf3
PABP2
AAAAAAA
Aberrant RNAs
Polyadenylation by TRAMP complex
Nuclear exosome
RNA degradation
Export
SRm160
Pinin
Tap
RNA transport
Nuclear Pore complex (NPC)
Nucleus
Cytoplasm
Ref/Aly
Ribosome binding
60S
40S
EJC
Ribosome
Cap binding complex (CBC)
CBP80
CBP20
Exon-junction complex (EJC)
Upf3
ACIN1
Y14
MLN51
SAP18
RNPS1
MAGOH
EIF4A3
Pinin
Ref/Aly
Transiently interacting factors
Tap
p15
UAP56
SRm160
PYM
pre-mRNA 3'-end processing machinery
Cleavage factor Im (CFIm) complex
CPSF5
CPSF6/7
PAP
Cleavage factor IIm (CFIIm) complex
Clp1
Pcf11
Cleavage andpolyadenylation specificity factor (CPSF) complex
(Saccharomyces cerevisiae)
CPSF1
CPSF2
CPSF3
MPE1
PFS2
Fip1
CPSF4
SWD2
REF2
GLC7
SSU72
Cleavage stimulation factor (CSTF) complex
CSTF1
CSTF2
CSTF3
SYMPK
mammals
EJC complex
Musashi
PABP1
Recognition of PTC (premature termination codon)
PTC
Stop codon
Upf1
eRF3
eRF1
Upf2
SMG1
+p
SMG7
SMG5
PP2A
SMG6
Assembly of the surveillance complex
Lacking a stop codon
AAAA
Recognition of empty A site
Ski7
AAAAAA
Cytoplasmic exosome
Nonstop decay (NSD)
Decapping
Deadenylation
Cytoplasmic exosome
Ski complex
Nonsense-mediated decay (NMD)
Translational stalling
Stem-loop
Hbs1
Dom34
No-go decay (NGD)
03015 9/30/11
(c) Kanehisa Laboratories

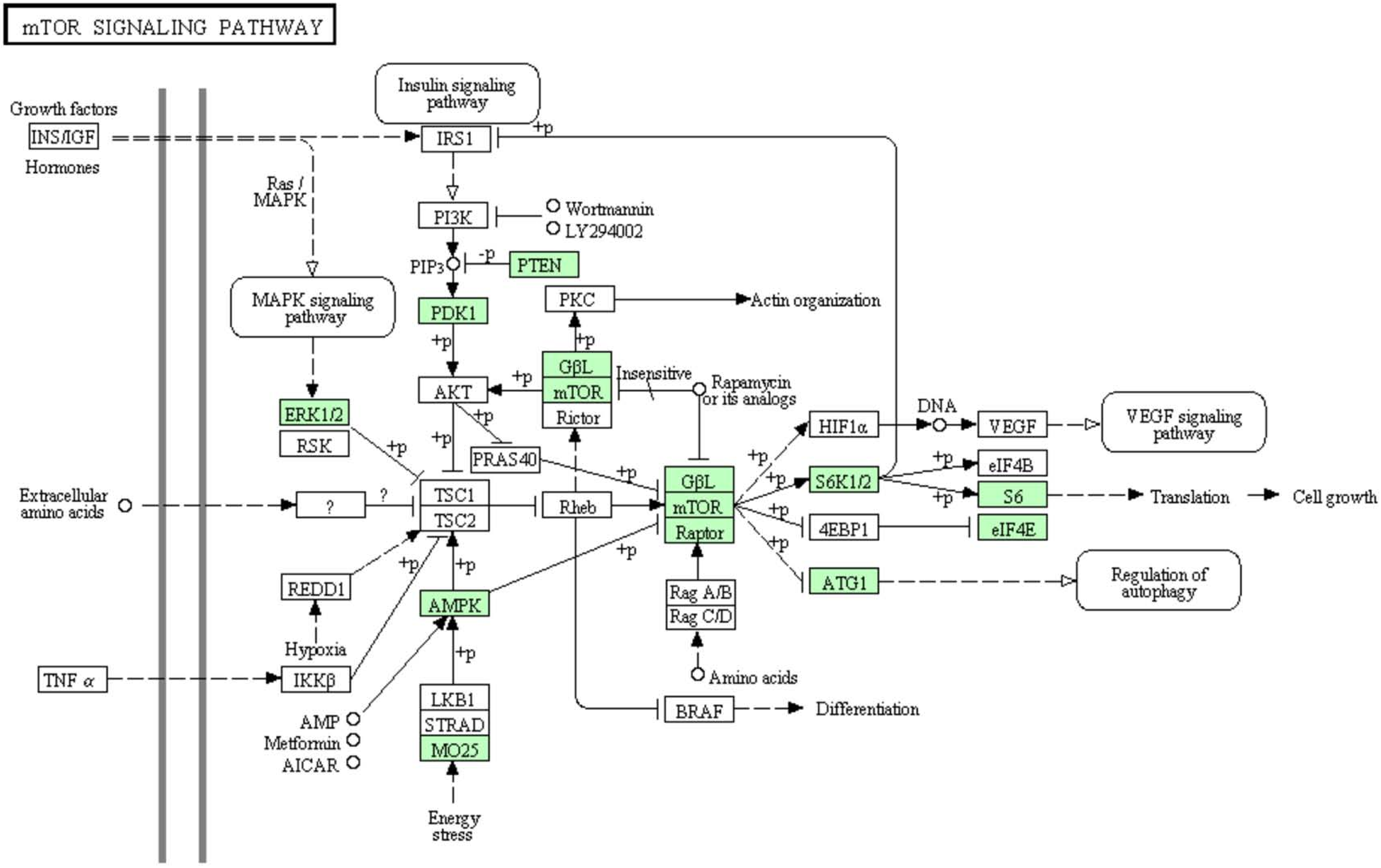

04150 5/30/13
(c) Kanehisa Laboratories

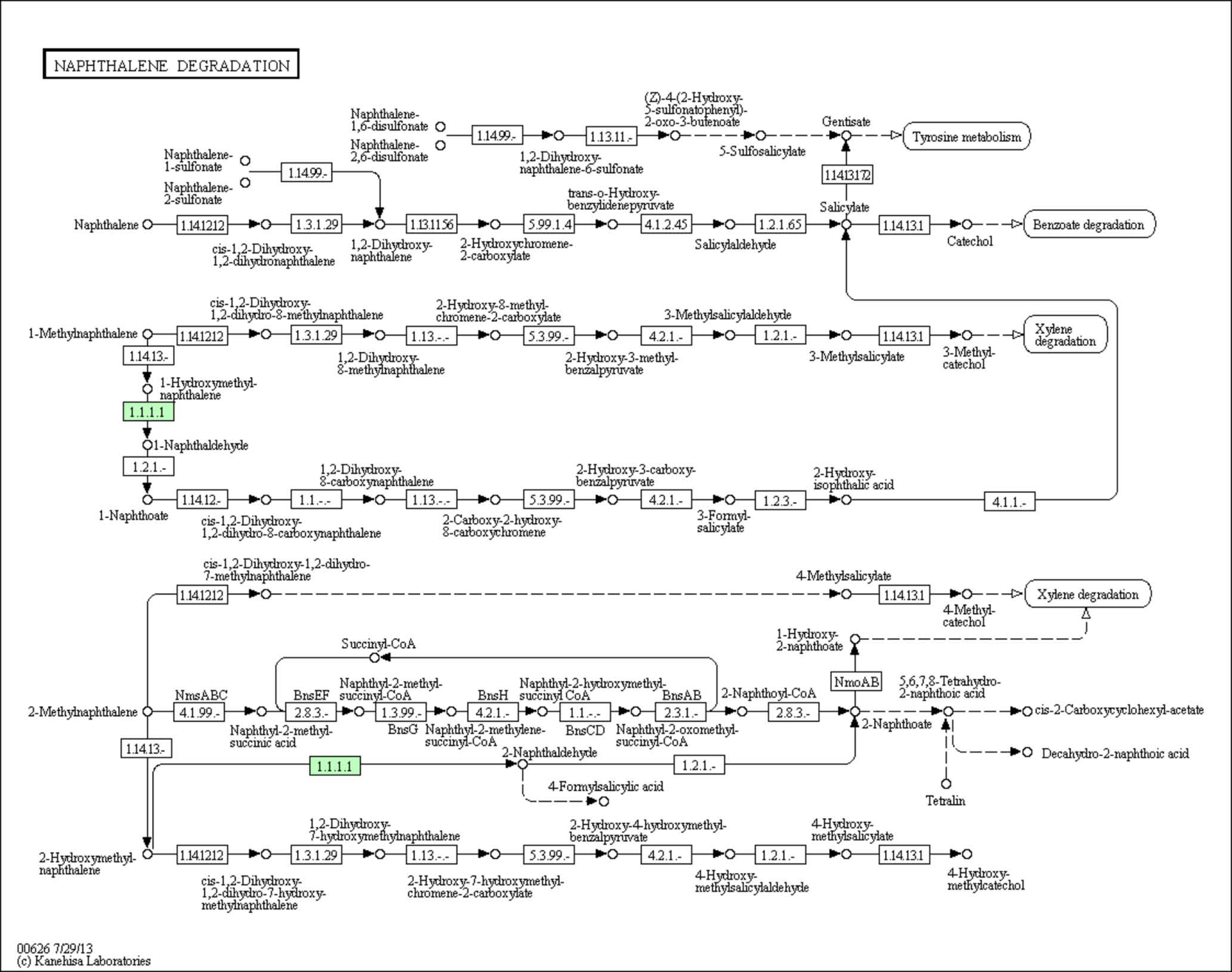
NAPHTHALENE DEGRADATION
Naphthalene-1,6-disulfonate
Naphthalene-2,6-disulfonate
1.14.99.-
1,2-Dihydroxy-naphthalene-6-sulfonate
1.13.11.-
(Z)-4-(2-Hydroxy-5-sulfonatophenyl)-2-oxo-3-butenoate
5-Sulfosalicylate
Gentisate
Tyrosine metabolism
Naphthalene-1-sulfonate
Naphthalene-2-sulfonate
1.14.99.-
1.14.13.172
Naphthalene
1.14.12.12
1.3.1.29
cis-1,2-Dihydroxy-1,2-dihydronaphthalene
1,2-Dihydroxy-naphthalene
1.13.11.56
2-Hydroxychromene-2-carboxylate
5.99.1.4
trans-o-Hydroxy-benzylidenepyruvate
4.1.2.45
Salicylaldehyde
1.2.1.65
Salicylate
1.14.13.1
Catechol
Benzoate degradation
cis-1,2-Dihydroxy-1,2-dihydro-8-methylnaphthalene
2-Hydroxy-8-methyl-chromene-2-carboxylate
3-Methylsalicylaldehyde
1-Methylnaphthalene
1.14.12.12
1.3.1.29
1.13.-.-
5.3.99.-
4.2.1.-
1.2.1.-
1.14.13.1
Xylene degradation
1,2-Dihydroxy-8-methylnaphthalene
2-Hydroxy-3-methyl-benzalpyruvate
3-Methylsalicylate
3-Methyl-catechol
1.14.13.-
1-Hydroxymethyl-naphthalene
1.1.1.1
1-Naphthaldehyde
1.2.1.-
1,2-Dihydroxy-8-carboxynaphthalene
2-Hydroxy-3-carboxy-benzalpyruvate
2-Hydroxy-isophthalic acid
1-Naphthoate
1.14.12.-
1.1.-.-
1.13.-.-
5.3.99.-
4.2.1.-
1.2.3.-
4.1.1.-
cis-1,2-Dihydroxy-1,2-dihydro-8-carboxynaphthalene
2-Carboxy-2-hydroxy-8-carboxychromene
3-Formyl-salicylate
cis-1,2-Dihydroxy-1,2-dihydro-7-methylnaphthalene
1.14.12.12
4-Methylsalicylate
1.14.13.1
4-Methyl-catechol
Xylene degradation
1-Hydroxy-2-naphthoate
Succinyl-CoA
NmoAB
2-Methylnaphthalene
NmsABC
4.1.99.-
Naphthyl-2-methyl-succinic acid
BnsEF
2.8.3.-
Naphthyl-2-methyl-succinyl-CoA
1.3.99.-
BnsG
BnsH
4.2.1.-
Naphthyl-2-methylene-succinyl-CoA
Naphthyl-2-hydroxymethyl-succinyl CoA
1.1.-.-
BnsCD
BnsAB
2.3.1.-
Naphthyl-2-oxomethyl-succinyl-CoA
2-Naphthoyl-CoA
2.8.3.-
2-Naphthoate
5,6,7,8-Tetrahydro-2-naphthoic acid
cis-2-Carboxycyclohexyl-acetate
Decahydro-2-naphthoic acid
Tetralin
1.14.13.-
1.1.1.1
2-Naphthaldehyde
1.2.1.-
4-Formylsalicylic acid
2-Hydroxymethyl-naphthalene
1.14.12.12
1,2-Dihydroxy-7-hydroxymethylnaphthalene
1.3.1.29
1.13.-.-
5.3.99.-
2-Hydroxy-4-hydroxymethyl-benzalpyruvate
4.2.1.-
1.2.1.-
4-Hydroxy-methylsalicylate
1.14.13.1
cis-1,2-Dihydroxy-1,2-dihydro-7-hydroxy-methylnaphthalene
2-Hydroxy-7-hydroxymethyl-chromene-2-carboxylate
4-Hydroxy-methylsalicylaldehyde
4-Hydroxy-methylcatechol
00626 7/29/13
(c) Kanehisa Laboratories

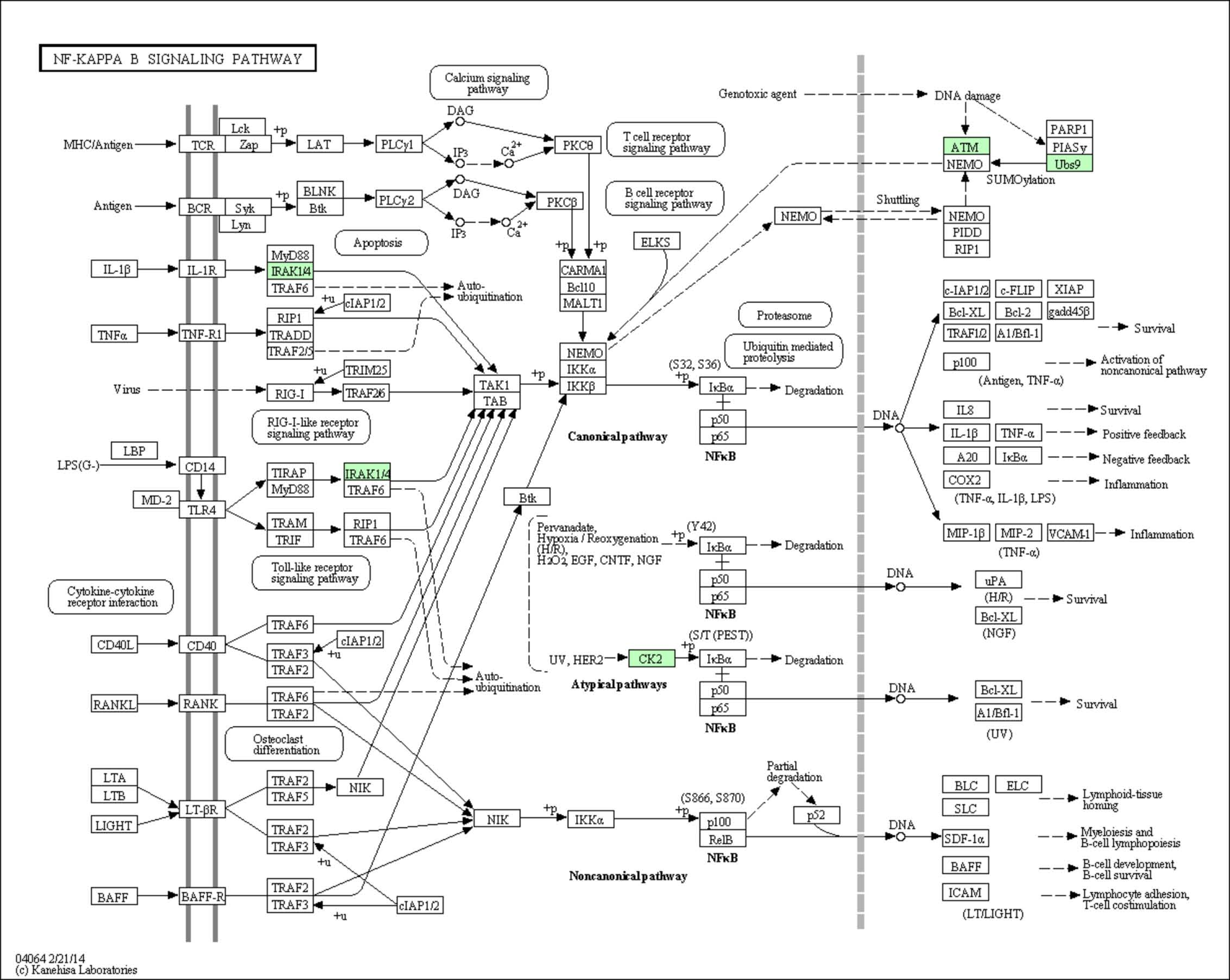

NF-KAPPA B SIGNALING PATHWAY
Calcium signaling pathway
T cell receptor signaling pathway
B cell receptor signaling pathway
Apoptosis
RIG-I-like receptor signaling pathway
Toll-like receptor signaling pathway
Cytokine-cytokine receptor interaction
Osteoclast differentiation
Proteasome
Ubiquitin mediated proteolysis
MHC/Antigen
TCR
Lck
Zap
+p
LAT
PLCγ1
DAG
IP3
Ca2+
PKCθ
Antigen
BCR
Syk
Lyn
BLNK
Btk
PLCγ2
PKCβ
IL-1β
IL-1R
MyD88
IRAK1/4
TRAF6
Auto-ubiquitination
+u
cIAP1/2
TNFα
TNF-R1
RIP1
TRADD
TRAF2/5
Virus
RIG-I
TRIM25
TRAF2/6
TAK1
TAB
LBP
LPS(G-)
CD14
MD-2
TLR4
TIRAP
MyD88
IRAK1/4
TRAF6
TRAM
TRIF
RIP1
TRAF6
CARMA1
Bcl10
MALT1
ELKS
NEMO
IKKα
IKKβ
Btk
Canonical pathway
(S32, S36)
IκBα
Degradation
p50
p65
NFκB
Genotoxic agent
DNA damage
ATM
NEMO
PARP1
PIASy
Ubs9
SUMOylation
Shuttling
NEMO
PIDD
RIP1
c-IAP1/2
c-FLIP
XIAP
Bcl-XL
Bcl-2
gadd45β
TRAF1/2
A1/Bfl-1
Survival
p100
(Antigen, TNF-α)
Activation of noncanonical pathway
DNA
IL8
IL-1β
TNF-α
A20
IκBα
COX2
(TNF-α, IL-1β, LPS)
Positive feedback
Negative feedback
Inflammation
MIP-1β
MIP-2
VCAM-1
(TNF-α)
Pervanadate, Hypoxia / Reoxygenation (H/R), H2O2, EGF, CNTF, NGF
(Y42)
uPA
(H/R)
Bcl-XL
(NGF)
UV, HER2
CK2
(S/T (PEST))
Atypical pathways
Bcl-XL
A1/Bfl-1
(UV)
CD40L
CD40
TRAF6
TRAF3
TRAF2
RANKL
RANK
LTA
LTB
LIGHT
LT-βR
TRAF2
TRAF5
NIK
TRAF2
TRAF3
BAFF
BAFF-R
NIK
IKKα
(S866, S870)
p100
RelB
Partial degradation
p52
Noncanonical pathway
BLC
ELC
SLC
SDF-1α
BAFF
ICAM
(LT/LIGHT)
Lymphoid-tissue homing
Myeloiesis and B-cell lymphopoiesis
B-cell development, B-cell survival
Lymphocyte adhesion, T-cell costimulation
04064 2/21/14
(c) Kanehisa Laboratories

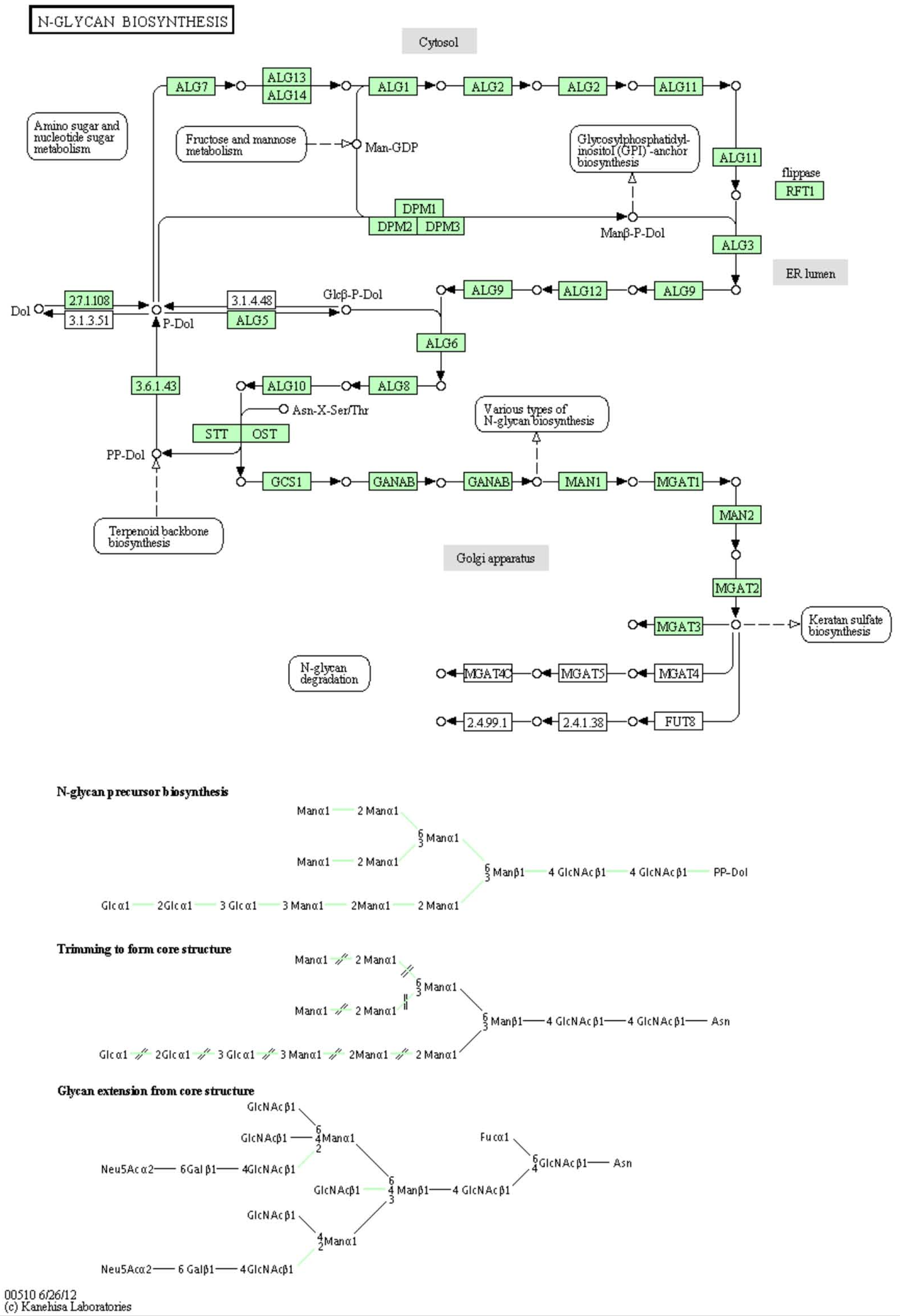
N-GLYCAN BIOSYNTHESIS
Cytosol
ER lumen
Golgi apparatus
Amino sugar and nucleotide sugar metabolism
Fructose and mannose metabolism
Glycosylphosphatidyl-inositol (GPI) -anchor biosynthesis
Various types of N-glycan biosynthesis
Terpenoid backbone biosynthesis
N-glycan degradation
Keratan sulfate biosynthesis
ALG7
ALG13
ALG14
ALG1
ALG2
ALG2
ALG11
ALG11
flippase
RFT1
Man-GDP
DPM1
DPM2
DPM3
Manβ-P-Dol
ALG3
ALG9
ALG12
ALG9
Dol
2.7.1.108
3.1.3.51
P-Dol
3.1.4.48
ALG5
Glcβ-P-Dol
ALG6
3.6.1.43
ALG10
ALG8
Asn-X-Ser/Thr
STT
OST
PP-Dol
GCS1
GANAB
GANAB
MAN1
MGAT1
MAN2
MGAT2
MGAT3
MGAT4C
MGAT5
MGAT4
2.4.99.1
2.4.1.38
FUT8
N-glycan precursor biosynthesis
Manα1 2 Manα1
6/3 Manα1
Manα1 2 Manα1
6/3 Manβ1 4 GlcNAcβ1 4 GlcNAcβ1 PP-Dol
Glcα1 2Glcα1 3 Glcα1 3 Manα1 2Manα1 2 Manα1
Trimming to form core structure
Manα1 2 Manα1
6/3 Manα1
Manα1 2 Manα1
6/3 Manβ1 4 GlcNAcβ1 4 GlcNAcβ1 Asn
Glcα1 2Glcα1 3 Glcα1 3 Manα1 2Manα1 2 Manα1
Glycan extension from core structure
GlcNAcβ1
GlcNAcβ1 6/4/2 Manα1
Neu5Acα2 6Galβ1 4GlcNAcβ1
Fucα1
6/4 GlcNAcβ1 Asn
GlcNAcβ1 6/4/3 Manβ1 4 GlcNAcβ1
GlcNAcβ1
4/2 Manα1
Neu5Acα2 6 Galβ1 4GlcNAcβ1
00510 6/26/12
(c) Kanehisa Laboratories

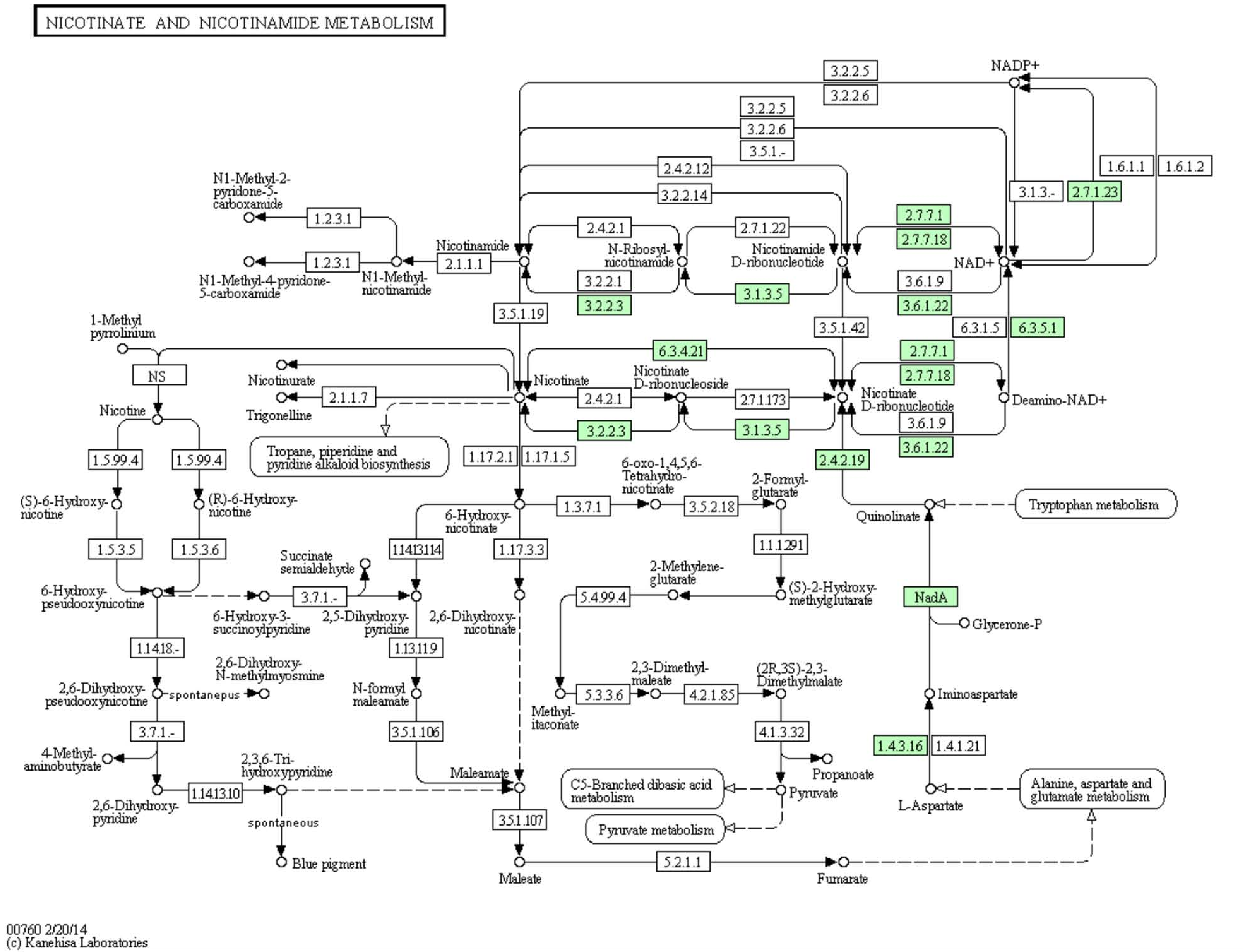
NICOTINATE AND NICOTINAMIDE METABOLISM
NADP+
3.2.2.5
3.2.2.6
3.2.2.5
3.2.2.6
3.5.1.-
2.4.2.12
3.2.2.14
1.6.1.1
1.6.1.2
3.1.3.-
2.7.1.23
N1-Methyl-2-pyridone-5-carboxamide
1.2.3.1
1.2.3.1
N1-Methyl-4-pyridone-5-carboxamide
N1-Methyl-nicotinamide
2.1.1.1
Nicotinamide
2.4.2.1
N-Ribosyl-nicotinamide
2.7.1.22
Nicotinamide D-ribonucleotide
2.7.7.1
2.7.7.18
NAD+
3.2.2.1
3.2.2.3
3.1.3.5
3.6.1.9
3.6.1.22
3.5.1.19
3.5.1.42
6.3.1.5
6.3.5.1
1-Methyl pyrrolinium
NS
Nicotine
Nicotinurate
Trigonelline
2.1.1.7
Nicotinate
6.3.4.21
Nicotinate D-ribonucleoside
2.4.2.1
2.7.1.173
Nicotinate D-ribonucleotide
2.7.7.1
2.7.7.18
Deamino-NAD+
3.2.2.3
3.1.3.5
3.6.1.9
3.6.1.22
2.4.2.19
Tropane, piperidine and pyridine alkaloid biosynthesis
1.5.99.4
1.5.99.4
1.17.2.1
1.17.1.5
(S)-6-Hydroxy-nicotine
(R)-6-Hydroxy-nicotine
6-oxo-1,4,5,6-Tetrahydro-nicotinate
2-Formyl-glutarate
1.3.7.1
3.5.2.18
Quinolinate
Tryptophan metabolism
6-Hydroxy-nicotinate
1.5.3.5
1.5.3.6
Succinate semialdehyde
1.14.13.114
1.17.3.3
1.1.1.291
2-Methylene-glutarate
6-Hydroxy-pseudooxynicotine
3.7.1.-
5.4.99.4
(S)-2-Hydroxy-methylglutarate
NadA
Glycerone-P
6-Hydroxy-3-succinoylpyridine
2,5-Dihydroxy-pyridine
2,6-Dihydroxy-nicotinate
1.14.18.-
1.13.11.9
2,6-Dihydroxy-N-methylmyosmine
2,3-Dimethyl-maleate
(2R,3S)-2,3-Dimethylmalate
2,6-Dihydroxy-pseudooxynicotine
spontanepus
N-formyl maleamate
5.3.3.6
4.2.1.85
Iminoaspartate
Methyl-itaconate
3.7.1.-
3.5.1.106
4.1.3.32
1.4.3.16
1.4.1.21
4-Methyl-aminobutyrate
2,3,6-Tri-hydroxypyridine
Maleamate
Propanoate
C5-Branched dibasic acid metabolism
Pyruvate
L-Aspartate
Alanine, aspartate and glutamate metabolism
2,6-Dihydroxy-pyridine
1.14.13.10
spontaneous
3.5.1.107
Pyruvate metabolism
Blue pigment
Maleate
5.2.1.1
Fumarate
00760 2/20/14
(c) Kanehisa Laboratories

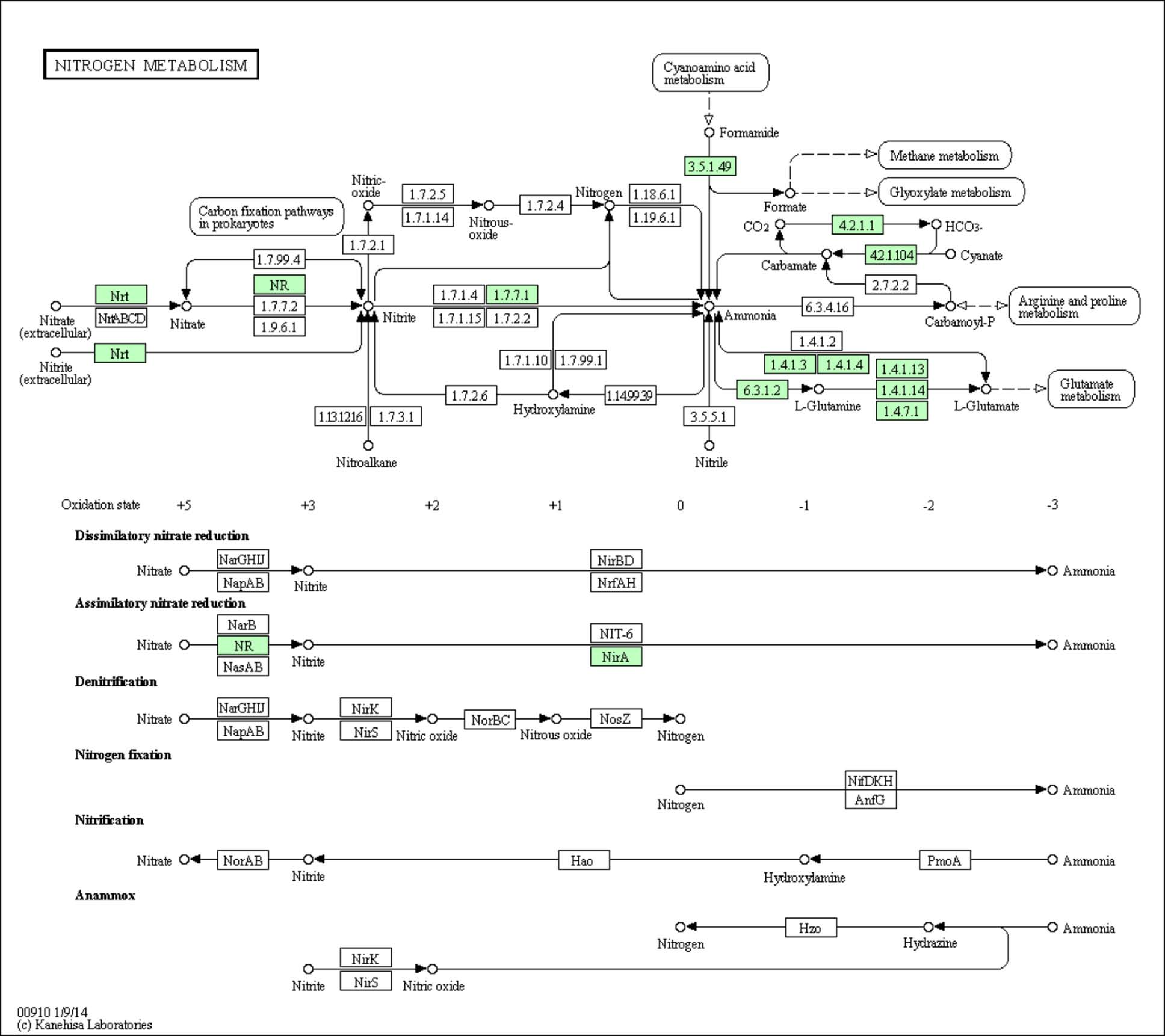
NITROGEN METABOLISM
Cyanoamino acid metabolism
Formamide
3.5.1.49
Methane metabolism
Glyoxylate metabolism
Formate
Nitric-oxide
1.7.2.5
1.7.1.14
Nitrous-oxide
1.7.2.4
Nitrogen
1.18.6.1
1.19.6.1
Carbon fixation pathways in prokaryotes
1.7.2.1
CO2
4.2.1.1
HCO3-
Carbamate
4.2.1.104
Cyanate
1.7.99.4
2.7.2.2
Nrt
NR
1.7.1.4
1.7.7.1
6.3.4.16
Arginine and proline metabolism
Nitrate (extracellular)
NrtABCD
Nitrate
1.7.7.2
1.9.6.1
Nitrite
1.7.1.15
1.7.2.2
Ammonia
Carbamoyl-P
Nitrite (extracellular)
Nrt
1.4.1.2
1.7.1.10
1.7.99.1
1.4.1.3
1.4.1.4
1.4.1.13
6.3.1.2
L-Glutamine
1.4.1.14
L-Glutamate
Glutamate metabolism
1.7.2.6
Hydroxylamine
1.14.99.39
1.4.7.1
1.13.12.16
1.7.3.1
3.5.5.1
Nitroalkane
Nitrile
Oxidation state
+5
+3
+2
+1
0
-1
-2
-3
Dissimilatory nitrate reduction
Nitrate
NarGHIJ
NapAB
Nitrite
NirBD
NrfAH
Ammonia
Assimilatory nitrate reduction
Nitrate
NarB
NR
NasAB
Nitrite
NIT-6
NirA
Ammonia
Denitrification
Nitrate
NarGHIJ
NapAB
Nitrite
NirK
NirS
Nitric oxide
NorBC
Nitrous oxide
NosZ
Nitrogen
Nitrogen fixation
Nitrogen
NifDKH
AnfG
Ammonia
Nitrification
Nitrate
NorAB
Nitrite
Hao
Hydroxylamine
PmoA
Ammonia
Anammox
Nitrogen
Hzo
Hydrazine
Ammonia
Nitrite
NirK
NirS
Nitric oxide
00910 1/9/14
(c) Kanehisa Laboratories

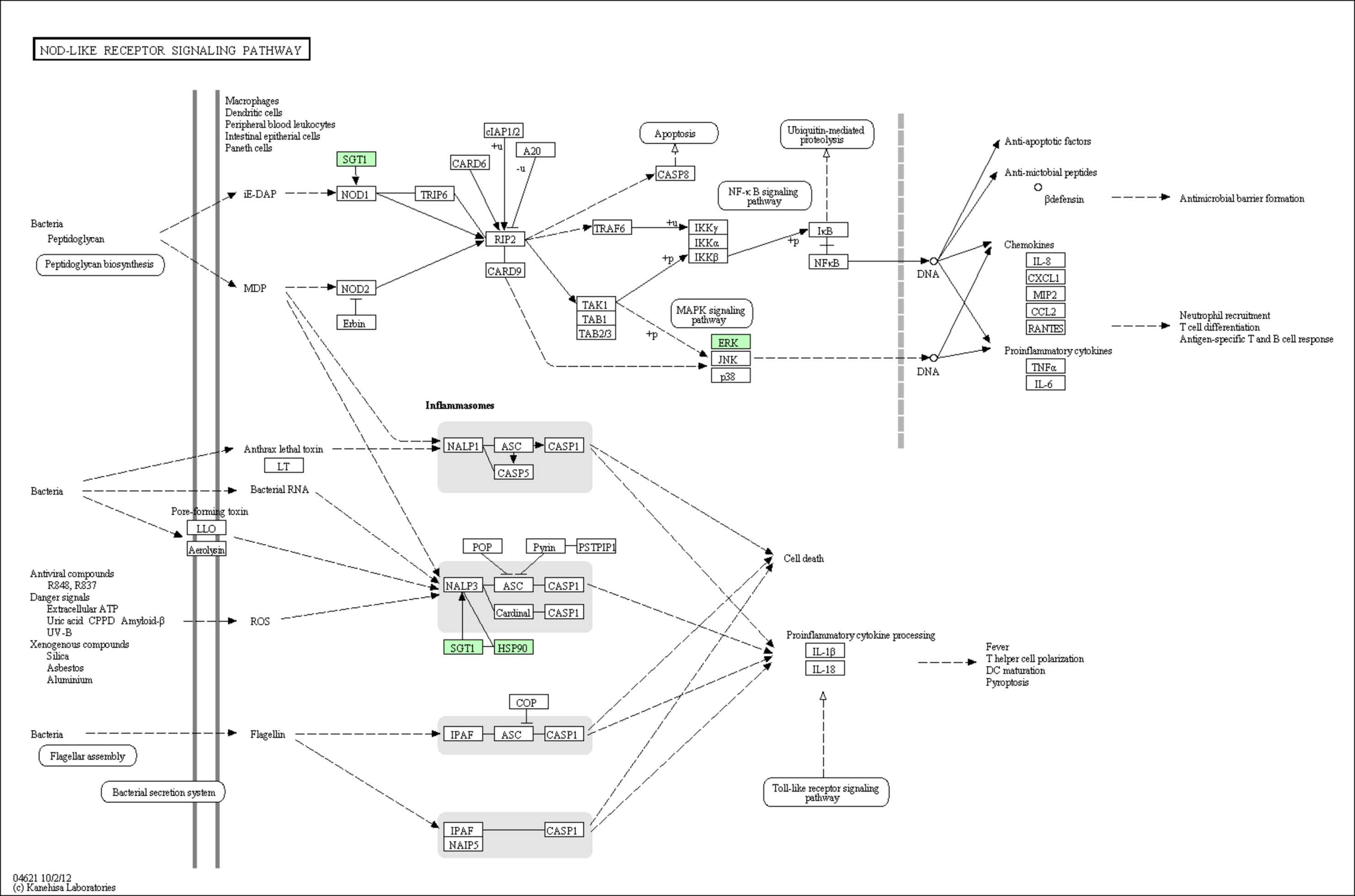
NOD-LIKE RECEPTOR SIGNALING PATHWAY
Macrophages
Dendritic cells
Peripheral blood leukocytes
Intestinal epitherial cells
Paneth cells
Bacteria
Peptidoglycan
Peptidoglycan biosynthesis
iE-DAP
MDP
SGT1
NOD1
NOD2
Erbin
TRIP6
CARD6
cIAP1/2
A20
+u
-u
RIP2
CARD9
Apoptosis
CASP8
TRAF6
TAK1
TAB1
TAB2/3
NF-κ B signaling pathway
IKKγ
IKKα
IKKβ
Ubiquitin-mediated proteolysis
IκB
NFκB
+p
MAPK signaling pathway
ERK
JNK
p38
DNA
Anti-apoptotic factors
Anti-mictobial peptides
βdefensin
Antimicrobial barrier formation
Chemokines
IL-8
CXCL1
MIP2
CCL2
RANTES
Neutrophil recruitment
T cell differentiation
Antigen-specific T and B cell response
Proinflammatory cytokines
TNFα
IL-6
Inflammasomes
Anthrax lethal toxin
LT
NALP1
ASC
CASP1
CASP5
Bacteria
Bacterial RNA
Pore-forming toxin
LLO
Aerolysin
POP
Pyrin
PSTPIP1
NALP3
Cardinal
HSP90
Antiviral compounds
R848, R837
Danger signals
Extracellular ATP
Uric acid CPPD Amyloid-β
UV-B
Xenogenous compounds
Silica
Asbestos
Aluminium
ROS
Cell death
Proinflammatory cytokine processing
IL-1β
IL-18
Fever
T helper cell polarization
DC maturation
Pyroptosis
COP
IPAF
Flagellin
Flagellar assembly
Bacterial secretion system
NAIP5
Toll-like receptor signaling pathway
04621 10/2/12
(c) Kanehisa Laboratories

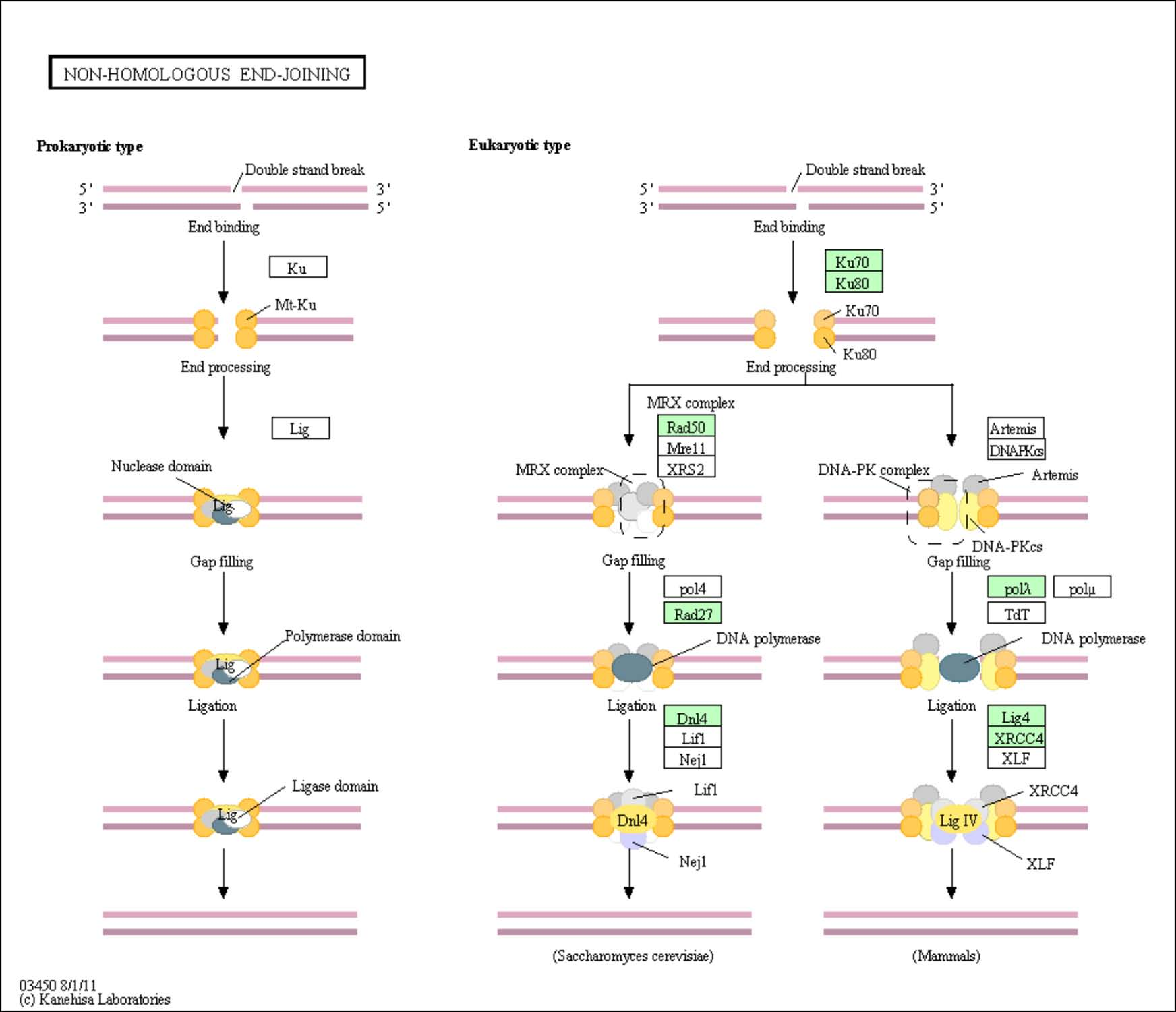
NON-HOMOLOGOUS END-JOINING
Prokaryotic type
Double strand break
5'
3'
3'
5'
End binding
Ku
Mt-Ku
End processing
Lig
Nuclease domain
Lig
Gap filling
Polymerase domain
Lig
Ligation
Ligase domain
Lig
Eukaryotic type
Double strand break
5'
3'
3'
5'
End binding
Ku70
Ku80
Ku70
Ku80
End processing
MRX complex
Rad50
Mre11
XRS2
MRX complex
Artemis
DNAPKcs
DNA-PK complex
Artemis
DNA-PKcs
Gap filling
pol4
Rad27
DNA polymerase
Gap filling
polλ
polμ
TdT
DNA polymerase
Ligation
Dnl4
Lif1
Nej1
Lif1
Dnl4
Nej1
Ligation
Lig4
XRCC4
XLF
XRCC4
Lig IV
XLF
(Saccharomyces cerevisiae)
(Mammals)
03450 8/1/11
(c) Kanehisa Laboratories

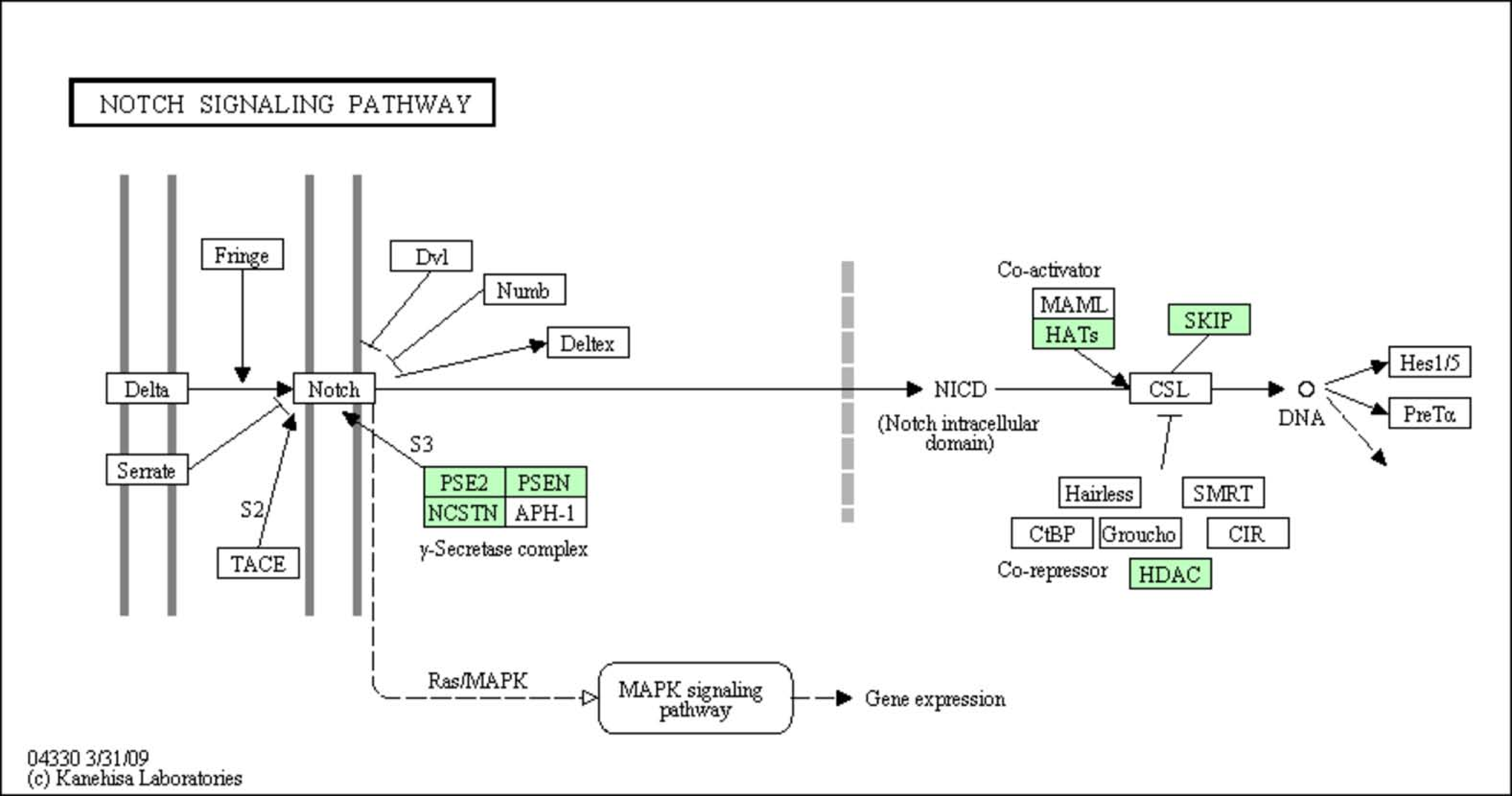
NOTCH SIGNALING PATHWAY
Fringe
Dvl
Numb
Deltex
Delta
Notch
Serrate
S2
TACE
S3
PSE2
PSEN
NCSTN
APH-1
γ-Secretase complex
NICD
(Notch intracellular domain)
Co-activator
MAML
HATs
SKIP
CSL
DNA
Hes1/5
PreTα
Hairless
SMRT
CtBP
Groucho
CIR
Co-repressor
HDAC
Ras/MAPK
MAPK signaling pathway
Gene expression
04330 3/31/09
(c) Kanehisa Laboratories

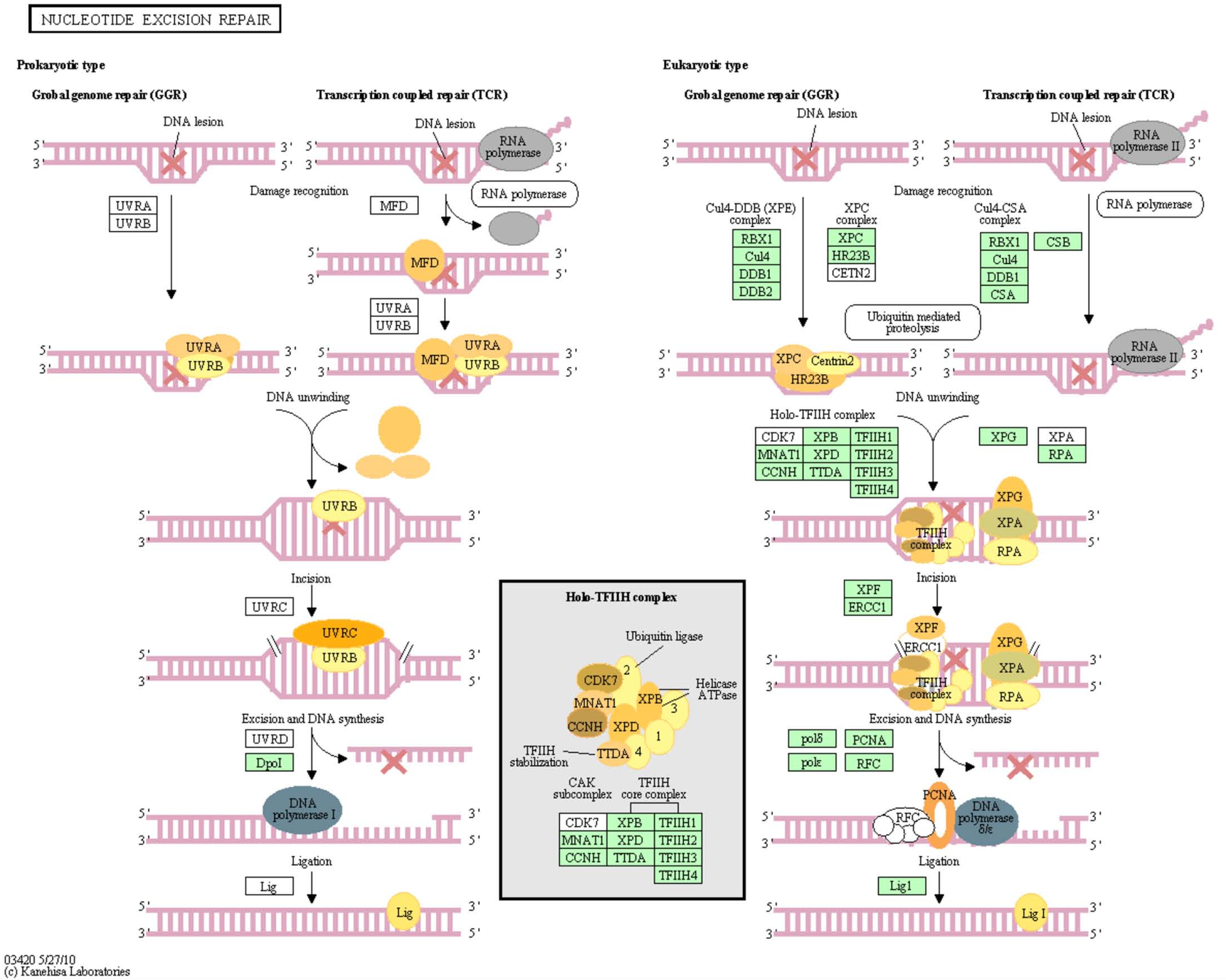

NUCLEOTIDE EXCISION REPAIR
Prokaryotic type
Grobal genome repair (GGR)
Transcription coupled repair (TCR)
DNA lesion
RNA polymerase
Damage recognition
UVRA
UVRB
MFD
DNA unwinding
Incision
UVRC
Excision and DNA synthesis
UVRD
DpoI
DNA polymerase I
Ligation
Lig
Eukaryotic type
Cul4-DDB (XPE) complex
RBX1
Cul4
DDB1
DDB2
XPC complex
XPC
HR23B
CETN2
Cul4-CSA complex
CSA
CSB
RNA polymerase II
Ubiquitin mediated proteolysis
Centrin2
Holo-TFIIH complex
CDK7
XPB
TFIIH1
MNAT1
XPD
TFIIH2
CCNH
TTDA
TFIIH3
TFIIH4
XPG
XPA
RPA
TFIIH complex
XPF
ERCC1
polδ
polε
PCNA
RFC
DNA polymerase δ/ε
Lig1
Lig I
Ubiquitin ligase
Helicase ATPase
TFIIH stabilization
CAK subcomplex
TFIIH core complex
03420 5/27/10
(c) Kanehisa Laboratories

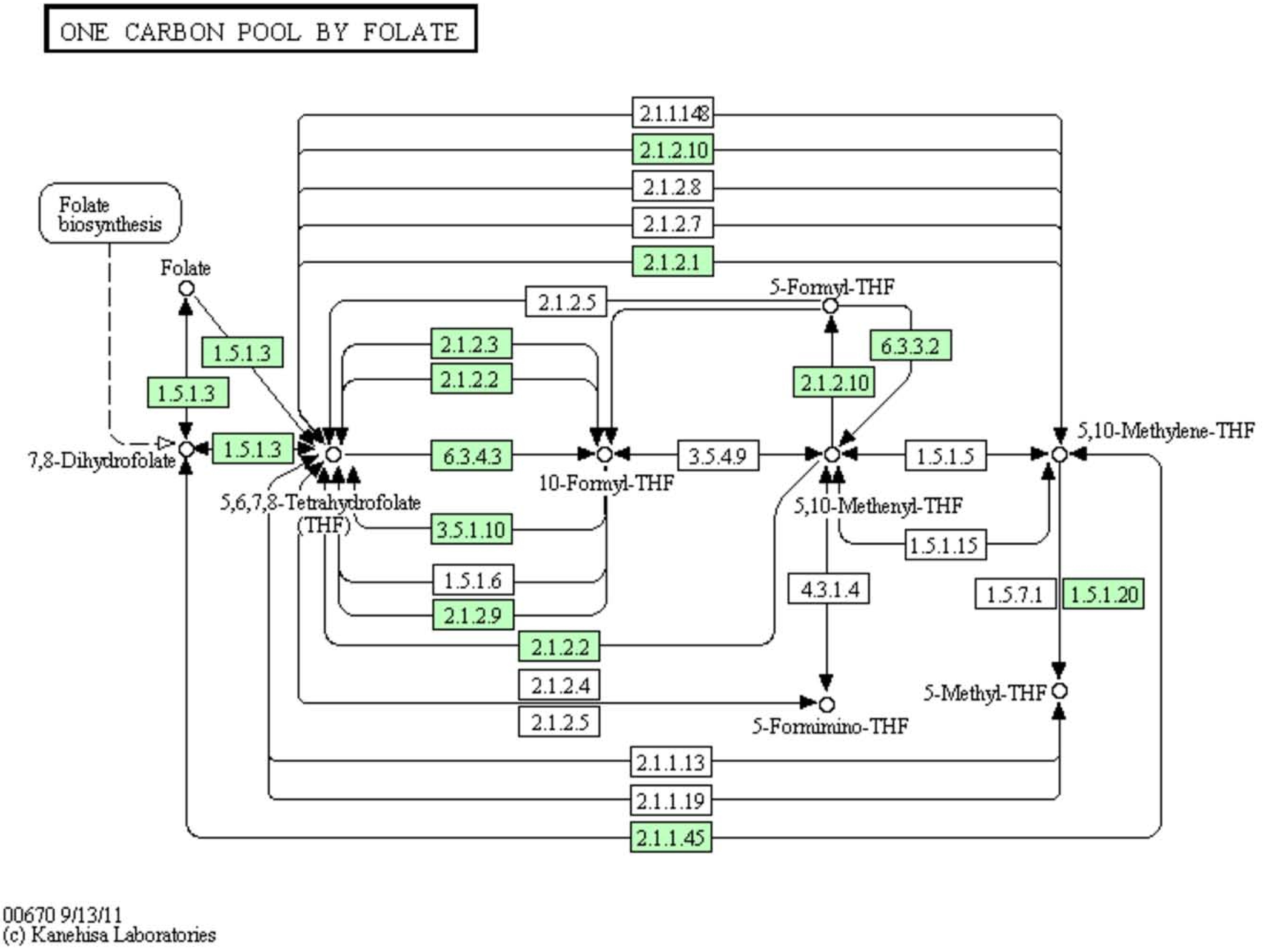
ONE CARBON POOL BY FOLATE
2.1.1.148
2.1.2.10
2.1.2.8
2.1.2.7
2.1.2.1
Folate biosynthesis
Folate
1.5.1.3
1.5.1.3
1.5.1.3
7,8-Dihydrofolate
2.1.2.5
5-Formyl-THF
6.3.3.2
2.1.2.3
2.1.2.2
2.1.2.10
6.3.4.3
3.5.4.9
1.5.1.5
5,10-Methylene-THF
10-Formyl-THF
5,6,7,8-Tetrahydrofolate (THF)
5,10-Methenyl-THF
3.5.1.10
1.5.1.15
1.5.1.6
4.3.1.4
1.5.7.1
1.5.1.20
2.1.2.9
2.1.2.2
2.1.2.4
2.1.2.5
5-Formimino-THF
5-Methyl-THF
2.1.1.13
2.1.1.19
2.1.1.45
00670 9/13/11
(c) Kanehisa Laboratories

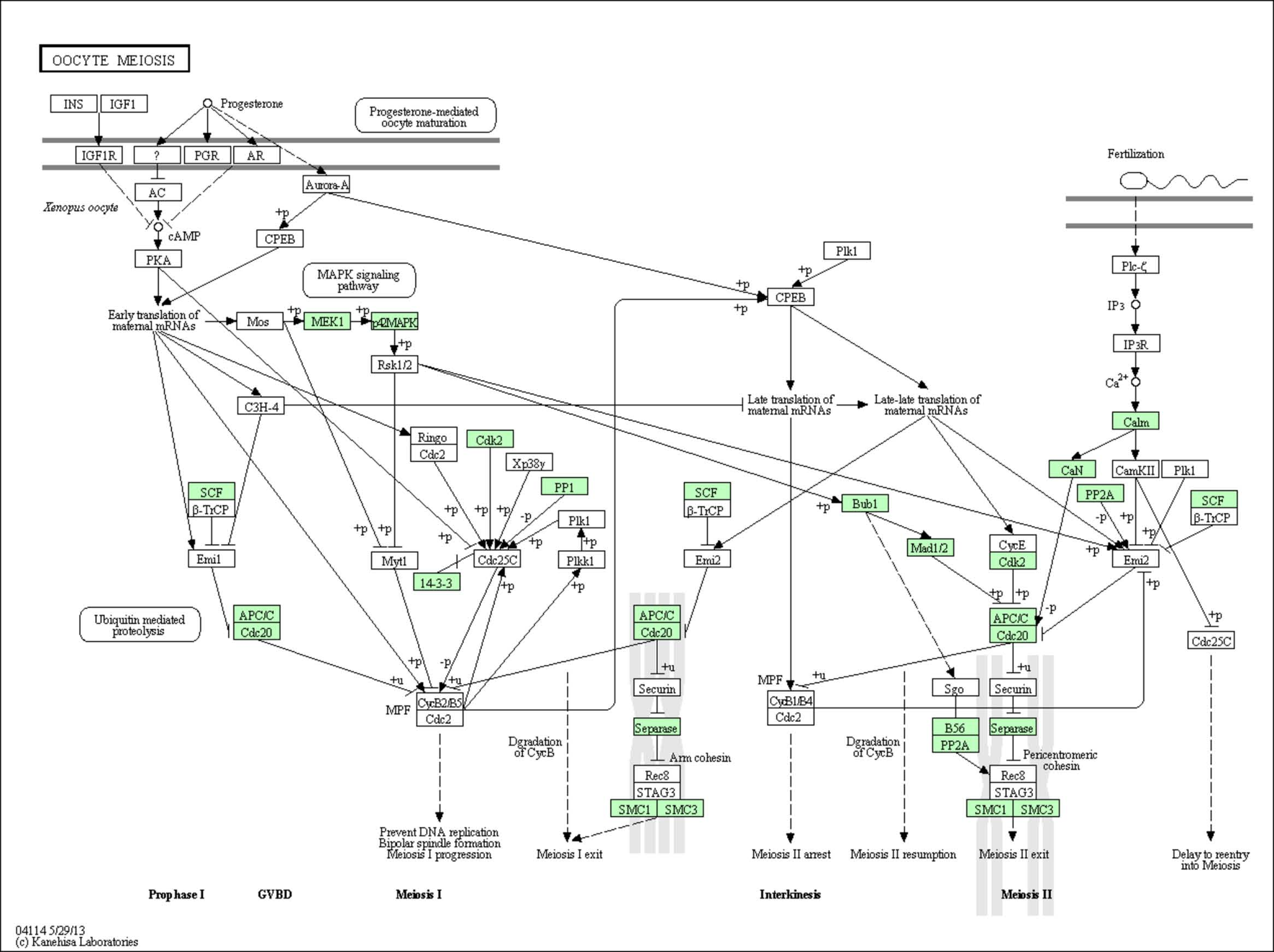
OOCYTE MEIOSIS
INS
IGF1
Progesterone
Progesterone-mediated oocyte maturation
IGF1R
?
PGR
AR
AC
Aurora-A
Xenopus oocyte
cAMP
CPEB
PKA
MAPK signaling pathway
Early translation of maternal mRNAs
Mos
MEK1
p42MAPK
Rsk1/2
C3H-4
Ringo
Cdc2
Cdk2
Xp38γ
PP1
Plk1
SCF
β-TrCP
Emi1
Myt1
Cdc25C
Plkk1
14-3-3
APC/C
Cdc20
Ubiquitin mediated proteolysis
CycB2/B5
MPF
Prevent DNA replication
Bipolar spindle formation
Meiosis I progression
Dgradation of CycB
Meiosis I exit
Securin
Separase
Arm cohesin
Rec8
STAG3
SMC1
SMC3
Fertilization
Plc-ζ
IP3
IP3R
Ca2+
Calm
CaN
CamKII
PP2A
Late translation of maternal mRNAs
Late-late translation of maternal mRNAs
Bub1
Mad1/2
CycE
Emi2
CycB1/B4
Sgo
B56
Pericentromeric cohesin
Meiosis II arrest
Meiosis II resumption
Meiosis II exit
Delay to reentry into Meiosis
Prophase I
GVBD
Meiosis I
Interkinesis
Meiosis II
04114 5/29/13
(c) Kanehisa Laboratories

OTHER GLYCAN DEGRADATION

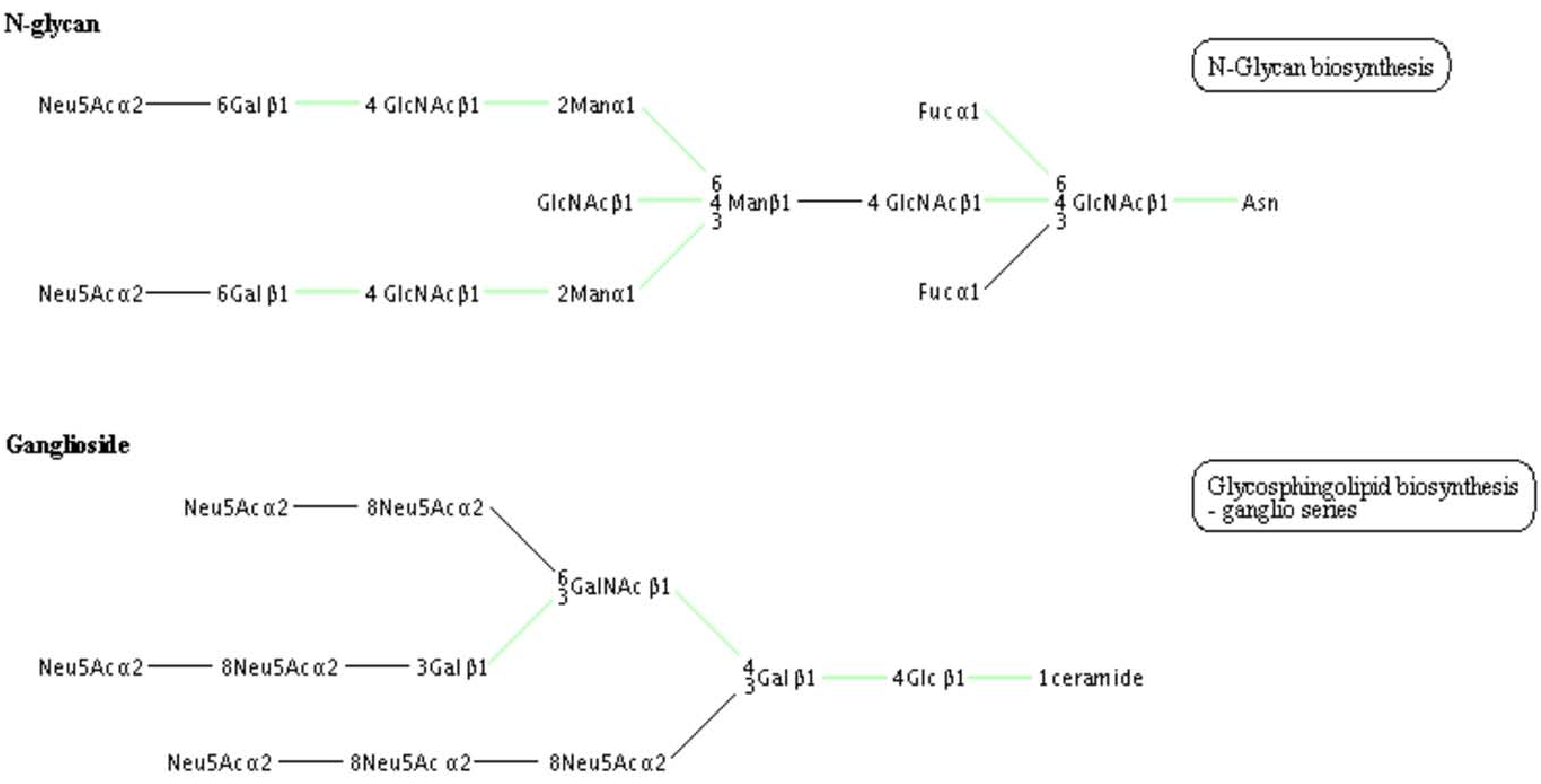

00511 6/3/13
(c) Kanehisa Laboratories

OXIDATIVE PHOSPHORYLATION

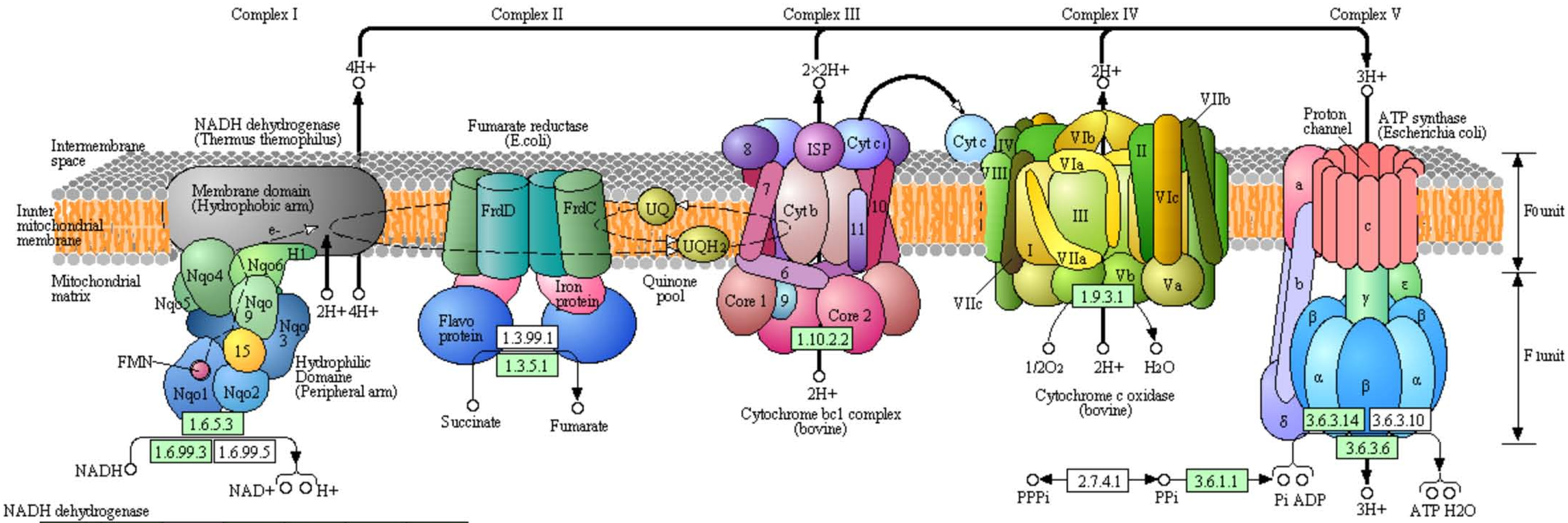

NADH dehydrogenase

E	ND1	ND2	ND3	ND4	ND4L	ND5	ND6										
E	Ndufs1	Ndufs2	Ndufs3	Ndufs4	Ndufs5	Ndufs6	Ndufs7	Ndufs8	Ndufv1	Ndufv2	Ndufv3						
B/A	NuoA	NuoB	NuoC	NuoD	NuoE	NuoF	NuoG	NuoH	NuoI	NuoJ	NuoK	NuoL	NuoM	NuoN			
B/A	NdhC	NdhK	NdhJ	NdhH	NdhA	NdhI	NdhG	NdhE	NdhF	NdhD	NdhB	NdhL	NdhM	NdhN	HoxE	HoxF	HoxU
E	Ndufa1	Ndufa2	Ndufa3	Ndufa4	Ndufa5	Ndufa6	Ndufa7	Ndufa8	Ndufa9	Ndufa10	Ndufab1	Ndufa11	Ndufa12	Ndufa13			
E	Ndufb1	Ndufb2	Ndufb3	Ndufb4	Ndufb5	Ndufb6	Ndufb7	Ndufb8	Ndufb9	Ndufb10	Ndufb11	Ndufc1	Ndufc2				

Succinate dehydrogenase / Fumarate reductase

E	SDHC	SDHD	SDHA	SDHB		
B/A	SdhC	SdhD	SdhA	SdhB		
			FrdA	FrdB	FrdC	FrdD

Cytochrome c reductase

E/B/A	ISP	Cytb	Cyt1				
E	COR1	QCR2	QCR6	QCR7	QCR8	QCR9	QCR10

Cytochrome c oxidase

E	COX10		COX3	COX1	COX2	COX4	COX5A	COX5B	COX6A	COX6B	COX6C	COX7A	COX7B	COX7C	COX8
E/B/A	COX11	COX15												
	COX17													
B/A	CyoE	CyoD	CyoC	CyoB	CyoA									
		CoxD	CoxC	CoxA	CoxB									
		QoxD	QoxC	QoxB	QoxA									

Cytochrome c oxidase , cbb3-type

B	I	II	IV	III

Cytochrome bd complex

B/A	CydA	CydB

F-type ATPase (Bacteria)

beta	alpha	gamma	delta	epsilon	c	a	b

F-type ATPase (Eukaryotes)

beta	alpha	gamma	OSCP	delta	epsilon	c	a
b	e	f6	f	8			
d	f	h	j	k	g		

V-type ATPase (Prokaryotes)

A	B	C	D	E	F	I	K

V-type ATPase (Eukaryotes)

A	B	C	D	E	F	G	H
I	AC39	54kD	S1	lipid			

00190 6/1/12

(c) Kanehisa Laboratories

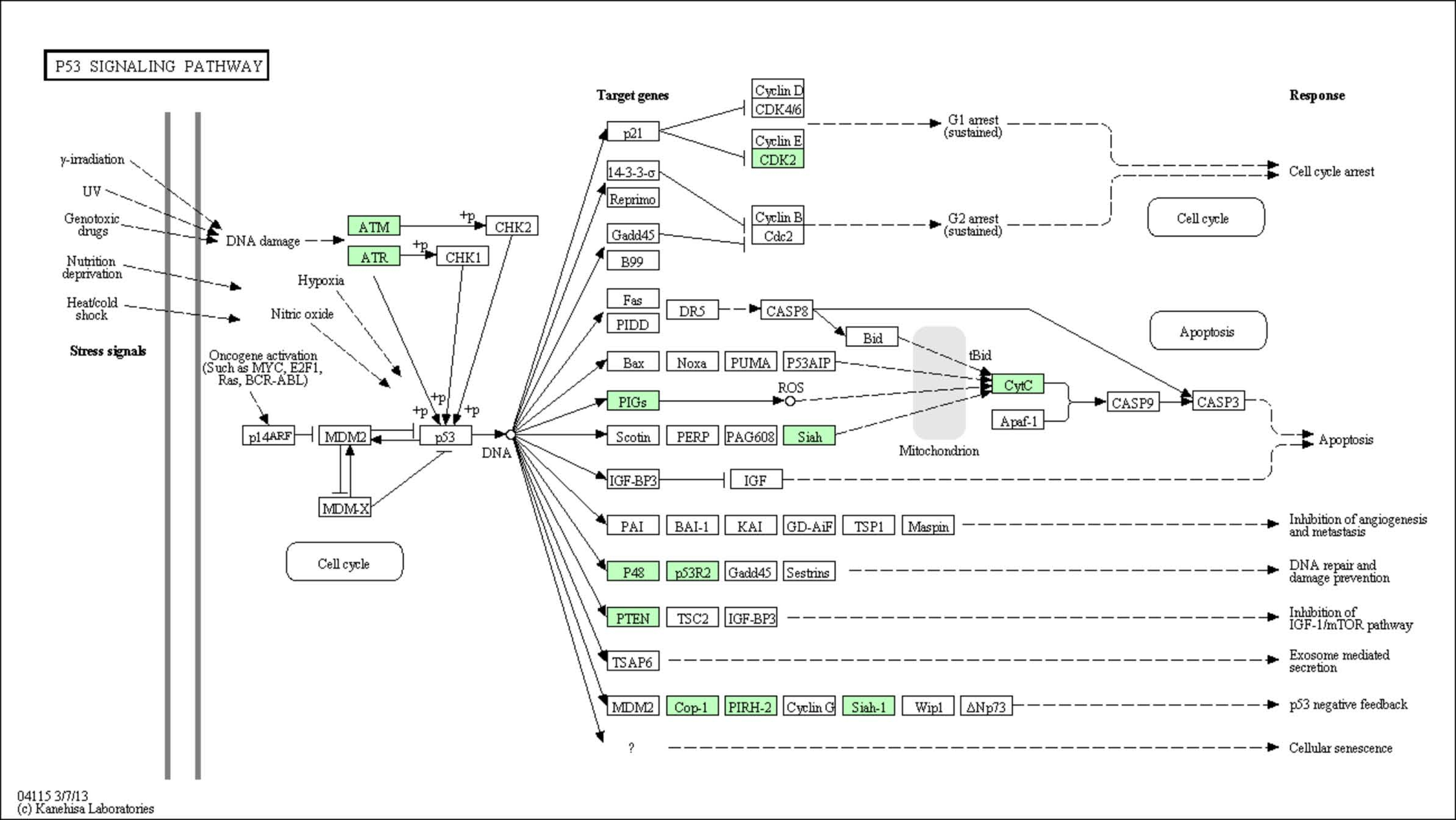
P53 SIGNALING PATHWAY
Target genes
Response
γ-irradiation
UV
Genotoxic drugs
DNA damage
Nutrition deprivation
Heat/cold shock
Hypoxia
Nitric oxide
Stress signals
Oncogene activation (Such as MYC, E2F1, Ras, BCR-ABL)
ATM
ATR
CHK2
CHK1
+p
p14ARF
MDM2
p53
DNA
MDM-X
Cell cycle
p21
14-3-3-σ
Reprimo
Gadd45
B99
Fas
PIDD
DR5
CASP8
Bid
Bax
Noxa
PUMA
P53AIP
PIGs
ROS
Scotin
PERP
PAG608
Siah
tBid
CytC
Apaf-1
CASP9
CASP3
Mitochondrion
IGF-BP3
IGF
Cyclin D
CDK4/6
Cyclin E
CDK2
Cyclin B
Cdc2
G1 arrest (sustained)
G2 arrest (sustained)
Cell cycle arrest
Cell cycle
Apoptosis
Apoptosis
PAI
BAI-1
KAI
GD-AiF
TSP1
Maspin
Inhibition of angiogenesis and metastasis
P48
p53R2
Gadd45
Sestrins
DNA repair and damage prevention
PTEN
TSC2
IGF-BP3
Inhibition of IGF-1/mTOR pathway
TSAP6
Exosome mediated secretion
MDM2
Cop-1
PIRH-2
Cyclin G
Siah-1
Wip1
ΔNp73
p53 negative feedback
?
Cellular senescence
04115 3/7/13
(c) Kanehisa Laboratories

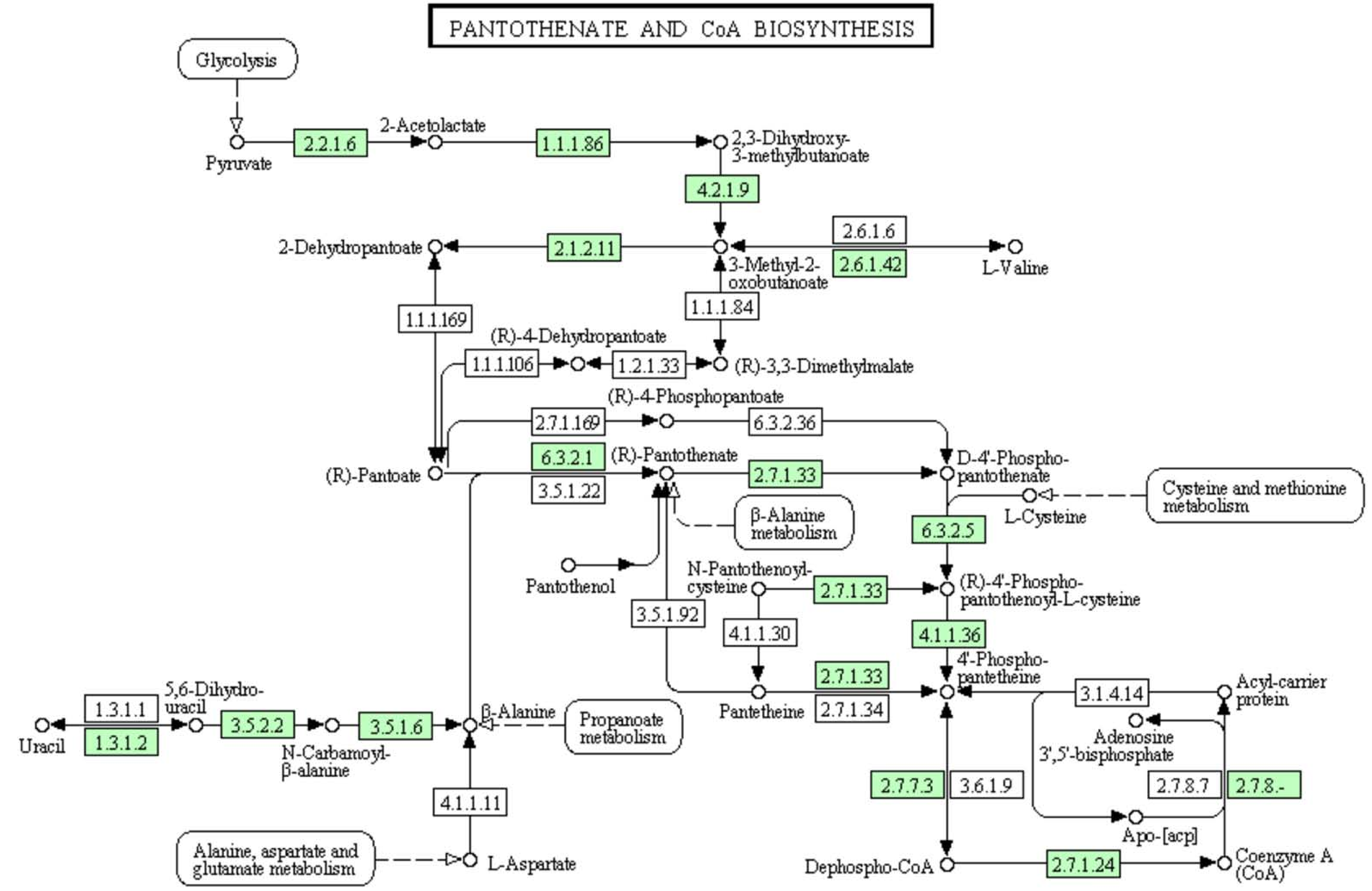

00770 6/8/12
(c) Kanehisa Laboratories

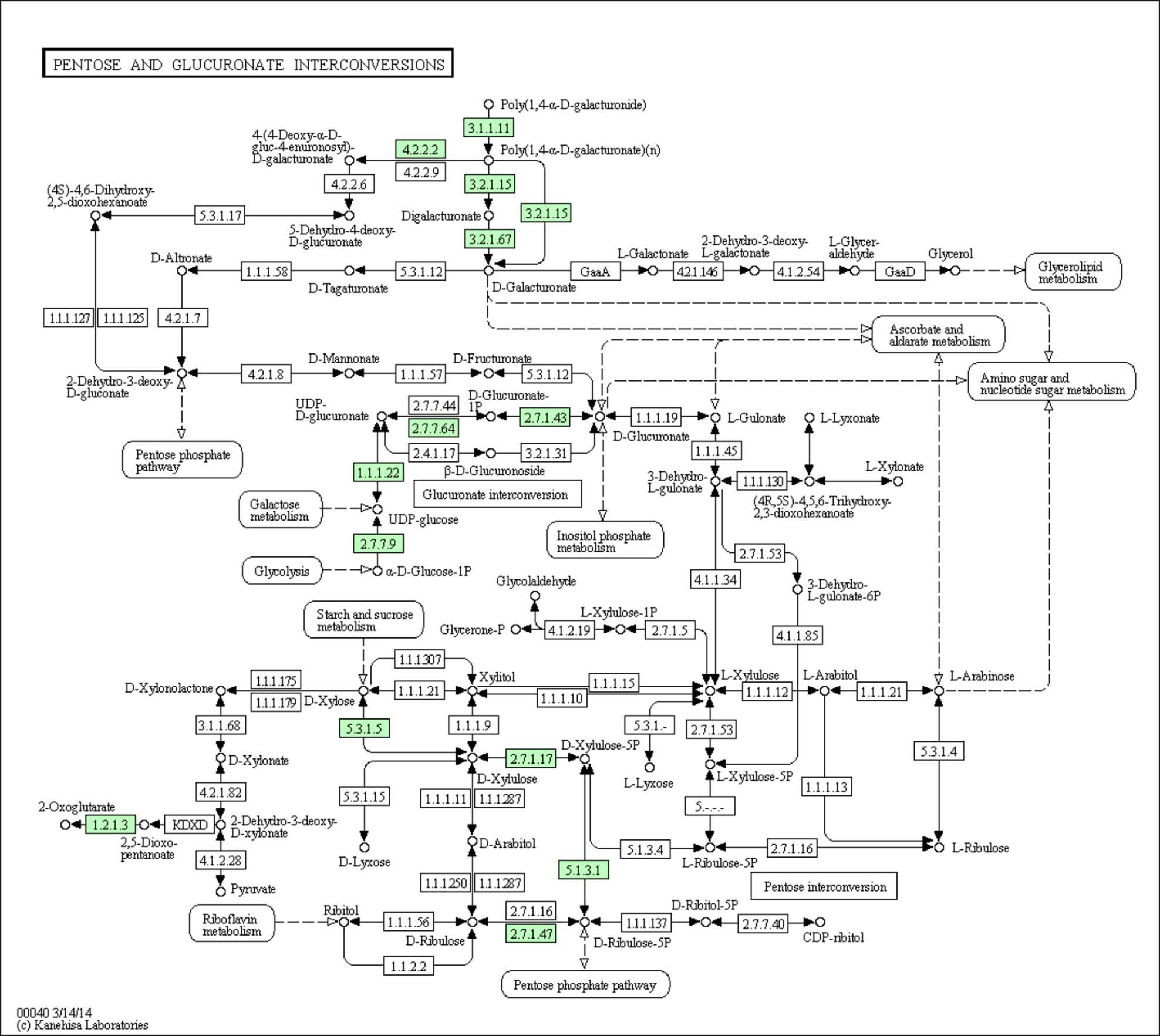
PENTOSE AND GLUCURONATE INTERCONVERSIONS
Poly(1,4-α-D-galacturonide)
3.1.1.11
Poly(1,4-α-D-galacturonate)(n)
4-(4-Deoxy-α-D-gluc-4-enuronosyl)-D-galacturonate
4.2.2.2
4.2.2.9
4.2.2.6
3.2.1.15
3.2.1.15
(4S)-4,6-Dihydroxy-2,5-dioxohexanoate
5.3.1.17
5-Dehydro-4-deoxy-D-glucuronate
Digalacturonate
3.2.1.67
D-Altronate
1.1.1.58
D-Tagaturonate
5.3.1.12
D-Galacturonate
GaaA
L-Galactonate
4.2.1.146
2-Dehydro-3-deoxy-L-galactonate
4.1.2.54
L-Glycer-aldehyde
GaaD
Glycerol
Glycerolipid metabolism
1.1.1.127
1.1.1.125
4.2.1.7
Ascorbate and aldarate metabolism
Amino sugar and nucleotide sugar metabolism
2-Dehydro-3-deoxy-D-gluconate
4.2.1.8
D-Mannonate
1.1.1.57
D-Fructuronate
5.3.1.12
UDP-D-glucuronate
2.7.7.44
2.7.7.64
D-Glucuronate-1P
2.7.1.43
1.1.1.19
L-Gulonate
L-Lyxonate
D-Glucuronate
Pentose phosphate pathway
2.4.1.17
3.2.1.31
β-D-Glucuronoside
1.1.1.45
L-Xylonate
1.1.1.22
Glucuronate interconversion
3-Dehydro-L-gulonate
1.1.1.130
(4R,5S)-4,5,6-Trihydroxy-2,3-dioxohexanoate
Galactose metabolism
UDP-glucose
Inositol phosphate metabolism
2.7.7.9
2.7.1.53
Glycolysis
α-D-Glucose-1P
4.1.1.34
3-Dehydro-L-gulonate-6P
Glycolaldehyde
L-Xylulose-1P
Starch and sucrose metabolism
Glycerone-P
4.1.2.19
2.7.1.5
4.1.1.85
1.1.1.307
Xylitol
L-Xylulose
L-Arabitol
L-Arabinose
1.1.1.15
D-Xylonolactone
1.1.1.175
1.1.1.179
D-Xylose
1.1.1.21
1.1.1.10
1.1.1.12
1.1.1.21
3.1.1.68
5.3.1.5
1.1.1.9
5.3.1.-
2.7.1.53
5.3.1.4
D-Xylonate
2.7.1.17
D-Xylulose-5P
D-Xylulose
L-Lyxose
L-Xylulose-5P
4.2.1.82
5.3.1.15
1.1.1.11
1.1.1.287
1.1.1.13
2-Oxoglutarate
1.2.1.3
KDXD
2-Dehydro-3-deoxy-D-xylonate
2,5-Dioxo-pentanoate
5.-.-.-
D-Lyxose
D-Arabitol
5.1.3.4
L-Ribulose-5P
2.7.1.16
L-Ribulose
4.1.2.28
5.1.3.1
Pyruvate
1.1.1.250
1.1.1.287
Pentose interconversion
Riboflavin metabolism
Ribitol
1.1.1.56
D-Ribulose
2.7.1.16
2.7.1.47
D-Ribulose-5P
1.1.1.137
D-Ribitol-5P
2.7.7.40
CDP-ribitol
1.1.2.2
Pentose phosphate pathway
00040 3/14/14
(c) Kanehisa Laboratories

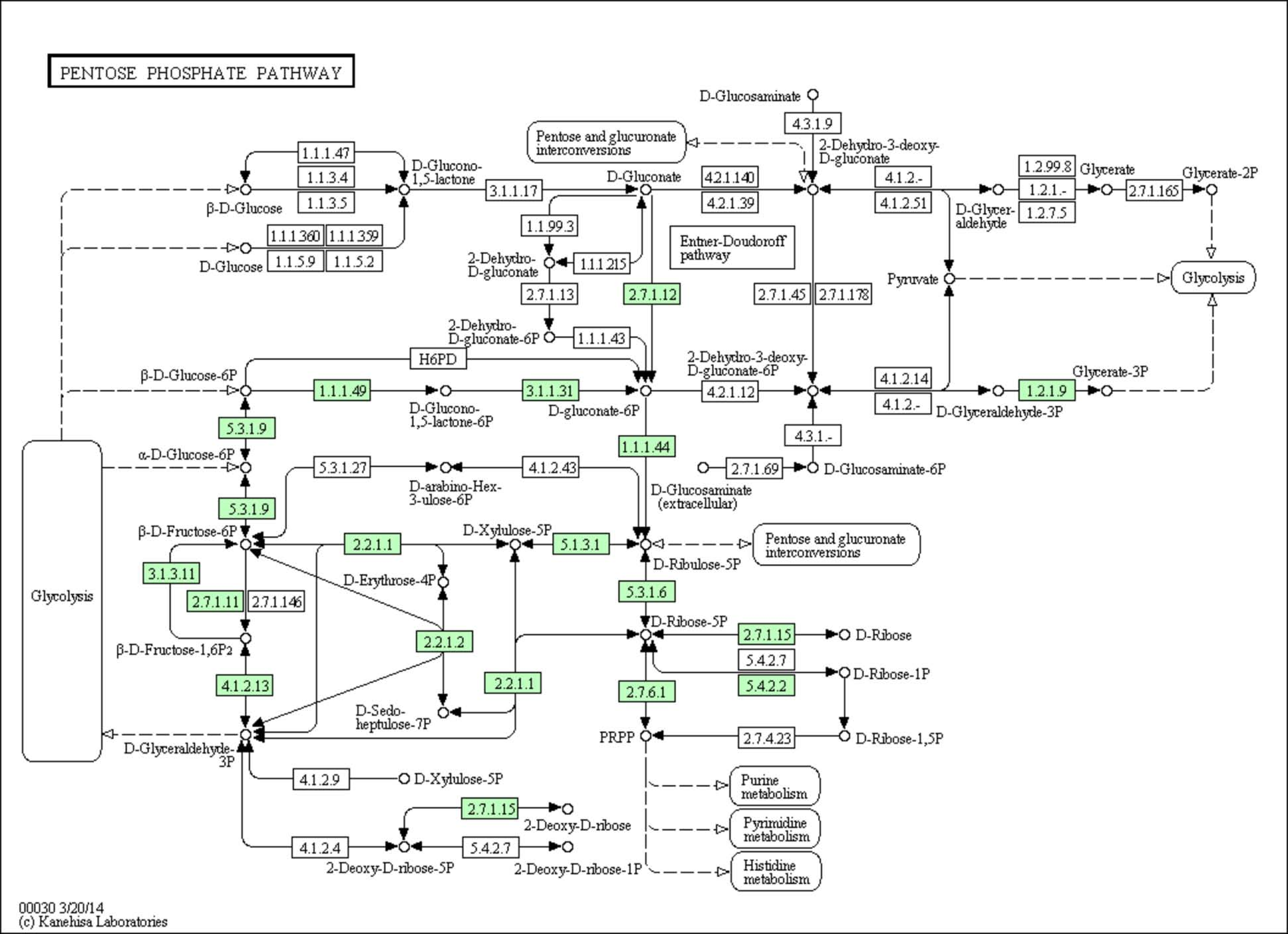
PENTOSE PHOSPHATE PATHWAY
D-Glucosaminate
4.3.1.9
Pentose and glucuronate interconversions
2-Dehydro-3-deoxy-D-gluconate
1.1.1.47
1.1.3.4
1.1.3.5
D-Glucono-1,5-lactone
3.1.1.17
D-Gluconate
4.2.1.140
4.2.1.39
4.1.2.-
4.1.2.51
1.2.99.8
Glycerate
1.2.1.-
1.2.7.5
2.7.1.165
Glycerate-2P
D-Glyceraldehyde
β-D-Glucose
1.1.1.360
1.1.1.359
1.1.5.9
1.1.5.2
D-Glucose
1.1.99.3
2-Dehydro-D-gluconate
1.1.1.215
Entner-Doudoroff pathway
2.7.1.12
2.7.1.13
2.7.1.45
2.7.1.178
Pyruvate
Glycolysis
2-Dehydro-D-gluconate-6P
1.1.1.43
H6PD
2-Dehydro-3-deoxy-D-gluconate-6P
β-D-Glucose-6P
1.1.1.49
3.1.1.31
4.2.1.12
4.1.2.14
4.1.2.-
1.2.1.9
Glycerate-3P
D-Glucono-1,5-lactone-6P
D-gluconate-6P
D-Glyceraldehyde-3P
5.3.1.9
1.1.1.44
4.3.1.-
α-D-Glucose-6P
5.3.1.27
4.1.2.43
2.7.1.69
D-Glucosaminate-6P
D-arabino-Hex-3-ulose-6P
D-Glucosaminate (extracellular)
5.3.1.9
β-D-Fructose-6P
2.2.1.1
D-Xylulose-5P
5.1.3.1
Pentose and glucuronate interconversions
D-Ribulose-5P
3.1.3.11
D-Erythrose-4P
Glycolysis
2.7.1.11
2.7.1.146
5.3.1.6
D-Ribose-5P
2.2.1.2
2.7.1.15
D-Ribose
β-D-Fructose-1,6P2
5.4.2.7
D-Ribose-1P
4.1.2.13
2.2.1.1
5.4.2.2
2.7.6.1
D-Sedo-heptulose-7P
PRPP
2.7.4.23
D-Ribose-1,5P
D-Glyceraldehyde-3P
4.1.2.9
D-Xylulose-5P
Purine metabolism
2.7.1.15
2-Deoxy-D-ribose
Pyrimidine metabolism
4.1.2.4
5.4.2.7
2-Deoxy-D-ribose-5P
2-Deoxy-D-ribose-1P
Histidine metabolism
00030 3/20/14
(c) Kanehisa Laboratories

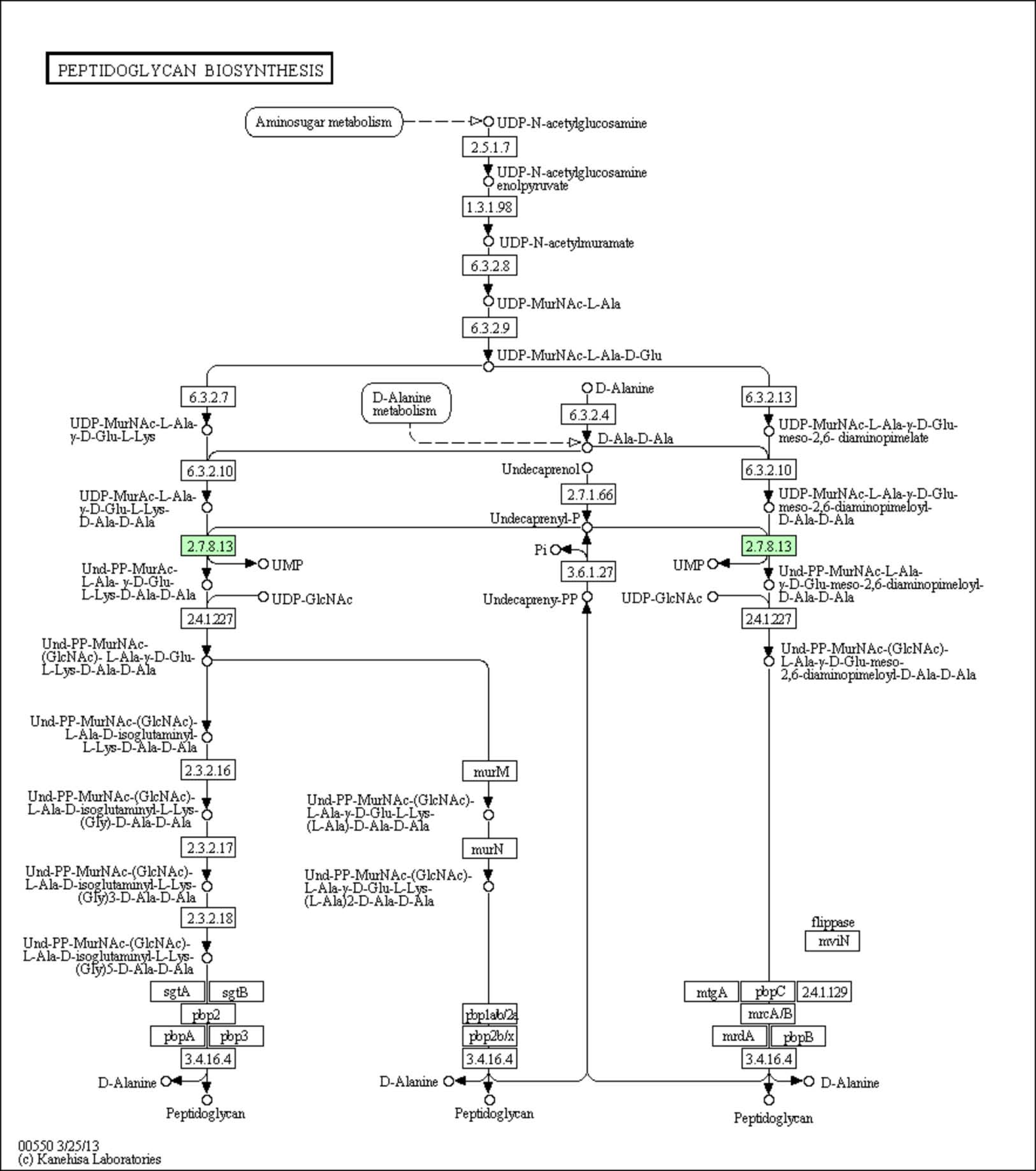
PEPTIDOGLYCAN BIOSYNTHESIS
Aminosugar metabolism
UDP-N-acetylglucosamine
2.5.1.7
UDP-N-acetylglucosamine enolpyruvate
1.3.1.98
UDP-N-acetylmuramate
6.3.2.8
UDP-MurNAc-L-Ala
6.3.2.9
UDP-MurNAc-L-Ala-D-Glu
6.3.2.7
D-Alanine metabolism
D-Alanine
6.3.2.4
6.3.2.13
UDP-MurNAc-L-Ala-γ-D-Glu-L-Lys
D-Ala-D-Ala
UDP-MurNAc-L-Ala-γ-D-Glu-meso-2,6- diaminopimelate
6.3.2.10
Undecaprenol
6.3.2.10
2.7.1.66
UDP-MurAc-L-Ala-γ-D-Glu-L-Lys-D-Ala-D-Ala
Undecaprenyl-P
UDP-MurNAc-L-Ala-γ-D-Glu-meso-2,6-diaminopimeloyl-D-Ala-D-Ala
2.7.8.13
Pi
2.7.8.13
UMP
UMP
3.6.1.27
Und-PP-MurAc-L-Ala- γ-D-Glu-L-Lys-D-Ala-D-Ala
Und-PP-MurNAc-L-Ala-γ-D-Glu-meso-2,6-diaminopimeloyl-D-Ala-D-Ala
UDP-GlcNAc
Undecapreny-PP
UDP-GlcNAc
2.4.1.227
2.4.1.227
Und-PP-MurNAc-(GlcNAc)- L-Ala-γ-D-Glu-L-Lys-D-Ala-D-Ala
Und-PP-MurNAc-(GlcNAc)-L-Ala-γ-D-Glu-meso-2,6-diaminopimeloyl-D-Ala-D-Ala
Und-PP-MurNAc-(GlcNAc)-L-Ala-D-isoglutaminyl-L-Lys-D-Ala-D-Ala
2.3.2.16
murM
Und-PP-MurNAc-(GlcNAc)-L-Ala-D-isoglutaminyl-L-Lys-(Gly)-D-Ala-D-Ala
Und-PP-MurNAc-(GlcNAc)-L-Ala-γ-D-Glu-L-Lys-(L-Ala)-D-Ala-D-Ala
2.3.2.17
murN
Und-PP-MurNAc-(GlcNAc)-L-Ala-D-isoglutaminyl-L-Lys-(Gly)3-D-Ala-D-Ala
Und-PP-MurNAc-(GlcNAc)-L-Ala-γ-D-Glu-L-Lys-(L-Ala)2-D-Ala-D-Ala
2.3.2.18
flippase
mviN
Und-PP-MurNAc-(GlcNAc)-L-Ala-D-isoglutaminyl-L-Lys-(Gly)5-D-Ala-D-Ala
sgtA
sgtB
mtgA
pbpC
2.4.1.129
pbp2
pbp1a/b/2a
mrcA/B
pbpA
pbp3
pbp2b/x
mrdA
pbpB
3.4.16.4
3.4.16.4
3.4.16.4
D-Alanine
D-Alanine
D-Alanine
Peptidoglycan
Peptidoglycan
Peptidoglycan
00550 3/25/13
(c) Kanehisa Laboratories

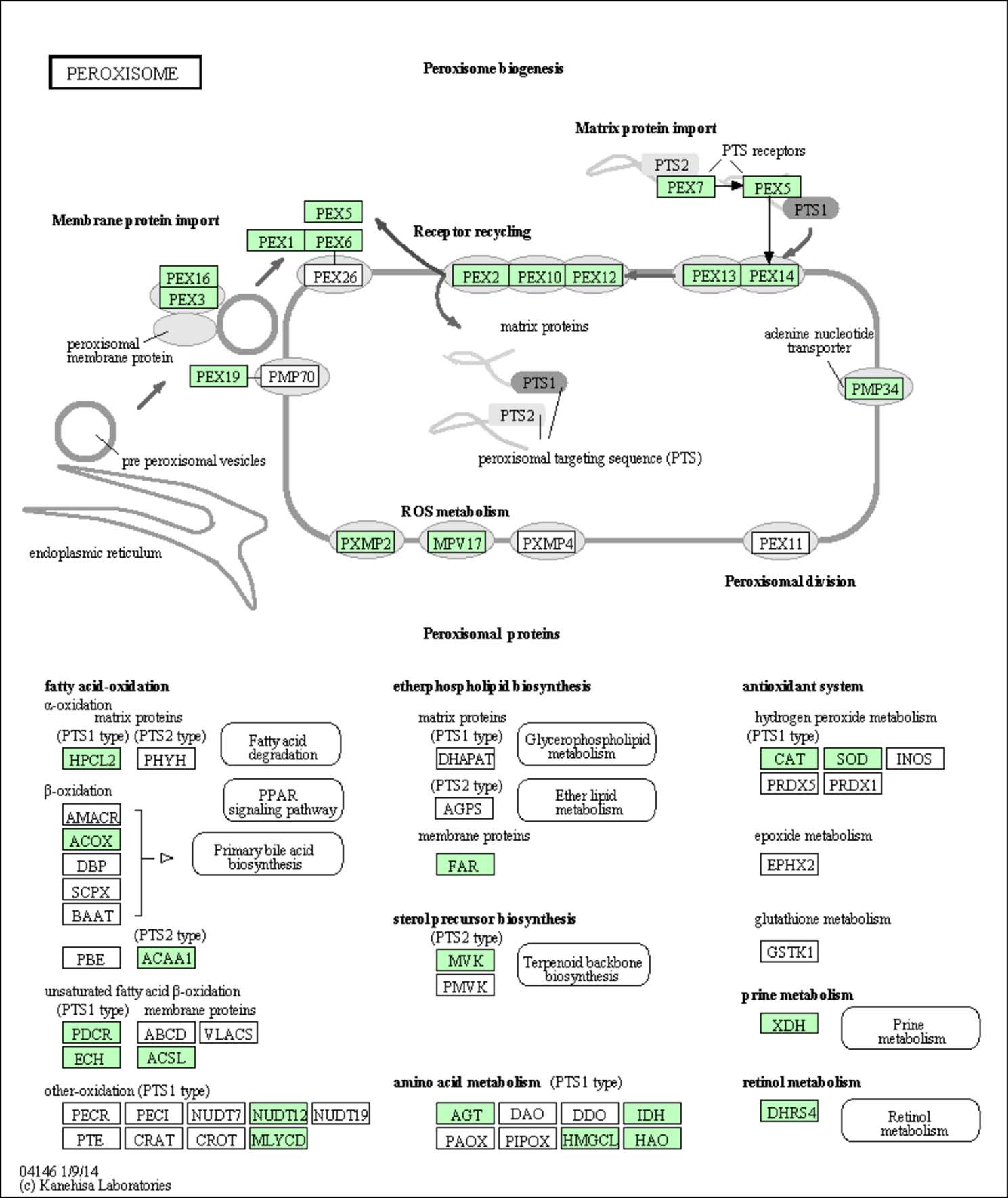
PEROXISOME
Peroxisome biogenesis
Matrix protein import
PTS2
PTS receptors
PEX7
PEX5
PTS1
Membrane protein import
PEX5
PEX1
PEX6
Receptor recycling
PEX16
PEX3
PEX26
PEX2
PEX10
PEX12
PEX13
PEX14
peroxisomal membrane protein
matrix proteins
adenine nucleotide transporter
PEX19
PMP70
PTS1
PMP34
PTS2
peroxisomal targeting sequence (PTS)
pre peroxisomal vesicles
endoplasmic reticulum
ROS metabolism
PXMP2
MPV17
PXMP4
PEX11
Peroxisomal division
Peroxisomal proteins
fatty acid-oxidation
α-oxidation
matrix proteins
(PTS1 type) (PTS2 type)
HPCL2
PHYH
Fatty acid degradation
β-oxidation
PPAR signaling pathway
AMACR
ACOX
DBP
SCPX
BAAT
Primary bile acid biosynthesis
(PTS2 type)
PBE
ACAA1
unsaturated fatty acid β-oxidation
(PTS1 type) membrane proteins
PDCR
ABCD
VLACS
ECH
ACSL
other-oxidation (PTS1 type)
PECR
PECI
NUDT7
NUDT12
NUDT19
PTE
CRAT
CROT
MLYCD
etherphospholipid biosynthesis
matrix proteins
(PTS1 type)
DHAPAT
Glycerophospholipid metabolism
(PTS2 type)
AGPS
Ether lipid metabolism
membrane proteins
FAR
sterol precursor biosynthesis
(PTS2 type)
MVK
PMVK
Terpenoid backbone biosynthesis
amino acid metabolism (PTS1 type)
AGT
DAO
DDO
IDH
PAOX
PIPOX
HMGCL
HAO
antioxidant system
hydrogen peroxide metabolism
(PTS1 type)
CAT
SOD
INOS
PRDX5
PRDX1
epoxide metabolism
EPHX2
glutathione metabolism
GSTK1
prine metabolism
XDH
Prine metabolism
retinol metabolism
DHRS4
Retinol metabolism
04146 1/9/14
(c) Kanehisa Laboratories

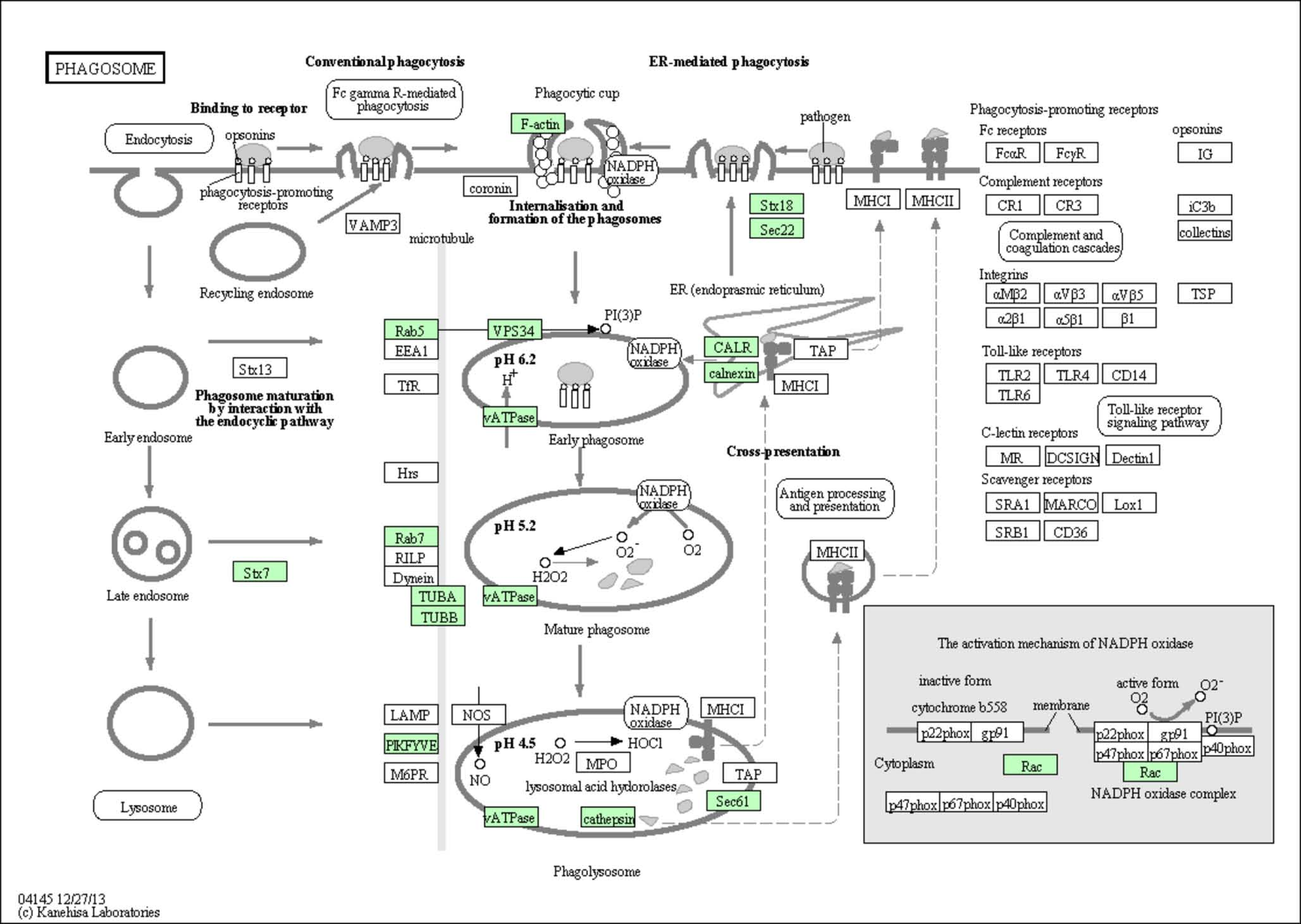
PHAGOSOME
Conventional phagocytosis
ER-mediated phagocytosis
Binding to receptor
Fc gamma R-mediated phagocytosis
Phagocytic cup
F-actin
Endocytosis
opsonins
phagocytosis-promoting receptors
NADPH oxidase
coronin
pathogen
Phagocytosis-promoting receptors
Fc receptors
FcαR
FcγR
opsonins
IG
Complement receptors
CR1
CR3
iC3b
collectins
Complement and coagulation cascades
Integrins
αMβ2
αVβ3
αVβ5
TSP
α2β1
α5β1
β1
Toll-like receptors
TLR2
TLR4
CD14
TLR6
Toll-like receptor signaling pathway
C-lectin receptors
MR
DCSIGN
Dectin1
Scavenger receptors
SRA1
MARCO
Lox1
SRB1
CD36
VAMP3
microtubule
Internalisation and formation of the phagosomes
Stx18
Sec22
MHCI
MHCII
Recycling endosome
ER (endoprasmic reticulum)
Rab5
VPS34
PI(3)P
EEA1
Stx13
pH 6.2
H+
CALR
TAP
calnexin
MHCI
TfR
Phagosome maturation by interaction with the endocyclic pathway
vATPase
Early endosome
Early phagosome
Cross-presentation
Hrs
Antigen processing and presentation
pH 5.2
O2-
O2
H2O2
Rab7
RILP
Dynein
Stx7
TUBA
TUBB
Late endosome
Mature phagosome
The activation mechanism of NADPH oxidase
inactive form
active form
cytochrome b558
membrane
p22phox
gp91
p47phox
p67phox
p40phox
Cytoplasm
Rac
NADPH oxidase complex
LAMP
NOS
PIKFYVE
M6PR
NO
pH 4.5
H2O2
HOCl
MPO
lysosomal acid hydrolases
Sec61
cathepsin
Lysosome
Phagolysosome
04145 12/27/13
(c) Kanehisa Laboratories

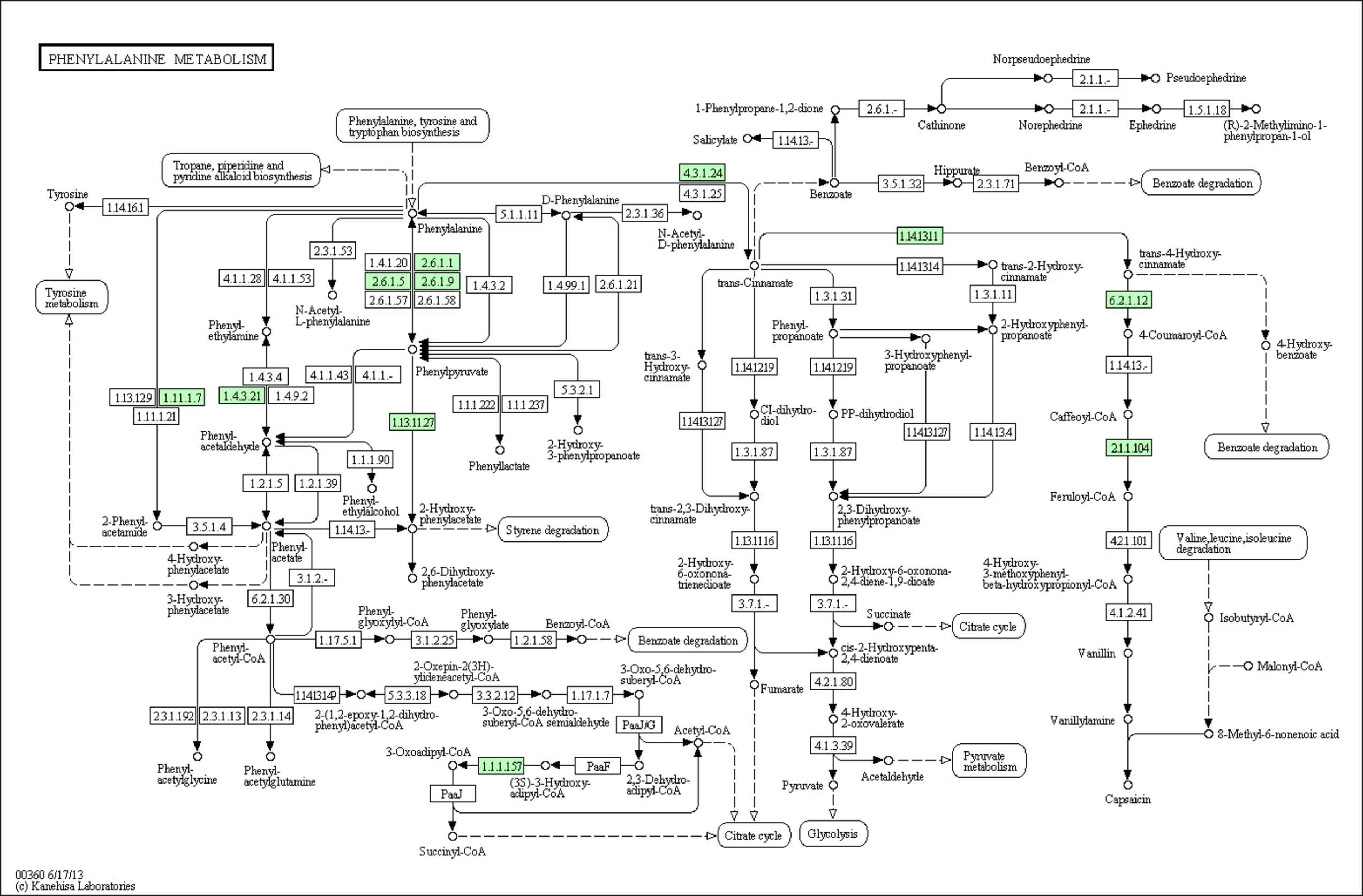
PHENYLALANINE METABOLISM
Phenylalanine, tyrosine and tryptophan biosynthesis
Tropane, piperidine and pyridine alkaloid biosynthesis
Tyrosine
1.14.16.1
Tyrosine metabolism
Phenylalanine
5.1.1.11
D-Phenylalanine
2.3.1.36
N-Acetyl-D-phenylalanine
4.3.1.24
4.3.1.25
2.3.1.53
N-Acetyl-L-phenylalanine
1.4.1.20
2.6.1.1
2.6.1.5
2.6.1.9
2.6.1.57
2.6.1.58
1.4.3.2
1.4.99.1
2.6.1.21
4.1.1.28
4.1.1.53
Phenyl-ethylamine
1.4.3.4
1.4.3.21
1.4.9.2
4.1.1.43
4.1.1.-
Phenylpyruvate
5.3.2.1
1.13.12.9
1.11.1.7
1.11.1.21
1.13.11.27
1.1.1.222
1.1.1.237
Phenyllactate
2-Hydroxy-3-phenylpropanoate
Phenyl-acetaldehyde
1.1.1.90
Phenyl-ethylalcohol
1.2.1.5
1.2.1.39
2-Phenyl-acetamide
3.5.1.4
1.14.13.-
2-Hydroxy-phenylacetate
Styrene degradation
2,6-Dihydroxy-phenylacetate
4-Hydroxy-phenylacetate
3-Hydroxy-phenylacetate
Phenyl-acetate
3.1.2.-
6.2.1.30
Phenyl-acetyl-CoA
1.17.5.1
Phenyl-glyoxylyl-CoA
3.1.2.25
Phenyl-glyoxylate
1.2.1.58
Benzoyl-CoA
Benzoate degradation
2.3.1.192
2.3.1.13
2.3.1.14
Phenyl-acetylglycine
Phenyl-acetylglutamine
1.14.13.149
2-(1,2-epoxy-1,2-dihydro-phenyl)acetyl-CoA
5.3.3.18
2-Oxepin-2(3H)-ylideneacetyl-CoA
3.3.2.12
3-Oxo-5,6-dehydro-suberyl-CoA semialdehyde
1.17.1.7
3-Oxo-5,6-dehydro-suberyl-CoA
PaaJ/G
Acetyl-CoA
PaaF
2,3-Dehydro-adipyl-CoA
1.1.1.157
(3S)-3-Hydroxy-adipyl-CoA
3-Oxoadipyl-CoA
PaaJ
Succinyl-CoA
Citrate cycle
1-Phenylpropane-1,2-dione
2.6.1.-
Cathinone
Norpseudoephedrine
2.1.1.-
Pseudoephedrine
Norephedrine
Ephedrine
1.5.1.18
(R)-2-Methylimino-1-phenylpropan-1-ol
Salicylate
1.14.13.-
Benzoate
3.5.1.32
Hippurate
2.3.1.71
Benzoyl-CoA
Benzoate degradation
1.14.13.11
trans-Cinnamate
1.14.13.14
trans-2-Hydroxy-cinnamate
trans-4-Hydroxy-cinnamate
1.3.1.11
1.3.1.31
Phenyl-propanoate
2-Hydroxyphenyl-propanoate
3-Hydroxyphenyl-propanoate
6.2.1.12
4-Coumaroyl-CoA
4-Hydroxy-benzoate
trans-3-Hydroxy-cinnamate
1.14.12.19
1.14.12.19
1.14.13.127
CI-dihydro-diol
PP-dihydrodiol
1.14.13.127
1.14.13.4
1.14.13.-
Caffeoyl-CoA
2.1.1.104
Benzoate degradation
1.3.1.87
1.3.1.87
trans-2,3-Dihydroxy-cinnamate
2,3-Dihydroxy-phenylpropanoate
Feruloyl-CoA
1.13.11.16
1.13.11.16
4.2.1.101
Valine, leucine, isoleucine degradation
2-Hydroxy-6-oxonona-trienedioate
2-Hydroxy-6-oxonona-2,4-diene-1,9-dioate
4-Hydroxy-3-methoxyphenyl-beta-hydroxypropionyl-CoA
3.7.1.-
3.7.1.-
Succinate
Citrate cycle
4.1.2.41
Isobutyryl-CoA
cis-2-Hydroxypenta-2,4-dienoate
Vanillin
Malonyl-CoA
Fumarate
4.2.1.80
4-Hydroxy-2-oxovalerate
Vanillylamine
8-Methyl-6-nonenoic acid
4.1.3.39
Acetaldehyde
Pyruvate metabolism
Pyruvate
Capsaicin
Citrate cycle
Glycolysis
00360 6/17/13
(c) Kanehisa Laboratories

PHENYLALANINE, TYROSINE AND TRYPTOPHAN BIOSYNTHESIS

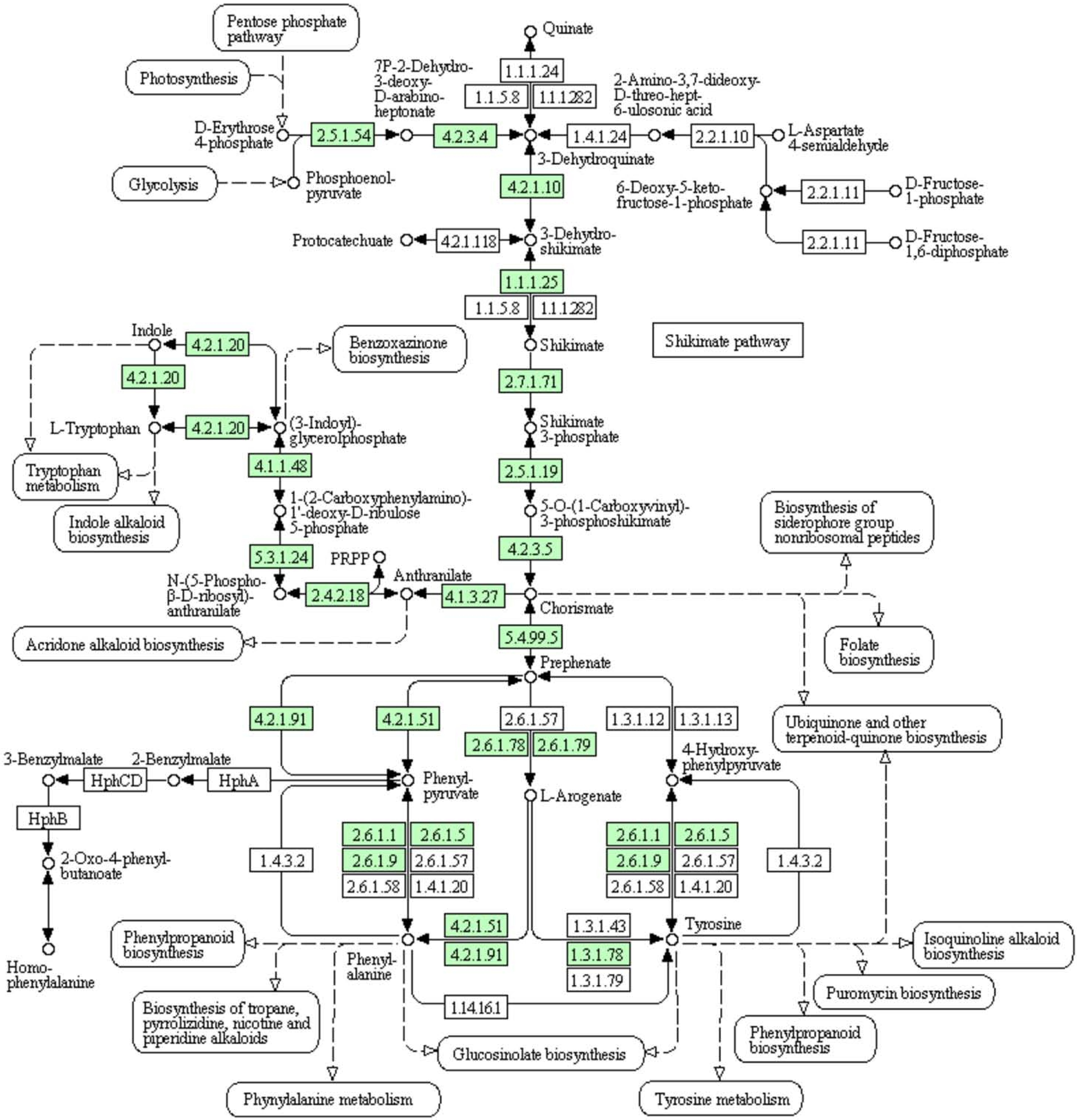

00400 11/18/13
(c) Kanehisa Laboratories

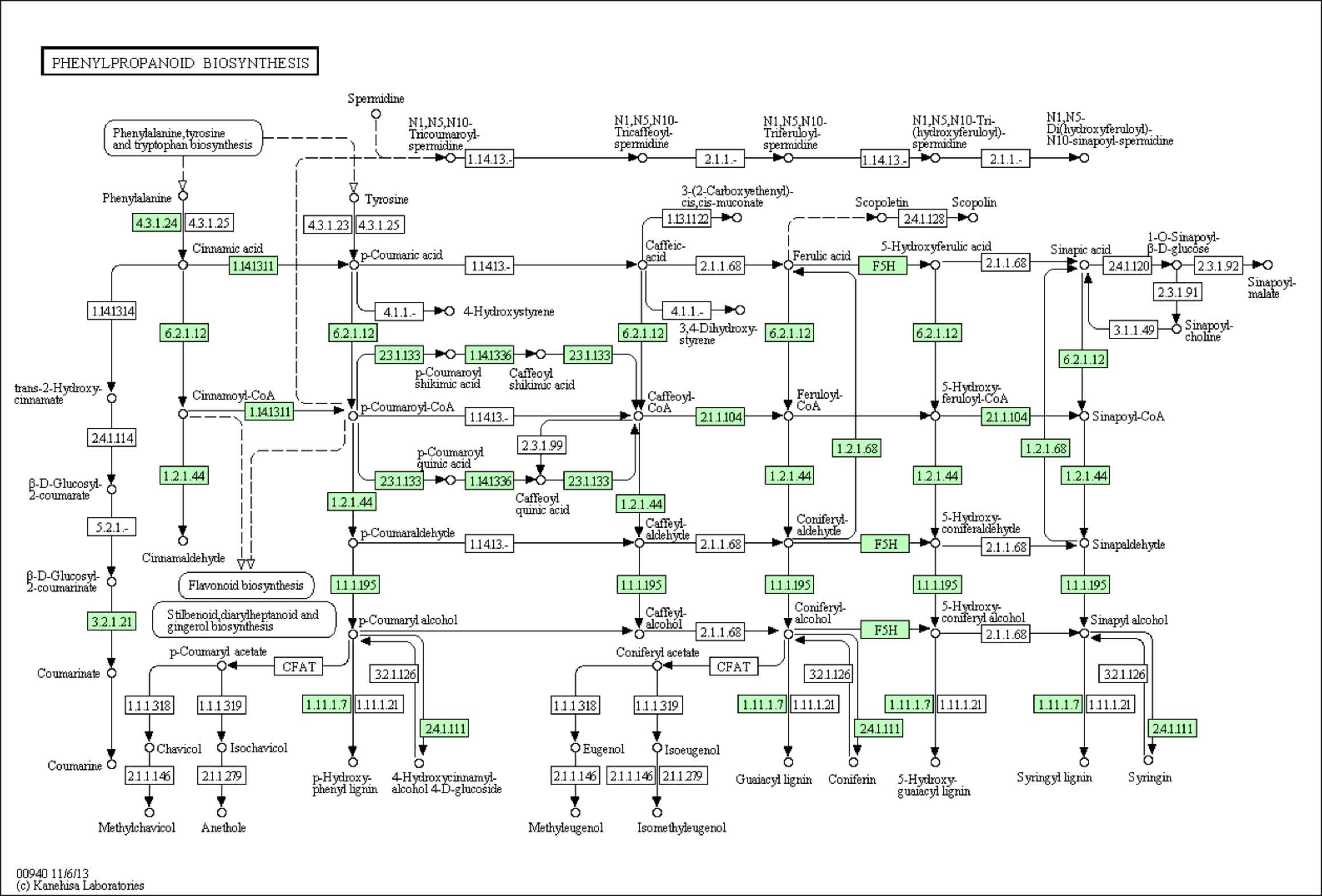
PHENYLPROPANOID BIOSYNTHESIS
Spermidine
Phenylalanine, tyrosine and tryptophan biosynthesis
N1,N5,N10-Tricoumaroyl-spermidine
1.14.13.-
N1,N5,N10-Tricaffeoyl-spermidine
2.1.1.-
N1,N5,N10-Triferuloyl-spermidine
1.14.13.-
N1,N5,N10-Tri-(hydroxyferuloyl)-spermidine
2.1.1.-
N1,N5-Di(hydroxyferuloyl)-N10-sinapoyl-spermidine
Phenylalanine
4.3.1.24
4.3.1.25
Tyrosine
4.3.1.23
4.3.1.25
3-(2-Carboxyethenyl)-cis,cis-muconate
1.13.11.22
Scopoletin
2.4.1.128
Scopolin
Cinnamic acid
1.14.13.11
p-Coumaric acid
1.14.13.-
Caffeic-acid
2.1.1.68
Ferulic acid
F5H
5-Hydroxyferulic acid
2.1.1.68
Sinapic acid
2.4.1.120
1-O-Sinapoyl-β-D-glucose
2.3.1.92
Sinapoyl-malate
2.3.1.91
3.1.1.49
Sinapoyl-choline
1.14.13.14
4.1.1.-
4-Hydroxystyrene
4.1.1.-
3,4-Dihydroxy-styrene
6.2.1.12
6.2.1.12
6.2.1.12
6.2.1.12
6.2.1.12
6.2.1.12
2.3.1.133
1.14.13.36
2.3.1.133
p-Coumaroyl shikimic acid
Caffeoyl shikimic acid
trans-2-Hydroxy-cinnamate
Cinnamoyl-CoA
1.14.13.11
p-Coumaroyl-CoA
1.14.13.-
Caffeoyl-CoA
2.1.1.104
Feruloyl-CoA
5-Hydroxy-feruloyl-CoA
2.1.1.104
Sinapoyl-CoA
2.4.1.114
2.3.1.99
p-Coumaroyl quinic acid
1.2.1.68
1.2.1.68
β-D-Glucosyl-2-coumarate
1.2.1.44
2.3.1.133
1.14.13.36
2.3.1.133
1.2.1.44
1.2.1.44
1.2.1.44
Caffeoyl quinic acid
1.2.1.44
1.2.1.44
5.2.1.-
Cinnamaldehyde
p-Coumaraldehyde
1.14.13.-
Caffeyl-aldehyde
2.1.1.68
Coniferyl-aldehyde
F5H
5-Hydroxy-coniferaldehyde
2.1.1.68
Sinapaldehyde
β-D-Glucosyl-2-coumarinate
Flavonoid biosynthesis
1.1.1.195
1.1.1.195
1.1.1.195
1.1.1.195
1.1.1.195
3.2.1.21
Stilbenoid, diarylheptanoid and gingerol biosynthesis
p-Coumaryl alcohol
Caffeyl-alcohol
2.1.1.68
Coniferyl-alcohol
F5H
5-Hydroxy-coniferyl alcohol
2.1.1.68
Sinapyl alcohol
p-Coumaryl acetate
CFAT
Coniferyl acetate
CFAT
Coumarinate
3.2.1.126
3.2.1.126
3.2.1.126
1.1.1.318
1.1.1.319
1.11.1.7
1.11.1.21
1.1.1.318
1.1.1.319
1.11.1.7
1.11.1.21
1.11.1.7
1.11.1.21
1.11.1.7
1.11.1.21
2.4.1.111
2.4.1.111
2.4.1.111
Chavicol
Isochavicol
Eugenol
Isoeugenol
Coumarine
2.1.1.146
2.1.1.279
2.1.1.146
2.1.1.146
2.1.1.279
p-Hydroxy-phenyl lignin
4-Hydroxycinnamyl-alcohol 4-D-glucoside
Guaiacyl lignin
Coniferin
5-Hydroxy-guaiacyl lignin
Syringyl lignin
Syringin
Methylchavicol
Anethole
Methyleugenol
Isomethyleugenol
00940 11/6/13
(c) Kanehisa Laboratories

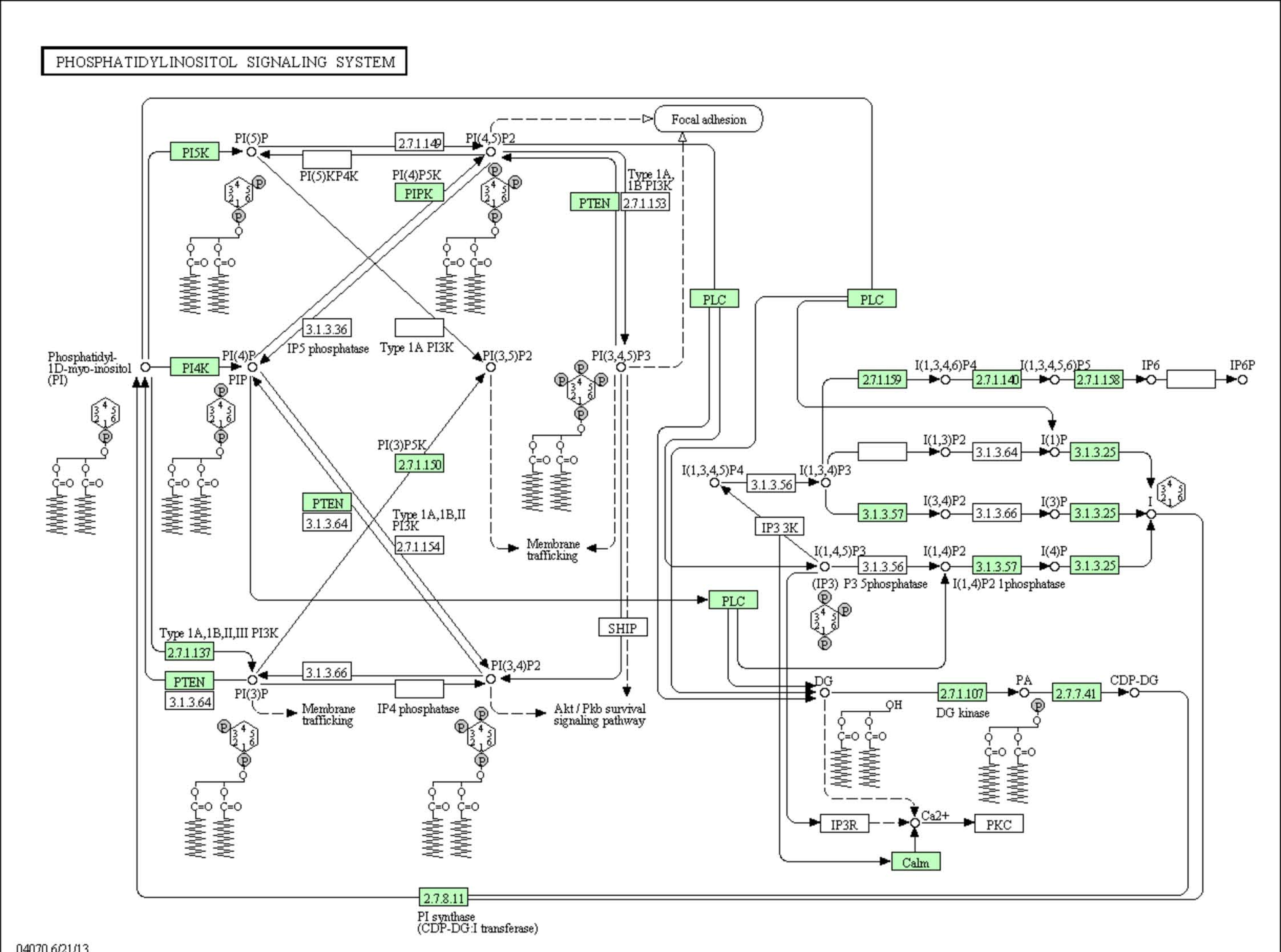

04070 6/21/13
(c) Kanehisa Laboratories

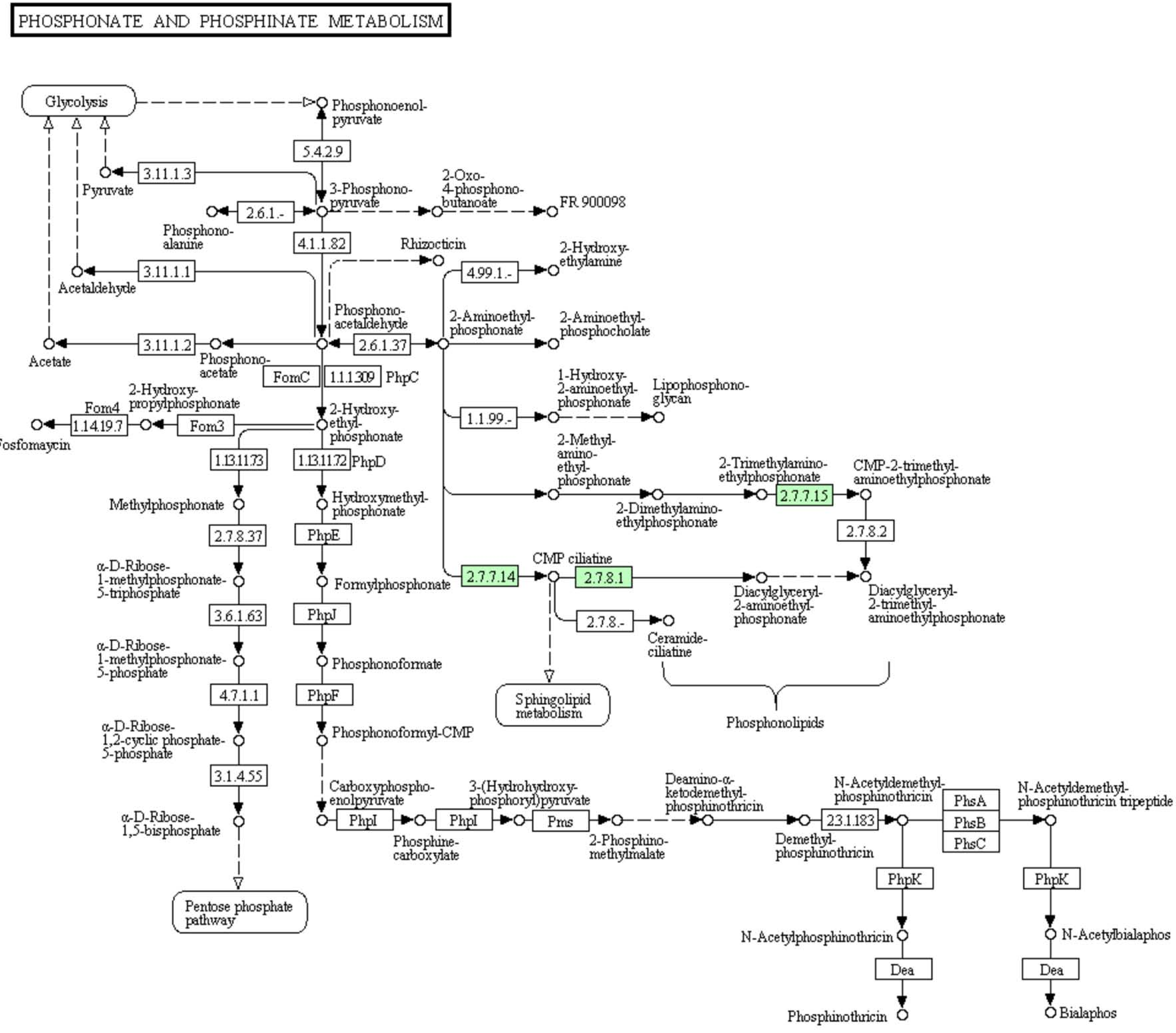

00440 2/21/14
(c) Kanehisa Laboratories

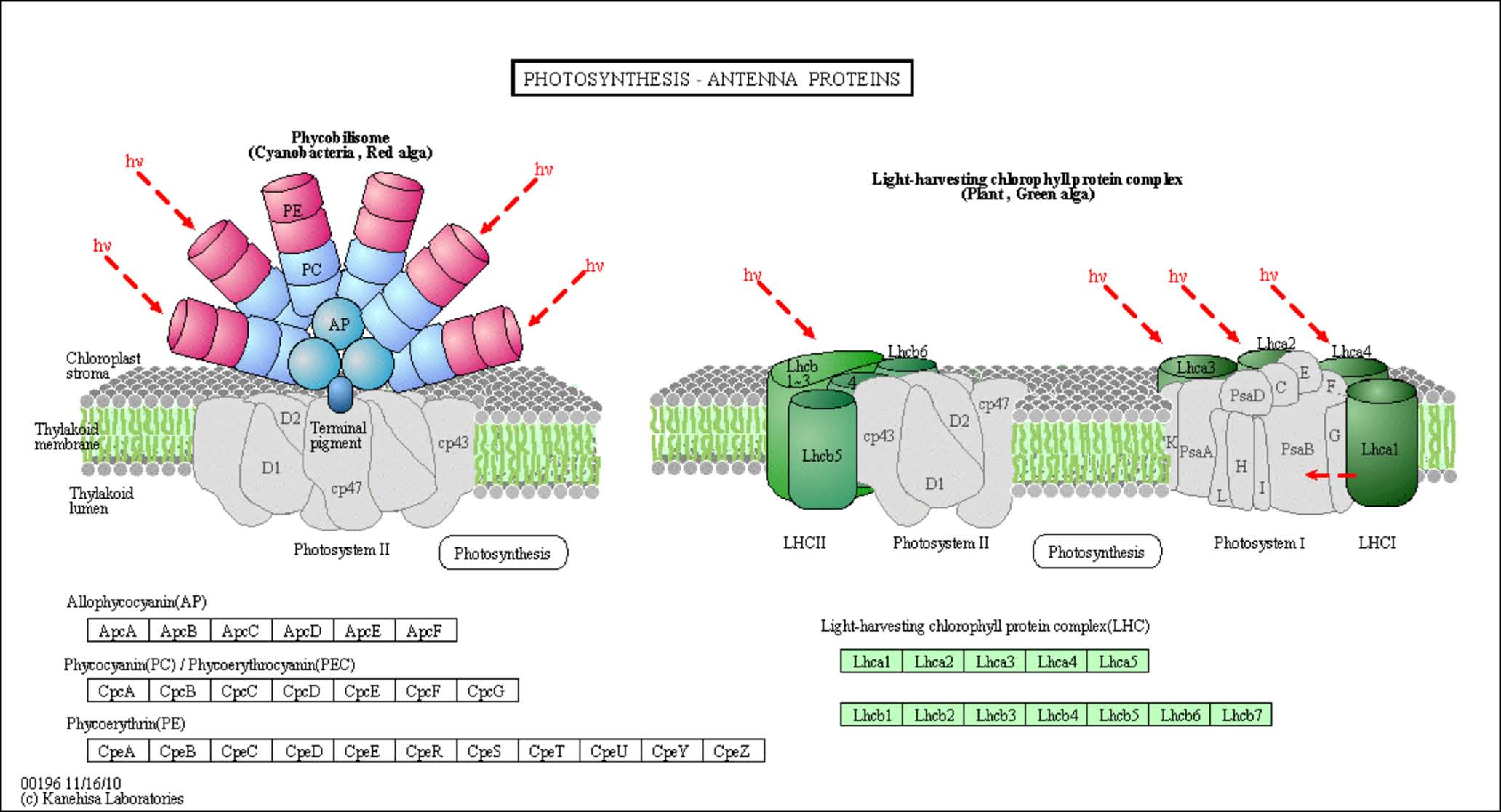

PHOTOSYNTHESIS - ANTENNA PROTEINS
Phycobilisome
(Cyanobacteria , Red alga)
hν
PE
PC
AP
Chloroplast stroma
Thylakoid membrane
Thylakoid lumen
D2
D1
Terminal pigment
cp43
cp47
Photosystem II
Photosynthesis
Light-harvesting chlorophyll protein complex
(Plant , Green alga)
Lhcb 1~3
4
Lhcb6
Lhcb5
cp43
cp47
D2
D1
LHCII
Photosystem II
Photosynthesis
Lhca3
Lhca2
Lhca4
PsaD
C
E
F
K
PsaA
H
L
I
PsaB
G
Lhca1
Photosystem I
LHCI
Allophycocyanin(AP)
ApcA
ApcB
ApcC
ApcD
ApcE
ApcF
Phycocyanin(PC) / Phycoerythrocyanin(PEC)
CpcA
CpcB
CpcC
CpcD
CpcE
CpcF
CpcG
Phycoerythrin(PE)
CpeA
CpeB
CpeC
CpeD
CpeE
CpeR
CpeS
CpeT
CpeU
CpeY
CpeZ
Light-harvesting chlorophyll protein complex(LHC)
Lhca1
Lhca2
Lhca3
Lhca4
Lhca5
Lhcb1
Lhcb2
Lhcb3
Lhcb4
Lhcb5
Lhcb6
Lhcb7
00196 11/16/10
(c) Kanehisa Laboratories

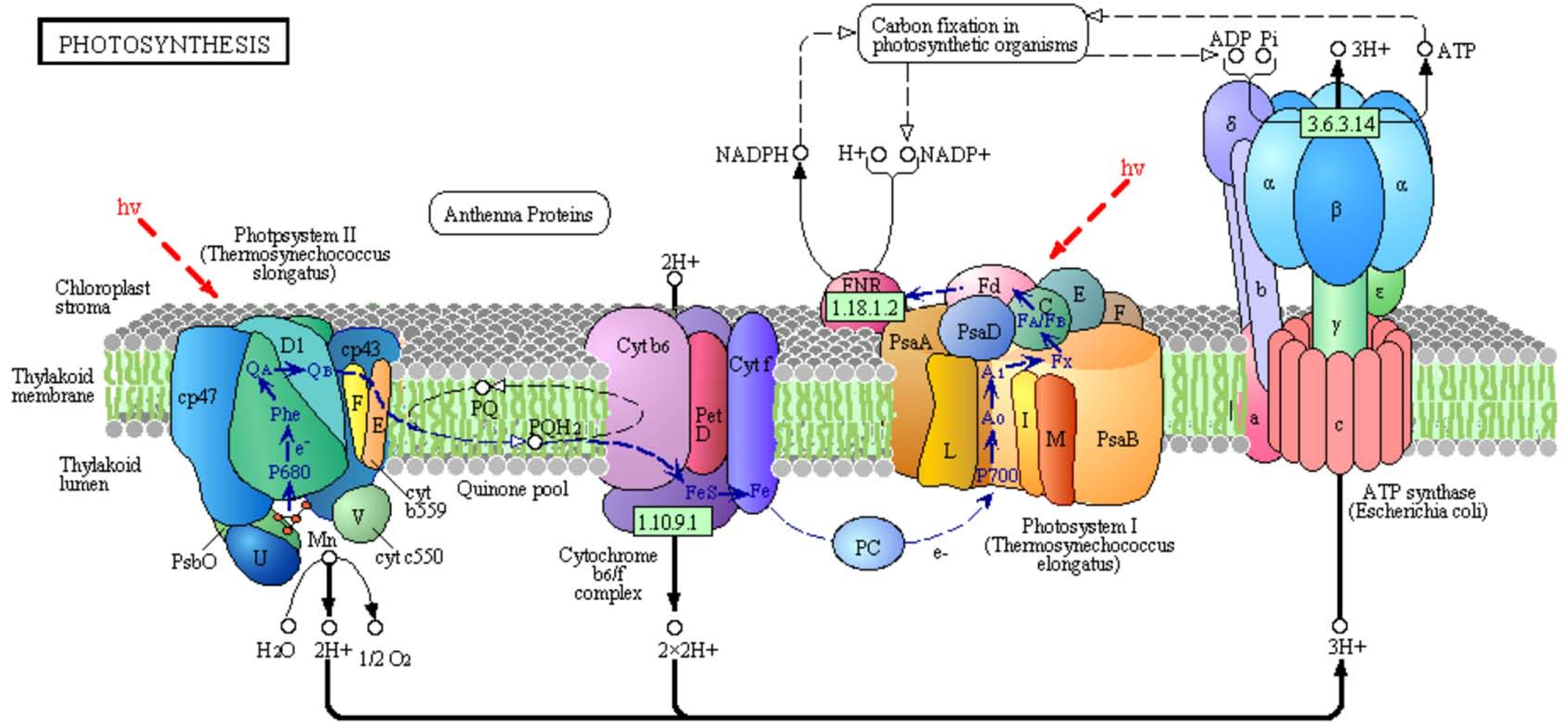

Photosystem II

D1	D2	cp43	cp47	cyt b559	
PsbA	PsbD	PsbC	PsbB	PsbE	PsbF

						MSP	OEC
Psb L	Psb J	Psb K	Psb M	Psb H	Psb I	Psb O	Psb P
Psb Q	Psb R	Psb S	Psb T	Psb U	Psb V	Psb W	Psb X
Psb Y	Psb Z	Psb 27	Psb 28	Psb28-2			

Photosystem I

PsaA	PsaB	PsaC	PsaD	PsaE	PsaF	PsaG	PsaH
PsaI	PsaJ	PsaK	PsaL	PsaM	PsaN	PsaO	PsaX

Cytochrome b6/f complex

PetB	PetD	PetA	PetC	PetL	PetM	PetN	PetG

Photosynthethic electron transport

PC	Fd	FNR	cyt c6
PetE	PetF	PetH	PetJ

F-type ATPase

beta	alpha	gamma	delta	epsilon	c	a	b

00195 1/15/14

(c) Kanehisa Laboratories

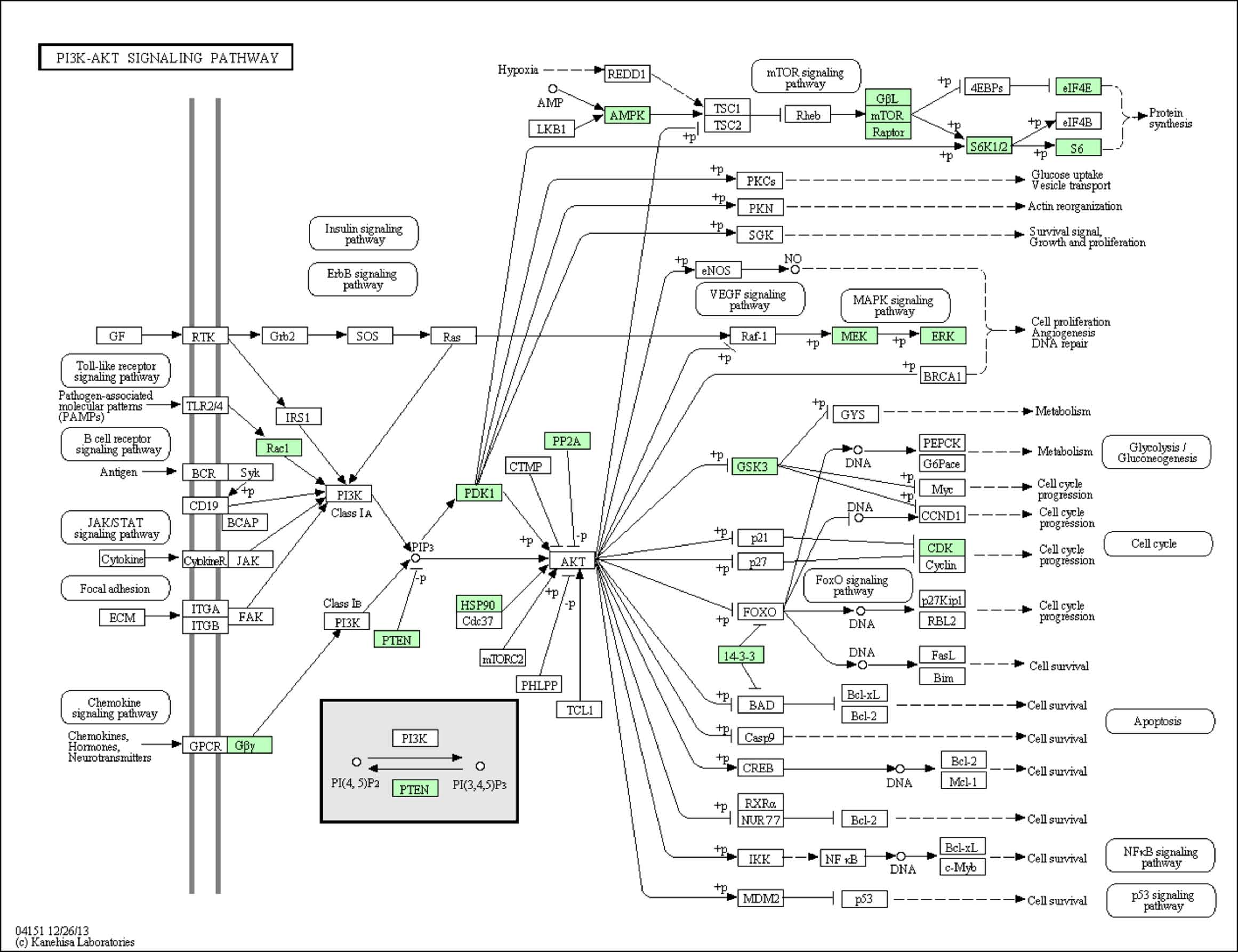
PI3K-AKT SIGNALING PATHWAY
Hypoxia
REDD1
AMP
AMPK
LKB1
TSC1
TSC2
Rheb
mTOR signaling pathway
GβL
mTOR
Raptor
4EBPs
eIF4E
eIF4B
S6K1/2
S6
Protein synthesis
+p
PKCs
Glucose uptake
Vesicle transport
PKN
Actin reorganization
SGK
Survival signal,
Growth and proliferation
Insulin signaling pathway
ErbB signaling pathway
eNOS
NO
VEGF signaling pathway
MAPK signaling pathway
Cell proliferation
Angiogenesis
DNA repair
GF
RTK
Grb2
SOS
Ras
Raf-1
MEK
ERK
BRCA1
Toll-like receptor signaling pathway
Pathogen-associated molecular patterns (PAMPs)
TLR2/4
IRS1
Rac1
B cell receptor signaling pathway
Antigen
BCR
Syk
CD19
BCAP
PI3K
Class IA
JAK/STAT signaling pathway
Cytokine
CytokineR
JAK
Focal adhesion
ECM
ITGA
ITGB
FAK
Class IB
PI3K
PIP3
-p
PTEN
PDK1
PP2A
CTMP
AKT
HSP90
Cdc37
mTORC2
PHLPP
TCL1
GYS
Metabolism
GSK3
DNA
PEPCK
G6Pace
Metabolism
Glycolysis / Gluconeogenesis
Myc
Cell cycle progression
CCND1
Cell cycle progression
p21
p27
CDK
Cyclin
Cell cycle progression
Cell cycle
FoxO signaling pathway
FOXO
p27Kip1
RBL2
Cell cycle progression
14-3-3
FasL
Bim
Cell survival
BAD
Bcl-xL
Bcl-2
Cell survival
Apoptosis
Casp9
Cell survival
CREB
Bcl-2
Mcl-1
Cell survival
RXRα
NUR77
Bcl-2
Cell survival
IKK
NF κB
Bcl-xL
c-Myb
Cell survival
NFκB signaling pathway
MDM2
p53
Cell survival
p53 signaling pathway
Chemokine signaling pathway
Chemokines,
Hormones,
Neurotransmitters
GPCR
Gβγ
PI3K
PI(4, 5)P2
PTEN
PI(3,4,5)P3
04151 12/26/13
(c) Kanehisa Laboratories

PLANT HORMONE SIGNAL TRANSDUCTION

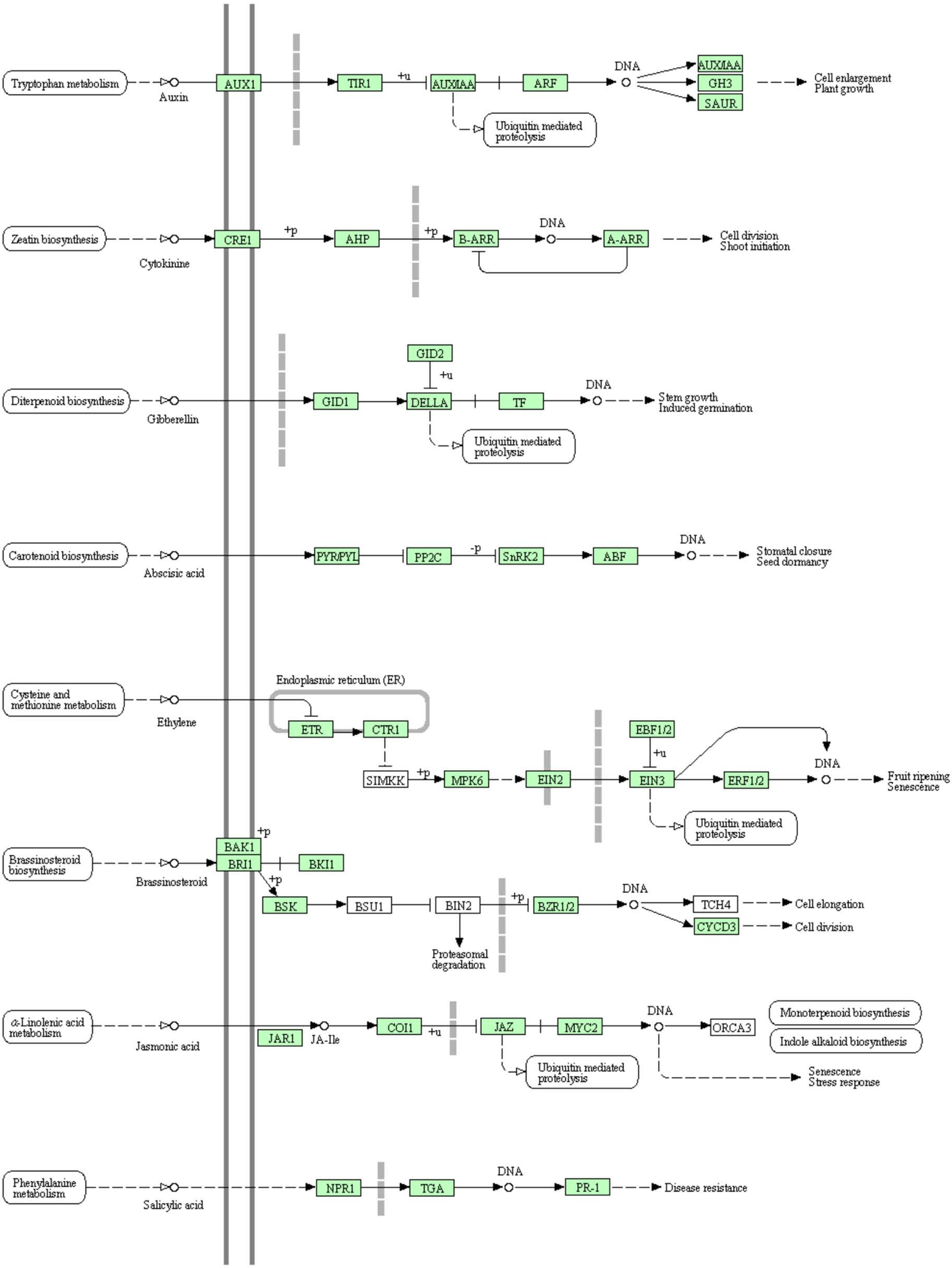

04075 10/30/12
(c) Kanehisa Laboratories

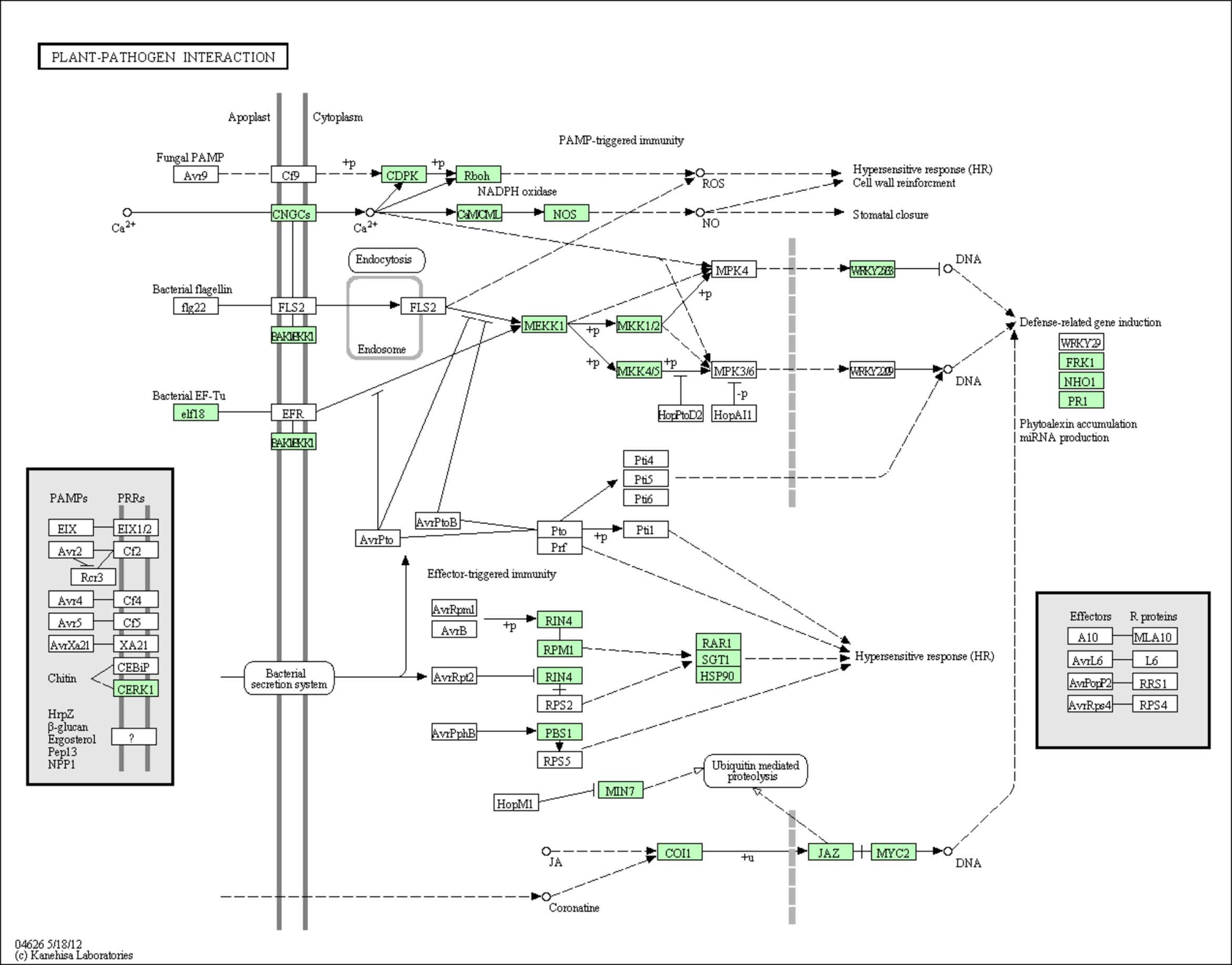
PLANT-PATHOGEN INTERACTION
Apoplast
Cytoplasm
PAMP-triggered immunity
Fungal PAMP
Avr9
Cf9
+p
CDPK
+p
Rboh
NADPH oxidase
ROS
Hypersensitive response (HR)
Cell wall reinforcement
Ca2+
CNGCs
Ca2+
CaM/CML
NOS
NO
Stomatal closure
Endocytosis
Bacterial flagellin
flg22
FLS2
FLS2
BAK1/BKK1
Endosome
MEKK1
+p
MKK1/2
+p
MPK4
WRKY22/33
DNA
Defense-related gene induction
WRKY29
FRK1
NHO1
PR1
+p
MKK4/5
+p
MPK3/6
WRKY22/29
DNA
-p
HopPtoD2
HopAI1
Phytoalexin accumulation
miRNA production
Bacterial EF-Tu
elf18
EFR
BAK1/BKK1
Pti4
Pti5
Pti6
PAMPs
PRRs
EIX
EIX1/2
Avr2
Cf2
Rcr3
Avr4
Cf4
Avr5
Cf5
AvrXa21
XA21
CEBiP
Chitin
CERK1
HrpZ
β-glucan
Ergosterol
Pep13
NPP1
?
AvrPtoB
Pto
AvrPto
Prf
+p
Pti1
Effector-triggered immunity
AvrRpm1
AvrB
+p
RIN4
RPM1
RAR1
SGT1
HSP90
Hypersensitive response (HR)
Bacterial secretion system
AvrRpt2
RIN4
RPS2
AvrPphB
PBS1
RPS5
Effectors
R proteins
A10
MLA10
AvrL6
L6
AvrPopP2
RRS1
AvrRps4
RPS4
Ubiquitin mediated proteolysis
HopM1
MIN7
JA
COI1
+u
JAZ
MYC2
DNA
Coronatine
04626 5/18/12
(c) Kanehisa Laboratories

POLYCYCLIC AROMATIC HYDROCARBON DEGRADATION

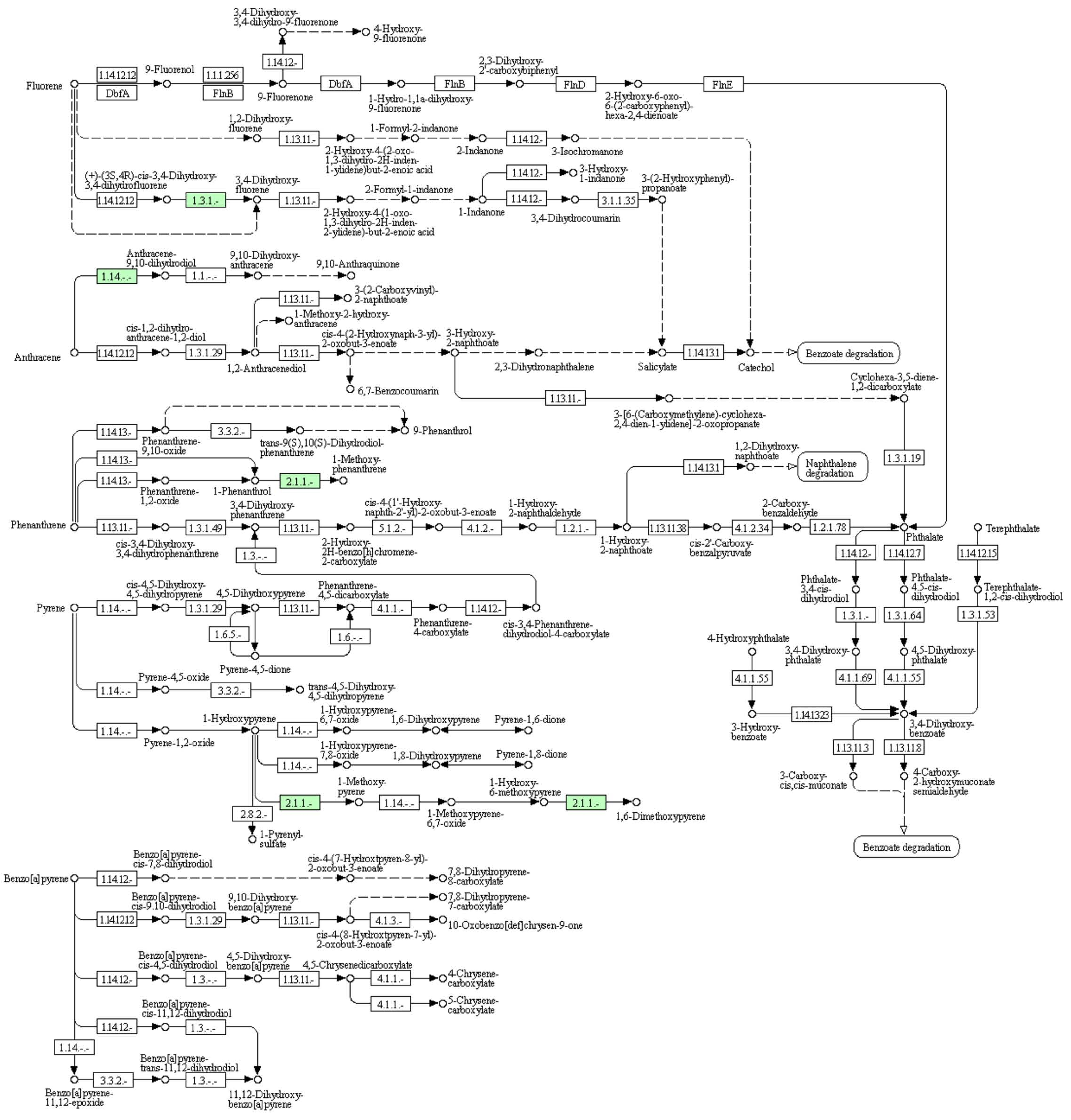

00624 11/6/12

(c) Kanehisa Laboratories

POLYKETIDE SUGAR UNIT BIOSYNTHESIS

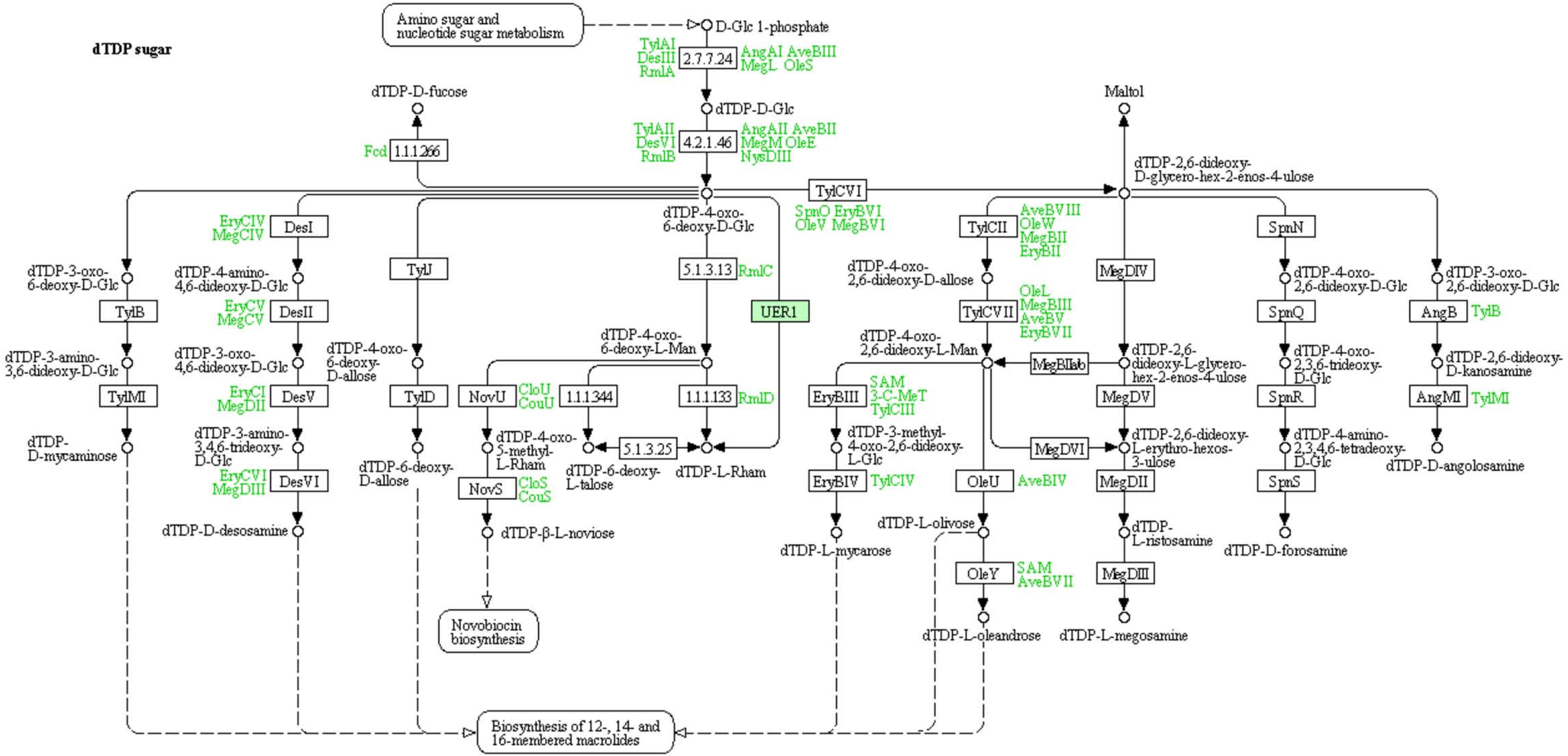

00523 2/4/14

(c) Kanehisa Laboratories

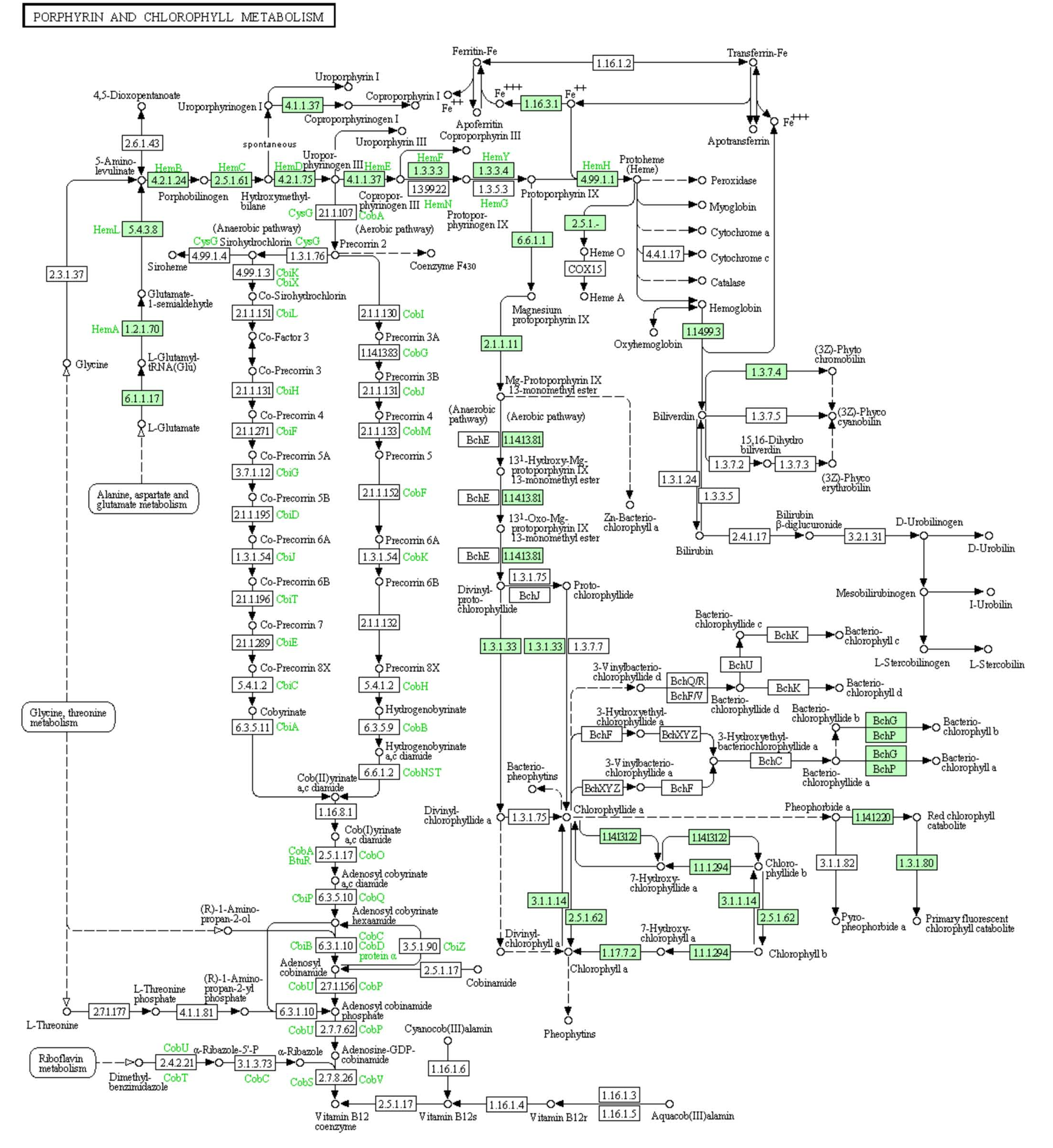

00860 2/13/14
(c) Kanehisa Laboratories

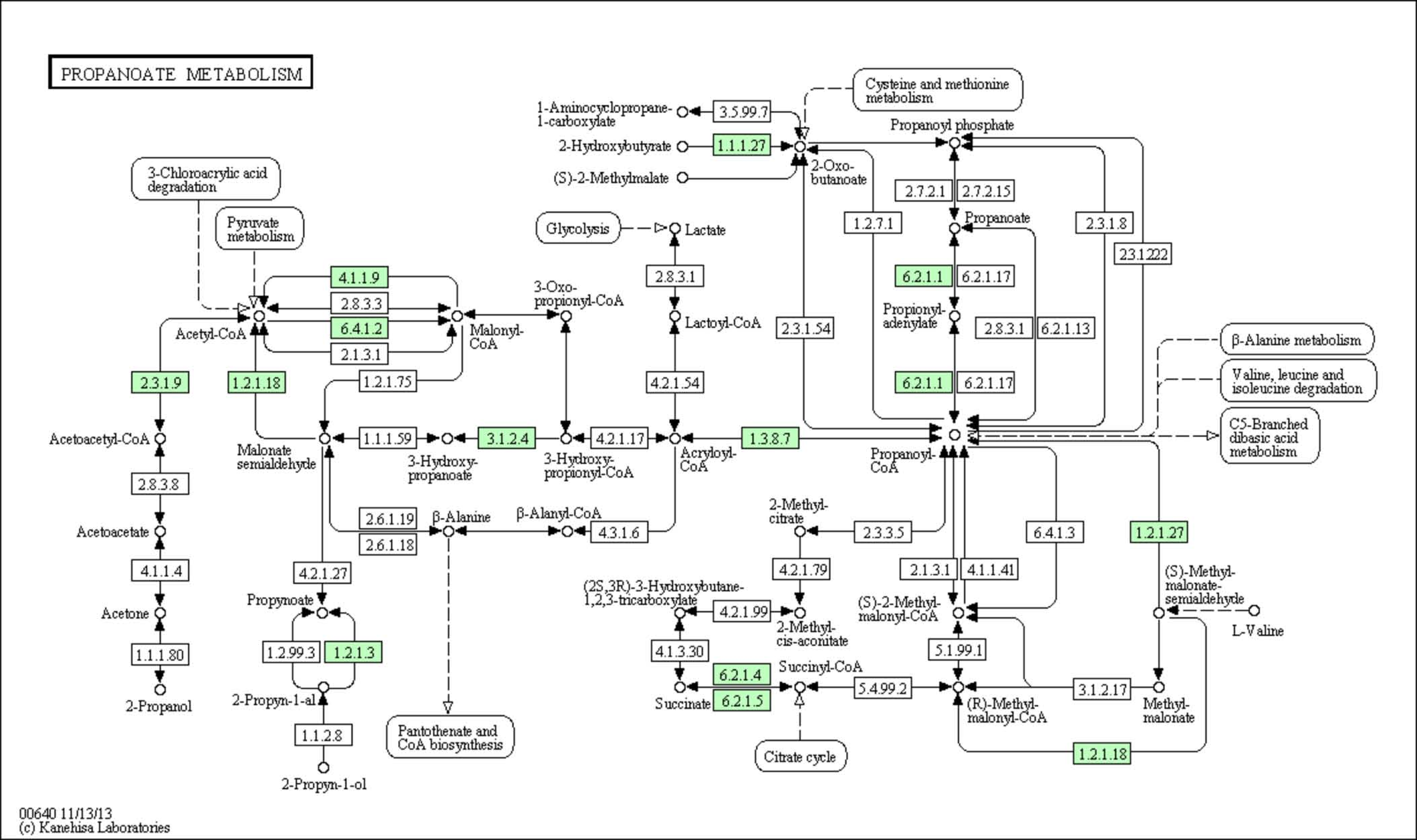
PROPANOATE METABOLISM
Cysteine and methionine metabolism
1-Aminocyclopropane-1-carboxylate
3.5.99.7
2-Hydroxybutyrate
1.1.1.27
(S)-2-Methylmalate
2-Oxo-butanoate
Propanoyl phosphate
2.7.2.1
2.7.2.15
Propanoate
1.2.7.1
2.3.1.8
2.3.1.222
6.2.1.1
6.2.1.17
Propionyl-adenylate
2.8.3.1
6.2.1.13
2.3.1.54
6.2.1.1
6.2.1.17
3-Chloroacrylic acid degradation
Pyruvate metabolism
Glycolysis
Lactate
2.8.3.1
Lactoyl-CoA
4.2.1.54
4.1.1.9
2.8.3.3
6.4.1.2
2.1.3.1
Acetyl-CoA
Malonyl-CoA
3-Oxo-propionyl-CoA
1.2.1.75
2.3.1.9
1.2.1.18
Acetoacetyl-CoA
Malonate semialdehyde
1.1.1.59
3-Hydroxy-propanoate
3.1.2.4
3-Hydroxy-propionyl-CoA
4.2.1.17
Acryloyl-CoA
1.3.8.7
Propanoyl-CoA
β-Alanine metabolism
Valine, leucine and isoleucine degradation
C5-Branched dibasic acid metabolism
2.8.3.8
Acetoacetate
4.1.1.4
Acetone
1.1.1.80
2-Propanol
2.6.1.19
2.6.1.18
β-Alanine
β-Alanyl-CoA
4.3.1.6
4.2.1.27
Propynoate
1.2.99.3
1.2.1.3
2-Propyn-1-al
1.1.2.8
2-Propyn-1-ol
Pantothenate and CoA biosynthesis
2-Methyl-citrate
2.3.3.5
4.2.1.79
(2S,3R)-3-Hydroxybutane-1,2,3-tricarboxylate
4.2.1.99
2-Methyl-cis-aconitate
4.1.3.30
Succinate
6.2.1.4
6.2.1.5
Succinyl-CoA
5.4.99.2
Citrate cycle
6.4.1.3
2.1.3.1
4.1.1.41
(S)-2-Methyl-malonyl-CoA
5.1.99.1
(R)-Methyl-malonyl-CoA
1.2.1.27
(S)-Methyl-malonate-semialdehyde
L-Valine
3.1.2.17
Methyl-malonate
1.2.1.18
00640 11/13/13
(c) Kanehisa Laboratories

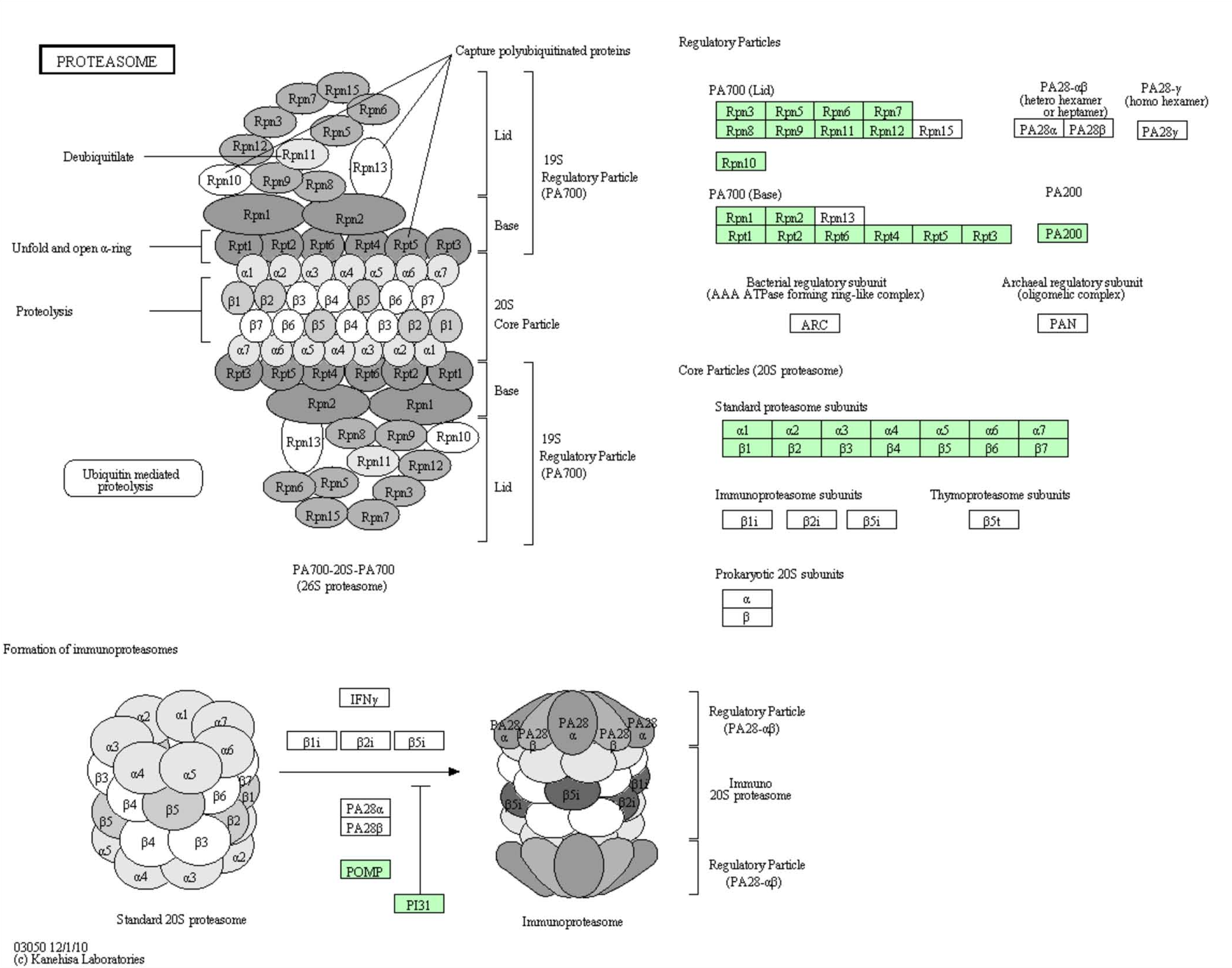
PROTEASOME
Capture polyubiquitinated proteins
Deubiquitilate
Unfold and open α-ring
Proteolysis
Lid
Base
19S Regulatory Particle (PA700)
20S Core Particle
Ubiquitin mediated proteolysis
PA700-20S-PA700 (26S proteasome)
Regulatory Particles
PA700 (Lid)
Rpn3 Rpn5 Rpn6 Rpn7
Rpn8 Rpn9 Rpn11 Rpn12 Rpn15
Rpn10
PA28-αβ (hetero hexamer or heptamer)
PA28α PA28β
PA28-γ (homo hexamer)
PA28γ
PA700 (Base)
Rpn1 Rpn2 Rpn13
Rpt1 Rpt2 Rpt6 Rpt4 Rpt5 Rpt3
PA200
Bacterial regulatory subunit (AAA ATPase forming ring-like complex)
ARC
Archaeal regulatory subunit (oligomelic complex)
PAN
Core Particles (20S proteasome)
Standard proteasome subunits
α1 α2 α3 α4 α5 α6 α7
β1 β2 β3 β4 β5 β6 β7
Immunoproteasome subunits
β1i β2i β5i
Thymoproteasome subunits
β5t
Prokaryotic 20S subunits
α
β
Formation of immunoproteasomes
IFNγ
β1i β2i β5i
PA28α
PA28β
POMP
PI31
Standard 20S proteasome
Immunoproteasome
Regulatory Particle (PA28-αβ)
Immuno 20S proteasome
03050 12/1/10
(c) Kanehisa Laboratories

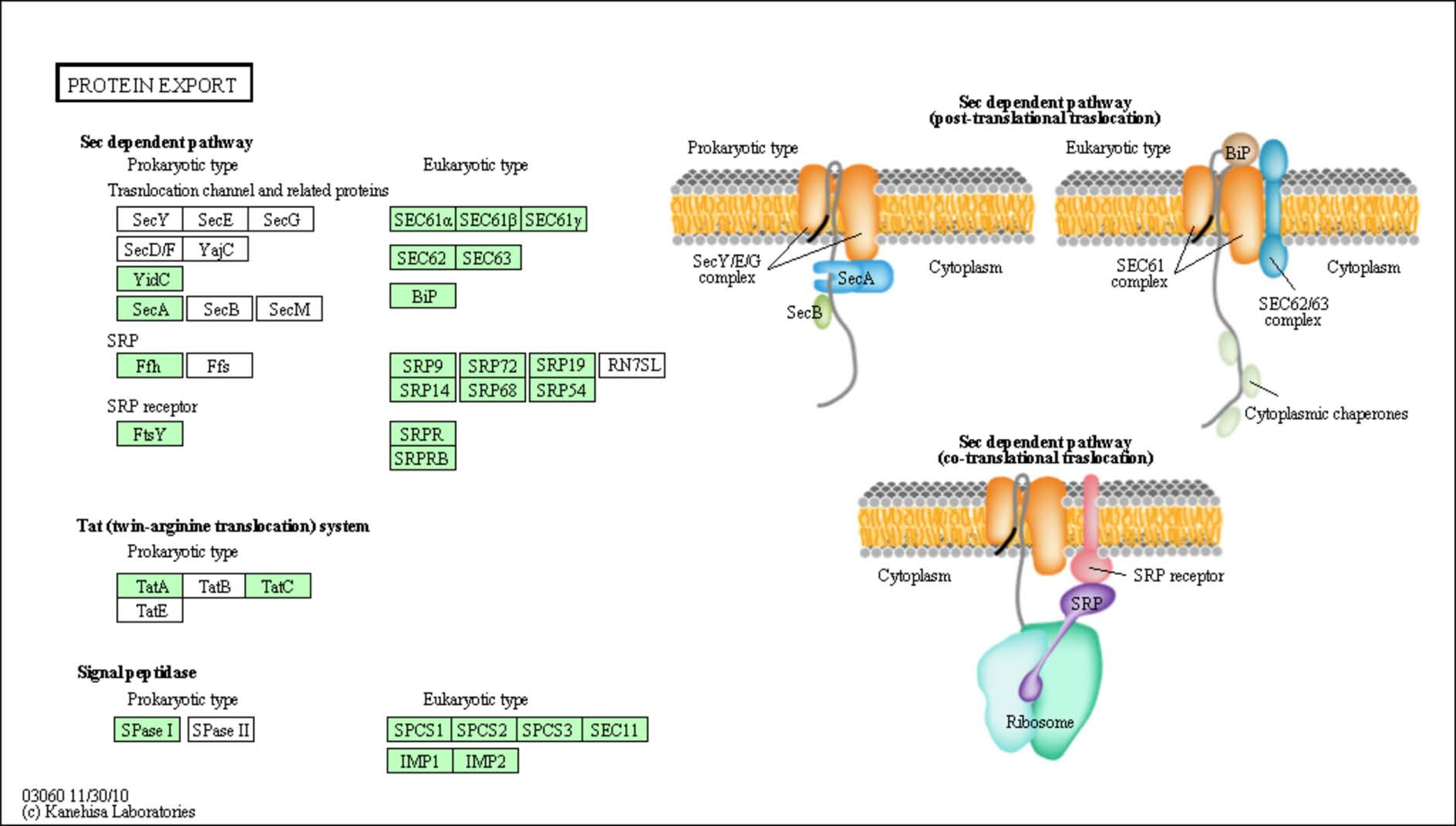
PROTEIN EXPORT
Sec dependent pathway
Prokaryotic type
Eukaryotic type
Trasnlocation channel and related proteins
SecY
SecE
SecG
SecD/F
YajC
YidC
SecA
SecB
SecM
SEC61α
SEC61β
SEC61γ
SEC62
SEC63
BiP
SRP
Ffh
Ffs
SRP9
SRP72
SRP19
RN7SL
SRP14
SRP68
SRP54
SRP receptor
FtsY
SRPR
SRPRB
Tat (twin-arginine translocation) system
Prokaryotic type
TatA
TatB
TatC
TatE
Signal peptidase
Prokaryotic type
SPase I
SPase II
Eukaryotic type
SPCS1
SPCS2
SPCS3
SEC11
IMP1
IMP2
Sec dependent pathway
(post-translational traslocation)
Prokaryotic type
SecY/E/G
complex
SecA
SecB
Cytoplasm
Eukaryotic type
BiP
SEC61
complex
SEC62/63
complex
Cytoplasm
Cytoplasmic chaperones
Sec dependent pathway
(co-translational traslocation)
Cytoplasm
SRP receptor
SRP
Ribosome
03060 11/30/10
(c) Kanehisa Laboratories

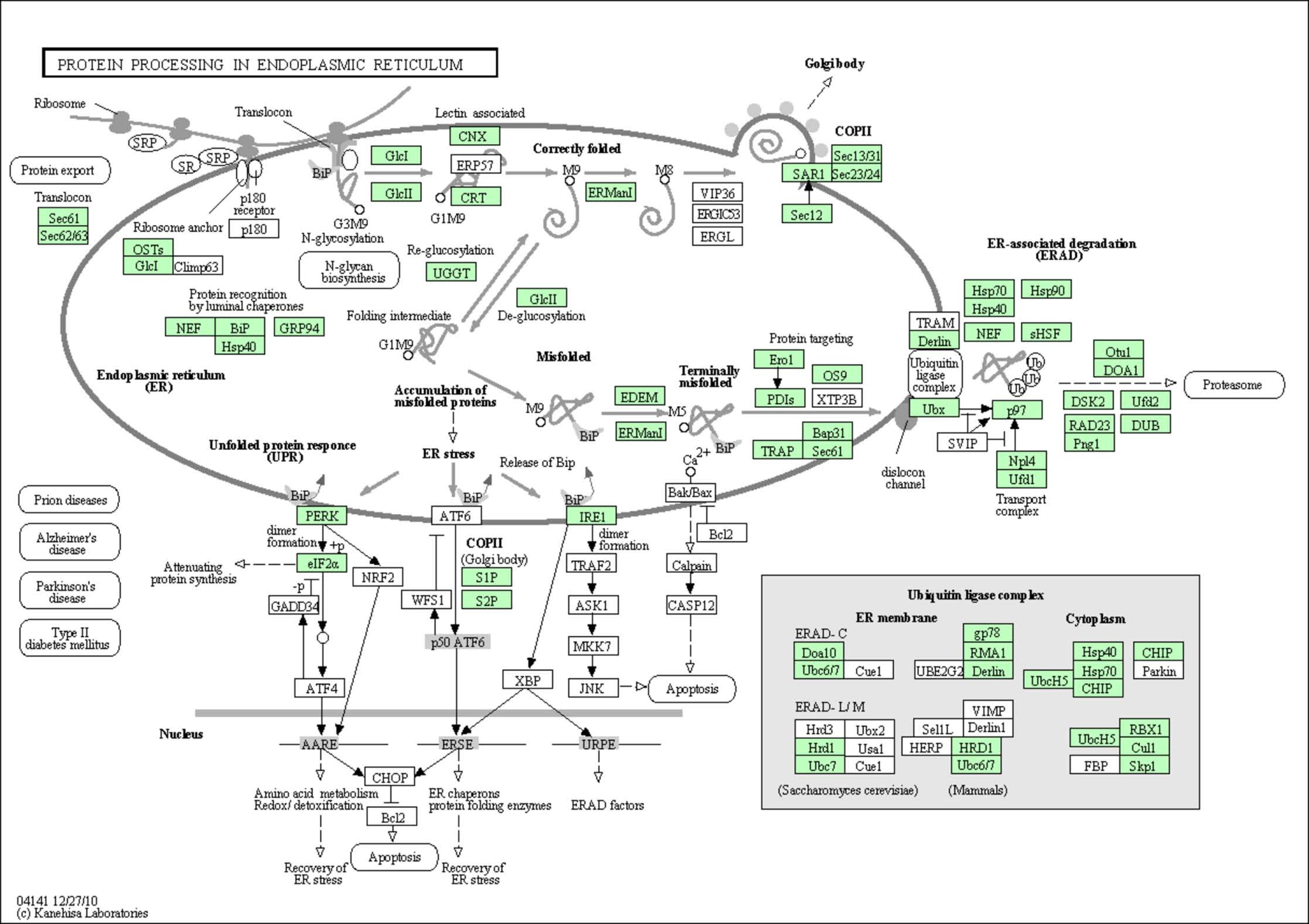
PROTEIN PROCESSING IN ENDOPLASMIC RETICULUM
Ribosome
SRP
SR
SRP
Translocon
BiP
p180 receptor
p180
Ribosome anchor
Protein export
Translocon
Sec61
Sec62/63
OSTs
GlcI
Climp63
G3M9
N-glycosylation
N-glycan biosynthesis
Lectin associated
CNX
GlcI
ERP57
GlcII
CRT
G1M9
Correctly folded
M9
ERManI
M8
VIP36
ERGIC53
ERGL
Re-glucosylation
UGGT
GlcII
De-glucosylation
Protein recognition by luminal chaperones
NEF
BiP
Hsp40
GRP94
Folding intermediate
G1M9
Misfolded
Endoplasmic reticulum (ER)
Accumulation of misfolded proteins
ER stress
M9
BiP
EDEM
ERManI
M5
Terminally misfolded
BiP
Protein targeting
Ero1
PDIs
OS9
XTP3B
Bap31
TRAP
Sec61
Golgi body
COPII
Sec13/31
SAR1
Sec23/24
Sec12
ER-associated degradation (ERAD)
Hsp70
Hsp40
Hsp90
NEF
sHSF
TRAM
Derlin
Ubiquitin ligase complex
Ub
Otu1
DOA1
Proteasome
Ubx
p97
SVIP
Npl4
Ufd1
Transport complex
dislocon channel
DSK2
Ufd2
RAD23
Png1
DUB
Unfolded protein responce (UPR)
Release of Bip
BiP
PERK
BiP
ATF6
BiP
IRE1
Ca2+
Bak/Bax
Bcl2
dimer formation
+p
eIF2α
NRF2
Attenuating protein synthesis
-p
GADD34
ATF4
WFS1
p50 ATF6
COPII (Golgi body)
S1P
S2P
dimer formation
TRAF2
ASK1
MKK7
JNK
XBP
Apoptosis
Calpain
CASP12
Nucleus
AARE
ERSE
URPE
CHOP
Bcl2
Amino acid metabolism
Redox/ detoxification
ER chaperons
protein folding enzymes
ERAD factors
Apoptosis
Recovery of ER stress
Recovery of ER stress
Prion diseases
Alzheimer's disease
Parkinson's disease
Type II diabetes mellitus
Ubiquitin ligase complex
ER membrane
Cytoplasm
ERAD- C
Doa10
Ubc6/7
Cue1
gp78
RMA1
UBE2G2
Derlin
Hsp40
Hsp70
CHIP
UbcH5
CHIP
Parkin
ERAD- L/ M
Hrd3
Ubx2
Hrd1
Usa1
Ubc7
Cue1
VIMP
Sel1L
Derlin1
HERP
HRD1
Ubc6/7
UbcH5
RBX1
Cul1
FBP
Skp1
(Saccharomyces cerevisiae)
(Mammals)
04141 12/27/10
(c) Kanehisa Laboratories

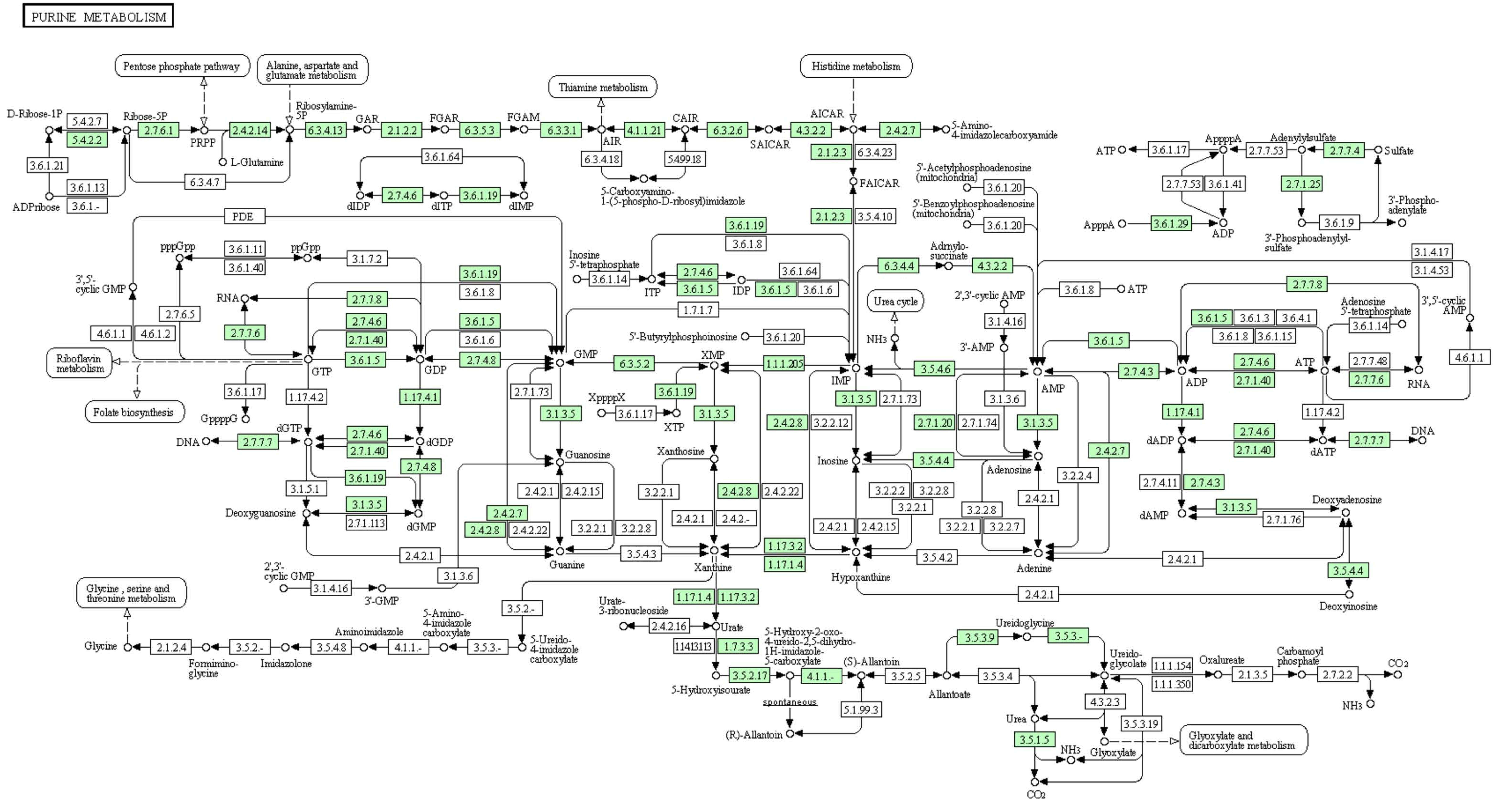

00230 11/7/13
(c) Kanehisa Laboratories

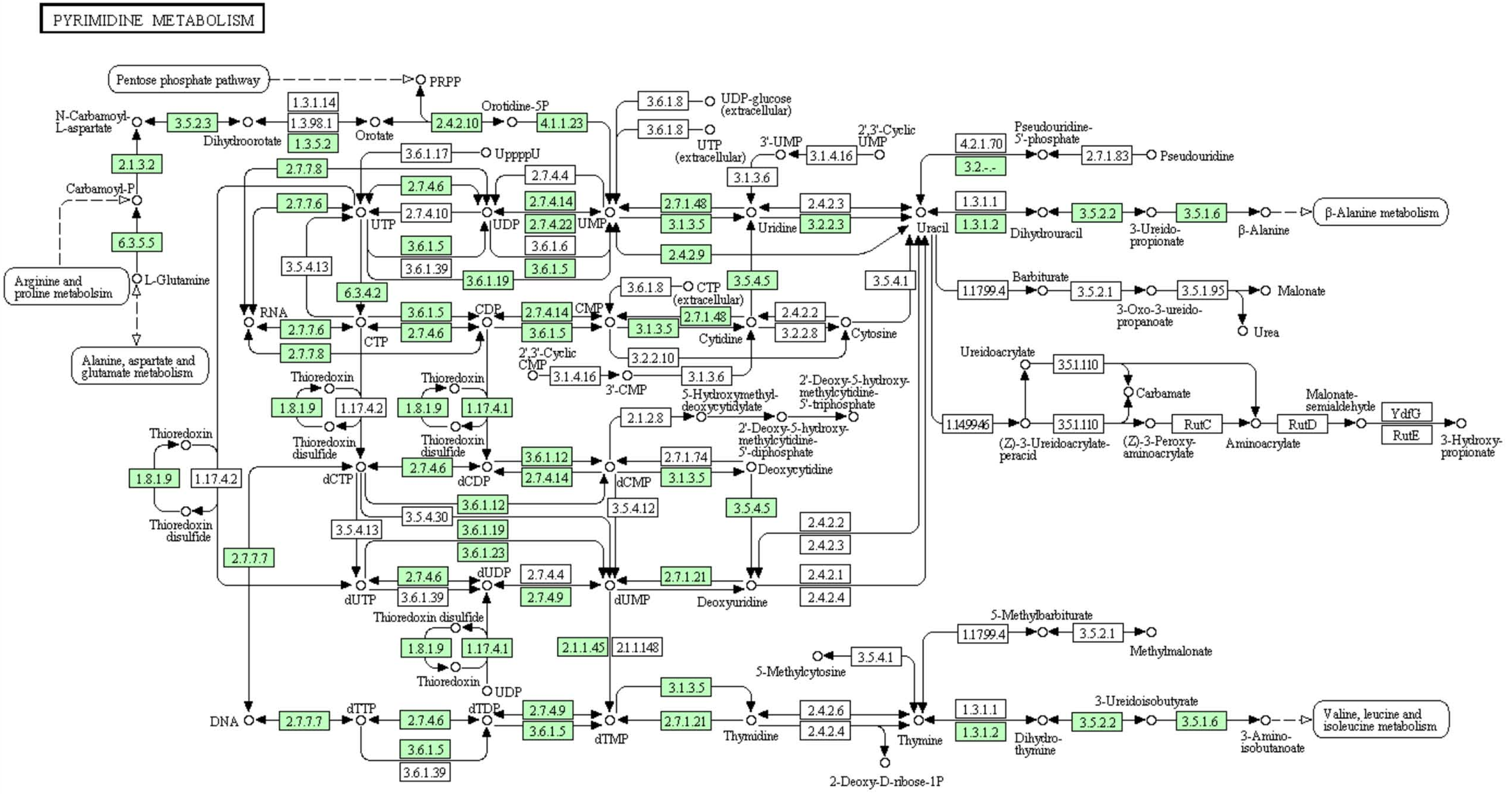

00240 11/13/13
(c) Kanehisa Laboratories

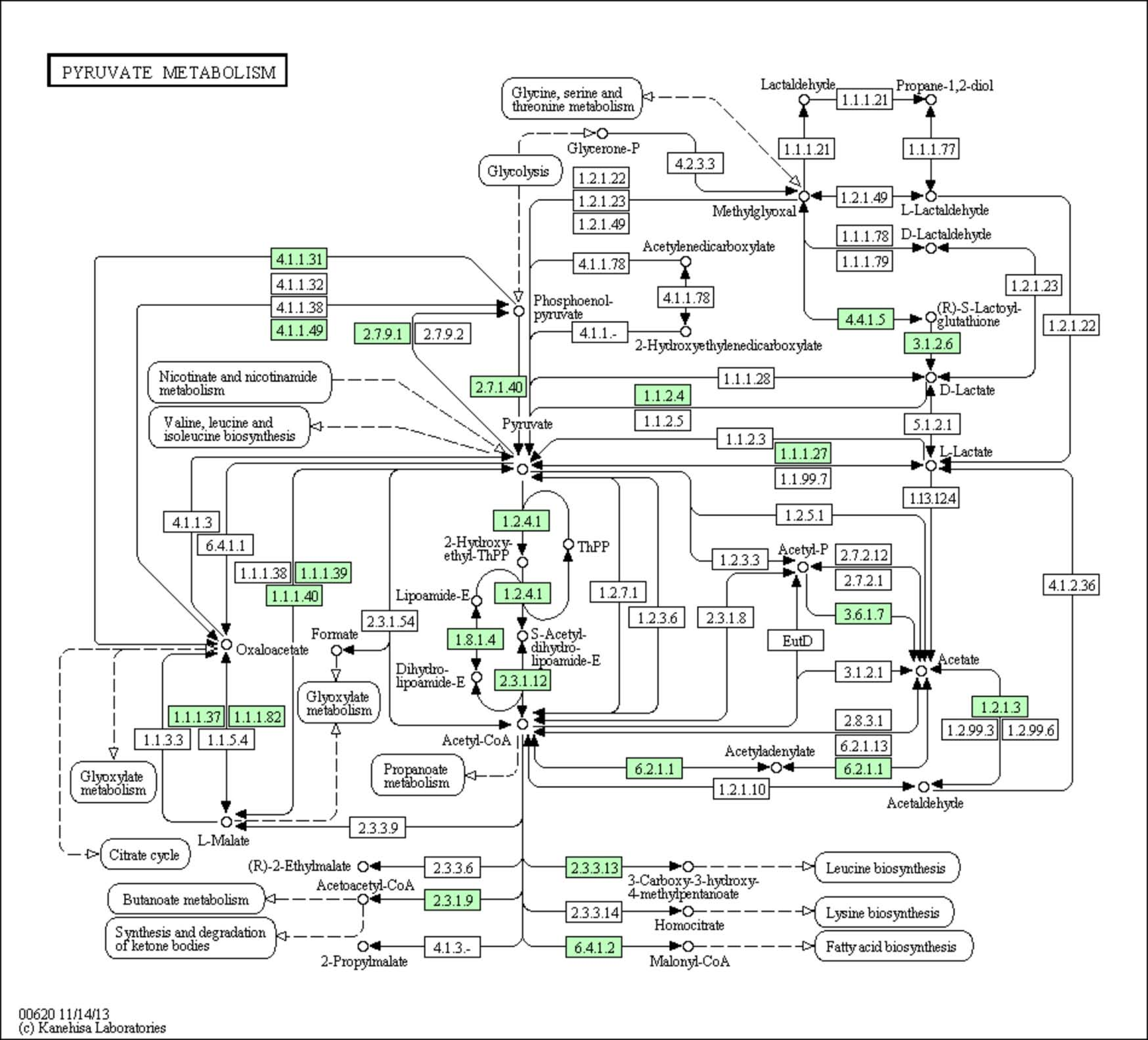
PYRUVATE METABOLISM
Glycine, serine and threonine metabolism
Lactaldehyde
Propane-1,2-diol
1.1.1.21
Glycerone-P
Glycolysis
4.2.3.3
1.1.1.21
1.1.1.77
1.2.1.22
1.2.1.23
1.2.1.49
Methylglyoxal
1.2.1.49
L-Lactaldehyde
1.1.1.78
D-Lactaldehyde
1.1.1.79
Acetylenedicarboxylate
4.1.1.31
4.1.1.78
4.1.1.32
4.1.1.38
4.1.1.49
Phosphoenol-pyruvate
4.1.1.78
4.1.1.-
2-Hydroxyethylenedicarboxylate
1.2.1.23
4.4.1.5
(R)-S-Lactoyl-glutathione
1.2.1.22
3.1.2.6
2.7.9.1
2.7.9.2
Nicotinate and nicotinamide metabolism
2.7.1.40
1.1.1.28
D-Lactate
1.1.2.4
1.1.2.5
Valine, leucine and isoleucine biosynthesis
Pyruvate
5.1.2.1
1.1.2.3
1.1.1.27
L-Lactate
1.1.99.7
1.13.12.4
1.2.5.1
4.1.1.3
1.2.4.1
6.4.1.1
2-Hydroxy-ethyl-ThPP
ThPP
1.2.3.3
Acetyl-P
2.7.2.12
1.1.1.38
1.1.1.39
2.7.2.1
1.1.1.40
1.2.4.1
1.2.7.1
4.1.2.36
Lipoamide-E
1.2.3.6
2.3.1.8
3.6.1.7
2.3.1.54
Formate
S-Acetyl-dihydro-lipoamide-E
EutD
1.8.1.4
Oxaloacetate
Acetate
3.1.2.1
Dihydro-lipoamide-E
2.3.1.12
Glyoxylate metabolism
1.2.1.3
1.1.1.37
1.1.1.82
2.8.3.1
1.2.99.3
1.2.99.6
Acetyl-CoA
1.1.3.3
1.1.5.4
6.2.1.13
Glyoxylate metabolism
Acetyladenylate
Propanoate metabolism
6.2.1.1
6.2.1.1
1.2.1.10
Acetaldehyde
2.3.3.9
L-Malate
Citrate cycle
(R)-2-Ethylmalate
2.3.3.6
2.3.3.13
Leucine biosynthesis
3-Carboxy-3-hydroxy-4-methylpentanoate
Acetoacetyl-CoA
Butanoate metabolism
2.3.1.9
2.3.3.14
Lysine biosynthesis
Homocitrate
Synthesis and degradation of ketone bodies
2-Propylmalate
4.1.3.-
6.4.1.2
Malonyl-CoA
Fatty acid biosynthesis
00620 11/14/13
(c) Kanehisa Laboratories

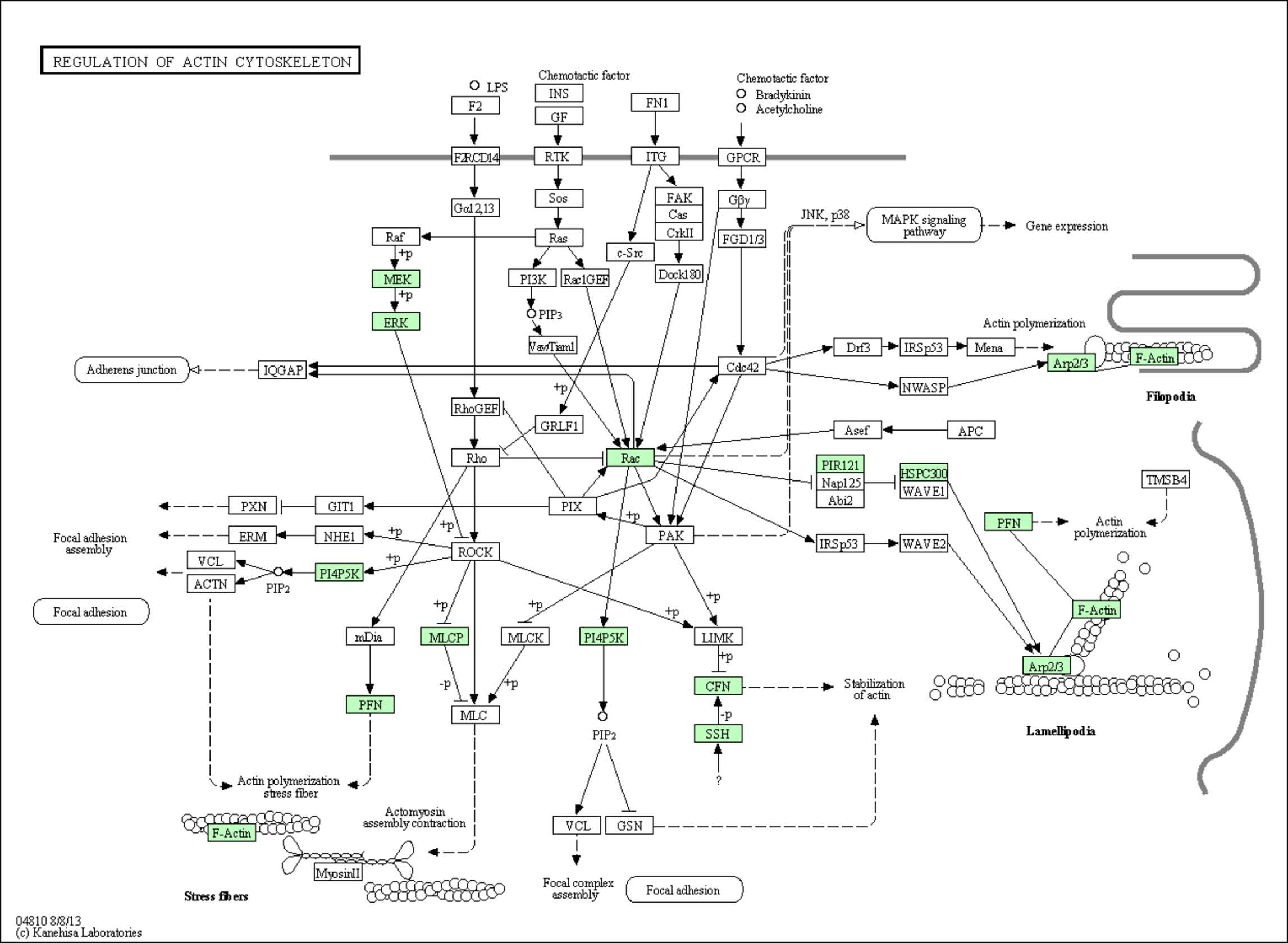
REGULATION OF ACTIN CYTOSKELETON
LPS
F2
Chemotactic factor
INS
GF
FN1
Chemotactic factor
Bradykinin
Acetylcholine
F2R
CD14
RTK
ITG
GPCR
Gα12,13
Sos
FAK
Cas
CrkII
Gβγ
JNK, p38
MAPK signaling pathway
Gene expression
Raf
+p
Ras
c-Src
FGD1/3
MEK
+p
PI3K
Rac1GEF
Dock180
ERK
PIP3
Vav/Tiam1
Actin polymerization
Drf3
IRSp53
Mena
Arp2/3
F-Actin
Adherens junction
IQGAP
Cdc42
NWASP
Filopodia
+p
RhoGEF
GRLF1
Asef
APC
Rho
Rac
PIR121
Nap125
Abi2
HSPC300
WAVE1
TMSB4
PXN
GIT1
PIX
PFN
Actin polymerization
+p
+p
Focal adhesion assembly
ERM
NHE1
+p
ROCK
PAK
IRSp53
WAVE2
VCL
ACTN
+p
PI4P5K
PIP2
Focal adhesion
+p
+p
+p
+p
F-Actin
mDia
MLCP
MLCK
PI4P5K
LIMK
+p
Arp2/3
PFN
-p
+p
MLC
CFN
Stabilization of actin
PIP2
-p
SSH
Lamellipodia
?
Actin polymerization stress fiber
Actomyosin assembly contraction
F-Actin
VCL
GSN
MyosinII
Focal complex assembly
Focal adhesion
Stress fibers
04810 8/8/13
(c) Kanehisa Laboratories

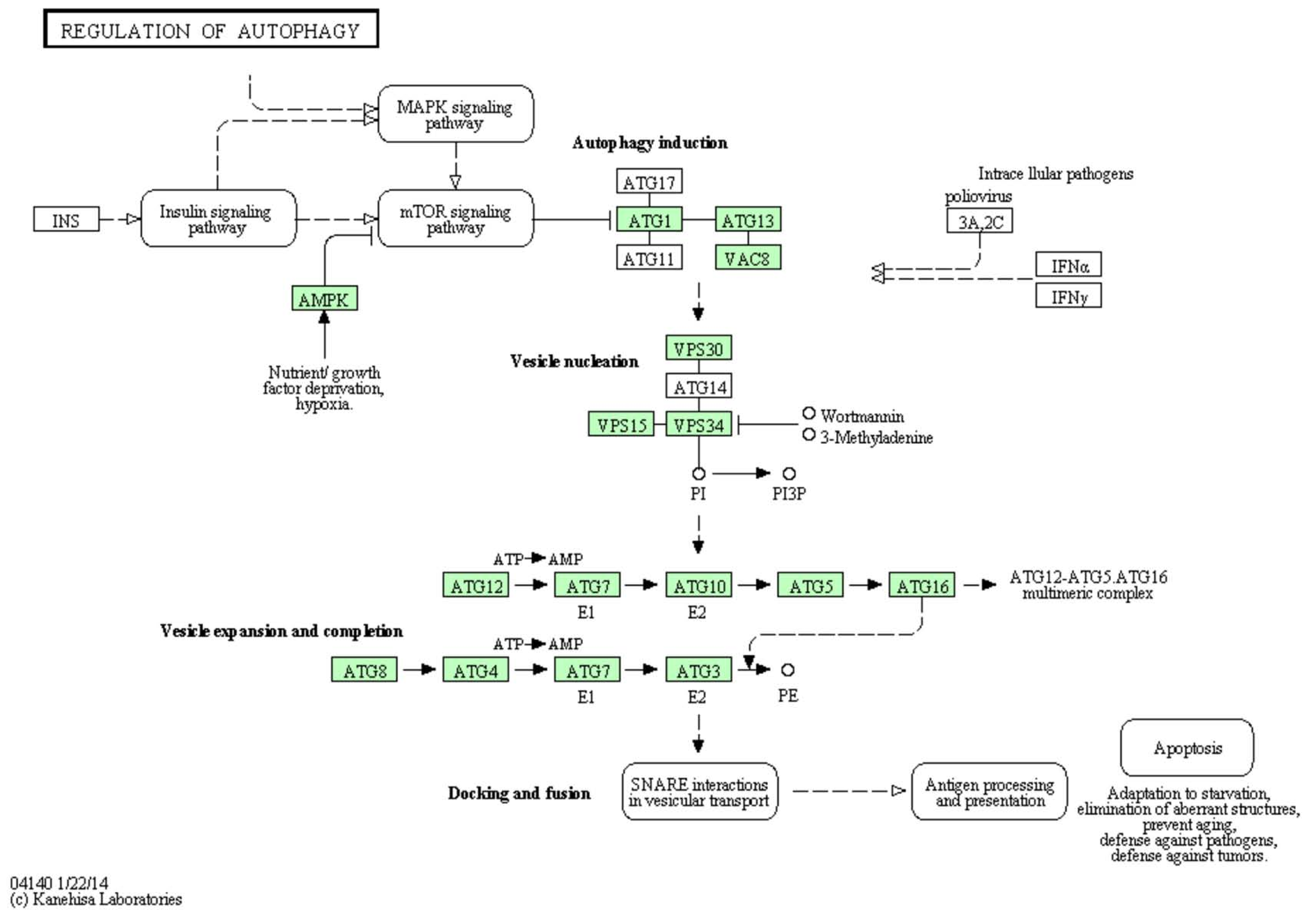
REGULATION OF AUTOPHAGY
MAPK signaling pathway
Autophagy induction
Intracellular pathogens
poliovirus
3A,2C
INS
Insulin signaling pathway
mTOR signaling pathway
ATG17
ATG1
ATG13
ATG11
VAC8
IFNα
IFNγ
AMPK
Nutrient/ growth factor deprivation, hypoxia.
Vesicle nucleation
VPS30
ATG14
VPS15
VPS34
Wortmannin
3-Methyladenine
PI
PI3P
ATP
AMP
ATG12
ATG7
ATG10
ATG5
ATG16
E1
E2
ATG12-ATG5.ATG16 multimeric complex
Vesicle expansion and completion
ATP
AMP
ATG8
ATG4
ATG7
ATG3
E1
E2
PE
Docking and fusion
SNARE interactions in vesicular transport
Antigen processing and presentation
Apoptosis
Adaptation to starvation, elimination of aberrant structures, prevent aging, defense against pathogens, defense against tumors.
04140 1/22/14
(c) Kanehisa Laboratories

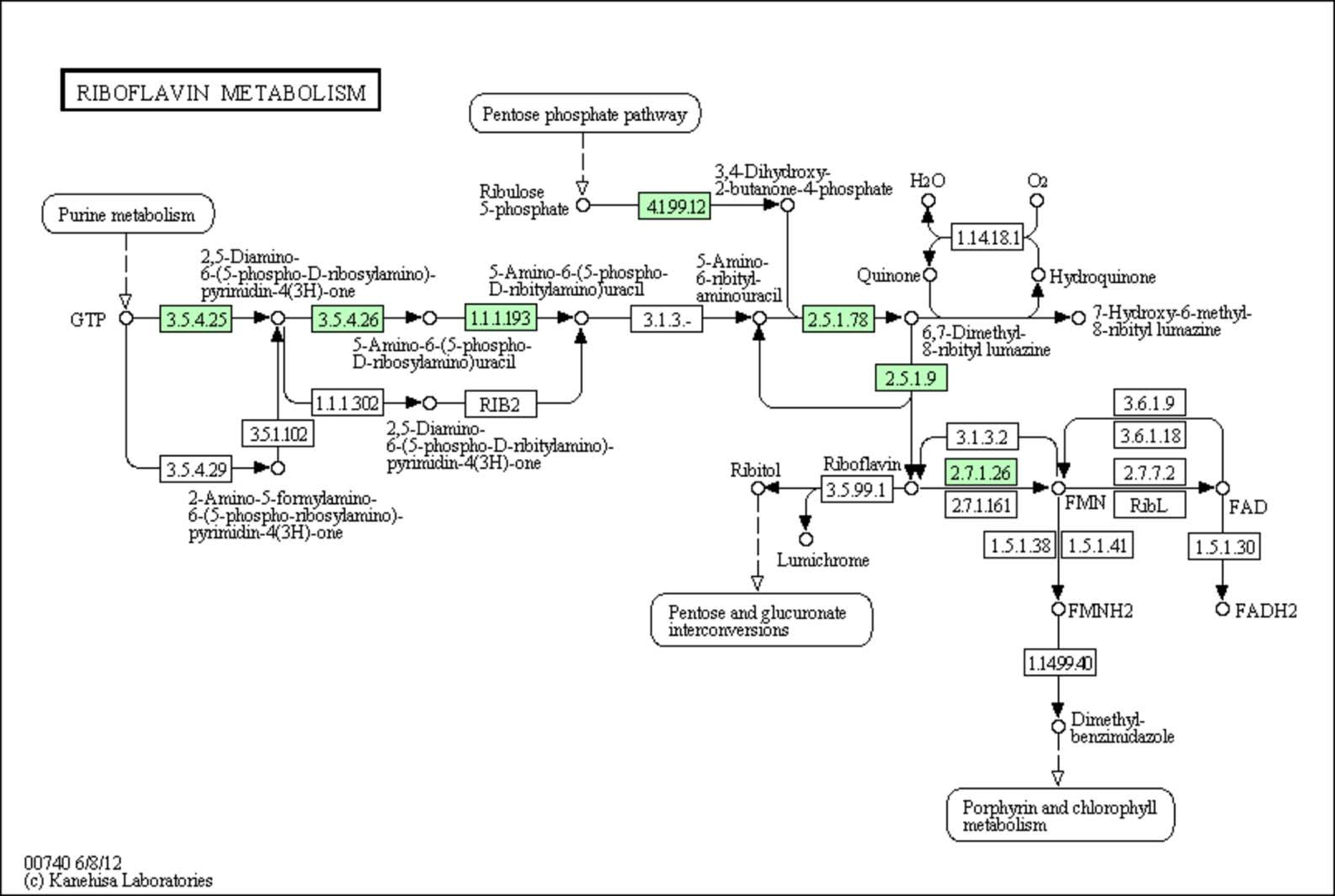

RIBOFLAVIN METABOLISM
Pentose phosphate pathway
Purine metabolism
Ribulose 5-phosphate
4.1.99.12
3,4-Dihydroxy-2-butanone-4-phosphate
H2O
O2
1.14.18.1
Quinone
Hydroquinone
GTP
3.5.4.25
2,5-Diamino-6-(5-phospho-D-ribosylamino)-pyrimidin-4(3H)-one
3.5.4.26
5-Amino-6-(5-phospho-D-ribosylamino)uracil
1.1.1.193
5-Amino-6-(5-phospho-D-ribitylamino)uracil
3.1.3.-
5-Amino-6-ribityl-aminouracil
2.5.1.78
6,7-Dimethyl-8-ribityl lumazine
7-Hydroxy-6-methyl-8-ribityl lumazine
2.5.1.9
1.1.1.302
RIB2
2,5-Diamino-6-(5-phospho-D-ribitylamino)-pyrimidin-4(3H)-one
3.5.1.102
3.5.4.29
2-Amino-5-formylamino-6-(5-phospho-ribosylamino)-pyrimidin-4(3H)-one
Ribitol
Riboflavin
3.5.99.1
Lumichrome
3.1.3.2
2.7.1.26
2.7.1.161
FMN
3.6.1.9
3.6.1.18
2.7.7.2
RibL
FAD
1.5.1.38
1.5.1.41
1.5.1.30
FMNH2
FADH2
1.14.99.40
Dimethyl-benzimidazole
Pentose and glucuronate interconversions
Porphyrin and chlorophyll metabolism
00740 6/8/12
(c) Kanehisa Laboratories

RIBOSOME BIOGENESIS IN EUKARYOTES

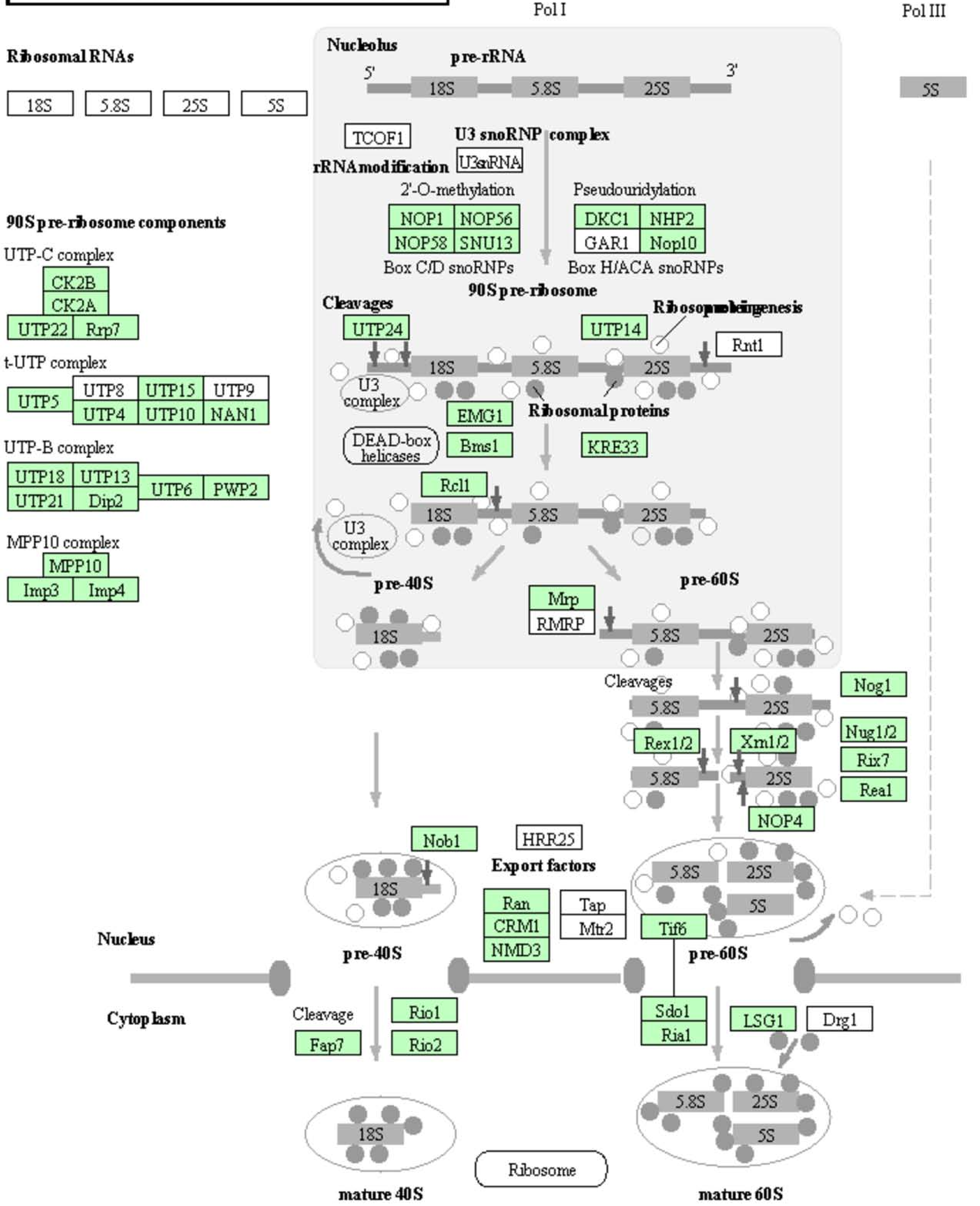

03008 4/13/11
(c) Kanehisa Laboratories

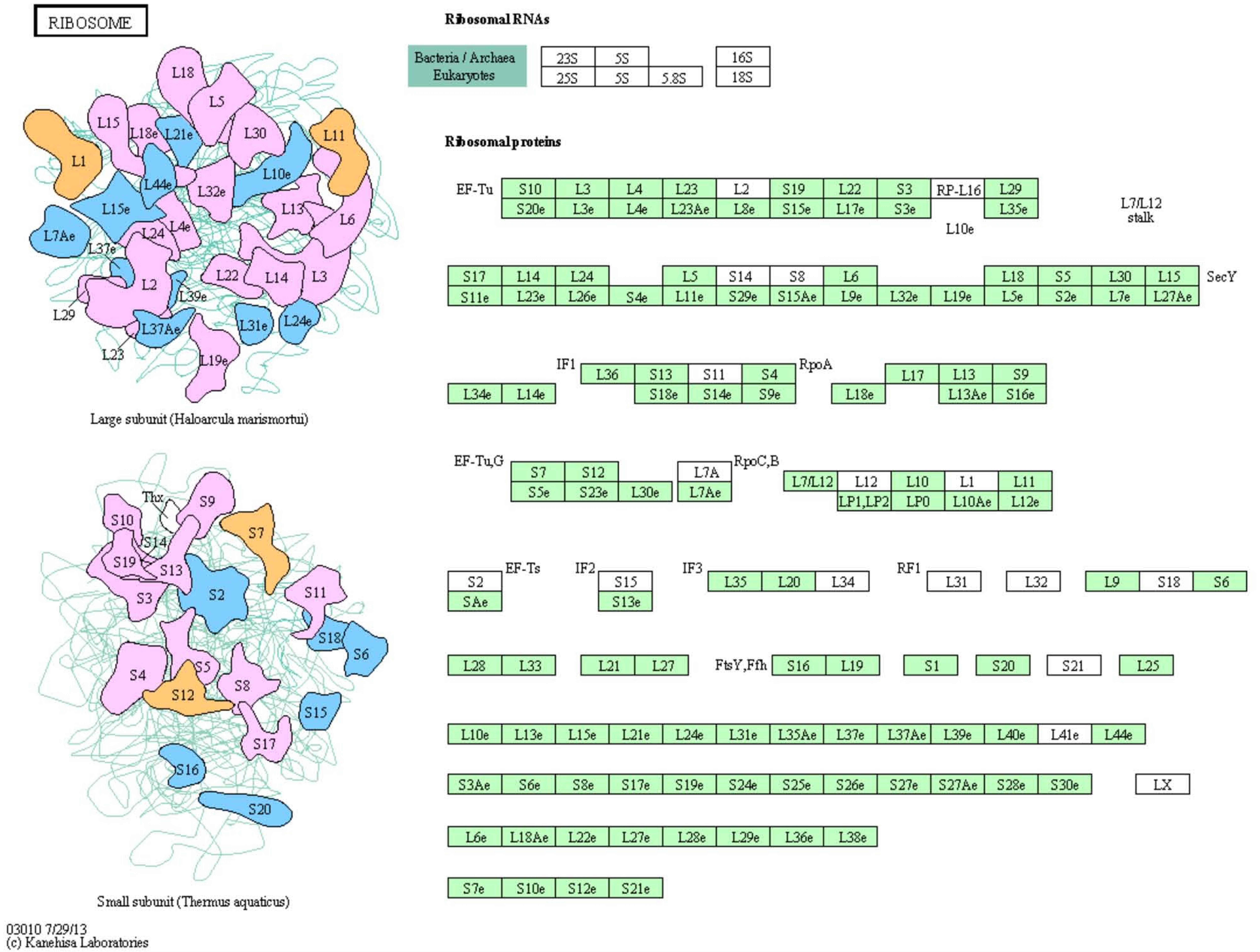
RIBOSOME
Ribosomal RNAs
Bacteria / Archaea
Eukaryotes
23S
5S
16S
25S
5S
5.8S
18S
Ribosomal proteins
EF-Tu
S10 S20e
L3 L3e
L4 L4e
L23 L23Ae
L2 L8e
S19 S15e
L22 L17e
S3 S3e
RP-L16
L10e
L29 L35e
L7/L12
stalk
S17 S11e
L14 L23e
L24 L26e
S4e
L5 L11e
S14 S29e
S8 S15Ae
L6 L9e
L32e
L19e
L18 L5e
S5 S2e
L30 L7e
L15 L27Ae
SecY
IF1
L34e
L14e
L36
S13 S18e
S11 S14e
S4 S9e
RpoA
L18e
L17
L13 L13Ae
S9 S16e
EF-Tu,G
S7 S5e
S12 S23e
L30e
L7A L7Ae
RpoC,B
L7/L12
L12 LP1,LP2
L10 LP0
L1 L10Ae
L11 L12e
S2 SAe
EF-Ts
IF2
S15 S13e
IF3
L35
L20
L34
RF1
L31
L32
L9
S18
S6
L28
L33
L21
L27
FtsY,Ffh
S16
L19
S1
S20
S21
L25
L10e L13e L15e L21e L24e L31e L35Ae L37e L37Ae L39e L40e L41e L44e
S3Ae S6e S8e S17e S19e S24e S25e S26e S27e S27Ae S28e S30e
LX
L6e L18Ae L22e L27e L28e L29e L36e L38e
S7e S10e S12e S21e
L18 L5 L15 L18e L21e L30 L11 L1 L10e L44e L32e L15e L13 L6 L7Ae L4e L24 L37e L22 L14 L3 L2 L39e L29 L37Ae L31e L24e L23 L19e
Large subunit (Haloarcula marismortui)
Thx S9 S10 S14 S7 S19 S13 S2 S3 S11 S18 S6 S4 S5 S12 S8 S15 S17 S16 S20
Small subunit (Thermus aquaticus)
03010 7/29/13
(c) Kanehisa Laboratories

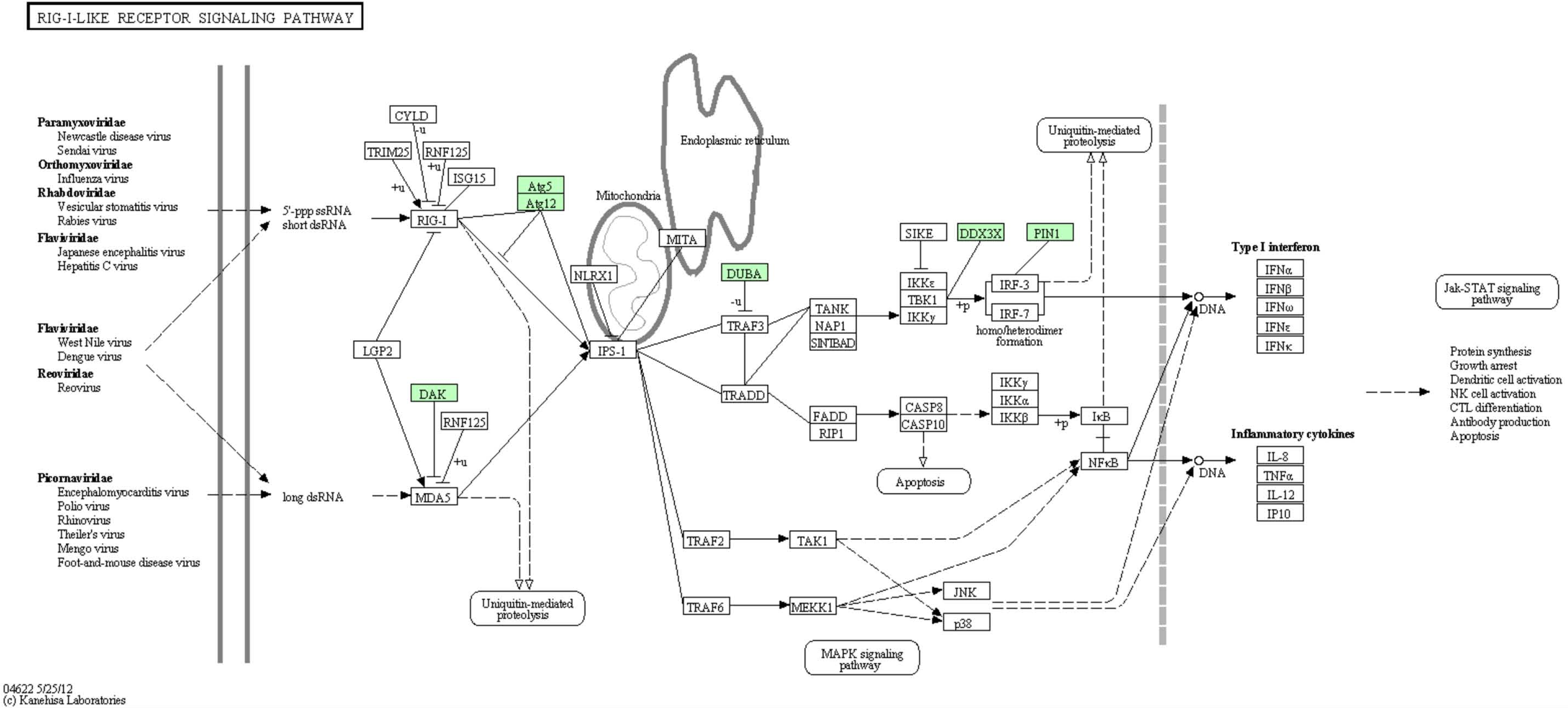
RIG-I-LIKE RECEPTOR SIGNALING PATHWAY
Paramyxoviridae
Newcastle disease virus
Sendai virus
Orthomyxoviridae
Influenza virus
Rhabdoviridae
Vesicular stomatitis virus
Rabies virus
Flaviviridae
Japanese encephalitis virus
Hepatitis C virus
Flaviviridae
West Nile virus
Dengue virus
Reoviridae
Reovirus
Picornaviridae
Encephalomyocarditis virus
Polio virus
Rhinovirus
Theiler's virus
Mengo virus
Foot-and-mouse disease virus
5'-ppp ssRNA
short dsRNA
long dsRNA
CYLD
-u
TRIM25
RNF125
+u
ISG15
+u
RIG-I
Atg5
Atg12
LGP2
DAK
RNF125
+u
MDA5
Uniquitin-mediated proteolysis
Mitochondria
Endoplasmic reticulum
MITA
NLRX1
IPS-1
DUBA
-u
TRAF3
TRADD
TANK
NAP1
SINTBAD
SIKE
DDX3X
PIN1
IKKε
TBK1
IKKγ
+p
IRF-3
IRF-7
homo/heterodimer formation
Uniquitin-mediated proteolysis
FADD
RIP1
CASP8
CASP10
Apoptosis
IKKγ
IKKα
IKKβ
+p
IκB
NFκB
TRAF2
TAK1
TRAF6
MEKK1
JNK
p38
MAPK signaling pathway
DNA
DNA
Type I interferon
IFNα
IFNβ
IFNω
IFNε
IFNκ
Inflammatory cytokines
IL-8
TNFα
IL-12
IP10
Jak-STAT signaling pathway
Protein synthesis
Growth arrest
Dendritic cell activation
NK cell activation
CTL differentiation
Antibody production
Apoptosis
04622 5/25/12
(c) Kanehisa Laboratories

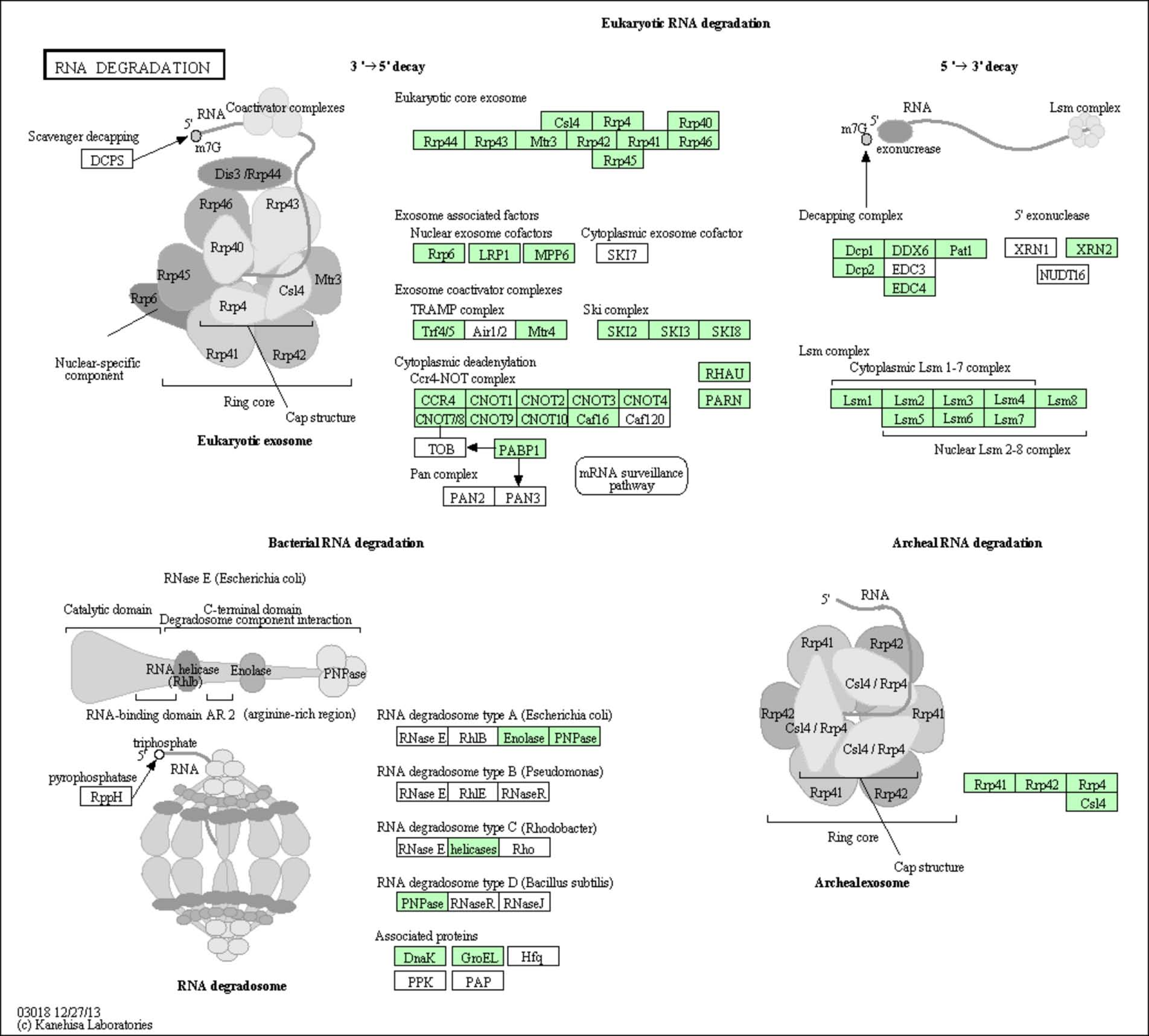
Eukaryotic RNA degradation
RNA DEGRADATION
3 '→ 5' decay
5 '→ 3' decay
Scavenger decapping
DCPS
5'
RNA
m7G
Coactivator complexes
Dis3 /Rrp44
Rrp46
Rrp43
Rrp40
Rrp45
Mtr3
Rrp6
Csl4
Rrp4
Rrp41
Rrp42
Nuclear-specific component
Ring core
Cap structure
Eukaryotic exosome
Eukaryotic core exosome
Csl4
Rrp4
Rrp40
Rrp44
Rrp43
Mtr3
Rrp42
Rrp41
Rrp46
Rrp45
Exosome associated factors
Nuclear exosome cofactors
Rrp6
LRP1
MPP6
Cytoplasmic exosome cofactor
SKI7
Exosome coactivator complexes
TRAMP complex
Trf4/5
Air1/2
Mtr4
Ski complex
SKI2
SKI3
SKI8
Cytoplasmic deadenylation
Ccr4-NOT complex
CCR4
CNOT1
CNOT2
CNOT3
CNOT4
CNOT7/8
CNOT9
CNOT10
Caf16
Caf120
RHAU
PARN
TOB
PABP1
Pan complex
PAN2
PAN3
mRNA surveillance pathway
RNA
m7G
5'
exonucrease
Lsm complex
Decapping complex
Dcp1
DDX6
Pat1
Dcp2
EDC3
EDC4
5' exonuclease
XRN1
XRN2
NUDT16
Lsm complex
Cytoplasmic Lsm 1-7 complex
Lsm1
Lsm2
Lsm3
Lsm4
Lsm8
Lsm5
Lsm6
Lsm7
Nuclear Lsm 2-8 complex
Bacterial RNA degradation
RNase E (Escherichia coli)
Catalytic domain
C-terminal domain
Degradosome component interaction
RNA helicase (Rhlb)
Enolase
PNPase
RNA-binding domain
AR 2
(arginine-rich region)
triphosphate
5'
RNA
pyrophosphatase
RppH
RNA degradosome
RNA degradosome type A (Escherichia coli)
RNase E
RhlB
Enolase
PNPase
RNA degradosome type B (Pseudomonas)
RNase E
RhlE
RNaseR
RNA degradosome type C (Rhodobacter)
RNase E
helicases
Rho
RNA degradosome type D (Bacillus subtilis)
PNPase
RNaseR
RNaseJ
Associated proteins
DnaK
GroEL
Hfq
PPK
PAP
Archeal RNA degradation
5'
RNA
Rrp41
Rrp42
Csl4 / Rrp4
Rrp42
Rrp41
Csl4 / Rrp4
Csl4 / Rrp4
Rrp41
Rrp42
Ring core
Cap structure
Archeal exosome
Rrp41
Rrp42
Rrp4
Csl4
03018 12/27/13
(c) Kanehisa Laboratories

RNA POLYMERASE

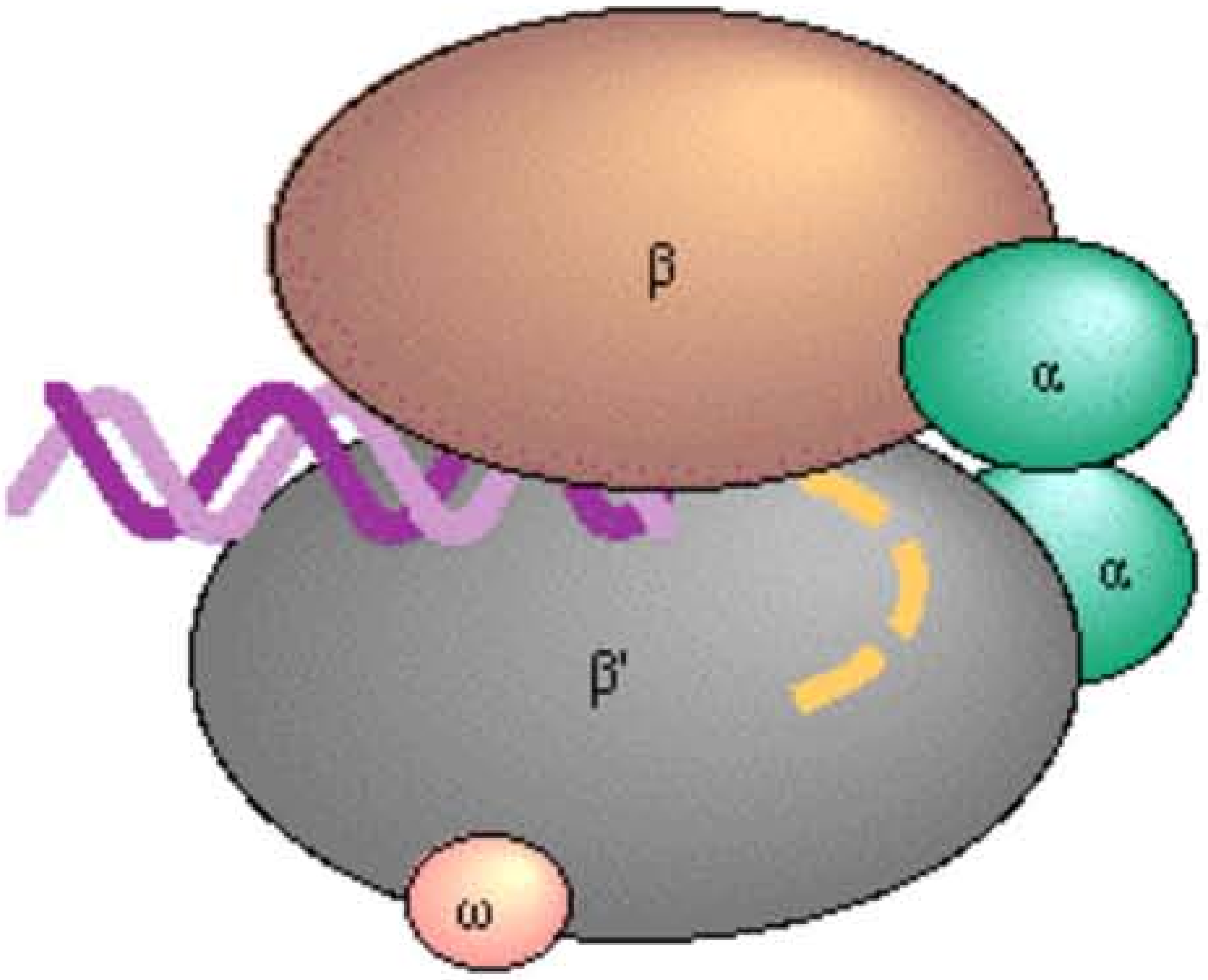

RNA polymerase (Thermus aquaticus)

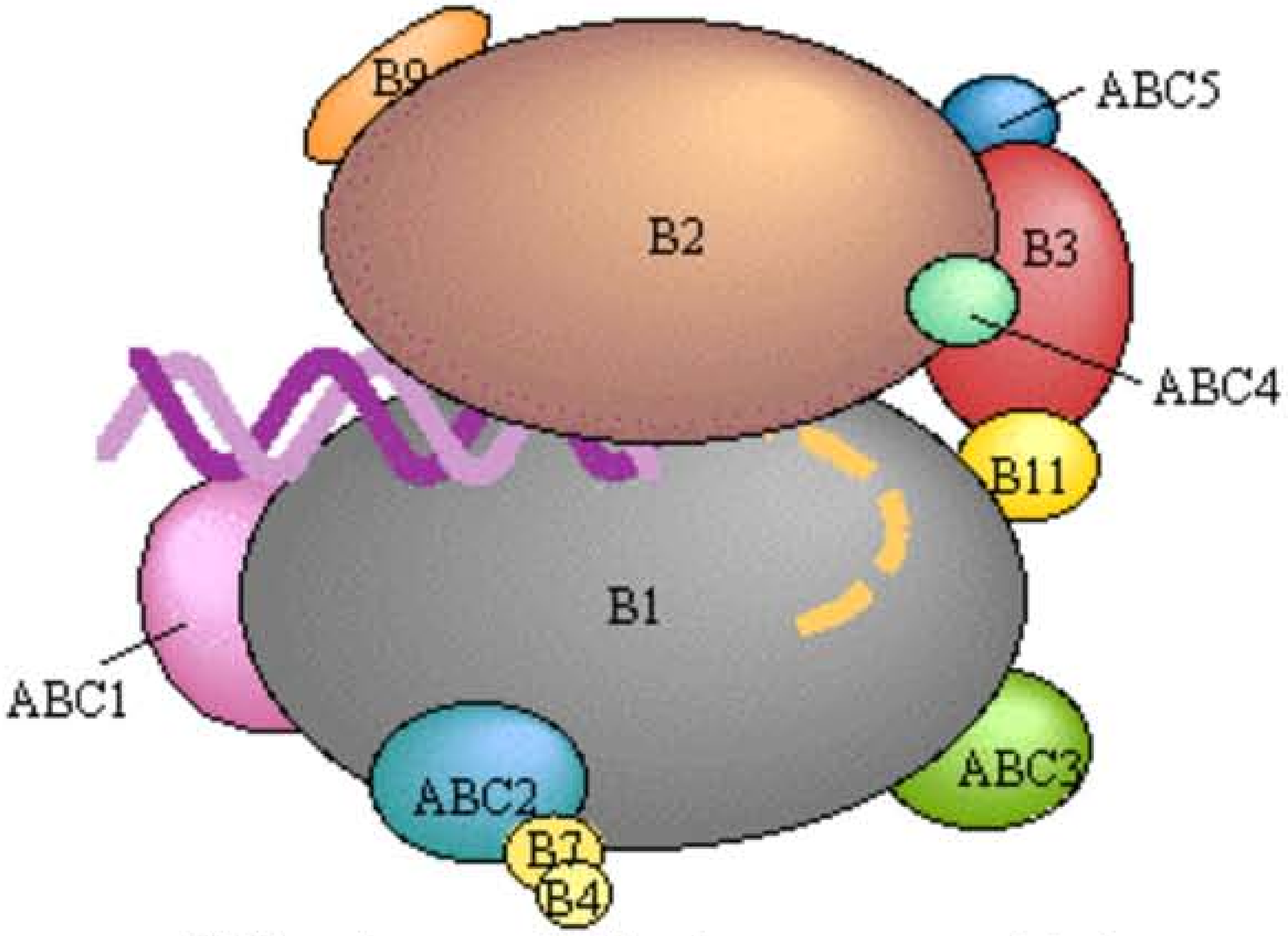

RNA polymerase II (Saccharomyces cerevisiae)

Bacterial

β			
β'	α	ω	δ

Archaeal

B					
A	D	F	H	K	E
	G		N	L	P

Eukaryotic Pol II

Core subunits

B2	B3
B1	B11

Pol II specific subunits

B4	B7	B9

Pol I, II, and III common subunits

ABC1	ABC2	ABC3
ABC4	ABC5	

Eukaryotic Pol III

Core subunits

C2	AC2
C1	AC1

Pol III specific subunits

C3	C4	C11	
C25	C31	C34	C37

Eukaryotic Pol I

Core subunits

A2	AC2
A1	AC1

Pol I specific subunits

A12	A14	A34
A49	A43	

03020 3/25/11
(c) Kanehisa Laboratories

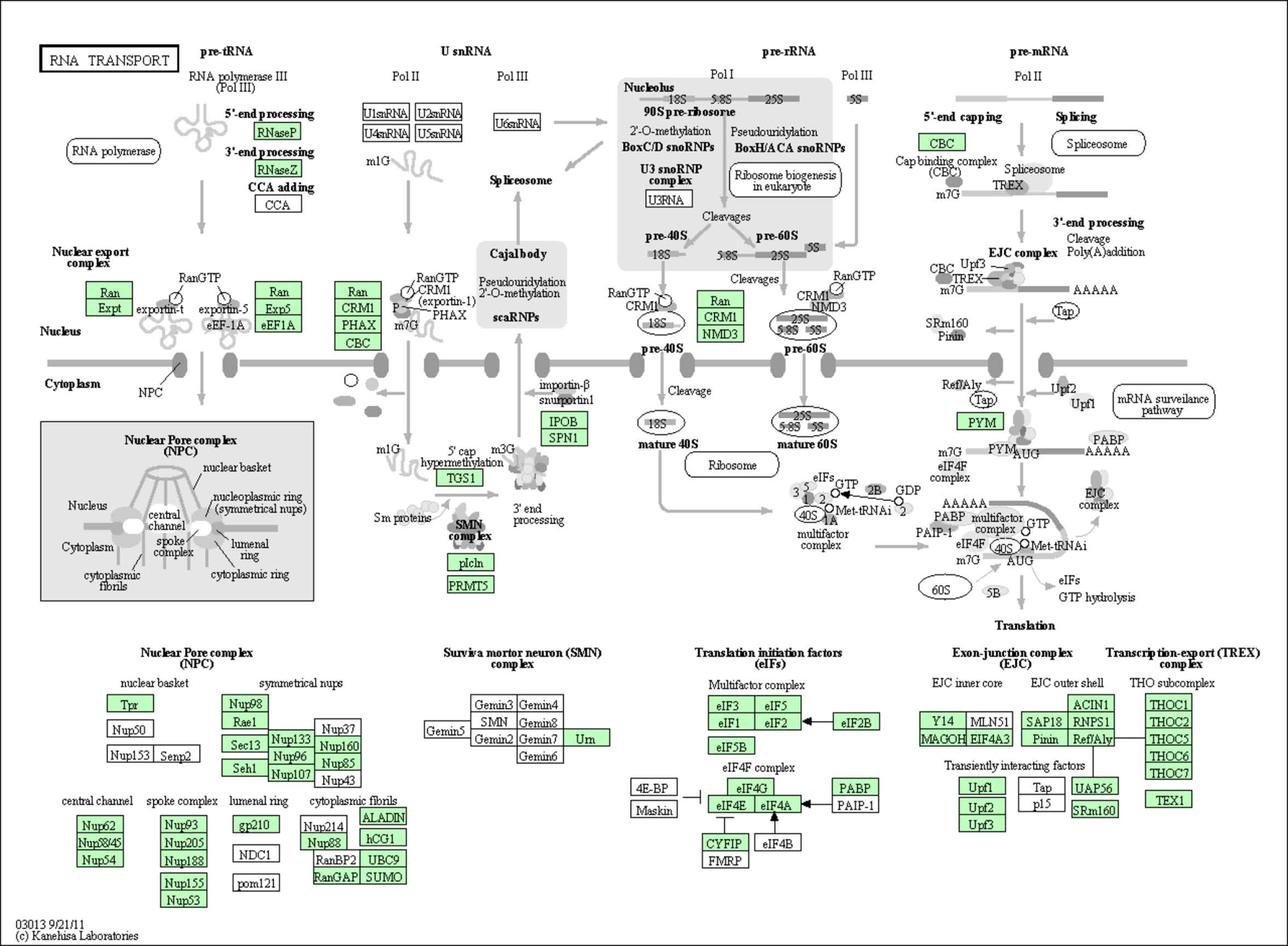
RNA TRANSPORT
pre-tRNA
RNA polymerase III (Pol III)
5'-end processing
RNaseP
3'-end processing
RNaseZ
CCA adding
CCA
RNA polymerase
U snRNA
Pol II
Pol III
U1snRNA
U2snRNA
U4snRNA
U5snRNA
U6snRNA
m1G
Spliceosome
pre-rRNA
Pol I
Pol III
Nucleolus
18S 5.8S 25S
5S
90S pre-ribosome
2'-O-methylation
BoxC/D snoRNPs
Pseudouridylation
BoxH/ACA snoRNPs
U3 snoRNP complex
U3RNA
Ribosome biogenesis in eukaryote
Cleavages
pre-40S
pre-60S
pre-mRNA
Pol II
5'-end capping
Splicing
CBC
Spliceosome
Cap binding complex (CBC)
Spliceosome
TREX
m7G
3'-end processing
Cleavage
Poly(A)addition
EJC complex
CBC
Upf3
TREX
m7G
AAAAA
Nuclear export complex
Ran
Expt
RanGTP
exportin-t
exportin-5
eEF-1A
Ran
Exp5
eEF1A
Ran
CRM1
PHAX
CBC
RanGTP
CRM1 (exportin-1)
P
PHAX
m7G
Cajal body
Pseudouridylation
2'-O-methylation
scaRNPs
RanGTP
CRM1
18S
pre-40S
Ran
CRM1
NMD3
Cleavages
RanGTP
CRM1
NMD3
25S
5.8S 5S
pre-60S
Tap
SRm160
Pinin
Nucleus
Cytoplasm
NPC
importin-β
snurportin1
IPOB
SPN1
Cleavage
18S
mature 40S
25S
5.8S 5S
mature 60S
Ribosome
Ref/Aly
Tap
PYM
Upf2
Upf1
mRNA surveilance pathway
Nuclear Pore complex (NPC)
nuclear basket
nucleoplasmic ring (symmetrical nups)
Nucleus
central channel
spoke complex
lumenal ring
Cytoplasm
cytoplasmic fibrils
cytoplasmic ring
m1G
5' cap hypermethylation
m3G
TGS1
Sm proteins
SMN complex
3' end processing
pIcln
PRMT5
eIFs
3 5
1 2
GTP
2B
GDP
40S
1A
Met-tRNAi
2
multifactor complex
m7G
PYM
AUG
PABP
AAAAA
eIF4F complex
AAAAA
PABP
PAIP-1
multifactor complex
GTP
eIF4F
40S
Met-tRNAi
m7G
AUG
EJC complex
60S
5B
eIFs
GTP hydrolysis
Translation
Nuclear Pore complex (NPC)
nuclear basket
Tpr
Nup50
Nup153
Senp2
symmetrical nups
Nup98
Rae1
Sec13
Seh1
Nup133
Nup96
Nup107
Nup37
Nup160
Nup85
Nup43
central channel
Nup62
Nup58/45
Nup54
spoke complex
Nup93
Nup205
Nup188
Nup155
Nup53
lumenal ring
gp210
NDC1
pom121
cytoplasmic fibrils
ALADIN
Nup214
Nup88
hCG1
RanBP2
UBC9
RanGAP
SUMO
Surviva mortor neuron (SMN) complex
Gemin3
Gemin4
SMN
Gemin8
Gemin5
Gemin2
Gemin7
Um
Gemin6
Translation initiation factors (eIFs)
Multifactor complex
eIF3
eIF5
eIF1
eIF2
eIF2B
eIF5B
eIF4F complex
4E-BP
Maskin
eIF4G
eIF4E
eIF4A
PABP
PAIP-1
CYFIP
FMRP
eIF4B
Exon-junction complex (EJC)
EJC inner core
Y14
MLN51
MAGOH
EIF4A3
EJC outer shell
ACIN1
SAP18
RNPS1
Pinin
Ref/Aly
Transiently interacting factors
Upf1
Upf2
Upf3
Tap
p15
UAP56
SRm160
Transcription-export (TREX) complex
THO subcomplex
THOC1
THOC2
THOC5
THOC6
THOC7
TEX1
03013 9/21/11
(c) Kanehisa Laboratories

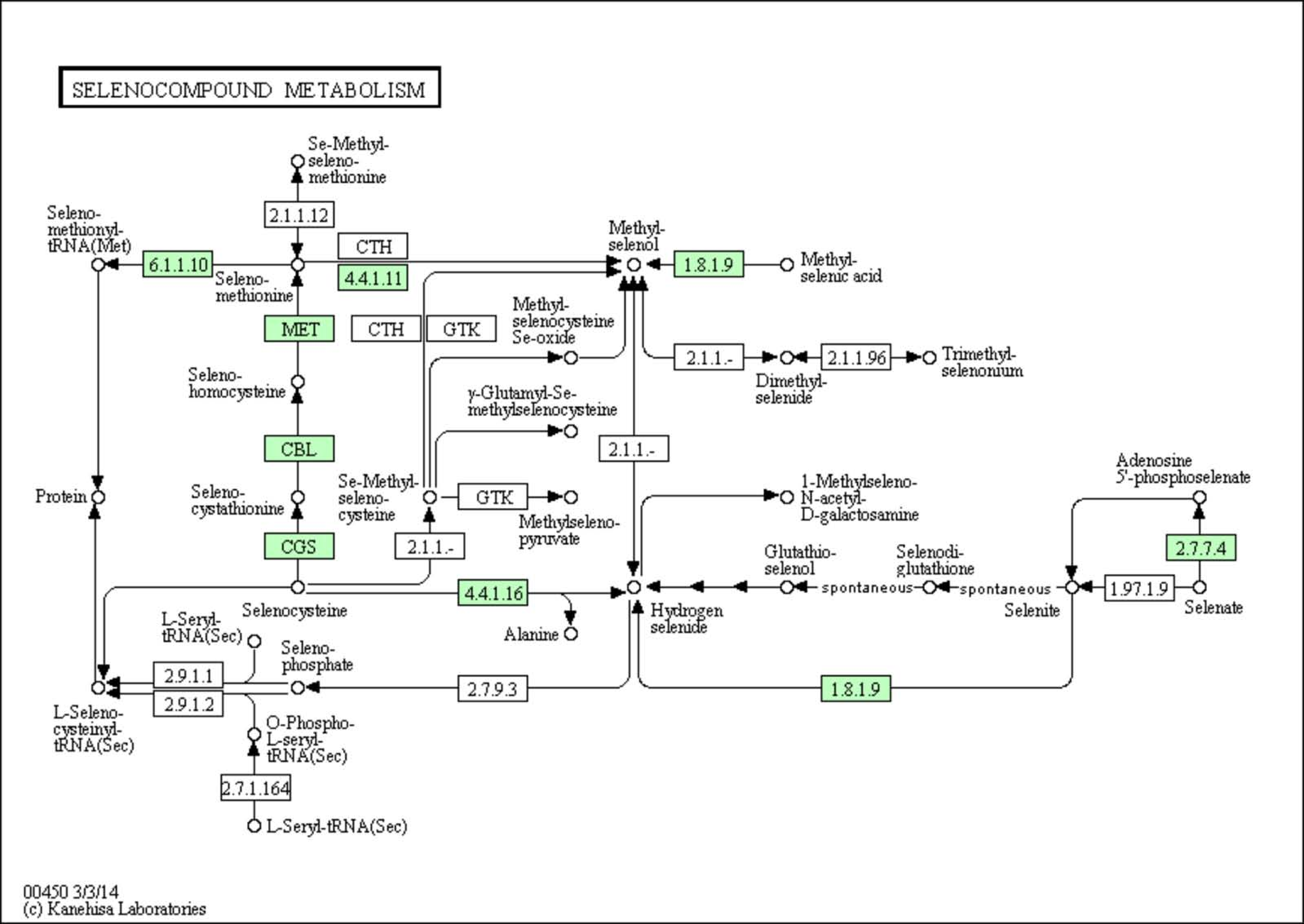
SELENOCOMPOUND METABOLISM
Se-Methyl-selenomethionine
2.1.1.12
Selenomethionyl-tRNA(Met)
6.1.1.10
Selenomethionine
CTH
4.4.1.11
Methylselenol
1.8.1.9
Methylselenic acid
MET
CTH
GTK
Methylselenocysteine Se-oxide
2.1.1.-
2.1.1.96
Trimethylselenonium
Dimethylselenide
Selenohomocysteine
γ-Glutamyl-Se-methylselenocysteine
CBL
2.1.1.-
Adenosine 5'-phosphoselenate
Selenocystathionine
Se-Methylselenocysteine
GTK
1-Methylseleno-N-acetyl-D-galactosamine
Protein
Methylselenopyruvate
CGS
2.1.1.-
2.7.7.4
Glutathioselenol
Selenodiglutathione
spontaneous
spontaneous
1.97.1.9
Selenocysteine
4.4.1.16
Hydrogen selenide
Selenite
Selenate
L-Seryl-tRNA(Sec)
Alanine
Selenophosphate
2.9.1.1
2.9.1.2
2.7.9.3
1.8.1.9
L-Selenocysteinyl-tRNA(Sec)
O-Phospho-L-seryl-tRNA(Sec)
2.7.1.164
L-Seryl-tRNA(Sec)
00450 3/3/14
(c) Kanehisa Laboratories

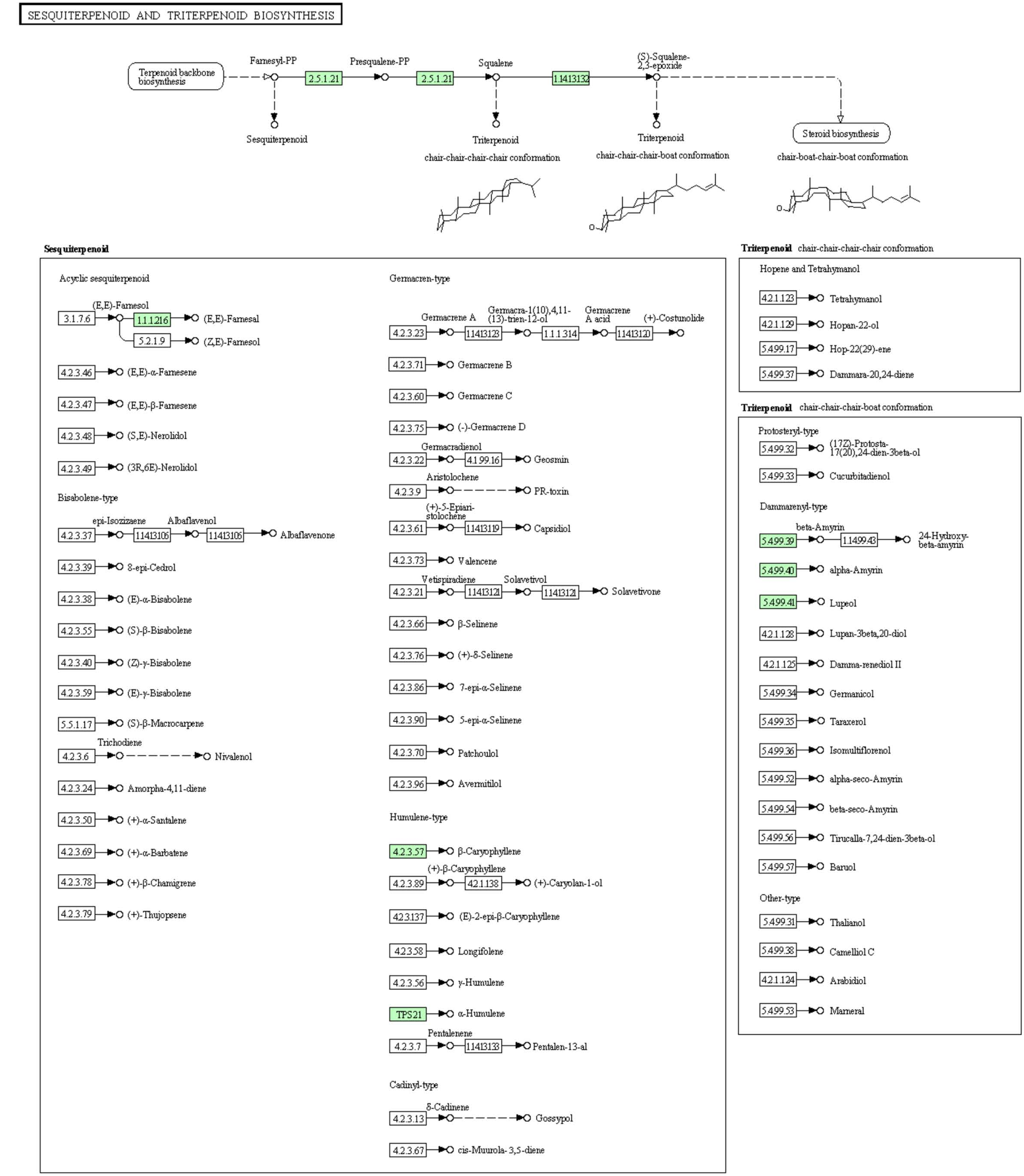

00909 3/13/14
(c) Kanehisa Laboratories

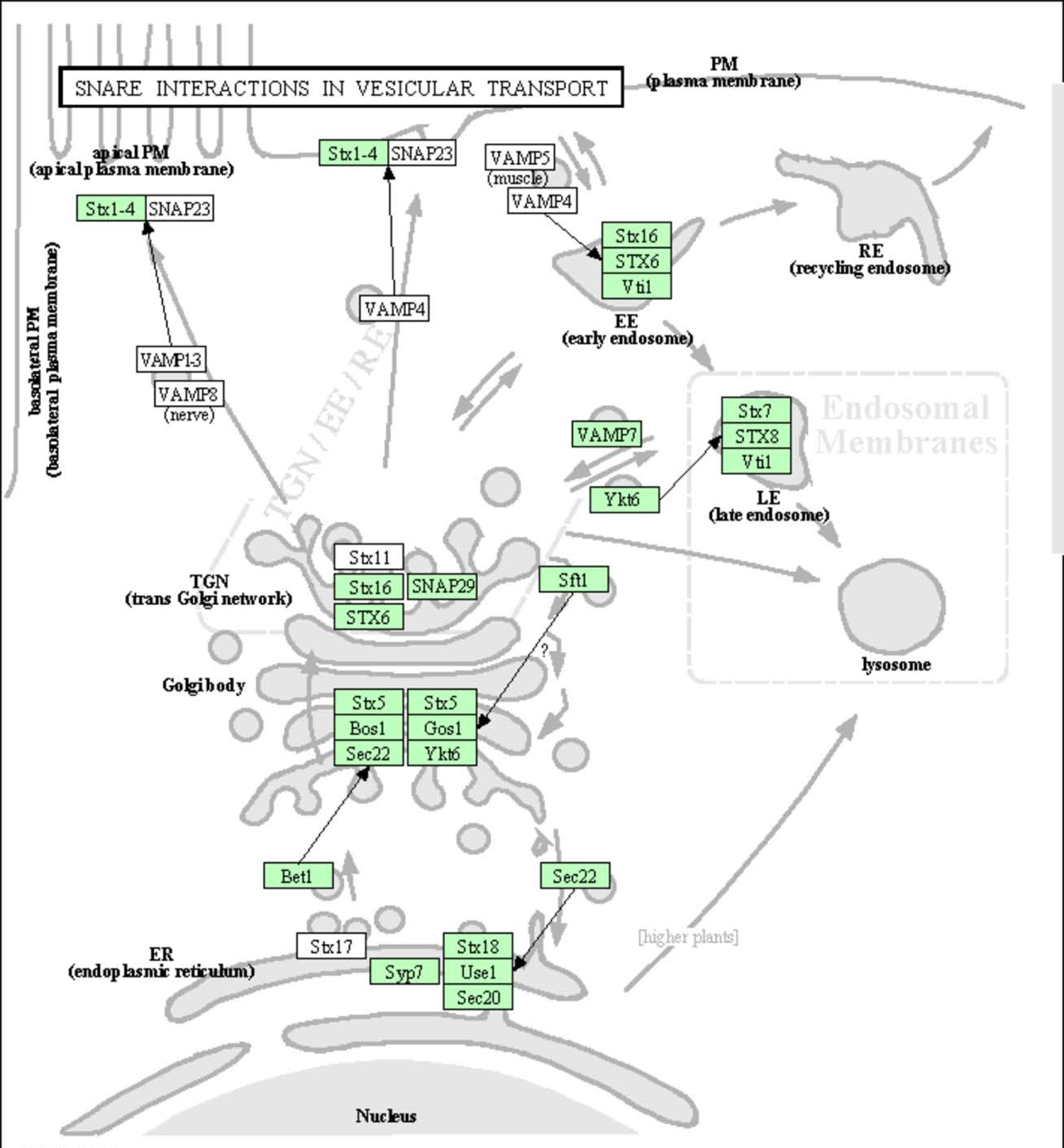

04130 3/4/10
(c) Kanehisa Laboratories

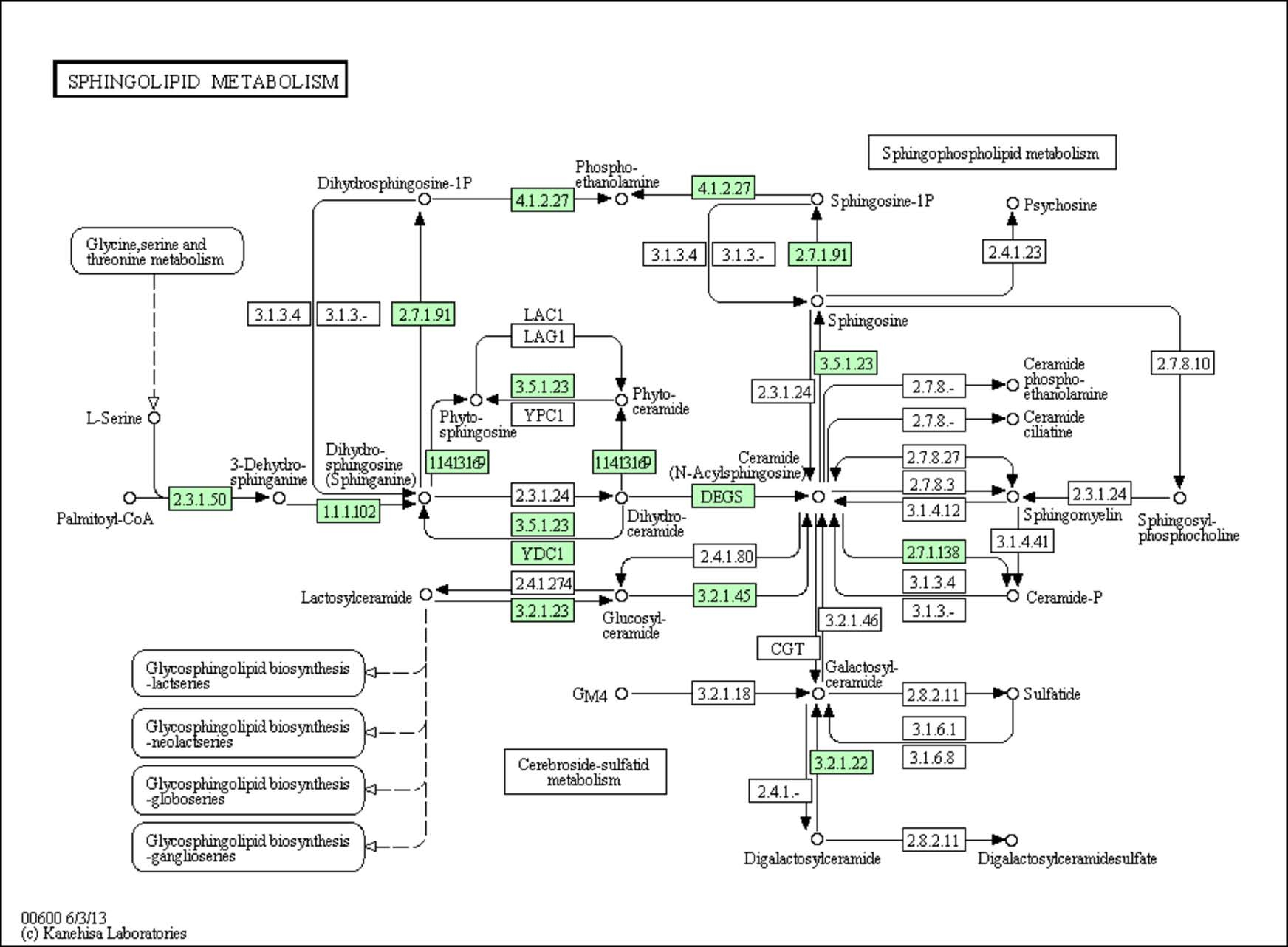
SPHINGOLIPID METABOLISM
Sphingophospholipid metabolism
Glycine,serine and threonine metabolism
Dihydrosphingosine-1P
Phospho-ethanolamine
Sphingosine-1P
Psychosine
4.1.2.27
4.1.2.27
3.1.3.4
3.1.3.-
2.7.1.91
2.4.1.23
3.1.3.4
3.1.3.-
2.7.1.91
Sphingosine
LAC1
LAG1
3.5.1.23
YPC1
Phyto-sphingosine
Phyto-ceramide
3.5.1.23
2.3.1.24
2.7.8.-
Ceramide phospho-ethanolamine
2.7.8.-
Ceramide ciliatine
2.7.8.10
L-Serine
3-Dehydro-sphinganine
Dihydro-sphingosine (Sphinganine)
1141316P
1141316P
Ceramide (N-Acylsphingosine)
2.7.8.27
2.7.8.3
3.1.4.12
Palmitoyl-CoA
2.3.1.50
1.1.1.102
2.3.1.24
Dihydro-ceramide
DEGS
Sphingomyelin
2.3.1.24
Sphingosyl-phosphocholine
3.5.1.23
YDC1
3.1.4.41
2.4.1.80
2.7.1.138
2.4.1.274
3.2.1.45
3.1.3.4
Lactosylceramide
Ceramide-P
3.2.1.23
Glucosyl-ceramide
3.1.3.-
3.2.1.46
CGT
Galactosyl-ceramide
Glycosphingolipid biosynthesis -lactseries
Glycosphingolipid biosynthesis -neolactseries
Glycosphingolipid biosynthesis -globoseries
Glycosphingolipid biosynthesis -ganglioseries
GM4
3.2.1.18
2.8.2.11
Sulfatide
3.1.6.1
3.1.6.8
3.2.1.22
Cerebroside-sulfatid metabolism
2.4.1.-
Digalactosylceramide
2.8.2.11
Digalactosylceramidesulfate
00600 6/3/13
(c) Kanehisa Laboratories

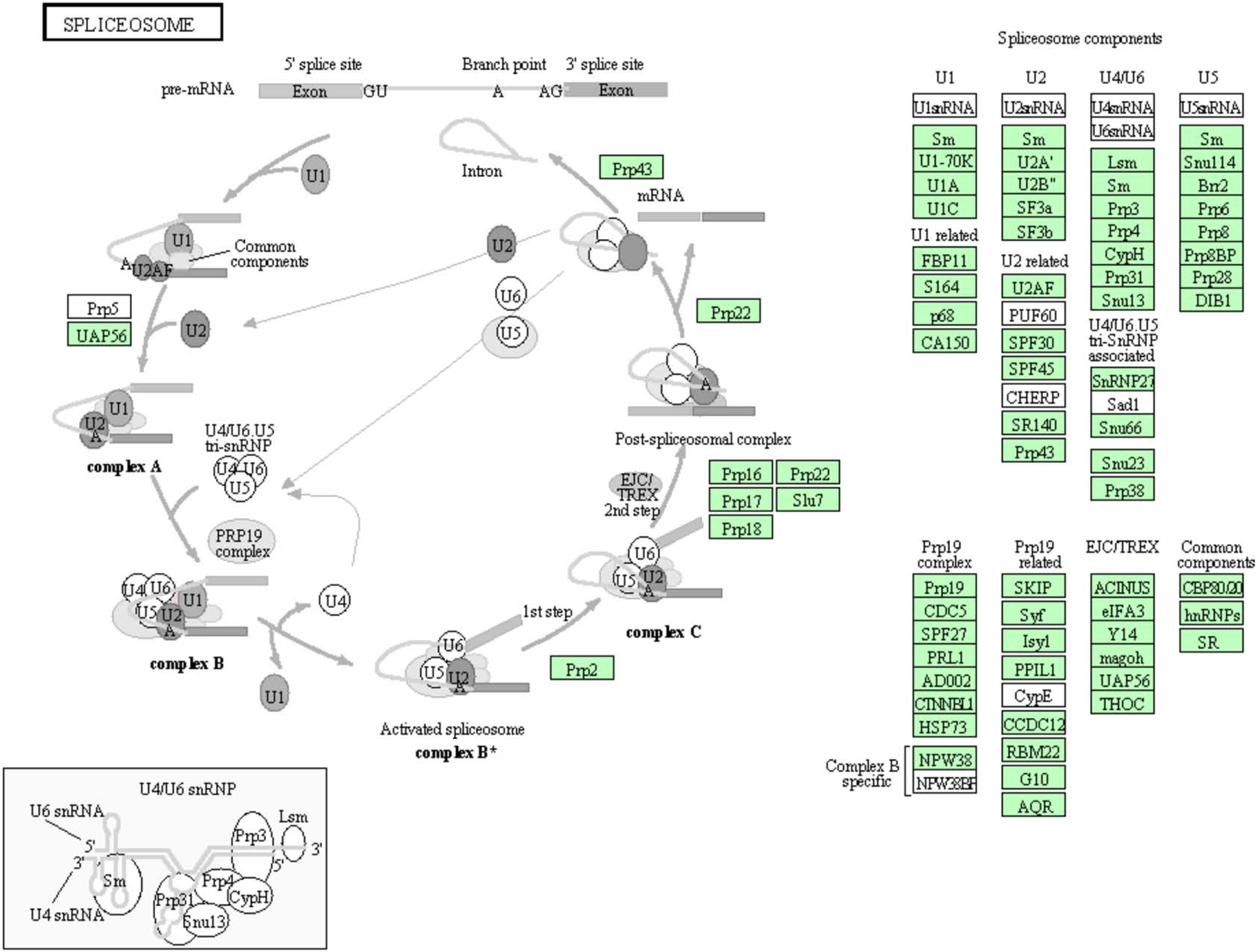

03040 12/6/10
(c) Kanehisa Laboratories

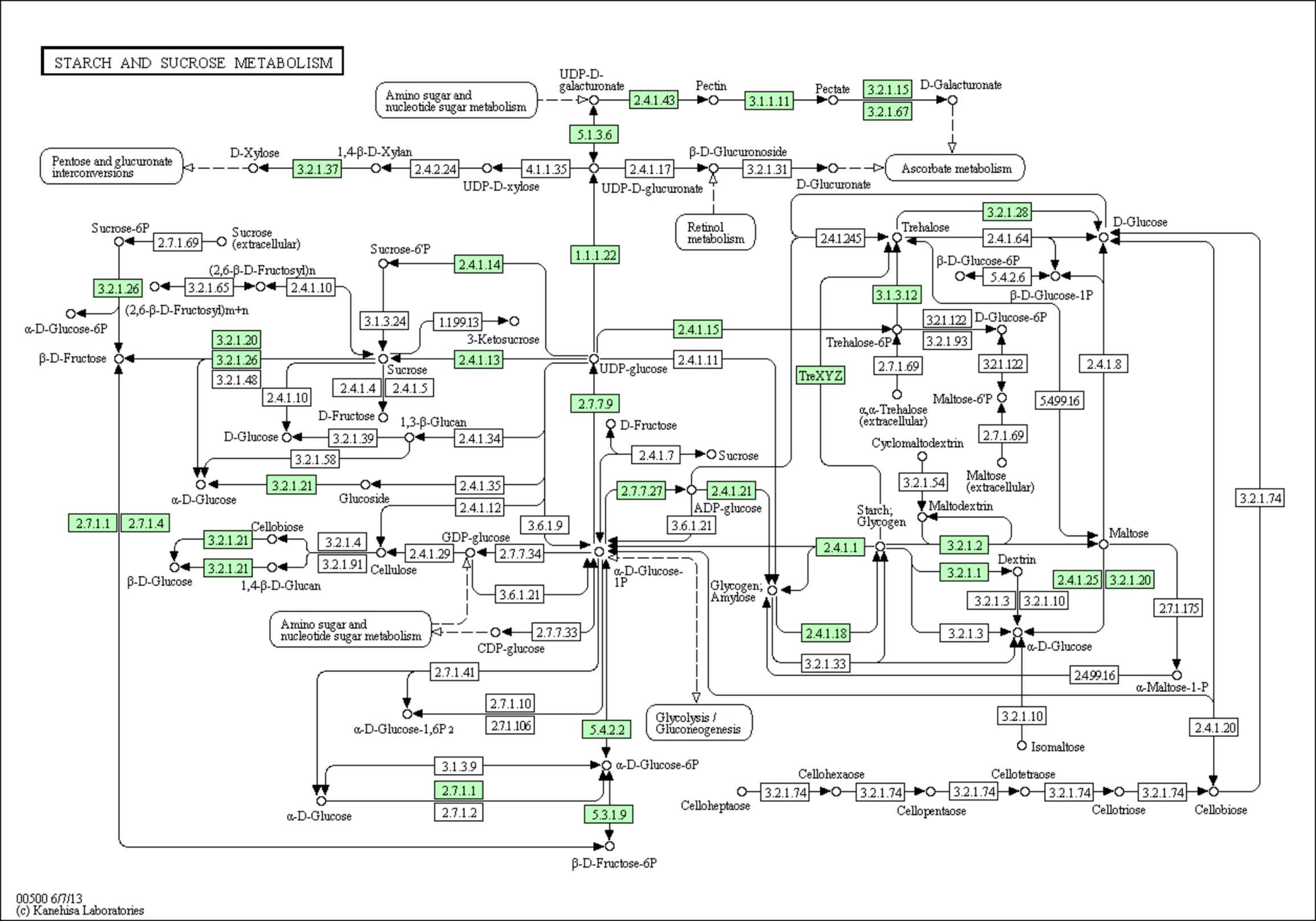
STARCH AND SUCROSE METABOLISM
Amino sugar and nucleotide sugar metabolism
UDP-D-galacturonate
2.4.1.43
Pectin
3.1.1.11
Pectate
3.2.1.15
3.2.1.67
D-Galacturonate
5.1.3.6
Pentose and glucuronate interconversions
D-Xylose
3.2.1.37
1,4-β-D-Xylan
2.4.2.24
UDP-D-xylose
4.1.1.35
UDP-D-glucuronate
2.4.1.17
β-D-Glucuronoside
3.2.1.31
D-Glucuronate
Ascorbate metabolism
Retinol metabolism
1.1.1.22
Sucrose-6P
2.7.1.69
Sucrose (extracellular)
3.2.1.26
(2,6-β-D-Fructosyl)n
3.2.1.65
2.4.1.10
(2,6-β-D-Fructosyl)m+n
α-D-Glucose-6P
Sucrose-6'P
2.4.1.14
3.1.3.24
1.1.99.13
3-Ketosucrose
β-D-Fructose
3.2.1.20
3.2.1.26
3.2.1.48
Sucrose
2.4.1.13
UDP-glucose
2.4.1.15
2.4.1.11
2.4.1.4
2.4.1.5
2.4.1.10
D-Fructose
1,3-β-Glucan
2.4.1.34
D-Glucose
3.2.1.39
3.2.1.58
2.7.7.9
α-D-Glucose
3.2.1.21
Glucoside
2.4.1.35
2.4.1.12
2.7.1.1
2.7.1.4
Cellobiose
3.2.1.21
3.2.1.4
3.2.1.91
3.2.1.21
β-D-Glucose
1,4-β-D-Glucan
Cellulose
2.4.1.29
GDP-glucose
2.7.7.34
3.6.1.9
3.6.1.21
D-Fructose
2.4.1.7
Sucrose
2.7.7.27
2.4.1.21
ADP-glucose
3.6.1.21
α-D-Glucose-1P
Amino sugar and nucleotide sugar metabolism
2.7.7.33
CDP-glucose
Glycolysis / Gluconeogenesis
2.7.1.41
2.7.1.10
2.7.1.106
α-D-Glucose-1,6P 2
5.4.2.2
3.1.3.9
α-D-Glucose-6P
2.7.1.1
2.7.1.2
α-D-Glucose
5.3.1.9
β-D-Fructose-6P
D-Glucose
3.2.1.28
Trehalose
2.4.1.245
2.4.1.64
β-D-Glucose-6P
5.4.2.6
β-D-Glucose-1P
3.1.3.12
3.2.1.122
3.2.1.93
D-Glucose-6P
Trehalose-6P
2.7.1.69
TreXYZ
α,α-Trehalose (extracellular)
3.2.1.122
Maltose-6'P
2.7.1.69
Maltose (extracellular)
2.4.1.8
5.4.99.16
Cyclomaltodextrin
3.2.1.54
Maltodextrin
Starch; Glycogen
3.2.1.2
Maltose
3.2.1.74
2.4.1.1
Glycogen; Amylose
3.2.1.1
Dextrin
2.4.1.25
3.2.1.20
3.2.1.3
3.2.1.10
2.7.1.175
2.4.1.18
3.2.1.3
α-D-Glucose
3.2.1.33
2.4.99.16
α-Maltose-1-P
3.2.1.10
Isomaltose
2.4.1.20
Celloheptaose
3.2.1.74
Cellohexaose
3.2.1.74
Cellopentaose
3.2.1.74
Cellotetraose
3.2.1.74
Cellotriose
3.2.1.74
Cellobiose
00500 6/7/13
(c) Kanehisa Laboratories

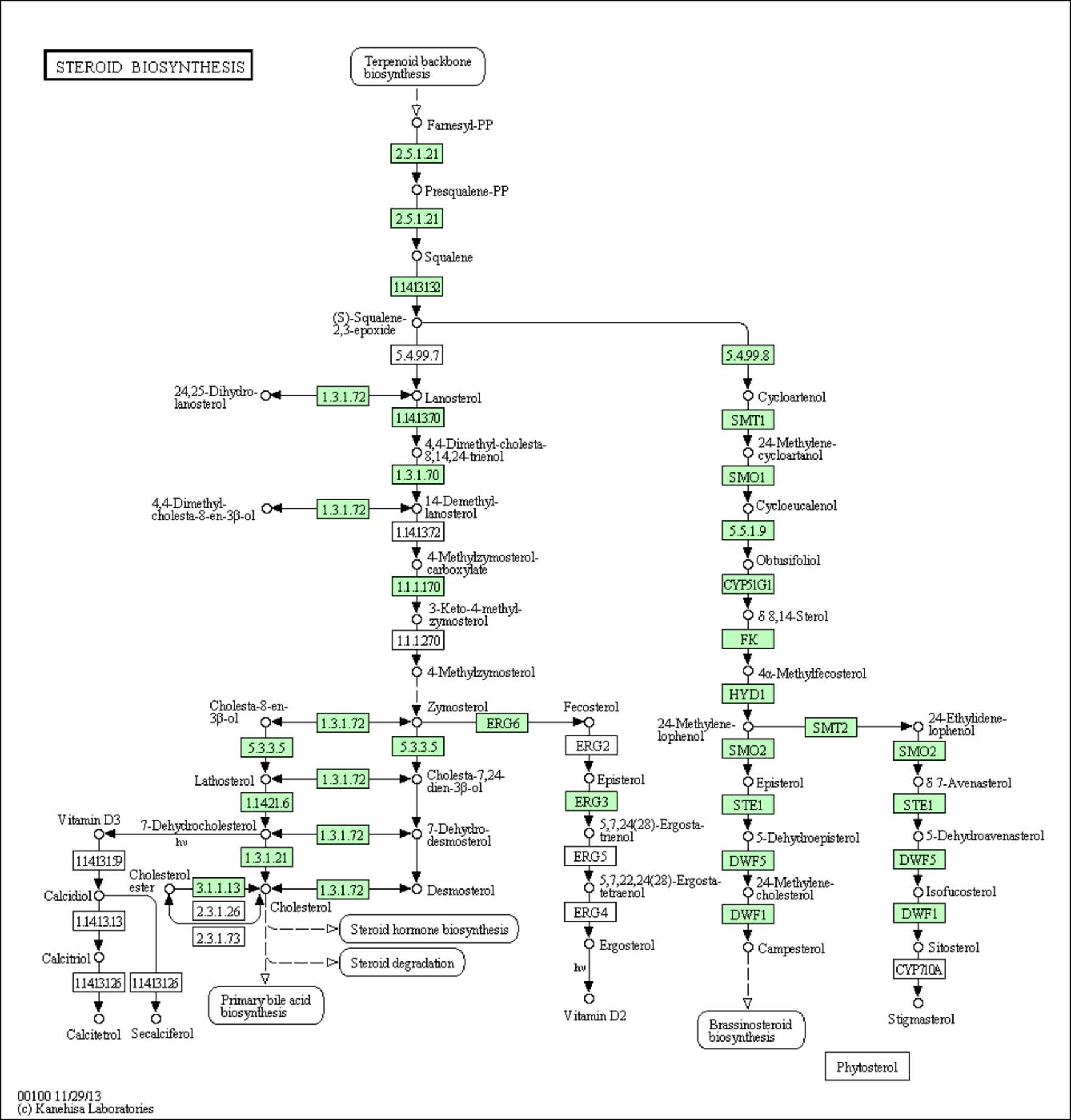

STEROID BIOSYNTHESIS
Terpenoid backbone biosynthesis
Farnesyl-PP
2.5.1.21
Presqualene-PP
2.5.1.21
Squalene
1.14.13.132
(S)-Squalene-2,3-epoxide
5.4.99.7
5.4.99.8
24,25-Dihydro-lanosterol
1.3.1.72
Lanosterol
1.14.13.70
4,4-Dimethyl-cholesta-8,14,24-trienol
1.3.1.70
4,4-Dimethyl-cholesta-8-en-3β-ol
1.3.1.72
14-Demethyl-lanosterol
1.14.13.72
4-Methylzymosterol-carboxylate
1.1.1.170
3-Keto-4-methyl-zymosterol
1.1.1.270
4-Methylzymosterol
Cholesta-8-en-3β-ol
1.3.1.72
Zymosterol
ERG6
Fecosterol
5.3.3.5
5.3.3.5
ERG2
Lathosterol
1.3.1.72
Cholesta-7,24-dien-3β-ol
Episterol
1.14.21.6
ERG3
Vitamin D3
7-Dehydrocholesterol
hν
1.3.1.72
7-Dehydro-desmosterol
5,7,24(28)-Ergosta-trienol
1.14.13.159
1.3.1.21
ERG5
Cholesterol ester
3.1.1.13
1.3.1.72
Desmosterol
Calcidiol
5,7,22,24(28)-Ergosta-tetraenol
2.3.1.26
Cholesterol
ERG4
1.14.13.13
Steroid hormone biosynthesis
2.3.1.73
Calcitriol
Steroid degradation
Ergosterol
hν
1.14.13.126
1.14.13.126
Primary bile acid biosynthesis
Vitamin D2
Calcitetrol
Secalciferol
Cycloartenol
SMT1
24-Methylene-cycloartanol
SMO1
Cycloeucalenol
5.5.1.9
Obtusifoliol
CYP51G1
δ 8,14-Sterol
FK
4α-Methylfecosterol
HYD1
24-Methylene-lophenol
SMT2
24-Ethylidene-lophenol
SMO2
SMO2
Episterol
δ 7-Avenasterol
STE1
STE1
5-Dehydroepisterol
5-Dehydroavenasterol
DWF5
DWF5
24-Methylene-cholesterol
Isofucosterol
DWF1
DWF1
Campesterol
Sitosterol
CYP710A
Brassinosteroid biosynthesis
Stigmasterol
Phytosterol
00100 11/29/13
(c) Kanehisa Laboratories

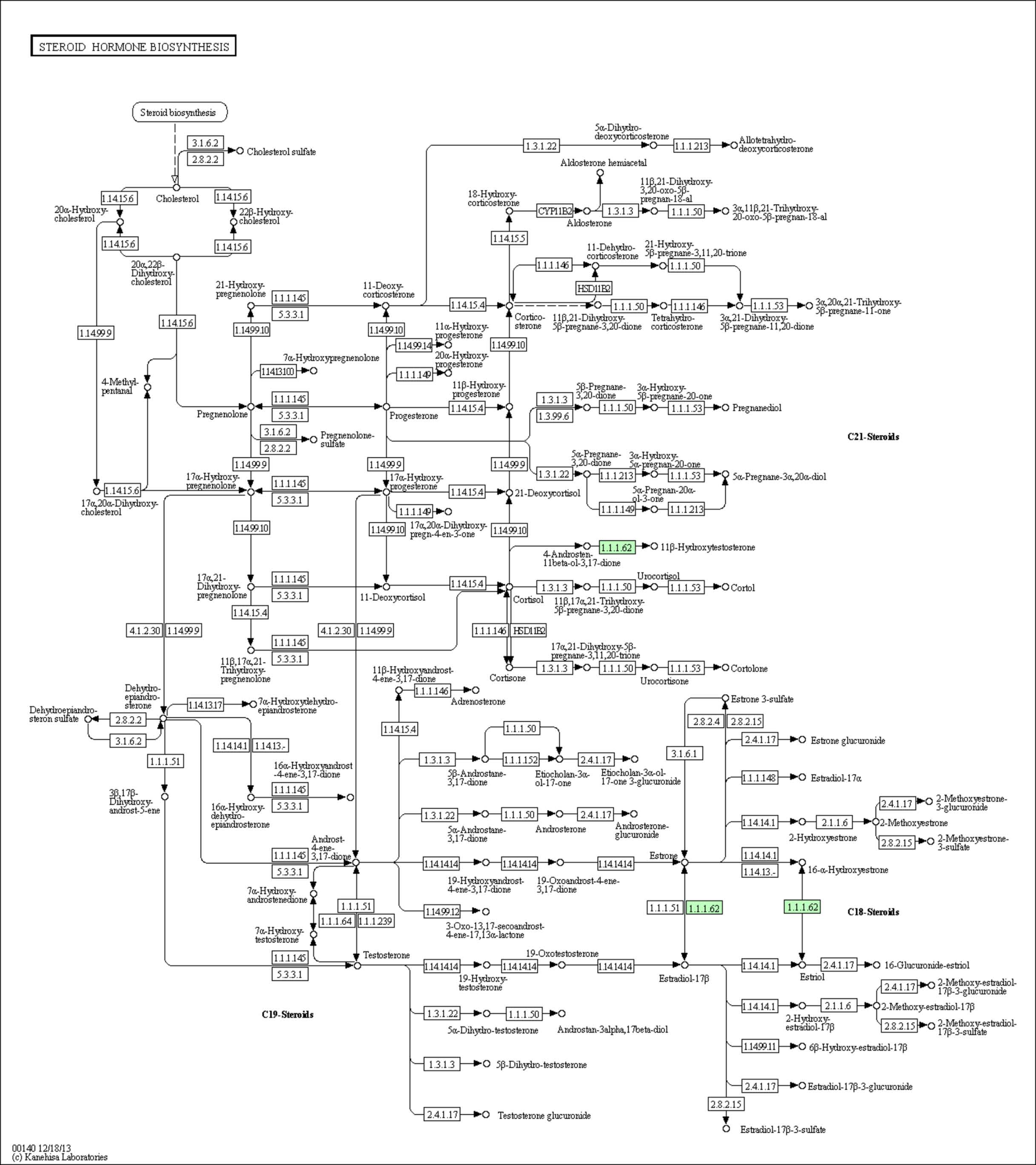

STEROID HORMONE BIOSYNTHESIS
Steroid biosynthesis
3.1.6.2
2.8.2.2
Cholesterol sulfate
1.14.15.6
Cholesterol
1.14.15.6
20α-Hydroxy-cholesterol
22β-Hydroxy-cholesterol
1.14.15.6
1.14.15.6
20α,22β-Dihydroxy-cholesterol
1.14.99.9
1.14.15.6
4-Methyl-pentanal
21-Hydroxy-pregnenolone
1.1.1.145
5.3.3.1
11-Deoxy-corticosterone
1.3.1.22
5α-Dihydro-deoxycorticosterone
1.1.1.213
Allotetrahydro-deoxycorticosterone
Aldosterone hemiacetal
18-Hydroxy-corticosterone
CYP11B2
Aldosterone
1.3.1.3
11β,21-Dihydroxy-3,20-oxo-5β-pregnan-18-al
1.1.1.50
3α,11β,21-Trihydroxy-20-oxo-5β-pregnan-18-al
1.14.15.5
1.1.1.146
11-Dehydro-corticosterone
21-Hydroxy-5β-pregnane-3,11,20-trione
1.1.1.50
HSD11B2
1.14.15.4
Cortico-sterone
1.1.1.50
1.1.1.146
1.1.1.53
3α,20α,21-Trihydroxy-5β-pregnane-11-one
11β,21-Dihydroxy-5β-pregnane-3,20-dione
Tetrahydro-corticosterone
3α,21-Dihydroxy-5β-pregnane-11,20-dione
1.14.99.10
1.14.99.10
11α-Hydroxy-progesterone
1.14.99.14
1.14.99.10
7α-Hydroxypregnenolone
1.14.13.100
20α-Hydroxy-progesterone
1.1.1.149
11β-Hydroxy-progesterone
1.1.1.145
5.3.3.1
Pregnenolone
Progesterone
1.14.15.4
1.3.1.3
1.3.99.6
5β-Pregnane-3,20-dione
1.1.1.50
3α-Hydroxy-5β-pregnan-20-one
1.1.1.53
Pregnanediol
C21-Steroids
3.1.6.2
2.8.2.2
Pregnenolone-sulfate
1.14.99.9
1.14.99.9
1.14.99.9
1.3.1.22
5α-Pregnane-3,20-dione
1.1.1.213
3α-Hydroxy-5α-pregnan-20-one
1.1.1.53
5α-Pregnane-3α,20α-diol
17α-Hydroxy-pregnenolone
1.1.1.145
5.3.3.1
17α-Hydroxy-progesterone
1.14.15.4
21-Deoxycortisol
5α-Pregnan-20α-ol-3-one
1.1.1.149
1.1.1.213
1.14.15.6
17α,20α-Dihydroxy-cholesterol
1.1.1.149
17α,20α-Dihydroxy-pregn-4-en-3-one
1.14.99.10
1.14.99.10
1.14.99.10
1.1.1.62
4-Androsten-11beta-ol-3,17-dione
11β-Hydroxytestosterone
17α,21-Dihydroxy-pregnenolone
1.1.1.145
5.3.3.1
11-Deoxycortisol
1.14.15.4
Cortisol
1.3.1.3
1.1.1.50
Urocortisol
1.1.1.53
Cortol
11β,17α,21-Trihydroxy-5β-pregnane-3,20-dione
1.14.15.4
4.1.2.30
1.14.99.9
4.1.2.30
1.14.99.9
1.1.1.146
HSD11B2
1.1.1.145
5.3.3.1
11β,17α,21-Trihydroxy-pregnenolone
17α,21-Dihydroxy-5β-pregnane-3,11,20-trione
Cortisone
1.3.1.3
1.1.1.50
1.1.1.53
Cortolone
Urocortisone
11β-Hydroxyandrost-4-ene-3,17-dione
1.1.1.146
Adrenosterone
Dehydro-epiandro-sterone
1.14.13.17
7α-Hydroxydehydro-epiandrosterone
Dehydroepiandro-steron sulfate
2.8.2.2
3.1.6.2
1.14.15.4
1.1.1.50
Estrone 3-sulfate
2.8.2.4
2.8.2.15
2.4.1.17
Estrone glucuronide
1.1.1.51
1.14.14.1
1.14.13.-
16α-Hydroxyandrost-4-ene-3,17-dione
1.3.1.3
1.1.1.152
2.4.1.17
5β-Androstane-3,17-dione
Etiocholan-3α-ol-17-one
Etiocholan-3α-ol-17-one 3-glucuronide
3.1.6.1
1.1.1.148
Estradiol-17α
3β,17β-Dihydroxy-androst-5-ene
1.1.1.145
5.3.3.1
16α-Hydroxy-dehydro-epiandrosterone
1.3.1.22
1.1.1.50
2.4.1.17
5α-Androstane-3,17-dione
Androsterone
Androsterone-glucuronide
2.4.1.17
2-Methoxyestrone-3-glucuronide
1.14.14.1
2.1.1.6
2-Methoxyestrone
2-Hydroxyestrone
2.8.2.15
2-Methoxyestrone-3-sulfate
Androst-4-ene-3,17-dione
1.1.1.145
5.3.3.1
1.14.14.14
1.14.14.14
1.14.14.14
Estrone
1.14.14.1
1.14.13.-
16-α-Hydroxyestrone
19-Hydroxyandrost-4-ene-3,17-dione
19-Oxoandrost-4-ene-3,17-dione
7α-Hydroxy-androstenedione
1.1.1.51
1.14.99.12
3-Oxo-13,17-secoandrost-4-ene-17,13α-lactone
1.1.1.51
1.1.1.62
1.1.1.62
C18-Steroids
1.1.1.64
1.1.1.239
7α-Hydroxy-testosterone
1.1.1.145
5.3.3.1
Testosterone
1.14.14.14
19-Hydroxy-testosterone
1.14.14.14
19-Oxotestosterone
1.14.14.14
Estradiol-17β
1.14.14.1
Estriol
2.4.1.17
16-Glucuronide-estriol
2.4.1.17
2-Methoxy-estradiol-17β-3-glucuronide
1.14.14.1
2.1.1.6
2-Methoxy-estradiol-17β
2-Hydroxy-estradiol-17β
2.8.2.15
2-Methoxy-estradiol-17β-3-sulfate
C19-Steroids
1.3.1.22
1.1.1.50
5α-Dihydro-testosterone
Androstan-3alpha,17beta-diol
1.14.99.11
6β-Hydroxy-estradiol-17β
1.3.1.3
5β-Dihydro-testosterone
2.4.1.17
Estradiol-17β-3-glucuronide
2.8.2.15
2.4.1.17
Testosterone glucuronide
Estradiol-17β-3-sulfate
00140 12/18/13
(c) Kanehisa Laboratories

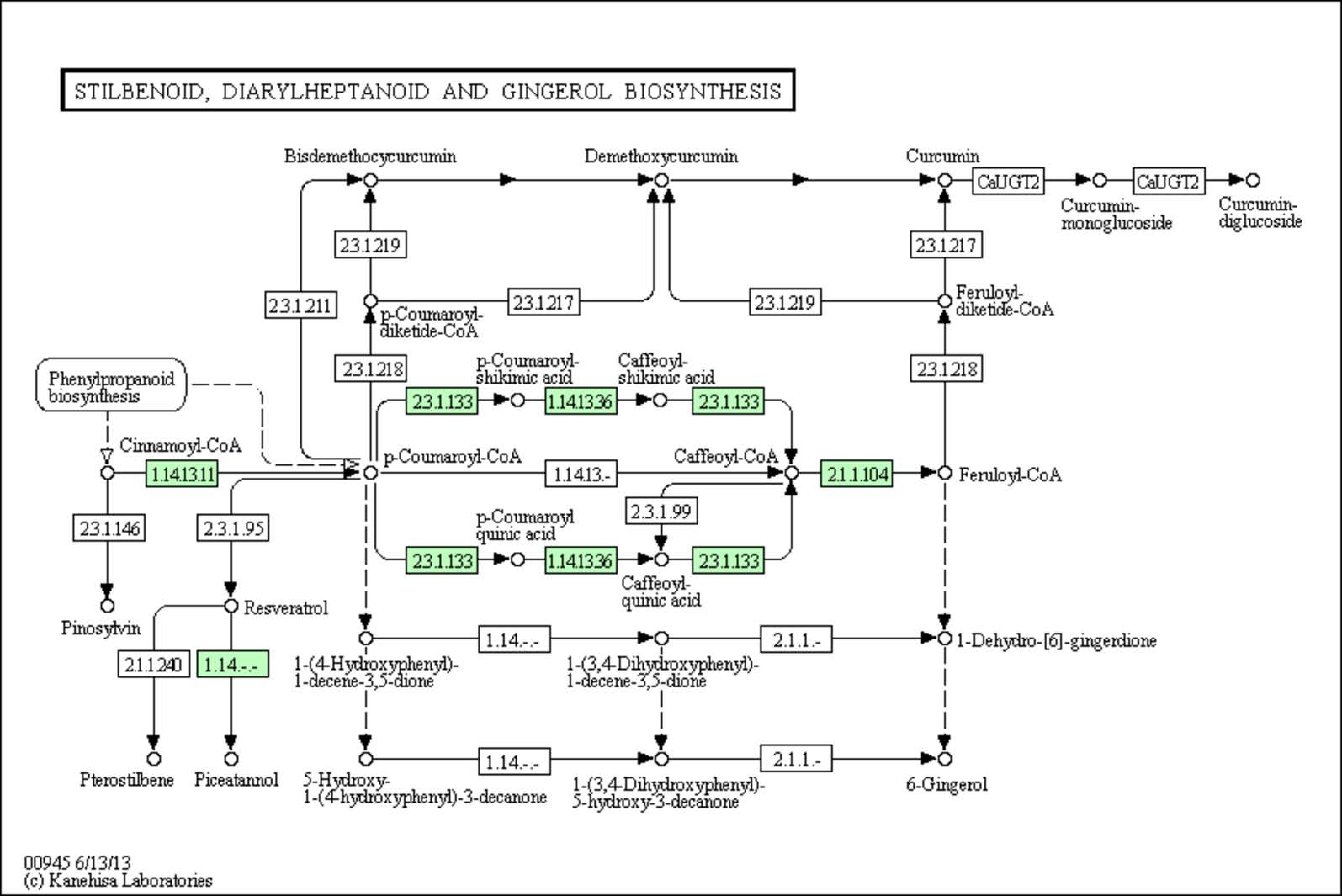
STILBENOID, DIARYLHEPTANOID AND GINGEROL BIOSYNTHESIS
Bisdemethocycurcumin
Demethoxycurcumin
Curcumin
CaUGT2
CaUGT2
Curcumin-monoglucoside
Curcumin-diglucoside
2.3.1.219
2.3.1.217
2.3.1.211
2.3.1.217
2.3.1.219
p-Coumaroyl-diketide-CoA
Feruloyl-diketide-CoA
2.3.1.218
2.3.1.218
Phenylpropanoid biosynthesis
p-Coumaroyl-shikimic acid
Caffeoyl-shikimic acid
2.3.1.133
1.14.13.36
2.3.1.133
Cinnamoyl-CoA
1.14.13.11
p-Coumaroyl-CoA
1.14.13.-
Caffeoyl-CoA
2.1.1.104
Feruloyl-CoA
2.3.1.146
2.3.1.95
2.3.1.99
p-Coumaroyl quinic acid
2.3.1.133
1.14.13.36
2.3.1.133
Caffeoyl-quinic acid
Pinosylvin
Resveratrol
2.1.1.240
1.14.-.-
1.14.-.-
2.1.1.-
1-Dehydro-[6]-gingerdione
1-(4-Hydroxyphenyl)-1-decene-3,5-dione
1-(3,4-Dihydroxyphenyl)-1-decene-3,5-dione
1.14.-.-
2.1.1.-
Pterostilbene
Piceatannol
5-Hydroxy-1-(4-hydroxyphenyl)-3-decanone
1-(3,4-Dihydroxyphenyl)-5-hydroxy-3-decanone
6-Gingerol
00945 6/13/13
(c) Kanehisa Laboratories

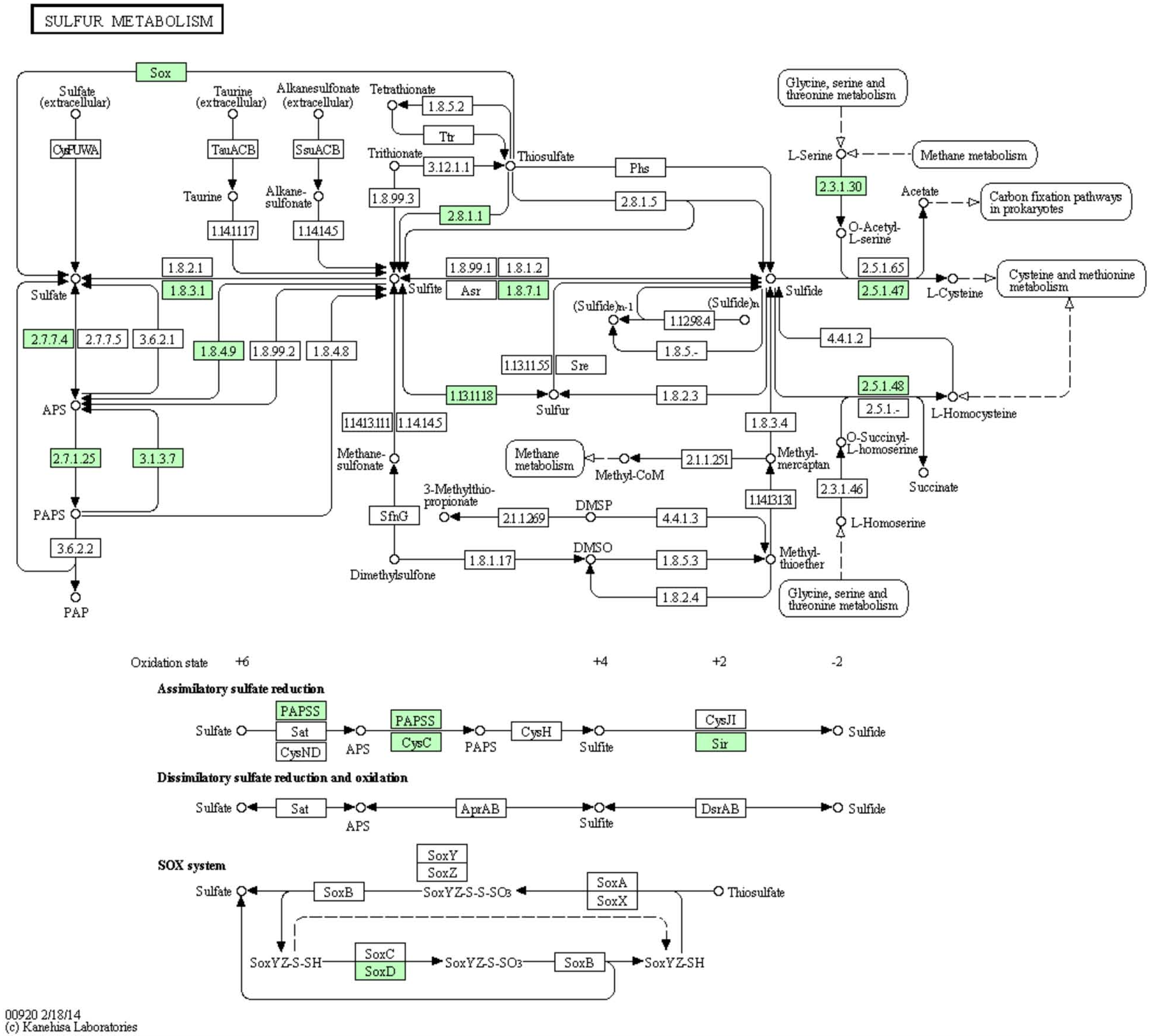
SULFUR METABOLISM
Sox
Sulfate (extracellular)
Taurine (extracellular)
Alkanesulfonate (extracellular)
Tetrathionate
CysPUWA
TauACB
SsuACB
1.8.5.2
Ttr
Trithionate
3.12.1.1
Thiosulfate
Phs
2.8.1.5
Taurine
Alkane-sulfonate
1.8.99.3
2.8.1.1
1.14.11.17
1.14.14.5
1.8.2.1
1.8.3.1
1.8.99.1
1.8.1.2
Asr
1.8.7.1
Sulfate
Sulfite
Sulfide
2.7.7.4
2.7.7.5
3.6.2.1
1.8.4.9
1.8.99.2
1.8.4.8
(Sulfide)n-1
(Sulfide)n
1.12.98.4
1.8.5.-
1.13.11.55
Sre
APS
1.13.11.18
Sulfur
1.8.2.3
1.14.13.111
1.14.14.5
2.7.1.25
3.1.3.7
Methane-sulfonate
Methane metabolism
Methyl-CoM
2.1.1.251
1.8.3.4
Methyl-mercaptan
3-Methylthio-propionate
DMSP
2.1.1.269
4.4.1.3
1.14.13.131
SfnG
PAPS
3.6.2.2
Dimethylsulfone
1.8.1.17
DMSO
1.8.5.3
Methyl-thioether
1.8.2.4
PAP
Glycine, serine and threonine metabolism
L-Serine
Methane metabolism
2.3.1.30
Acetate
Carbon fixation pathways in prokaryotes
O-Acetyl-L-serine
2.5.1.65
2.5.1.47
L-Cysteine
Cysteine and methionine metabolism
4.4.1.2
2.5.1.48
2.5.1.-
L-Homocysteine
O-Succinyl-L-homoserine
Succinate
2.3.1.46
L-Homoserine
Glycine, serine and threonine metabolism
Oxidation state +6 +4 +2 -2
Assimilatory sulfate reduction
Sulfate
PAPSS
Sat
CysND
APS
PAPSS
CysC
PAPS
CysH
Sulfite
CysJI
Sir
Sulfide
Dissimilatory sulfate reduction and oxidation
Sulfate
Sat
APS
AprAB
Sulfite
DsrAB
Sulfide
SOX system
SoxY
SoxZ
Sulfate
SoxB
SoxYZ-S-S-SO3
SoxA
SoxX
Thiosulfate
SoxYZ-S-SH
SoxC
SoxD
SoxYZ-S-SO3
SoxB
SoxYZ-SH
00920 2/18/14
(c) Kanehisa Laboratories

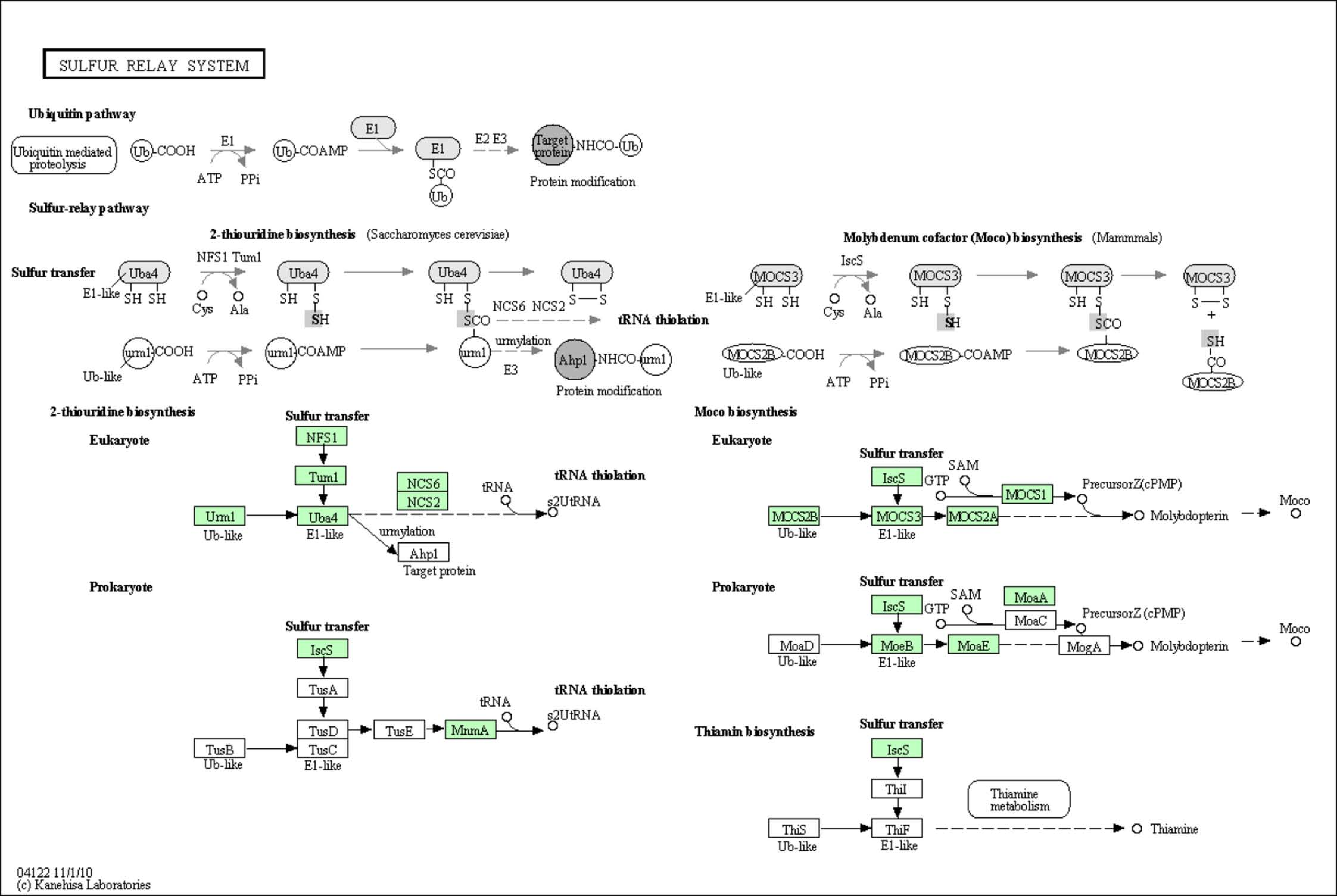
SULFUR RELAY SYSTEM
Ubiquitin pathway
Ubiquitin mediated proteolysis
Ub-COOH
E1
ATP PPi
Ub-COAMP
E1
E1
SCO
Ub
E2 E3
Target protein-NHCO-Ub
Protein modification
Sulfur-relay pathway
2-thiouridine biosynthesis (Saccharomyces cerevisiae)
Sulfur transfer
Uba4
E1-like SH SH
NFS1 Tum1
Cys Ala
Uba4
SH S
SH
Uba4
SH S
SCO
NCS6 NCS2
Uba4
S—S
tRNA thiolation
urm1-COOH
Ub-like
ATP PPi
urm1-COAMP
urm1
urmylation
E3
Ahp1-NHCO-urm1
Protein modification
Molybdenum cofactor (Moco) biosynthesis (Mammals)
MOCS3
E1-like SH SH
IscS
Cys Ala
MOCS3
SH S
SH
MOCS3
SH S
SCO
MOCS3
S—S
+
SH
CO
MOCS2B
MOCS2B-COOH
Ub-like
ATP PPi
MOCS2B-COAMP
MOCS2B
2-thiouridine biosynthesis
Eukaryote
Sulfur transfer
NFS1
Tum1
NCS6
NCS2
Urm1
Ub-like
Uba4
E1-like
tRNA
s2UtRNA
tRNA thiolation
urmylation
Ahp1
Target protein
Prokaryote
Sulfur transfer
IscS
TusA
TusD
TusC
E1-like
TusB
Ub-like
TusE
MnmA
tRNA
s2UtRNA
tRNA thiolation
Moco biosynthesis
Eukaryote
Sulfur transfer
IscS
SAM
GTP
MOCS1
PrecursorZ(cPMP)
MOCS2B
Ub-like
MOCS3
E1-like
MOCS2A
Molybdopterin
Moco
Prokaryote
Sulfur transfer
IscS
SAM
GTP
MoaA
MoaC
PrecursorZ (cPMP)
MoaD
Ub-like
MoeB
E1-like
MoaE
MogA
Molybdopterin
Moco
Thiamin biosynthesis
Sulfur transfer
IscS
ThiI
Thiamine metabolism
ThiS
Ub-like
ThiF
E1-like
Thiamine
04122 11/1/10
(c) Kanehisa Laboratories

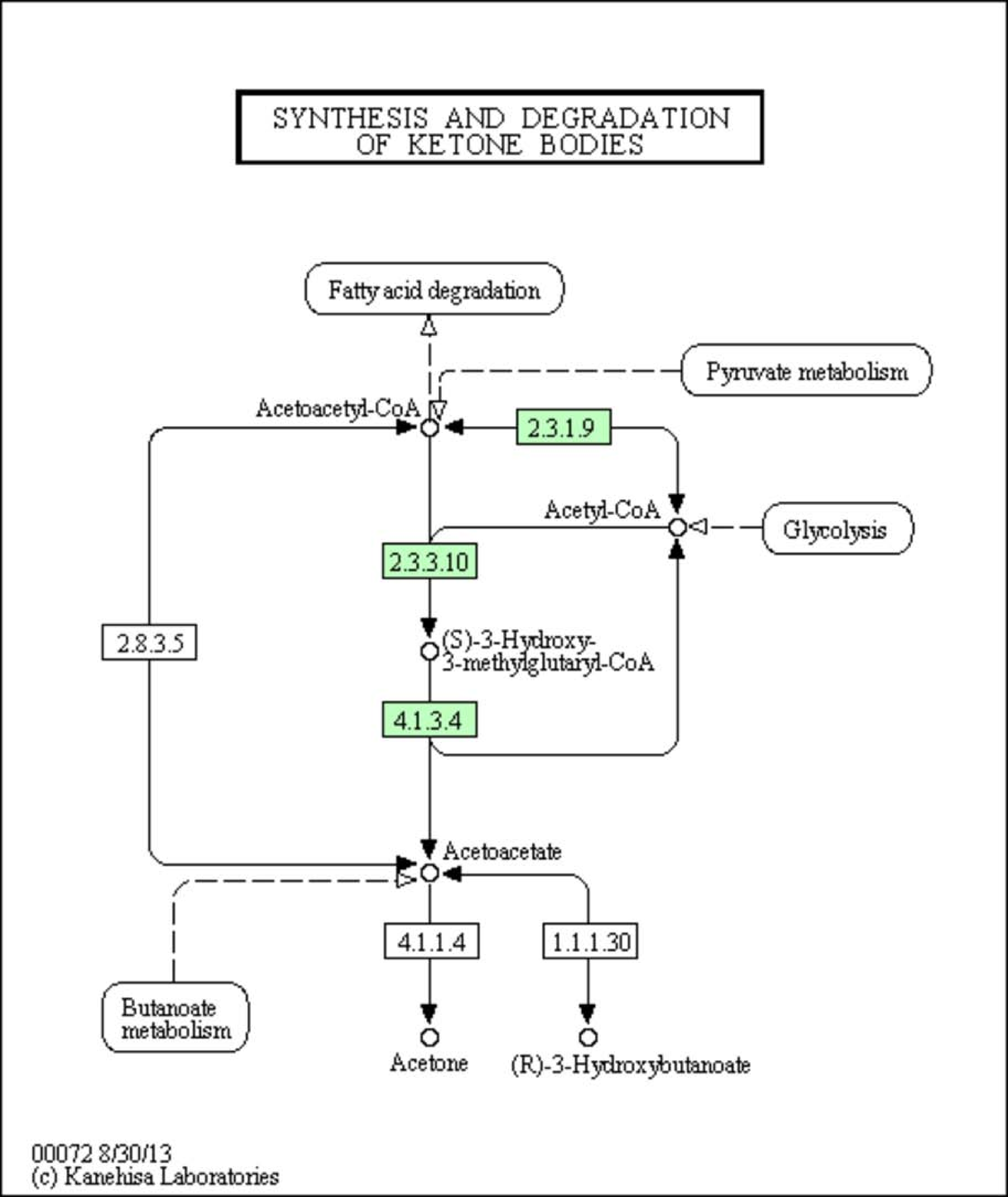
SYNTHESIS AND DEGRADATION
OF KETONE BODIES
Fatty acid degradation
Pyruvate metabolism
Acetoacetyl-CoA
2.3.1.9
Acetyl-CoA
Glycolysis
2.3.3.10
2.8.3.5
(S)-3-Hydroxy-
3-methylglutaryl-CoA
4.1.3.4
Acetoacetate
4.1.1.4
1.1.1.30
Butanoate
metabolism
Acetone
(R)-3-Hydroxybutanoate
00072 8/30/13
(c) Kanehisa Laboratories

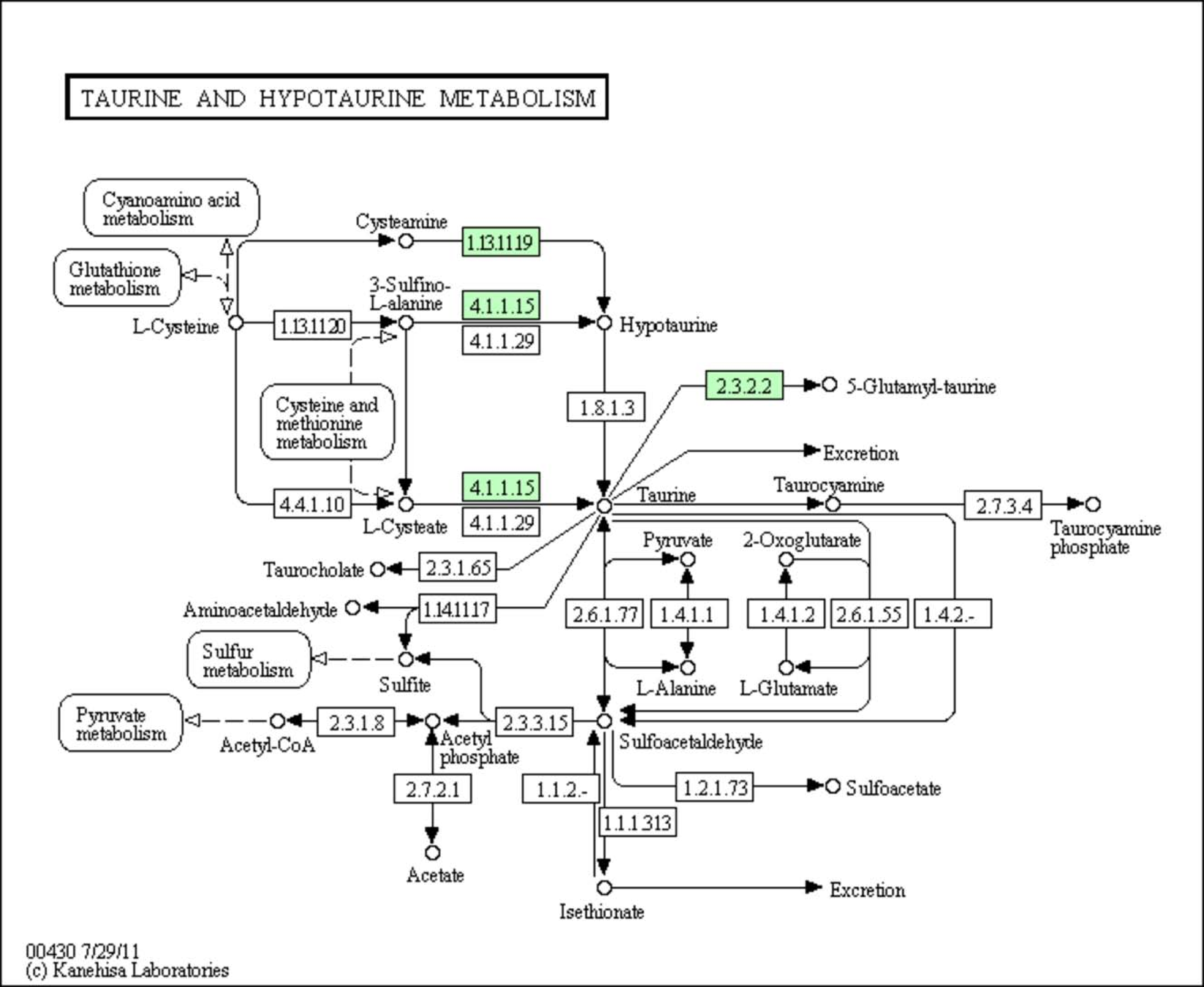

TAURINE AND HYPOTAURINE METABOLISM
Cyanoamino acid metabolism
Glutathione metabolism
Cysteamine
1.13.11.19
3-Sulfino-L-alanine
4.1.1.15
L-Cysteine
1.13.11.20
Hypotaurine
4.1.1.29
2.3.2.2
5-Glutamyl-taurine
Cysteine and methionine metabolism
1.8.1.3
Excretion
4.1.1.15
Taurine
Taurocyamine
4.4.1.10
2.7.3.4
L-Cysteate
4.1.1.29
Taurocyamine phosphate
Pyruvate
2-Oxoglutarate
Taurocholate
2.3.1.65
Aminoacetaldehyde
1.14.11.17
2.6.1.77
1.4.1.1
1.4.1.2
2.6.1.55
1.4.2.-
Sulfur metabolism
Sulfite
L-Alanine
L-Glutamate
Pyruvate metabolism
2.3.1.8
2.3.3.15
Acetyl-CoA
Acetyl phosphate
Sulfoacetaldehyde
2.7.2.1
1.1.2.-
1.2.1.73
Sulfoacetate
1.1.1.313
Acetate
Excretion
Isethionate
00430 7/29/11
(c) Kanehisa Laboratories

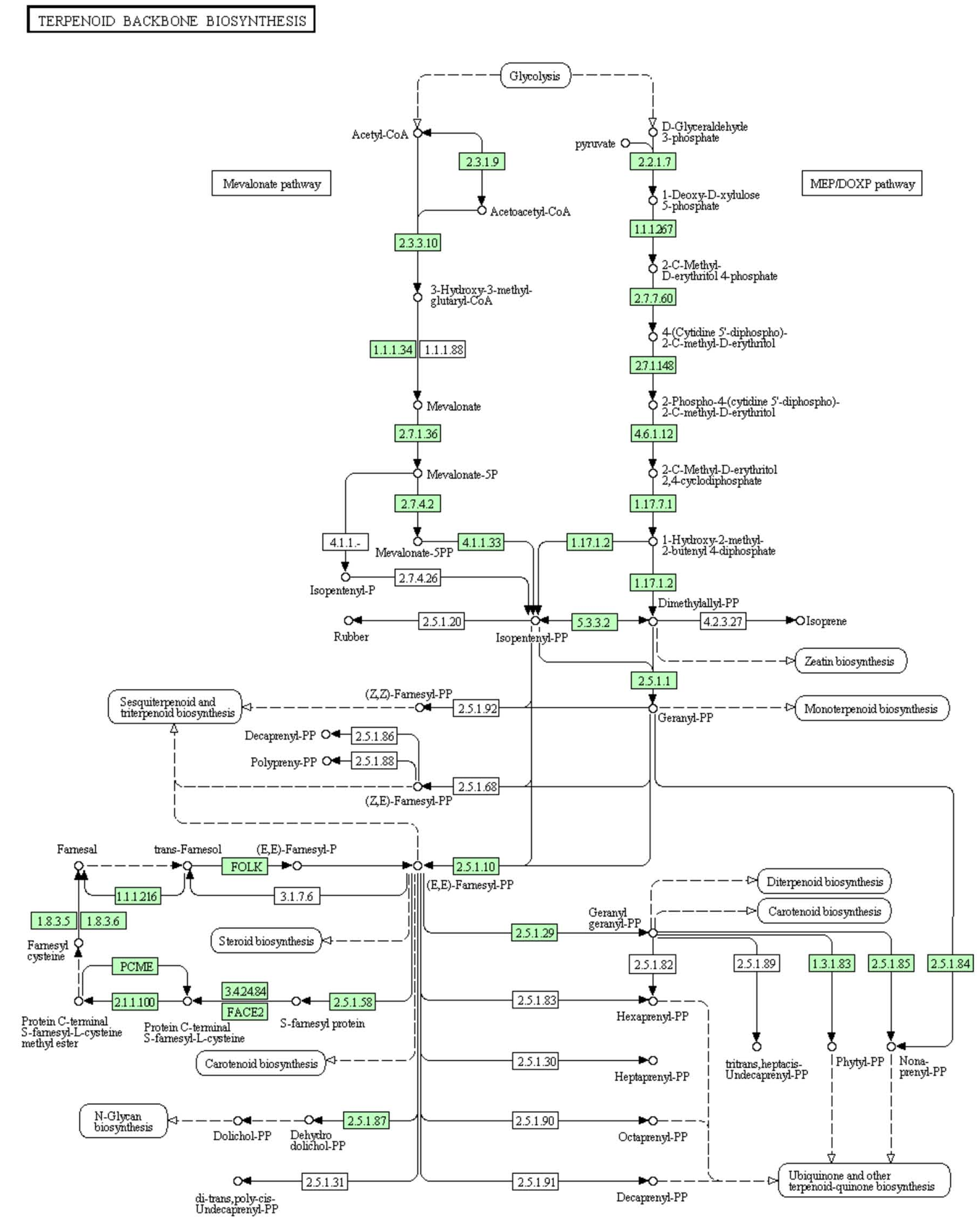

00900 2/12/14
(c) Kanehisa Laboratories

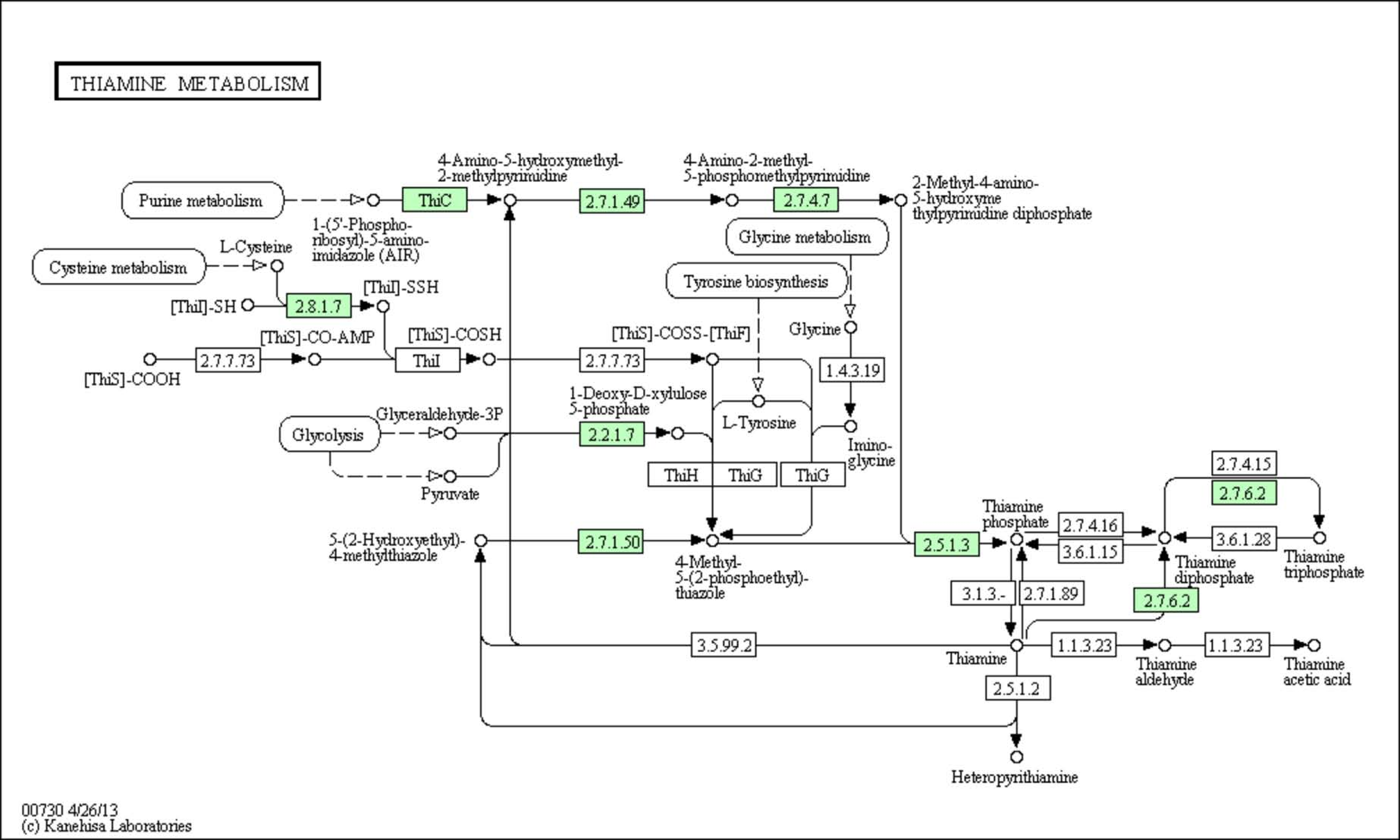
THIAMINE METABOLISM
Purine metabolism
ThiC
4-Amino-5-hydroxymethyl-2-methylpyrimidine
2.7.1.49
4-Amino-2-methyl-5-phosphomethylpyrimidine
2.7.4.7
2-Methyl-4-amino-5-hydroxymethylpyrimidine diphosphate
1-(5'-Phosphoribosyl)-5-aminoimidazole (AIR)
Cysteine metabolism
L-Cysteine
[ThiI]-SH
2.8.1.7
[ThiI]-SSH
Glycine metabolism
Tyrosine biosynthesis
Glycine
[ThiS]-COOH
2.7.7.73
[ThiS]-CO-AMP
ThiI
[ThiS]-COSH
2.7.7.73
[ThiS]-COSS-[ThiF]
1.4.3.19
1-Deoxy-D-xylulose 5-phosphate
L-Tyrosine
Glycolysis
Glyceraldehyde-3P
2.2.1.7
Imino-glycine
Pyruvate
ThiH
ThiG
ThiG
2.7.4.15
2.7.6.2
Thiamine phosphate
2.7.4.16
5-(2-Hydroxyethyl)-4-methylthiazole
2.7.1.50
4-Methyl-5-(2-phosphoethyl)-thiazole
2.5.1.3
3.6.1.15
3.6.1.28
Thiamine diphosphate
Thiamine triphosphate
3.1.3.-
2.7.1.89
2.7.6.2
3.5.99.2
Thiamine
1.1.3.23
Thiamine aldehyde
1.1.3.23
Thiamine acetic acid
2.5.1.2
Heteropyrithiamine
00730 4/26/13
(c) Kanehisa Laboratories

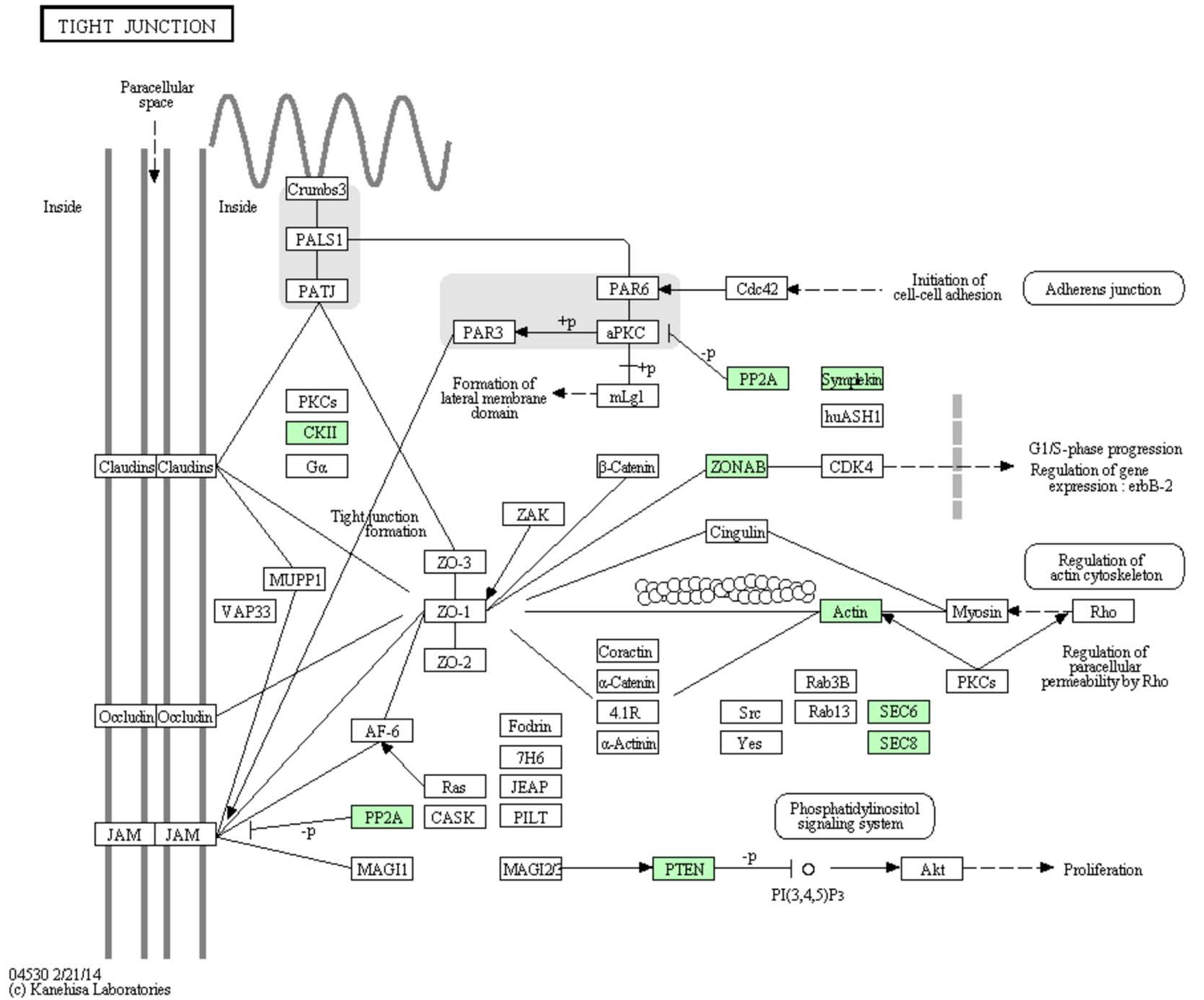
TIGHT JUNCTION
Paracellular space
Inside
Inside
Crumbs3
PALS1
PATJ
PAR6
Cdc42
Initiation of cell-cell adhesion
Adherens junction
PAR3
+p
aPKC
-p
PP2A
Symplekin
+p
Formation of lateral membrane domain
mLgl
PKCs
CKII
huASH1
Gα
Claudins
Claudins
β-Catenin
ZONAB
CDK4
G1/S-phase progression
Regulation of gene expression : erbB-2
ZAK
Tight junction formation
Cingulin
ZO-3
Regulation of actin cytoskeleton
MUPP1
VAP33
ZO-1
Actin
Myosin
Rho
ZO-2
Coractin
α-Catenin
Regulation of paracellular permeability by Rho
Rab3B
PKCs
Occludin
Occludin
4.1R
Src
Rab13
SEC6
AF-6
Fodrin
α-Actinin
Yes
SEC8
7H6
Ras
JEAP
Phosphatidylinositol signaling system
PP2A
CASK
PILT
JAM
JAM
-p
MAGI1
MAGI2/3
PTEN
-p
Akt
Proliferation
PI(3,4,5)P3
04530 2/21/14
(c) Kanehisa Laboratories

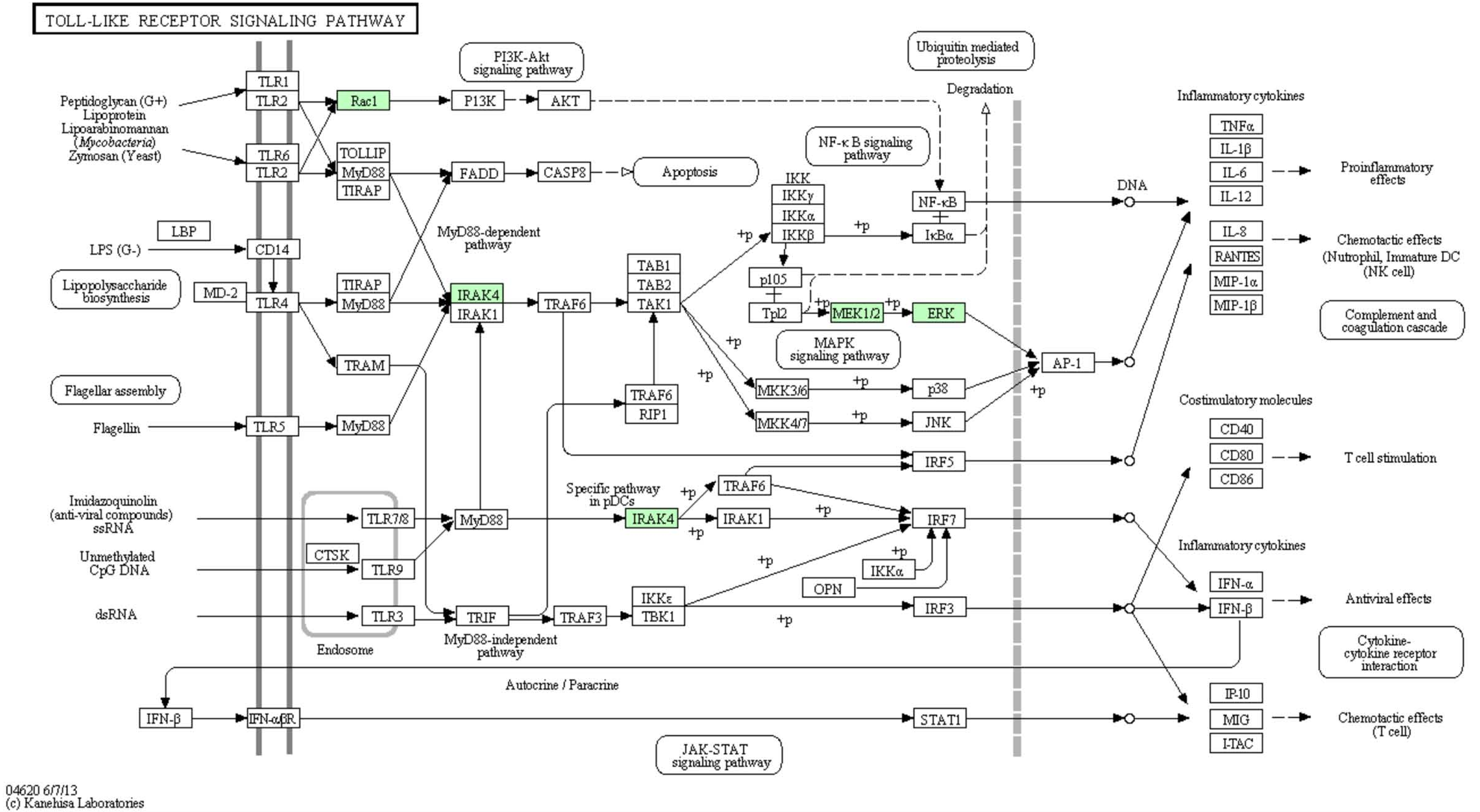
TOLL-LIKE RECEPTOR SIGNALING PATHWAY
PI3K-Akt signaling pathway
Ubiquitin mediated proteolysis
Degradation
Inflammatory cytokines
Peptidoglycan (G+)
Lipoprotein
Lipoarabinomannan
(Mycobacteria)
Zymosan (Yeast)
TLR1
TLR2
Rac1
PI3K
AKT
TLR6
TLR2
TOLLIP
MyD88
TIRAP
FADD
CASP8
Apoptosis
NF-κB signaling pathway
IKK
IKKγ
IKKα
IKKβ
NF-κB
IκBα
DNA
TNFα
IL-1β
IL-6
IL-12
Proinflammatory effects
IL-8
RANTES
MIP-1α
MIP-1β
Chemotactic effects
(Nutrophil, Immature DC
(NK cell)
LBP
LPS (G-)
CD14
MyD88-dependent pathway
Lipopolysaccharide biosynthesis
MD-2
TLR4
TIRAP
MyD88
IRAK4
IRAK1
TRAF6
TAB1
TAB2
TAK1
p105
Tpl2
MEK1/2
ERK
Complement and coagulation cascade
MAPK signaling pathway
TRAM
AP-1
Flagellar assembly
TRAF6
RIP1
MKK3/6
p38
MKK4/7
JNK
Costimulatory molecules
Flagellin
TLR5
MyD88
CD40
CD80
CD86
T cell stimulation
IRF5
Specific pathway in pDCs
TRAF6
Imidazoquinolin
(anti-viral compounds)
ssRNA
TLR7/8
MyD88
IRAK4
IRAK1
IRF7
Inflammatory cytokines
CTSK
Unmethylated CpG DNA
TLR9
IKKα
OPN
IFN-α
dsRNA
TLR3
TRIF
TRAF3
IKKε
TBK1
IRF3
IFN-β
Antiviral effects
Endosome
MyD88-independent pathway
Cytokine-cytokine receptor interaction
Autocrine / Paracrine
IP-10
IFN-β
IFN-αβR
STAT1
MIG
Chemotactic effects
(T cell)
I-TAC
JAK-STAT signaling pathway
+p
04620 6/7/13
(c) Kanehisa Laboratories

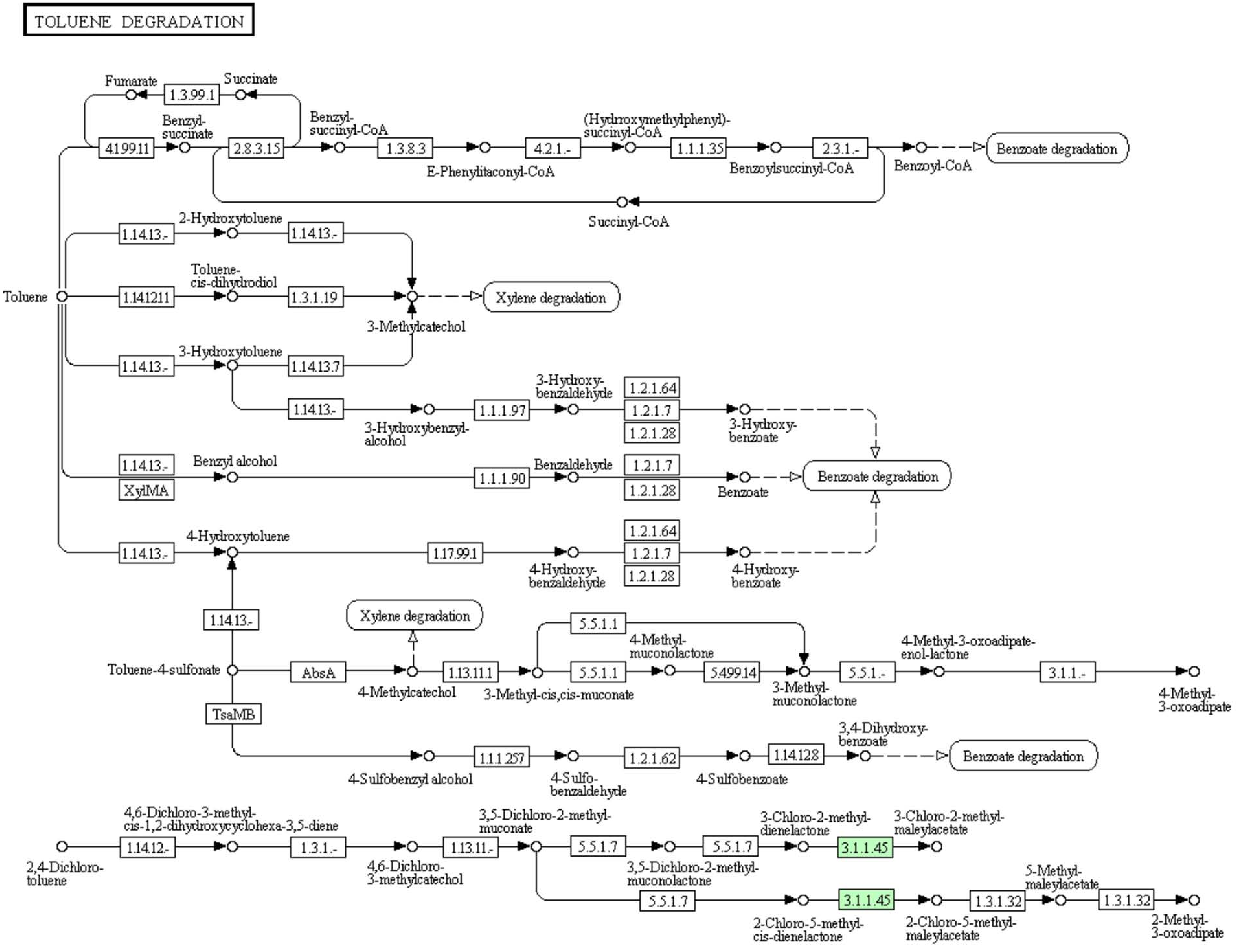

00623 3/6/14
(c) Kanehisa Laboratories

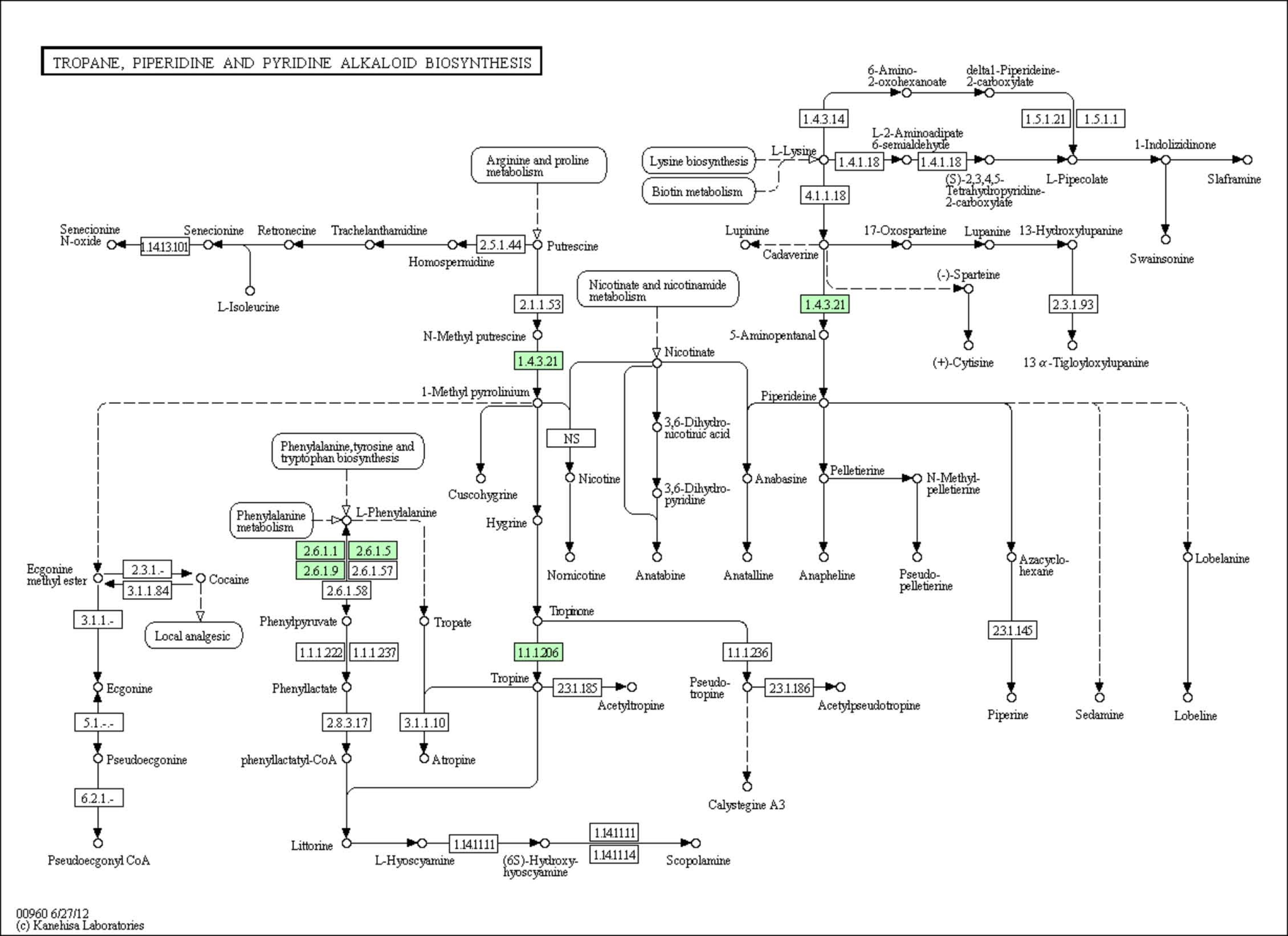
TROPANE, PIPERIDINE AND PYRIDINE ALKALOID BIOSYNTHESIS
6-Amino-
2-oxohexanoate
delta1-Piperideine-
2-carboxylate
1.4.3.14
1.5.1.21
1.5.1.1
L-2-Aminoadipate
6-semialdehyde
1-Indolizidinone
Lysine biosynthesis
L-Lysine
1.4.1.18
1.4.1.18
Slaframine
Biotin metabolism
(S)-2,3,4,5-
Tetrahydropyridine-
2-carboxylate
L-Pipecolate
4.1.1.18
Arginine and proline
metabolism
Senecionine
N-oxide
1.14.13.101
Senecionine
Retronecine
Trachelanthamidine
2.5.1.44
Putrescine
Homospermidine
L-Isoleucine
Lupinine
17-Oxosparteine
Lupanine
13-Hydroxylupanine
Cadaverine
Swainsonine
(-)-Sparteine
Nicotinate and nicotinamide
metabolism
2.1.1.53
1.4.3.21
2.3.1.93
N-Methyl putrescine
5-Aminopentanal
1.4.3.21
Nicotinate
(+)-Cytisine
13 α-Tigloyloxylupanine
1-Methyl pyrrolinium
Piperideine
3,6-Dihydro-
nicotinic acid
NS
Phenylalanine,tyrosine and
tryptophan biosynthesis
Pelletierine
N-Methyl-
pelletierine
Nicotine
Anabasine
Cuscohygrine
3,6-Dihydro-
pyridine
Phenylalanine
metabolism
L-Phenylalanine
Hygrine
2.6.1.1
2.6.1.5
Nornicotine
Anatabine
Anatalline
Anapheline
Pseudo-
pelletierine
Azacyclo-
hexane
Lobelanine
Ecgonine
methyl ester
2.3.1.-
2.6.1.9
2.6.1.57
Cocaine
3.1.1.84
2.6.1.58
3.1.1.-
Phenylpyruvate
Tropate
Tropinone
Local analgesic
1.1.1.222
1.1.1.237
1.1.1.206
1.1.1.236
2.3.1.145
Tropine
Pseudo-
tropine
Ecgonine
Phenyllactate
2.3.1.185
2.3.1.186
Acetyltropine
Acetylpseudotropine
Piperine
Sedamine
Lobeline
5.1.-.-
2.8.3.17
3.1.1.10
Pseudoecgonine
phenyllactatyl-CoA
Atropine
6.2.1.-
Calystegine A3
1.14.11.11
1.14.11.11
1.14.11.14
Pseudoecgonyl CoA
Littorine
L-Hyoscyamine
(6S)-Hydroxy-
hyoscyamine
Scopolamine
00960 6/27/12
(c) Kanehisa Laboratories

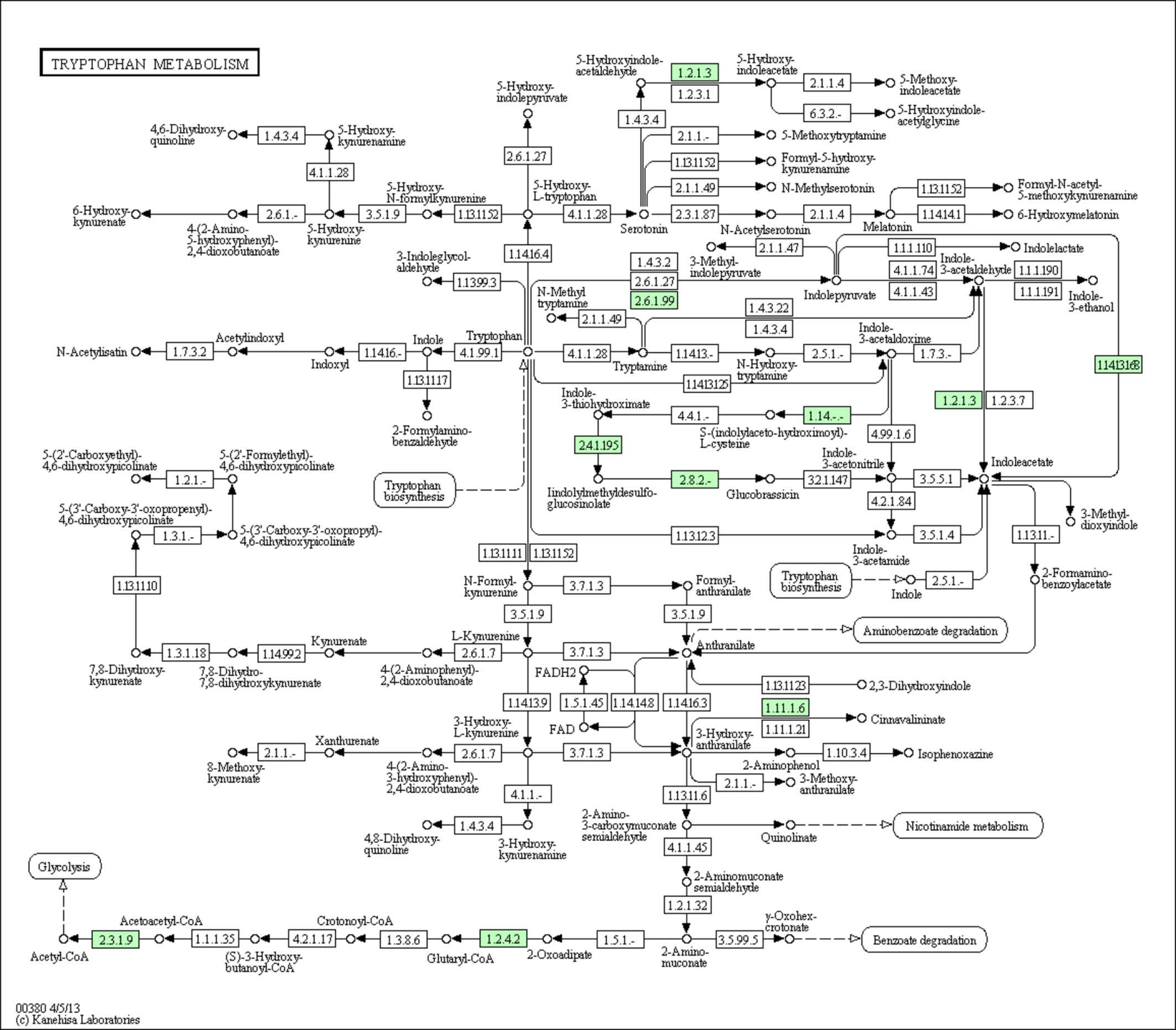
TRYPTOPHAN METABOLISM
5-Hydroxyindole-acetaldehyde
1.2.1.3
1.2.3.1
5-Hydroxy-indoleacetate
2.1.1.4
5-Methoxy-indoleacetate
6.3.2.-
5-Hydroxyindole-acetylglycine
5-Hydroxy-indolepyruvate
1.4.3.4
2.1.1.-
5-Methoxytryptamine
1.13.11.52
Formyl-5-hydroxy-kynurenamine
2.1.1.49
N-Methylserotonin
2.3.1.87
N-Acetylserotonin
Melatonin
Formyl-N-acetyl-5-methoxykynurenamine
1.14.14.1
6-Hydroxymelatonin
4,6-Dihydroxy-quinoline
5-Hydroxy-kynurenamine
4.1.1.28
2.6.1.27
6-Hydroxy-kynurenate
2.6.1.-
4-(2-Amino-5-hydroxyphenyl)-2,4-dioxobutanoate
5-Hydroxy-kynurenine
3.5.1.9
5-Hydroxy-N-formylkynurenine
5-Hydroxy-L-tryptophan
Serotonin
1.14.16.4
3-Indoleglycol-aldehyde
1.13.99.3
1.4.3.2
2.6.1.99
3-Methyl-indolepyruvate
2.1.1.47
1.1.1.110
Indolelactate
4.1.1.74
4.1.1.43
Indole-3-acetaldehyde
1.1.1.190
1.1.1.191
Indole-3-ethanol
Indolepyruvate
N-Methyl tryptamine
1.4.3.22
Acetylindoxyl
N-Acetylisatin
1.7.3.2
Indoxyl
1.14.16.-
Indole
4.1.99.1
Tryptophan
Tryptamine
1.14.13.-
N-Hydroxy-tryptamine
2.5.1.-
Indole-3-acetaldoxime
1.7.3.-
1.13.11.17
2-Formylamino-benzaldehyde
1.14.13.125
1.14.13.168
Indole-3-thiohydroximate
4.4.1.-
1.14.-.-
S-(indolylaceto-hydroximoyl)-L-cysteine
1.2.3.7
4.99.1.6
2.4.1.195
Indoleacetate
5-(2'-Carboxyethyl)-4,6-dihydroxypicolinate
1.2.1.-
5-(2'-Formylethyl)-4,6-dihydroxypicolinate
Tryptophan biosynthesis
Indole-3-acetonitrile
Indolylmethyldesulfo-glucosinolate
2.8.2.-
Glucobrassicin
3.2.1.147
3.5.5.1
3-Methyl-dioxyindole
4.2.1.84
5-(3'-Carboxy-3'-oxopropenyl)-4,6-dihydroxypicolinate
1.3.1.-
5-(3'-Carboxy-3'-oxopropyl)-4,6-dihydroxypicolinate
1.13.12.3
3.5.1.4
Indole-3-acetamide
1.13.11.-
1.13.11.11
1.13.11.10
N-Formyl-kynurenine
3.7.1.3
Formyl-anthranilate
Indole
2-Formamino-benzoylacetate
Aminobenzoate degradation
Anthranilate
L-Kynurenine
Kynurenate
7,8-Dihydroxy-kynurenate
1.3.1.18
1.14.99.2
7,8-Dihydro-7,8-dihydroxykynurenate
2.6.1.7
4-(2-Aminophenyl)-2,4-dioxobutanoate
FADH2
1.14.13.9
1.5.1.45
1.14.14.8
1.14.16.3
1.13.11.23
2,3-Dihydroxyindole
1.11.1.6
1.11.1.21
Cinnavalininate
FAD
3-Hydroxy-L-kynurenine
3-Hydroxy-anthranilate
Xanthurenate
2-Aminophenol
1.10.3.4
Isophenoxazine
8-Methoxy-kynurenate
4-(2-Amino-3-hydroxyphenyl)-2,4-dioxobutanoate
4.1.1.-
3-Methoxy-anthranilate
1.13.11.6
1.4.3.4
4,8-Dihydroxy-quinoline
3-Hydroxy-kynurenamine
2-Amino-3-carboxymuconate semialdehyde
Quinolinate
Nicotinamide metabolism
4.1.1.45
Glycolysis
2-Aminomuconate semialdehyde
1.2.1.32
Acetoacetyl-CoA
Crotonoyl-CoA
γ-Oxohex-crotonate
2.3.1.9
1.1.1.35
4.2.1.17
1.3.8.6
1.2.4.2
1.5.1.-
3.5.99.5
Benzoate degradation
Acetyl-CoA
(S)-3-Hydroxy-butanoyl-CoA
Glutaryl-CoA
2-Oxoadipate
2-Amino-muconate
00380 4/5/13
(c) Kanehisa Laboratories

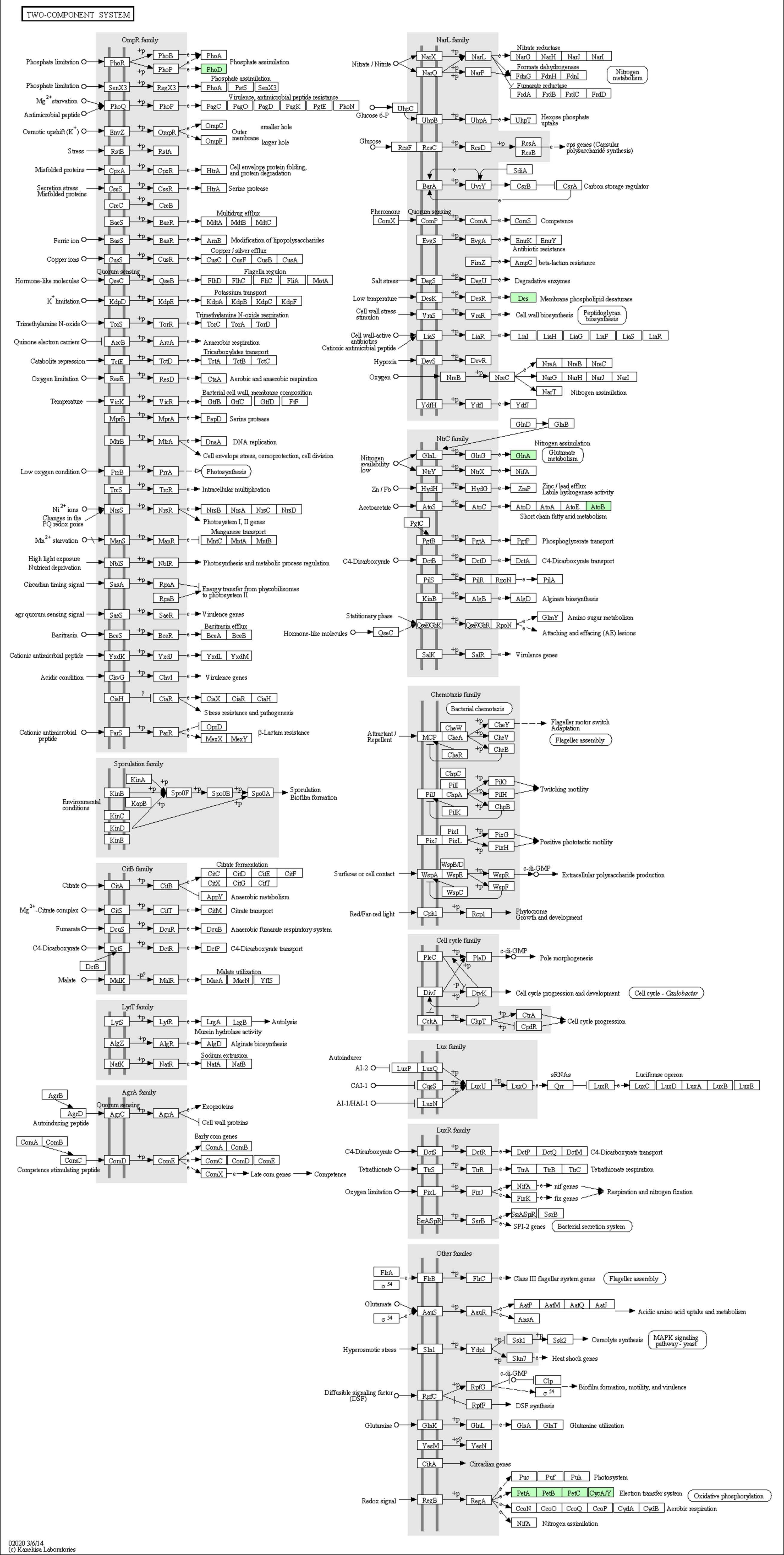

TWO-COMPONENT SYSTEM
OmpR family
NarL family
NtrC family
Chemotaxis family
Sporulation family
CitB family
Cell cycle family
LytT family
Lux family
AgrA family
LuxR family
Other families
Phosphate assimilation
Virulence, antimicrobial peptide resistance
Nitrate reductase
Formate dehydrogenase
Fumarate reductase
Nitrogen metabolism
Hexose phosphate uptake
cps genes (Capsular polysaccharide synthesis)
Carbon storage regulator
Competence
Antibiotic resistance
beta-lactam resistance
Degradative enzymes
Membrane phospholipid desaturase
Cell wall biosynthesis
Peptidoglycan biosynthesis
Nitrogen assimilation
Glutamate metabolism
Zinc / lead efflux
Labile hydrogenase activity
Short chain fatty acid metabolism
Phosphoglycerate transport
C4-Dicarboxyrate transport
Alginate biosynthesis
Amino sugar metabolism
Attaching and effacing (AE) lesions
Virulence genes
Bacterial chemotaxis
Flageller motor switch
Adaptation
Flageller assembly
Twitching motility
Positive phototactic motility
Extracellular polysaccharide production
Phytocrome
Growth and development
Pole morphogenesis
Cell cycle progression and development
Cell cycle - Caulobacter
Cell cycle progression
sRNAs
Luciferase operon
Sporulation
Biofilm formation
Citrate fermentation
Anaerobic metabolism
Citrate transport
Anaerobic fumarate respiratory system
Malate utilization
Autolysis
Murein hydrolase activity
Sodium extrusion
Exoproteins
Cell wall proteins
Early com genes
Late com genes
Competence stimulating peptide
Autoinducing peptide
Tetrathionate respiration
Respiration and nitrogen fixation
SPI-2 genes
Bacterial secretion system
Class III flagellar system genes
Acidic amino acid uptake and metabolism
Osmolyte synthesis
MAPK signaling pathway - yeast
Heat shock genes
Biofilm formation, motility, and virulence
DSF synthesis
Glutamine utilization
Circadian genes
Photosystem
Electron transfer system
Oxidative phosphorylation
Aerobic respiration
Nitrogen assimilation
02020 3/6/14
(c) Kanehisa Laboratories

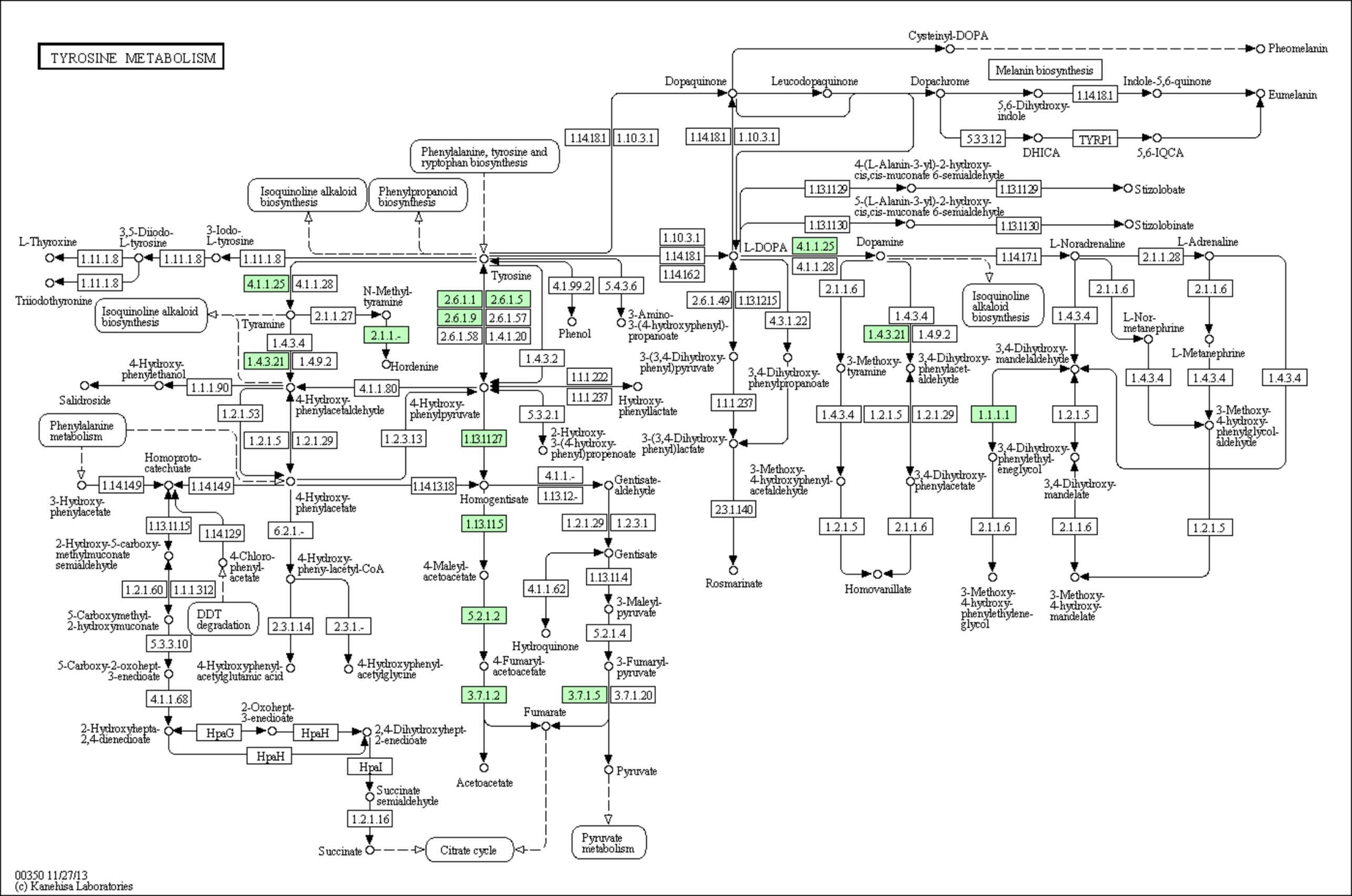

TYROSINE METABOLISM
Cysteinyl-DOPA
Pheomelanin
Melanin biosynthesis
Dopaquinone
Leucodopaquinone
Dopachrome
Indole-5,6-quinone
Eumelanin
5,6-Dihydroxy-indole
DHICA
5,6-IQCA
TYRP1
5.3.3.12
1.14.18.1
1.10.3.1
Phenylalanine, tyrosine and tryptophan biosynthesis
Isoquinoline alkaloid biosynthesis
Phenylpropanoid biosynthesis
4-(L-Alanin-3-yl)-2-hydroxy-cis,cis-muconate 6-semialdehyde
5-(L-Alanin-3-yl)-2-hydroxy-cis,cis-muconate 6-semialdehyde
1.13.11.29
1.13.11.30
Stizolobate
Stizolobinate
L-Thyroxine
3,5-Diiodo-L-tyrosine
3-Iodo-L-tyrosine
1.11.1.8
Triiodothyronine
L-DOPA
4.1.1.25
4.1.1.28
Dopamine
1.14.17.1
L-Noradrenaline
L-Adrenaline
2.1.1.28
1.14.16.2
Tyrosine
4.1.99.2
5.4.3.6
Phenol
3-Amino-3-(4-hydroxyphenyl)-propanoate
2.6.1.1
2.6.1.5
2.6.1.9
2.6.1.57
2.6.1.58
1.4.1.20
N-Methyl-tyramine
Tyramine
2.1.1.27
2.1.1.-
Hordenine
1.4.3.4
1.4.3.21
1.4.9.2
1.4.3.2
4-Hydroxy-phenylethanol
1.1.1.90
Salidroside
4.1.1.80
4-Hydroxy-phenylacetaldehyde
4-Hydroxy-phenylpyruvate
1.1.1.222
1.1.1.237
Hydroxy-phenyllactate
5.3.2.1
2-Hydroxy-3-(4-hydroxyphenyl)propenoate
1.2.1.53
1.2.1.5
1.2.1.29
1.2.3.13
1.13.11.27
Phenylalanine metabolism
Homoprotocatechuate
3-Hydroxy-phenylacetate
1.14.14.9
4-Hydroxy-phenylacetate
1.14.13.18
Homogentisate
4.1.1.-
1.13.12.-
Gentisate-aldehyde
1.2.1.29
1.2.3.1
Gentisate
1.13.11.15
1.14.12.9
2-Hydroxy-5-carboxy-methylmuconate semialdehyde
4-Chloro-phenyl-acetate
6.2.1.-
4-Hydroxy-pheny-lacetyl-CoA
1.13.11.5
4-Maleyl-acetoacetate
4.1.1.62
1.13.11.4
3-Maleyl-pyruvate
1.2.1.60
1.1.1.312
DDT degradation
5-Carboxymethyl-2-hydroxymuconate
5.3.3.10
2.3.1.14
2.3.1.-
5.2.1.2
Hydroquinone
5.2.1.4
5-Carboxy-2-oxohept-3-enedioate
4-Hydroxyphenyl-acetylglutamic acid
4-Hydroxyphenyl-acetylglycine
4-Fumaryl-acetoacetate
3-Fumaryl-pyruvate
4.1.1.68
3.7.1.2
3.7.1.5
3.7.1.20
Fumarate
2-Oxohept-3-enedioate
2-Hydroxyhepta-2,4-dienedioate
HpaG
HpaH
2,4-Dihydroxyhept-2-enedioate
HpaI
Succinate semialdehyde
Acetoacetate
Pyruvate
1.2.1.16
Succinate
Citrate cycle
Pyruvate metabolism
2.6.1.49
1.13.12.15
4.3.1.22
3-(3,4-Dihydroxy-phenyl)pyruvate
3,4-Dihydroxy-phenylpropanoate
1.1.1.237
3-(3,4-Dihydroxy-phenyl)lactate
2.3.1.140
Rosmarinate
2.1.1.6
3-Methoxy-tyramine
3,4-Dihydroxy-phenylacet-aldehyde
3,4-Dihydroxy-mandelaldehyde
L-Nor-metanephrine
L-Metanephrine
1.1.1.1
3-Methoxy-4-hydroxyphenyl-acetaldehyde
3,4-Dihydroxy-phenylacetate
3,4-Dihydroxy-phenylethyl-eneglycol
3,4-Dihydroxy-mandelate
3-Methoxy-4-hydroxy-phenylglycol-aldehyde
Homovanillate
3-Methoxy-4-hydroxy-phenylethylene-glycol
3-Methoxy-4-hydroxy-mandelate
00350 11/27/13
(c) Kanehisa Laboratories

UBIQUINONE AND OTHER TERPENOID-QUINONE BIOSYNTHESIS

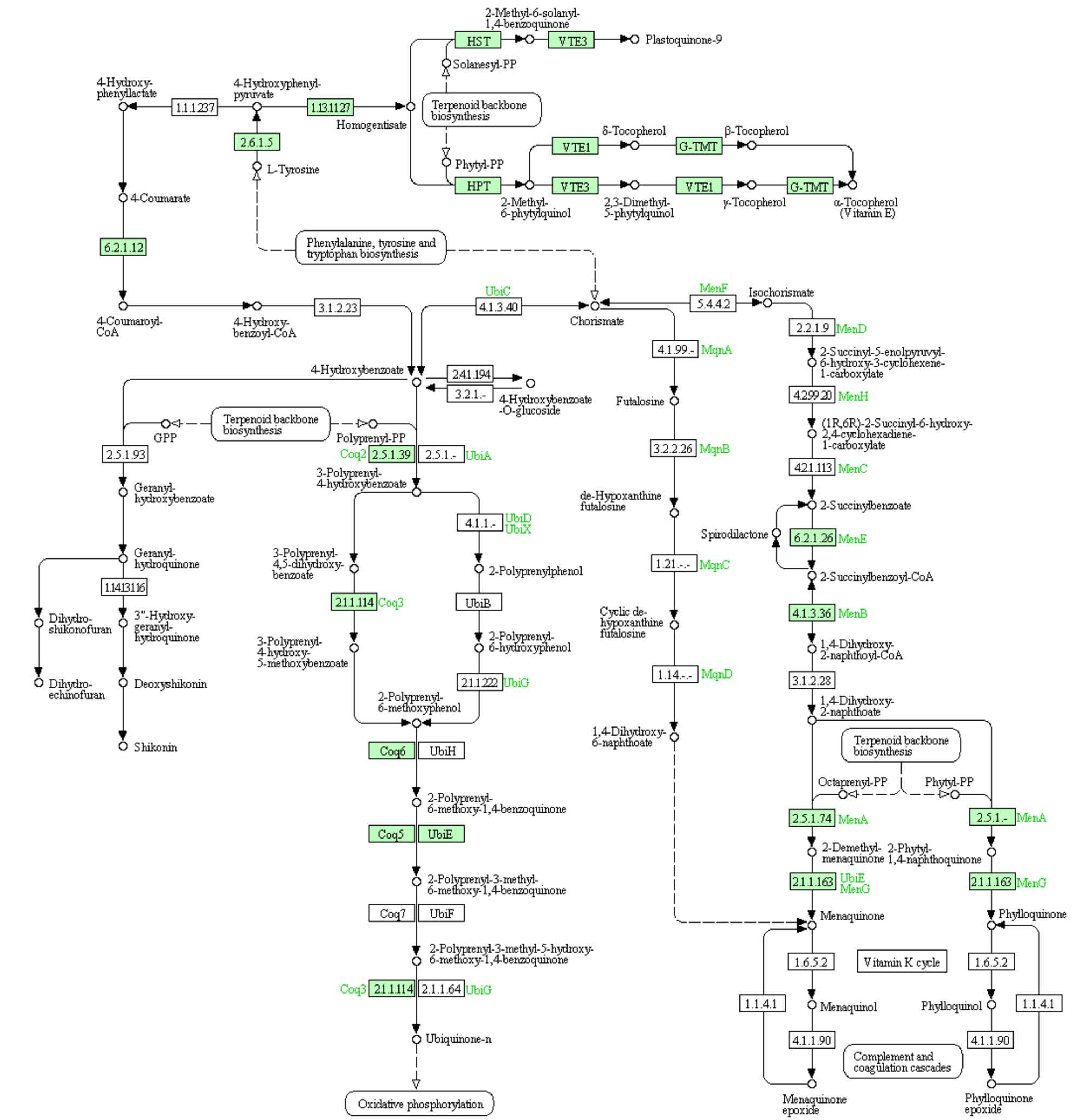

00130 6/19/12

(c) Kanehisa Laboratories

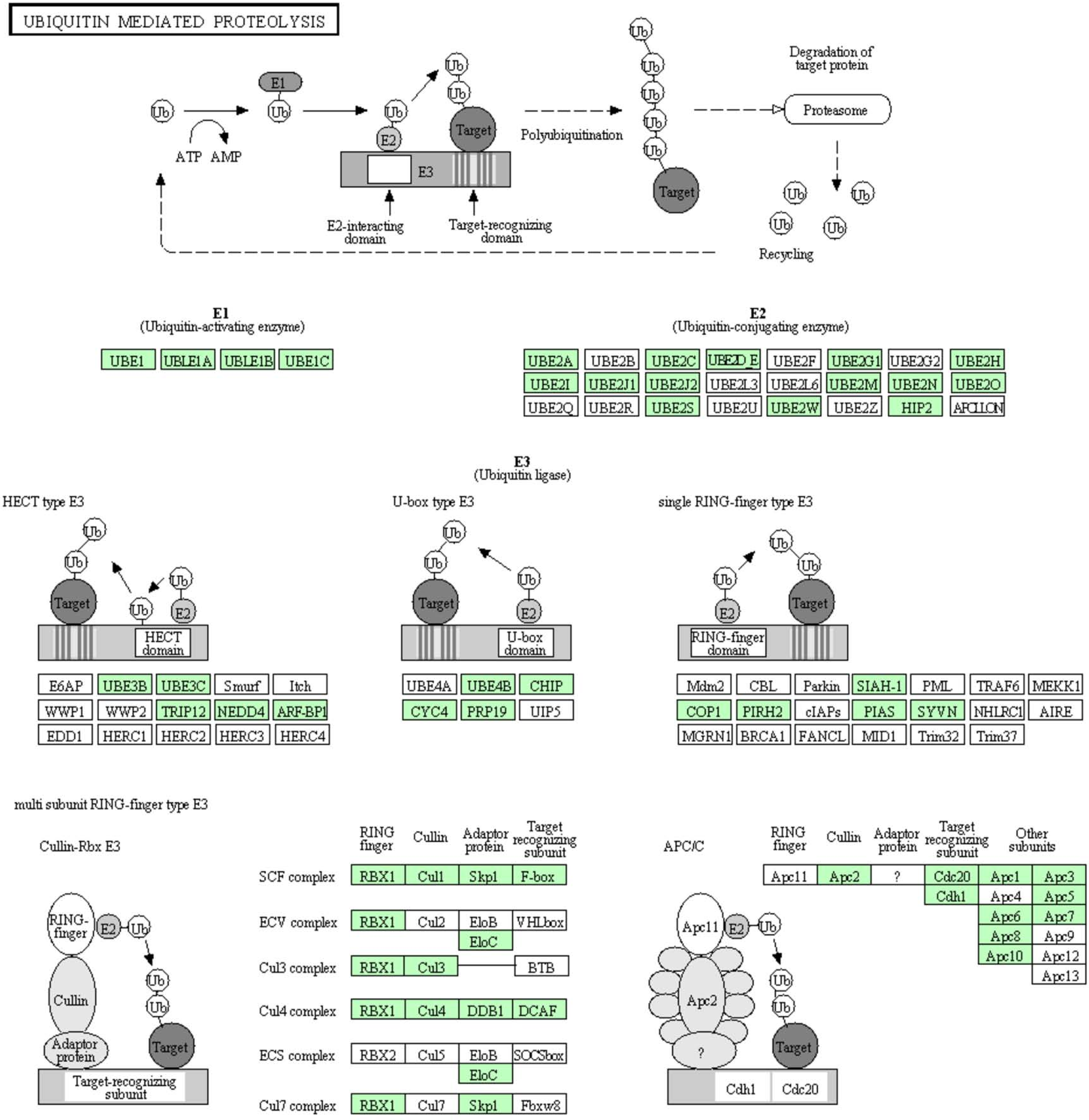

04120 6/8/12
(c) Kanehisa Laboratories

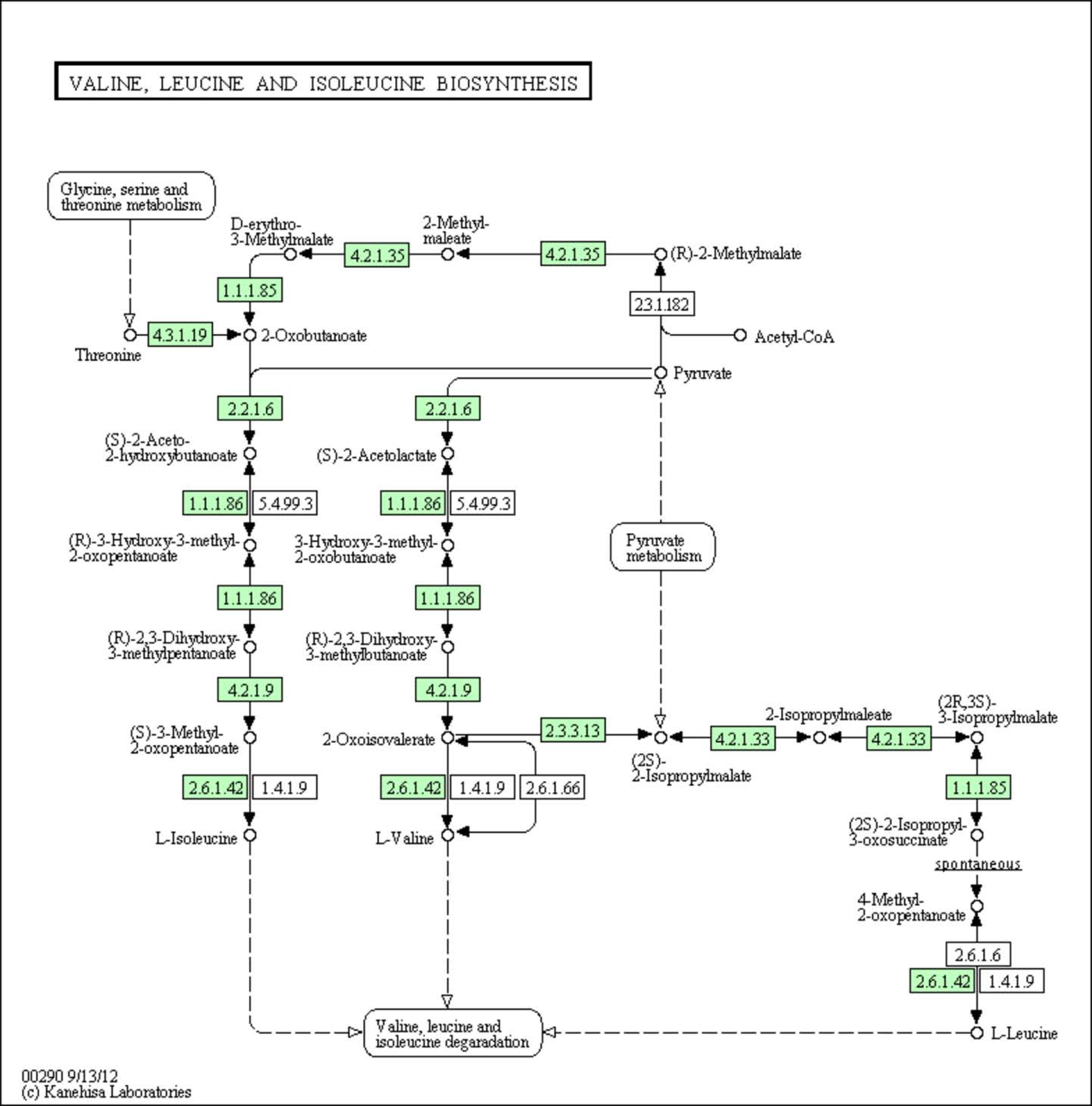
VALINE, LEUCINE AND ISOLEUCINE BIOSYNTHESIS
Glycine, serine and threonine metabolism
D-erythro-3-Methylmalate
4.2.1.35
2-Methyl-maleate
4.2.1.35
(R)-2-Methylmalate
1.1.1.85
2.3.1.182
Threonine
4.3.1.19
2-Oxobutanoate
Acetyl-CoA
Pyruvate
2.2.1.6
2.2.1.6
(S)-2-Aceto-2-hydroxybutanoate
(S)-2-Acetolactate
1.1.1.86
5.4.99.3
1.1.1.86
5.4.99.3
(R)-3-Hydroxy-3-methyl-2-oxopentanoate
3-Hydroxy-3-methyl-2-oxobutanoate
Pyruvate metabolism
1.1.1.86
1.1.1.86
(R)-2,3-Dihydroxy-3-methylpentanoate
(R)-2,3-Dihydroxy-3-methylbutanoate
4.2.1.9
4.2.1.9
(S)-3-Methyl-2-oxopentanoate
2-Oxoisovalerate
2.3.3.13
(2S)-2-Isopropylmalate
4.2.1.33
2-Isopropylmaleate
4.2.1.33
(2R,3S)-3-Isopropylmalate
2.6.1.42
1.4.1.9
2.6.1.42
1.4.1.9
2.6.1.66
1.1.1.85
L-Isoleucine
L-Valine
(2S)-2-Isopropyl-3-oxosuccinate
spontaneous
4-Methyl-2-oxopentanoate
2.6.1.6
2.6.1.42
1.4.1.9
Valine, leucine and isoleucine degaradation
L-Leucine
00290 9/13/12
(c) Kanehisa Laboratories

VALINE, LEUCINE AND ISOLEUCINE DEGRADATION

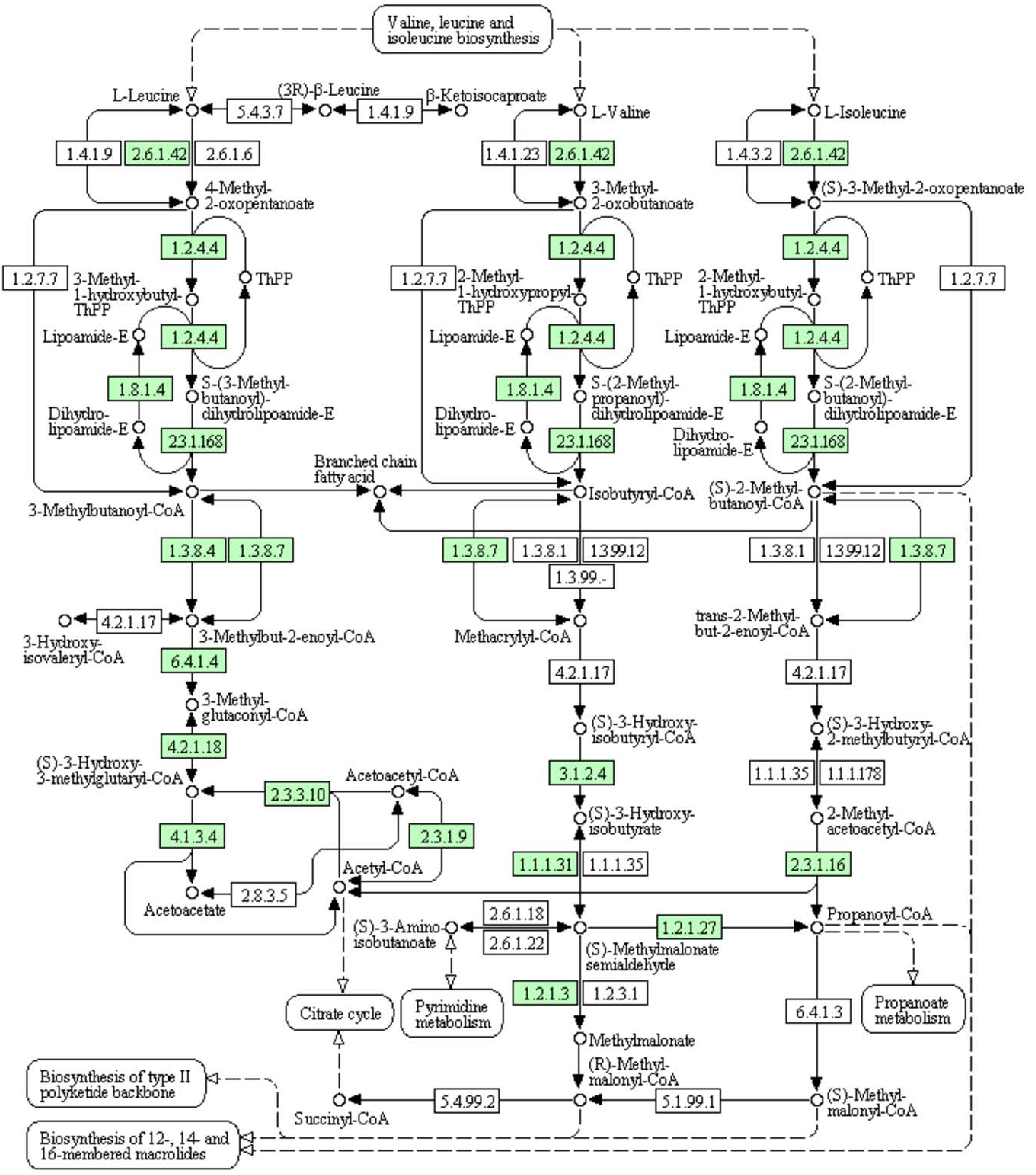

00280 9/12/12

(c) Kanehisa Laboratories

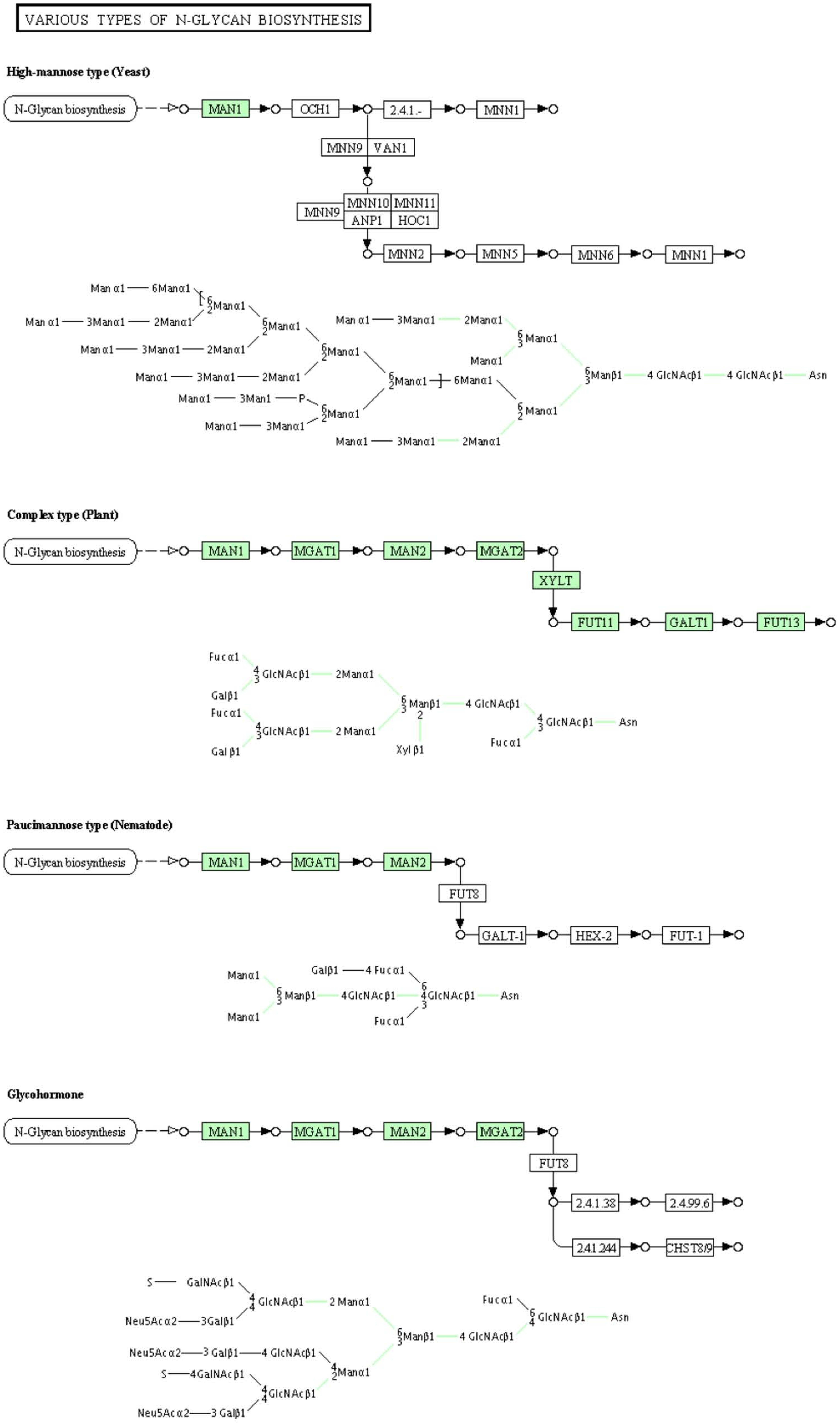

00513 11/20/12
(c) Kanehisa Laboratories

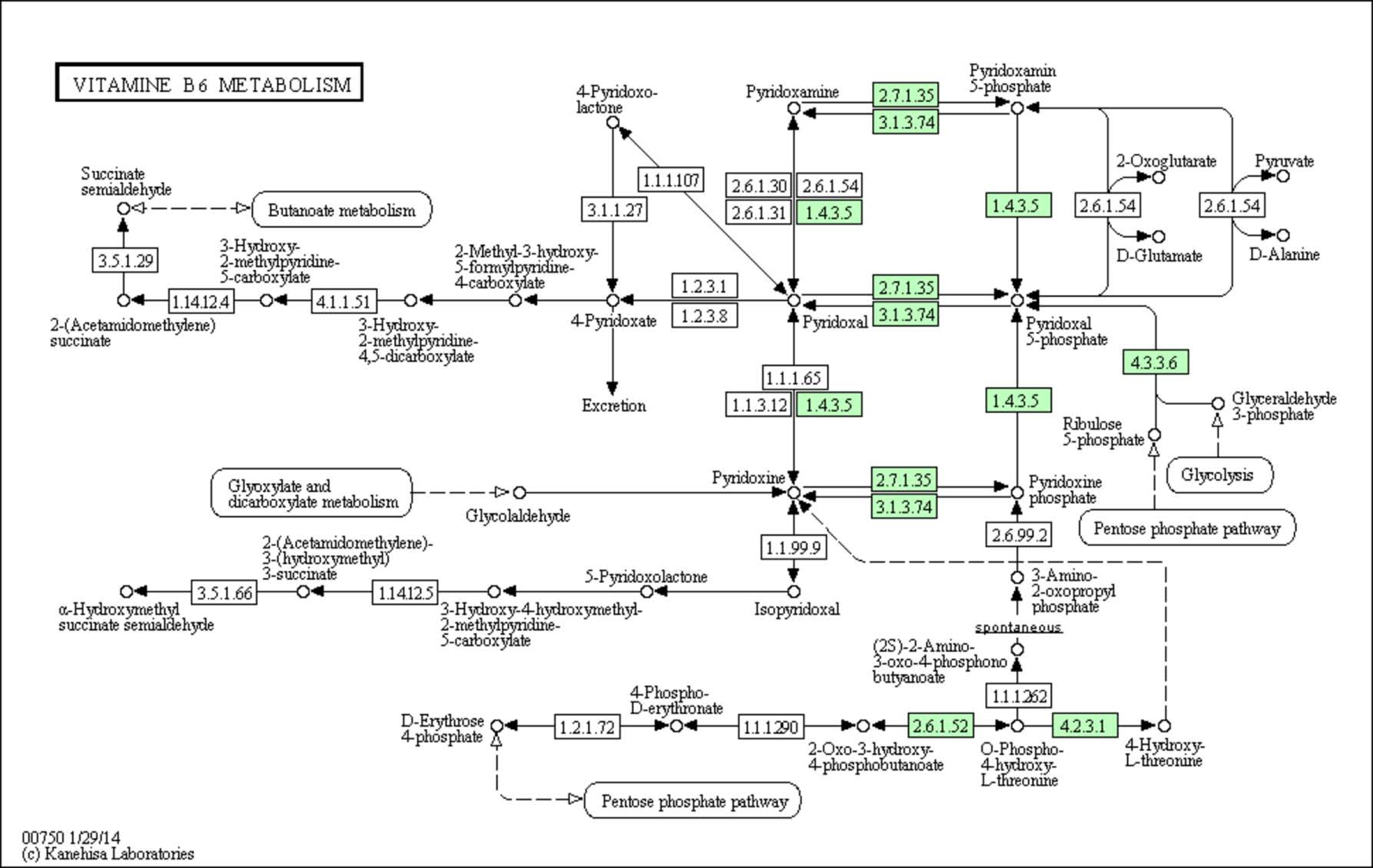
VITAMINE B6 METABOLISM
4-Pyridoxo-lactone
Pyridoxamine
2.7.1.35
3.1.3.74
Pyridoxamin 5-phosphate
Succinate semialdehyde
Butanoate metabolism
1.1.1.107
3.1.1.27
2.6.1.30
2.6.1.54
2.6.1.31
1.4.3.5
1.4.3.5
2-Oxoglutarate
2.6.1.54
D-Glutamate
Pyruvate
2.6.1.54
D-Alanine
3.5.1.29
3-Hydroxy-2-methylpyridine-5-carboxylate
2-Methyl-3-hydroxy-5-formylpyridine-4-carboxylate
1.14.12.4
4.1.1.51
1.2.3.1
1.2.3.8
2-(Acetamidomethylene) succinate
3-Hydroxy-2-methylpyridine-4,5-dicarboxylate
4-Pyridoxate
Pyridoxal
2.7.1.35
3.1.3.74
Pyridoxal 5-phosphate
4.3.3.6
Excretion
1.1.1.65
1.1.3.12
1.4.3.5
1.4.3.5
Glyceraldehyde 3-phosphate
Ribulose 5-phosphate
Glyoxylate and dicarboxylate metabolism
Pyridoxine
2.7.1.35
3.1.3.74
Pyridoxine phosphate
Glycolysis
Glycolaldehyde
Pentose phosphate pathway
2.6.99.2
1.1.99.9
2-(Acetamidomethylene)-3-(hydroxymethyl)-3-succinate
5-Pyridoxolactone
3.5.1.66
1.14.12.5
3-Amino-2-oxopropyl phosphate
α-Hydroxymethyl succinate semialdehyde
3-Hydroxy-4-hydroxymethyl-2-methylpyridine-5-carboxylate
Isopyridoxal
spontaneous
(2S)-2-Amino-3-oxo-4-phosphono butyanoate
1.1.1.262
4-Phospho-D-erythronate
D-Erythrose 4-phosphate
1.2.1.72
1.1.1.290
2.6.1.52
4.2.3.1
2-Oxo-3-hydroxy-4-phosphobutanoate
O-Phospho-4-hydroxy-L-threonine
4-Hydroxy-L-threonine
Pentose phosphate pathway
00750 1/29/14
(c) Kanehisa Laboratories

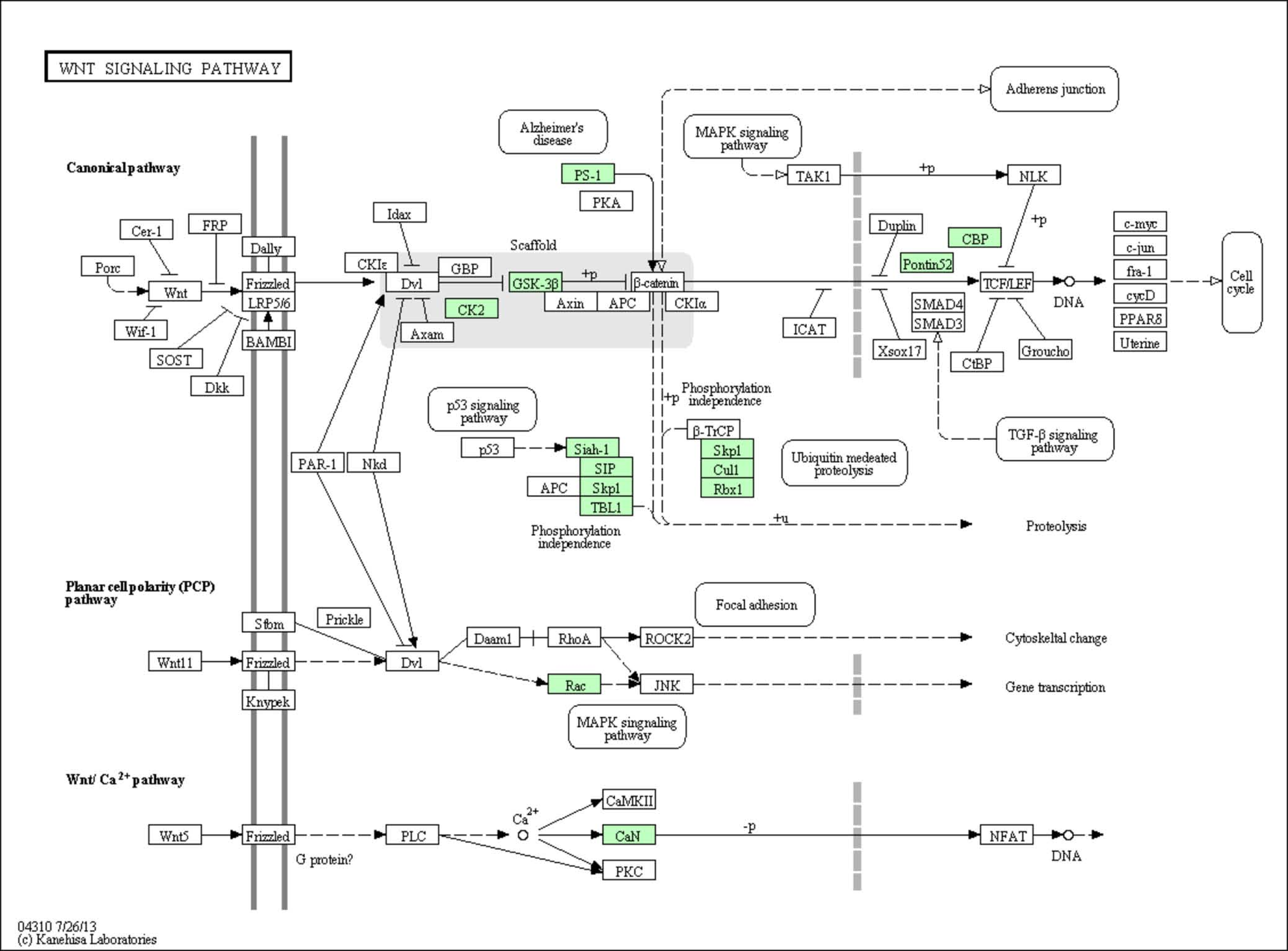
WNT SIGNALING PATHWAY
Canonical pathway
Porc
Cer-1
FRP
Dally
Wnt
Frizzled
LRP5/6
Wif-1
SOST
Dkk
BAMBI
Idax
CKIε
GBP
Dvl
Scaffold
GSK-3β
+p
β-catenin
Axin
APC
CKIα
CK2
Axam
Alzheimer's disease
PS-1
PKA
Adherens junction
MAPK signaling pathway
TAK1
+p
NLK
+p
Duplin
CBP
Pontin52
TCF/LEF
DNA
SMAD4
SMAD3
ICAT
Xsox17
CtBP
Groucho
c-myc
c-jun
fra-1
cycD
PPARδ
Uterine
Cell cycle
p53 signaling pathway
p53
Siah-1
SIP
APC
Skp1
TBL1
Phosphorylation independence
+p
Phosphorylation independence
β-TrCP
Skp1
Cul1
Rbx1
Ubiquitin mediated proteolysis
TGF-β signaling pathway
+u
Proteolysis
PAR-1
Nkd
Planar cell polarity (PCP) pathway
Sfbm
Prickle
Wnt11
Frizzled
Knypek
Dvl
Daam1
RhoA
ROCK2
Focal adhesion
Cytoskeltal change
Rac
JNK
Gene transcription
MAPK singnaling pathway
Wnt/ Ca2+ pathway
Wnt5
Frizzled
G protein?
PLC
Ca2+
CaMKII
CaN
-p
PKC
NFAT
DNA
04310 7/26/13
(c) Kanehisa Laboratories

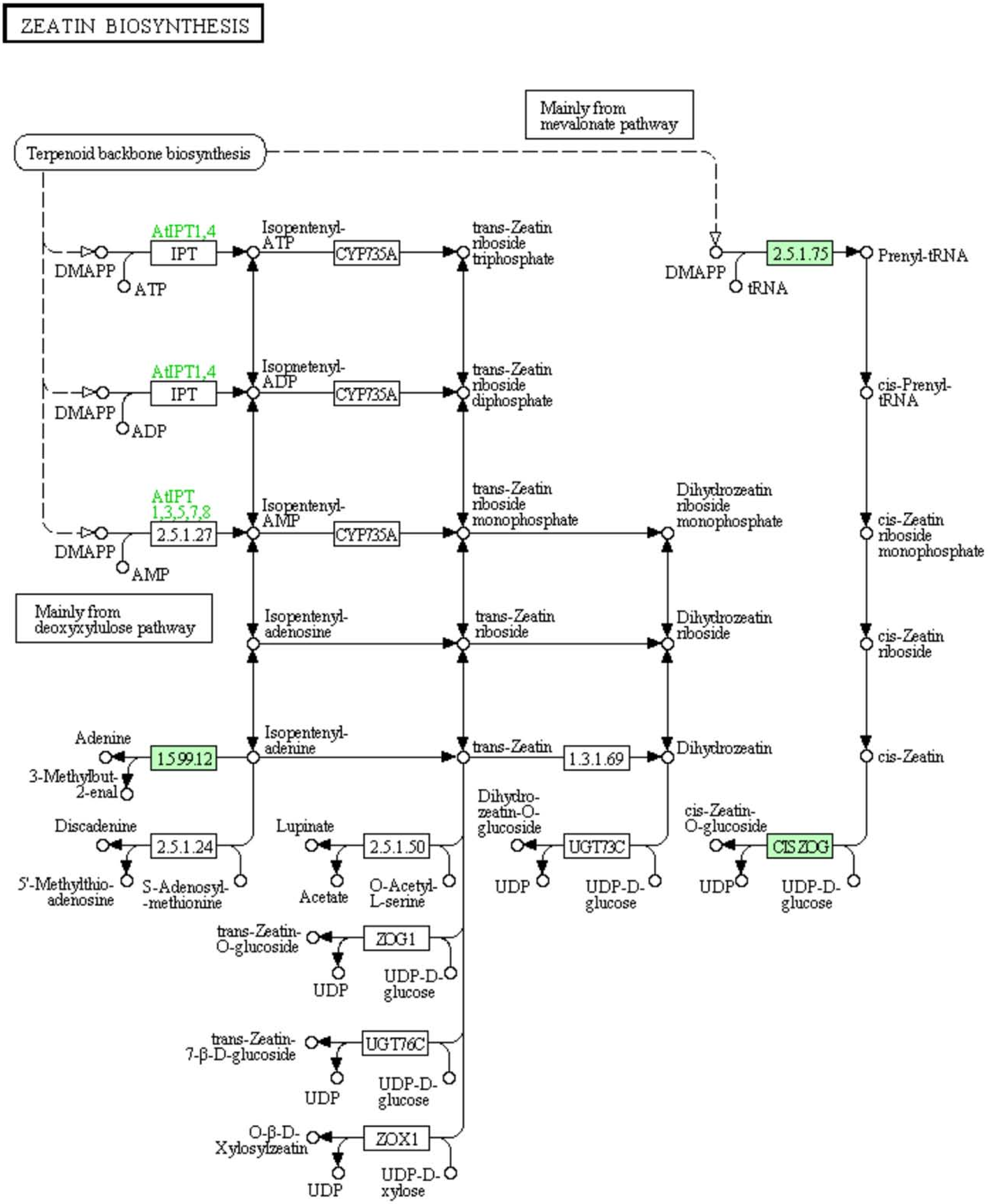

00908 6/22/12
(c) Kanehisa Laboratories
