# Supplementary material for: Exploring the Genes of Yerba Mate (Ilex paraguariensis A. St.-Hil.) by NGS and De Novo Transcriptome Assembly
Source: PLoS One. 2014 Oct 16;9(10):e109835. doi: 10.1371/journal.pone.0109835 (PMC4199719; doi:10.1371/journal.pone.0109835)
Supplement: Data S3 — Alternative language abstract in Spanish. (DOC) [file pone.0109835.s032.doc]

**Data S3** Alternative language abstract

**Explorando los genes de yerba mate (*Ilex paraguariensis* A. St.-Hil.) mediante NGS y ensamblado *de novo* de un Transcriptoma**

Humberto J. Debat^1†^, Mauro Grabiele^2,4†^, Patricia M. Aguilera^2,4^, Rosana E. Bubillo^3^, Mónica B. Otegui^4^, Daniel A. Ducasse^1^, Pedro D. Zapata^4^ and Dardo A. Marti^2,4*^

**^1^** Instituto de Patología Vegetal, Centro de Investigaciones Agropecuarias, Instituto Nacional de Tecnología Agropecuaria (IPAVE-CIAP-INTA), Córdoba, X5020ICA, Argentina. **^2^** Instituto de Biología Subtropical, Universidad Nacional de Misiones (IBS-UNaM-CONICET), Posadas, Misiones, 3300, Argentina. **^3^** Estación Experimental Cerro Azul, Instituto Nacional de Tecnología Agropecuaria (EEA Cerro Azul-INTA), Misiones, 3313, Argentina. **^4^** Instituto de Biotecnología de Misiones, Facultad de Ciencias Exactas Químicas y Naturales, Universidad Nacional de Misiones (INBIOMIS-FCEQyN-UNaM), Misiones, 3300, Argentina.

Yerba mate (*Ilex paraguariensis* A. St.-Hil.) es un importante árbol subtropical, cultivado en un área de 326.000 hectáreas en Argentina, Brasil y Paraguay, alcanzando una producción total anual de más de 1.000.000 de toneladas. Este cultivo presenta una fuerte limitación en cuanto a la disponibilidad de información genética de secuencias de ácidos nucleicos. El NCBI GenBank carece de una base de datos de bibliotecas de expresión (EST) de yerba mate y sólo incluye unas 80 secuencias de ADN de la especie, en su mayoría, no caracterizadas. En este escenario, con el objetivo de elucidar el repertorio de expresión de genes de yerba mate, mediante secuenciación de nueva generación (NGS), exploramos y descubrimos una vasta colección de transcriptos de *I. paraguariensis*. El ARN total de *I. paraguariensis* fue secuenciado con la plataforma Illumina HiSeqTM-2000 obteniéndose 72.031.388 secuencias de 100 pb. Las lecturas obtenidas de alta calidad fueron ensambladas *de novo* en 44.907 transcriptos, abarcando 40 millones de bases con una cobertura estimada de 180X. Múltiples análisis de secuencias nos permitieron inferir que la yerba mate contiene ~32.355 genes y 12.551 variantes de genes/isoformas. En líneas generales, se identificaron y categorizaron transcriptos pertenecientes a más de 100 vías metabólicas. Asimismo, hemos identificado ~1.000 factores de transcripción putativos, genes implicados en estrés por calor y estrés oxidativo, respuesta a patógenos, resistencia a enfermedades y respuesta a hormonas. También hemos identificado nuevos transcriptos relacionadas a estrés osmótico, sequía, salinidad, estrés por frío, senescencia y floración temprana. De igual forma encontramos varios miembros de la vía de silenciamiento génico, y caracterizamos el efector de silenciamiento Argonaute1. Predecimos *in silico* un diverso número de precursores putativos de microARNs que participan en procesos del desarrollo en vegetales. Hemos generado un borrador de los genomas transcritos de cloroplastos y mitocondrias de yerba mate. Se determinó la secuencia primaria y predijo la estructura tridimensional de la enzima responsable de la síntesis de cafeína de yerba mate. Finalmente, proveemos una colección de más de 10.800 microsatélites (SSR) accesibles a la comunidad interesada en el mejoramiento genético de la yerba mate. Esta contribución expande profundamente el limitado conocimiento de genes de yerba mate, y se presenta como el primer recurso genómico de este importante cultivo Sudamericano.
